# Sulfone Group as a Versatile and Removable Directing Group for Asymmetric Transfer Hydrogenation of Ketones

**DOI:** 10.1002/anie.202004658

**Published:** 2020-07-01

**Authors:** Vijyesh K. Vyas, Guy J. Clarkson, Martin Wills

**Affiliations:** ^1^ Department of Chemistry The University of Warwick Coventry CV4 7AL UK

**Keywords:** alcohols, asymmetric catalysis, reduction, ruthenium, sulfones

## Abstract

The sulfone functional group has a strong capacity to direct the asymmetric transfer hydrogenation (ATH) of ketones in the presence of [(arene)Ru(TsDPEN)H] complexes by adopting a position distal to the η^6^‐arene ring. This preference provides a means for the prediction of the sense of asymmetric reduction. The sulfone group also facilitates the formation of a range of reduction substrates, and its ready removal provides a route to enantiomerically enriched alcohols that would otherwise be extremely difficult to prepare by direct ATH of the corresponding ketones.

## Graphical abstract

**En route**: The sulfone group facilitates the formation of a range of reduction substrates through asymmetric transfer hydrogenation (ATH) of ketones, and its ready removal provides a route to challenging enantiomerically enriched alcohols.

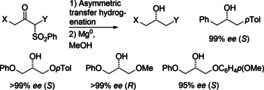

Asymmetric transfer hydrogenation (ATH) is a highly practical method for the synthesis of enantiomerically enriched alcohols as it is effective under mild reaction conditions and avoids the need for the use of high‐pressure hydrogen gas.^1^ The [(arene)Ru(TsDPEN)Cl] class of catalysts first reported by Noyori et al. (e.g. **1**, Figure 1) are very efficient in this application, and reduce many classes of ketones, notably acetophenone derivatives and progargylic ketones, with high *ee* values.^2^ We, and others, have reported “tethered” derivatives of the Noyori catalysts (e.g. **2**, Figure 1) which in some applications exhibit higher activities and stabilities.^3^ Catalysts such as **1** and **2** have been employed in ATH for the synthesis of pharmaceutically relevant targets,^4^ including multikilo applications,4a and in high‐volume flow chemistry.4b The triflate derivative **3**, which readily ionizes in methanol, has been employed in closely related asymmetric hydrogenation (AH) of ketones.^5^

**Figure 1:**
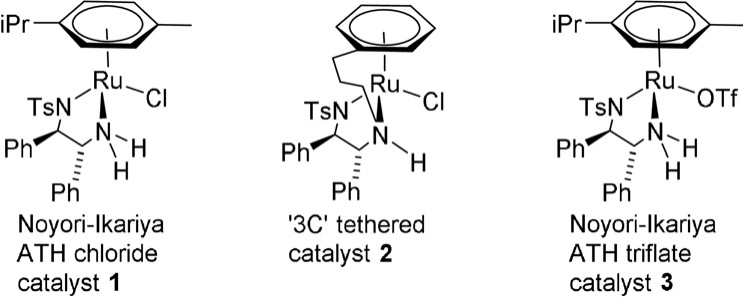
The Noyori–Ikariya catalyst **1**, the 3C tethered derivative **2**, and the Noyori–Ikariya triflate‐derived catalyst **3**. The active catalysts are generated through HCl elimination when the catalysts are activated.

However, despite its high value and practicality, this class of catalyst does not work well for all substrates. For example, in the asymmetric reduction of alkyl/alkyl ketones or substrates with minimal electronic or steric differences between the groups flanking the ketone,^6^ the catalysts are less enantioselective. To address this shortcoming, we investigated the use of a temporary directing group to influence the selectivity of a reduction, followed by its removal, to “unmask” what would previously have represented a very difficult target for ATH. In this regard, sulfones seemed promising since they have been reported to be effective partners for ATH reactions (Figure 2). Zhang et al. reported, in 2009, the dynamic kinetic resolution (DKR)/ATH of cyclic α‐sulfone‐substituted ketones (**4**) with the catalyst (*S*,*S*)‐**1** to give the products **5** with high dr and *ee* values (Figure 2 A).^7^ Cyclic examples, including the conversion of **6** into **7**, were exceptionally selective (Figure 2 B).^7^ In 2009, Wang et al. reported the use of (*R*,*R*)‐**3** for asymmetric hydrogenation (AH) of α‐sulfonyl and α‐sulfonamidyl ketones (**8**; Figure 2 D) to the alcohols **9** and the DKR/AH of related cyclic substrates.^8^ The majority of examples contained an aryl substituent and gave greater than 90 % *ee*, although the reduction of EtCOCH_2_SO_2_Ph in 84 % ee^8^/80 % ee^9^ and MeCOCH_2_SO_2_Ph in 82 % ee^8^/91 % *ee* 9 were also reported.^8^ Later reports featured a one‐pot formation, and then ATH, of sulfone‐substituted ketones under aqueous conditions,^9^, ^10^ and the application of a silica‐supported variant.^11^ Bhanage and Vyas reported DKR/ATH of cyclic α‐sulfone ketones using a proline‐derived catalyst.^12^

**Figure 2:**
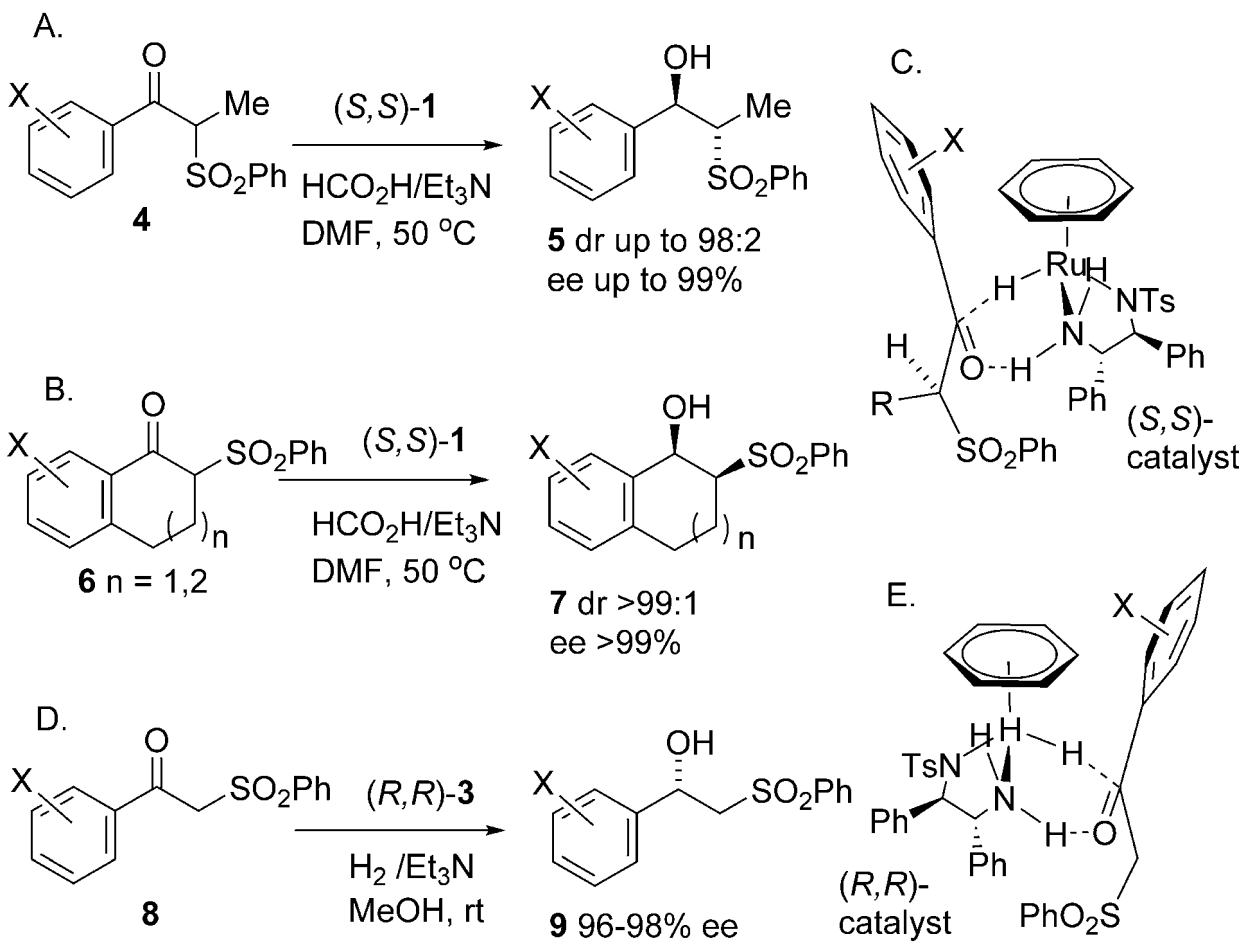
Reported classes of asymmetric reductions of sulfone‐substituted ketones. A) DKR/ATH of acyclic substrates. B) DKR/ATH of cyclic substrates. C) Mode of hydride transfer for (A) and (B). D) AH of sulfone‐substituted acetophones. E) Mode of hydride transfer for (D).

Zhang et al. reported the reduction of α‐sulfonamide ketones with very high *ee* values and conversion using the three‐carbon (3C) tethered catalyst **2**.^13^ Asymmetric hydrogenation of sulfone‐containing acetophenone derivatives has also been reported using other catalysts including Ru/diphosphine catalysts, and CBS reagents.14a, 14b, 14c, 14d

In reductions with the [(arene)Ru(TsDPEN)Cl] complexes **1**–**3**, where acetophenone derivatives are most commonly studied, the sense of reduction indicates that the sulfone adopts a position in the transition state for ATH^15^ in which it is positioned distal from the η^6^‐arene ring on the ruthenium hydride, which is the active catalyst form in the reaction (Figures 2 C and E). The η^6^‐arene is presumed to engage in a productive electrostatic interaction with the aromatic substituent on the substrate.

In contrast, very few reductions of alkyl‐containing, α‐sulfonyl ketones have been reported, possibly because of the perceived lack of fit to the traditional “acetophenone‐based” reduction model.^15^ The potential for the use of a sulfone as a temporary directing group prompted us to examine what range of substituents could successfully partner with a sulfone and whether it could hence be used as a removable group to facilitate the synthesis of otherwise challenging alcohol target molecules (Figure 3).

**Figure 3:**
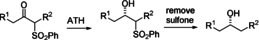
Investigations in this report, and strategy for synthesis of challenging alcohols with high *ee* values.

## Results and Discussion

**Figure 4:**
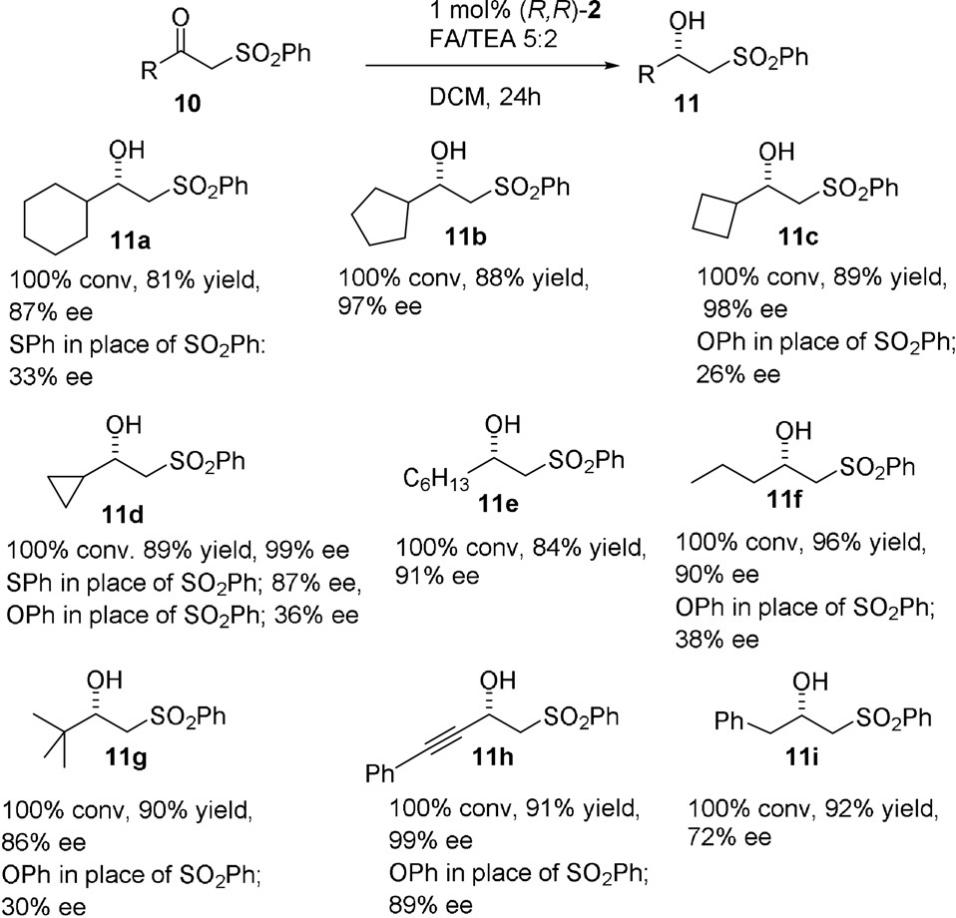
ATH of α‐sulfonyl ketones with a tethered ATH catalyst and the resulting products **11 a**–**i**, and comparisons to *ee* values of corresponding ATH products containing sulfides and ethers in place of the sulfone.

We first prepared a diverse range of α‐sulfonyl ketones (**10 a**–**i**) and studied their reductions using (*R*,*R*)‐**2** (Figure 4).^3^ The majority of these ketones were prepared from the bromoketone using PhSO_2_Na. Racemic **11 h** was prepared by addition of PhSO_2_CH_2_ anion to PhCCCHO, and subsequently oxidised to the ketone **10 h**. The ketone precursor to **11 i** (i.e., **10 i**) was prepared by addition of PhSO_2_CH_2_ anion to the Weinreb amide PhCH_2_CONMe(OMe).

The ATH [using formic acid/triethylamine (FA/TEA) 5:2 azeotrope] of **10 a**–**d** revealed an unexpected trend in which the product *ee* value increased as the ring became smaller in the series from cyclohexyl (**11 a**, 87 % *ee*) to cyclopropyl (**11 d**, 99 % *ee*), although it remained high in each case (Figure 4). In representative cases, the results were compared with those for the ATH of the analogous thiol‐ or ether‐containing substrates. It was found that these were consistently reduced in lower enantioselectivity in every case. The reduction of the sulfone‐containing cyclopropyl ketone **10 d** gave **11 d** in a very high 99 % *ee*, whereas the thiophenyl‐substituted analogue gave a product with a reasonable *ee* value of 87 % and the phenoxy substrate gave a product of just 36 % *ee*. Substrates containing sulfones and linear alkyl chains (**10 e**, **10 f**) were also reduced. The product *ee* values increased with the length of the chain and were higher than for the reported reductions of substrates containing Me and Et substituents. Even a substrate containing a hindered *t*‐butyl group, that is, **10 g**, was reduced in a valuable 86 % *ee*. The substrate containing a triple bond, **10 h**, was reduced in particularly high enantioselectivity to **11 h** (99 % *ee*) although the benzyl‐substituted substrate **10 i** gave **11 i** with just 72 % *ee*.

**Figure 5:**
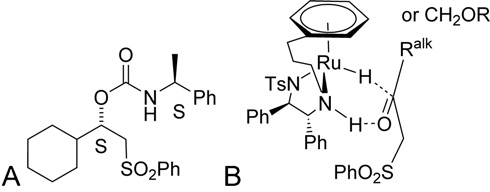
A) Structure of **11 a** functionalised using (*S*)‐1‐phenylethylisocyate (X‐ray structure is in the Supporting Information). B) Proposed mode of reduction of sulfone‐substituted alkyl ketones.

A derivative of the major enantiomer of the cyclohexyl‐containing reduction product **11 a** was prepared through reaction with (*S*)‐1‐phenylethylisocyate and the X‐ray crystallographic structure of this product (Figure 5 A) led to the product configuration assignment of *S*, in agreement with the comparison of the optical rotation to that reported (see the Supporting Information) and also with the reported precedents.^7^, ^8^, ^9^, ^10^, ^11^, ^12^, ^13^ The configurations of **11 f**, the OPh derivative of **11 f**, and of **11 i** were also confirmed by optical rotation comparisons with those reported and the other products were assigned by analogy.

The results suggest that the sense of reduction of the examples follows the model for the earlier‐reported (predominantly aromatic) substrates, that is, in which the sulfone group favours the position in the transition state in which it is distal from the η^6^‐arene of the complex (Figure 5 B).^7^, ^8^, ^9^, ^10^, ^11^, ^12^, ^13^

**Figure 6:**
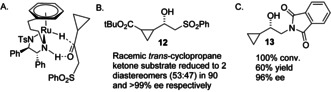
Cyclopropyl‐functionalised ketones are also excellent substrates for ATH reactions. A) Likely mode of hydrogen transfer by ATH using (*R*,*R*)‐**2**. B) Ester‐functionalized cyclopropane ATH product **12**. C) Phthalimide‐containing ATH product **13** (ATH conditions as given in Figure 4).

The cyclopropyl group is a particularly compatible substituent in substrates for ATH reactions, with reports having been published of applications to natural product synthesis in which ketones adjacent to cyclopropanes are reduced with high *ee* values.^16^ The sense of induction suggests that it is compatible with an interaction with the η^6^‐arene, which accords with our results (Figure 6 A). We also found that the product **12** was formed as a 53:47 mixture of two enantiomerically enriched diastereosiomers (90 and >99 % *ee*, respectively) through reduction of the racemic *trans*‐cyclopropane substrate (Figure 6 B). Another example of a related ketone, bearing a phthalimide group (and hence a precursor to 2‐hydroxy amines) was reduced in 96 % *ee* by ATH to **13** (Figure 6 C) to further highlight the value of the cyclopropyl group.

**Figure 7:**
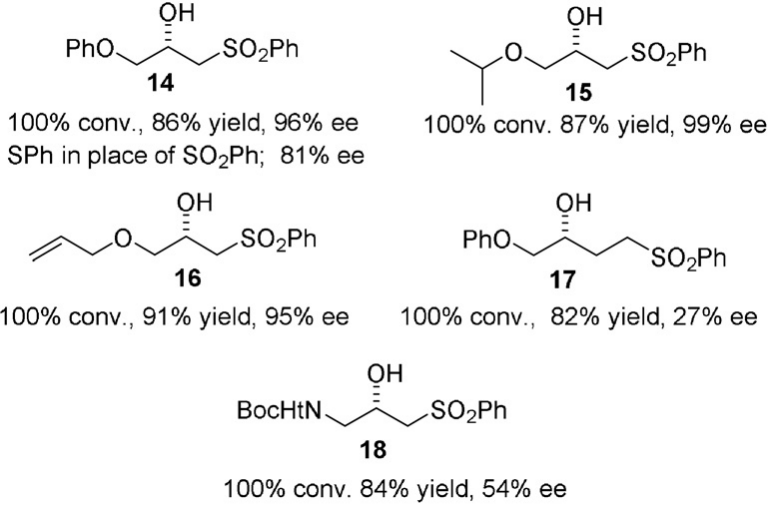
ATH products formed from alkoxy‐ and amino‐substituted ketone substrates containing sulfone groups (ATH conditions as given in Figure 4).

Encouraged by these results, we investigated further derivatives with more challenging functionalisation. Previous studies in our group had revealed that aryloxy and alkoxy groups can adopt positions adjacent to the η^6^‐arene of the catalyst during reductions and it seemed that the pairing of these with a sulfone could create an ideal substrate for reduction. In the event, we found that oxygen‐containing groups were tolerated well in ATH substrates. PhOCH_2_COCH_2_SO_2_Ph was reduced to **14** in 96 % *ee* and two related ketones were also converted in similarly high enantioselectivity into **15** and **16** (Figure 7). Again, a corresponding sulfide‐containing substrate was reduced in lower *ee* (81 % *ee*), underlining the importance of the sulfone group to the control of the reduction (Figure 5 C). A sulfone group one carbon further away from the ketone was less effective at directing the reaction, and the product **17** of 27 % *ee* was formed. The reduction of a substrate containing a *t*Boc‐ protected amine, as opposed to a sulfone, gave the product **18** with just 53 % *ee*, however.

**Figure 8:**
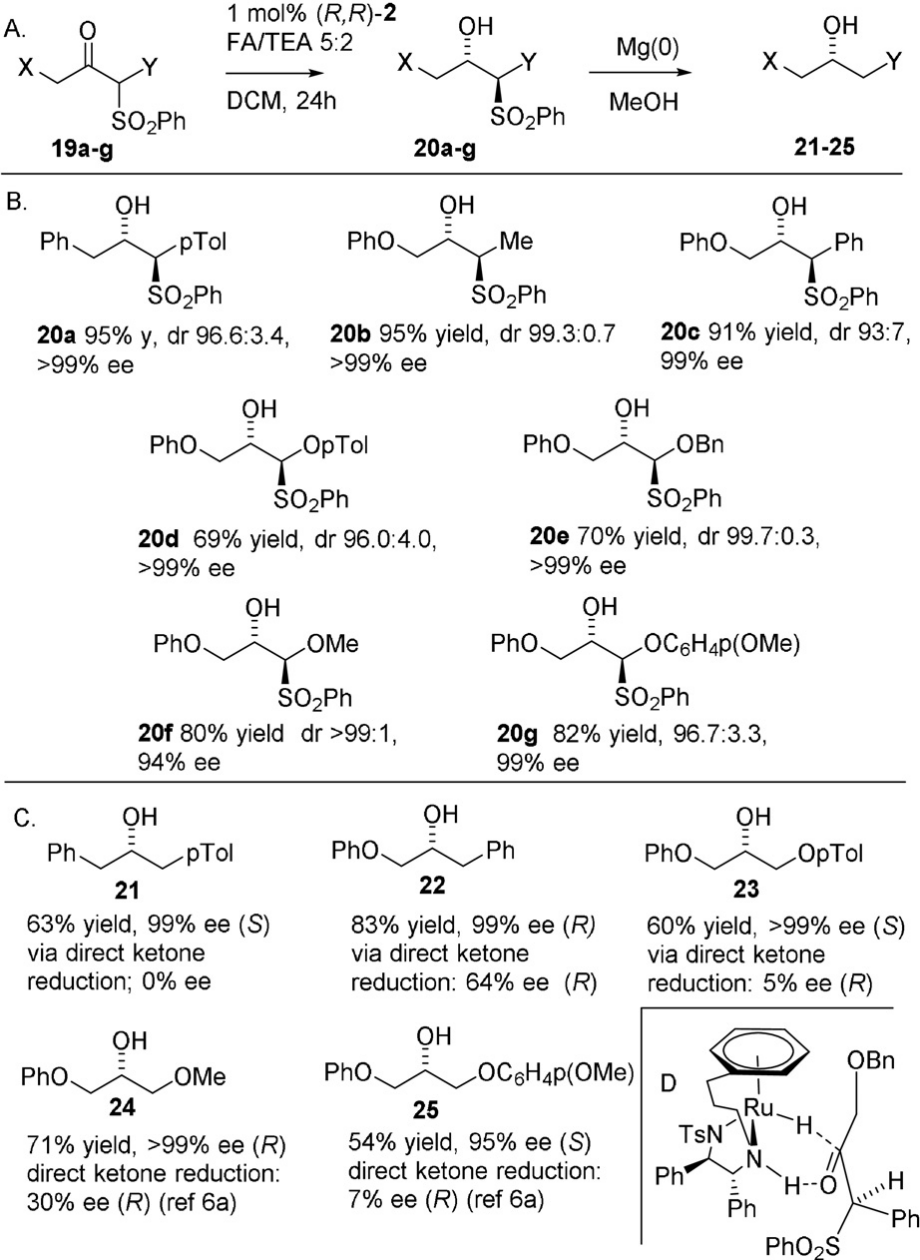
A) Diastereoselective ATH products with DKR where a racemisable center was present in the substrate. B) ATH/DKR products. The product *ee* value is in each case of the major diastereoisomer. The products **20 a**–**g** were formed in 100 % conversion. C) The products **21**–**25** of sulfone reduction. D) Proposed mode of reduction of **19 a**–**g** in the ATH/DKR reaction.

With the directing factors established, the dynamic kinetic resolution (DKR) of sulfone‐containing substrates (**19 a**—**g**) was examined next. This reaction also proved successful, with **20 a**–**g** being obtained with high dr and *ee* values, that is, where the substituent was adjacent to the ketone and able to racemize rapidly to facilitate the DKR process (Figure 8 A and 8B). The absolute stereochemistry of **20 c** was confirmed by X‐ray crystallography (see the Supporting Information) and others were assigned by analogy. In these examples, the starting materials **19 a**– **g** were prepared by addition of the corresponding sulfone anion to the precursor aldehyde followed by oxidation. In all cases, the sense of reduction to **20 a**–**g** followed that in the model previously described (Figure 5 B) with the additional requirement for the avoidance of steric clashes between the substituent adjacent to the sulfone and the catalyst (Figure 8 D), hence the preferred reduction of the *R*‐configured enantiomer of the substrate when (*R*,*R*)‐**2** was used.

**Figure 9:**
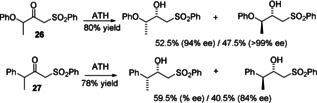
Attempted ATH/DKR where a racemisable centre was not present in the substrate. ATH conditions are as given in Figure 8. Relative stereochemistry was not confirmed.

As previously outlined, this transformation provides access to a strategy for the synthesis of otherwise highly challenging asymmetric alcohol products with high *ee* values. To demonstrate this, we reductively removed the sulfone group^17^ from a representative number of products to generate the unsubstituted products **21**–**25** with high *ee* values, which in each case was an alcohol with very little steric or electronic difference between the groups flanking it (Figure 9 C). Traditionally such targets would be regarded as very difficult to prepare through direct ketone ATH. For each case, comparisons of enantioselectivities of reductions of the direct ketone precursors are included for comparison (see the Supporting information). The product **21** was formed as a racemate by direct ketone reduction, and **23** was formed in just 5 % *ee*. Direct reduction of the ketone precursor to **22** gave the best result of 64 % *ee*, and in the same sense, resulting from the electronic difference between phenyl and phenoxy substituents. The compounds **24** and **25** have previously been reported by us,6a with *ee* values of just 30 % and 7 % *ee*, respectively, obtained by direct ketone reduction. In the case of **25**, the opposite enantiomer of alcohol is formed by direct reduction (using the same configuration of catalyst), thus serving to confirm that the absolute sense of reduction of **19 g** matches that predicted by the model in Figure 8 D. However, by proceeding through the sulfone intermediate, products of greater than 99 % *ee* (**24**) and 95 % *ee* (**25**) were obtained. The sharp contrast illustrates how a sulfone acts as a temporary group for achievement of the required syntheses. Attempted removal of the sulfone from **20 e** did not yield the required alcohol product, possibly because of debenzylation and decomposition.

In cases where the existing chiral center was located distal from the sulfone, that is, in **26** and **27**, a DKR was not achieved, and both product diastereoisomers were formed, with variable results (Figure 9) and some differences between the matched and mismatched substrate/catalyst enantiomer combinations.

**Figure 10:**
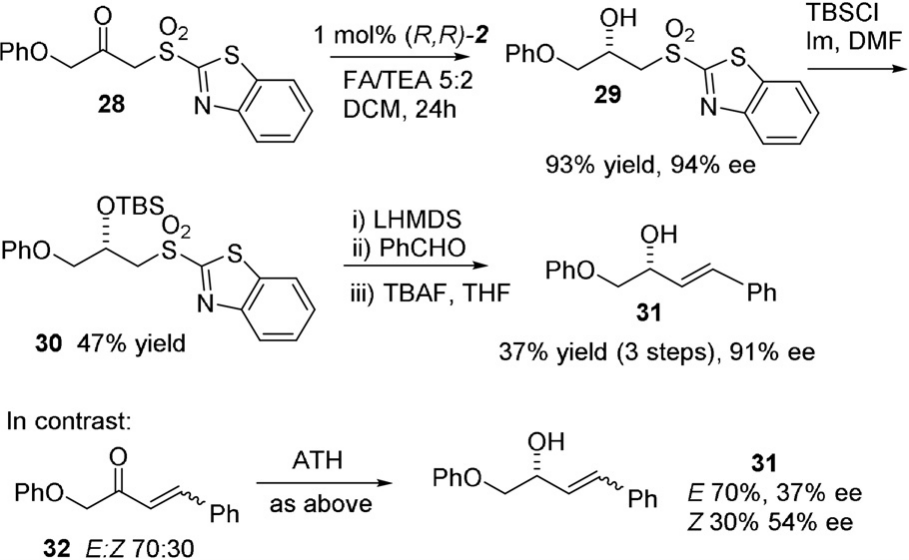
Formation of allylic alcohols with high *ee* values by Julia–Kocienski coupling post ATH‐DKR.

In an extension of this strategy (Figure 10), an allylic alkene was prepared through reduction of the heterocyclic sulfone **28** (prepared from the anion of 2‐(methylsulfonyl)benzo[d]thiazol‐6‐ylium and 2‐phenoxyacetyl chloride) to the alcohol **29** in 94 % *ee*. Protection of the alcohol gave **30** and this step was followed by a Julia–Kocienski olefination reaction with benzaldehyde and deprotection to give (*E*)‐**31** in 91 % *ee*.^18^ The analogous compound without the sulfone (**32**) was prepared as a mixture of *E*/*Z* isomers in a 70:30 ratio (see the Supporting Information) and was reduced in much lower enantiomeric excess in contrast (two alkene isomers of **31** in just 37 and 54 % *ee*, respectively), again demonstrating the added value of the sulfone group in the generation of a practical and selective route to a product that would otherwise be extremely difficult to prepare in high enantiomeric excess.

## Conclusion

In conclusion, we have demonstrated that the sulfone group is a powerful tool for directing efficient asymmetric reductions and provides an access to products which would otherwise be very difficult to generate in high enantiomeric excess through direct reduction.

**Supporting Information**: The Supporting Information is available free of charge on the ACS Publications website. Experimental procedures, NMR spectra, X‐ray crystallographic data and HPLC data (PDF).

**Data sharing statement**. The research data (and/or materials) supporting this publication can be accessed at http://wrap.warwick.ac.uk/TBA.

## Conflict of interest

The authors declare no conflict of interest.

## Supporting information

As a service to our authors and readers, this journal provides supporting information supplied by the authors. Such materials are peer reviewed and may be re‐organized for online delivery, but are not copy‐edited or typeset. Technical support issues arising from supporting information (other than missing files) should be addressed to the authors.

Supplementary
