# Supplementary material for: Sulfone Group as a Versatile and Removable Directing Group for Asymmetric Transfer Hydrogenation of Ketones
Source: Angew Chem Int Ed Engl. 2020 Jul 1;59(34):14265–9. doi: 10.1002/anie.202004658 (PMC7496949; doi:10.1002/anie.202004658)
Supplement: Supplementary file 1 — Supplementary [file ANIE-59-14265-s001.pdf]

Angewandte Chemie
Eine Zeitschrift der Gesellschaft Deutscher Chemiker

Supporting Information

Sulfone Group as a Versatile and Removable Directing Group for Asymmetric Transfer Hydrogenation of Ketones

*Vijyesh K. Vyas, Guy J. Clarkson, and Martin Wills**

anie_202004658_sm_miscellaneous_information.pdf

<u>Index</u>

**General procedures for the syntheses**	**S2**
**Data for Ketones and Alcohols**	**S6**
**NMR spectra and HPLC data**	**S64**
**X-ray crystal structure details.**	**S217**

General procedures for the syntheses.

Solvents and reagents for the synthesis of complexes and catalytic reactions were degassed prior to use and all reactions were carried out under either a nitrogen or argon atmosphere. Reactions were monitored by TLC using aluminum backed silica gel 60 (F254) plates, visualized using UV 254 nm and phosphomolybdic acid (PMA), potassium permanganate or vanillin dips as appropriate. Flash column chromatography was carried out routinely using 60 micrometer silica gel. Reagents were used as received from commercial sources unless otherwise stated. FA/TEA (5:2) was bought from Sigma-Aldrich and used as received. ^{1}H NMR spectra were recorded on a Bruker DPX (300, 400 or 500 MHz) spectrometer. Chemical shifts are reported in δ units, parts per million relative to the singlet at 7.26 ppm for chloroform and 0.00 ppm for TMS. Coupling constants (J) are measured in Hertz. IR spectra were recorded on a Perkin-Elmer Spectrum One FT-IR Golden Gate. Mass spectra were recorded on a Bruker Esquire2000 or a Bruker MicroTOF mass spectrometer. Melting points were recorded on a Stuart Scientific SMP 1 instrument and are uncorrected. GC analysis was performed using a Hewlett Packard 5890. Dry solvents were purchased and used as received. HPLC analyses on a Hewlett-Packard 1050 instrument. Optical rotations were measured on an AA-1000 polarimeter. The details of the X-ray instrument are given in the X-ray crystallography section. Enantiomeric excesses in this Supporting Information are measured to one decimal place, however in the paper, these have been rounded to whole numbers or to >99% ee where the measured ee was 99.5% or above.

Synthesis of bromoketones

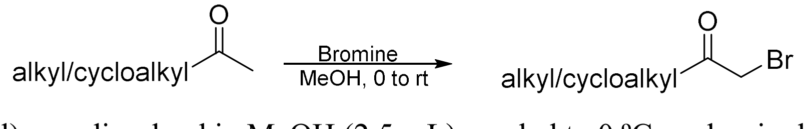

Ketone (1 mmol) was dissolved in MeOH (2.5 mL), cooled to 0 ºC, and a single portion Br_2 (1 mmol) added, and the resulting mixture stirred at a temperature below 15 ºC for 3 h. Water (5 mL) was then added and stirring continued for a further 15 h at room temperature. The reaction mixture was then extracted with ethyl acetate (15 mL), and the organic extracts were dried and evaporated (procedure adapted from Gheorghe, A.; Quiclet-Sire, B.; Vila, X.; Zard, S. Z.*Org. Lett*. **2005,** *7*, 1653–1656).

Procedure A: Synthesis of sulfone Ketones.

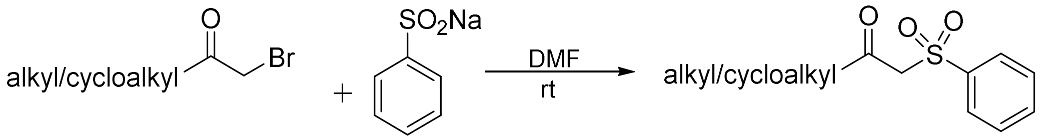

The bromoketone (1.0 mmol) was dissolved in dimethylformamide (DMF 5 mL) under nitrogen at 0 °C, then $PhSO_2Na$ (1.2 mmol) was added. The mixture was stirred vigorously at room temperature until complete consumption of the substrate. The mixture was quenched with water (10 mL) and extracted with EtOAc (15 mL). The organic layer was washed with brine (15 mL), evaporated and purified by flash chromatography on silica gel to give β-arylketosulfones. Procedure adapted from Yang, D.-T.; Meng, Q.-Y.; Zhong, J.-J.; Xiang, M.; Liu, Q.; Wu, L.-Z. *Eu. J. Org. Chem.,* 2013, *33*, 7528-7532.

Procedure B: Synthesis of ether/thioether Ketones.

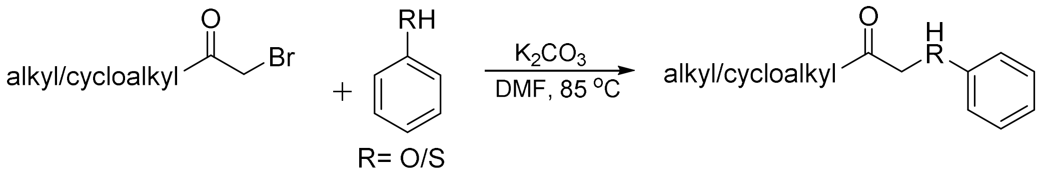

The bromoketone (1.0 mmol) was dissolved in DMF (5 mL) under nitrogen at rt under moisture free condition, then phenol/thiophenol (1.2 mmol) and K_2CO_3 (1.5 mmol) was added and stirred overnight at 85 °C. The mixture was quenched with H_2O (5 mL) and extracted with EtOAc (15 mL). The organic layer was washed with brine (15 mL), evaporated and purified by flash chromatography on silica gel to give β-arylketoethers. Procedure adapted from Janin, Y.-L.; Guillou, S.; Lucas-Hourani, M.; Munier-Lehmann, H.; Noel, A.; Salanouve, E.; Tangy, F.; Vidalain, P.-O. WO2015/155680, 2015, A2.

Procedure C: Synthesis of Racemic Alcohols.

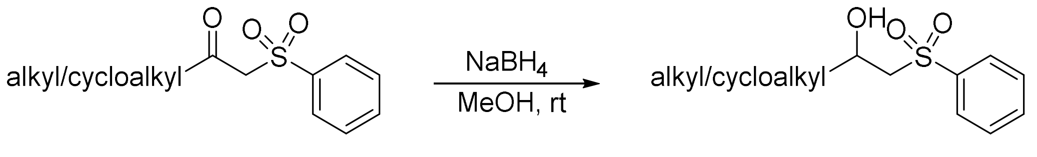

The Ketone (1 mmol) was dissolved in MeOH (5 mL) at rt and $NaBH_4$ (1.6 mmol) was added portion wise over 10 minute. The reaction was allowed to stir at rt for 2h. After the completion of reaction, it was quenched by water (5 mL) and extracted with ethyl acetate (12 mL). The organic layer was dried with Na_2SO_4 and concentrated to obtain a residue which is purified by flash chromatography on silica gel.

Procedure D: Asymmetric Transfer Hydrogenation (ATH) of ketones.

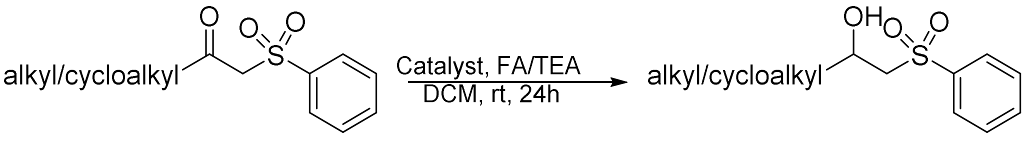

The ketone (0.5 mmol), catalyst (1 mol%), DCM (3 mL) and FA/TEA (0.5 mL) azeotrope was added sequentially to the reaction tube and stirred at rt. The reaction was monitored by TLC. After the completion of reaction, it was quenched by water (3 mL) and extracted with ethyl acetate (8 mL). The organic layer was dried with Na_2SO_4 and concentrated to obtain a residue. The residue was purified with a silica gel column eluted with petroleum ether and ethyl acetate to obtain the pure desired product.

Procedure E: General procedure to synthesize 1-alkoxy-3-(phenylsulfonyl)propan-2-ol

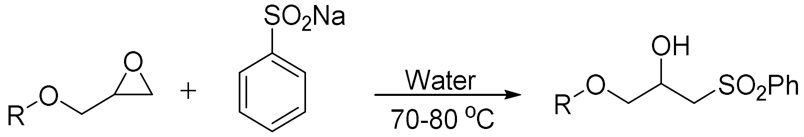

To a flask containing the epoxide (1 mmol) in water (20 mL), sodium sulfinate (1.1 mmol) was added. The suspension was magnetically stirred at 70–80 °C until reaction was complete. After completion of the reaction, the reaction mixture was extracted with ethyl acetate (20 mL). The extract was further washed with water (10 mL) and saturated brine solution (10 mL), dried over anhydrous Na_2SO_4, and evaporated under reduced pressure to give β-hydroxy sulfones. The product was further purified by silica gel column chromatography using ethyl acetate and hexane as an eluent.

Procedure F: General procedure to synthesize 1-alkoxy-3-(phenylsulfonyl)propan-2-one

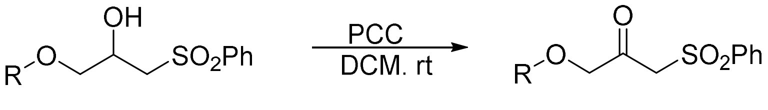

To a flask containing the 1-alkoxy-3-(phenylsulfonyl)propan-2-ol (1 mmol) in DCM (10 mL) was added PCC (2 mmol) and stirred at rt for 48 hours. After completion of the reaction, the reaction mixture was filtered through celite and filtrate was concentrated on vacuo. The crude mass was further purified by silica gel column chromatography using ethyl acetate and hexane as an eluent.

Procedure G: **General procedure for sulfone reduction**

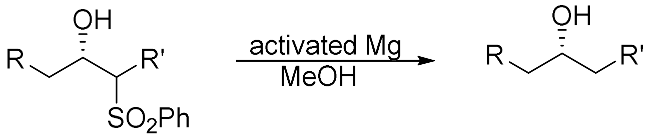

A suspension of activated magnesium turnings (30.0 mmol) and sulfone (1.0 mmol) in MeOH (10 mL) was stirred at room temperature for 1 h. The suspension was filtered through celite, the filter cake was washed with MeOH (50 mL), and the filtrate was diluted with saturated aqueous NH_4Cl solution (20 mL) and extracted with ethyl acetate (20 mL). The organic layers were dried ($MgSO_4$), filtered and concentrated in vacuo and purified by column chromatography on silica gel (pet ether/ EtOAc) to yield alcohol.

Data for Ketones and Alcohols

1-Cyclohexyl-2-(phenylsulfonyl)ethan-1-one 10a

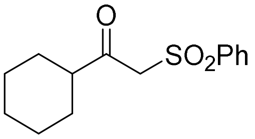

This compound is known and has been fully characterized:

Thomsen, M. W.; Handwerker, B. M.; Katz, S. A.; Belser, R. B. *J. Org. Chem.* **1988**, *53*, 906-907.

This compound was prepared following procedure A using: 2-bromo-1-cyclohexylethan-1-one (1.0 g, 4.8 mmol, 1.0 equiv), benzene sulphinic acid (1.04 g, 6.30 mmol, 1.3 equiv) and dry DMF (15 mL). 1-cyclohexyl-2-(phenylsulfonyl)ethan-1-one was isolated by flash chromatography (pet ether/ EtOAc: 85:15) as a colourless oil (432 mg, 1.6 mmol, 33.3%).

^{1}H NMR (400 MHz, $CDCl_3$) δ 7.90 (d, *J* = 7.6 Hz, 2H, ArH), 7.68 (t, *J* = 7.2 Hz, 1H, ArH), 7.58 (t, *J* = 7.3 Hz, 2H, ArH), 4.21 (s, 2H, CH_2), 2.64 (s, 1H, CH), 1.95 – 1.61 (m, 5H, CH), 1.37 – 1.13 (m, 5H, CH).

^{13}C NMR (101 MHz, $CDCl_3$) δ 201.22, 138.97, 134.19, 129.27, 128.40, 64.78, 51.51, 27.89, 25.62, 25.32.

m/z (ESI) 289.2 ([M + Na]+, 100 %).

Racemic and (*S*)-1-cyclohexyl-2-(phenylsulfonyl)ethan-1-ol 11a

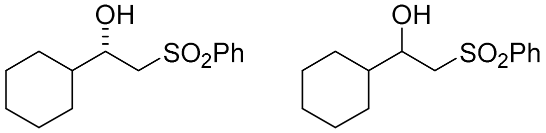

This compound is known and has been fully characterized:

Wan, X.; Meng, Q.; Zhang, H.; Sun, Y.; Fan, W.; Zhang, Z. *Org. Lett*, **2007**, *9*, 5613-5616.

This compound was prepared in racemic form following procedure C using: 1-cyclohexyl-2-(phenylsulfonyl)ethan-1-one (200 mg, 0.752 mmol, 1.0 equiv), $NaBH_4$ (42 mg, 1.1 mmol, 1.6 equiv) and MeOH (5 mL). 1-cyclohexyl-2-(phenylsulfonyl)ethan-1-ol was isolated by flash chromatography (pet ether/ EtOAc: 85:15) as a colourless oil (171 mg, 0.638 mmol, 84.8%).

This compound was prepared in enantiomerically-enriched form following procedure D, using 1-cyclohexyl-2-(phenylsulfonyl)ethan-1-one (100 mg, 0.376 mmol, 1.0 equiv), FA/TEA (0.3 mL), [(*R,R*)Teth-TsDpenRuCl] (2.3 mg, 3.7 x 10^{-3} mmol, 1 mol%) and DCM (2 mL). (*S*)- 1-cyclohexyl-2-(phenylsulfonyl)ethan-1-ol was isolated by flash chromatography (pet ether/ EtOAc: 85:15) as a colourless oil (82 mg, 0.30 mmol, 81%).

$[\alpha]_D^{25}$ +17.5° (c 0.1 in $CHCl_3$) 86.6 % ee (*S*) (lit(above) $[\alpha]^D$ +10.6° (c 1.5 in $CHCl_3$, 90% ee (*S*).

^{1}H NMR (400 MHz, $CDCl_3$) δ 7.87 (d, *J* = 7.6 Hz, 2H), 7.61 (t, *J* = 7.2 Hz, 1H), 7.52 (t, *J* = 7.3 Hz, 2H), 3.88 (d, *J* = 4.4 Hz, 1H), 3.17 (s, 1H), 3.15 (d, *J* = 6.1 Hz, 2H), 1.73 – 1.50 (m, 5H), 1.39 – 1.28 (m, 1H), 1.17 – 0.87 (m, 5H).

^{13}C NMR (101 MHz, $CDCl_3$) δ 139.34, 134.00, 129.45, 127.91, 69.80, 60.32, 43.17, 28.50, 27.53, 26.23, 25.98, 25.89.

m/z (ESI) 291.2 ([M + Na]+, 100 %).

Enantiomeric excess determined by HPLC analysis (CHIRALCEL OD-H column, hexane 93:07 iPrOH, 0.7 mL/min, T = 25°C, λ = 210 nm, Ketone 18.8 min, *R* enantiomer 26.2 min, *S*-enantiomer 29.3 min). 86.6% ee (*S*).

1-Cyclopentyl-2-(phenylsulfonyl)ethan-1-one 10b

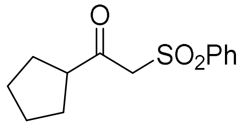

This compound is novel.

This compound was prepared following procedure A using: 2-bromo-1-cyclopentylethan-1-one (400 mg, 2.10 mmol, 1.0 equiv), benzene sulphinic acid (414 mg, 2.52 mmol, 1.2 equiv) and dry DMF (8 mL). 1-cyclopentyl-2-(phenylsulfonyl)ethan-1-one was isolated by flash chromatography (pet ether/ EtOAc: 85:15) as a white solid (365 mg, 1.44 mmol, 68.4%).

mp 69-70 °C

(found (ESI) [M+Na]+, 275.0714. $C_{13}H_{16}NaO_3S$ requires 275.0712)

ν_{max}: 2958, 2905, 1713, 1446, 1305, 1143, 1028, 760, 686, 552, 518 cm^{-1}.

^{1}H NMR (400 MHz, $CDCl_3$) δ 7.90 (d, *J* = 7.5 Hz, 2H), 7.68 (t, *J* = 7.3 Hz, 1H), 7.58 (t, *J* = 7.4 Hz, 2H), 4.22 (s, 2H), 3.25 – 3.11 (m, 1H), 1.87 – 1.54 (m, 8H).

^{13}C NMR (101 MHz, $CDCl_3$) δ 200.51, 138.96, 134.20, 129.28, 128.40, 65.91, 52.52, 28.49, 25.87.

m/z (ESI) 275.2 ([M + Na]+, 100 %).

Racemic and (*S*)-1-cyclopentyl-2-(phenylsulfonyl)ethan-1-ol 11b

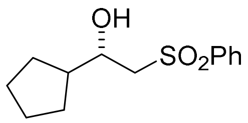

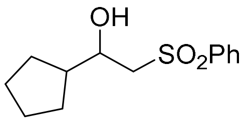

This compound is novel.

This compound was prepared in racemic form following procedure C using: 1-cyclopentyl-2-(phenylsulfonyl)ethan-1-one (100 mg, 0.397 mmol, 1.0 equiv), $NaBH_4$ (22 mg, 0.58 mmol, 1.5 equiv) and MeOH (5 mL). 1-cyclopentyl-2-(phenylsulfonyl)ethan-1-ol was isolated by flash chromatography (pet ether/ EtOAc: 85:15) as a colourless oil (79 mg, 0.31 mmol, 78%).

This compound was prepared in enantiomerically-enriched form following procedure D, using 1-cyclopentyl-2-(phenylsulfonyl)ethan-1-one (56 mg, 0.22 mmol, 1.0 equiv), FA/TEA (0.3 mL), [(*R,R*)Teth-TsDpenRuCl] (1.5 mg, 2.4 x 10^{-3} mmol, 1 mol%) and DCM (2 mL). (*S*)- 1-cyclopentyl-2-(phenylsulfonyl)ethan-1-ol was isolated by flash chromatography (pet ether/ EtOAc: 85:15) as a colourless oil (50 mg, 0.20 mmol, 88%).

$[\alpha]_D^{25}$ +10.5° (c 0.1 in $CHCl_3$) 97.2 % ee (*S*).

(found (ESI) [M+Na]+, 277.0869. $C_{13}H_{18}NaO_3S$ requires 277.0869)

ν_{max}: 3519 (broad), 2950, 2867, 1446, 1393, 1295, 1135, 1083, 744, 687, 542 cm^{-1}.

^1H NMR (400 MHz, $CDCl_3$) δ 7.94 (d, J = 7.5 Hz, 2H), 7.69 (t, J = 7.2 Hz, 1H), 7.60 (t, J = 7.3 Hz, 2H), 3.97 (d, J = 3.7 Hz, 1H), 3.29 (d, J = 11.4 Hz, 1H), 3.26 – 3.18 (m, 2H), 2.00 – 1.85 (m, 1H), 1.79 – 1.38 (m, 7H), 1.14 (s, 1H).

^{13}C NMR (101 MHz, $CDCl_3$) δ 134.02, 129.46, 127.92, 69.70, 61.63, 45.26, 28.69, 28.19, 25.57.

Enantiomeric excess determined by HPLC analysis (CHIRALCEL OD-H column, hexane 90:10 iPrOH, 1.0 mL/min, T = 25°C, λ = 210 nm, Ketone 18.5 min, *R* enantiomer 13.7 min, *S*-enantiomer 16.2 min). 97.2% ee (*S*).

m/z (ESI) 277.2 ([M + Na]+, 100 %).

1-Cyclobutyl-2-(phenylsulfonyl)ethan-1-one 10c

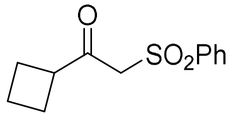

This compound is novel.

This compound was prepared following procedure A using: 2-bromo-1-cyclobutylethan-1-one (800 mg, 4.54 mmol, 1.0 equiv), benzene sulphinic acid (900 mg, 5.49 mmol, 1.2 equiv) and dry DMF (15 mL). 1-cyclobutyl-2-(phenylsulfonyl)ethan-1-one was isolated by flash chromatography (pet ether/ EtOAc: 86:14) as a white solid (887 mg, 3.69 mmol, 81.5%).

(found (ESI) [M+Na]+, 261.0559. $C_{12}H_{14}NaO_3S$ requires 261.0556)

mp 65-66 °C

ν_{max}: 3371 (broad), 2974, 2932, 1446, 1284, 1168, 1138, 1078, 747, 685, 522 cm^{-1}.

^{1}H NMR (400 MHz, $CDCl_3$) δ 7.89 (d, *J* = 7.6 Hz, 2H), 7.68 (t, *J* = 7.3 Hz, 1H), 7.58 (t, *J* = 7.3 Hz, 2H), 4.12 (s, 2H), 3.58 (p, *J* = 8.5 Hz, 1H), 2.22 (dt, *J* = 14.5, 7.4 Hz, 4H), 2.05 – 1.92 (m, 1H), 1.82 (s, 1H).

^{13}C NMR (101 MHz, $CDCl_3$) δ 199.04, 138.94, 134.23, 129.31, 128.34, 64.59, 46.37, 24.14, 17.35.

m/z (ESI) 261.1 ([M + Na]+, 100 %).

Racemic and (*S*)-1-cyclobutyl-2-(phenylsulfonyl)ethan-1-ol 11c

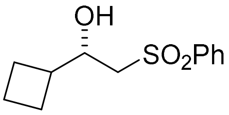

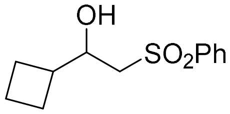

This compound is novel.

This compound was prepared in racemic form following procedure C using: 1-cyclopentyl-2-(phenylsulfonyl)ethan-1-one (119 mg, 0.50 mmol, 1 equiv), $NaBH_4$ (29 mg, 0.77 mmol, 1.5 equiv) and MeOH (5 mL). 1-cyclobutyl-2-(phenylsulfonyl)ethan-1-ol was isolated by flash chromatography (pet ether/ EtOAc: 85:15) as a white solid (101 mg, 0.421 mmol, 84.1%).

This compound was prepared in enantiomerically-enriched form following procedure D, using 1-cyclobutyl-2-(phenylsulfonyl)ethan-1-one (119 mg, 0.50 mmol, 1.0 equiv), FA/TEA (0.4 mL), [(*R,R*)Teth-TsDpenRuCl] (3.1 mg, 5.0 x 10^{-3} mmol, 1 mol%) and DCM (3 mL). (*S*)- 1-cyclobutyl-2-(phenylsulfonyl)ethan-1-ol was isolated by flash chromatography (pet ether/ EtOAc: 85:15) as a white solid (107 mg, 0.446 mmol, 89.1%).

mp 73-74 °C

$[\alpha]_D^{25}$ +13.2° (c 0.1 in $CHCl_3$) 98.4 % ee (*S*)

(found (ESI) [M+Na]+, 263.0712. $C_{12}H_{16}NaO_3S$ requires 263.0712)

ν_{max}: 2984, 2931, 1707, 1446 1307, 1109, 1027, 720, 686, 564, 527, 512 cm^{-1}.

^{1}H NMR (400 MHz, $CDCl_3$) δ 7.94 (d, *J* = 7.5 Hz, 2H), 7.69 (t, *J* = 7.2 Hz, 1H), 7.60 (t, *J* = 7.3 Hz, 2H), 4.07 (d, *J* = 4.2 Hz, 1H), 3.23 (s, 1H), 3.11 (t, *J* = 10.0 Hz, 2H), 2.35 (dd, *J* = 15.2, 7.6 Hz, 1H), 2.04 – 1.66 (m, 6H).

^{13}C NMR (101 MHz, $CDCl_3$) δ 139.35, 134.03, 129.46, 127.92, 69.49, 59.99, 40.07, 23.85, 23.50, 17.62.

Enantiomeric excess determined by HPLC analysis (CHIRALCEL OD-H column, hexane 90:10 iPrOH, 1.0 mL/min, T = 25°C, λ = 210 nm, Ketone 20.9 min, *R* enantiomer 16.2 min, *S*-enantiomer 17.9 min). 98.4% ee (*S*).

m/z (ESI) 263.2 ([M + Na]+, 100 %).

1-Cyclopropyl-2-(phenylsulfonyl)ethan-1-one 10d

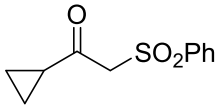

This compound is known and has been fully characterized

Thomsen, M. W.; Handwerker, B. M.; Katz, S. A.; Belser, R. B. *J. Org. Chem.* **1988**, *53*, 906-907.

This compound was prepared following procedure A using: 2-bromo-1-cyclopropylethan-1-one (1.0 g, 6.2 mmol, 1 equiv), benzene sulphinic acid (1.1 g, 6.8 mmol, 1.1 equiv) and dry DMF (20 mL). 1-cyclopropyl-2-(phenylsulfonyl)ethan-1-one was isolated by flash chromatography (pet ether/ EtOAc: 80:20) as a white solid (1.2 g, 5.3 mmol, 88%).

mp 57-58 ºC

^{1}H NMR (400 MHz, $CDCl_3$) δ 7.91 (t, *J* = 6.9 Hz, 2H), 7.69 (dd, *J* = 14.6, 7.2 Hz, 1H), 7.63 – 7.50 (m, 2H), 4.25 (d, *J* = 41.2 Hz, 2H), 2.30 – 2.06 (m, 1H), 1.19 – 0.98 (m, 4H).

13C NMR (101 MHz, $CDCl_3$) δ 198.39, 138.76, 134.28, 129.30, 128.33, 68.08, 22.10, 13.37.

m/z (ESI) 247.1 ([M + Na]+, 100 %).

Racemic and (*S*)- 1-cyclopropyl-2-(phenylsulfonyl)ethan-1-ol 11d

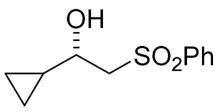

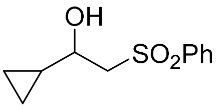

This compound is known and has been fully characterized

Moure, A. L.; Arrayás, R. G.; Carretero, J. C. *Chem Commun.*, **2011**, *47*, 6701 – 6703.

This compound was prepared in racemic form following procedure C using: 1-cyclopropyl-2-(phenylsulfonyl)ethan-1-one (100 mg, 0.446 mmol, 1 equiv), $NaBH_4$ (26 mg, 0.69 mmol, 1.5 equiv) and MeOH (5 mL). 1-cyclopropyl-2-(phenylsulfonyl)ethan-1-ol was isolated by flash chromatography (pet ether/ EtOAc: 75:25) as a colourless oil (86 mg, 0.38 mmol, 85%).

This compound was prepared in enantiomerically-enriched form following procedure D, using 1-cyclopropyl-2-(phenylsulfonyl)ethan-1-one (116 mg, 0.517 mmol, 1.0 equiv), FA/TEA (0.4 mL), [(*R,R*)Teth-TsDpenRuCl] (3.1 mg, 5.0 x 10^{-3} mmol, 1 mol%) and DCM (3 mL). (*S*)- 1-cyclopropyl-2-(phenylsulfonyl)ethan-1-ol was isolated by flash chromatography (pet ether/ EtOAc: 75:25) as a colourless oil (104 mg, 0.460 mmol, 88.8%).

$[\alpha]_D^{25}$ +16.2º (c 0.1 in $CHCl_3$) 99.2 % ee (*S*).

^{1}H NMR (400 MHz, $CDCl_3$) δ 7.77 (d, J = 7.7 Hz, 2H), 7.51 (t, J = 7.3 Hz, 1H), 7.42 (t, J = 7.3 Hz, 2H), 3.34 (t, J = 7.8 Hz, 1H), 3.29 – 3.16 (m, 2H), 3.11 – 2.94 (m, 1H), 0.85 – 0.70 (m, 1H), 0.48 – 0.21 (m, 3H), 0.01 (d, J = 5.4 Hz, 1H).

^{13}C NMR (101 MHz, $CDCl_3$) δ 137.42, 132.04, 127.45, 125.96, 68.39, 60.37, 14.94, 1.64, -0.00.

Enantiomeric excess determined by HPLC analysis (CHIRALPAK AD-H column, hexane 90:10 iPrOH, 1.0 mL/min, T = 25°C, λ = 210 nm, Ketone 26.7 min, *R* enantiomer 52.6 min, *S*-enantiomer 39.9 min). 99.2% ee (*S*).

m/z (ESI) 249.1 ([M + Na]+, 100 %).

1-(Phenylsulfonyl)octan-2-one 10e

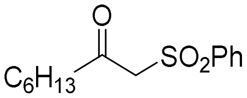

This compound is known and has been fully characterized

Fortes, C. C.; Garrote, C. F.D. *Synth. Commun.* **1997**, *27*, 2993 – 3026.

This compound was prepared following procedure A using: 1-bromooctan-2-one (900 mg, 4.37 mmol, 1.0 equiv), benzene sulphinic acid (900 mg, 5.49 mmol, 1.2 equiv) and dry DMF (15 mL). 1-(phenylsulfonyl)octan-2-one was isolated by flash chromatography (pet ether/ EtOAc: 85:15) as a white solid (500 mg, 1.86 mmol, 41.5%).

mp 45-46 °C

^{1}H NMR (400 MHz, $CDCl_3$) δ 7.82 (d, J = 7.5 Hz, 2H), 7.62 (t, J = 7.3 Hz, 1H), 7.51 (t, J = 7.3 Hz, 2H), 4.08 (s, 2H), 2.63 (t, J = 7.1 Hz, 2H), 1.53 – 1.41 (m, 2H), 1.19 (s, 6H), 0.81 (t, J = 5.8 Hz, 3H).

^{13}C NMR (101 MHz, $CDCl_3$) δ 200.38, 136.67, 134.30, 129.23, 129.12, 76.02, 31.49, 31.29, 26.76, 26.56, 22.18, 13.83.

m/z (ESI) 291.2 ([M + Na]+, 100 %).

Racemic and (*S*)- 1-(phenylsulfonyl)octan-2-ol 11e

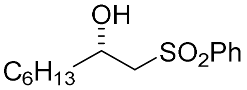

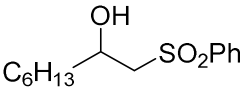

This compound is known and has been fully characterized

Still, I. W. J.; Ablenas, F. J. Synth. Commun., **1982**, *12*, 1103 – 1110.

This compound was prepared in racemic form following procedure C using: 1-(phenylsulfonyl)octan-2-one (150 mg, 0.559 mmol, 1 equiv), $NaBH_4$ (35 mg, 0.92 mmol, 1.5 equiv) and MeOH (5 mL). 1-(phenylsulfonyl)octan-2-ol was isolated by flash chromatography (pet ether/ EtOAc: 85:15) as a colourless oil (132 mg, 0.489 mmol, 87.3%).

This compound was prepared in enantiomerically-enriched form following procedure D, using 1-(phenylsulfonyl)octan-2-one (96 mg, 0.35 mmol, 1.0 equiv), FA/TEA (0.3 mL), [(*R,R*)Teth-TsDpenRuCl] (2.2 mg, 3.5 x 10^{-3} mmol, 1 mol%) and DCM (2 mL). (*S*)- 1-(phenylsulfonyl)octan-2-ol was isolated by flash chromatography (pet ether/ EtOAc: 85:15) as a colourless oil (81 mg, 0.30 mmol, 84%).

$[\alpha]_D^{25}$ +20.6° (c 0.1 in $CHCl_3$) 91.0 % ee (*S*).

^{1}H NMR (400 MHz, $CDCl_3$) δ 7.87 (d, *J* = 7.6 Hz, 2H), 7.62 (t, *J* = 7.2 Hz, 1H), 7.53 (t, *J* = 7.3 Hz, 2H), 4.09 (s, 1H), 3.27 (s, 1H), 3.20 – 3.06 (m, 2H), 1.49 – 1.11 (m, 10H), 0.78 (t, *J* = 5.9 Hz, 3H).

^{13}C NMR (101 MHz, $CDCl_3$) δ 139.32, 134.05, 129.48, 127.92, 65.96, 62.28, 36.46, 31.64, 28.94, 24.92, 22.51, 14.02.

Enantiomeric excess determined by HPLC analysis (CHIRALCEL OD-H column, hexane 90:10 iPrOH, 1.0 mL/min, T = 25°C, λ = 210 nm, Ketone 24.0 min, *R* enantiomer 16.1 min, *S*-enantiomer 18.5 min). 91.0% ee (*S*).

m/z (ESI) 293.2 ([M + Na]+, 100 %).

1-(Phenylsulfonyl)pentan-2-one 10f

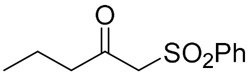

This compound is known but not fully characterized.

Chen, Q.; Wang, K.; Yuan, C. *New J. Chem.*, **2009**, *33,* 972 – 975.

This compound was prepared following procedure A using: 1-bromopentan-2-one (1.0 g, 6.1 mmol, 1 equiv), benzene sulphinic acid (1.1 g, 6.7 mmol, 1.1 equiv) and dry DMF (20 mL). 1-(phenylsulfonyl)pentan-2-one was isolated by flash chromatography (pet ether/ EtOAc: 85:15) as a white solid (941 mg, 4.44 mmol, 68.3%).

mp 54-55 °C

(found (ESI) [M+Na]+, 249.0557. $C_{11}H_{14}NaO_3S$ requires 249.0556)

ν_{max}: 2956, 2902, 1720, 1447, 1296, 1142, 1031, 754, 689, 553, 528 cm^{-1}.

^{1}H NMR (400 MHz, $CDCl_3$) δ 7.82 (d, *J* = 7.6 Hz, 2H), 7.62 (t, *J* = 7.3 Hz, 1H), 7.51 (t, *J* = 7.3 Hz, 2H), 4.07 (s, 2H), 2.61 (t, *J* = 7.0 Hz, 2H), 1.52 (h, *J* = 6.9 Hz, 2H), 0.84 (t, *J* = 7.3 Hz, 3H).

^{13}C NMR (101 MHz, $CDCl_3$) δ 198.14, 138.74, 134.30, 129.35, 128.30, 66.85, 46.28, 16.62, 13.38.

m/z (ESI) 249.1 ([M + Na]+, 100 %).

Racemic and (*S*)- 1-(phenylsulfonyl)pentan-2-ol 11f

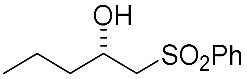

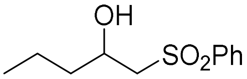

This compound is known and has been fully characterized.

Chen, Q.; Wang, K.; Yuan, C. *New J. Chem.*, **2009**, *33,* 972 – 975.

This compound was prepared in racemic form following procedure C using: 1-(phenylsulfonyl)pentan-2-one (100 mg, 0.472 mmol, 1.0 equiv), $NaBH_4$ (25 mg, 0.66 mmol, 1.5 equiv) and MeOH (5 mL). 1-(phenylsulfonyl)pentan-2-ol was isolated by flash chromatography (pet ether/ EtOAc: 85:15) as a colourless oil (96 mg, 0.45 mmol, 95%).

This compound was prepared in enantiomerically-enriched form following procedure D, using 1-(phenylsulfonyl)pentan-2-one (113 mg, 0.533 mmol, 1.0 equiv), FA/TEA (0.4 mL), [(*R,R*)Teth-TsDpenRuCl] (3.1 mg, 5.0 x 10^{-3} mmol, 1 mol%) and DCM (3 mL). (*S*)- 1-(phenylsulfonyl)pentan-2-ol was isolated by flash chromatography (pet ether/ EtOAc: 85:15) as a colourless oil (109 mg, 0.509 mmol, 95.6%).

$[\alpha]_D^{25}$ +26.0° (c 0.1 in $CHCl_3$) 90.4 % ee (*S*). (lit(above) $[\alpha]^D$ +11.0° (c 1.0 in $CHCl_3$, 70% ee (*S*)

^{1}H NMR (400 MHz, $CDCl_3$) δ 7.87 (d, *J* = 7.5 Hz, 2H), 7.62 (t, *J* = 7.2 Hz, 1H), 7.53 (t, *J* = 7.3 Hz, 2H), 4.10 (s, 1H), 3.26 (s, 1H), 3.14 (p, *J* = 14.3 Hz, 2H), 1.55 – 1.18 (m, 4H), 0.82 (d, *J* = 6.5 Hz, 3H).

^{13}C NMR (101 MHz, $CDCl_3$) δ 139.31, 134.06, 129.48, 127.91, 65.69, 62.30, 38.50, 18.23, 13.73.

Enantiomeric excess determined by HPLC analysis (CHIRALPAK IA column, hexane 90:10 iPrOH, 1.0 mL/min, T = 25°C, λ = 210 nm, Ketone 9.5 min, *R* enantiomer 19.0 min, *S*-enantiomer 16.9 min). 90.4% ee (*S*).

m/z (ESI) 251.1 ([M + Na]+, 100 %).

3,3-Dimethyl-1-(phenylsulfonyl)butan-2-one 11g

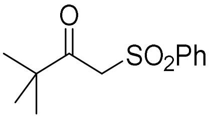

This compound is known and has been fully characterized

Xiang, J.; Ipek, M.; Suri, V.; Tam, M.; Xing, Y.; Huang, N.; Zhang, Y.; Tobin, J.; Mansour, T. S.; McKew, J. *Bioorg. Med. Chem.,* **2007**, *15*, 4396 – 4405.

This compound was prepared following procedure A using: 1-bromo-3,3-dimethylbutan-2-one (800 mg, 4.49 mmol, 1.0 equiv), benzene sulphinic acid (900 mg, 5.49 mmol, 1.2 equiv) and dry DMF (15 mL). 3,3-dimethyl-1-(phenylsulfonyl)butan-2-one was isolated by flash chromatography (pet ether/ EtOAc: 85:15) as a white solid (900 mg, 3.75 mmol, 84.1%).

mp 80-81 °C

^{1}H NMR (400 MHz, $CDCl_3$) δ 7.96 (d, *J* = 7.4 Hz, 2H), 7.67 (t, *J* = 7.3 Hz, 1H), 7.58 (t, *J* = 7.3 Hz, 2H), 4.33 (s, 2H), 1.12 (s, 9H).

^{13}C NMR (101 MHz, $CDCl_3$) δ 203.25, 139.50, 134.01, 129.11, 128.71, 60.79, 45.32, 25.58.

m/z (ESI) 263.2 ([M + Na]+, 100 %).

Racemic and (*S*)- 3,3-dimethyl-1-(phenylsulfonyl)butan-2-ol 11g

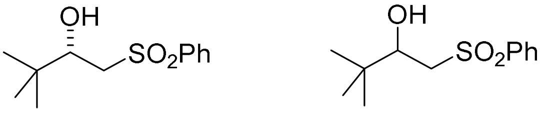

This compound is known and has been fully characterized

Zhao, G.; Hu, J.-b.; Qian, Z.-s.; Yin, W.-x. *Tetrahedron Asymmetry*, **2002**, *13*, 2095 – 2098.

This compound was prepared in racemic form following procedure C using: 3,3-dimethyl-1-(phenylsulfonyl)butan-2-one (120 mg, 0.50 mmol, 1 equiv), $NaBH_4$ (29 mg, 0.77 mmol, 1.5 equiv) and MeOH (5 mL). 3,3-dimethyl-1-(phenylsulfonyl)butan-2-ol was isolated by flash chromatography (pet ether/ EtOAc: 83:17) as a colourless oil (107 mg, 0.442 mmol, 88.4%).

This compound was prepared in enantiomerically-enriched form following procedure D, using 3,3-dimethyl-1-(phenylsulfonyl)butan-2-one (120 mg, 0.50 mmol, 1.0 equiv), FA/TEA (0.4 mL), [(*R,R*)Teth-TsDpenRuCl] (3.1 mg, 5.0 x 10^{-3} mmol, 1 mol%) and DCM (3 mL). (*S*)- 3,3-dimethyl-1-(phenylsulfonyl)butan-2-ol was isolated by flash chromatography (pet ether/ EtOAc: 83:17) as a colourless oil (109 mg, 0.450 mmol, 90.0%).

$[\alpha]_D^{25}$ +21.3° (c 0.1 in $CHCl_3$) 86.4 % ee (*S*).

^{1}H NMR (400 MHz, $CDCl_3$) δ 7.95 (d, *J* = 7.2 Hz, 2H), 7.69 (t, *J* = 7.3 Hz, 1H), 7.60 (t, *J* = 7.3 Hz, 2H), 3.78 (d, *J* = 9.9 Hz, 1H), 3.34 – 3.20 (m, 2H), 3.19 – 3.07 (m, 1H), 0.87 (s, 9H).

^{13}C NMR (101 MHz, $CDCl_3$) δ 139.24, 134.00, 129.45, 127.94, 73.22, 58.82, 34.92, 25.37.
Enantiomeric excess determined by HPLC analysis (CHIRALCEL OD-H column, hexane 93:07 iPrOH, 0.7 mL/min, T = 25°C, λ = 210 nm, Ketone 16.5 min, *R* enantiomer 17.5 min, *S*-enantiomer 20.4 min). 86.4% ee (*S*).
m/z (ESI) 265.2 ([M + Na]+, 100 %).

1-Cyclobutyl-2-phenoxyethan-1-one

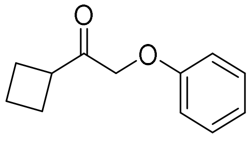

This compound is novel.
This compound was prepared following procedure B using: 2-bromo-1-cyclobutylethan-1-one (700 mg, 3.98 mmol, 1 equiv), Phenol (400 mg, 4.25 mmol, 1.1 equiv), K_2CO_3 (800 mg, 5.80 mmol, 1.5 equiv) and dry DMF (20 mL). 1-cyclobutyl-2-phenoxyethan-1-one was isolated by flash chromatography (pet ether/ EtOAc: 98:02) as a white solid (513 mg, 2.70 mmol, 67.5%).
mp 39-40 °C
(found (ESI) [M+Na]+, 213.0889. $C_{12}H_{14}NaO_2$ requires 213.0886)
ν_{max}: 2941, 2863, 1714, 1587, 1487, 1223, 1018, 753, 691 cm^{-1}.
^1H NMR (400 MHz, $CDCl_3$) δ 7.26 – 7.15 (m, 2H), 6.91 (t, *J* = 7.4 Hz, 1H), 6.80 (dd, *J* = 8.7, 0.9 Hz, 2H), 4.49 (s, 2H), 3.57 – 3.41 (m, 1H), 2.24 (dtd, *J* = 17.5, 8.7, 2.1 Hz, 2H), 2.10 (qdd, *J* = 8.6, 4.1, 2.6 Hz, 2H), 2.03 – 1.87 (m, 1H), 1.86 – 1.70 (m, 1H).
^{13}C NMR (101 MHz, $CDCl_3$) δ 208.43, 157.94, 129.65, 121.60, 114.50, 71.40, 42.26, 24.29, 18.17.
m/z (ESI) 213.2 ([M + Na]+, 100 %).

Racemic and (*S*)- 1-cyclobutyl-2-phenoxyethan-1-ol

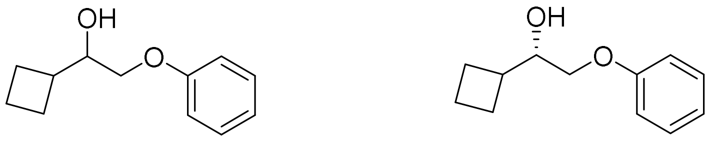

This compound is novel.
This compound was prepared in racemic form following procedure C using: 1-cyclobutyl-2-phenoxyethan-1-one (95 mg, 0.50 mmol, 1 equiv), $NaBH_4$ (29 mg, 0.76 mmol, 1.5 equiv) and MeOH (5 mL). 1-cyclobutyl-2-phenoxyethan-1-ol was isolated by flash chromatography (pet ether/ EtOAc: 96:04) as a white solid (88 mg, 0.46 mmol, 92%).

This compound was prepared in enantiomerically-enriched form following procedure D, using 1-cyclobutyl-2-phenoxyethan-1-one (95 mg, 0.50 mmol, 1.0 equiv), FA/TEA (0.4 mL), [(*R,R*)Teth-TsDpenRuCl] (3.1 mg, 5.0 x 10^{-3} mmol, 1 mol%) and DCM (3 mL). (*S*)- 1-cyclobutyl-2-phenoxyethan-1-ol was isolated by flash chromatography (pet ether/ EtOAc: 96:04) as a white solid (93 mg, 0.48 mmol, 97%).

mp 45-46 °C

$[\alpha]_D^{25}$ +8.2° (c 0.1 in $CHCl_3$) 25.6 % ee (*S*).

(found (ESI) [M+Na]+, 215.1042. $C_{12}H_{16}NaO_2$ requires 215.1043)

ν_{max}: 3209 (broad), 2931, 1595, 1494, 1459, 1244, 1039, 749, 688 cm^{-1}.

^1H NMR (400 MHz, $CDCl_3$) δ 7.27 (d, *J* = 7.9 Hz, 2H), 6.96 (t, *J* = 7.3 Hz, 1H), 6.90 (d, *J* = 7.7 Hz, 2H), 3.93 (d, *J* = 8.6 Hz, 2H), 3.75 (t, *J* = 8.7 Hz, 1H), 2.52 (dd, *J* = 14.9, 7.4 Hz, 1H), 2.28 (s, 1H), 2.16 – 1.78 (m, 6H).

^{13}C NMR (101 MHz, $CDCl_3$) δ 158.68, 129.51, 121.08, 114.58, 73.91, 70.13, 37.58, 24.42, 24.20, 18.46.

Enantiomeric excess determined by HPLC analysis (CHIRALCEL OD-H column, hexane 90:10 iPrOH, 1.0 mL/min, T = 25°C, λ = 210 nm, Ketone 9.9 min, *R* enantiomer 13.0 min, *S*-enantiomer 8.0 min). 25.6% ee (*R*).

m/z (ESI) 215.2 ([M + Na]+, 100 %).

1-Cyclopropyl-2-phenoxyethan-1-one

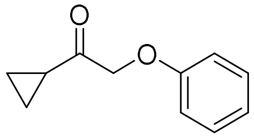

This compound is known and has been fully characterized.

Janin, Y.-L.; Guillou, S.; Lucas-Hourani, M.; Munier-Lehmann, H.; Noel, A.; Salanouve, E.; Tangy, F.; Vidalain, P.-O. Wo2015/155680, **2015,** A2

This compound was prepared following procedure B using: 2-bromo-1-cyclopropylethan-1-one (1.0 g, 6.2 mmol, 1 equiv), Phenol (0.6 g, 6.8 mmol, 1.1 equiv), K_2CO_3 (1.3 g, 9.2 mmol, 1.5 equiv) and dry DMF (20 mL). 1-cyclobutyl-2-phenoxyethan-1-one was isolated by flash chromatography (pet ether/ EtOAc: 98:02) as a colourless oil (806 mg, 4.58 mmol, 74.2%).

^1H NMR (400 MHz, $CDCl_3$) δ 7.36 – 7.26 (m, 2H), 6.99 (t, *J* = 7.3 Hz, 1H), 6.91 (d, *J* = 7.6 Hz, 2H), 4.68 (s, 2H), 2.31 (dd, *J* = 7.6, 3.8 Hz, 1H), 1.13 (t, *J* = 10.5 Hz, 2H), 1.03 – 0.90 (m, 2H).

^{13}C NMR (101 MHz, $CDCl_3$) δ 207.59, 157.98, 129.65, 121.64, 114.64, 73.09, 17.09, 11.93.

m/z (ESI) 199.2 ([M + Na]+, 100 %).

Racemic and (*S*)- 1-cyclopropyl-2-phenoxyethan-1-ol

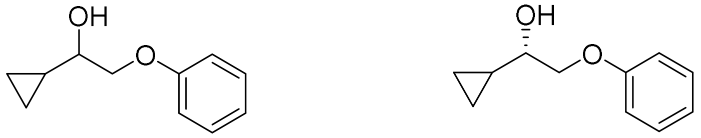

This compound is novel.

This compound was prepared in racemic form following procedure C using: 1-cyclopropyl-2-phenoxyethan-1-one (100 mg, 0.568 mmol, 1 equiv), $NaBH_4$ (32 mg, 0.84 mmol, 1.5 equiv) and MeOH (5 mL). 1-cyclopropyl-2-phenoxyethan-1-ol was isolated by flash chromatography (pet ether/ EtOAc: 96:04) as a colourless oil (91 mg, 0.51 mmol, 90%).

This compound was prepared in enantiomerically-enriched form following procedure D, using 1-cyclopropyl-2-phenoxyethan-1-one (106 mg, 0.602 mmol, 1.0 equiv), FA/TEA (0.4 mL), [(*R,R*)Teth-TsDpenRuCl] (3.7 mg, 5.9 x 10^{-3} mmol, 1 mol%) and DCM (3 mL). (*S*)- 1-cyclopropyl-2-phenoxyethan-1-ol was isolated by flash chromatography (pet ether/ EtOAc: 96:04) as a colourless oil (95 mg, 0.53 mmol, 89%).

$[\alpha]_D^{25}$ +15.0° (c 0.1 in $CHCl_3$) 35.6 % ee (*S*).

(found (ESI) [M+Na]+, 201.0885. $C_{11}H_{14}NaO_2$ requires 201.0886)

ν_{max}: 3392 (broad), 3077, 3006, 2924, 1598, 1494, 1241, 1018, 750, 689 cm^{-1}.

^1H NMR (400 MHz, $CDCl_3$) δ 7.29 (t, *J* = 7.3 Hz, 2H), 6.95 (dd, *J* = 16.5, 7.8 Hz, 3H), 4.11 (d, *J* = 9.4 Hz, 1H), 3.98 (t, *J* = 8.5 Hz, 1H), 3.31 (s, 1H), 2.37 (s, 1H), 1.07 – 0.93 (m, 1H), 0.66 – 0.50 (m, 2H), 0.44 (s, 1H), 0.32 (d, *J* = 3.7 Hz, 1H).

^{13}C NMR (101 MHz, $CDCl_3$) δ 156.64, 127.54, 119.12, 112.61, 72.79, 69.93, 11.57, 0.72, -0.00.

Enantiomeric excess determined by HPLC analysis (CHIRALCEL OD-H column, hexane 90:10 iPrOH, 1.0 mL/min, T = 25°C, λ = 210 nm, Ketone 21.9 min, *R* enantiomer 16.5 min, *S*-enantiomer 10.2 min). 35.6% ee (*R*).

m/z (ESI) 201.2 ([M + Na]+, 100 %).

1-Phenoxypentan-2-one

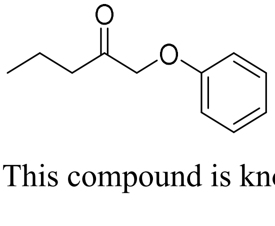

This compound is known and has been fully characterized.

Ogawa; Terada; Muranaka; Hamakawa; Hashimoto; FuJii [Chemical and Pharmaceutical Bulletin, 1986, vol. 34, # 3, p. 1118 - 1127]

This compound was prepared following procedure B using: 1-bromopentan-2-one (1.0 g, 6.1 mmol, 1 equiv), Phenol (0.6 g, 6.7 mmol, 1.1 equiv), K_2CO_3 (1.2 g, 9.0 mmol, 1.5 equiv) and dry DMF (20 mL). 1-phenoxypentan-2-one was isolated by flash chromatography (pet ether/ EtOAc: 98:02) as a colourless liquid (806 mg, 4.53 mmol, 74.2%).

^{1}H NMR (400 MHz, $CDCl_3$) δ 7.29 – 7.18 (m, 2H), 6.92 (t, *J* = 7.3 Hz, 1H), 6.81 (d, *J* = 7.7 Hz, 2H), 4.46 (s, 2H), 2.50 (t, *J* = 7.1 Hz, 2H), 1.68 – 1.48 (m, 2H), 0.87 (t, *J* = 7.3 Hz, 3H).

^{13}C NMR (101 MHz, $CDCl_3$) δ 207.89, 157.85, 129.69, 121.68, 114.54, 72.83, 40.93, 16.64, 13.73.

m/z (ESI) 201.2 ([M + Na]+, 100 %).

Racemic and (*S*)- 1-phenoxypentan-2-ol

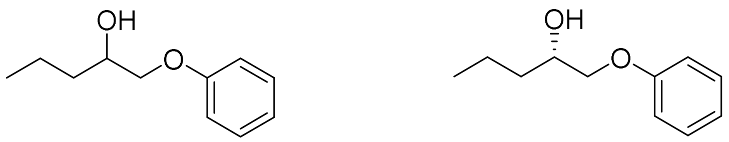

This compound is known and has been fully characterized.

Jacobsen, E. E.; Andresen, L. S.; Anthonsen, T. *Tetrahedron Asymmetry*, **2005**, *16*, 847 – 850.

This compound was prepared in racemic form following procedure C using: 1-phenoxypentan-2-one (98 mg, 0.55 mmol, 1 equiv), $NaBH_4$ (29 mg, 0.77 mmol, 1.5 equiv) and MeOH (5 mL). 1-phenoxypentan-2-ol was isolated by flash chromatography (pet ether/ EtOAc: 96:04) as a colourless oil (90 mg, 0.50 mmol, 91%).

This compound was prepared in enantiomerically-enriched form following procedure D, using 1-phenoxypentan-2-one (98 mg, 0.55 mmol, 1.0 equiv), FA/TEA (0.4 mL), [(*R,R*)Teth-TsDpenRuCl] (3.1 mg, 5.0 x 10^{-3} mmol, 1 mol%) and DCM (3 mL). (*S*)- 1-phenoxypentan-2-ol was isolated by flash chromatography (pet ether/ EtOAc: 96:04) as a colourless oil (91 mg, 0.50 mmol, 92%).

$[\alpha]_D^{25}$ +7.5° (c 0.1 in $CHCl_3$) 38.2 % ee (*S*) (lit (above) $[\alpha]_D^{30}$ -12.25° (c 1.14 in $CHCl_3$, 99.3% ee (*R*).

^{1}H NMR (400 MHz, $CDCl_3$) δ 7.22 (t, *J* = 7.3 Hz, 2H), 6.95 – 6.77 (m, 3H), 3.93 (dd, *J* = 15.6, 6.1 Hz, 2H), 3.76 (t, *J* = 8.2 Hz, 1H), 2.24 (s, 1H), 1.59 – 1.32 (m, 4H), 0.90 (t, *J* = 5.9 Hz, 3H).

^{13}C NMR (101 MHz, $CDCl_3$) δ 158.64, 129.54, 121.11, 114.59, 72.20, 69.92, 35.21, 18.75, 14.09.

Enantiomeric excess determined by HPLC analysis (CHIRALCEL OD-H column, hexane 90:10 iPrOH, 1.0 mL/min, T = 25°C, λ = 210 nm, Ketone 5.5 min, *R* enantiomer 7.5 min, *S*-enantiomer 12.9 min). 38.2% ee (*R*).
m/z (ESI) 203.2 ([M + Na]+, 100 %).

3,3-Dimethyl-1-phenoxybutan-2-one

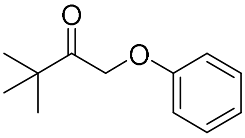

This compound is known and has been fully characterized.
Enthaler, S.; Spilker, B.; Erre, G.; Junge, K.; Tse, M. K.; Beller, M. *Tetrahedron* **2008**, *64*, 3867 – 3876.
This compound was prepared following procedure B using: 1-bromo-3,3-dimethylbutan-2-one (800 mg, 4.49 mmol, 1 equiv), Phenol (500 mg, 5.32 mmol, 1.2 equiv), K_2CO_3 (900 mg, 6.52 mmol, 1.5 equiv) and dry DMF (15 mL). 3,3-dimethyl-1-phenoxybutan-2-one was isolated by flash chromatography (pet ether/ EtOAc: 98:02) as a white solid (587 mg, 3.06 mmol, 68.0%).
mp 37-38 °C
^{1}H NMR (400 MHz, $CDCl_3$) δ 7.28 (t, *J* = 7.5 Hz, 2H), 6.96 (dd, *J* = 16.5, 9.2 Hz, 1H), 6.88 (d, *J* = 7.6 Hz, 2H), 4.87 (s, 2H), 1.25 (s, 9H).
^{13}C NMR (101 MHz, $CDCl_3$) δ 209.51, 158.10, 129.53, 121.54, 114.74, 68.94, 43.20, 26.38.
m/z (ESI) 215.3 ([M + Na]+, 100 %).

Racemic and (*S*)- 3,3-dimethyl-1-phenoxybutan-2-ol

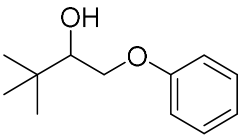

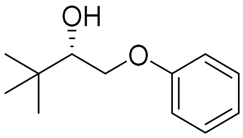

This compound is known but not fully characterized:
Enthaler, S.; Spilker, B.; Erre, G.; Junge, K.; Tse, M. K.; Beller, M. *Tetrahedron*, **2008**, *64*, 3867 – 3876.
This compound was prepared in racemic form following procedure C using: 3,3-dimethyl-1-phenoxybutan-2-one (96 mg, 0.50 mmol, 1 equiv), $NaBH_4$ (29 mg, 0.77 mmol, 1.5 equiv) and MeOH (5 mL). 3,3-dimethyl-1-phenoxybutan-2-ol was isolated by flash chromatography (pet ether/ EtOAc: 96:04) as a colourless oil (88 mg, 0.45 mmol, 91%).

This compound was prepared in enantiomerically-enriched form following procedure D, using 3,3-dimethyl-1-phenoxybutan-2-one (96 mg, 0.50 mmol, 1.0 equiv), FA/TEA (0.4 mL), [(*R,R*)Teth-TsDpenRuCl] (3.1 mg, 5.0 x 10^{-3} mmol, 1 mol%) and DCM (3 mL). (*S*)- 3,3-dimethyl-1-phenoxybutan-2-ol was isolated by flash chromatography (pet ether/ EtOAc: 96:04) as a colourless oil (91 mg, 0.47 mmol, 94%).

$[\alpha]_D^{25}$ +4.7° (c 0.1 in $CHCl_3$) 30.4 % ee.

ν_{max}: 3256 (broad), 2950, 2849, 1598, 1496, 1238, 1041, 751, 689 cm^{-1}.

^1H NMR (400 MHz, $CDCl_3$) δ 7.29 (dd, *J* = 14.3, 7.0 Hz, 2H), 7.03 – 6.85 (m, 3H), 4.12 (d, *J* = 9.2 Hz, 1H), 3.87 (t, *J* = 9.0 Hz, 1H), 3.76 – 3.60 (m, 1H), 2.41 (s, 1H), 1.01 (s, 9H).

^{13}C NMR (101 MHz, $CDCl_3$) δ 158.69, 129.53, 121.14, 114.67, 77.22, 69.38, 33.59, 26.06.

Enantiomeric excess determined by HPLC analysis (CHIRALCEL OD-H column, hexane 90:10 iPrOH, 1.0 mL/min, T = 25°C, λ = 210 nm, Ketone 9.9 min, *R* enantiomer 5.8 min, *S*-enantiomer 8.3 min). 30.4% ee (*R*).

m/z (ESI) 217.3 ([M + Na]+, 100 %).

1-Cyclohexyl-2-(phenylthio)ethan-1-one

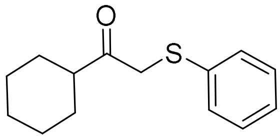

This compound is known and has been fully characterized.

Itter, N. S.-v.; Steckhan, E. *Tetrahedron,* **1987**, *43*, 2475 – 2484.

This compound was prepared following procedure B using: 2-bromo-1-cyclohexylethan-1-one (600 mg, 2.94 mmol, 1 equiv), Thiophenol (300 mg, 2.72 mmol, 1.0 equiv), K_2CO_3 (400 mg, 2.89 mmol, 1.0 equiv) and dry DMF (10 mL). 1-cyclohexyl-2-(phenylthio)ethan-1-one was isolated by flash chromatography (pet ether/ EtOAc: 98:02) as a colourless liquid (368 mg, 1.57 mmol, 52.4%).

^1H NMR (300 MHz, CDCl3) δ 7.37 – 7.23 (m, 5H), 3.74 (s, 2H), 2.67 (t, *J* = 10.6 Hz, 1H), 1.79 (t, *J* = 11.0 Hz, 4H), 1.66 (d, *J* = 9.0 Hz, 1H), 1.27 (dt, *J* = 22.3, 12.8 Hz, 5H).

^{13}C NMR (75 MHz, CDCl3) δ 208.24, 138.93, 135.13, 129.78, 126.65, 48.49, 42.65, 28.72, 28.56, 25.65, 25.49.

m/z (ESI) 257.2 ([M + Na]+, 100 %).

Racemic and (*S*)- 1-cyclohexyl-2-(phenylthio)ethan-1-ol

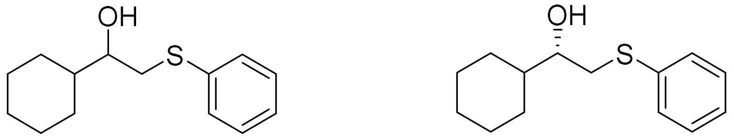

This compound is known but not fully characterized:

Augeri, D. J.; Baumeister, S. A.; Bruncko, M.; Dickman, D. A.; Ding, H.; Dinges, J.; Fesik, S. W.; HaJduk, P. J.; Kunzer, A.R.; William, M; Nettesheim, D. J.; Oost, T.; Petros, A. M.; Rosenberg, S. H.; Shen, W.; Thomas, S. A.; Wang, X.; Wendt, M. D. US2002/55631, 2002, A1

This compound was prepared in racemic form following procedure C using: 1-cyclohexyl-2-(phenylthio)ethan-1-one (117 mg, 0.50 mmol, 1 equiv), $NaBH_4$ (29 mg, 0.77 mmol, 1.5 equiv) and MeOH (5 mL). 1-cyclohexyl-2-(phenylthio)ethan-1-ol was isolated by flash chromatography (pet ether/ EtOAc: 97:03) as a colourless oil (98 mg, 0.41 mmol, 83%).

This compound was prepared in enantiomerically-enriched form following procedure D, using 1-cyclohexyl-2-(phenylthio)ethan-1-one (80 mg, 0.34 mmol, 1.0 equiv), FA/TEA (0.3 mL), [(R,R)Teth-TsDpenRuCl] (2.0 mg, 3.2 x 10-3 mmol, 1 mol%) and DCM (2 mL). (S)- 1-cyclohexyl-2-(phenylthio)ethan-1-one was isolated by flash chromatography (pet ether/ EtOAc: 97:03) as a colourless oil (61 mg, 0.26 mmol, 76%).

$[\alpha]_D^{25}$ +5.1° (c 0.1 in $CHCl_3$) 33.4 % ee.

ν_{max}: 3408 (broad), 2921, 2849, 1598, 1495, 1240, 1048, 748, 690 cm^{-1}.

(found (ESI) [M+Na]+, 259.1128. $C_{14}H_{20}NaOS$ requires 259.1127)

^{1}H NMR (300 MHz, $CDCl_3$) δ 7.38 – 7.17 (m, 5H), 3.51 – 3.33 (m, 1H), 3.23 (d, *J* = 13.6 Hz, 1H), 2.86 (dd, *J* = 13.5, 9.4 Hz, 1H), 2.42 (s, 1H), 1.87 – 1.64 (m, 5H), 1.57 – 1.41 (m, 1H), 1.32 – 1.03 (m, 5H).

^{13}C NMR (75 MHz, $CDCl_3$) δ 135.39, 130.05, 129.74, 128.92, 73.31, 73.13, 42.76, 39.87, 29.00, 28.11, 26.36, 26.09.

Enantiomeric excess determined by HPLC analysis (CHIRALCEL OD-H column, hexane 93:07 iPrOH, 0.7 mL/min, T = 25°C, λ = 210 nm, Ketone 9.8 min, *R* enantiomer 11.4 min, *S*-enantiomer 13.4 min). 33.4% ee (*R*).

m/z (ESI) 259.2 ([M + Na]+, 100 %).

1-cyclopropyl-2-(phenylthio)ethan-1-one

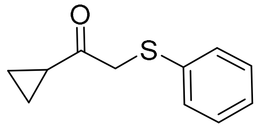

This compound is known and has been fully characterized.

Wang, Q.; Li, T.-R.; Lu, L.-Q.; Li, M.-M.; Zhang, K.; Xiao, W.-J. *J. Am. Chem. Soc.*, **2016**, *138*, 8360 – 8363.

This compound was prepared following procedure B using: 2-bromo-1-cyclopropylethan-1-one (700 mg, 4.32 mmol, 1 equiv), Thiophenol (500 mg, 4.54 mmol, 1.0 equiv), K_2CO_3 (900 mg, 6.52 mmol, 1.5 equiv) and dry DMF (20 mL). 1-cyclopropyl-2-(phenylthio)ethan-1-one was isolated by flash chromatography (pet ether/ EtOAc: 98:02) as a colourless liquid (592 mg, 3.08 mmol, 68.4%).

^{1}H NMR (400 MHz, $CDCl_3$) δ 7.36 (d, J = 7.6 Hz, 2H), 7.28 (t, J = 7.6 Hz, 2H), 7.21 (t, J = 7.0 Hz, 1H), 3.80 (s, 2H), 2.30 – 2.16 (m, 1H), 1.00 (d, J = 15.6 Hz, 2H), 0.96 – 0.84 (m, 2H).

^{13}C NMR (101 MHz, $CDCl_3$) δ 205.40, 134.89, 129.82, 129.07, 126.85, 44.81, 19.21, 11.79.

m/z (ESI) 215.1 ([M + Na]+, 100 %).

Racemic and (*S*)- 1-cyclopropyl-2-(phenylthio)ethan-1-ol

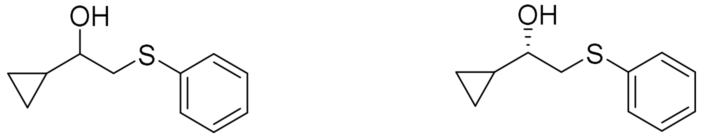

This compound is novel.

This compound was prepared in racemic form following procedure C using: 1-cyclopropyl-2-(phenylthio)ethan-1-one (96 mg, 0.50 mmol, 1 equiv), $NaBH_4$ (29 mg, 0.77 mmol, 1.5 equiv) and MeOH (5 mL). 1-cyclopropyl-2-(phenylthio)ethan-1-ol was isolated by flash chromatography (pet ether/ EtOAc: 97:03) as a colourless oil (87 mg, 0.45 mmol, 90%).

This compound was prepared in enantiomerically-enriched form following procedure D, using 1-cyclopropyl-2-(phenylthio)ethan-1-one (96 mg, 0.50 mmol, 1.0 equiv), FA/TEA (0.4 mL), [(R,R)Teth-TsDpenRuCl] (3.1 mg, 5.0 x 10^{-3} mmol, 1 mol%) and DCM (3 mL). (S)- 1-cyclopropyl-2-(phenylthio)ethan-1-ol was isolated by flash chromatography (pet ether/ EtOAc: 97:03) as a colourless oil (89 mg, 0.46 mmol, 92%).

$[\alpha]_D^{25}$ +33.2° (c 0.1 in $CHCl_3$) 87.2 % ee.

(found (ESI) [M+Na]+, 217.0658. $C_{11}H_{14}NaOS$ requires 217.0658)

ν_{max}: 3385 (broad), 3077, 3002, 2917, 1583, 1479, 1437, 1023, 753, 688 cm^{-1}.

^{1}H NMR (400 MHz, $CDCl_3$) δ 7.16 (d, *J* = 7.5 Hz, 2H), 7.06 (t, *J* = 7.6 Hz, 2H), 6.98 (t, *J* = 7.2 Hz, 1H), 3.08 (q, *J* = 7.5 Hz, 1H), 2.87 – 2.71 (m, 2H), 2.16 (s, 1H), 0.74 (d, *J* = 5.2 Hz, 1H), 0.41 – 0.23 (m, 2H), 0.14 (d, *J* = 9.2 Hz, 1H), -0.01 (d, *J* = 11.3 Hz, 1H).

^{13}C NMR (101 MHz, $CDCl_3$) δ 133.17, 127.38, 126.67, 124.08, 71.61, 39.31, 14.09, 0.64, -0.00.

Enantiomeric excess determined by HPLC analysis (CHIRALCEL OD-H column, hexane 93:07 iPrOH, 0.7 mL/min, T = 25°C, λ = 210 nm, Ketone 7.8 min, *R* enantiomer 25.7 min, *S*-enantiomer 35.4 min). 87.2% ee (*R*).

m/z (ESI) 217.1 ([M + Na]+, 100 %).

Racemic and (*S*)-4-Phenyl-1-(phenylsulfonyl)but-3-yn-2-ol 11h

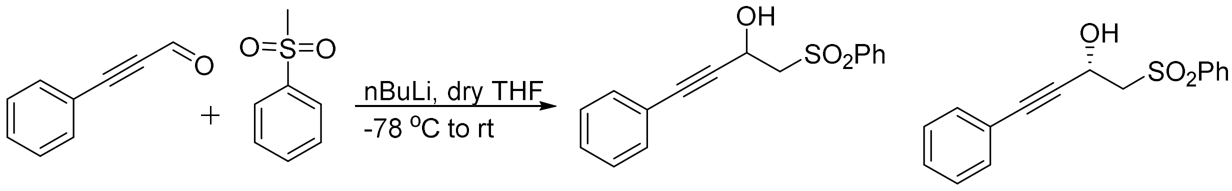

This compound is novel.

To a solution of methyl phenyl sulphone (1.65 g, 10.6 mmol, 1.1 equiv) in dry THF (35 mL) was added *n*BuLi (2.5 M in *n*-hexane, 5.20 mL, 10.6 mmol, 1.1 equiv) dropwise at –78 ºC under nitrogen atmosphere. After the reaction mixture had been stirred at –78 ºC for 30 minutes, 3-phenylpropiolaldehyde (1.25 g, 9.60 mmol, 1.0 equiv) was added dropwise at -78 ºC. Upon stirring at same temperature for 1 h, the reaction mixture was stirred at ambient temperature for 1 h. It was then concentrated under reduced pressure, extracted with ethyl acetate (2 x 50 mL), washed with brine (50 mL), dried over Na_2SO_4, filtered, concentrated, and purified by column chromatography on silica gel (pet ether/ EtOAc: 85:15) to yield ketone as yellow oil (1.8 g, 6.2 mmol, 64.3%).

This compound was prepared in enantiomerically-enriched form following procedure D, using 4-phenyl-1-(phenylsulfonyl)but-3-yn-2-one (143 mg, 0.50 mmol, 1.0 equiv), FA/TEA (0.5 mL), [(*R,R*)Teth-TsDpenRuCl] (3.1 mg, 5.0 x 10^{-3} mmol, 1 mol%) and DCM (3 mL). (*S*)- 4-phenyl-1-(phenylsulfonyl)but-3-yn-2-ol was isolated by flash chromatography (pet ether/ EtOAc: 85:15) as a yellow oil (143 mg, 0.45 mmol, 90.9%).

$[\alpha]_D^{25}$ +26.0° (c 0.1 in $CHCl_3$) 99.4 % ee.

(found (ESI) [M+Na]+, 309.0550. $C_{16}H_{14}NaO_3S$ requires 309.0556)

ν_{max}: 3451 (broad), 3062, 2928, 1446, 2233, 1288, 1133, 1023, 752, 685, 525 cm^{-1}.

^{1}H NMR (400 MHz, $CDCl_3$) δ 8.02 – 7.93 (m, 2H), 7.72 – 7.52 (m, 3H), 7.39 – 7.23 (m, 5H), 5.16 (dd, *J* = 8.4, 2.7 Hz, 1H), 3.71 – 3.53 (m, 2H), 3.33 (d, *J* = 15.3 Hz, 1H).

^{13}C NMR (101 MHz, $CDCl_3$) δ 139.22, 134.19, 131.76, 129.45, 128.97, 128.32, 128.19, 121.58, 86.58, 85.88, 62.00, 58.05.

Enantiomeric excess determined by HPLC analysis (CHIRALCEL OD-H column, hexane 90:10 iPrOH, 1.0 mL/min, T = 25°C, λ = 210 nm, Ketone 23.2 min, *R* enantiomer 45.5 min, *S*-enantiomer 38.8 min). 99.4% ee (*S*).

m/z (ESI) 309.2 ([M + Na]+, 100 %).

4-phenyl-1-(phenylsulfonyl)but-3-yn-2-one 10h

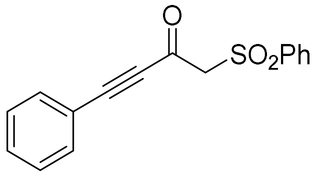

This compound is novel.

To a stirred solution of alkynol (1.0 g, 3.5 mmol, 1 equiv.) in DCM (30 mL) was added activated manganese dioxide (3.3 g, 38 mmol, 11.0 equiv) at room temperature. After 24 h, the reaction mixture was filtered through a celite pad with CH_2Cl_2. The filtrate was concentrated and purified by column chromatography on silica gel to yield the ketone. 4-phenyl-1-(phenylsulfonyl)but-3-yn-2-one was isolated by flash chromatography (pet ether/ EtOAc: 87:13) as a white solid (650 mg, 2.30 mmol, 65.4%).

mp 85-86 °C

(found (ESI) [M+Na]+, 307.0403. $C_{16}H_{12}NaO_3S$ requires 307.0399)

ν_{max}: 2992, 2929, 2204, 1709, 1657, 1321, 1153, 1069, 798, 758, 528 cm^{-1}.

^{1}H NMR (300 MHz, CDCl3) δ 8.00 (d, *J* = 7.6 Hz, 2H), 7.74 – 7.48 (m, 6H), 7.47 – 7.36 (m, 2H), 4.45 (s, 2H).

^{13}C NMR (101 MHz, $CDCl_3$) δ 173.53, 138.65, 134.45, 133.57, 131.70, 129.39, 128.75, 128.63, 118.92, 95.82, 88.01, 69.03.

m/z (ESI) 307.2 ([M + Na]+, 100 %).

Synthesis of 1-phenyl-3-(phenylsulfonyl)propan-2-one 10i

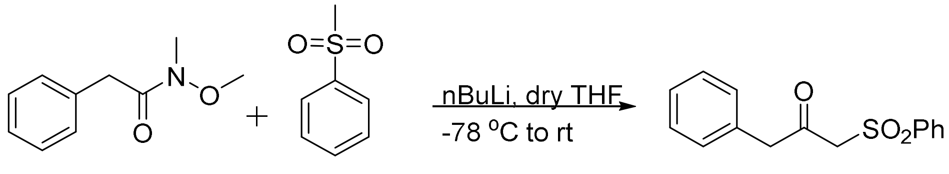

This compound is known and has been fully characterized

To a solution of methyl phenyl sulphone (1.09 g, 7.0 mmol, 1.0 equiv) in dry THF (30 mL) was added *n*BuLi (2.5 M in *n*-hexane, 3.10 mL, 7.70 mmol, 1.1 equiv) dropwise at –78 ºC under nitrogen atmosphere. After the reaction mixture had been stirred at –78 ºC for 30 minutes, N-methoxy-N-methyl-2-phenylacetamide (1.40 g, 7.70 mmol, 1.1 equiv) was added dropwise at -78 ºC. Upon stirring at same temperature for 1 h, the reaction mixture was stirred at ambient temperature for 1 h. It was then concentrated under reduced pressure, extracted with ethyl acetate (2 x 30 mL), washed with brine (50 mL), dried over Na_2SO_4, filtered, concentrated, and purified by column chromatography on silica gel (pet ether/ EtOAc: 80:20) to yield ketone as white solid (700 mg, 2.5 mmol, 36.5%).

mp 89-90 ºC

^{1}H NMR (400 MHz, $CDCl_3$) δ 7.88 (d, *J* = 7.5 Hz, 2H), 7.68 (t, *J* = 7.5 Hz, 1H), 7.57 (t, *J* = 7.7 Hz, 2H), 7.39 – 7.27 (m, 3H), 7.18 (d, *J* = 6.9 Hz, 2H), 4.16 (s, 2H), 3.98 (s, 2H).

^{13}C NMR (101 MHz, $CDCl_3$) δ 195.68, 138.88, 134.36, 132.32, 129.68, 129.39, 129.06, 128.34, 127.66, 65.57, 50.95.

m/z (ESI) 297.2 ([M + Na]+, 100 %).

Racemic and (*S*)-1-phenyl-3-(phenylsulfonyl)propan-2-ol 11i

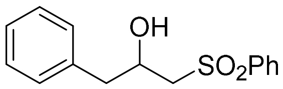

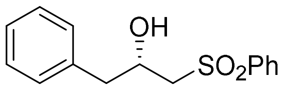

This compound is known and has been fully characterized.

Tanikaga, R.; Hosoya, K.; KaJi, A. *Synthesis,* **1987,** *4,* 389-390.

This compound was prepared in racemic form following procedure C using: 1-phenyl-3-(phenylsulfonyl)propan-2-one (100 mg, 0.4 mmol, 1 equiv), $NaBH_4$ (23 mg, 0.5 mmol, 1.25 equiv) and MeOH (5 mL). 1-phenyl-3-(phenylsulfonyl)propan-2-ol was isolated by flash chromatography (pet ether/ EtOAc: 80:20) as a white solid (80 mg, 0.3 mmol, 79.4%).

This compound was prepared in enantiomerically-enriched form following procedure D, using 1-phenyl-3-(phenylsulfonyl)propan-2-one (137 mg, 0.5 mmol, 1.0 equiv), FA/TEA (0.5 mL), [(*R,R*)Teth-TsDpenRuCl] (3.1 mg, 5.0 x 10^{-3} mmol, 1 mol%) and DCM (3 mL). (*S*)- 1-phenyl-3-(phenylsulfonyl)propan-2-ol was isolated by flash chromatography (pet ether/ EtOAc: 80:20) as a white solid (127 mg, 0.46 mmol, 92.0%).

mp 107-108 ºC.

$[\alpha]_D^{25}$ +23.0º (c 0.1 in $CHCl_3$) 72 % ee (*S*) (lit(above) $[\alpha]_D^{24}$ -31.3º (c 1.0 in $CHCl_3$, 100% ee (*S*).

^{1}H NMR (400 MHz, $CDCl_3$) δ 7.90 – 7.83 (m, 2H), 7.70 – 7.63 (m, 1H), 7.58 – 7.52 (m, 2H), 7.31 – 7.22 (m, 3H), 7.15 – 7.08 (m, 2H), 4.46 – 4.31 (m, 1H), 3.27 – 3.16 (m, 3H), 2.89 (dd, *J* = 13.8, 6.9 Hz, 1H), 2.78 (dd, *J* = 13.8, 6.2 Hz, 1H).

^{13}C NMR (101 MHz, $CDCl_3$) δ 139.18, 136.29, 134.00, 129.40, 128.67, 127.91, 127.36, 126.97, 67.03, 61.25, 42.62.

Enantiomeric excess determined by HPLC analysis (CHIRALPAK IA column, hexane 90:10 iPrOH, 1.0 mL/min, T = 25°C, λ = 210 nm, Ketone 20.9 min, *R* enantiomer 32.3 min, *S*-enantiomer 25.3 min). 72% ee (*S*).

m/z (ESI) 299.2 ([M + Na]+, 100 %).

1-phenoxy-4-phenylbut-3-yn-2-one

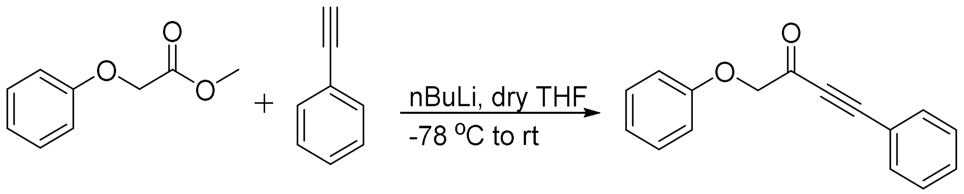

This compound is novel.

To a solution of phenyl acetylene (0.27 g, 2.7 mmol, 0.9 equiv) in dry THF (15 mL) was added *n*BuLi (2.5 M in *n*-hexane, 0.6 mL, 1.2 mmol, 1.2 equiv) dropwise at –78 ºC under nitrogen atmosphere. After the reaction mixture had been stirred at –78 ºC for 30 minutes, methyl 2-phenoxyacetate (0.56 g, 3.0 mmol, 1.0 equiv) was added dropwise at -78 ºC. Upon stirring at same temperature for 1 h, the reaction mixture was stirred at ambient temperature for 1 h. It was then concentrated under reduced pressure, extracted with ethyl acetate (2 x 20 mL), washed with brine (20 mL), dried over Na_2SO_4, filtered, concentrated, and purified by column chromatography on silica gel (pet ether/ EtOAc: 90:10) to yield ketone as colourless oil (0.40 g, 1.7 mmol, 64%).

(found (ESI) [M+Na]+, 259.0729. $C_{16}H_{12}NaO_2$ requires 259.0730)

ν_{max}: 2929, 2855, 1697, 1450, 1275, 1124, 710 cm^{-1}.

^{1}H NMR (400 MHz, $CDCl_3$) δ 7.57 – 7.44 (m, 3H), 7.41 – 7.28 (m, 4H), 7.06 – 6.97 (m, 1H), 6.99 – 6.91 (m, 2H), 4.82 (s, 2H).

^{13}C NMR (101 MHz, $CDCl_3$) δ 183.49, 157.77, 133.32, 131.25, 129.67, 128.71, 121.86, 119.41,114.78, 95.19, 85.76, 73.34.

m/z (ESI) 259.1 ([M + Na]+, 100 %).

Racemic and *(R)*-1-phenoxy-4-phenylbut-3-yn-2-ol

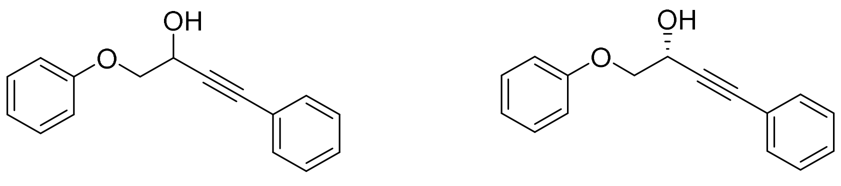

This compound is novel.

This compound was prepared in racemic form following procedure C using: 1-phenoxy-4-phenylbut-3-yn-2-one (100 mg, 0.424 mmol, 1.0 equiv), $NaBH_4$ (23 mg, 0.61 mmol, 1.43 equiv) and MeOH (5 mL). 1-phenoxy-4-phenylbut-3-yn-2-ol was isolated by flash chromatography (pet ether/ EtOAc: 90:10) as a white solid (72 mg, 0.30 mmol, 71%).

This compound was prepared in enantiomerically-enriched form following procedure D, using 1-phenoxy-4-phenylbut-3-yn-2-one (118 mg, 0.5 mmol, 1.0 equiv), FA/TEA (0.5 mL), [(*R,R*)Teth-TsDpenRuCl] (3.1 mg, 5.0 x 10^{-3} mmol, 1 mol%) and DCM (3 mL). (*S*)- 1-phenoxy-4-phenylbut-3-yn-2-ol was isolated by flash chromatography (pet ether/ EtOAc: 90:10) as a white solid (102 mg, 0.428 mmol, 85.7%).

mp 82-83 °C.

$[\alpha]_D^{25}$ +19.0° (c 0.1 in $CHCl_3$) 89.0 % ee.

(found (ESI) [M+Na]+, 261.0886. $C_{16}H_{14}NaO_2$ requires 261.0886)

ν_{max}: 3395 (broad), 3056, 2867, 1599, 1497, 1247, 1078, 1045, 748, 688, 590 cm^{-1}.

^{1}H NMR (400 MHz, $CDCl_3$) δ 7.50 – 7.42 (m, 2H), 7.36 – 7.26 (m, 5H), 7.02 – 6.93 (m, 3H), 5.04 – 4.94 (m, 1H), 4.28 – 4.12 (m, 2H), 2.64 (d, *J* = 5.0 Hz, 1H).

^{13}C NMR (101 MHz, $CDCl_3$) δ 158.26, 131.88, 129.62, 128.76, 128.34, 122.12, 121.52, 114.84, 86.20, 86.01, 71.57, 61.98.

Enantiomeric excess determined by HPLC analysis (CHIRALPAK IC column, hexane 93:07 iPrOH, 0.7 mL/min, T = 25°C, λ = 210 nm, Ketone 8.5 min, *R* enantiomer 14.6 min, *S*-enantiomer 18.6 min). 89.0% ee (*S*).

m/z (ESI) 261.1 ([M + Na]+, 100 %).

Synthesis of *tert*-butyl 2-(2-(phenylsulfonyl)acetyl)cyclopropane-1-carboxylate

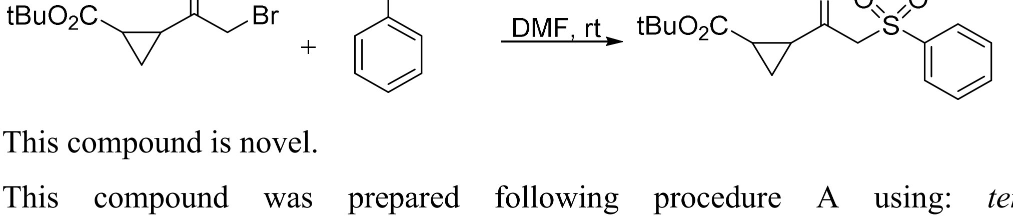

This compound is novel.

This compound was prepared following procedure A using: *tert*-butyl 2-(2-bromoacetyl)cyclopropane-1-carboxylate (2.2 g, 8.1 mmol, 1.0 equiv), benzene sulphinic acid

(1.6 g, 9.8 mmol, 1.2 equiv) and dry DMF (30 mL). tert-butyl 2-(2-(phenylsulfonyl)acetyl)cyclopropane-1-carboxylate was isolated by flash chromatography (pet ether/ EtOAc: 80:20) as a white solid (874 mg, 2.69 mmol, 32.1%).

mp 73-74 °C

(found (ESI) [M+Na]+, 347.0925. $C_{16}H_{20}NaO_5S$ requires 347.0924)

ν_{max}: 2980, 2928, 1723, 1698, 1321, 1138, 1073, 731, 687, 595, 529 cm^{-1}.

^{1}H NMR (400 MHz, $CDCl_3$) δ 7.92 (d, *J* = 7.1 Hz, 2H), 7.69 (t, *J* = 6.8 Hz, 1H), 7.58 (t, *J* = 7.2 Hz, 2H), 4.33 (s, 2H), 2.66 (d, *J* = 3.9 Hz, 1H), 2.14 – 2.02 (m, 1H), 1.55 – 1.35 (m, 11H).

^{13}C NMR (101 MHz, $CDCl_3$) δ 195.77, 169.97, 138.60, 134.42, 129.38, 128.36, 81.71, 68.24, 29.96, 28.05, 27.59, 18.35.

m/z (ESI) 347.2 ([M + Na]+, 100 %).

Racemic and *(S)-tert*-butyl 2-(1-hydroxy-2-(phenylsulfonyl)ethyl)cyclopropane-1-carboxylate 12

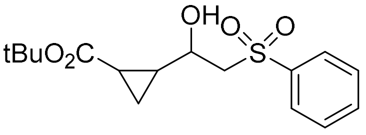

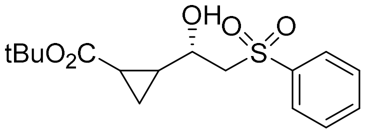

This compound is novel.

This compound was prepared in racemic form following procedure C using: *tert*-butyl 2-(2-(phenylsulfonyl)acetyl)cyclopropane-1-carboxylate (162 mg, 0.50 mmol, 1 equiv), $NaBH_4$ (29 mg, 0.77 mmol, 1.5 equiv) and MeOH (5 mL). *tert*-butyl 2-(1-hydroxy-2-(phenylsulfonyl)ethyl)cyclopropane-1-carboxylate was isolated by flash chromatography (pet ether/ EtOAc: 80:20) as a white solid (130 mg, 0.398 mmol, 79.7%).

This compound was prepared in enantiomerically-enriched form following procedure D, using tert-butyl 2-(2-(phenylsulfonyl)acetyl)cyclopropane-1-carboxylate (162 mg, 0.50 mmol, 1.0 equiv), FA/TEA (0.5 mL), [(*R,R*)Teth-TsDpenRuCl] (3.1 mg, 5.0 x 10^{-3} mmol, 1 mol%) and DCM (3 mL). (*S*)- tert-butyl 2-(1-hydroxy-2-(phenylsulfonyl)ethyl)cyclopropane-1-carboxylate was isolated by flash chromatography (pet ether/ EtOAc: 80:20) as a white solid (148 mg, 0.454 mmol, 90.8%).

mp 117-118 °C.

(found (ESI) [M+Na]+, 349.1078. $C_{16}H_{22}NaO_5S$ requires 349.1080)

ν_{max}: 3406 (broad), 2974, 2882, 1682, 1302, 1145, 1081, 737, 687, 544 cm^{-1}.

^{1}H NMR (400 MHz, $CDCl_3$) δ 7.94 (d, J = 7.2 Hz, 2H), 7.69 (d, J = 6.7 Hz, 1H), 7.62 (d, J = 7.1 Hz, 2H), 3.91 (d, J = 49.8 Hz, 1H), 3.38 – 3.21 (m, 3H), 1.73 – 1.60 (m, 1H), 1.42 (s, 111H), 1.09 – 0.98 (m, 1H), 0.92 – 0.78 (m, 1H).

^{13}C NMR (101 MHz, $CDCl_3$) δ 172.45, 134.21, 134.19, 129.55, 129.52, 127.97, 127.95, 66.83, 65.95, 62.07, 62.04, 28.10, 26.30, 25.78, 18.79, 18.03, 11.44, 11.28.

Enantiomeric excess and diastereomeric ratio determined by HPLC analysis (CHIRALPAK IC column, hexane 80:20 iPrOH, 1.0 mL/min, T = 25°C, λ = 210 nm, Ketone 26.0 and 29.0 min, t_R = 23.2 min (major, d1), 26.3 min (minor, d1), 34.6 min (minor, d2), 68.7 min (major, d2). 53:47 dr, 90.4% ee (d1), 99.8% ee (d2).

m/z (ESI) 349.2 ([M + Na]+, 100 %).

2-(2-Cyclopropyl-2-oxoethyl)isoindoline-1,3-dione

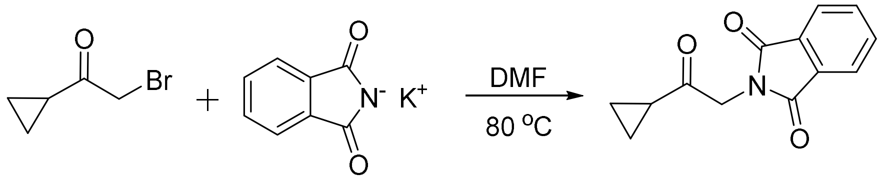

This compound is known and has been fully characterized.

Sueda, T.; Kawada, A.; Urashi, Y.; Teno, N. *Org. Lett.,* **2013,** *15,* 1560-1563.

A mixture of 2-bromo-1-cyclopropylethanone (1.0 g, 6.2 mmol, 1 equiv.) and potassium pthalimide (1.4 g, 7.4 mmol, 1.2 equiv.) in dimethylformamide (25 mL) was stirred at 80°C overnight, then cooled and diluted with water (100 mL). The precipitate was collected by filtration and and purified by column chromatography on silica gel (pet ether/ EtOAc: 80:20) to afford 2-(2-cyclopropyl-2-oxoethyl)isoindoline-1,3-dione (1.1 g, 4.8 mmol, 78%) as a white solid.

mp 145-146 °C

^{1}H NMR (400 MHz, $CDCl_3$) δ 7.92 – 7.82 (m, 2H), 7.79 – 7.68 (m, 2H), 4.67 (s, 2H), 2.08 – 1.96 (m, 1H), 1.16 (d, J = 2.7 Hz, 2H), 1.07 – 0.95 (m, 2H).

^{13}C NMR (101 MHz, $CDCl_3$) δ 202.06, 167.74, 134.12, 132.13, 123.52, 47.09, 18.34, 11.68.

m/z (ESI) 252.2 ([M + Na]+, 100 %).

Racemic and *(S)*-2-(2-cyclopropyl-2-hydroxyethyl)isoindoline-1,3-dione 13

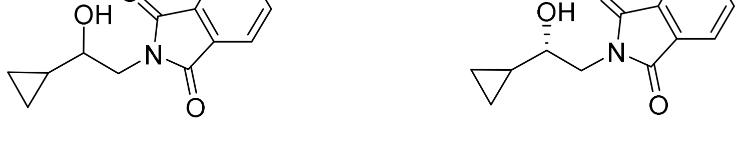

This compound is novel.

This compound was prepared in racemic form following procedure C using: 2-(2-cyclopropyl-2-oxoethyl)isoindoline-1,3-dione (115 mg, 0.502 mmol, 1 equiv), $NaBH_4$ (29 mg, 0.77 mmol, 1.5 equiv) and MeOH (5 mL). 2-(2-cyclopropyl-2-hydroxyethyl)isoindoline-1,3-dione was isolated by flash chromatography (pet ether/ EtOAc: 70:30) as a white solid (62 mg, 0.27 mmol, 53%).

This compound was prepared in enantiomerically-enriched form following procedure D, using 2-(2-cyclopropyl-2-oxoethyl)isoindoline-1,3-dione (115 mg, 0.502 mmol, 1.0 equiv), FA/TEA (0.5 mL), [(*R,R*)Teth-TsDpenRuCl] (3.1 mg, 5.0 x 10^{-3} mmol, 1 mol%) and DCM (3 mL). (*S)*-2-(2-cyclopropyl-2-hydroxyethyl)isoindoline-1,3-dione was isolated by flash chromatography (pet ether/ EtOAc: 70:30) as a white solid (70 mg, 0.30 mmol, 60%).

mp 97-98 °C

$[\alpha]_D^{25}$ +10.0° (c 0.1 in $CHCl_3$) 96.4 % ee.

(found (ESI) [M+Na]+, 254.0784. $C_{13}H_{11}NNaO_3$ requires 254.0793)

ν_{max}: 3529 (broad), 2988 (broad), 2901, 1688, 1388, 717, 530 cm^{-1}.

^1H NMR (400 MHz, $CDCl_3$) δ 7.86 (s, 2H), 7.73 (d, *J* = 2.8 Hz, 2H), 3.91 (d, *J* = 4.7 Hz, 2H), 3.29 (s, 1H), 2.27 (s, 1H), 0.93 (dd, *J* = 11.7, 7.9 Hz, 1H), 0.53 (d, *J* = 8.1 Hz, 2H), 0.34 (d, *J* = 15.0 Hz, 2H).

^{13}C NMR (101 MHz, $CDCl_3$) δ 166.52, 131.75, 129.64, 121.07, 72.03, 41.71, 13.10, -0.00, -0.30.

Enantiomeric excess determined by HPLC analysis (CHIRALPAK IA column, hexane 90:10 iPrOH, 1.0 mL/min, T = 25°C, λ = 210 nm, Ketone 18.3 min, *R* enantiomer 18.5 min, *S*-enantiomer 23.3 min). 96.4% ee (*S*).

m/z (ESI) 254.2 ([M + Na]+, 100 %).

Racemic and *(S)*-1-Phenoxy-3-(phenylsulfonyl)propan-2-ol 14

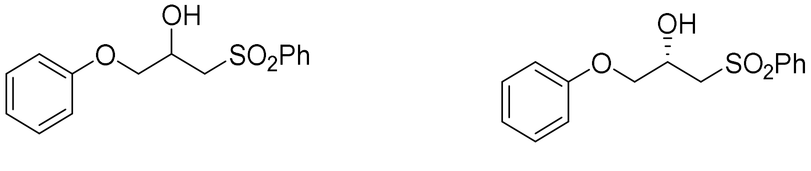

This compound is known and has been fully characterized

Murthy, S. N.; Madhav. B.; Reddy, V. P.; Rao, K. R.; Nageswar, Y. V. D. *Tetrahedron Lett.,* **2009**, *50*, 5009-5011.

This compound was prepared following procedure E using: 2-(phenoxymethyl)oxirane (0.67 g, 5.0 mmol, 1.0 equiv), benzene sulphinic acid (0.90 g, 5.5 mmol, 1.1 equiv) and water (30 mL). 1-phenoxy-3-(phenylsulfonyl)propan-2-ol was isolated by flash chromatography (pet ether/ EtOAc: 25:75) as a white solid (635 mg, 2.20 mmol, 43.5%).

This compound was prepared in enantiomerically-enriched form following procedure D, using 1-phenoxy-3-(phenylsulfonyl)propan-2-one (130 mg, 0.45 mmol, 1.0 equiv), FA/TEA (0.5 mL), [(*R,R*)Teth-TsDpenRuCl] (3.0 mg, 4.8 x 10^{-3} mmol, 1 mol%) and DCM (3 mL). (*S*)- 1-phenoxy-3-(phenylsulfonyl)propan-2-ol was isolated by flash chromatography (pet ether/ EtOAc: 25:75) as a white solid (113 mg, 0.387 mmol, 86.3%).

mp 76-77 °C

$[\alpha]_D^{25}$ +22.0° (c 0.1 in $CHCl_3$) 96.4 % ee.

^1H NMR (400 MHz, $CDCl_3$) δ 8.02 – 7.91 (m, 2H), 7.75 – 7.64 (m, 1H), 7.64 – 7.53 (m, 2H), 7.34 – 7.19 (m, 2H), 7.05 – 6.91 (m, 1H), 6.90 – 6.77 (m, 2H), 4.54 (d, *J* = 3.3 Hz, 1H), 4.08 – 3.92 (m, 2H), 3.54 – 3.41 (m, 2H), 3.37 (s, 1H).

^{13}C NMR (101 MHz, $CDCl_3$) δ 157.94, 139.14, 134.16, 129.58, 129.50, 128.01, 121.56, 114.55, 69.98, 65.18, 59.44.

Enantiomeric excess determined by HPLC analysis (CHIRALCEL OD-H column, hexane 80:20 iPrOH, 1.0 mL/min, T = 25°C, λ = 210 nm, Ketone 63.5 min, *R* enantiomer 28.6 min, *S*-enantiomer 21.8 min). 96.4% ee (*S*).

m/z (ESI) 315.2 ([M + Na]+, 100 %).

1-Phenoxy-3-(phenylsulfonyl)propan-2-one

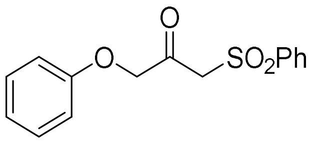

This compound is known but not fully characterized.

Bernard, A. M.; Frongia, A.; Piras, P. P.; Secci, F.; Spiga, M. *Tetrahedron Lett.,* **2008**, *49*, 3037-3041

This compound was prepared following procedure F using: 1-phenoxy-3-(phenylsulfonyl)propan-2-ol (0.701 g, 2.41 mmol, 1 equiv), PCC (1.03 g, 4.80 mmol, 2.0 equiv) and DCM (20 mL). 1-phenoxy-3-(phenylsulfonyl)propan-2-one was isolated by flash chromatography (pet ether/ EtOAc: 80:20) as a white solid (332 mg, 1.14 mmol, 47.7%).

This compound also made by using this procedure: To a solution of methyl phenyl sulphone (1.0 g, 6.41 mmol, 1.0 equiv) in dry THF (30 mL) was added *n*BuLi (2.5 M in *n*-hexane, 3.30

mL, 7.70 mmol, 1.2 equiv) dropwise at –78 ºC under nitrogen atmosphere. After the reaction mixture had been stirred at –78 ºC for 30 minutes, *N*-methoxy-*N*-methyl-2-phenoxyacetamide (1.50 g, 7.70 mmol, 1.2 equiv) was added dropwise at -78 ºC. Upon stirring at same temperature for 1 h, the reaction mixture was stirred at ambient temperature for 1 h. It was then concentrated under reduced pressure, extracted with ethyl acetate (2 x 30 mL), washed with brine (50 mL), dried over Na_2SO_4, filtered, concentrated, and purified by column chromatography on silica gel (pet ether/ EtOAc: 80:20) to yield ketone as white solid (690 mg, 2.38 mmol, 37.1%).
mp 86-87 ºC
^{1}H NMR (400 MHz, $CDCl_3$) δ 7.88 – 7.78 (m, 2H), 7.67 – 7.55 (m, 1H), 7.54 – 7.43 (m, 2H), 7.30 – 7.13 (m, 2H), 7.00 – 6.89 (m, 1H), 6.86 – 6.72 (m, 2H), 4.65 (s, 2H), 4.32 (s, 2H).
^{13}C NMR (101 MHz, $CDCl_3$) δ 195.16, 157.16, 138.71, 134.45, 129.78, 129.42, 128.40, 122.23, 114.64, 72.86, 63.27.
m/z (ESI) 313.2 ([M + Na]+, 100 %).

Racemic and *(S)*-1-Phenoxy-3-(phenylthio)propan-2-ol

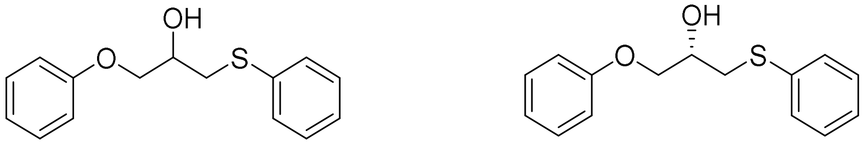

This compound is known and has been fully characterized.
Su, W.; Chen, J.; Wu, H.; Jin, C. *J. Org. Chem.,* **2007**, *72*, 4524-4527.
This compound was prepared following procedure E using: 2-(phenoxymethyl)oxirane (0.67 g, 5.0 mmol, 1 equiv), Thiophenol (0.60 g, 5.5 mmol, 1.1 equiv) and water (20 mL). 1-phenoxy-3-(phenylthio)propan-2-ol was isolated by flash chromatography (pet ether/ EtOAc: 90:10) as a colourless oil (1.2 g, 4.6 mmol, 92%).
This compound was prepared in enantiomerically-enriched form following procedure D, using 1-phenoxy-3-(phenylthio)propan-2-one (40 mg, 0.15 mmol, 1.0 equiv), FA/TEA (0.5 mL), [(*R,R*)Teth-TsDpenRuCl] (1.1 mg, 1.7 x 10^{-3} mmol, 1 mol%) and DCM (2 mL). (*S*)- 1-phenoxy-3-(phenylthio)propan-2-ol was isolated by flash chromatography (pet ether/ EtOAc: 90:10) as a colourless oil (33 mg, 0.13 mmol, 82%).
$[\alpha]_D^{25}$ +14.0º (c 0.1 in $CHCl_3$) 80.8 % ee.
^{1}H NMR (400 MHz, $CDCl_3$) δ 7.46 – 7.37 (m, 2H), 7.34 – 7.24 (m, 4H), 7.24 – 7.17 (m, 1H), 7.01 – 6.93 (m, 1H), 6.92 – 6.83 (m, 2H), 4.18 – 3.97 (m, 3H), 3.31 – 3.09 (m, 2H), 2.69 (s, 1H).

^{13}C NMR (101 MHz, $CDCl_3$) δ 158.37, 135.11, 129.89, 129.54, 129.15, 126.69, 121.28, 114.57, 70.07, 68.62, 37.65.

Enantiomeric excess determined by HPLC analysis (CHIRALCEL OD-H column, hexane 90:10 iPrOH, 1.0 mL/min, T = 25°C, λ = 210 nm, Ketone 19.2 min, *R* enantiomer 16.1 min, *S*-enantiomer 24.8 min). 80.8% ee (*R*).

m/z (ESI) 283.2 ([M + Na]+, 100 %).

1-Phenoxy-3-(phenylthio)propan-2-one

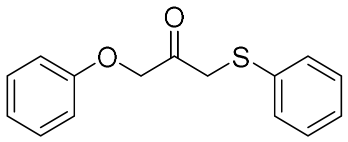

This compound is known and has been fully characterized.

Apparao, S.; Schmidt, R. R. *Synthesis,* **1987,** 896-899.

This compound was prepared following procedure F using: 1-phenoxy-3-(phenylthio)propan-2-ol (0.6 g, 2.3 mmol, 1 equiv), PCC (1.0 g, 4.6 mmol, 2.0 equiv) and DCM (30 mL). 1-phenoxy-3-(phenylthio)propan-2-one was isolated by flash chromatography (pet ether/ EtOAc: 95:05) as a colourless oil (60 mg, 0.23 mmol, 10%).

^{1}H NMR (400 MHz, $CDCl_3$) δ 7.43 – 7.33 (m, 2H), 7.34 – 7.23 (m, 5H), 7.06 – 6.93 (m, 1H), 6.89 – 6.78 (m, 2H), 4.73 (s, 2H), 3.87 (s, 2H).

^{13}C NMR (101 MHz, $CDCl_3$) δ 201.79, 157.58, 134.30, 130.10, 129.72, 129.27, 127.27, 121.90, 114.58, 71.36, 40.82.

m/z (ESI) 281.2 ([M + Na]+, 100 %).

Racemic and *(S)*-1-Isopropoxy-3-(phenylsulfonyl)propan-2-ol 15

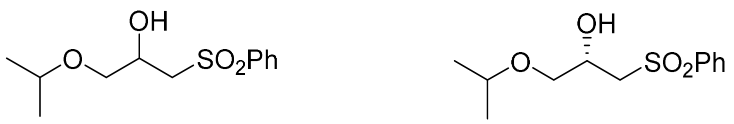

This compound is known and has been fully characterized.

Murthy, S. N.; Madhav. B.; Reddy, V. P.; Rao, K. R.; Nageswar, Y. V. D. *Tetrahedron Lett.,* **2009**, *50*, 5009-5011.

This compound was prepared following procedure E using: 2-(isopropoxymethyl)oxirane (2.01 g, 17.2 mmol, 1.0 equiv), benzene sulphinic acid (3.10 g, 19.0 mmol, 1.10 equiv) and water (50 mL). 1-isopropoxy-3-(phenylsulfonyl)propan-2-ol was isolated by flash chromatography (pet ether/ EtOAc: 25:75) as a colourless oil (1.47 g, 5.70 mmol, 33.1%).

This compound was prepared in enantiomerically-enriched form following procedure D, using 1-isopropoxy-3-(phenylsulfonyl)propan-2-one (128 mg, 0.50 mmol, 1.0 equiv), FA/TEA (0.5 mL), [(*R,R*)Teth-TsDpenRuCl] (3.1 mg, 5.0 x 10^{-3} mmol, 1 mol%) and DCM (3 mL). (*S*)- 1-isopropoxy-3-(phenylsulfonyl)propan-2-ol was isolated by flash chromatography (pet ether/ EtOAc: 25:75) as a colourless oil (112 mg, 0.43 mmol, 86.8%).

$[\alpha]_D^{25}$ +30.0° (c 0.1 in $CHCl_3$) 98.6 % ee.

^{1}H NMR (400 MHz, $CDCl_3$) δ 7.95 (d, *J* = 7.3 Hz, 2H), 7.68 (t, *J* = 6.9 Hz, 1H), 7.59 (t, *J* = 7.3 Hz, 2H), 4.26 (d, *J* = 2.6 Hz, 1H), 3.55 (dd, *J* = 11.7, 5.8 Hz, 1H), 3.46 (d, *J* = 1.8 Hz, 2H), 3.40 – 3.28 (m, 2H), 3.18 (s, 1H), 1.11 (t, *J* = 6.5 Hz, 6H).

^{13}C NMR (101 MHz, $CDCl_3$) δ 139.44, 133.96, 129.37, 128.04, 72.42, 70.33, 65.63, 59.62, 21.93.

Enantiomeric excess determined by HPLC analysis (CHIRALPAK IC column, hexane 80:20 iPrOH, 1.0 mL/min, T = 25°C, λ = 210 nm, Ketone 47.8 min, *R* enantiomer 28.7 min, *S*-enantiomer 37.2 min). 98.6% ee (*S*).

m/z (ESI) 281.2 ([M + Na]+, 100 %).

1-Isopropoxy-3-(phenylsulfonyl)propan-2-one

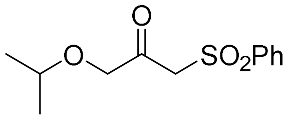

This compound is novel.

This compound was prepared following F procedure using: 1-isopropoxy-3-(phenylsulfonyl)propan-2-ol (1.40 g, 5.40 mmol, 1.0 equiv), PCC (2.30 g, 10.8 mmol, 2.0 equiv) and DCM (40 mL). 1-isopropoxy-3-(phenylsulfonyl)propan-2-one was isolated by flash chromatography (pet ether/ EtOAc: 80:20) as a white solid (1.02 g, 3.95 mmol, 73.4%).

mp 40-41 °C

(found (ESI) [M+Na]+, 279.0662. $C_{12}H_{16}NaO_4S$ requires 279.0662)

ν_{max}: 2987, 1726, 1324, 1145, 1080, 733, 685, 607, 505 cm^{-1}.

^{1}H NMR (400 MHz, $CDCl_3$) δ 7.91 (d, *J* = 7.2 Hz, 2H), 7.69 (t, *J* = 7.1 Hz, 1H), 7.59 (t, *J* = 7.3 Hz, 2H), 4.34 (s, 2H), 4.14 (s, 2H), 3.69 – 3.56 (m, 1H), 1.18 (d, *J* = 5.0 Hz, 6H).

^{13}C NMR (101 MHz, $CDCl_3$) δ 197.35, 138.96, 134.29, 129.33, 128.40, 73.80, 73.16, 62.88, 21.82.

m/z (ESI) 279.2 ([M + Na]+, 100 %).

Racemic and *(S)*-1-(Allyloxy)-3-(phenylsulfonyl)propan-2-ol 16

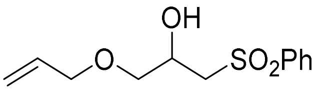

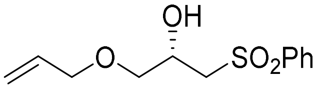

This compound is novel.

This compound was prepared following procedure E using: 2-((allyloxy)methyl)oxirane (2.01 g, 17.5 mmol, 1.0 equiv), benzene sulphinic acid (3.10 g, 19.0 mmol, 1.10 equiv) and water (50 mL). 1-(allyloxy)-3-(phenylsulfonyl)propan-2-ol was isolated by flash chromatography (pet ether/ EtOAc: 25:75) as a colourless oil (1.27 g, 4.96 mmol, 28.3%).

This compound was prepared in enantiomerically-enriched form following procedure D, using 1-(allyloxy)-3-(phenylsulfonyl)propan-2-one (127 mg, 0.50 mmol, 1.0 equiv), FA/TEA (0.5 mL), [(*R,R*)Teth-TsDpenRuCl] (3.1 mg, 5.0 x 10^{-3} mmol, 1 mol%) and DCM (3 mL). (*S*)- 1-(allyloxy)-3-(phenylsulfonyl)propan-2-ol was isolated by flash chromatography (pet ether/ EtOAc: 25:75) as a colourless oil (116 mg, 0.453 mmol, 90.6%).

$[\alpha]_D^{25}$ +18.0° (c 0.1 in $CHCl_3$) 95.4 % ee.

(found (ESI) [M+Na]+, 279.0664. $C_{12}H_{16}NaO_4S$ requires 279.0662)

ν_{max}: 3486 (broad), 2921, 2932, 1447, 1290, 1138, 1080, 747, 686, 526 cm^{-1}.

^{1}H NMR (400 MHz, $CDCl_3$) δ 7.95 (d, *J* = 7.2 Hz, 2H), 7.69 (t, *J* = 6.9 Hz, 1H), 7.59 (t, *J* = 7.2 Hz, 2H), 5.91 – 5.74 (m, 1H), 5.30 – 5.11 (m, 2H), 4.31 (s, 1H), 4.00 (d, *J* = 17.9 Hz, 2H), 3.49 (s, 2H), 3.35 (d, *J* = 4.2 Hz, 2H), 3.25 (s, 1H).

^{13}C NMR (101 MHz, $CDCl_3$) δ 139.33, 134.03, 129.42, 128.02, 117.69, 72.38, 72.25, 65.46, 59.52.

Enantiomeric excess determined by HPLC analysis (CHIRALPAK IC column, hexane 80:20 iPrOH, 1.0 mL/min, T = 25°C, λ = 210 nm, Ketone 40.8 min, *R* enantiomer 34.0 min, *S*-enantiomer 51.5 min). 95.4% ee (*S*).

m/z (ESI) 279.2 ([M + Na]+, 100 %).

1-(Allyloxy)-3-(phenylsulfonyl)propan-2-one

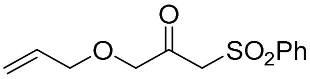

This compound is novel.

This compound was prepared following procedure F using: 1-(allyloxy)-3-(phenylsulfonyl)propan-2-ol (1.2 g, 4.7 mmol, 1 equiv), PCC (2.0 g, 9.4 mmol, 2.0 equiv) and DCM (40 mL). 1-(allyloxy)-3-(phenylsulfonyl)propan-2-one was isolated by flash chromatography (pet ether/ EtOAc: 80:20) as a white solid (0.87 g, 3.4 mmol, 73%).

mp 75-76 ºC

(found (ESI) [M+Na]+, 277.0502. $C_{12}H_{14}NaO_4S$ requires 277.0498)

ν_{max}: 3065, 2957, 1735, 1447, 1287, 1145, 1018, 783, 688, 523 cm^{-1}.

^{1}H NMR (400 MHz, $CDCl_3$) δ 7.91 (d, *J* = 7.2 Hz, 2H), 7.70 (t, *J* = 6.8 Hz, 1H), 7.59 (t, *J* = 7.2 Hz, 2H), 5.97 – 5.77 (m, 1H), 5.38 – 5.19 (m, 2H), 4.30 (s, 2H), 4.19 (s, 2H), 4.04 (d, *J* = 4.0 Hz, 2H).

13C NMR (101 MHz, $CDCl_3$) δ 196.55, 138.80, 134.37, 133.32, 129.37, 128.39, 118.62, 75.13, 72.53, 63.14.

m/z (ESI) 277.1 ([M + Na]+, 100 %).

1-Phenoxy-4-(phenylsulfonyl)butan-2-ol 17

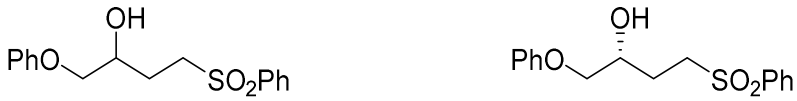

This compound is novel.

To a solution of methyl phenyl sulphone (1.01 g, 6.47 mmol, 1.0 equiv) in dry THF (30 mL) was added *n*BuLi (2.5 M in *n*-hexane, 4.30 mL, 8.70 mmol, 1.2 equiv) dropwise at –78 ºC under nitrogen atmosphere. After the reaction mixture had been stirred at –78 ºC for 30 minutes, 2-(phenoxymethyl)oxirane (1.14 g, 7.30 mmol, 1.10 equiv) was added dropwise at -78 ºC. Upon stirring at same temperature for 1 h, the reaction mixture was stirred at ambient temperature for 1 h. It was then concentrated under reduced pressure, extracted with ethyl acetate (2 x 30 mL), washed with brine (50 mL), dried over Na_2SO_4, filtered, concentrated, and purified by column chromatography on silica gel (pet ether/ EtOAc: 75:25) to yield alcohol as white solid (1.23 g, 4.01 mmol, 60.7%).

This compound was prepared in enantiomerically-enriched form following procedure D, using 1-phenoxy-4-(phenylsulfonyl)butan-2-one (152 mg, 0.50 mmol, 1.0 equiv), FA/TEA (0.5 mL), [(*R,R*)Teth-TsDpenRuCl] (3.1 mg, 5.0 x 10^{-3} mmol, 1 mol%) and DCM (3 mL). (*S*)- 1-phenoxy-4-(phenylsulfonyl)butan-2-ol was isolated by flash chromatography (pet ether/ EtOAc: 75:25) as a white solid (126 mg, 0.412 mmol, 82.3%).

mp 100-101 ºC

$[\alpha]_D^{25}$ +3.0º (c 0.1 in $CHCl_3$) 27.0 % ee.

(found (ESI) [M+Na]+, 329.0821. $C_{16}H_{18}NaO_4S$ requires 329.0818)

ν_{max}: 3520, 3076, 1598, 1494, 1286, 1142, 741, 684, 580 cm^{-1}.

^{1}H NMR (400 MHz, $CDCl_3$) δ 7.91 – 7.79 (m, 2H), 7.67 – 7.55 (m, 1H), 7.56 – 7.44 (m, 2H), 7.29 – 7.13 (m, 2H), 7.00 – 6.84 (m, 1H), 6.86 – 6.73 (m, 2H), 4.07 – 3.95 (m, 1H), 3.88 (dd, *J* = 9.4, 3.7 Hz, 1H), 3.77 (dd, *J* = 9.4, 6.7 Hz, 1H), 3.41 – 3.28 (m, 1H), 3.26 – 3.15 (m, 1H), 2.46 (d, *J* = 4.7 Hz, 1H), 2.06 – 1.83 (m, 2H).
^{13}C NMR (101 MHz, CDCl3) δ 158.21, 139.03, 133.84, 129.61, 129.40, 128.06, 121.45, 114.53, 71.40, 68.34, 52.88, 26.38.
Enantiomeric excess determined by HPLC analysis (CHIRALPAK IA column, hexane 90:10 iPrOH, 1.0 mL/min, T = 25°C, λ = 210 nm, Ketone 34.4 min, *R* enantiomer 23.4 min, *S*-enantiomer 19.9 min). 27.0% ee (*S*).
m/z (ESI) 329.2 ([M + Na]+, 100 %).

1-Phenoxy-4-(phenylsulfonyl)butan-2-one

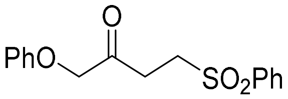

This compound is novel.
This compound was prepared following procedure F using: 1-phenoxy-4-(phenylsulfonyl)butan-2-ol (1.20 g, 3.94 mmol, 1 equiv), PCC (1.70 g, 7.91 mmol, 2.0 equiv) and DCM (30 mL). 1-phenoxy-4-(phenylsulfonyl)butan-2-one was isolated by flash chromatography (pet ether/ EtOAc: 80:20) as a white solid (683 mg, 2.23 mmol, 57.3%).
mp 93-94 °C.
(found (ESI) [M+Na]+, 327.0659. $C_{16}H_{16}NaO_4S$ requires 327.0662)
ν_{max}: 3057, 2995, 1735, 1490, 1249, 1143, 1068, 682, 579, 529 cm^{-1}.
^{1}H NMR (400 MHz, CDCl3) δ 7.98 – 7.88 (m, 2H), 7.73 – 7.65 (m, 1H), 7.64 – 7.54 (m, 2H), 7.39 – 7.29 (m, 2H), 7.04 (t, *J* = 7.4 Hz, 1H), 6.89 (d, *J* = 7.9 Hz, 2H), 4.58 (s, 2H), 3.47 (t, *J* = 7.4 Hz, 2H), 3.16 (t, *J* = 7.4 Hz, 2H).
^{13}C NMR (101 MHz, CDCl3) δ 203.83, 157.40, 138.82, 134.03, 129.82, 129.46, 128.05, 122.08, 114.50, 72.53, 50.07, 32.15.
m/z (ESI) 327.2 ([M + Na]+, 100 %).

Racemic and *(S)-tert*-Butyl (2-hydroxy-3-(phenylsulfonyl)propyl)carbamate 18

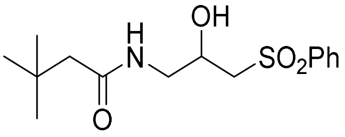

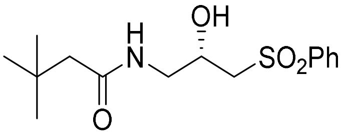

This compound is novel.

This compound was prepared following procedure E using: tert-butyl (oxiran-2-ylmethyl)carbamate (1.01 g, 5.80 mmol, 1 equiv), benzene sulphinic acid (1.11 g, 6.90 mmol, 1.20 equiv) and water (25 mL). tert-butyl (2-hydroxy-3-(phenylsulfonyl)propyl)carbamate was isolated by flash chromatography (pet ether/ EtOAc: 70:30) as a white solid (614 mg, 1.95 mmol, 33.7%).

This compound was prepared in enantiomerically-enriched form following procedure D, using tert-butyl (2-oxo-3-(phenylsulfonyl)propyl)carbamate (85 mg, 0.27 mmol, 1.0 equiv), FA/TEA (0.3 mL), [(*R,R*)Teth-TsDpenRuCl] (1.5 mg, 2.4 x 10^{-3} mmol, 1 mol%) and DCM (2 mL). (*S)*-tert-butyl (2-hydroxy-3-(phenylsulfonyl)propyl)carbamate was isolated by flash chromatography (pet ether/ EtOAc: 70:30) as a white solid (72 mg, 0.23 mmol, 84%).

mp 110-111 °C

$[\alpha]_D^{25}$ +9.0° (c 0.1 in $CHCl_3$) 53.5 % ee.

(found (ESI) [M+Na]+, 338.1035. $C_{14}H_{21}NNaO_5S$ requires 338.1033)

ν_{max}: 3374 (broad), 2978, 2921, 1673, 1536, 1283, 1136, 1082, 744, 683, 573, 527 cm^{-1}.

^1H NMR (400 MHz, $CDCl_3$) δ 7.90 – 7.79 (m, 2H), 7.62 (t, *J* = 7.4 Hz, 1H), 7.53 (t, *J* = 7.6 Hz, 2H), 4.96 (s, 1H), 4.20 – 4.03 (m, 1H), 3.90 (s, 1H), 3.32 – 3.11 (m, 4H), 1.35 (s, 9H).

^{13}C NMR (101 MHz, $CDCl_3$) δ 156.83, 139.06, 134.19, 129.53, 127.93, 80.14, 66.09, 59.50, 45.31, 28.30.

Enantiomeric excess determined by HPLC analysis (CHIRALPAK IA column, hexane 90:10 H, 0.7 mL/min, T = 25°C, λ = 210 nm, Ketone 37.4 min, *R* enantiomer 21.7 min, *S*-enantiomer 16.4 min). 53.5% ee (*S*).

m/z (ESI) 338.2 ([M + Na]+, 100 %).

***tert*-Butyl 2-oxo-3-(phenylsulfonyl)propyl)carbamate**

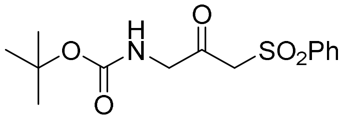

This compound is novel.

This compound was prepared following procedure F using: tert-butyl (2-hydroxy-3-(phenylsulfonyl)propyl)carbamate (0.41 g, 1.3 mmol, 1 equiv), PCC (0.56 g, 2.6 mmol, 2.0 equiv) and DCM (10 mL). tert-butyl (2-oxo-3-(phenylsulfonyl)propyl)carbamate was isolated by flash chromatography (pet ether/ EtOAc: 70:30) as a white solid (0.28 g, 0.89 mmol, 69%).

mp 156-157 °C

(found (ESI) [M+Na]+, 336.0879. $C_{14}H_{19}NNaO_5S$ requires 336.0876)

ν_{max}: 3378 (sharp), 2974, 2928, 1744, 1689, 1520 1285, 1145, 752, 683, 572, 527 cm^{-1}.

^{1}H NMR (400 MHz, $CDCl_3$) δ 7.89 (d, J = 7.4 Hz, 2H), 7.70 (t, J = 7.5 Hz, 1H), 7.59 (t, J = 7.7 Hz, 2H), 5.20 (s, 1H), 4.25 – 4.15 (m, 4H), 1.44 (s, 9H).

^{13}C NMR (101 MHz, $CDCl_3$) δ 194.71, 151.61, 138.41, 134.54, 129.47, 128.34, 80.41, 64.44, 51.61, 28.27.

m/z (ESI) 336.2 ([M + Na]+, 100 %).

Racemic and (*1S,2S*)-3-phenyl-1-(phenylsulfonyl)-1-(p-tolyl)propan-2-ol 20a

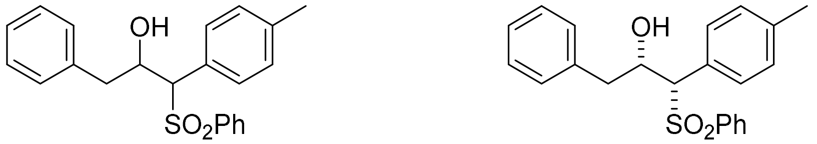

This compound is novel.

To a solution of 1-methyl-4-((phenylsulfonyl)methyl)benzene (1.20 g, 4.87 mmol, 1.0 equiv) in dry THF (35 mL) was added *n*BuLi (2.5 M in *n*-hexane, 2.63 mL, 5.36 mmol, 1.1 equiv) dropwise at –78 ºC under nitrogen atmosphere. After the reaction mixture had been stirred at –78 ºC for 30 minutes, 2-phenylacetaldehyde (643 mg, 5.36 mmol, 1.0 equiv) was added dropwise at -78 ºC. Upon stirring at same temperature for 1 h, the reaction mixture was stirred at ambient temperature for 1 h. It was then concentrated under reduced pressure, extracted with ethyl acetate (2 x 40 mL), washed with brine (80 mL), dried over Na_2SO_4, filtered, concentrated, and purified by column chromatography on silica gel (pet ether/ EtOAc: 80:20) to yield 3-phenyl-1-(phenylsulfonyl)-1-(p-tolyl)propan-2-ol as white solid (664 mg, 1.80 mmol, 37.2%).

This compound was prepared in enantiomerically-enriched form following procedure D, using 3-phenyl-1-(phenylsulfonyl)-1-(p-tolyl)propan-2-one (100 mg, 0.275 mmol, 1.0 equiv), FA/TEA (0.25 mL), [(*R,R*)Teth-TsDpenRuCl] (1.5 mg, 2.4 x 10^{-3} mmol, 1 mol%) and DCM (2 mL). (1S,2S)-3-phenyl-1-(phenylsulfonyl)-1-(p-tolyl)propan-2-ol was isolated by flash chromatography (pet ether/ EtOAc: 80:20) as a white solid (96 mg, 0.26 mmol, 95%).

mp 136-137 ºC.

$[\alpha]_D^{25}$ +51.0º (c 0.1 in $CHCl_3$) >99.9% ee.

(found (ESI) [M+Na]+, 389.1185. $C_{22}H_{22}NaO_3S$ requires 389.1182).

ν_{max}: 3480 (broad), 2926, 2867, 1447, 1290, 1138, 1080, 747, 686, 526 cm^{-1}.

^{1}H NMR (400 MHz, $CDCl_3$) δ 7.59 – 7.48 (m, 3H), 7.40 – 7.32 (m, 2H), 7.28 – 7.19 (m, 5H), 7.09 – 7.02 (m, 4H), 5.03 (t, J = 7.0 Hz, 1H), 3.92 (d, J = 1.6 Hz, 1H), 3.18 (d, J = 2.2 Hz, 1H), 2.88 – 2.75 (m, 1H), 2.65 – 2.52 (m, 1H), 2.33 (s, 3H).

^{13}C NMR (101 MHz, $CDCl_3$) δ 139.00, 137.73, 136.97, 133.60, 131.53, 129.31, 129.17, 128.78, 128.70, 128.61, 126.81, 125.98, 73.07, 70.27, 40.89, 21.23.
Enantiomeric excess and diastereomeric ratio determined by HPLC analysis (CHIRALPAK IA column, hexane 90:10 iPrOH, 0.5 mL/min, T = 25°C, λ = 210 nm, Ketone 36.9, t_R = 28.4 min (maJor, d1), 34.7 min (major, d2), 42.7 min (minor, d2). 96.6:3.4 dr, >99.9% ee (d1).
m/z (ESI) 389.2 ([M + Na]+, 100 %).

3-phenyl-1-(phenylsulfonyl)-1-(p-tolyl)propan-2-one 19a

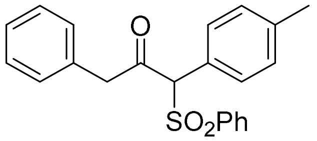

This compound is Novel.
This compound was prepared following procedure F using: 3-phenyl-1-(phenylsulfonyl)-1-(p-tolyl)propan-2-ol (664 mg, 1.80 mmol, 1 equiv), PCC (780 mg, 3.60 mmol, 2.0 equiv) and DCM (20 mL). 3-phenyl-1-(phenylsulfonyl)-1-(p-tolyl)propan-2-one was isolated by flash chromatography (pet ether/ EtOAc: 80:20) as a yellow solid (402 mg, 1.10 mmol, 60.9%).
mp 160-161 °C.
(found (ESI) [M+Na]+, 387.1023. $C_{22}H_{20}NaO_3S$ requires 387.1025).
ν_{max}: 2935, 1721, 1315, 1139, 1034, 725, 689, 597, 514 cm^{-1}.
^1H NMR (400 MHz, $CDCl_3$) δ 7.58 – 7.54 (m, 3H), 7.42 – 7.35 (m, 2H), 7.29 – 7.23 (m, 3H), 7.10 – 7.01 (m, 6H), 5.29 (s, 1H), 3.90 – 3.78 (m, 2H), 2.33 (s, 3H).
^{13}C NMR (101 MHz, $CDCl_3$) δ 198.00, 139.81, 136.72, 133.91, 132.46, 130.33, 129.96, 129.65, 129.49, 128.90, 128.49, 127.50, 124.51, 77.88, 51.00, 21.24.
m/z (ESI) 387.2 ([M + Na]+, 100 %).

Racemic and (*2S,3R*)-1-phenoxy-3-(phenylsulfonyl)butan-2-ol 20b

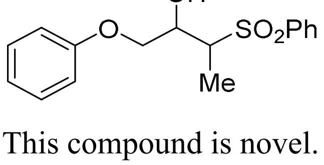

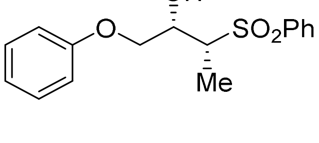

This compound is novel.
To a solution of ethyl phenyl sulphone (573 mg, 3.37 mmol, 1.0 equiv) in dry THF (15 mL) was added *n*BuLi (2.5 M in *n*-hexane, 2.0 mL, 4.0 mmol, 1.2 equiv) dropwise at –78 °C under nitrogen atmosphere. After the reaction mixture had been stirred at –78 °C for 30 minutes, 2-phenoxyacetaldehyde (500 mg, 3.68 mmol, 1.1 equiv) was added dropwise at -78 °C. Upon

stirring at same temperature for 1 h, the reaction mixture was stirred at ambient temperature for 1 h. It was then concentrated under reduced pressure, extracted with ethyl acetate (2 x 20 mL), washed with brine (50 mL), dried over Na_2SO_4, filtered, concentrated, and purified by column chromatography on silica gel (pet ether/ EtOAc: 80:20) to yield 1-phenoxy-3-(phenylsulfonyl)butan-2-ol as colourless oil (700 mg, 2.29 mmol, 67.9%).

This compound was prepared in enantiomerically-enriched form following procedure D, using 1-phenoxy-3-(phenylsulfonyl)butan-2-one (152 mg, 0.50 mmol, 1.0 equiv), FA/TEA (0.5 mL), [(*R,R*)Teth-TsDpenRuCl] (3.1 mg, 5.0 x 10^{-3} mmol, 1 mol%) and DCM (3 mL). (*S*)- 1-phenoxy-3-(phenylsulfonyl)butan-2-ol was isolated by flash chromatography (pet ether/ EtOAc: 75:25) as a colourless oil (146 mg, 0.477 mmol, 95.4%).

$[\alpha]_D^{25}$ +43.0° (c 0.1 in $CHCl_3$) >99.9 % ee

(found (ESI) [M+Na]+, 329.0818. $C_{16}H_{18}NaO_4S$ requires 329.0818)

ν_{max}: 3500 (broad), 2971, 2930, 1447, 1302, 1139, 1080, 748, 687, 526 cm^{-1}.

^1H NMR (400 MHz, $CDCl_3$) δ 8.03 – 7.89 (m, 2H), 7.79 – 7.67 (m, 1H), 7.67 – 7.54 (m, 2H), 7.37 – 7.21 (m, 2H), 7.04 – 6.93 (m, 1H), 6.91 – 6.78 (m, 2H), 4.67 (s, 1H), 4.11 (t, *J* = 7.3 Hz, 1H), 3.93 (t, *J* = 8.2 Hz, 1H), 3.47 (q, *J* = 6.8 Hz, 1H), 3.08 (s, 1H), 1.43 (d, *J* = 7.0 Hz, 3H).

^{13}C NMR (101 MHz, $CDCl_3$) δ 157.91, 137.33, 134.10, 129.58, 129.37, 128.76, 121.51, 114.53, 67.94, 66.47, 60.81, 6.78.

Enantiomeric excess and diastereomeric ratio determined by HPLC analysis (CHIRALPAK IC column, hexane 90:10 iPrOH, 1.0 mL/min, T = 25°C, λ = 210 nm, Ketone 35.1 and 42.0 min, t_R = 44.0 min (major, d1), 48.2 min (minor, d1), 59.9 min (minor, d2). 99.3:0.7 dr, >99.9% ee (d1).

m/z (ESI) 329.2 ([M + Na]+, 100 %).

1-Phenoxy-3-(phenylsulfonyl)butan-2-one 19b

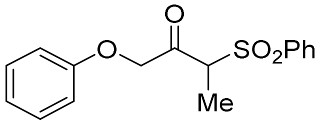

This compound is novel.

This compound was prepared following procedure F using: 1-phenoxy-3-(phenylsulfonyl)butan-2-ol (700 mg, 2.28 mmol, 1.0 equiv), PCC (983 mg, 4.57 mmol, 2.0 equiv) and DCM (30 mL). 1-phenoxy-3-(phenylsulfonyl)butan-2-one was isolated by flash chromatography (pet ether/ EtOAc: 80:20) as a white solid (340 mg, 1.12 mmol, 48.9%).

mp 96-97 °C.

(found (ESI) [M+Na]+, 327.0659. $C_{16}H_{16}NaO_4S$ requires 327.0655)
ν_{max}: 3057, 1734, 1599, 1495, 1226, 988, 740, 684, 580, 511 cm^{-1}.
^{1}H NMR (400 MHz, $CDCl_3$) δ 7.91 – 7.79 (m, 2H), 7.76 – 7.66 (m, 1H), 7.64 – 7.54 (m, 2H), 7.40 – 7.30 (m, 2H), 7.05 (t, *J* = 7.1 Hz, 1H), 6.94 (d, *J* = 7.5 Hz, 2H), 4.96 (d, *J* = 17.1 Hz, 1H), 4.80 (d, *J* = 17.1 Hz, 1H), 4.71 – 4.59 (m, 1H), 1.43 (d, *J* = 6.8 Hz, 3H).
^{13}C NMR (101 MHz, $CDCl_3$) δ 198.99, 157.41, 135.95, 134.49, 129.76, 129.52, 129.20, 122.12, 114.72, 73.10, 65.68, 11.82.
m/z (ESI) 327.2 ([M + Na]+, 100 %).

Racemic and (*1R,2S*)-3-phenoxy-1-phenyl-1-(phenylsulfonyl)propan-2-ol 20c

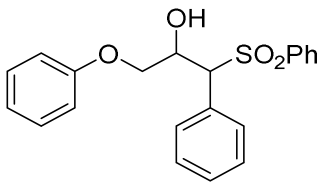

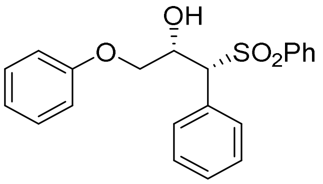

This compound is novel.
To a solution of benzyl phenyl sulphone (858 mg, 3.69 mmol, 1.0 equiv) in dry THF (15 mL) was added *n*BuLi (2.2 M in *n*-hexane, 4.4 mL, 4.0 mmol, 1.2 equiv) dropwise at –78 ºC under nitrogen atmosphere. After the reaction mixture had been stirred at –78 ºC for 30 minutes, 2-phenoxyacetaldehyde (500 mg, 3.69 mmol, 1.0 equiv) was added dropwise at -78 ºC. Upon stirring at same temperature for 1 h, the reaction mixture was stirred at ambient temperature for 1 h. It was then concentrated under reduced pressure, extracted with ethyl acetate (2 x 20 mL), washed with brine (50 mL), dried over Na_2SO_4, filtered, concentrated, and purified by column chromatography on silica gel (pet ether/ EtOAc: 80:20) to yield 3-phenoxy-1-phenyl-1-(phenylsulfonyl)propan-2-ol as white solid (501 mg, 1.36 mmol, 36.8%).
This compound was prepared in enantiomerically-enriched form following procedure D, using 3-phenoxy-1-phenyl-1-(phenylsulfonyl)propan-2-one (183 mg, 0.500 mmol, 1.0 equiv), FA/TEA (0.5 mL), [(*R,R*)Teth-TsDpenRuCl] (3.1 mg, 5.0 x 10^{-3} mmol, 1 mol%) and DCM (3 mL). (*S)*- 3-phenoxy-1-phenyl-1-(phenylsulfonyl)propan-2-ol was isolated by flash chromatography (pet ether/ EtOAc: 80:20) as a white solid (167 mg, 0.454 mmol, 90.8%).
mp 124-125 ºC.
$[\alpha]_D^{25}$ -86.0º (c 0.1 in $CHCl_3$) 99.2 % ee.
(found (ESI) [M+Na]+, 391.0979. $C_{21}H_{20}NaO_4S$ requires 391.0975)
ν_{max}: 3361 (broad), 2970, 2833, 1587, 1468, 1307, 1145, 950, 683, 589, 515 cm^{-1}.

^{1}H NMR (400 MHz, $CDCl_3$) δ 7.53 – 7.42 (m, 3H), 7.35 – 7.26 (m, 2H), 7.21 – 6.98 (m, 7H), 6.81 (t, *J* = 7.3 Hz, 1H), 6.63 (d, *J* = 7.9 Hz, 2H), 5.04 – 4.92 (m, 1H), 4.52 (d, *J* = 9.6 Hz, 1H), 4.09 (d, *J* = 2.2 Hz, 1H), 4.00 – 3.91 (m, 1H), 3.64 – 3.52 (m, 1H).

^{13}C NMR (101 MHz, $CDCl_3$) δ 158.28, 137.61, 133.86, 130.58, 130.02, 129.33, 129.13, 128.94, 128.72, 128.68, 121.26, 114.71, 73.51, 69.88, 69.49.

Enantiomeric excess and diastereomeric ratio determined by HPLC analysis (CHIRALPAK IC column, hexane 80:20 iPrOH, 1.0 mL/min, T = 25°C, λ = 210 nm, Ketone 24.1 and 27.9 min, t_R = 17.2 min (minor, d1), 19.6 min (major, d1), 49.6 min (minor, d2), 55.3 min (minor, d2). 93:7 dr, 99.2% ee (d1).

m/z (ESI) 391.2 ([M + Na]+, 100 %).

3-Phenoxy-1-phenyl-1-(phenylsulfonyl)propan-2-one 19c

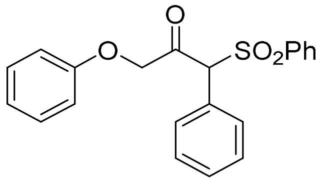

This compound is Novel.

This compound was prepared following procedure F using: 3-phenoxy-1-phenyl-1-(phenylsulfonyl)propan-2-ol (500 mg, 1.36 mmol, 1 equiv), PCC (584 mg, 2.71 mmol, 2.0 equiv) and DCM (20 mL). 3-phenoxy-1-phenyl-1-(phenylsulfonyl)propan-2-one was isolated by flash chromatography (pet ether/ EtOAc: 80:20) as a white solid (278 mg, 0.759 mmol, 55.9%).

mp 147-148 °C.

(found (ESI) [M+Na]+, 389.0819. $C_{21}H_{18}NaO_4S$ requires 389.0818)

ν_{max}: 2922, 2853, 1729, 1596, 1492 1250, 1116, 1062, 975, 752, 728, 685 cm^{-1}.

^{1}H NMR (400 MHz, $CDCl_3$) δ 7.63 – 7.44 (m, 3H), 7.36 – 7.11 (m, 9H), 6.98 – 6.83 (m, 1H), 6.79 – 6.66 (m, 2H), 5.60 (s, 1H), 4.65 (s, 2H).

^{13}C NMR (101 MHz, $CDCl_3$) δ 197.46, 157.20, 136.34, 134.22, 130.51, 129.96, 129.75, 129.72, 128.74, 128.62, 127.17, 122.12, 114.59, 75.27, 73.01.

m/z (ESI) 389.2 ([M + Na]+, 100 %).

Racemic and (1R,2S)-3-phenoxy-1-(phenylsulfonyl)-1-(p-tolyloxy)propan-2-ol 20d

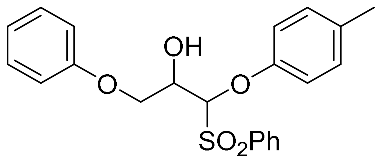 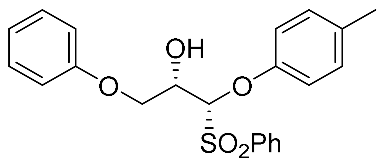

This compound is novel.

To a solution of 1-methyl-4-((phenylsulfonyl)methoxy)benzene (700 mg, 2.67 mmol, 1.0 equiv) in dry THF (25 mL) was added *n*BuLi (2.5 M in *n*-hexane, 1.75 mL, 3.47 mmol, 1.3 equiv) dropwise at –78 ºC under nitrogen atmosphere. After the reaction mixture had been stirred at –78 ºC for 30 minutes, 2-phenoxyacetaldehyde (472 mg, 3.47 mmol, 1.3 equiv) was added dropwise at -78 ºC. Upon stirring at same temperature for 1 h, the reaction mixture was stirred at ambient temperature for 1 h. It was then concentrated under reduced pressure, extracted with ethyl acetate (2 x 40 mL), washed with brine (80 mL), dried over Na_2SO_4, filtered, concentrated, and purified by column chromatography on silica gel (pet ether/ EtOAc: 80:20) to yield 3-phenoxy-1-(phenylsulfonyl)-1-(p-tolyloxy)propan-2-ol as colourless oil (758 mg, 1.90 mmol, 71.3%).

This compound was prepared in enantiomerically-enriched form following procedure D, using 3-phenoxy-1-(phenylsulfonyl)-1-(p-tolyloxy)propan-2-one (116 mg, 0.293 mmol, 1.0 equiv), FA/TEA (0.30 mL), [(*R,R*)Teth-TsDpenRuCl] (1.7 mg, 2.7 x 10^{-3} mmol, 1 mol%) and DCM (2 mL). (*1S,2S*)- 3-phenoxy-1-(phenylsulfonyl)-1-(p-tolyloxy)propan-2-ol was isolated by flash chromatography (pet ether/ EtOAc: 80:20) as a colourless oil (81 mg, 0.20 mmol, 69%).

$[\alpha]_D^{25}$ +73.0º (c 0.1 in $CHCl_3$) >99.9% ee.

(found (ESI) [M+Na]+, 421.1079. $C_{22}H_{22}NaO_5S$ requires 421.1080)

ν_{max}: 3485 (broad), 3064, 2925, 1598, 1506, 1305, 1213, 1145, 1058, 750, 686, 578 cm^{-1}.

^{1}H NMR (400 MHz, $CDCl_3$) δ 7.99 – 7.86 (m, 2H), 7.66 – 7.56 (m, 1H), 7.53 – 7.43 (m, 2H), 7.31 – 7.21 (m, 2H), 6.99 – 6.82 (m, 5H), 6.77 – 6.68 (m, 2H), 5.48 (d, *J* = 1.8 Hz, 1H), 4.89 (dd, *J* = 11.5, 5.6 Hz, 1H), 4.25 – 4.16 (m, 1H), 4.09 – 3.98 (m, 1H), 2.91 (d, *J* = 6.2 Hz, 1H), 2.19 (s, 3H).

^{13}C NMR (101 MHz, $CDCl_3$) δ 157.81, 155.54, 136.91, 134.36, 132.88, 130.06, 129.76, 129.59, 129.05, 121.62, 116.09, 114.70, 92.31, 68.10, 67.11, 20.47.

Enantiomeric excess and diastereomeric ratio determined by HPLC analysis (CHIRALPAK IG column, hexane 90:10 iPrOH, 1.0 mL/min, T = 25°C, λ = 210 nm, Ketone 64.6 and 78.7 min, t_R = 59.3 min (major, d1), 71.0 min (minor, d1), 79.2 min (major, d2), 87.7 min (minor, d2). 96.0:4.0 dr, >99.9% ee (d1).

m/z (ESI) 421.2 ([M + Na]+, 100 %).

3-Phenoxy-1-(phenylsulfonyl)-1-(p-tolyloxy)propan-2-one 19d

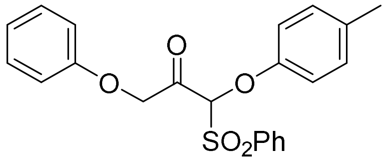

This compound is Novel.

To a flask containing the 3-phenoxy-1-(phenylsulfonyl)-1-(p-tolyloxy)propan-2-ol (300 mg, 0.754 mmol, 1 equiv) in DCM was added DMP (450 mg, 1.13 mmol, 1.5 equiv) and stirred at rt for 48 hours. After completion of the reaction, the reaction mixture is washed with sodium thiosulfate (2 x 20 mL), followed by $NaHCO_3$ (30 mL) and brine (30 mL) dried over Na_2SO_4, filtered, concentrated, and purified by column chromatography on silica gel (pet ether/ EtOAc: 85:15) to yield 3-phenoxy-1-(phenylsulfonyl)-1-(p-tolyloxy)propan-2-one as white solid (213 mg, 0.535 mmol, 71.3%).

mp 89-90 ºC.

(found (ESI) [M+Na]+, 419.0928. $C_{22}H_{20}NaO_5S$ requires 419.0924)

ν_{max}: 2930, 1742, 1493, 1315, 1214, 1148, 1062, 742, 686, 583, 513 cm^{-1}.

^{1}H NMR (400 MHz, $CDCl_3$) δ 7.86 (d, J = 7.6 Hz, 2H), 7.62 (t, J = 7.4 Hz, 1H), 7.49 (t, J = 7.7 Hz, 2H), 7.20 (dd, J = 13.9, 5.9 Hz, 2H), 6.94 (dd, J = 17.4, 7.9 Hz, 3H), 6.82 (t, J = 10.6 Hz, 2H), 6.67 (d, J = 8.5 Hz, 2H), 5.69 (s, 1H), 4.98 (d, J = 11.7 Hz, 2H), 2.19 (s, 3H).

^{13}C NMR (101 MHz, $CDCl_3$) δ 196.03, 157.45, 154.67, 135.60, 135.02, 133.76, 130.42, 129.90, 129.68, 129.34, 122.07, 116.09, 114.87, 94.63, 71.85, 20.56.

m/z (ESI) 419.2 ([M + Na]+, 100 %).

Racemic and (*1R,2S*)-1-(Benzyloxy)-3-phenoxy-1-(phenylsulfonyl)propan-2-ol 20e

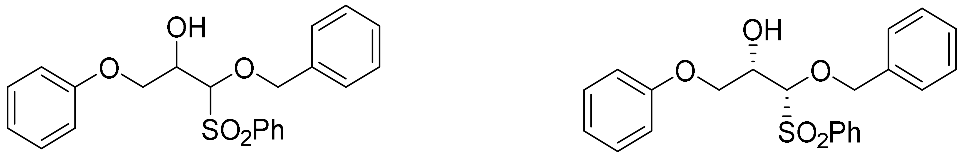

This compound is novel.

To a solution of (((benzyloxy)methyl)sulfonyl)benzene (430 mg, 1.64 mmol, 1.0 equiv) in dry THF (15 mL) was added *n*BuLi (2.5 M in *n*-hexane, 1.0 mL, 1.96 mmol, 1.2 equiv) dropwise at –78 ºC under nitrogen atmosphere. After the reaction mixture had been stirred at –78 ºC for 30 minutes, 2-phenoxyacetaldehyde (268 mg, 1.96 mmol, 1.2 equiv) was added dropwise at -78 ºC. Upon stirring at same temperature for 1 h, the reaction mixture was stirred at ambient

temperature for 1 h. It was then concentrated under reduced pressure, extracted with ethyl acetate (2 x 30 mL), washed with brine (40 mL), dried over Na_2SO_4, filtered, concentrated, and purified by column chromatography on silica gel (pet ether/ EtOAc: 80:20) to yield **1-**(benzyloxy)-3-phenoxy-1-(phenylsulfonyl)propan-2-ol as white solid (301 mg, 0.756 mmol, 46.1%).

This compound was prepared in enantiomerically-enriched form following procedure D, using 1-(benzyloxy)-3-phenoxy-1-(phenylsulfonyl)propan-2-one (100 mg, 0.252 mmol, 1.0 equiv), FA/TEA (0.25 mL), [(*R,R*)Teth-TsDpenRuCl] (1.6 mg, 2.58 x 10^{-3} mmol, 1 mol%) and DCM (2 mL). (*1R,2S*)-1-(benzyloxy)-3-phenoxy-1-(phenylsulfonyl)propan-2-ol was isolated by flash chromatography (pet ether/ EtOAc: 80:20) as a white solid (70 mg, 0.17 mmol, 70%).

mp 82-83 °C.

$[\alpha]_D^{25}$ +67.0° (c 0.1 in $CHCl_3$) 99.6 % ee.

(found (ESI) [M+Na]+, 421.1078. $C_{22}H_{22}NaO_5S$ requires 421.1080)

ν_{max}: 3462 (broad), 3060, 2938, 1602, 1493, 1289, 1041, 735, 685, 516 cm^{-1}.

^1H NMR (400 MHz, $CDCl_3$) δ 8.03 – 7.97 (m, 2H), 7.73 – 7.65 (m, 1H), 7.62 – 7.53 (m, 2H), 7.27 – 7.21 (m, 7H), 6.96 (t, *J* = 7.4 Hz, 1H), 6.73 (d, *J* = 7.9 Hz, 2H), 4.93 (d, *J* = 11.1 Hz, 1H), 4.75 (d, *J* = 1.7 Hz, 1H), 4.72 (d, *J* = 11.2 Hz, 1H), 4.58 – 4.49 (m, 1H), 3.92 (dd, *J* = 9.2, 5.3 Hz, 1H), 3.68 (dd, *J* = 9.1, 7.5 Hz, 1H).

^{13}C NMR (101 MHz, $CDCl_3$) δ 157.80, 137.13, 135.15, 134.29, 133.36, 129.83, 129.46, 129.08, 128.82, 128.66, 121.38, 114.45, 92.86, 76.04, 67.86, 67.25.

Enantiomeric excess and diastereomeric ratio determined by HPLC analysis (CHIRALPAK IG column, hexane 80:20 iPrOH, 1.0 mL/min, T = 25°C, λ = 210 nm, Ketone 36.3 and 42.1 min, t_R = 20.2 min (minor, d1), 33.6 min (major, d1), 39.0 min (minor, d2), 62.1 min (major, d2). 99.7:0.3 dr, 99.6% ee (d2).

m/z (ESI) 421.2 ([M + Na]+, 100 %).

1-(Benzyloxy)-3-phenoxy-1-(phenylsulfonyl)propan-2-one 19e

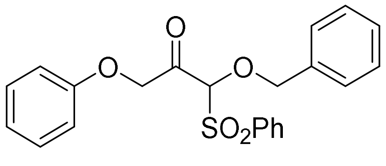

This compound is Novel.

To a flask containing the 1-(benzyloxy)-3-phenoxy-1-(phenylsulfonyl)propan-2-ol (300 mg, 0.754 mmol, 1.0 equiv) in DCM (8 mL) was added DMP (479 mg, 1.13 mmol, 1.5 equiv) and

stirred at rt for 48 hours. After completion of the reaction, the reaction mixture is washed with sodium thiosulfate (2 x 20 mL), followed by $NaHCO_3$ (30 mL) and brine (30 mL) dried over Na_2SO_4, filtered, concentrated, and purified by column chromatography on silica gel (pet ether/ EtOAc: 85:15) to yield 1-(benzyloxy)-3-phenoxy-1-(phenylsulfonyl)propan-2-one as white solid (240 mg, 0.606 mmol, 80.4%).

mp 120-121 °C.

(found (ESI) [M+Na]+, 419.0921. $C_{22}H_{20}NaO_5S$ requires 419.0924)

ν_{max}: 3059, 2909, 1735, 1596, 1307, 1219, 1137, 1069, 750, 586 cm^{-1}.

^{1}H NMR (400 MHz, $CDCl_3$) δ 7.84 – 7.75 (m, 2H), 7.66 – 7.58 (m, 1H), 7.54 – 7.44 (m, 2H), 7.32 – 7.12 (m, 7H), 6.91 (t, *J* = 7.4 Hz, 1H), 6.70 (d, *J* = 7.9 Hz, 2H), 5.05 (s, 1H), 4.96 (d, *J* = 11.7 Hz, 1H), 4.85 – 4.73 (m, 2H), 4.71 – 4.61 (m, 1H).

^{13}C NMR (101 MHz, $CDCl_3$) δ 196.31, 157.45, 135.61, 134.80, 134.72, 129.83, 129.59, 129.29, 129.11, 128.96, 128.80, 121.91, 114.74, 94.58, 75.60, 71.54.

m/z (ESI) 419.2 ([M + Na]+, 100 %).

Racemic and (*1R,2S*)-1-methoxy-3-phenoxy-1-(phenylsulfonyl)propan-2-ol 20f

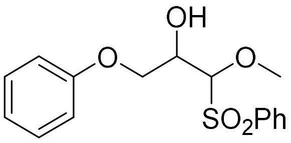

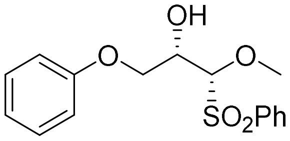

This compound is novel.

To a solution of ((methoxymethyl)sulfonyl)benzene (559 mg, 3.01 mmol, 1.0 equiv) in dry THF (20 mL) was added *n*BuLi (2.5 M in *n*-hexane, 1.76 mL, 3.30 mmol, 1.1 equiv) dropwise at –78 °C under nitrogen atmosphere. After the reaction mixture had been stirred at –78 °C for 30 minutes, 2-phenoxyacetaldehyde (449 mg, 3.30 mmol, 1.1 equiv) was added dropwise at -78 °C. Upon stirring at same temperature for 1 h, the reaction mixture was stirred at ambient temperature for 1 h. It was then concentrated under reduced pressure, extracted with ethyl acetate (2 x 30 mL), washed with brine (40 mL), dried over Na_2SO_4, filtered, concentrated, and purified by column chromatography on silica gel (pet ether/ EtOAc: 80:20) to yield 1-methoxy-3-phenoxy-1-(phenylsulfonyl)propan-2-ol as colourless oil (472 mg, 1.46 mmol, 48.9%).

This compound was prepared in enantiomerically-enriched form following procedure D, using 1-methoxy-3-phenoxy-1-(phenylsulfonyl)propan-2-one (120 mg, 0.373 mmol, 1.0 equiv), FA/TEA (0.35 mL), [(*R,R*)Teth-TsDpenRuCl] (2.3 mg, 3.70 x 10^{-3} mmol, 1 mol%) and DCM (3 mL). (1R,2S)-1-methoxy-3-phenoxy-1-(phenylsulfonyl)propan-2-ol was isolated by flash chromatography (pet ether/ EtOAc: 80:20) as a colourless oil (97 mg, 0.30 mmol, 80%).

$[\alpha]_D^{25}$ +37.0° (c 0.1 in $CHCl_3$) 94.4 % ee.

(found (ESI) [M+Na]+, 345.0765. $C_{16}H_{18}NaO_5S$ requires 345.0767)

ν_{max}: 3417 (broad), 2925, 2822, 1598, 1495, 1241, 1040, 751, 690, 508 cm^{-1}.

^1H NMR (400 MHz, $CDCl_3$) δ 8.10 – 7.96 (m, 2H), 7.81 – 7.70 (m, 1H), 7.68 – 7.56 (m, 2H), 7.34 – 7.27 (m, 2H), 7.06 – 6.88 (m, 3H), 4.58 (d, J = 8.3 Hz, 1H), 4.21 – 4.08 (m, 2H), 4.07 – 3.97 (m, 1H), 3.56 (s, 3H), 3.48 (d, J = 3.1 Hz, 1H).

^{13}C NMR (101 MHz, $CDCl_3$) δ 158.31, 135.74, 134.46, 129.75, 129.57, 129.22, 121.42, 114.65, 96.55, 69.24, 67.83, 62.12.

Enantiomeric excess and diastereomeric ratio determined by HPLC analysis (CHIRALPAK IG column, hexane 80:20 iPrOH, 1.0 mL/min, T = 25°C, λ = 210 nm, Ketone 12.0 and 30.1 min, t_R = 22.6 min (major, d1), 33.6 min (minor, d1). >99:1 dr, 94.4% ee (d1).

m/z (ESI) 345.1 ([M + Na]+, 100 %).

1-Methoxy-3-phenoxy-1-(phenylsulfonyl)propan-2-one 19f

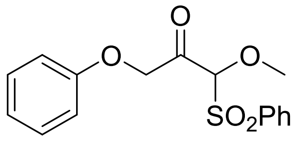

This compound is Novel.

To a flask containing the 1-methoxy-3-phenoxy-1-(phenylsulfonyl)propan-2-ol (200 mg, 0.625 mmol, 1 equiv) in DCM (8 mL) was added DMP (395 mg, 0.930 mmol, 1.5 equiv) and stirred at rt for 48 hours. After completion of the reaction, the reaction mixture is washed with sodium thiosulfate (2 x 20 mL), followed by $NaHCO_3$ (30 mL) and brine (30 mL) dried over Na_2SO_4, filtered, concentrated, and purified by column chromatography on silica gel (pet ether/ EtOAc: 85:15) to yield 1-methoxy-3-phenoxy-1-(phenylsulfonyl)propan-2-one as white solid (136 mg, 0.422 mmol, 68.4%).

mp 79-80 °C.

(found (ESI) [M+Na]+, 343.0606. $C_{16}H_{16}NaO_5S$ requires 343.0611)

ν_{max}: 3064, 2892, 1729, 1586, 1496, 1234, 1002, 840, 747, 687 cm^{-1}.

^1H NMR (400 MHz, $CDCl_3$) δ 7.98 – 7.91 (m, 1H), 7.90 – 7.82 (m, 1H), 7.68 – 7.60 (m, 2H), 7.54 – 7.44 (m, 2H), 7.26 – 7.21 (m, 1H), 6.93 (t, J = 7.4 Hz, 1H), 6.80 (d, J = 8.0 Hz, 2H), 4.94 (s, 1H), 4.87 (d, J = 18.0 Hz, 1H), 4.73 (d, J = 18.0 Hz, 1H), 3.67 (s, 3H).

^{13}C NMR (101 MHz, $CDCl_3$) δ 196.38, 154.73, 135.15, 134.84, 129.76, 129.64, 129.29, 122.01, 114.86, 98.26, 71.52, 62.10.

m/z (ESI) 343.1 ([M + Na]+, 100 %).

Racemic and (*1R,2S*)-1-(4-methoxyphenoxy)-3-phenoxy-1-(phenylsulfonyl)propan-2-ol 20g

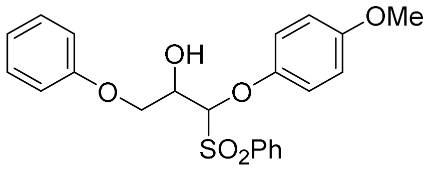

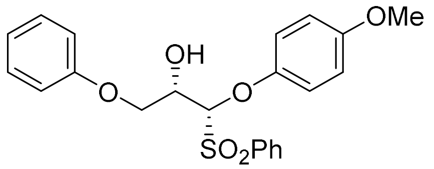

This compound is novel.

To a solution of 1-methoxy-4-((phenylsulfonyl)methoxy)benzene (700 mg, 2.50 mmol, 1.0 equiv) in dry THF (25 mL) was added *n*BuLi (2.5 M in *n*-hexane, 1.50 mL, 3.0 mmol, 1.2 equiv) dropwise at –78 ºC under nitrogen atmosphere. After the reaction mixture had been stirred at –78 ºC for 30 minutes, 2-phenoxyacetaldehyde (409 mg, 3.01 mmol, 1.2 equiv) was added dropwise at -78 ºC. Upon stirring at same temperature for 1 h, the reaction mixture was stirred at ambient temperature for 1 h. It was then concentrated under reduced pressure, extracted with ethyl acetate (2 x 40 mL), washed with brine (80 mL), dried over Na_2SO_4, filtered, concentrated, and purified by column chromatography on silica gel (pet ether/ EtOAc: 80:20) to yield 1-(4-methoxyphenoxy)-3-phenoxy-1-(phenylsulfonyl)propan-2-ol as colourless oil (653 mg, 1.57 mmol, 62.8%).

This compound was prepared in enantiomerically-enriched form following procedure D, using 1-(4-methoxyphenoxy)-3-phenoxy-1-(phenylsulfonyl)propan-2-one (140 mg, 0.339 mmol, 1.0 equiv), FA/TEA (0.35 mL), [(*R,R*)Teth-TsDpenRuCl] (2.0 mg, 3.22×10^{-3} mmol, 1 mol%) and DCM (2 mL). (*1R,2S*)-1-(4-methoxyphenoxy)-3-phenoxy-1-(phenylsulfonyl)propan-2-ol was isolated by flash chromatography (pet ether/ EtOAc: 80:20) as a colourless oil (116 mg, 0.278 mmol, 82.4%).

$[\alpha]_D^{25}$ +66.0° (c 0.1 in $CHCl_3$) 99.2 % ee.

(found (ESI) [M+Na]+, 437.1030. $C_{22}H_{22}NaO_6S$ requires 437.1029)

ν_{max}: 3477 (broad), 3061, 2835, 1502, 1202, 1143, 1031, 825, 749, 685, 579, 507 cm^{-1}.

^1H NMR (400 MHz, CDCl3) δ dr 7.90 – 7.81 (m, 2H), 7.59 – 7.50 (m, 1H), 7.46 – 7.38 (m, 2H), 7.23 – 7.15 (m, 2H), 6.95 – 6.85 (m, 1H), 6.82 – 6.75 (m, 2H), 6.72 – 6.65 (m, 2H), 6.60 – 6.51 (m, 2H), 5.34 (d, *J* = 2.0 Hz, 1H), 4.85 – 4.77 (m, 1H), 4.19 – 4.10 (m, 1H), 4.05 – 3.95 (m, 1H), 3.61 (s, 3H).

^{13}C NMR (101 MHz, CDCl3) δ 157.81, 155.51, 151.70, 136.98, 134.39, 129.72, 129.62, 129.08, 121.65, 117.57, 114.70, 114.61, 93.14, 68.07, 67.11, 55.61.

Enantiomeric excess and diastereomeric ratio determined by HPLC analysis (CHIRALPAK IG column, hexane 80:20 iPrOH, 1.0 mL/min, T = 25°C, λ = 210 nm, Ketone 18.1 and 19.8 min, t_R = 40.0 min (major, d1), 46.0 min (minor, d1), 55.2 min (major, d2), 62.8 min (minor, d2). 96.7:3.3 dr, 99.2% ee (d1).
m/z (ESI) 437.2 ([M + Na]+, 100 %).

1-(4-Methoxyphenoxy)-3-phenoxy-1-(phenylsulfonyl)propan-2-one 19g

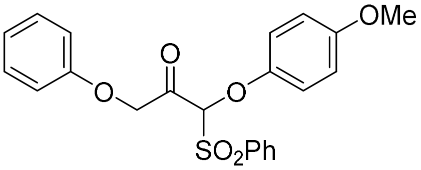

This compound is Novel.
To a flask containing the 1-(4-methoxyphenoxy)-3-phenoxy-1-(phenylsulfonyl)propan-2-ol (208 mg, 0.502 mmol, 1 equiv) in DCM (8 mL) was added DMP (318 mg, 0.75 mmol, 1.5 equiv) and stirred at rt for 48 hours. After completion of the reaction, the reaction mixture is washed with sodium thiosulfate (2 x 20 mL), followed by $NaHCO_3$ (30 mL) and brine (30 mL) dried over Na_2SO_4, filtered, concentrated, and purified by column chromatography on silica gel (pet ether/ EtOAc: 85:15) to yield 1-(4-methoxyphenoxy)-3-phenoxy-1-(phenylsulfonyl)propan-2-one as white solid (120 mg, 0.291 mmol, 58.0%).
mp 151-152 °C.
(found (ESI) [M+Na]+, 435.0876. $C_{22}H_{20}NaO_6S$ requires 435.0873)
ν_{max}: 2997, 2934, 1741, 1423, 1241, 1188, 1033, 830, 741, 586, 504 cm^{-1}.
^{1}H NMR (400 MHz, $CDCl_3$) δ 7.97 – 7.91 (m, 2H), 7.75 – 7.69 (m, 1H), 7.63 – 7.52 (m, 2H), 7.30 – 7.23 (m, 2H), 7.04 – 6.97 (m, 1H), 6.94 – 6.87 (m, 2H), 6.85 – 6.71 (m, 4H), 5.70 (s, 1H), 5.05 (d, J = 2.4 Hz, 2H), 3.74 (s, 3H).
^{13}C NMR (101 MHz, $CDCl_3$) δ 196.10, 157.43, 156.17, 150.86, 136.15, 135.58, 135.02, 133.23, 131.35, 129.90, 129.68, 129.34, 122.08, 117.80, 114.87, 95.47, 71.87, 55.67.
m/z (ESI) 435.2 ([M + Na]+, 100 %).

Racemic and (*S*)-1-Phenyl-3-(p-tolyl)propan-2-ol 21

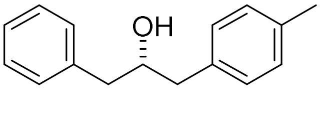

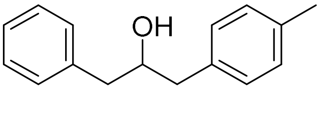

This compound is known and has been fully characterized.

This compound was prepared following procedure G using: (1*R*,2*S*)-3-phenyl-1-(phenylsulfonyl)-1-(p-tolyl)propan-2-ol **20a** (80 mg, 0.22 mmol, 1.0 equiv.), activated Mg turnings (158 mg, 6.50 mmol, 30 equiv.) and MeOH (6 mL). (*S*)-1-phenyl-3-(*p*-tolyl)propan-2-ol was isolated by flash chromatography (pet ether/ EtOAc: 92:08) as a colourless oil (31 mg, 0.14 mmol, 63%).

This compound was prepared in racemic form following procedure C: 1-phenyl-3-(p-tolyl)propan-2-one (113 mg, 0.504 mmol, 1.0 equiv), $NaBH_4$ (34 mg, 0.89 mmol, 1.80 equiv) and MeOH (5 mL). 1-phenyl-3-(p-tolyl)propan-2-ol was isolated by flash chromatography (pet ether/ EtOAc: 90:10) as a white solid (101 mg, 0.446 mmol, 90.2%).

$[\alpha]_D^{25}$ +22.0° (c 0.1 in $CHCl_3$) 99.4 % ee.

^{1}H NMR (400 MHz, $CDCl_3$) δ 7.38 – 7.32 (m, 2H), 7.30 – 7.24 (m, 3H), 7.16 (s, 4H), 4.13 – 4.02 (m, 1H), 2.95 – 2.68 (m, 4H), 2.36 (s, 3H), 1.66 (d, *J* = 3.3 Hz, 1H).

^{13}C NMR (101 MHz, $CDCl_3$) δ 138.55, 136.05, 135.28, 129.44, 129.31, 129.28, 128.55, 126.48, 73.64, 43.37, 42.96, 21.06.

Enantiomeric excess determined by HPLC analysis (CHIRALCEL OD-H column, hexane 98:02 iPrOH, 1.0 mL/min, T = 25°C, λ = 210 nm, *R* enantiomer 13.4 min, *S*-enantiomer 15.1 min). 99.4% ee (*S*).

m/z (ESI) 421.2 ([M + Na]+, 100 %).

For control experiment **(*S*)-1-phenyl-3-(p-tolyl)propan-2-ol also been prepared by ATH using general procedure D:** 1-phenyl-3-(p-tolyl)propan-2-one (112 mg, 0.50 mmol, 1.0 equiv), FA/TEA (0.5 mL), [(*R,R*)Teth-TsDpenRuCl] (3.1 mg, 5.0 x 10^{-3} mmol, 1 mol%) and DCM (3 mL). (*S*)-1-phenyl-3-(p-tolyl)propan-2-ol was isolated by flash chromatography (pet ether/ EtOAc: 90:10) as a colourless oil (108 mg, 0.478 mmol, 95.6%).

$[\alpha]_D^{25}$ 0 (c 0.1 in $CHCl_3$) 0 % ee.

Enantiomeric excess determined by HPLC analysis (CHIRALCEL OD-H column, hexane 98:02 iPrOH, 1.0 mL/min, T = 25°C, λ = 210 nm, *R* enantiomer 14.8 min, *S*-enantiomer 17.7 min). 0% ee.

Racemic and (*R*)-1-phenoxy-3-phenylpropan-2-ol 22

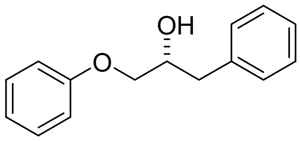

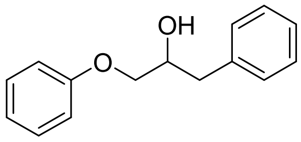

This compound is known and has been fully characterized.

This compound was prepared following procedure G using: (1*R*,2*S*)-3-phenoxy-1-phenyl-1-(phenylsulfonyl)propan-2-ol **20c** (315 mg, 0.856 mmol, 1.0 equiv.), activated Mg turnings (619 mg, 25.5 mmol, 30.0 equiv.) and MeOH (10 mL). (*R*)-1-phenoxy-3-phenylpropan-2-ol was isolated by flash chromatography (pet ether/ EtOAc: 90:10) as a white solid (161 mg, 0.706 mmol, 82.5%).

This compound was prepared in racemic form following this procedure: Phenyl glycidyl ether (2.01 g, 13.3 mmol, 1.0 equiv.) was dissolved in dry THF (40 mL) and cooled to 0 °C. Phenyl magnesium bromide (3 M in THF, 8.90 mL, 26.6 mmol, 2.0 equiv.) was added dropwise at the same 0 °C and allowed it to warm at rt and stirred for 1 hours. Reaction mixture was diluted with saturated aqueous NH_4Cl solution and extracted with ethyl acetate. The organic layers were dried ($MgSO_4$), filtered and concentrated in vacuo and purified by column chromatography on silica gel (pet ether/ EtOAc: 90:10) to yield 1-phenoxy-3-phenylpropan-2-ol as a white solid (1.43 g, 6.27 mmol, 47.0%).

mp 96-97 °C.

$[\alpha]_D^{25}$ -62.0° (c 0.1 in $CHCl_3$) 99 % ee

^{1}H NMR (400 MHz, $CDCl_3$) δ 7.32 – 7.21 (m, 7H), 7.02 – 6.84 (m, 3H), 4.29 – 4.19 (m, 1H), 3.97 (dd, *J* = 9.4, 3.6 Hz, 1H), 3.89 (dd, *J* = 9.3, 6.6 Hz, 1H), 2.94 (d, *J* = 6.7 Hz, 2H), 2.29 (d, *J* = 3.7 Hz, 1H).

^{13}C NMR (101 MHz, $CDCl_3$) δ 158.54, 137.55, 129.54, 129.40, 128.65, 126.67, 121.19, 114.62, 71.12, 70.95, 39.89.

Enantiomeric excess determined by HPLC analysis (CHIRALPAK IA column, hexane 93:07 iPrOH, 0.7 mL/min, T = 25°C, λ = 210 nm, *R* enantiomer 10.5 min, *S*-enantiomer 16.6 min. 99.0% ee (*R*).

m/z (ESI) 421.2 ([M + Na]+, 100 %).

For control experiment **(*R*)-1-phenoxy-3-phenylpropan-2-ol also been prepared by ATH using general procedure D:** 1-phenoxy-3-phenylpropan-2-one (113 mg, 0.50 mmol, 1.0 equiv), FA/TEA (0.5 mL), [(*R,R*)Teth-TsDpenRuCl] (3.1 mg, 5.0 x 10^{-3} mmol, 1 mol%) and DCM (3 mL). (*R*)-1-phenoxy-3-phenylpropan-2-ol was isolated by flash chromatography (pet ether/ EtOAc: 90:10) as a white solid (102 mg, 0.451 mmol, 89.5%).

Enantiomeric excess determined by HPLC analysis (CHIRALPAK IA column, hexane 93:07 iPrOH, 0.7 mL/min, T = 25°C, λ = 210 nm, *R* enantiomer 11.0 min, *S*-enantiomer 18.8 min). 64.2% ee (*R*).

Racemic and (*S*)-1-phenoxy-3-(p-tolyloxy)propan-2-ol 23

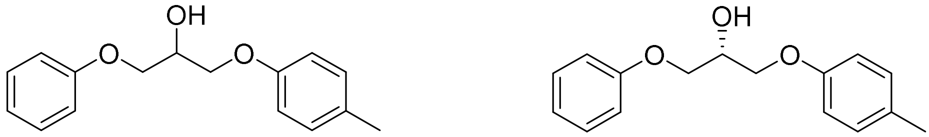

This compound is known and has been fully characterized.

This compound was prepared following procedure G using: (1*S*,2*S*)-3-phenoxy-1-(phenylsulfonyl)-1-(p-tolyloxy)propan-2-ol **20d** (80 mg, 0.20 mmol, 1 equiv.), activated Mg turnings (144 mg, 5.92 mmol, 30 equiv.) and MeOH (6 mL). (S)-1-phenoxy-3-(p-tolyloxy)propan-2-ol was isolated by flash chromatography (pet ether/ EtOAc: 90:10) as a white solid (31 mg, 0.12 mmol, 60%).

This compound was prepared in racemic form following this procedure: To a solution of $PdCl_2$ (10 mg, 0.06 mmol, 0.01 eq), TBAI (590 mg, 1.60 mmol, 0.25 eq) and K_2CO_3 (220 mg, 1.6 mmol, 0.25 eq) in deionized H_2O (50 mL) at 60 °C phenyl glycidyl ether (1.01 g, 6.68 mmol, 1 eq) and p-cresol (649 mg, 6.01 mmol, 0.9 eq) were added. The reaction mixture was then stirred for 3.5 h at 60 °C and a further 12 hrs at rt. EtOAc (3 x 30 mL) was added to extract the product from the aqueous solution. The organic layers were separated, combined, and dried over Na_2SO_4, before being concentrated under vacuum. The crude product was then purified by flash chromatography (pet ether/ EtOAc: 90:10) as a white solid (910 mg, 3.53 mmol, 58.7%).

mp 77-78 °C.

$[\alpha]_D^{25}$ +21.0° (c 0.1 in $CHCl_3$) 99.8 % ee.

ν_{max}: 3514 (broad), 2926, 1596, 1511, 1228, 1117, 902, 815, 751, 692, 510 cm^{-1}.

^{1}H NMR (400 MHz, $CDCl_3$) δ 7.36 – 7.25 (m, 2H), 7.14 – 7.03 (m, 2H), 7.01 – 6.88 (m, 3H), 6.87 – 6.79 (m, 2H), 4.37 (d, *J* = 3.5 Hz, 1H), 4.19 – 4.06 (m, 4H), 2.64 (d, *J* = 3.2 Hz, 1H), 2.28 (s, 3H).

^{13}C NMR (101 MHz, $CDCl_3$) δ 158.47, 156.37, 130.57, 130.00, 129.57, 121.27, 114.60, 114.47, 68.90, 68.88, 68.72, 20.49.

Enantiomeric excess determined by HPLC analysis (CHIRALPAK IG column, hexane 90:10 iPrOH, 0.7 mL/min, T = 25°C, λ = 210 nm, *R* enantiomer 20.8 min, *S*-enantiomer 22.2 min). 99.8% ee (*S*).

m/z (ESI) 421.2 ([M + Na]+, 100 %).

For control experiment **(*S*)-1-phenoxy-3-(p-tolyloxy)propan-2-ol also been prepared by ATH using general procedure D:** 1-phenoxy-3-(p-tolyloxy)propan-2-one (128 mg, 0.50 mmol, 1.0 equiv), FA/TEA (0.5 mL), [(*R,R*)Teth-TsDpenRuCl] (3.1 mg, 5.0 x 10^{-3} mmol, 1

mol%) and DCM (3 mL). (*S*)-1-phenoxy-3-(p-tolyloxy)propan-2-ol was isolated by flash chromatography (pet ether/ EtOAc: 90:10) as a white solid (111 mg, 0.430 mmol, 86.0%).

$[\alpha]_D^{25}$ +3.0° (c 0.1 in $CHCl_3$) 4.6 % ee.

Enantiomeric excess determined by HPLC analysis (CHIRALPAK IG column, hexane 90:10 iPrOH, 0.7 mL/min, T = 25°C, λ = 210 nm, *R* enantiomer 20.7 min, *S*-enantiomer 23.0 min). 4.6% ee (*R*).

Racemic and (*R*)-1-methoxy-3-phenoxypropan-2-ol 24

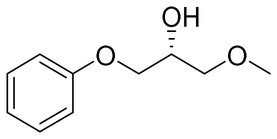

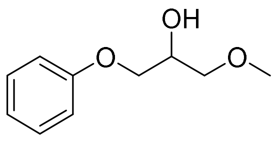

This compound is known and has been fully characterized.

This compound was prepared following procedure G using: (1*S*,2*S*)-1-methoxy-3-phenoxy-1-(phenylsulfonyl)propan-2-ol **20f** (70 mg, 0.22 mmol, 1.0 equiv.), activated Mg turnings (158 mg, 6.50 mmol, 30 equiv.) and MeOH (8 mL). (*R*)-1-methoxy-3-phenoxypropan-2-ol was isolated by flash chromatography (pet ether/ EtOAc: 80:20) as a colourless oil (28 mg, 0.15 mmol, 71%).

This compound was prepared in racemic form following this procedure: To methanol (20 mL), sodium hydride (400 mg, 10 mmol, 1.5 eq) was added and the mixture was stirred at room temperature for 30 minutes to allow deprotonation. Phenyl glycidyl ether (1.0 g, 6.7 mmol, 1 eq) was then added and allowed to react for at least 2 hours at room temperature. The reaction was quenched with ammonium chloride (10 mL). Ethyl acetate (20 mL) and water (10 mL) were added to allow clear separation into two layers. The organic layer was extracted using ethyl acetate (3 x 10 mL) and water (10 mL) and was dried using sodium sulfate, filtered and concentrated in vacuo to give the 1-methoxy-3-phenoxypropan-2-ol as a colourless oil (946 mg, 5.19 mmol, 77.9%).

$[\alpha]_D^{25}$ +31.0° (c 0.1 in $CHCl_3$) >99.9 % ee.

^{1}H NMR (400 MHz, $CDCl_3$) δ 7.26 – 7.16 (m, 2H), 6.92 – 6.80 (m, 3H), 4.16 – 4.04 (m, 1H), 3.99 – 3.89 (m, 2H), 3.55 – 3.43 (m, 2H), 3.35 (s, 3H), 2.51 (d, *J* = 4.7 Hz, 1H).

^{13}C NMR (101 MHz, $CDCl_3$) δ 158.56, 129.52, 121.13, 114.57, 73.49, 69.06, 68.88, 59.30.

Enantiomeric excess determined by HPLC analysis (CHIRALPAK IA column, hexane 90:10 iPrOH, 1.0 mL/min, T = 25°C, λ = 210 nm, *R* enantiomer 10.9 min, *S*-enantiomer 8.6 min). >99.9% ee (*R*).

m/z (ESI) 205.1 ([M + Na]+, 100 %).

For control experiment, this compound was already been reported via ATH in the following reference and was reduced in 30% ee.

Forshaw, S.; Matthews, A. J.; Brown, T. J.; Diorazio, L. J.; Williams L, Wills M. *Org. Lett*, **2017**, *19*, 2789-2792.

Racemic and (*S*)-1-(4-methoxyphenoxy)-3-phenoxypropan-2-ol 25

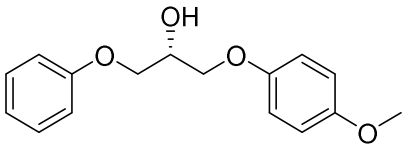

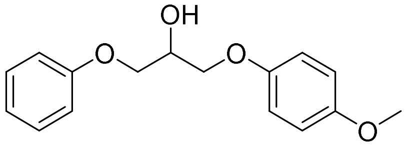

This compound is known and has been fully characterized.

This compound was prepared following procedure G using: (1*S*,2*S*)-1-(4-methoxyphenoxy)-3-phenoxy-1-(phenylsulfonyl)propan-2-ol **20g** (100 mg, 0.241 mmol, 1 equiv.), activated Mg turnings (176 mg, 7.24 mmol, 30 equiv.) and MeOH (8 mL). (*S*)-1-(4-methoxyphenoxy)-3-phenoxypropan-2-ol was isolated by flash chromatography (pet ether/ EtOAc: 85:15) as a white solid (36 mg, 0.13 mmol, 54%).

This compound was prepared in racemic form following this procedure: To a solution of $PdCl_2$ (5 mg, 0.03 mmol, 0.01 eq), TBAI (300 mg, 0.80 mmol, 0.25 eq) and K_2CO_3 (110 mg, 0.8 mmol, 0.25 eq) in deionized H_2O (25 mL) at 60 ºC phenyl glycidyl ether (500 mg, 3.34 mmol, 1 eq) and 4-methoxyphenol (455mg, 3.67 mmol, 1.1 eq) were added. The reaction mixture was then stirred for 3.5 h at 60 ºC and a further 12 hrs at rt. EtOAc (3 x 15 mL) was added to extract the product from the aqueous solution. The organic layers were separated, combined, and dried over Na_2SO_4, before being concentrated under vacuum. The crude product was then purified by flash chromatography (pet ether/ EtOAc: 90:10) as a white solid (612 mg, 2.23 mmol, 67.0%).

mp 70-71 ºC.

$[\alpha]_D^{25}$ +22.0º (c 0.1 in $CHCl_3$) 95.4 % ee.

^1H NMR (400 MHz, $CDCl_3$) δ 7.34 – 7.25 (m, 2H), 7.01 – 6.91 (m, 3H), 6.90 – 6.78 (m, 4H), 4.42 – 4.30 (m, 1H), 4.18 – 4.03 (m, 4H), 3.77 (s, 3H), 2.62 (d, *J* = 5.1 Hz, 1H).

^{13}C NMR (101 MHz, $CDCl_3$) δ 158.46, 154.23, 152.62, 129.57, 121.27, 115.61, 114.73, 114.59, 69.52, 68.90, 68.70, 55.75.

Enantiomeric excess determined by HPLC analysis (CHIRALPAK IC column, hexane 95:05 iPrOH, 1.0 mL/min, T = 25°C, λ = 210 nm, *R* enantiomer 29.1 min, *S*-enantiomer 27.2 min. 95.4% ee (*S*).

m/z (ESI) 421.2 ([M + Na]+, 100 %).

For control experiment, this compound was already been reported via ATH in the following reference and was reduced in 7% ee.

Forshaw, S.; Matthews, A. J.; Brown, T. J.; Diorazio, L. J.; Williams L, Wills M. *Org. Lett*, **2017**, *19*, 2789-2792.

3-Phenoxy-1-(phenylsulfonyl)butan-2-ol (mixture of diastereomers)

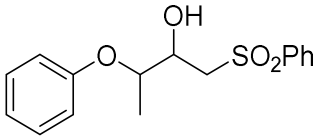

This compound is novel.

To a solution of methyl phenyl sulphone (812 mg, 5.20 mmol, 1.1 equiv) in dry THF (25 mL) was added *n*BuLi (2.2 M in *n*-hexane, 2.58 mL, 5.20 mmol, 1.1 equiv) dropwise at –78 ºC under nitrogen atmosphere. After the reaction mixture had been stirred at –78 ºC for 30 minutes, 2-phenoxypropanal (700 mg, 4.73 mmol, 1.0 equiv) was added dropwise at -78 ºC. Upon stirring at same temperature for 1 h, the reaction mixture was stirred at ambient temperature for 1 h. It was then concentrated under reduced pressure, extracted with ethyl acetate (2 x 20 mL), washed with brine (50 mL), dried over Na_2SO_4, filtered, concentrated, and purified by column chromatography on silica gel (pet ether/ EtOAc: 80:20) to yield 3-phenoxy-1-(phenylsulfonyl)butan-2-ol as colourless oil (787 mg, 2.57 mmol, 54.4%).

This compound was prepared in enantiomerically-enriched form following procedure D, using 3-phenoxy-1-(phenylsulfonyl)butan-2-one **26** (153 mg, 0.503 mmol, 1.0 equiv), FA/TEA (0.5 mL), [(*R,R*)Teth-TsDpenRuCl] (3.1 mg, 5.0 x 10^{-3} mmol, 1 mol%) and DCM (3 mL). (*S)*- 3-phenoxy-1-(phenylsulfonyl)butan-2-ol was isolated by flash chromatography (pet ether/ EtOAc: 80:20) as a colourless oil (136 mg, 0.444 mmol, 80.0%).

(found (ESI) [M+Na]+, 329.0817. $C_{16}H_{18}NaO_4S$ requires 329.0818)

ν_{max}: 3502 (broad), 3062, 2928, 1596, 1491, 1291, 1141, 1070, 744, 686, 525 cm^{-1}.

^{1}H NMR (300 MHz, $CDCl_3$) δ 8.06 – 7.89 (m, 4H), 7.76 – 7.51 (m, 6H), 7.34 – 7.23 (m, 4H), 7.05 – 6.92 (m, 2H), 6.90 – 6.76 (m, 4H), 4.46 (dd, *J* = 11.1, 5.0 Hz, 1H), 4.42 – 4.21 (m, 3H), 3.56 (d, *J* = 14.4 Hz, 1H), 3.37 (ddd, *J* = 33.2, 18.1, 11.1 Hz, 5H), 1.33 (dd, *J* = 6.1, 2.1 Hz, 6H).

^{13}C NMR (101 MHz, $CDCl_3$) δ 157.02, 139.27, 134.11, 134.02, 133.72, 129.65, 129.48, 129.41, 129.39, 127.98, 127.37, 121.68, 121.56, 116.10, 116.02, 75.65, 74.88, 69.28, 68.00, 58.86, 58.72, 44.50, 15.65, 14.62.

Enantiomeric excess and diastereomeric ratio determined by HPLC analysis (CHIRALPAK IC column, hexane 80:20 iPrOH, 1.0 mL/min, T = 25°C, λ = 210 nm, Ketone 31.2, t_R = 21.1 min (major, d1), 23.1 min (minor, d2), 26.3 min (major, d2), 27.3 min (minor, d1). 52.5:47.5 dr, 94.4% ee (d1), >99.9% ee (d2).
m/z (ESI) 329.2 ([M + Na]+, 100 %).

3-Phenoxy-1-(phenylsulfonyl)butan-2-one 26

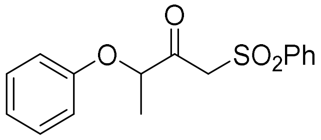

This compound is known but not fully characterised.
Syntex (U.S.A.) Inc.US4888358, **1989**, A
This compound was prepared following procedure F using: 3-phenoxy-1-(phenylsulfonyl)butan-2-ol (780 mg, 2.72 mmol, 1 equiv), PCC (1.17 g, 5.45 mmol, 2.0 equiv) and DCM (30 mL). 3-phenoxy-1-(phenylsulfonyl)butan-2-one was isolated by flash chromatography (pet ether/ EtOAc: 80:20) as a white solid (373 mg, 1.23 mmol, 47.8%).
mp 99-100 ºC.
(found (ESI) [M+Na]+, 327.0663. $C_{16}H_{16}NaO_4S$ requires 327.0662)
ν_{max}: 2993, 1732, 1320, 1235, 1148, 1044, 799, 682, 515 cm^{-1}.
^{1}H NMR (300 MHz, $CDCl_3$) δ 7.90 – 7.75 (m, 2H), 7.66 – 7.54 (m, 1H), 7.53 – 7.39 (m, 2H), 7.26 – 7.11 (m, 2H), 6.93 (t, *J* = 7.3 Hz, 1H), 6.76 (d, *J* = 8.1 Hz, 2H), 4.70 (q, *J* = 6.8 Hz, 1H), 4.42 (d, *J* = 14.8 Hz, 1H), 4.26 (d, *J* = 14.8 Hz, 1H), 1.39 (d, *J* = 6.8 Hz, 3H).
^{13}C NMR (101 MHz, $CDCl_3$) δ 199.21, 156.64, 139.09, 134.21, 129.90, 129.26, 128.51, 122.31, 115.33, 78.76, 61.37, 16.37.
m/z (ESI) 327.3 ([M + Na]+, 100 %).

3-Phenyl-1-(phenylsulfonyl)butan-2-ol (mixture of diastereomers)

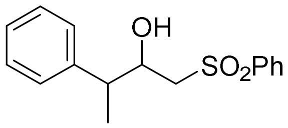

This compound is novel.
To a solution of methyl phenyl sulphone (1.01 g, 6.47 mmol, 1.0 equiv) in dry THF (25 mL) was added *n*BuLi (2.5 M in *n*-hexane, 3.50 mL, 7.05 mmol, 1.1 equiv) dropwise at –78 ºC under nitrogen atmosphere. After the reaction mixture had been stirred at –78 ºC for 30

minutes, 2-phenylpropanal (1.03 g, 7.69 mmol, 1.0 equiv) was added dropwise at -78 ºC. Upon stirring at same temperature for 1 h, the reaction mixture was stirred at ambient temperature for 1 h. It was then concentrated under reduced pressure, extracted with ethyl acetate (2 x 20 mL), washed with brine (50 mL), dried over Na_2SO_4, filtered, concentrated, and purified by column chromatography on silica gel (pet ether/ EtOAc: 80:20) to yield 3-phenyl-1-(phenylsulfonyl)butan-2-ol as colourless oil (1.57 g, 5.41 mmol, 81.5%).

This compound was prepared in enantiomerically-enriched form following procedure D, using 3-phenyl-1-(phenylsulfonyl)butan-2-one **27** (76 mg, 0.25 mmol, 1.0 equiv), FA/TEA (0.25 mL), [(*R,R*)Teth-TsDpenRuCl] (1.5 mg, 2.4 x 10^{-3} mmol, 1 mol%) and DCM (2 mL). (*S)*- 3-phenyl-1-(phenylsulfonyl)butan-2-ol was isolated by flash chromatography (pet ether/ EtOAc: 80:20) as a colourless oil (60 mg, 0.20 mmol, 78%).

(found (ESI) [M+Na]+, 313.0872. $C_{16}H_{18}NaO_3S$ requires 313.0869)

ν_{max}: 3507 (broad), 3061, 2968, 1447, 1299, 1135, 1082, 1012, 742, 686, 528 cm^{-1}.

^1H NMR (400 MHz, $CDCl_3$) δ 7.90 – 7.76 (m, 10H), 7.73 – 7.61 (m, 5H), 7.59 – 7.48 (m, 10H), 7.29 – 7.16 (m, 19H), 7.00 (dd, *J* = 7.6, 1.5 Hz, 6H), 4.30 (ddd, *J* = 14.4, 7.5, 4.8 Hz, 2H), 4.19 – 4.09 (m, 3H), 3.36 (d, *J* = 2.5 Hz, 3H), 3.16 (dd, *J* = 14.3, 1.6 Hz, 2H), 3.09 – 3.02 (m, 9H), 2.91 – 2.82 (m, 2H), 2.77 (p, *J* = 7.0 Hz, 3H).

^{13}C NMR (101 MHz, $CDCl_3$) δ 142.50, 141.20, 138.93, 133.92, 129.37, 128.80, 128.56, 128.21, 127.94, 127.90, 127.53, 127.05, 127.02, 70.55, 70.02, 60.41, 60.25, 45.57, 44.86, 17.50, 16.64.

Enantiomeric excess and diastereomeric ratio determined by HPLC analysis (CHIRALPAK IC column, hexane 90:10 iPrOH, 1.0 mL/min, T = 25°C, λ = 210 nm, Ketone 42.7 and 50.5 min, t_R = 51.2 min (major, d1), 59.6 min (minor, d2), 63.5 min (major, d2), 68.2 min (minor, d1). 59.5:40.5 dr, 2.6% ee (d2), 83.4% ee (d1).

m/z (ESI) 313.2 ([M + Na]+, 100 %).

3-Phenyl-1-(phenylsulfonyl)butan-2-one 27

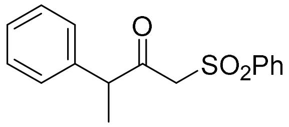

This compound is Novel.

This compound was prepared following procedure F using: 3-phenyl-1-(phenylsulfonyl)butan-2-ol (1.57 g, 5.40 mmol, 1 equiv), PCC (2.32 g, 10.8 mmol, 2.0 equiv) and DCM (30 mL). 3-

phenyl-1-(phenylsulfonyl)butan-2-one was isolated by flash chromatography (pet ether/ EtOAc: 80:20) as a white solid (987 mg, 3.43 mmol, 62.9%).
mp 105-106 °C.
(found (ESI) [M+Na]+, 311.0711. $C_{16}H_{16}NaO_3S$ requires 311.0712)
ν_{max}: 3000, 2933, 1706, 1449, 1311, 1153, 1029, 728, 686, 527 cm^{-1}.
^{1}H NMR (300 MHz, $CDCl_3$) δ 7.94 – 7.81 (m, 2H), 7.77 – 7.65 (m, 1H), 7.65 – 7.50 (m, 2H), 7.45 – 7.23 (m, 3H), 7.16 (d, *J* = 7.2 Hz, 2H), 4.27 (d, *J* = 13.7 Hz, 1H), 4.13 (q, *J* = 6.8 Hz, 1H), 3.90 (d, *J* = 13.7 Hz, 1H), 1.40 (d, *J* = 6.8 Hz, 3H).
^{13}C NMR (101 MHz, $CDCl_3$) δ 197.95, 138.76, 138.46, 134.24, 129.43, 129.30, 128.36, 128.23, 127.87, 64.85, 53.98, 17.07.
m/z (ESI) 311.2 ([M + Na]+, 100 %).

Julia-Kocienski Olefination

Synthesis of 1-(benzo[d]thiazol-2-ylsulfonyl)-3-phenoxypropan-2-one 28

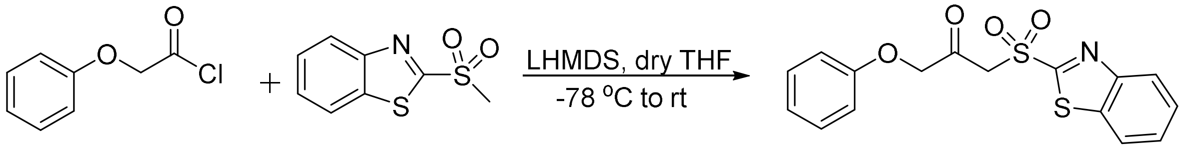

This compound is novel.
To a solution of 2-(methylsulfonyl)benzo[d]thiazol-6-ylium (1.0 g, 4.71 mmol, 1.0 equiv) in dry THF (25 mL) was added (LHMDS, 1.0 M in *n*-hexane, 10.4 mL, 10.4 mmol, 2.2 equiv) dropwise at –78 °C under nitrogen atmosphere. After the reaction mixture had been stirred at – 78 °C for 30 minutes, 2-phenoxyacetyl chloride (882 mg, 5.18 mmol, 1.10 equiv) was added dropwise at -78 °C. Upon stirring at same temperature for 1 h, the reaction mixture was stirred at ambient temperature for 1 h. It was then concentrated under reduced pressure, extracted with ethyl acetate (2 x 30 mL), washed with brine (40 mL), dried over Na_2SO_4, filtered, concentrated, and purified by column chromatography on silica gel (pet ether/ EtOAc: 80:20) to yield 1-(benzo[d]thiazol-2-ylsulfonyl)-3-phenoxypropan-2-one as brown solid (442 mg, 1.52 mmol, 32.3%).
mp 72-73 °C
(found (ESI) [M+Na]+, 370.0179. $C_{16}H_{13}NNaO_4S_2$ requires 370.0178)
ν_{max}: 2987, 2936, 1736, 1473, 1337, 1218, 1154, 1024, 751, 513, 451 cm^{-1}.
^{1}H NMR (400 MHz, $CDCl_3$) δ 8.21 – 8.13 (m, 1H), 8.03 – 7.95 (m, 1H), 7.67 – 7.55 (m, 2H), 7.31 – 7.22 (m, 2H), 7.05 – 6.96 (m, 1H), 6.87 – 6.79 (m, 2H), 4.86 (s, 2H), 4.75 (s, 2H).

^{13}C NMR (101 MHz, $CDCl_3$) δ 194.69, 157.01, 152.37, 136.96, 129.80, 128.35, 127.83, 125.58, 122.41, 122.34, 114.54, 72.71, 61.39.
m/z (ESI) 370.1 ([M + Na]+, 100 %).

Racemic and (*S*)-1-(benzo[d]thiazol-2-ylsulfonyl)-3-phenoxypropan-2-ol 29

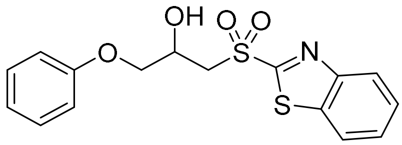

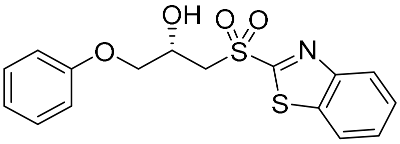

This compound is novel.

This compound was prepared in racemic form following procedure D using: 1-(benzo[d]thiazol-2-ylsulfonyl)-3-phenoxypropan-2-one **28** (116 mg, 0.334 mmol, 1 equiv), FA/TEA (0.5 mL), [(*R,R*)Teth-TsDpenRuCl] (0.62 mg, 1.0 x 10^{-3} mmol, 0.05 mol%), [(*S,S*)Teth-TsDpenRuCl] (0.65 mg, 1.0 x 10^{-3} mmol, 0.05 mol%), and DCM (3 mL). 1-(benzo[d]thiazol-2-ylsulfonyl)-3-phenoxypropan-2-ol was isolated by flash chromatography (pet ether/ EtOAc: 80:20) as a brown solid (100 mg, 0.286 mmol, 85.7%). The racemic compound was prepared by this method because $NaBH_4$ reduction was not successful.

This compound was prepared in enantiomerically-enriched form following procedure D, using 1-(benzo[d]thiazol-2-ylsulfonyl)-3-phenoxypropan-2-one **28** (205 mg, 0.591 mmol, 1.0 equiv), FA/TEA (0.5 mL), [(*R,R*)Teth-TsDpenRuCl] (3.5 mg, 5.6 x 10^{-3} mmol, 1 mol%) and DCM (3 mL). (*S*)- 1-(benzo[d]thiazol-2-ylsulfonyl)-3-phenoxypropan-2-ol was isolated by flash chromatography (pet ether/ EtOAc: 80:20) as a brown solid (192 mg, 0.550 mmol, 93.1%).

mp 103-104 °C
$[\alpha]_D^{25}$ +33.0° (c 0.1 in $CHCl_3$) 94.0 % ee (*S*)
(found (ESI) [M+Na]+, 372.0339. $C_{16}H_{15}NNaO_4S_2$ requires 372.0335)
ν_{max}: 3374 (broad), 2988, 2936, 1491, 1310, 1147, 1082, 1028, 755, 591, 512 cm^{-1}.
^{1}H NMR (400 MHz, CDCl3) δ 8.24 – 8.15 (m, 1H), 8.07 – 7.93 (m, 1H), 7.68 – 7.52 (m, 2H), 7.30 – 7.18 (m, 2H), 6.96 (t, *J* = 7.4 Hz, 1H), 6.87 (d, *J* = 7.9 Hz, 2H), 4.84 – 4.67 (m, 1H), 4.20 – 4.01 (m, 2H), 3.98 – 3.79 (m, 2H), 3.57 (d, *J* = 4.2 Hz, 1H).
^{13}C NMR (101 MHz, CDCl3) δ 157.91, 152.35, 136.61, 129.59, 128.30, 127.88, 125.46, 122.41, 121.63, 114.55, 69.77, 65.36, 58.77.

Enantiomeric excess determined by HPLC analysis (CHIRALPAK IG column, hexane 90:10 iPrOH, 1.0 mL/min, T = 25°C, λ = 210 nm, Ketone 41.4 min, *R* enantiomer 86.0 min, *S*-enantiomer 75.9 min). 94.0% ee (*S*).

m/z (ESI) 372.1 ([M + Na]+, 100 %).

Synthesis of protected hydroxyl

(*S*)-2-((2-((tert-butyldimethylsilyl)oxy)-3-phenoxypropyl)sulfonyl)benzo[d]thiazole 30

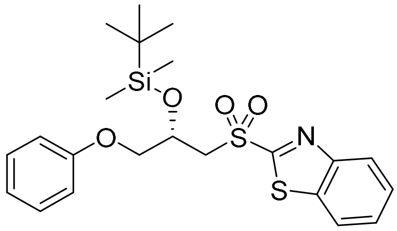

This compound is novel.

This compound was prepared using following this procedure:- To a stirred solution of (*S*)- 1-(benzo[d]thiazol-2-ylsulfonyl)-3-phenoxypropan-2-ol **29** (230 mg, 0.659 mmol, 1 equiv.) and imidazole (112 mg, 1.65 mmol, 2.50 equiv.) in DMF (10 mL) was added tert-butyldimethylsilyl chloride (196 mg, `1.30 mmol, 2.0 equiv.) at room temperature. The mixture was stirred for 4 h and then quenched by water (20 mL) at 0 °C. The mixture was extracted with ethyl acetate (2 x 20 mL). The collected organic layer was washed with water (2 × 30 mL) and then dried ($MgSO_4$). The solvent was evaporated and the residue was purified by flash chromatography (pet ether/ EtOAc: 90:10) as a colourless oil (144 mg, 0.311 mmol, 47.2%).

Onishi, Y.; Nishimoto, Y.; Yasuda, M.; Baba, A. *Org. Lett.,* **2011**, *13*, 2762-2765.

(found (ESI) [M+Na]+, 486.1201. $C_{22}H_{29}NNaO_4S_2Si$ requires 486.1199)

ν_{max}: 2927, 2855, 1599, 1495, 1242, 1147, 835, 752, 689, 512 cm^{-1}.

^{1}H NMR (400 MHz, $CDCl_3$) δ 8.15 – 8.07 (m, 1H), 7.98 – 7.88 (m, 1H), 7.61 – 7.43 (m, 2H), 7.20 – 7.14 (m, 2H), 6.86 (t, *J* = 7.4 Hz, 1H), 6.76 (d, *J* = 7.9 Hz, 2H), 4.68 (p, *J* = 5.4 Hz, 1H), 4.07 – 3.90 (m, 3H), 3.81 – 3.69 (m, 1H), 0.74 (s, 9H), 0.00 (d, *J* = 3.5 Hz, 6H).

^{13}C NMR (101 MHz, $CDCl_3$) δ 158.10, 152.62, 136.73, 129.49, 128.04, 127.67, 125.46, 122.33, 121.24, 114.46, 70.47, 66.21, 58.70, 25.64, 17.97, -4.54, -4.90.

m/z (ESI) 486.2 ([M + Na]+, 100 %).

Julia-Kocienski addition to form 31.

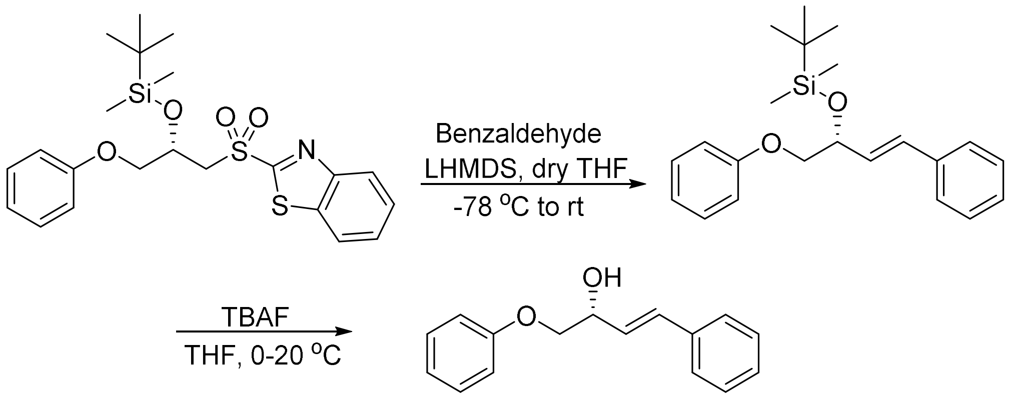

Procedure for Julia-Kocienski addition A magnetically stirred solution of (S)-2-((2-((tert-butyldimethylsilyl)oxy)-3-phenoxypropyl)sulfonyl)benzo[d]thiazole **30** (144 mg, 0.311 mmol, 1.0 equiv.) and benzaldehyde (66 mg, 0.62 mmol, 2.0 equiv.) in DME (7 mL) was maintained at -78 °C under nitrogen atmosphere. The resulting mixture was then treated with LHMDS (LHMDS, 1.0 M in *n*-hexane, 0.7 mL, 0.7 mmol, 2.2 equiv) at -78 °C. Allowed it to warm at rt over 2 hours. After 2 hours, the reaction mixture was treated with 1N HCl and extracted with ethyl acetate (2 x 12 mL) to afford crude residue which was directly subjected to deprotection. To a precooled solution of crude compound (136 mg) at 0 °C in THF (5 mL) was added TBAF (1 M solution in THF, 0.6 mL) dropwise under nitrogen atmosphere. The solution was allowed to warm at rt and stirred at rt for 3 hours. After completion was extracted with ethyl acetate acetate (2 x 10 mL) and washed with brine solution and then dried ($MgSO_4$). The solvent was evaporated and the residue was purified by flash chromatography (pet ether/ EtOAc: 88:12) to yield (*R,Z*)-1-phenoxy-4-phenylbut-3-en-2-ol **31** as a colourless oil (27 mg, 0.11 mmol, 37%).

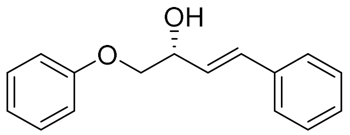

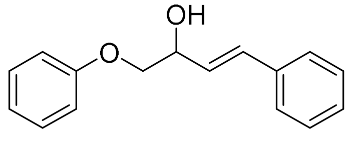

This compound is known and has been fully characterized.

$[\alpha]_D^{25}$ -38° (c 0.1 in $CHCl_3$) 90.8% ee (*R*) for *trans(E)*- isomer.

^{1}H NMR (400 MHz, $CDCl_3$) δ 7.37 – 7.17 (m, 7H), 6.96 – 6.83 (m, 3H), 6.71 (d, *J* = 16.0 Hz, 1H), 6.22 (dd, *J* = 16.0, 6.2 Hz, 1H), 4.72 – 4.59 (m, 1H), 4.08 – 3.98 (m, 1H), 3.96 – 3.85 (m, 1H), 2.45 (d, *J* = 3.5 Hz, 1H).

^{13}C NMR (101 MHz, $CDCl_3$) δ 136.40, 132.42, 129.59, 128.64, 127.97, 127.03, 126.60, 121.33, 114.69, 71.83, 71.14.

NMR for racemic 1-phenoxy-4-phenylbut-3-en-2-ol

^{1}H NMR (400 MHz, $CDCl_3$) δ 7.41 – 7.24 (m, 27H), 7.03 – 6.89 (m, 12H), 6.75 (dd, *J* = 18.3, 13.9 Hz, 4H), 6.29 (dd, *J* = 16.0, 6.2 Hz, 3H), 5.78 (dd, *J* = 11.7, 9.1 Hz, 1H), 5.03 – 4.92 (m, 1H), 4.77 – 4.66 (m, 3H), 4.11 – 3.93 (m, 8H), 2.55 (d, *J* = 3.5 Hz, 3H), 2.49 (d, *J* = 3.5 Hz, 1H).

^{13}C NMR (101 MHz, $CDCl_3$) δ 158.45, 136.41, 133.86, 132.41, 129.59, 129.07, 128.78, 128.64, 128.45, 127.98, 127.62, 127.05, 126.61, 121.33, 121.30, 114.70, 114.64, 71.83, 71.38, 71.14, 66.75.

Enantiomeric excess and diastereomeric ratio determined by HPLC analysis (CHIRALPAK IA column, hexane 94:06 iPrOH, 1.0 mL/min, T = 25°C, λ = 210 nm, t_R = 15.8 min (major, d1), 18.7 min (minor, d1), 20.4 min (major, d2), 21.8 min (minor, d2). 98.8 (*trans/E*):1.2 (*cis/Z*) dr, 90.8% ee (d2, *trans/E*), >99.9% ee (d1, *cis/Z*).

m/z (ESI) 263.2 ([M + Na]+, 100 %).

For control experiments, 1-phenoxy-4-phenylbut-3-en-2-ol prepared via ATH using procedure D: (*E/Z*)-1-phenoxy-4-phenylbut-3-en-2-one (100 mg, 0.420 mmol, 1.0 equiv), FA/TEA (0.5 mL), [(*R,R*)Teth-TsDpenRuCl] (2.6 mg, 4.19 x 10^{-3} mmol, 1 mol%) and DCM (3 mL). 1-phenoxy-4-phenylbut-3-en-2-ol was isolated by flash chromatography (pet ether/ EtOAc: 88:12) as a colourless oil (91 mg, 0.38 mmol, 90%).

Enantiomeric excess determined by HPLC analysis (CHIRALPAK IC column, hexane 95:05 iPrOH, 0.5 mL/min, T = 25°C, λ = 210 nm, Cis enantiomer t_{major} = 29.0 min t_{minor} = 34.4 min, Trans enantiomer t_{minor} = 37.2 min t_{major} = 49.2 min. *Cis(Z)/Trans(E)* ratio 30:70, *Cis(Z)* isomer 54.2% ee (*S*). *Trans(E)* isomer 37.6% ee (*S*).

NMR spectra and HPLC data

1-Cyclohexyl-2-(phenylsulfonyl)ethan-1-one 10a

^{1}H NMR (400 MHz, $CDCl_3$)

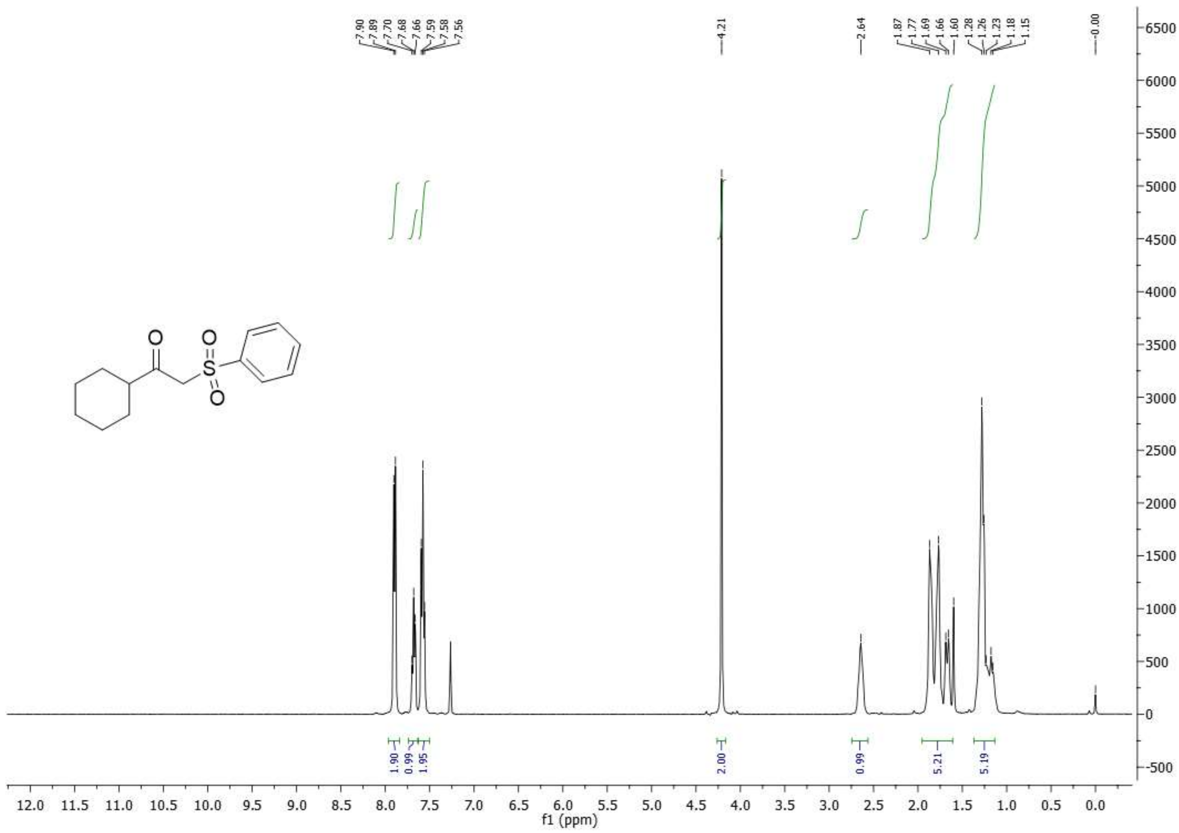

^{13}C NMR (101 MHz, $CDCl_3$

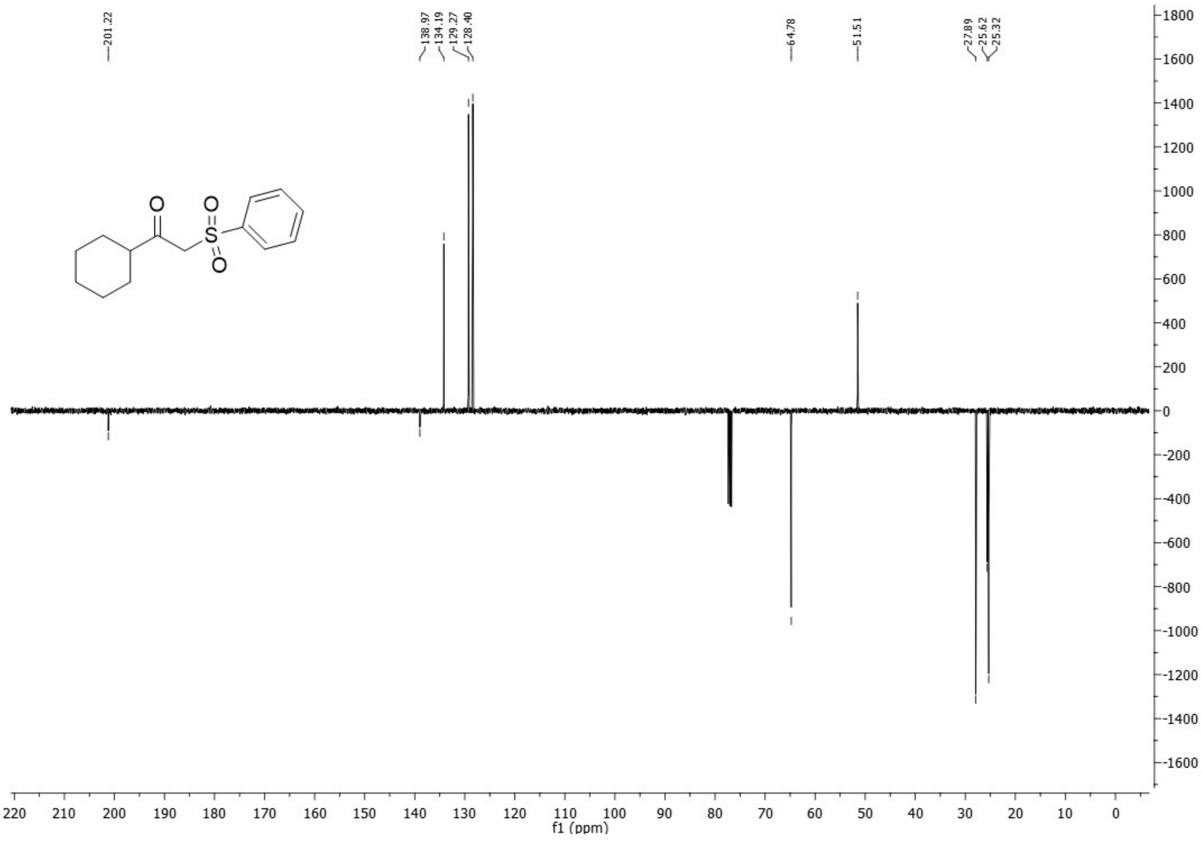

Ketone HPLC of 1-cyclohexyl-2-(phenylsulfonyl)ethan-1-one.

Clarity - Chromatography SW

DataApex 2006

www.dataapex.com

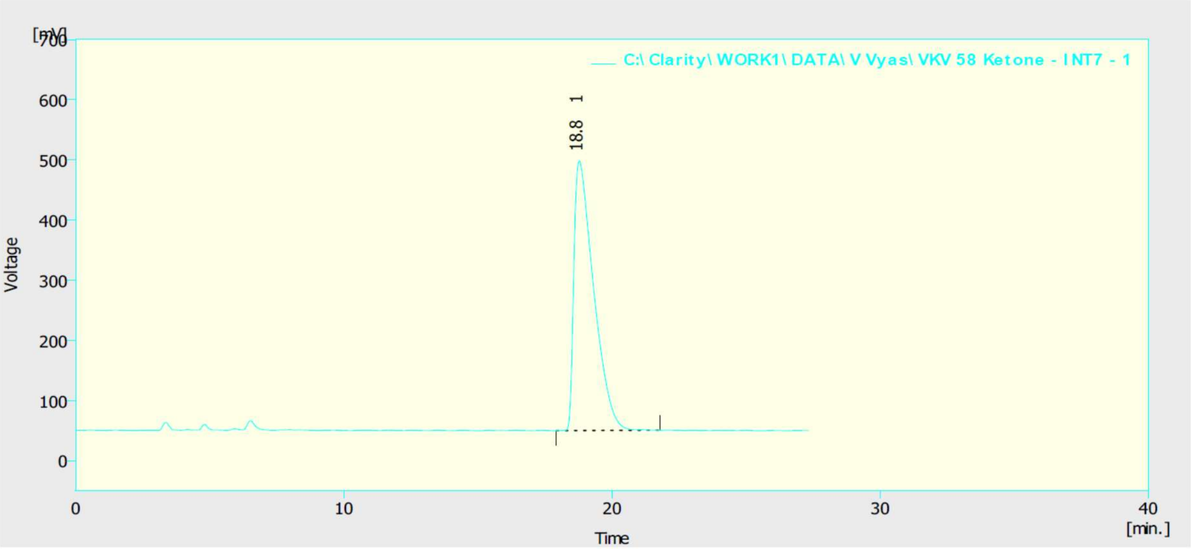

Result Table (Uncal - C:\Clarity\WORK1\DATA\V Vyas\VKV 58 Ketone - INT7 - 1)

	Reten. Time [min]	Area [mV.s]	Height [mV]	Area [%]	Height [%]	W05 [min]	Compound Name
1	18.783	22978.600	448.392	100.0	100.0	0.79	
	Total	22978.600	448.392	100.0	100.0		

1-Cyclohexyl-2-(phenylsulfonyl)ethan-1-ol 11a
^{1}H NMR (400 MHz, $CDCl_3$)

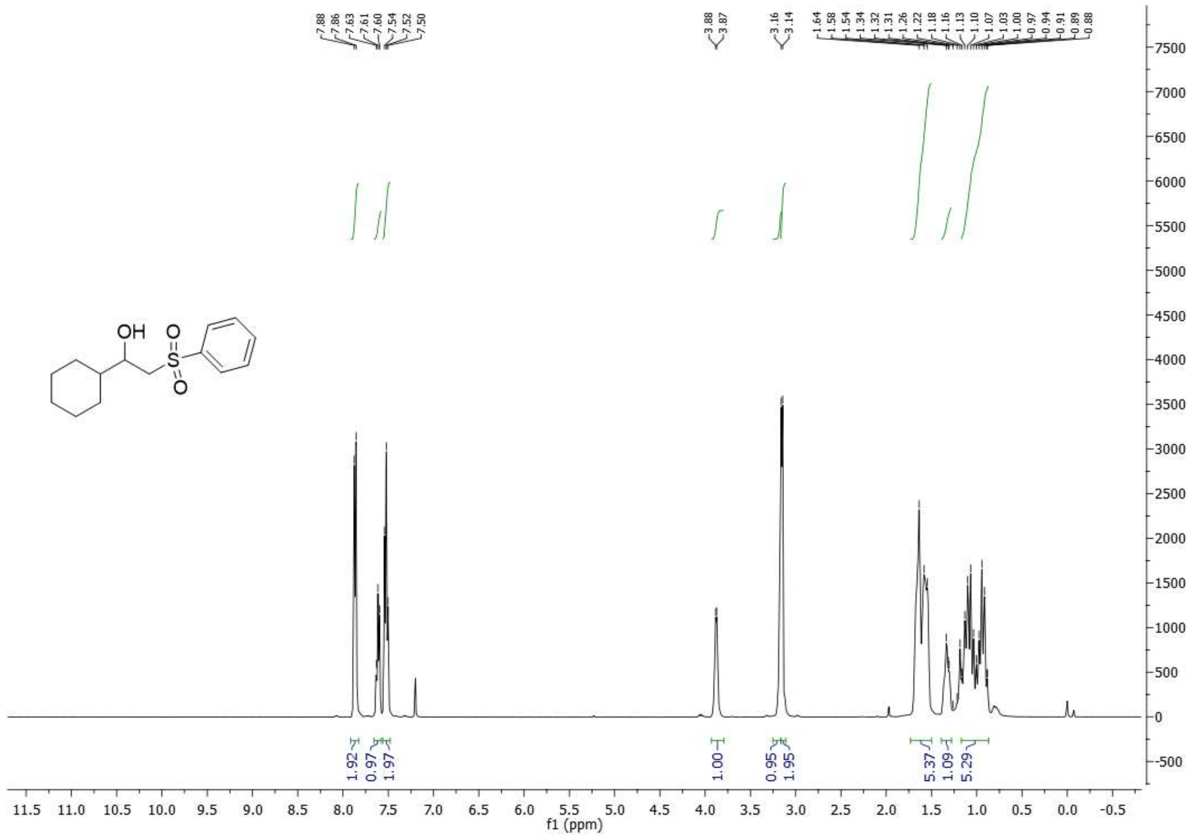

^{13}C NMR (101 MHz, $CDCl_3$

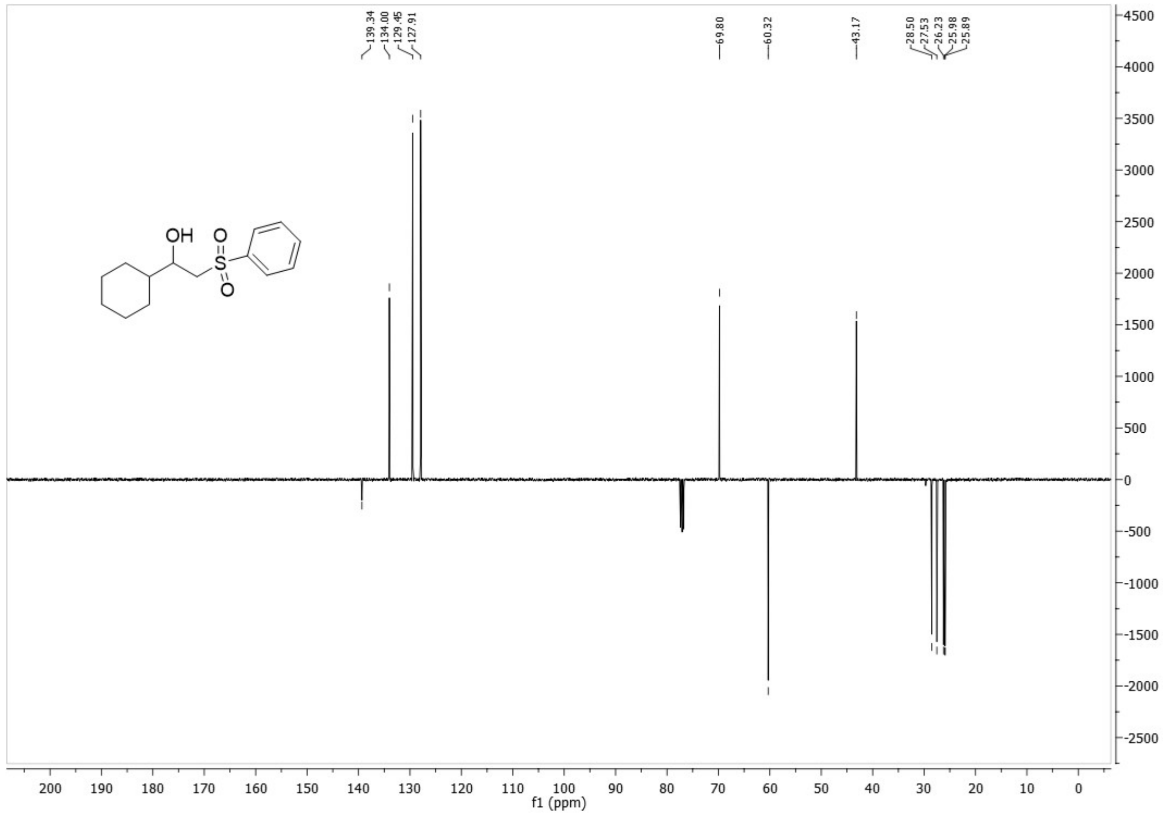

Racemic HPLC of 1-cyclohexyl-2-(phenylsulfonyl)ethan-1-ol.

Clarity - Chromatography SW
DataApex 2006
www.dataapex.com

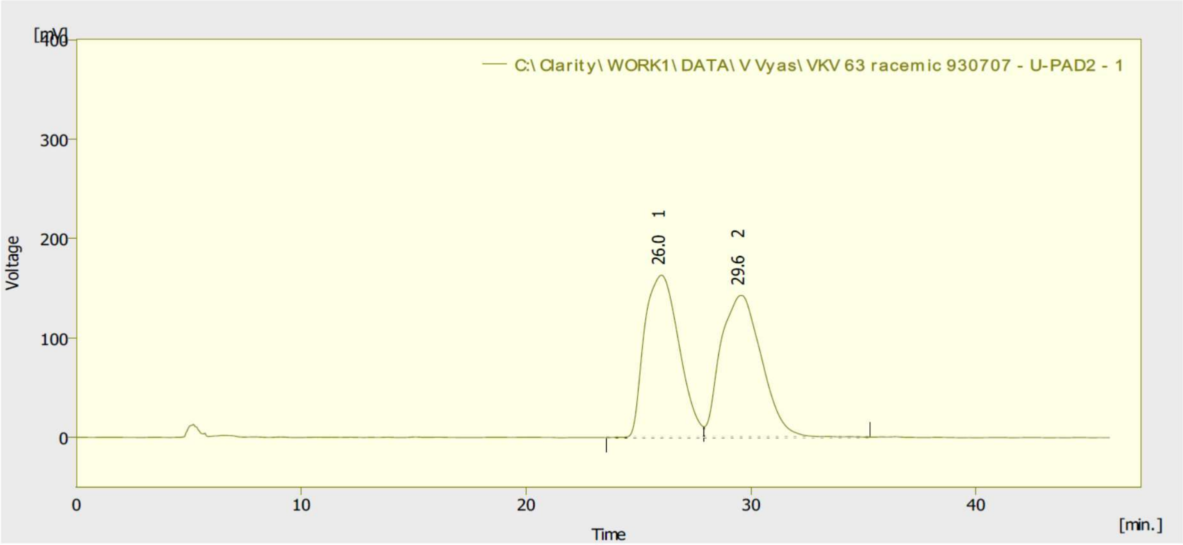

Result Table (Uncal - C:\Clarity\WORK1\DATA\V Vyas\VKV 63 racemic 930707 - U-PAD2 - 1)

	Reten. Time [min]	Area [mV.s]	Height [mV]	Area [%]	Height [%]	W05 [min]	Compound Name
1	26.012	17468.359	163.226	49.1	53.3	1.77	
2	29.556	18072.685	142.791	50.9	46.7	2.09	
	Total	35541.044	306.017	100.0	100.0		

HPLC after ATH 1-cyclohexyl-2-(phenylsulfonyl)ethan-1-ol. (100% conversion, 86.6% ee).

Clarity - Chromatography SW
DataApex 2006
www.dataapex.com

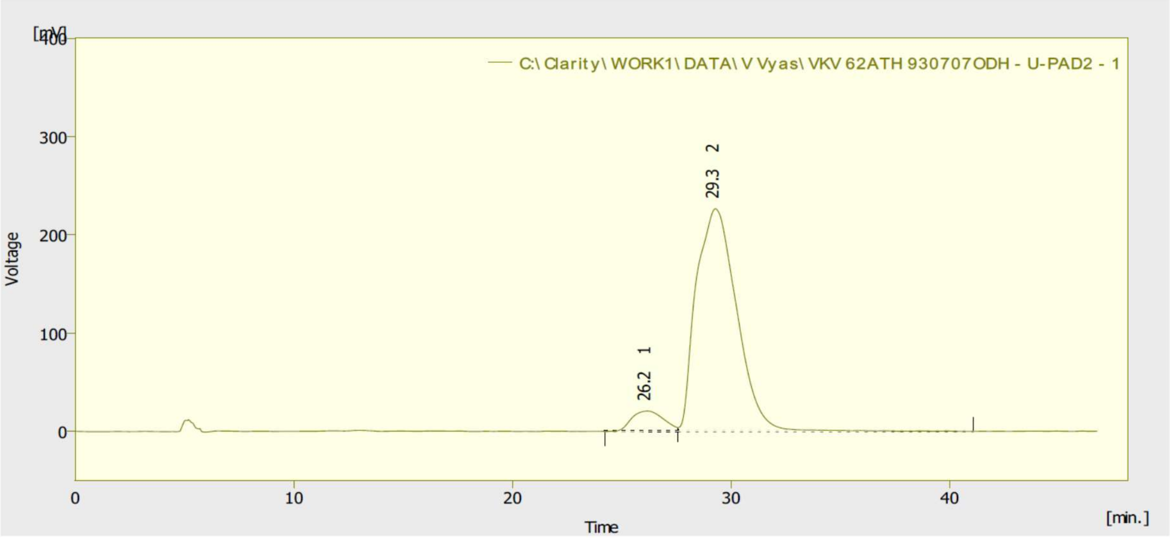

Result Table (Uncal - C:\Clarity\WORK1\DATA\V Vyas\VKV 62ATH 930707ODH - U-PAD2 - 1)

	Reten. Time [min]	Area [mV.s]	Height [mV]	Area [%]	Height [%]	W05 [min]	Compound Name
1	26.160	2098.578	20.680	6.7	8.4	1.71	
2	29.280	29274.946	226.443	93.3	91.6	2.13	
	Total	31373.524	247.122	100.0	100.0		

1-Cyclopentyl-2-(phenylsulfonyl)ethan-1-one 10b

^{1}H NMR (400 MHz, $CDCl_3$)

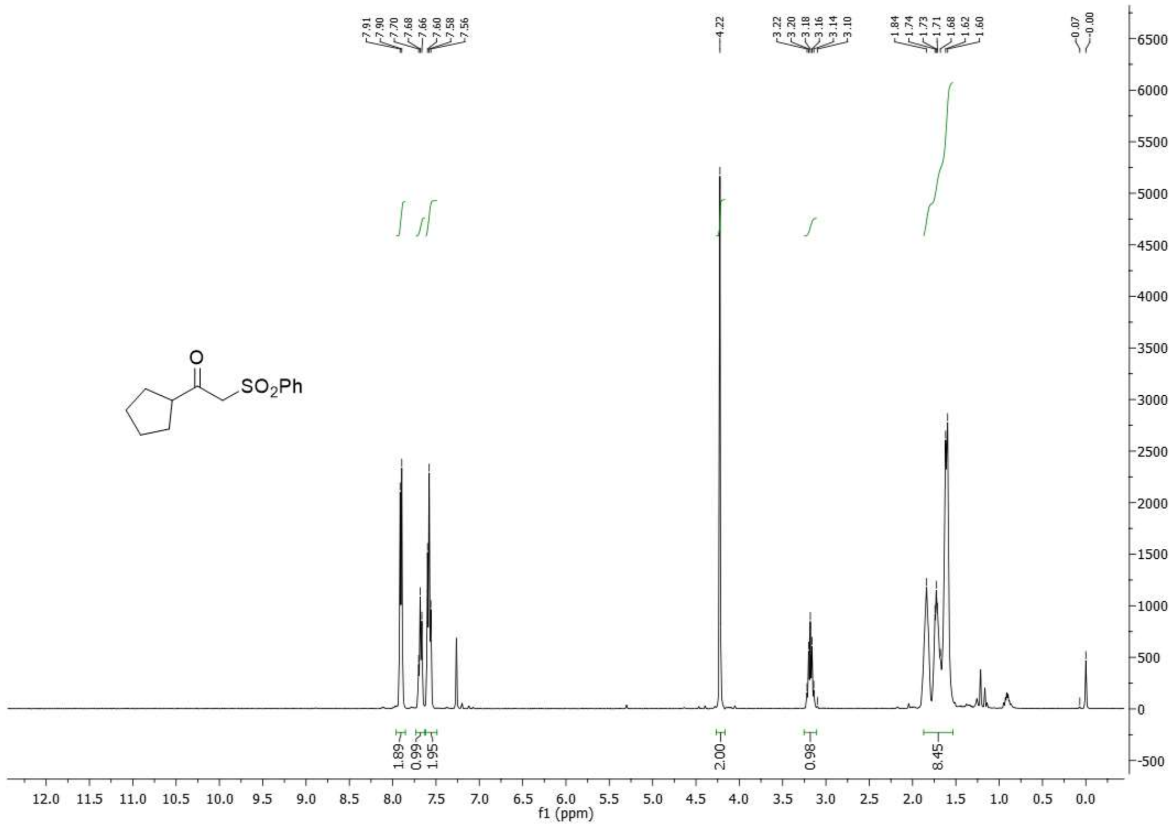

^{13}C NMR (101 MHz, $CDCl_3$

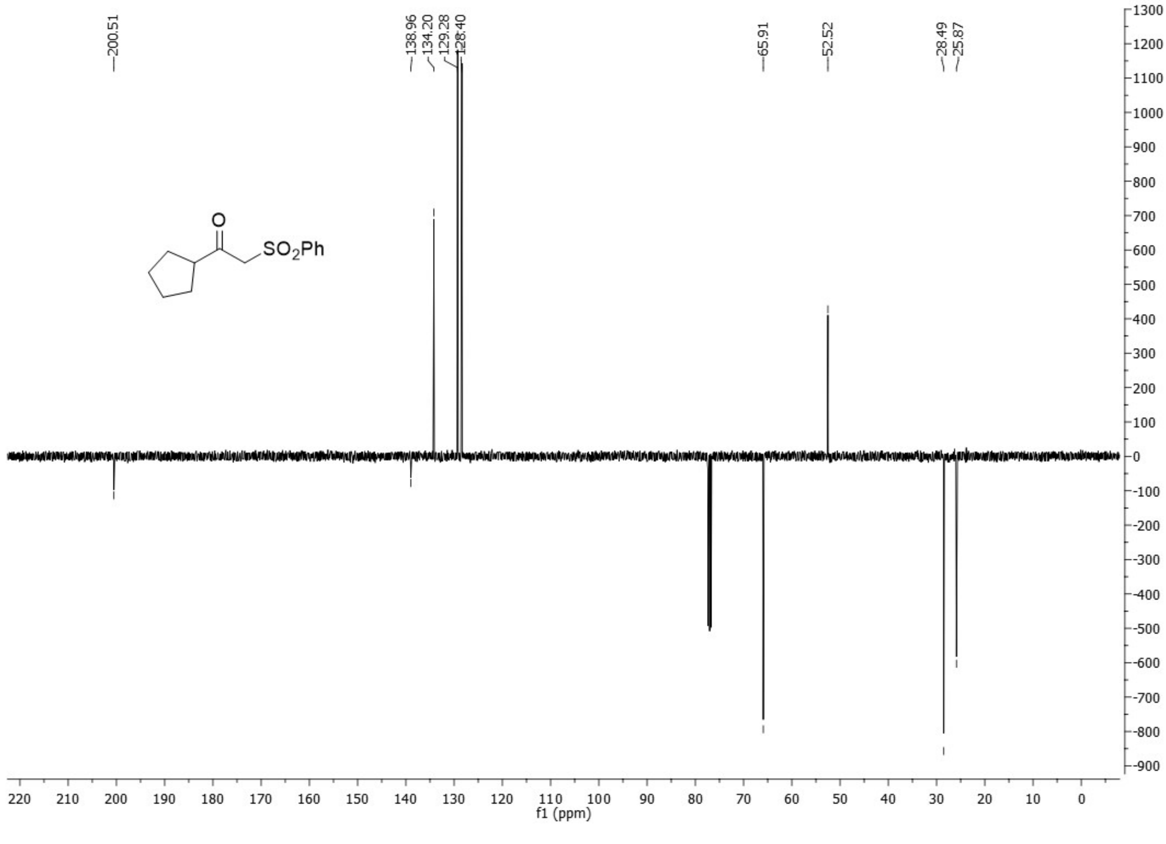

Ketone HPLC of 1-cyclopentyl-2-(phenylsulfonyl)ethan-1-one.

Clarity - Chromatography SW
DataApex 2006
www.dataapex.com

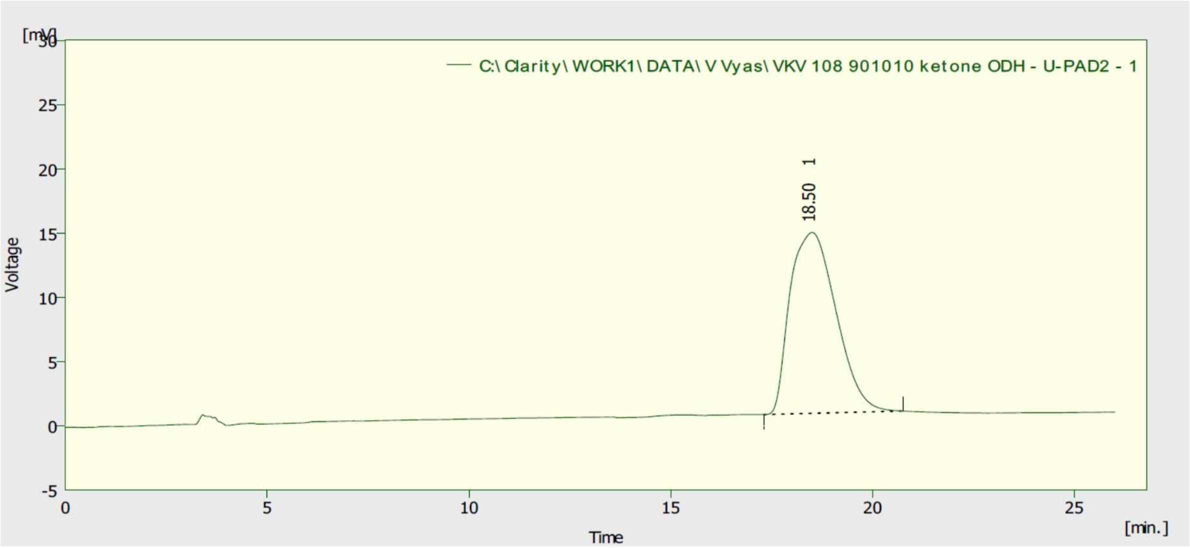

Result Table (Uncal - C:\Clarity\WORK1\DATA\V Vyas\VKV 108 901010 ketone ODH - U-PAD2 - 1)

	Reten. Time [min]	Area [mV.s]	Height [mV]	Area [%]	Height [%]	W05 [min]	Compound Name
1	18.500	1117.526	14.082	100.0	100.0	1.30	
	Total	1117.526	14.082	100.0	100.0		

1-Cyclopentyl-2-(phenylsulfonyl)ethan-1-ol 11b

^{1}H NMR (400 MHz, CDCl$_3$)

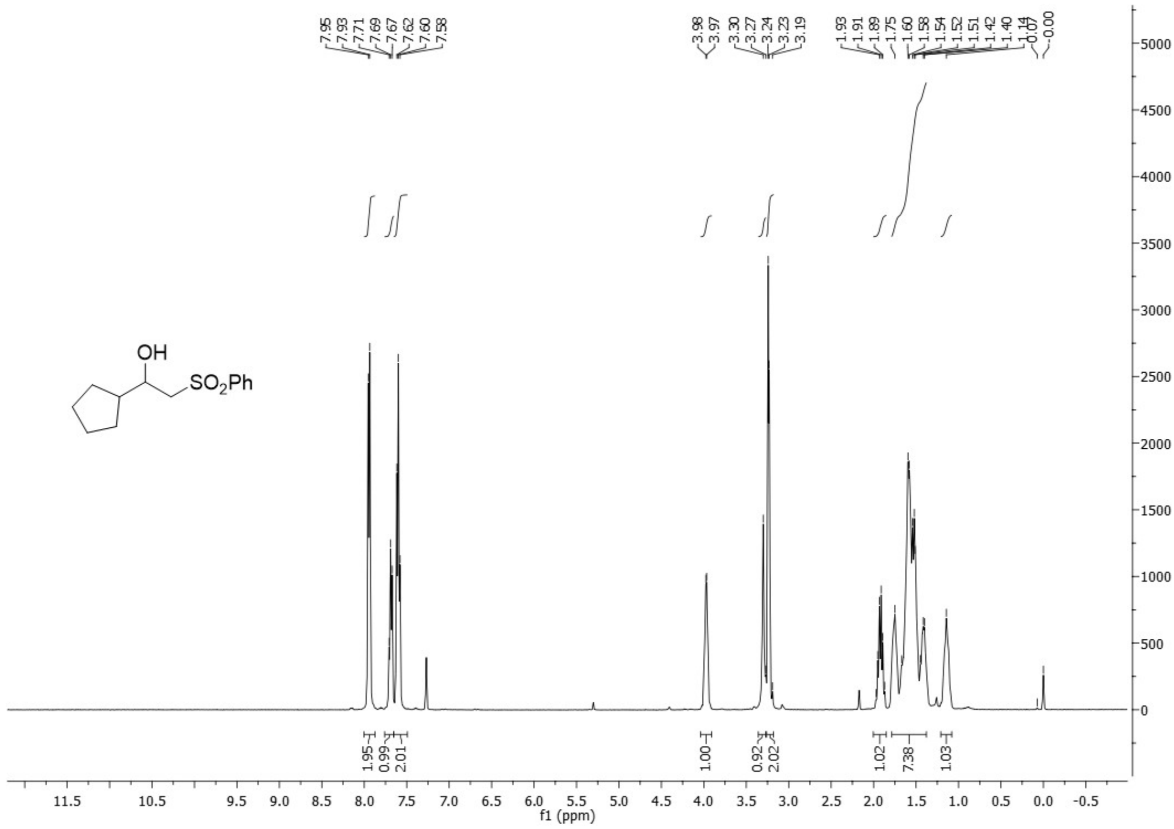

^{13}C NMR (101 MHz, CDCl$_3$

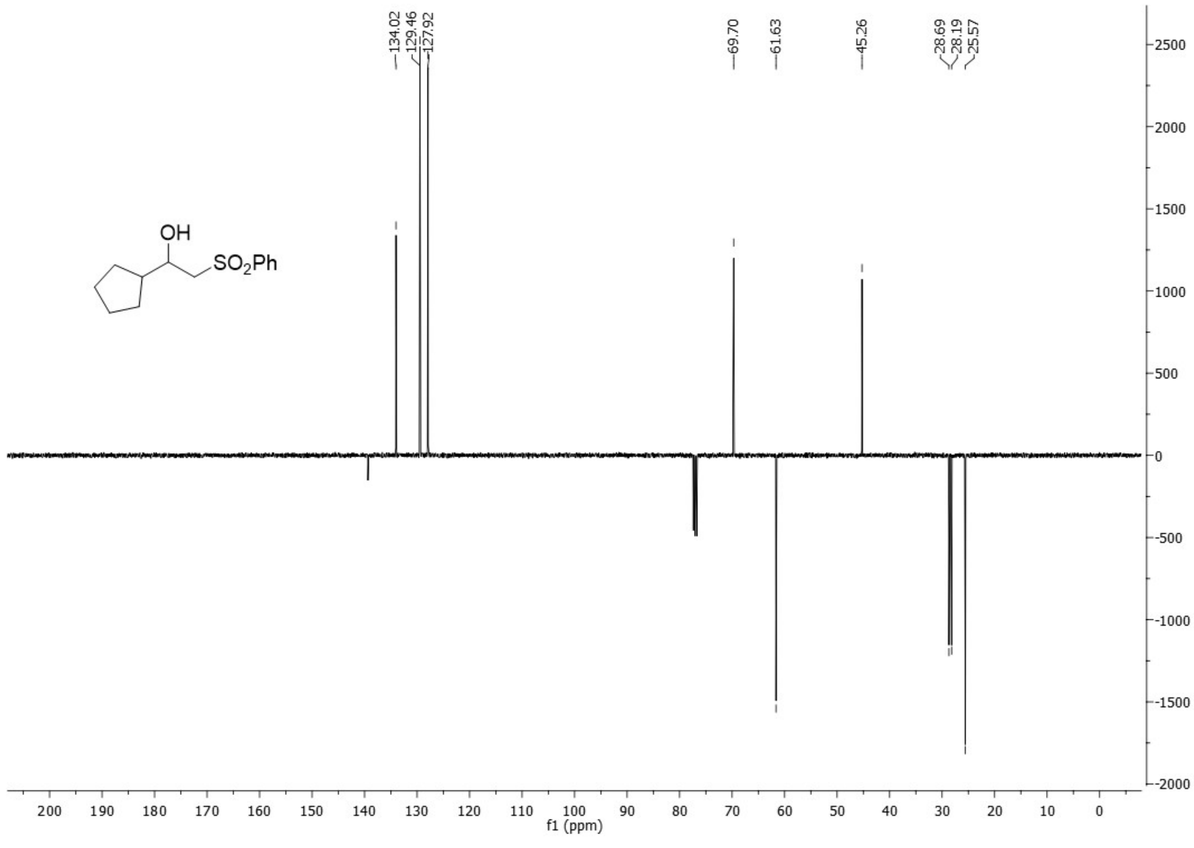

Racemic HPLC of 1-cyclopentyl-2-(phenylsulfonyl)ethan-1-ol.

Clarity - Chromatography SW
DataApex 2006
www.dataapex.com

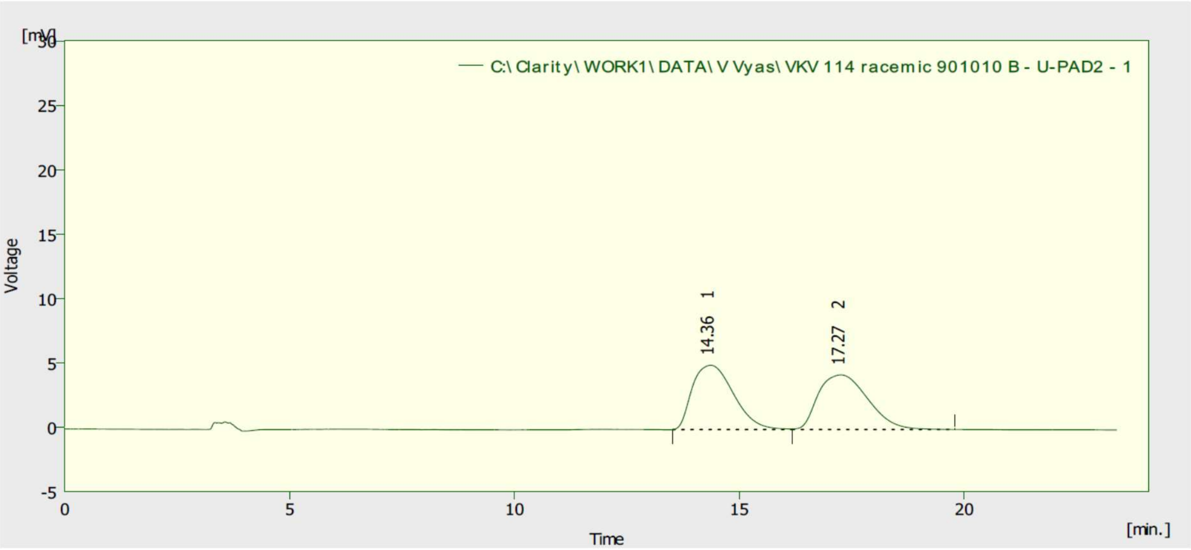

Result Table (Uncal - C:\Clarity\WORK1\DATA\V Vyas\VKV 114 racemic 901010 B - U-PAD2 - 1)

	Reten. Time [min]	Area [mV.s]	Height [mV]	Area [%]	Height [%]	W05 [min]	Compound Name
1	14.356	319.134	4.999	49.6	54.1	1.03	
2	17.268	324.634	4.244	50.4	45.9	1.24	
	Total	643.768	9.242	100.0	100.0		

Clarity - Chromatography SW
DataApex 2006
www.dataapex.com

HPLC after ATH 1-cyclopentyl-2-(phenylsulfonyl)ethan-1-ol. (100% conversion, 97.2% ee).

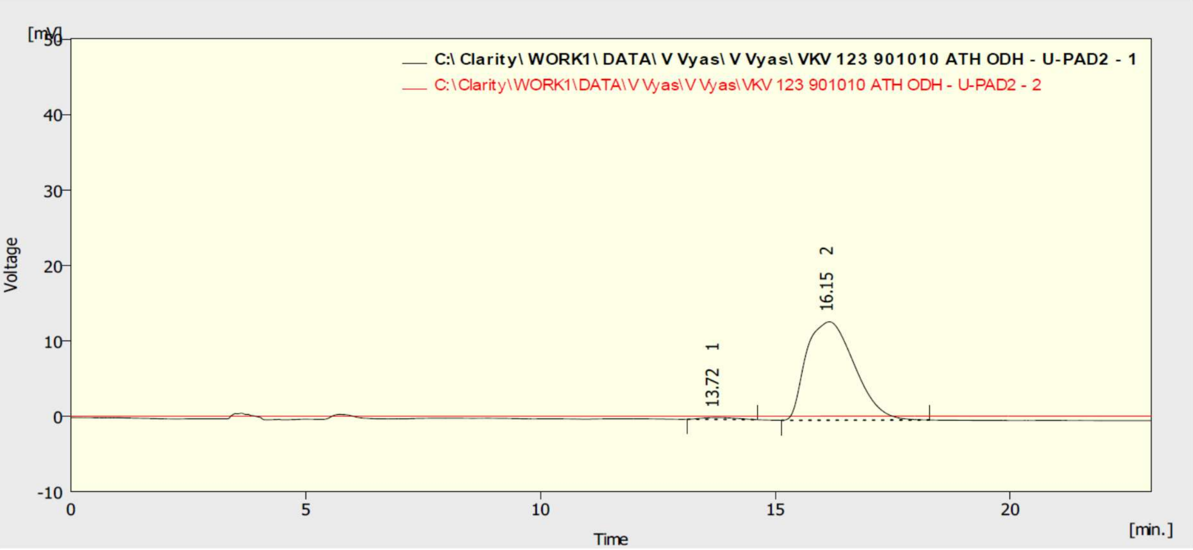

Result Table (Uncal - C:\Clarity\WORK1\DATA\V Vyas\V Vyas\VKV 123 901010 ATH ODH - U-PAD2 - 1)

	Reten. Time [min]	Area [mV.s]	Height [mV]	Area [%]	Height [%]	W05 [min]	Compound Name
1	13.724	13.022	0.250	1.4	1.9	0.86	
2	16.152	932.179	13.052	98.6	98.1	1.17	
	Total	945.201	13.302	100.0	100.0		

1-Cyclobutyl-2-(phenylsulfonyl)ethan-1-one 10c

^{1}H NMR (400 MHz, $CDCl_3$)

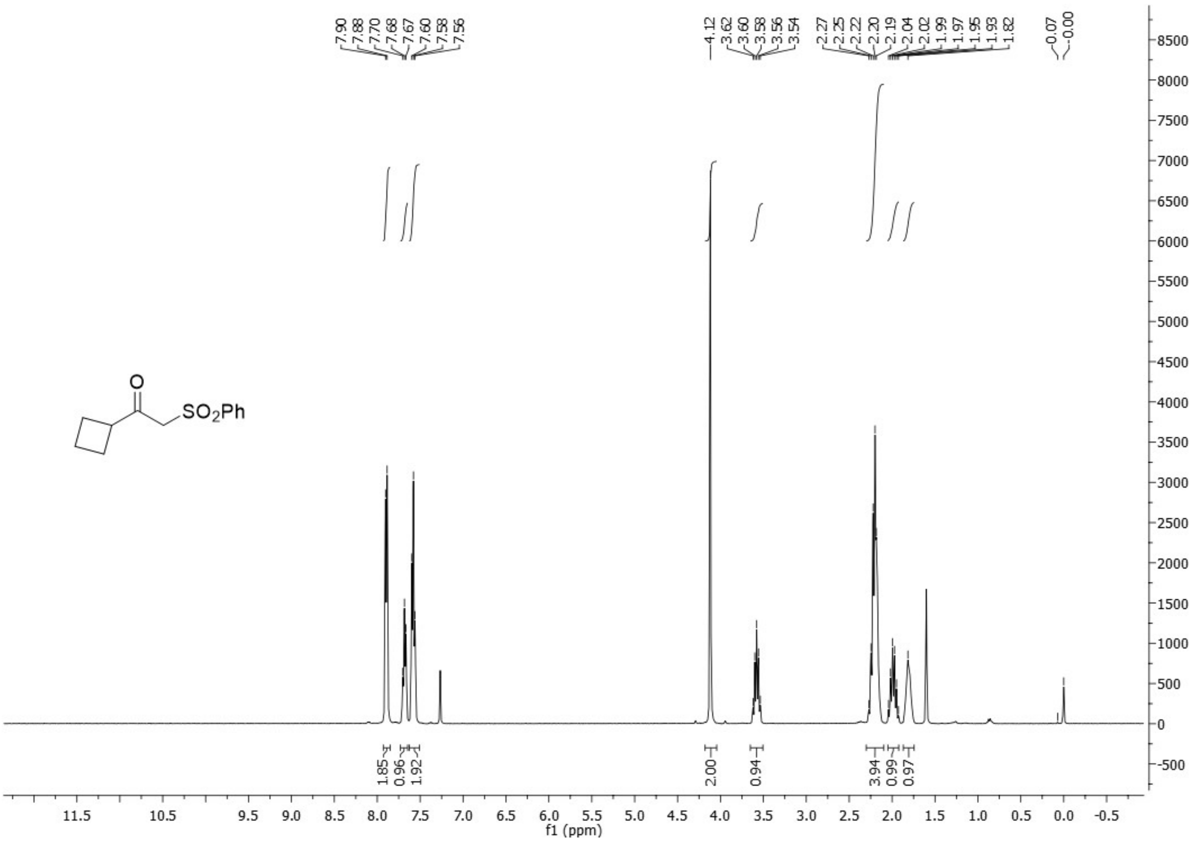

^{13}C NMR (101 MHz, $CDCl_3$

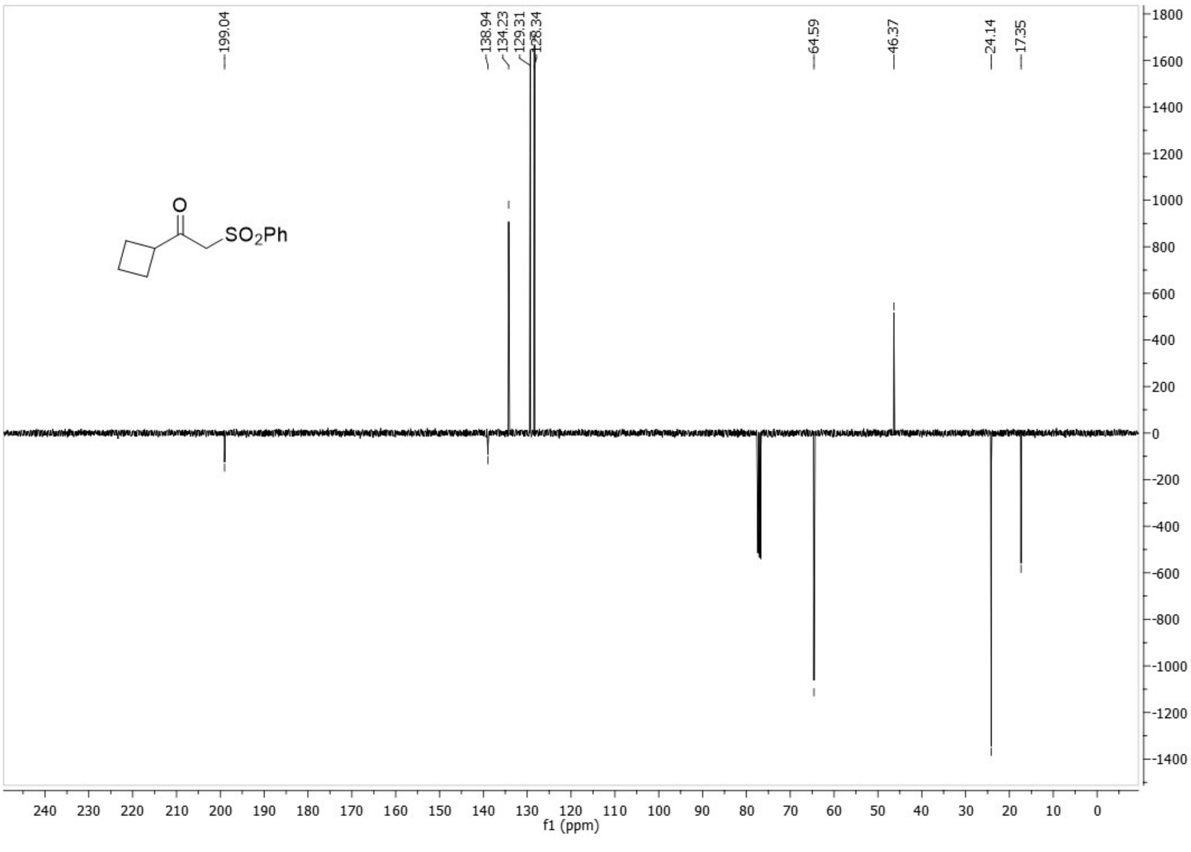

Ketone HPLC of 1-cyclobutyl-2-(phenylsulfonyl)ethan-1-one.

Clarity - Chromatography SW
DataApex 2006
www.dataapex.com

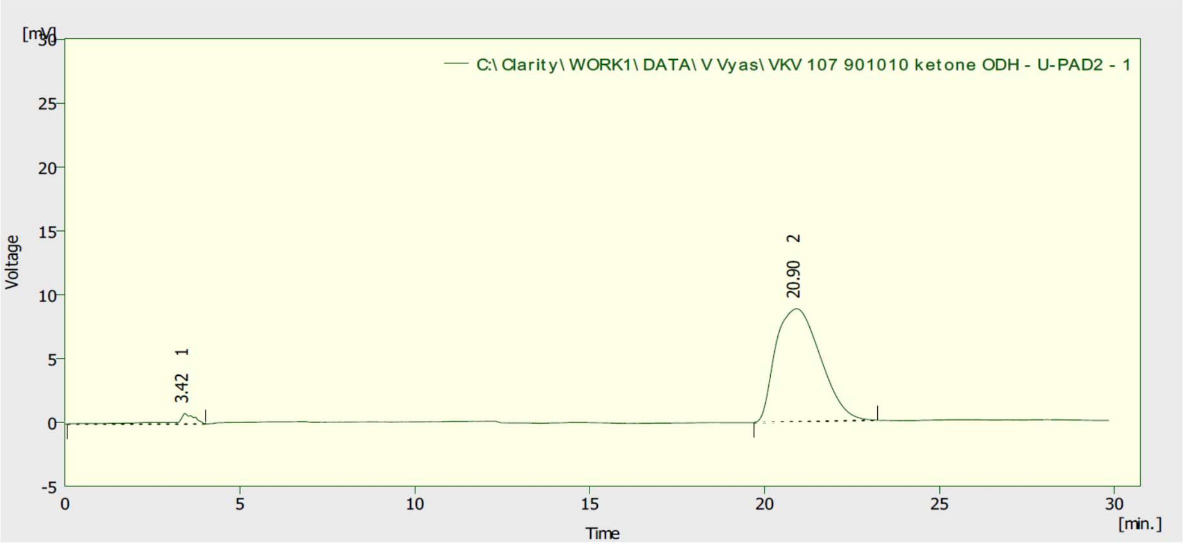

Result Table (Uncal - C:\Clarity\WORK1\DATA\V Vyas\VKV 107 901010 ketone ODH - U-PAD2 - 1)

	Reten. Time [min]	Area [mV.s]	Height [mV]	Area [%]	Height [%]	W05 [min]	Compound Name
1	3.420	37.542	0.836	4.5	8.6	0.45	
2	20.904	788.410	8.843	95.5	91.4	1.47	
	Total	825.952	9.679	100.0	100.0		

1-Cyclobutyl-2-(phenylsulfonyl)ethan-1-ol 11c

^{1}H NMR (400 MHz, $CDCl_3$)

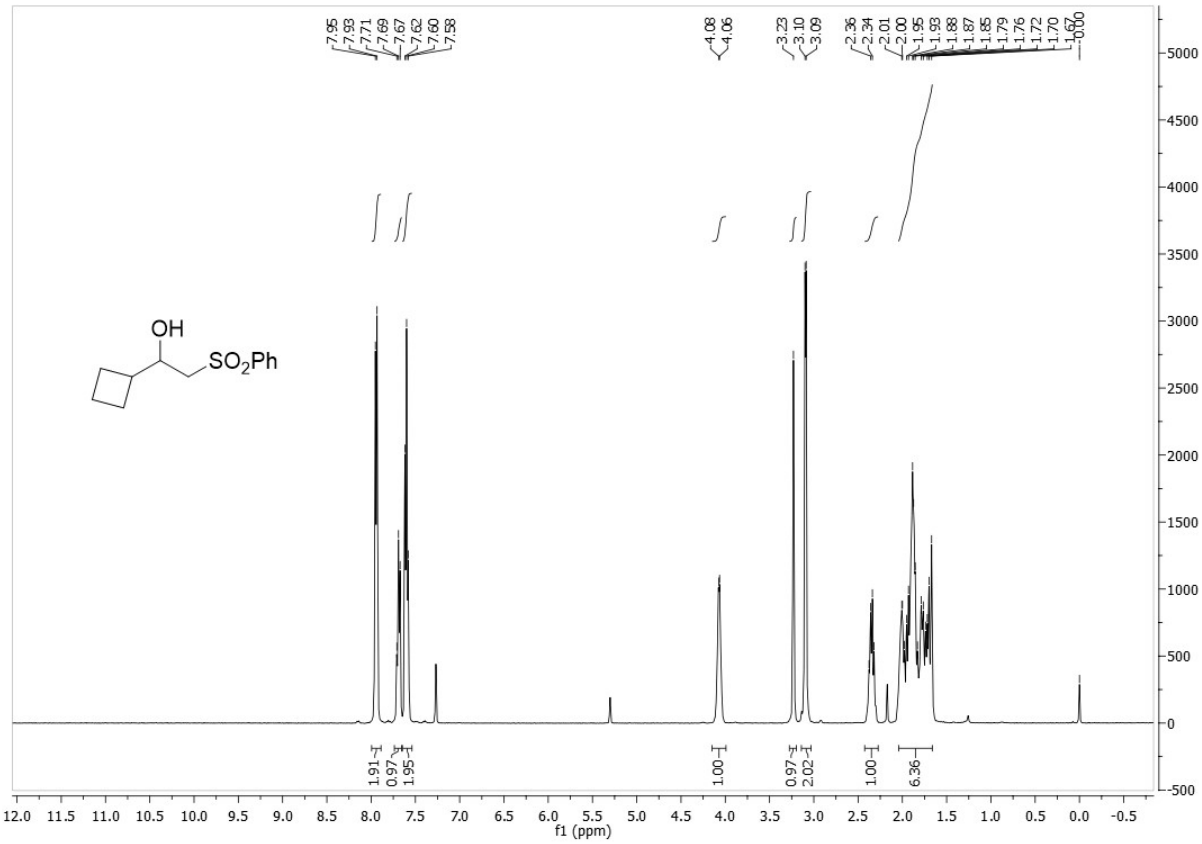

^{13}C NMR (101 MHz, $CDCl_3$

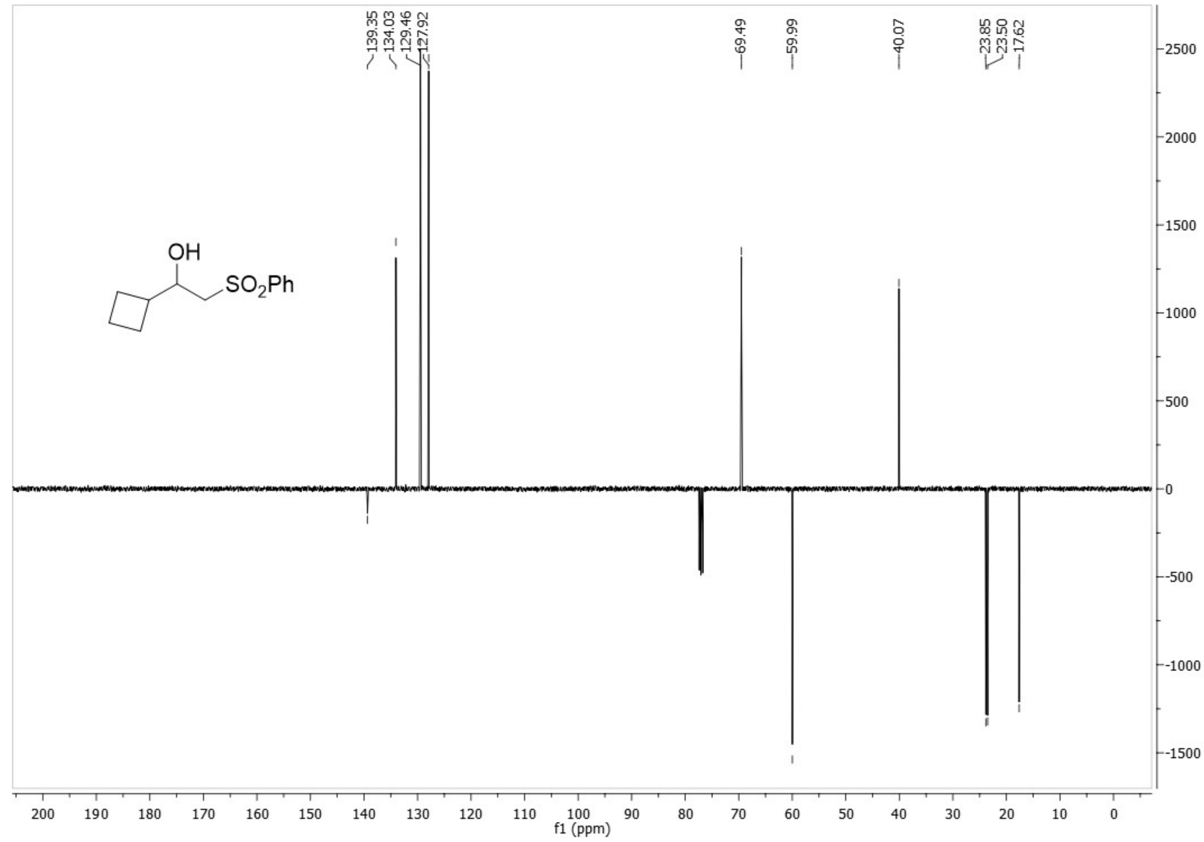

Racemic HPLC of 1-cyclobutyl-2-(phenylsulfonyl)ethan-1-ol.

Clarity - Chromatography SW
DataApex 2006
www.dataapex.com

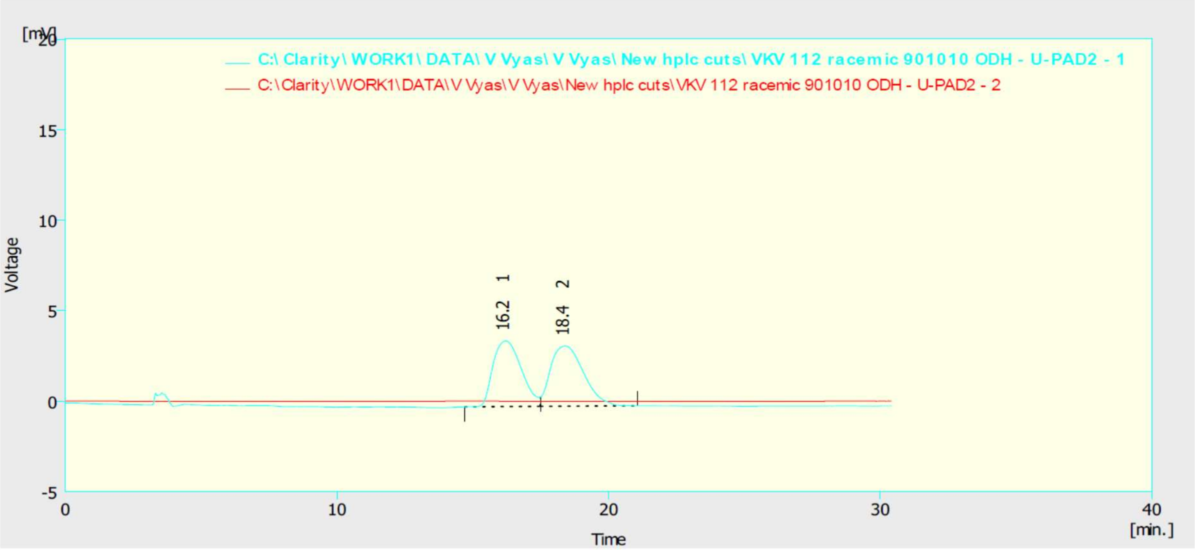

Result Table (Uncal - C:\Clarity\WORK1\DATA\V Vyas\V Vyas\New hplc cuts\VKV 112 racemic 901010 ODH - U-PAD2 - 1)

	Reten. Time [min]	Area [mV.s]	Height [mV]	Area [%]	Height [%]	W05 [min]	Compound Name
1	16.208	264.078	3.629	48.4	52.2	1.19	
2	18.416	281.338	3.330	51.6	47.8	1.36	
	Total	545.416	6.959	100.0	100.0		

HPLC after ATH 1-cyclobutyl-2-(phenylsulfonyl)ethan-1-ol. (100% conversion, 98.4% ee).

Clarity - Chromatography SW
DataApex 2006
www.dataapex.com

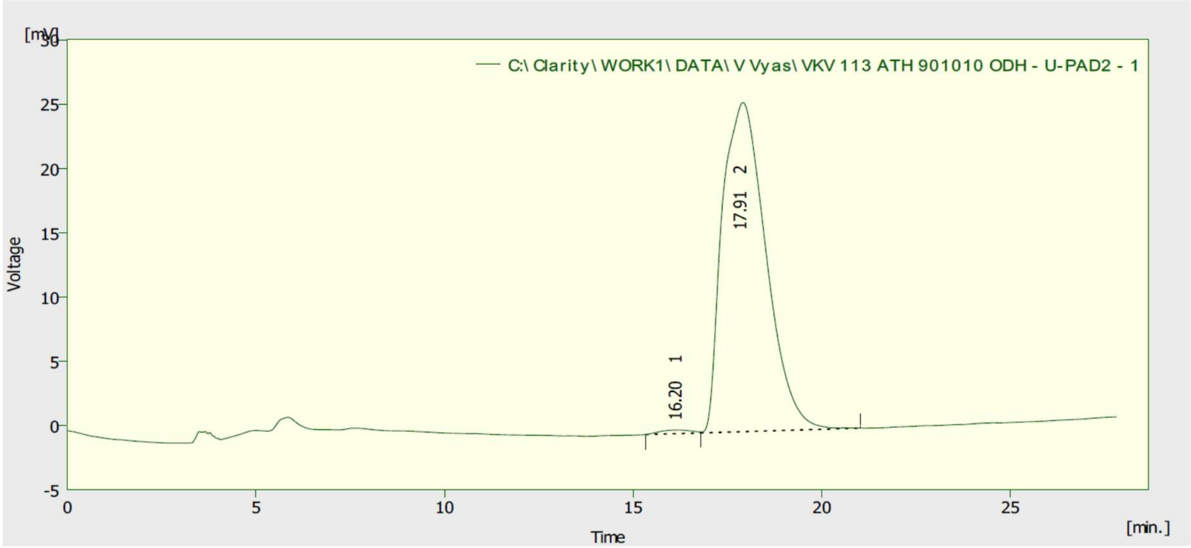

Result Table (Uncal - C:\Clarity\WORK1\DATA\V Vyas\VKV 113 ATH 901010 ODH - U-PAD2 - 1)

	Reten. Time [min]	Area [mV.s]	Height [mV]	Area [%]	Height [%]	W05 [min]	Compound Name
1	16.196	16.347	0.282	0.8	1.1	1.03	
2	17.912	2058.177	25.602	99.2	98.9	1.31	
	Total	2074.524	25.884	100.0	100.0		

1-Cyclopropyl-2-(phenylsulfonyl)ethan-1-one 10d

^{1}H NMR (400 MHz, $CDCl_3$)

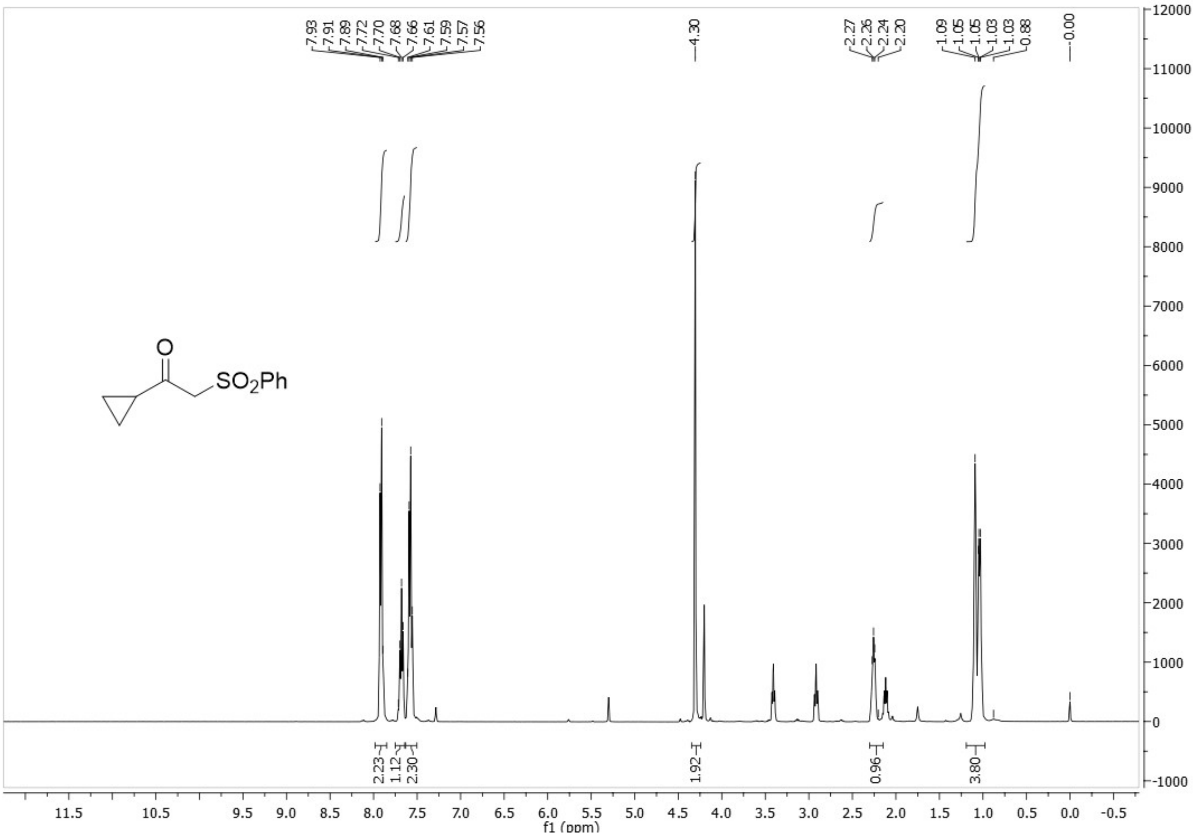

^{13}C NMR (101 MHz, $CDCl_3$

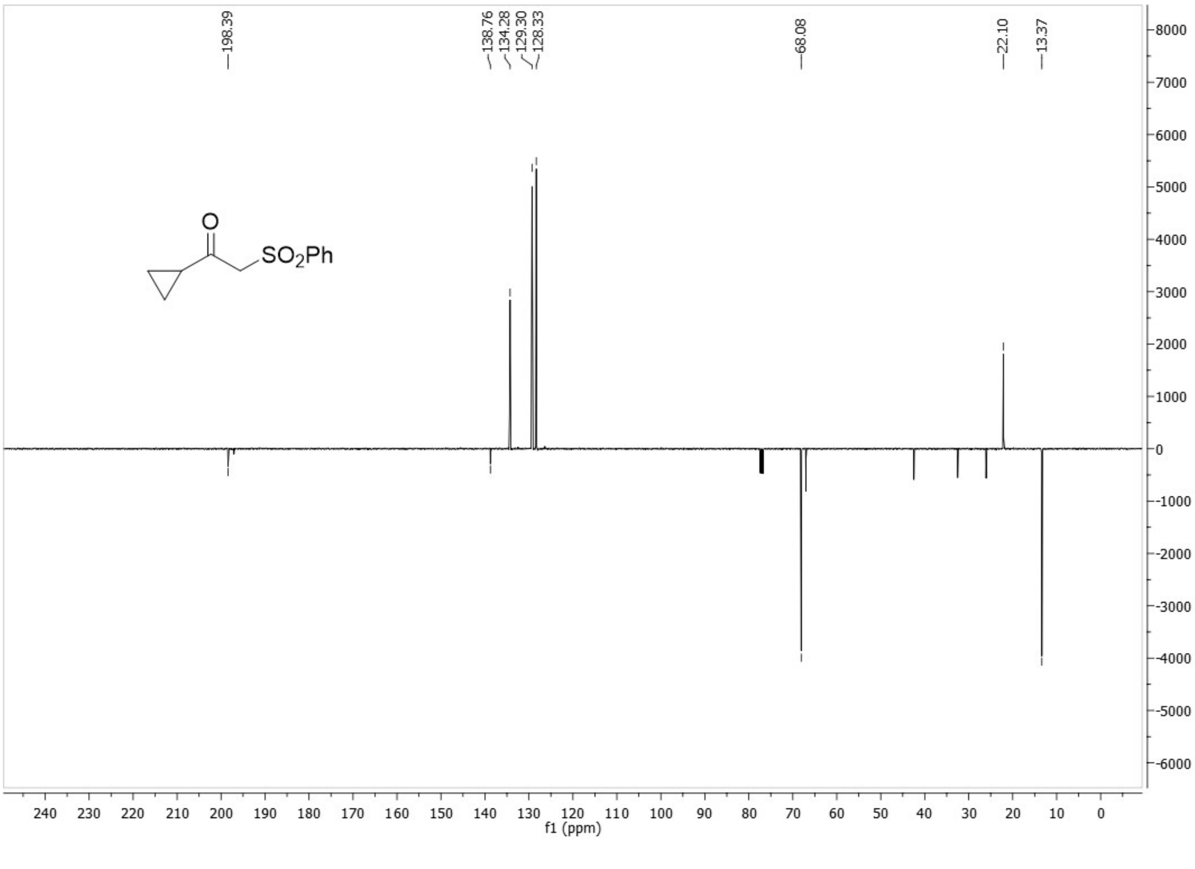

Ketone HPLC of 1-cyclopropyl-2-(phenylsulfonyl)ethan-1-one.

Clarity - Chromatography SW
DataApex 2006
www.dataapex.com

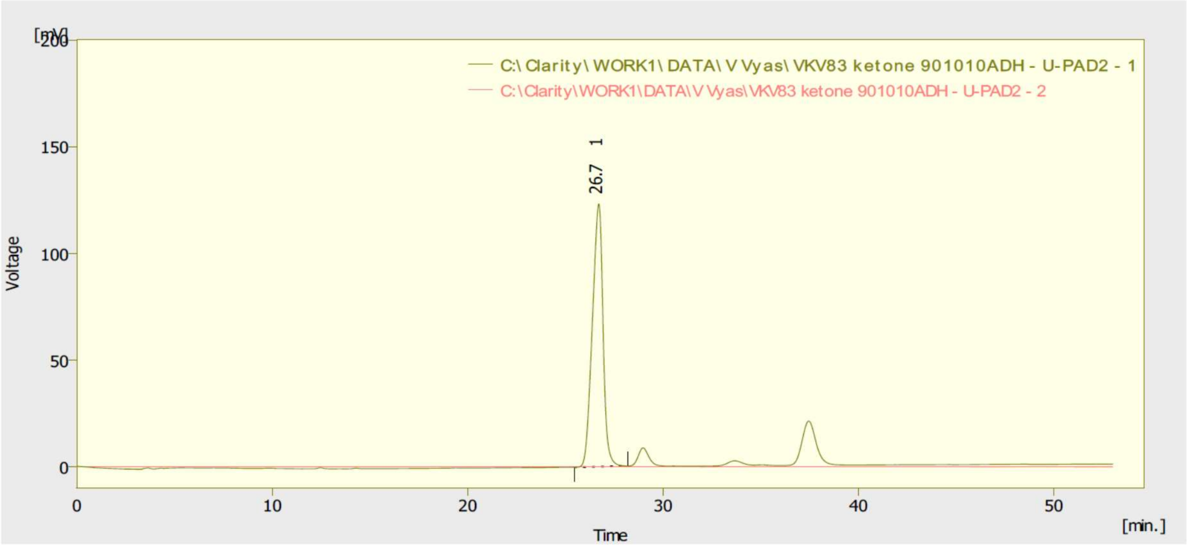

Result Table (Uncal - C:\Clarity\WORK1\DATA\V Vyas\VKV83 ketone 901010ADH - U-PAD2 - 1)

	Reten. Time [min]	Area [mV.s]	Height [mV]	Area [%]	Height [%]	W05 [min]	Compound Name
1	26.712	4672.059	123.126	100.0	100.0	0.59	
	Total	4672.059	123.126	100.0	100.0		

1-Cyclopropyl-2-(phenylsulfonyl)ethan-1-ol 11d
^{1}H NMR (400 MHz, $CDCl_3$)

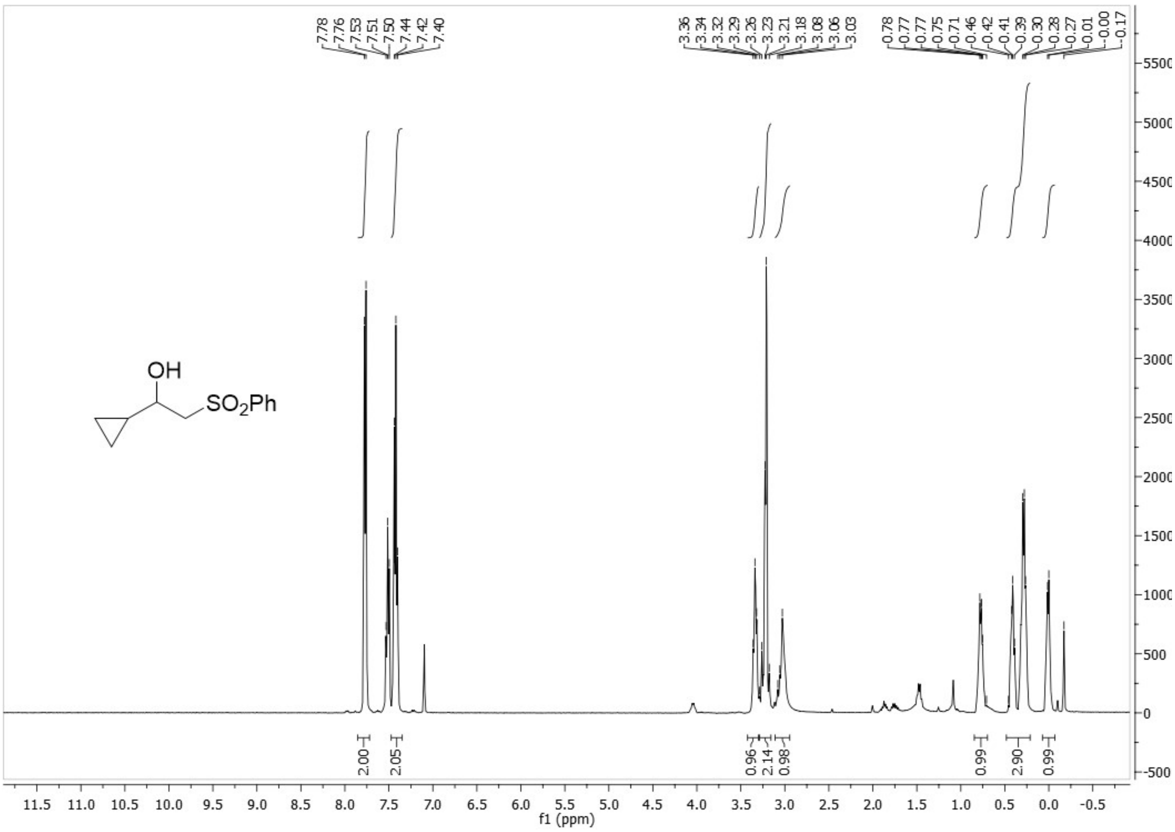

^{13}C NMR (101 MHz, $CDCl_3$

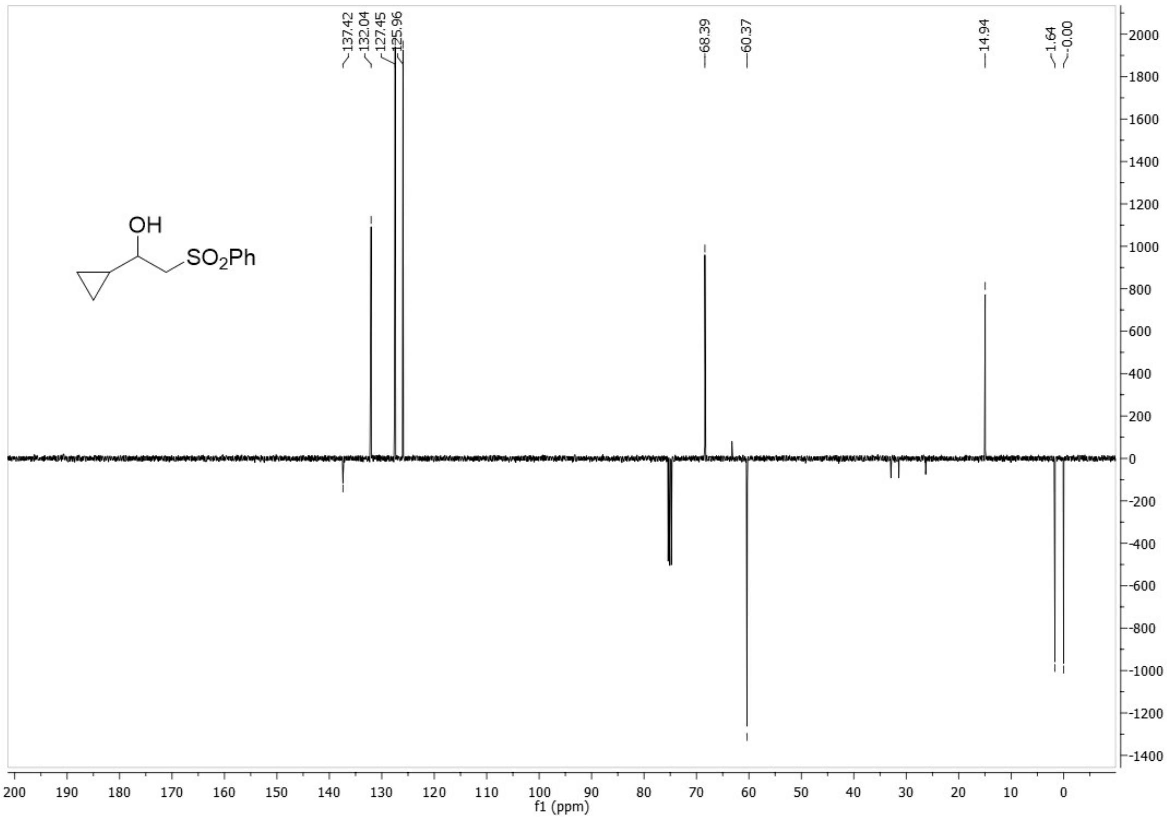

Racemic HPLC of 1-cyclopropyl-2-(phenylsulfonyl)ethan-1-ol.

Clarity - Chromatography SW
DataApex 2006
www.dataapex.com

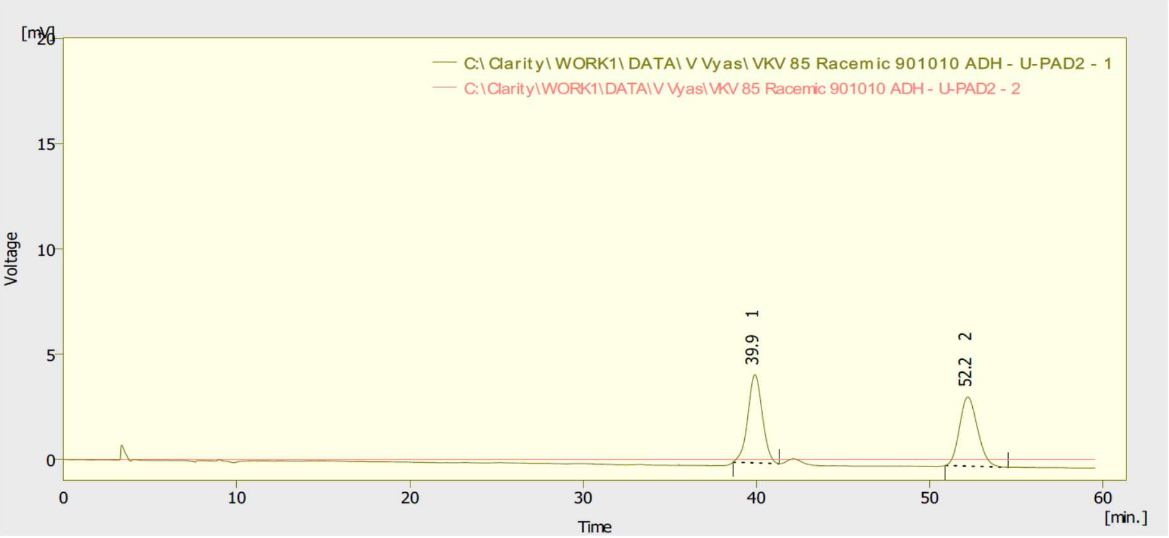

Result Table (Uncal - C:\Clarity\WORK1\DATA\V Vyas\VKV 85 Racemic 901010 ADH - U-PAD2 - 1)

	Reten. Time [min]	Area [mV.s]	Height [mV]	Area [%]	Height [%]	W05 [min]	Compound Name
1	39.912	244.349	4.193	50.7	56.1	0.89	
2	52.200	238.021	3.285	49.3	43.9	1.13	
	Total	482.370	7.477	100.0	100.0		

HPLC after ATH 1-cyclopropyl-2-(phenylsulfonyl)ethan-1-ol. (100% conversion, 99.2% ee).

Clarity - Chromatography SW
DataApex 2006
www.dataapex.com

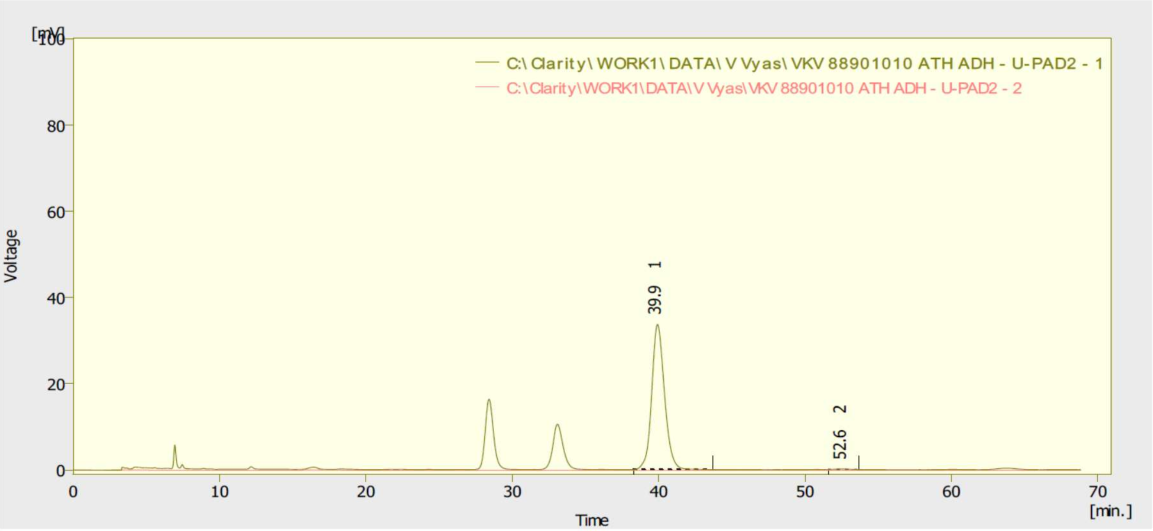

Result Table (Uncal - C:\Clarity\WORK1\DATA\V Vyas\VKV 88901010 ATH ADH - U-PAD2 - 1)

	Reten. Time [min]	Area [mV.s]	Height [mV]	Area [%]	Height [%]	W05 [min]	Compound Name
1	39.920	2045.149	33.614	99.6	99.6	0.91	
2	52.576	7.688	0.125	0.4	0.4	1.00	
	Total	2052.837	33.739	100.0	100.0		

1-(Phenylsulfonyl)octan-2-one 10e

^{1}H NMR (400 MHz, $CDCl_3$)

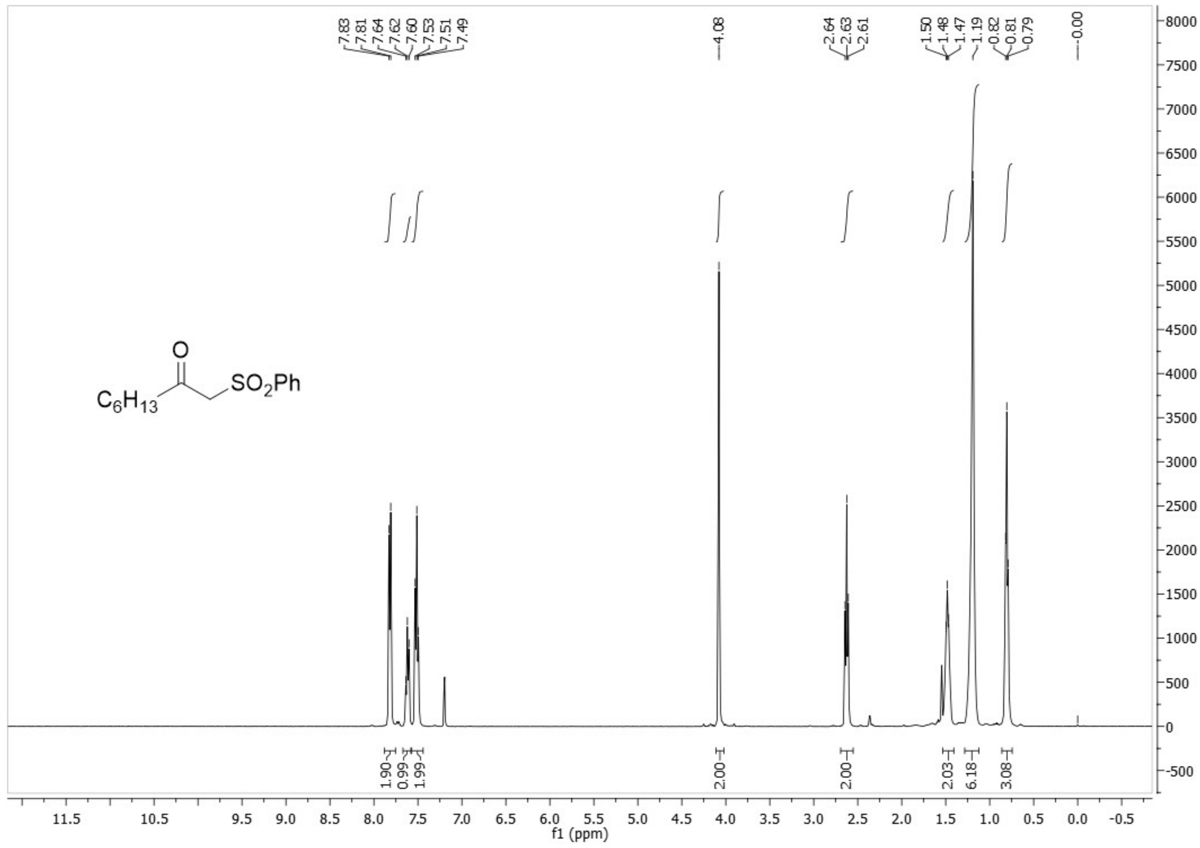

^{13}C NMR (101 MHz, $CDCl_3$

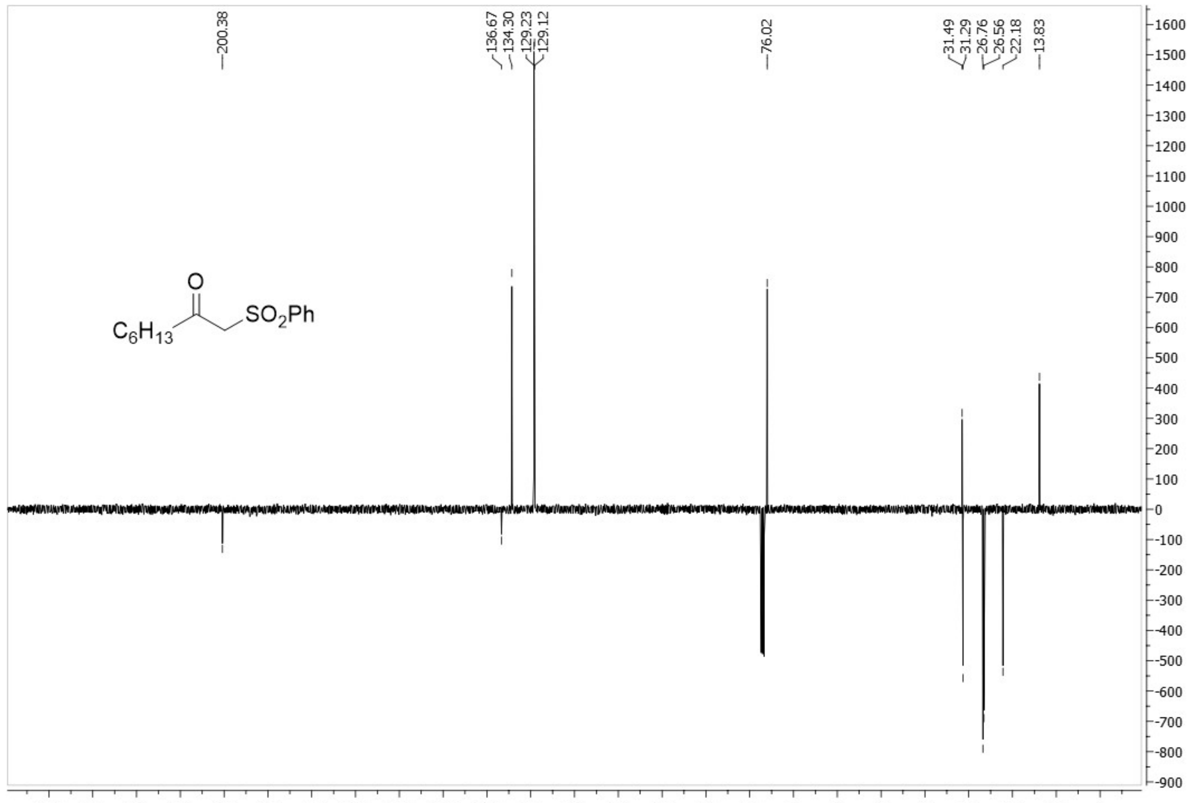

Ketone HPLC of 1-(phenylsulfonyl)octan-2-one.

Clarity - Chromatography SW
DataApex 2006
www.dataapex.com

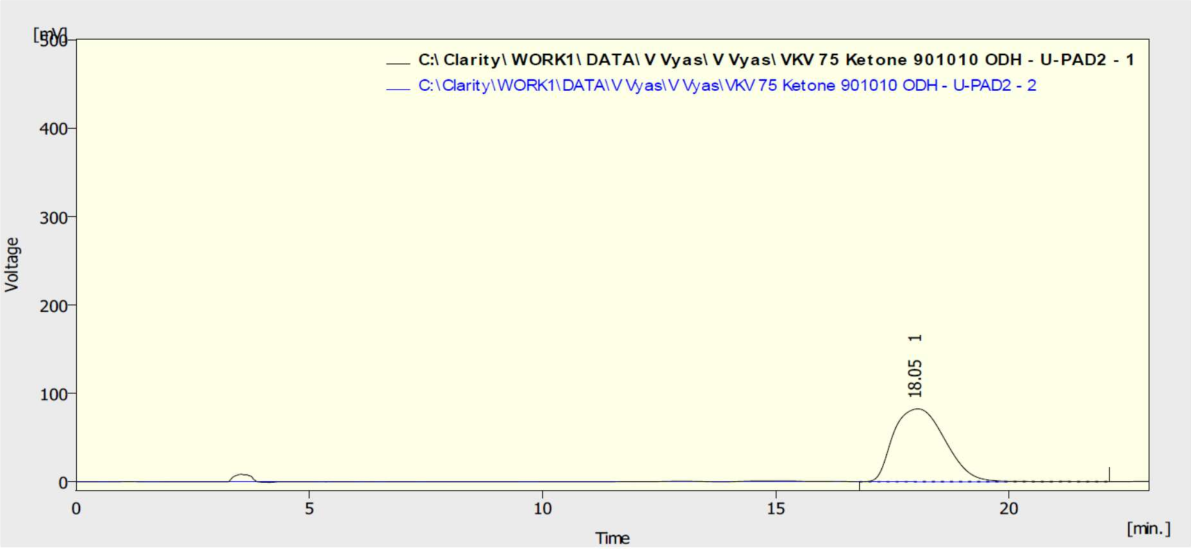

Result Table (Uncal - C:\Clarity\WORK1\DATA\V Vyas\V Vyas\VKV 75 Ketone 901010 ODH - U-PAD2 - 1)

	Reten. Time [min]	Area [mV.s]	Height [mV]	Area [%]	Height [%]	W05 [min]	Compound Name
1	18.048	6337.211	82.327	100.0	100.0	1.26	
	Total	6337.211	82.327	100.0	100.0		

1-(Phenylsulfonyl)octan-2-ol 11e

^{1}H NMR (400 MHz, $CDCl_3$)

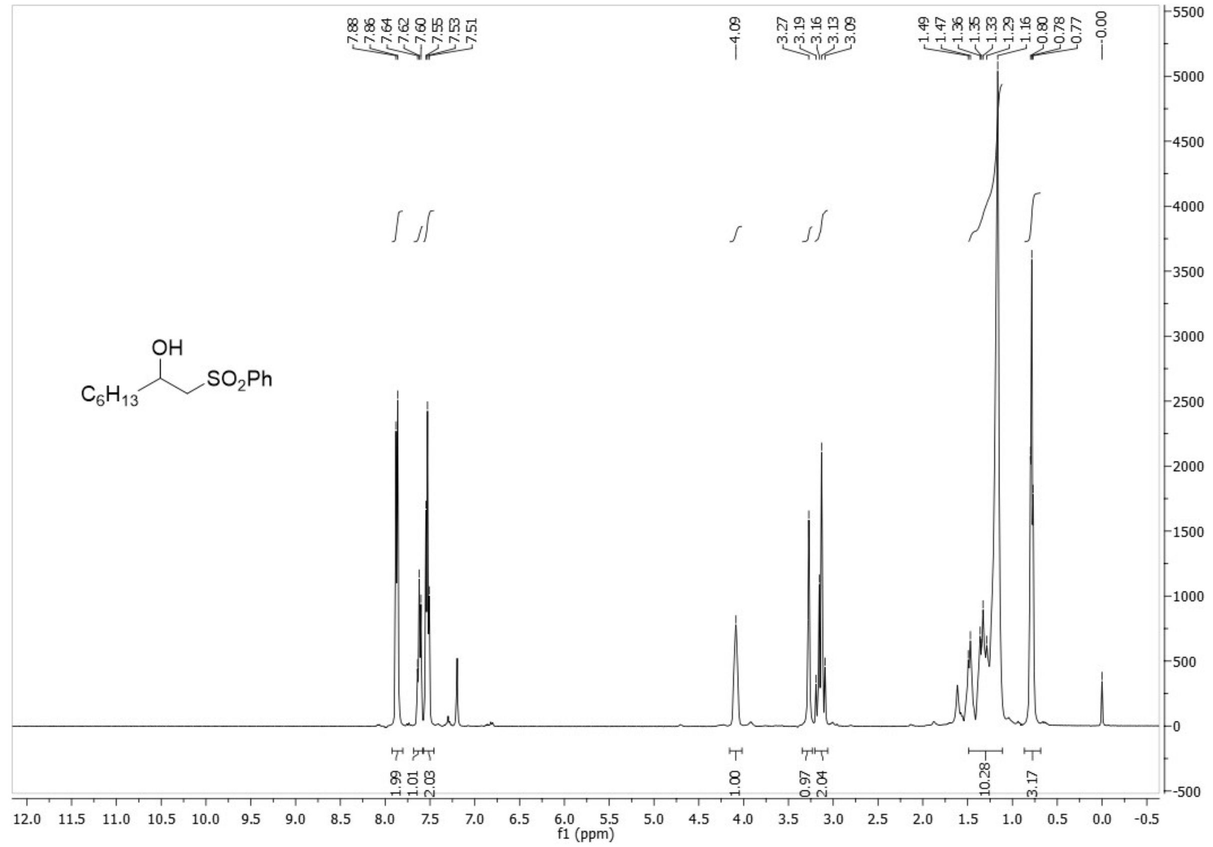

^{13}C NMR (101 MHz, $CDCl_3$

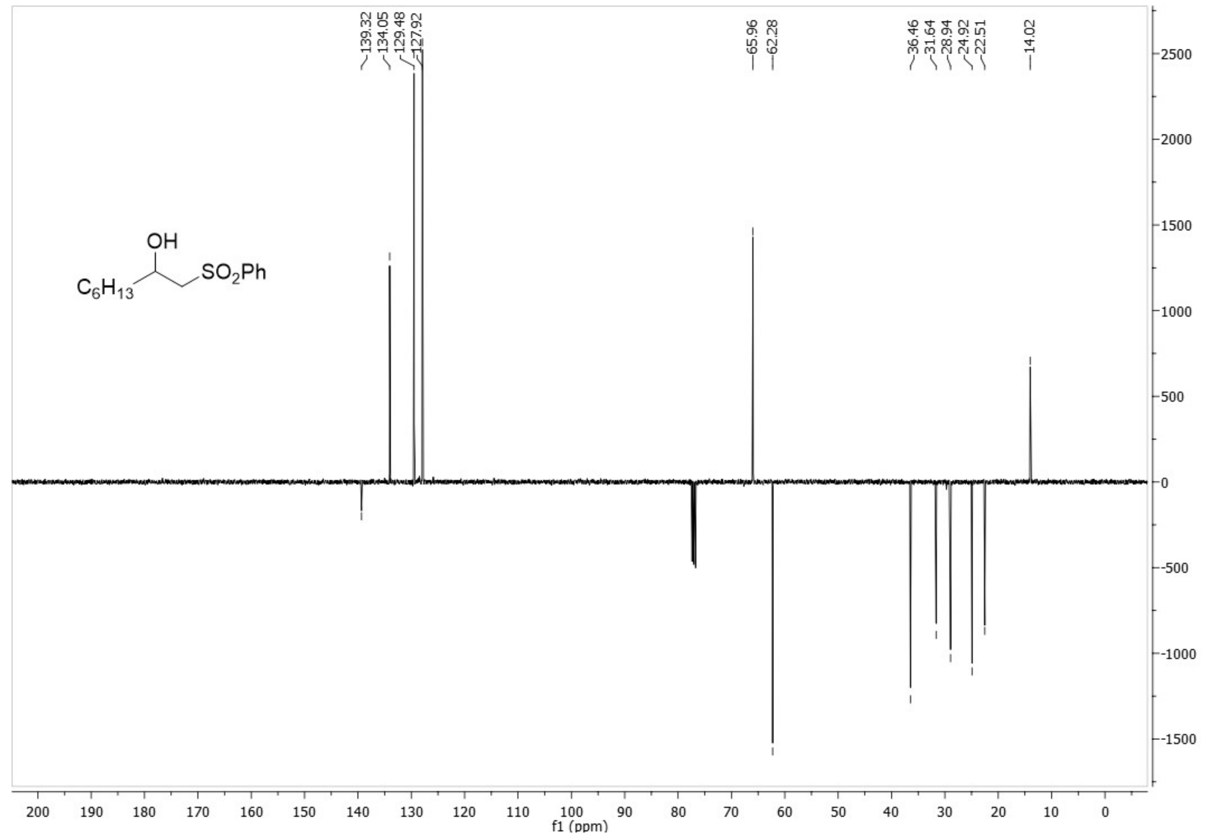

Racemic HPLC of 1-(phenylsulfonyl)octan-2-ol.

Clarity - Chromatography SW
DataApex 2006
www.dataapex.com

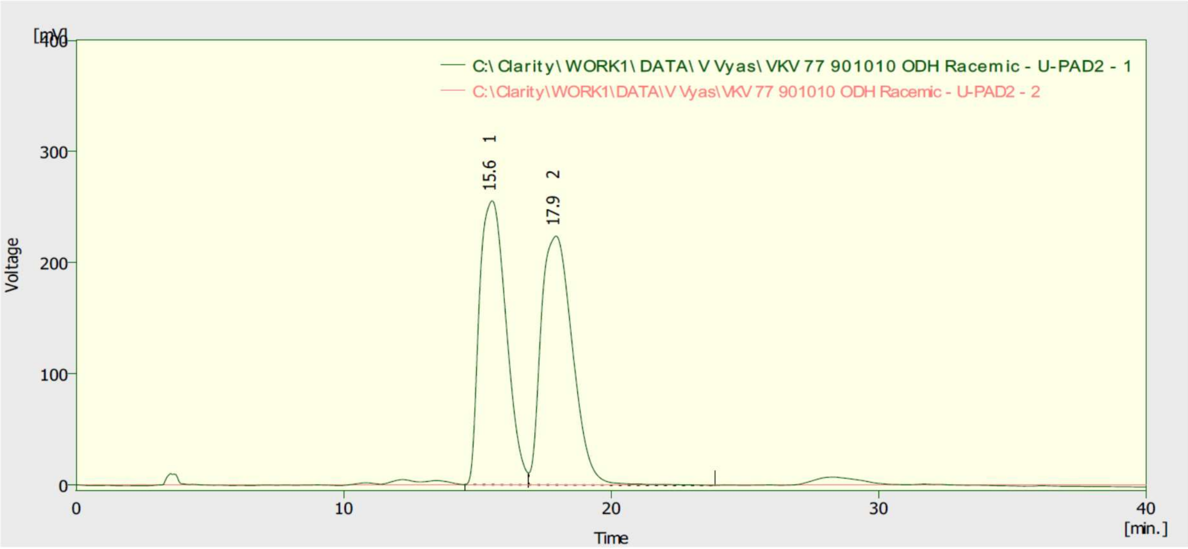

Result Table (Uncal - C:\Clarity\WORK1\DATA\V Vyas\VKV 77 901010 ODH Racemic - U-PAD2 - 1)

	Reten. Time [min]	Area [mV.s]	Height [mV]	Area [%]	Height [%]	W05 [min]	Compound Name
1	15.552	17715.872	255.365	49.1	53.3	1.13	
2	17.936	18334.797	223.814	50.9	46.7	1.31	
	Total	36050.669	479.179	100.0	100.0		

HPLC after ATH 1-(phenylsulfonyl)octan-2-ol. (100% conversion, 91% ee).

Clarity - Chromatography SW
DataApex 2006
www.dataapex.com

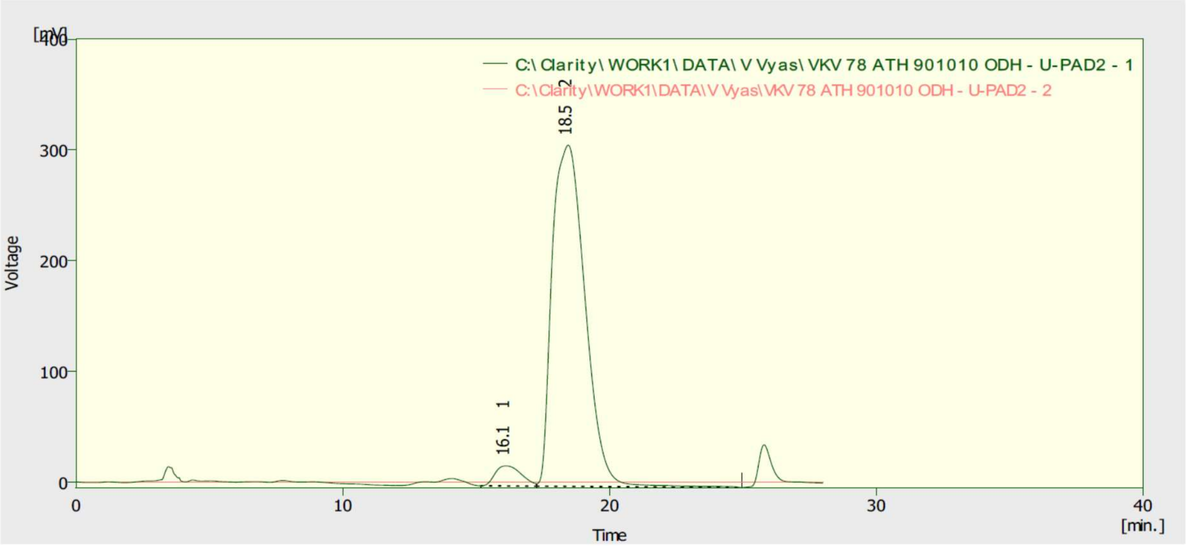

Result Table (Uncal - C:\Clarity\WORK1\DATA\V Vyas\VKV 78 ATH 901010 ODH - U-PAD2 - 1)

	Reten. Time [min]	Area [mV.s]	Height [mV]	Area [%]	Height [%]	W05 [min]	Compound Name
1	16.116	1243.858	18.167	4.5	5.6	1.12	
2	18.452	26446.789	307.968	95.5	94.4	1.38	
	Total	27690.647	326.135	100.0	100.0		

1-(Phenylsulfonyl)pentan-2-one 10f

^{1}**H NMR** (400 MHz, $CDCl_3$)

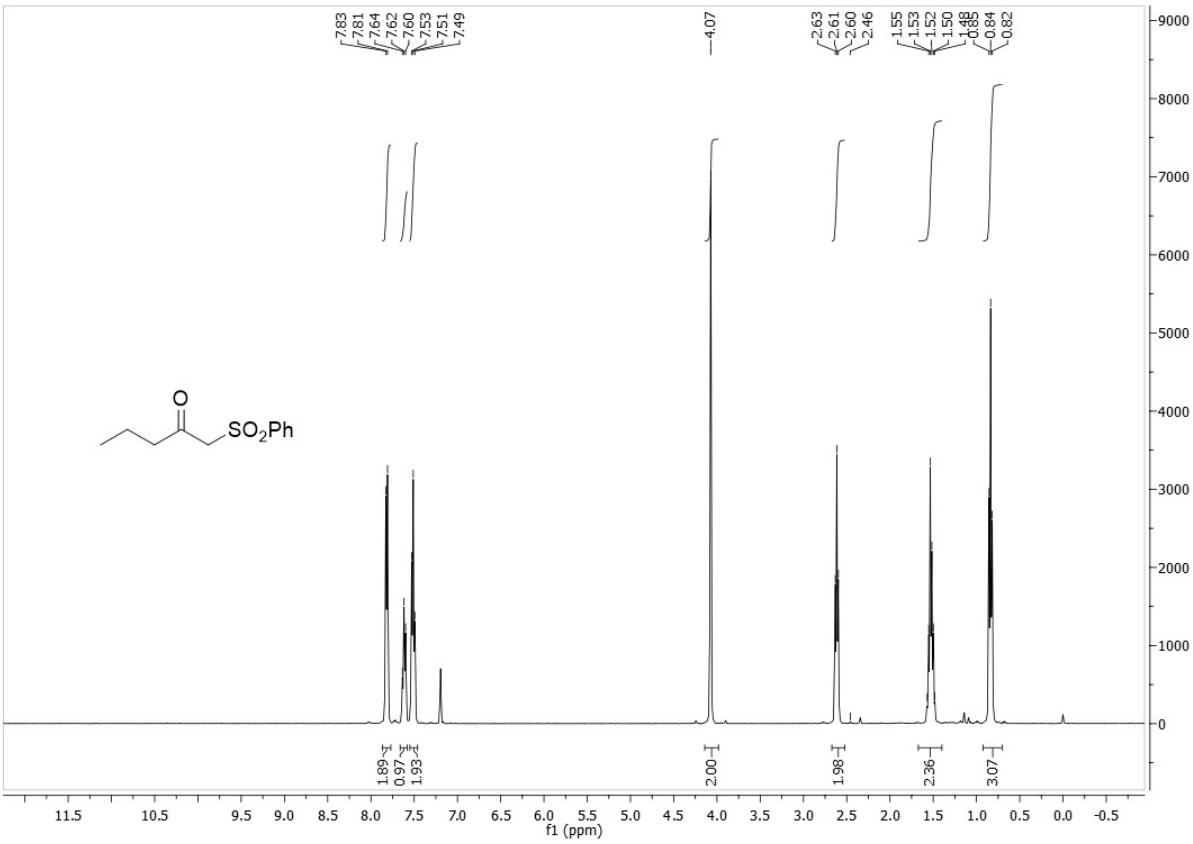

^{13}**C NMR** (101 MHz, $CDCl_3$

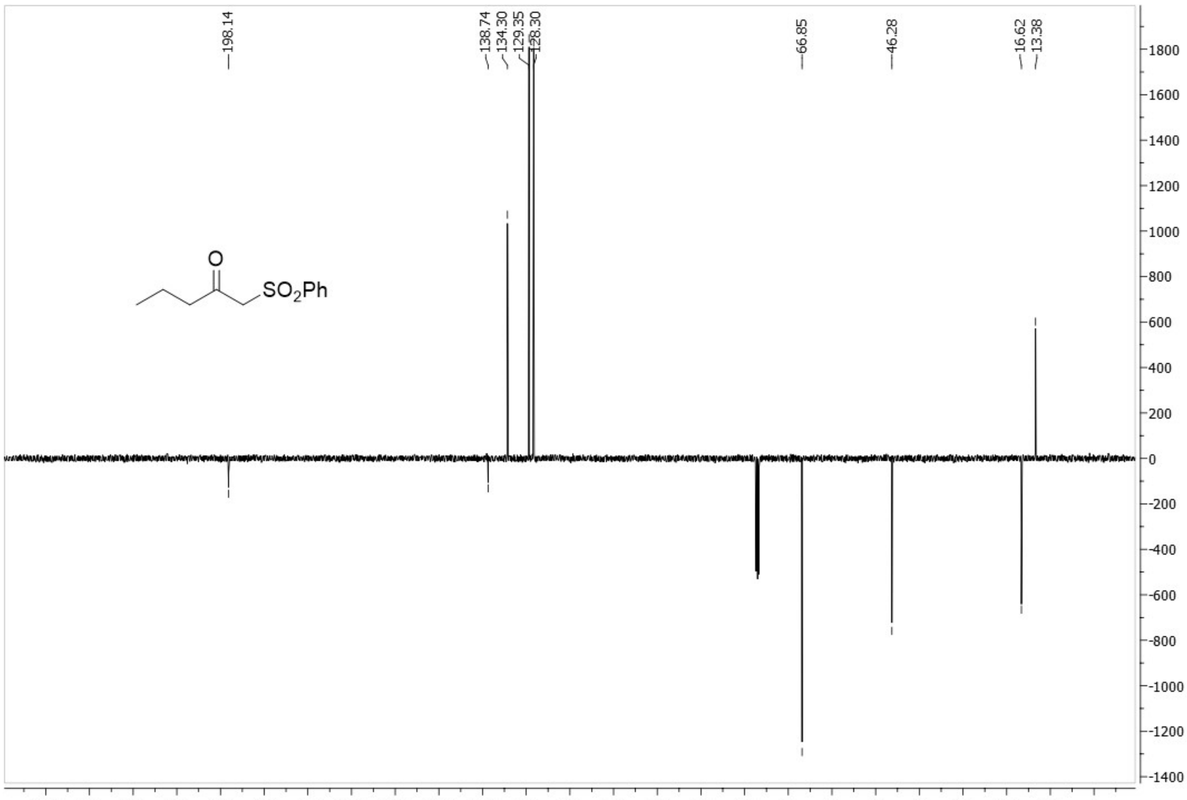

Ketone HPLC of 1-(phenylsulfonyl)pentan-2-one.

Clarity - Chromatography SW
DataApex 2006
www.dataapex.com

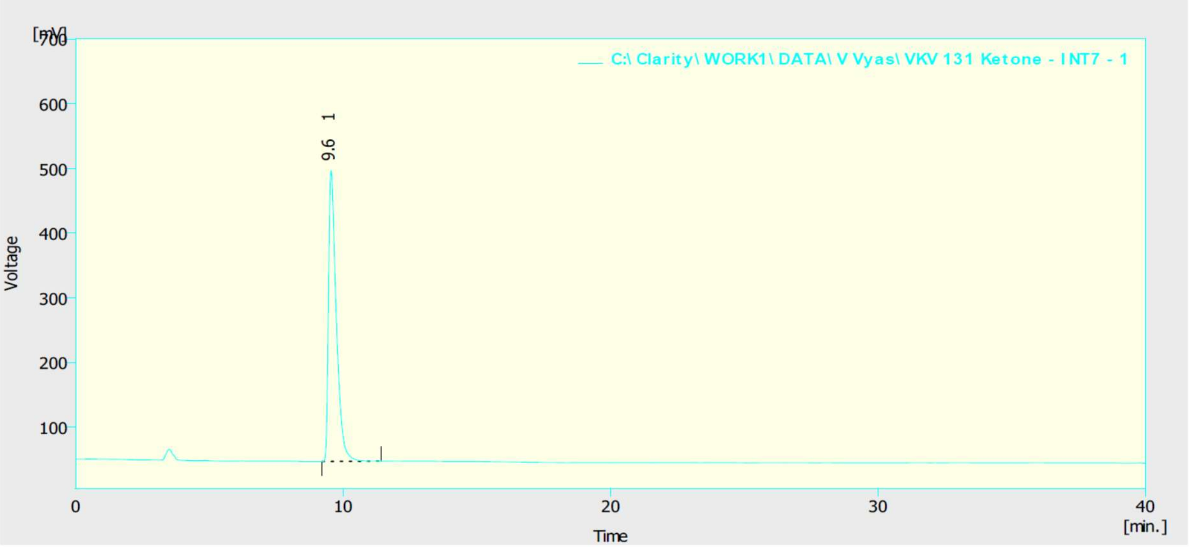

Result Table (Uncal - C:\Clarity\WORK1\DATA\V Vyas\VKV 131 Ketone - INT7 - 1)

	Reten. Time [min]	Area [mV.s]	Height [mV]	Area [%]	Height [%]	W05 [min]	Compound Name
1	9.557	9137.619	449.492	100.0	100.0	0.30	
	Total	9137.619	449.492	100.0	100.0		

1-(Phenylsulfonyl)pentan-2-ol 11f

^{1}H NMR (400 MHz, $CDCl_3$)

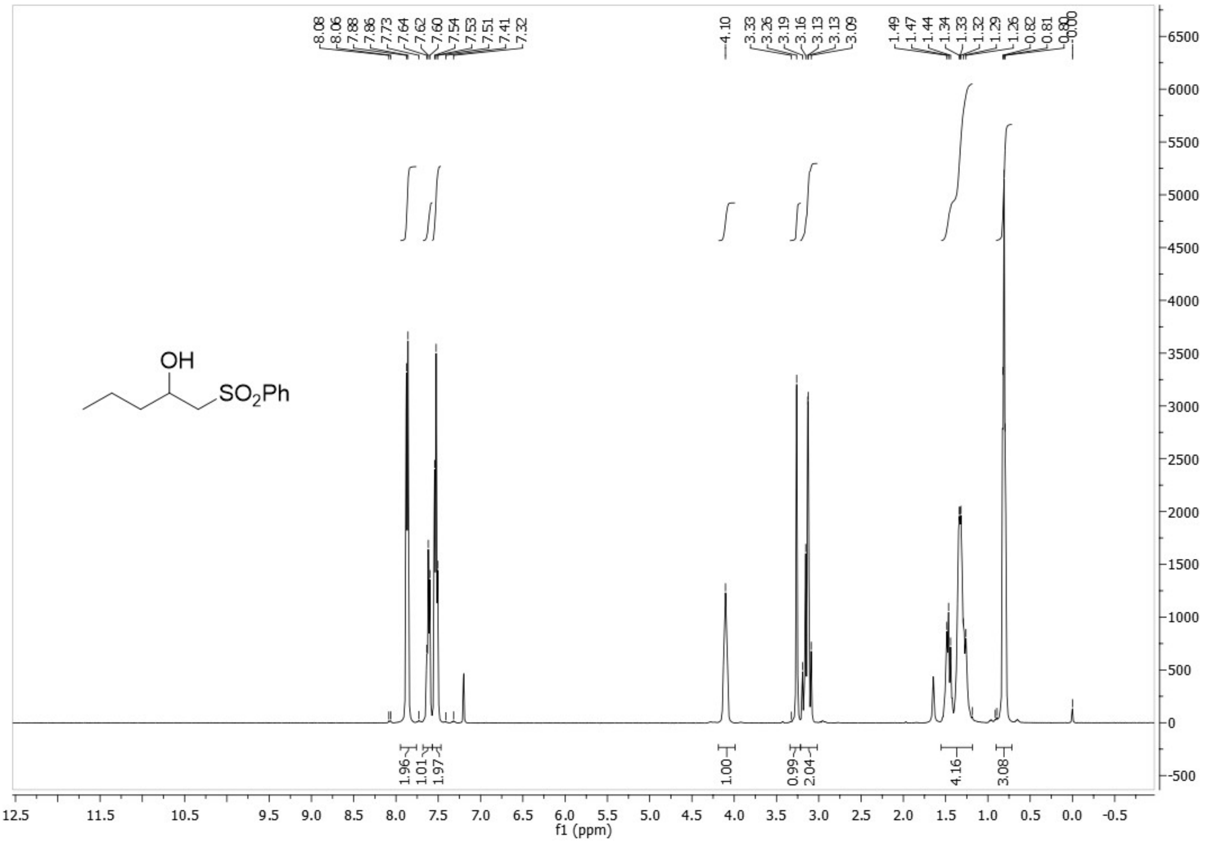

^{13}C NMR (101 MHz, $CDCl_3$

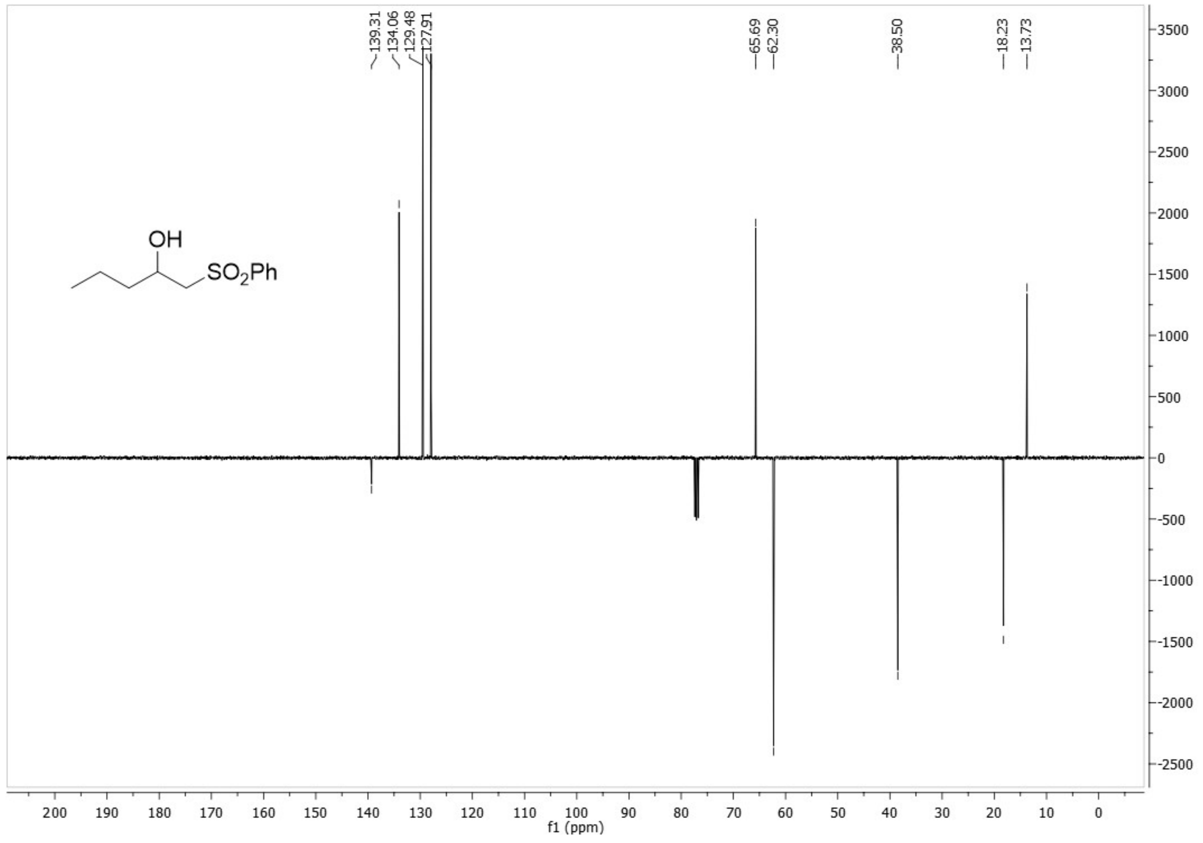

Racemic HPLC of 1-(phenylsulfonyl)pentan-2-ol.

Clarity - Chromatography SW
DataApex 2006
www.dataapex.com

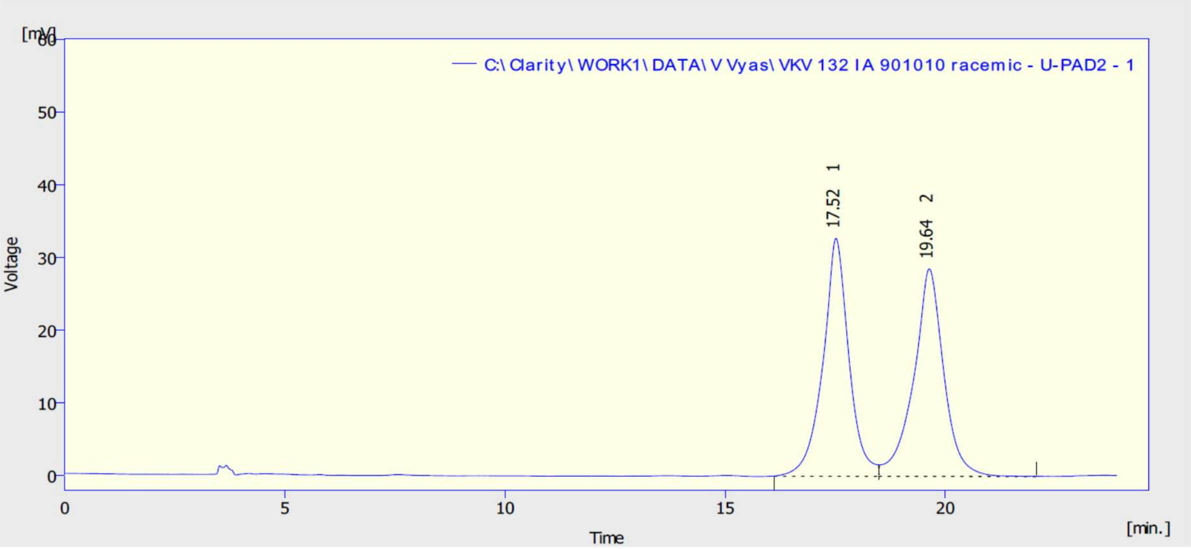

Result Table (Uncal - C:\Clarity\WORK1\DATA\V Vyas\VKV 132 IA 901010 racemic - U-PAD2 - 1)

	Reten. Time [min]	Area [mV.s]	Height [mV]	Area [%]	Height [%]	W05 [min]	Compound Name
1	17.516	1337.787	32.662	49.8	53.4	0.58	
2	19.636	1350.848	28.499	50.2	46.6	0.67	
	Total	2688.635	61.161	100.0	100.0		

HPLC after ATH 1-(phenylsulfonyl)pentan-2-ol. (100% conversion, 90.4% ee).

Clarity - Chromatography SW
DataApex 2006
www.dataapex.com

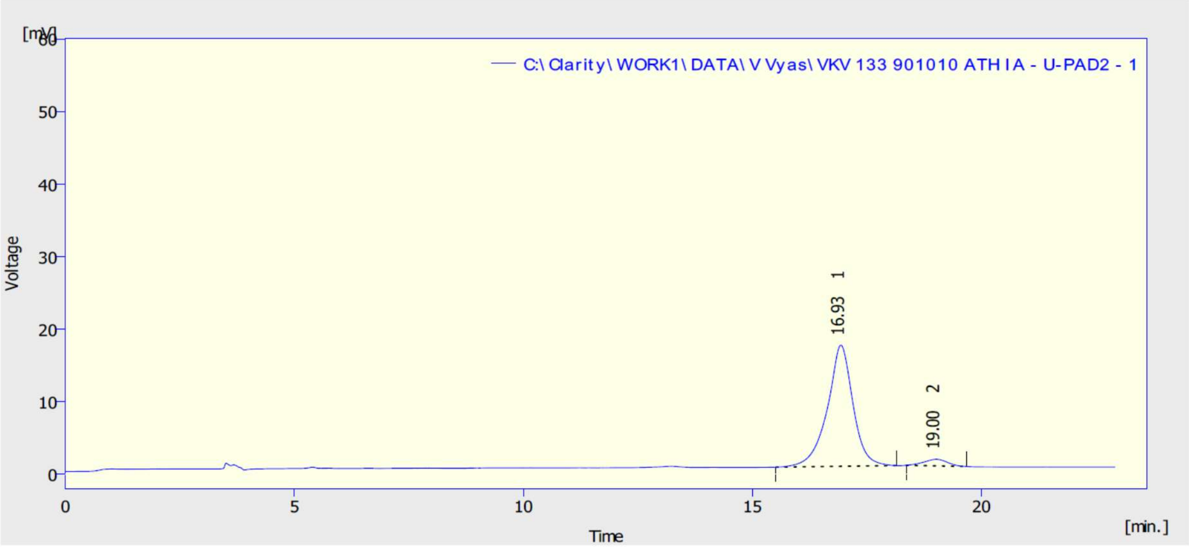

Result Table (Uncal - C:\Clarity\WORK1\DATA\V Vyas\VKV 133 901010 ATH IA - U-PAD2 - 1)

	Reten. Time [min]	Area [mV.s]	Height [mV]	Area [%]	Height [%]	W05 [min]	Compound Name
1	16.928	650.879	16.700	95.2	94.8	0.56	
2	19.004	32.473	0.908	4.8	5.2	0.57	
	Total	683.352	17.608	100.0	100.0		

3,3-Dimethyl-1-(phenylsulfonyl)butan-2-one 10g

^{1}H NMR (400 MHz, $CDCl_3$)

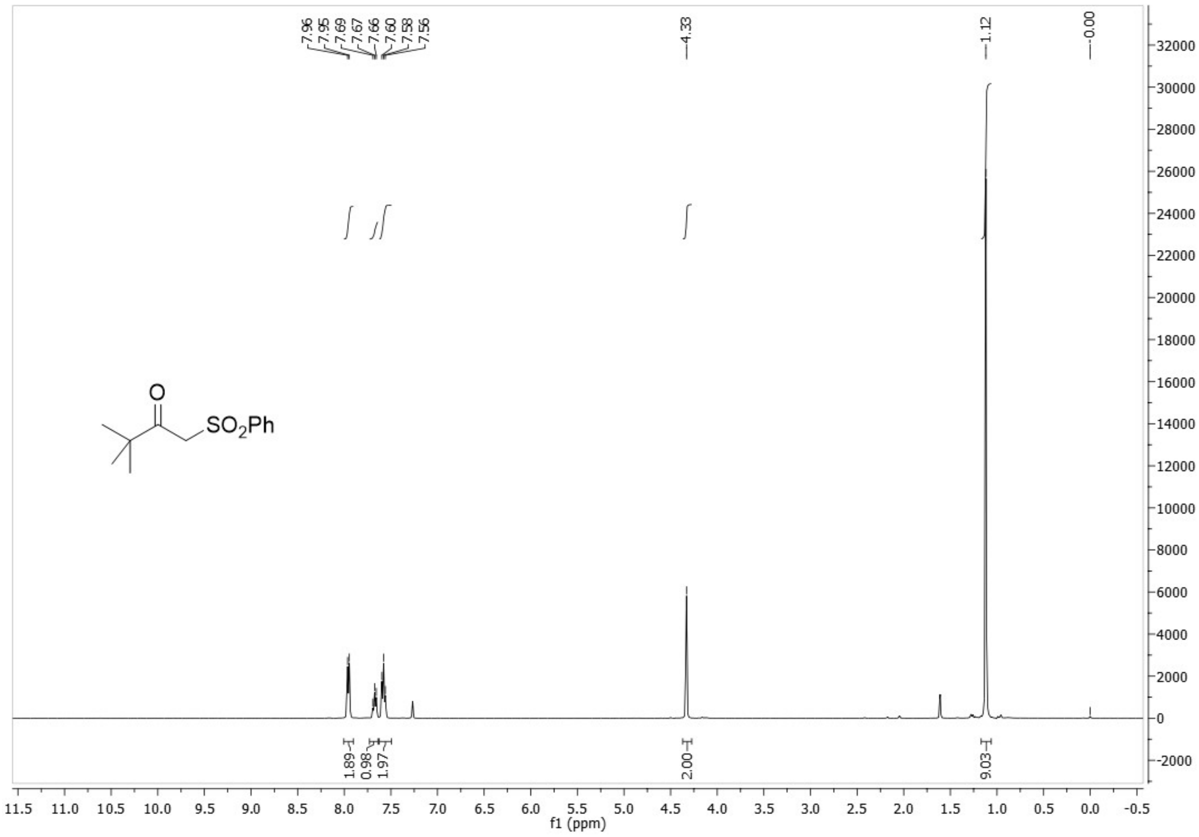

^{13}C NMR (101 MHz, $CDCl_3$

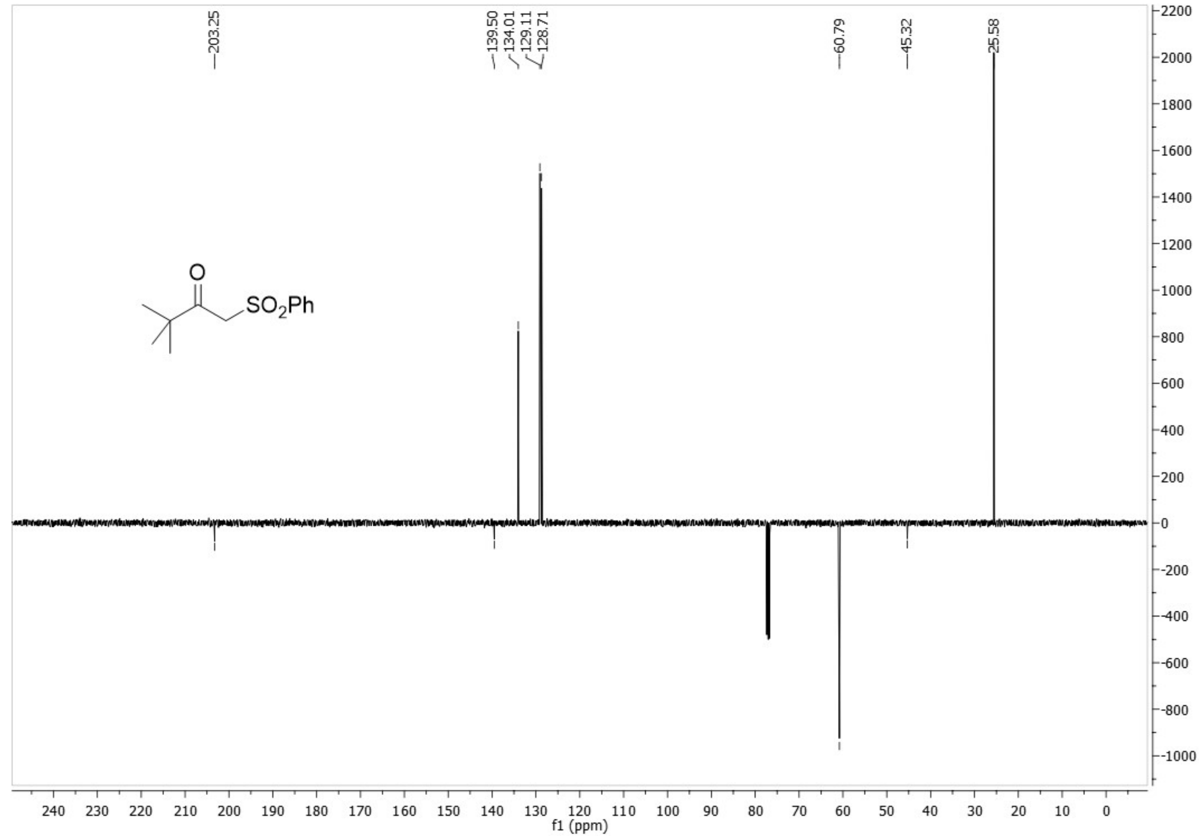

Ketone HPLC of 3,3-dimethyl-1-(phenylsulfonyl)butan-2-one.

Clarity - Chromatography SW
DataApex 2006
www.dataapex.com

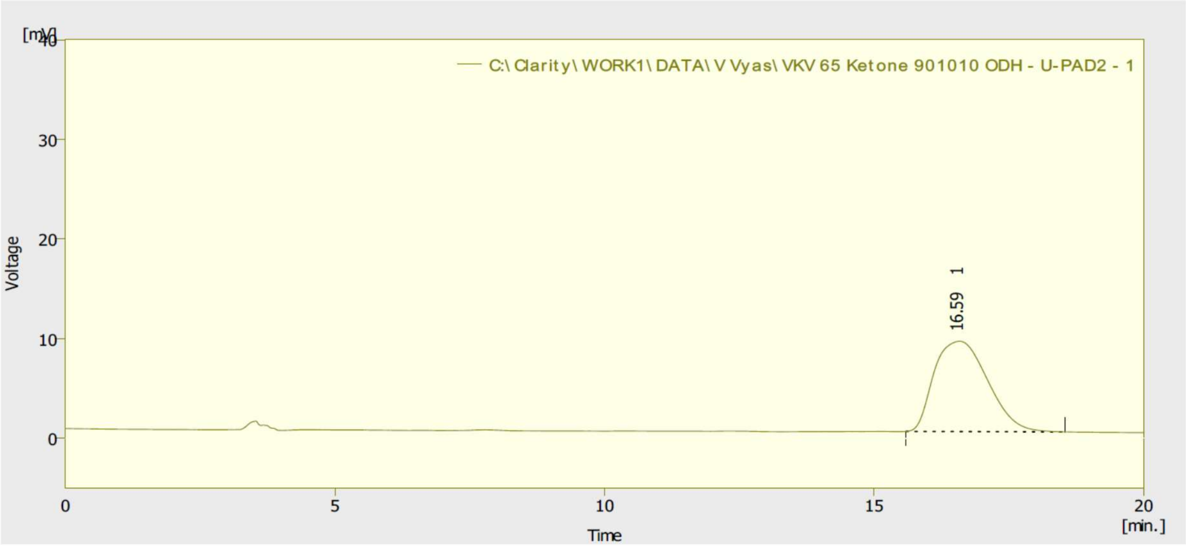

Result Table (Uncal - C:\Clarity\WORK1\DATA\V Vyas\VKV 65 Ketone 901010 ODH - U-PAD2 - 1)

	Reten. Time [min]	Area [mV.s]	Height [mV]	Area [%]	Height [%]	W05 [min]	Compound Name
1	16.588	626.586	9.072	100.0	100.0	1.13	
	Total	626.586	9.072	100.0	100.0		

3,3-Dimethyl-1-(phenylsulfonyl)butan-2-ol 11g

^{1}H NMR (400 MHz, $CDCl_3$)

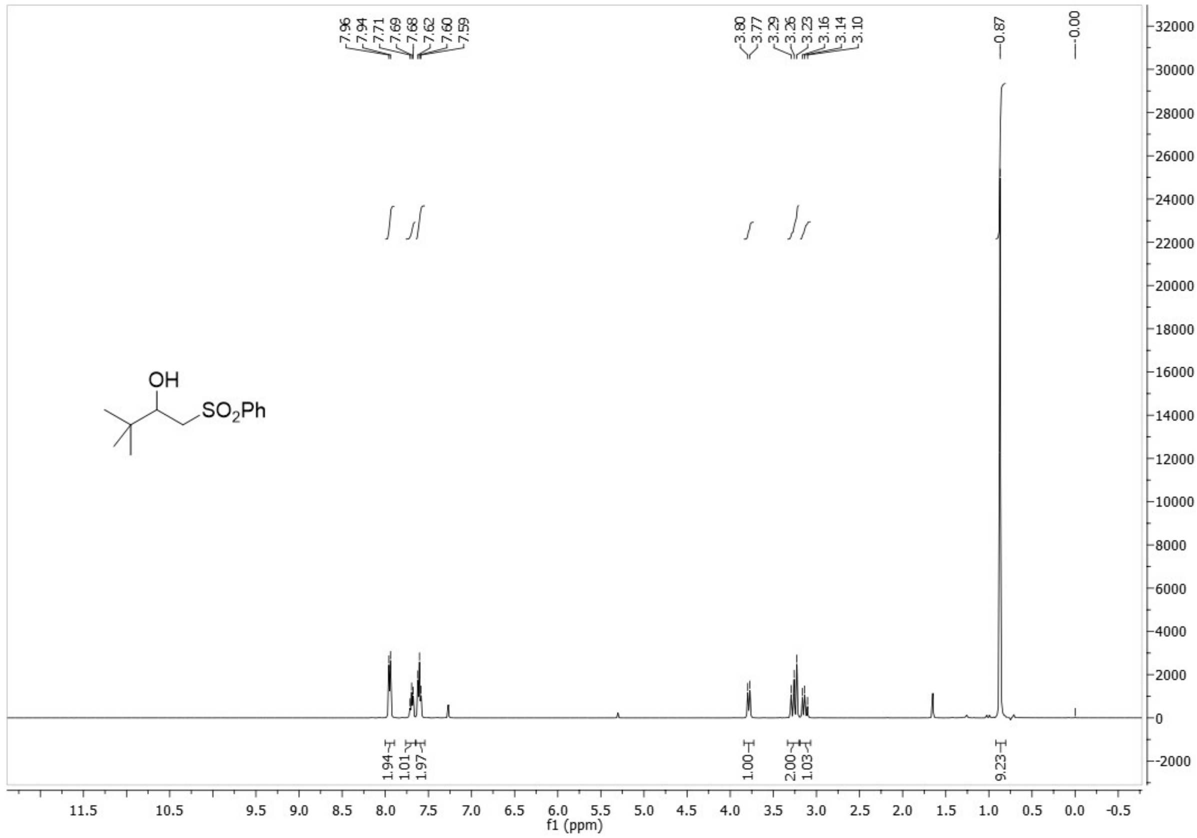

^{13}C NMR (101 MHz, $CDCl_3$

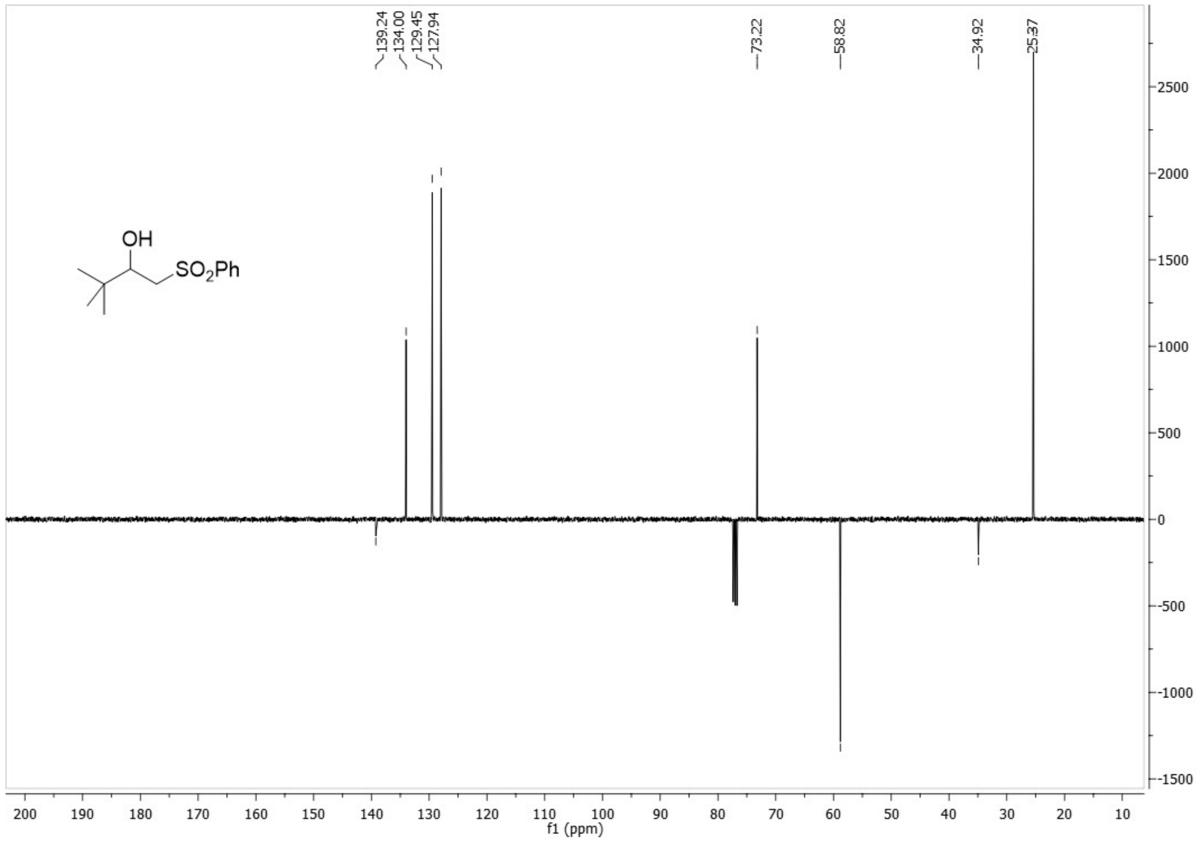

Racemic HPLC of 3,3-dimethyl-1-(phenylsulfonyl)butan-2-ol.

22/02/2019 08:47 Chromatogram C:\Clarity\WORK1\DATA\V Vyas\VKV 71 930707 ODH Racemic.prm Page 1 of 2

Clarity - Chromatography SW
DataApex 2006
www.dataapex.com

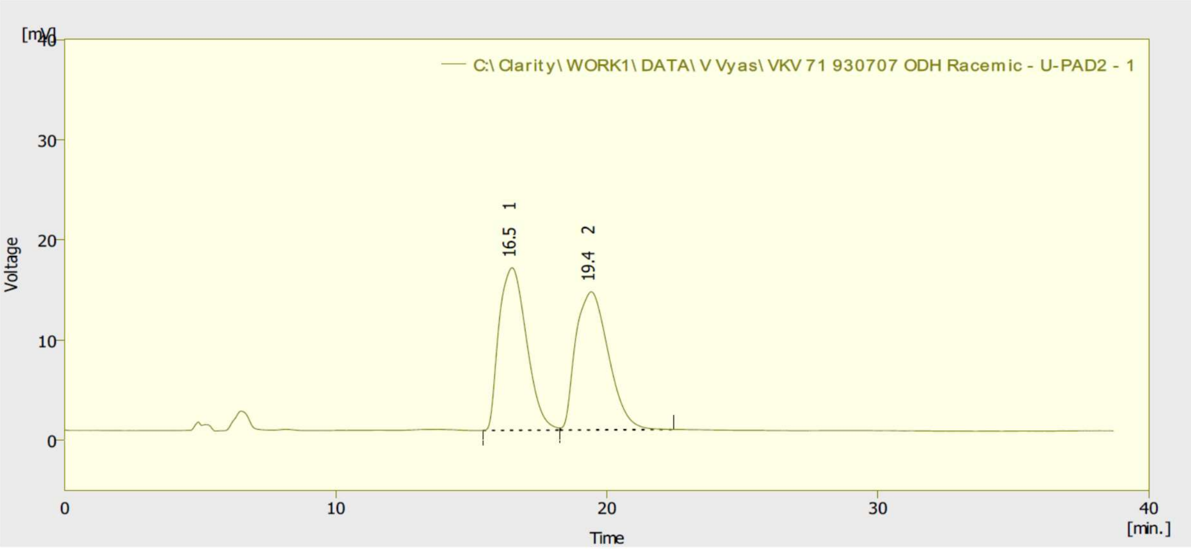

Result Table (Uncal - C:\Clarity\WORK1\DATA\V Vyas\VKV 71 930707 ODH Racemic - U-PAD2 - 1)

	Reten. Time [min]	Area [mV.s]	Height [mV]	Area [%]	Height [%]	W05 [min]	Compound Name
1	16.508	1148.015	16.226	49.8	54.1	1.15	
2	19.424	1159.102	13.784	50.2	45.9	1.37	
	Total	2307.116	30.011	100.0	100.0		

HPLC after ATH 3,3-dimethyl-1-(phenylsulfonyl)butan-2-ol. (100% conversion, 86.4% ee).

29/03/2019 07:22 Chromatogram C:\Clarity\WORK1\DATA\V Vyas\VKV76 final 930707 ODH ATH.prm Page 1 of 2

Clarity - Chromatography SW
DataApex 2006
www.dataapex.com

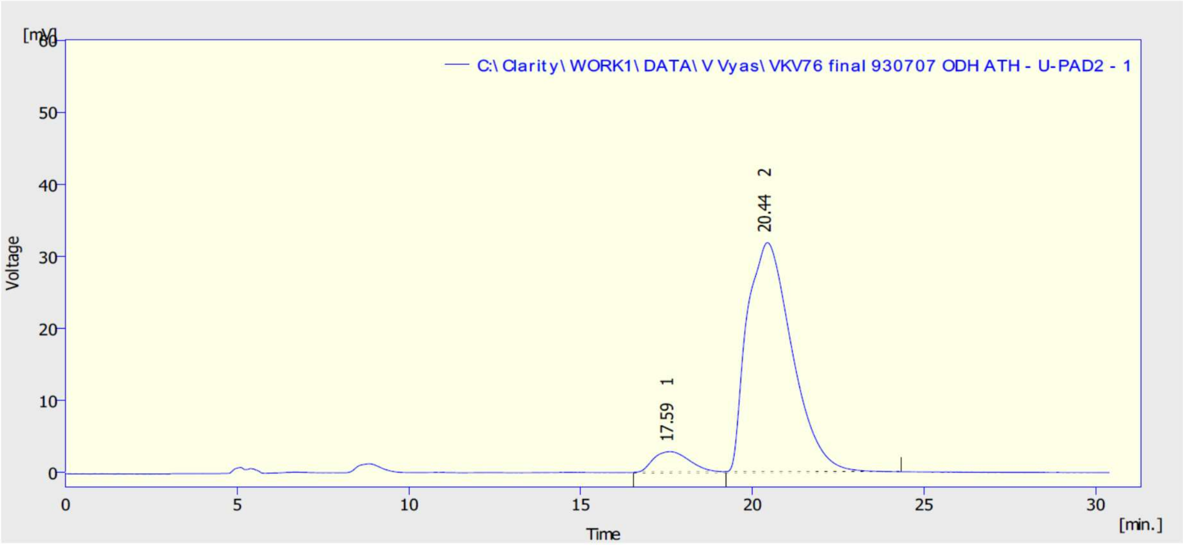

Result Table (Uncal - C:\Clarity\WORK1\DATA\V Vyas\VKV76 final 930707 ODH ATH - U-PAD2 - 1)

	Reten. Time [min]	Area [mV.s]	Height [mV]	Area [%]	Height [%]	W05 [min]	Compound Name
1	17.592	212.244	2.878	6.8	8.3	1.20	
2	20.440	2907.926	31.839	93.2	91.7	1.49	
	Total	3120.170	34.718	100.0	100.0		

1-Cyclobutyl-2-phenoxyethan-1-one

^{1}H NMR (400 MHz, $CDCl_3$)

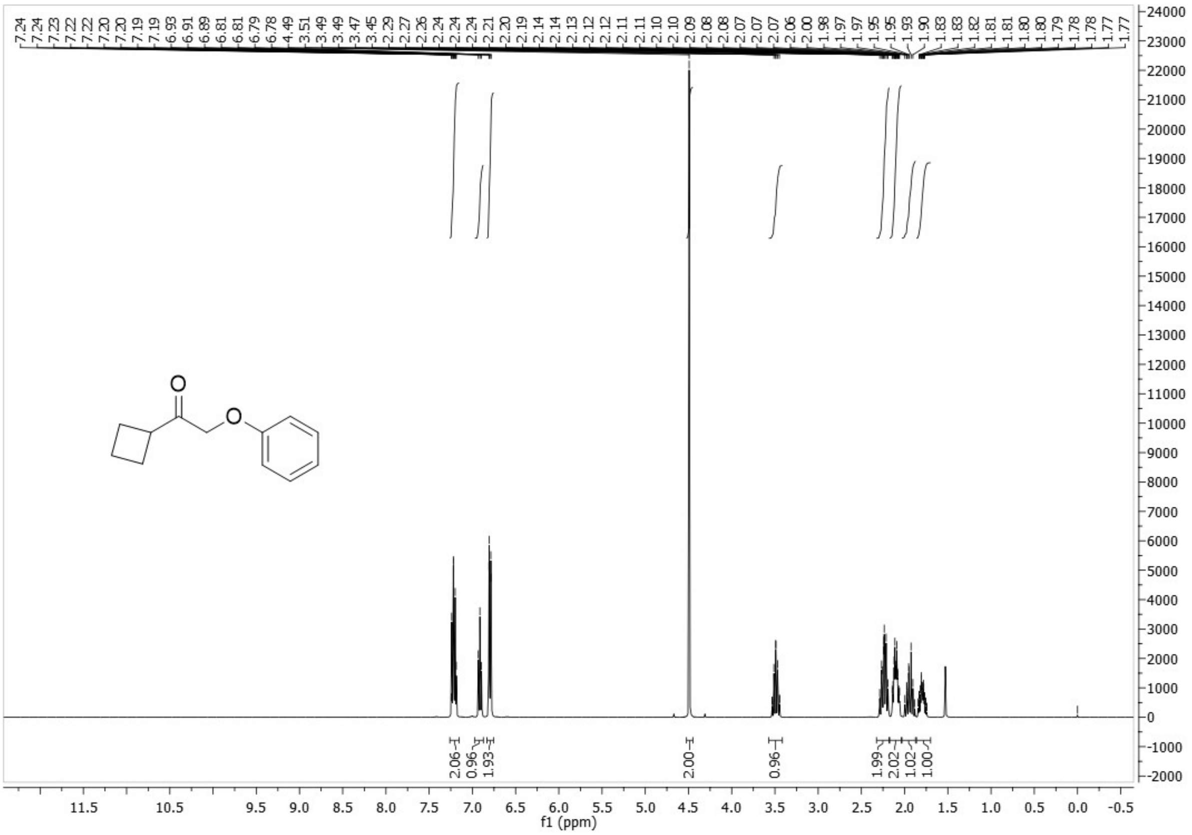

^{13}C NMR (101 MHz, $CDCl_3$

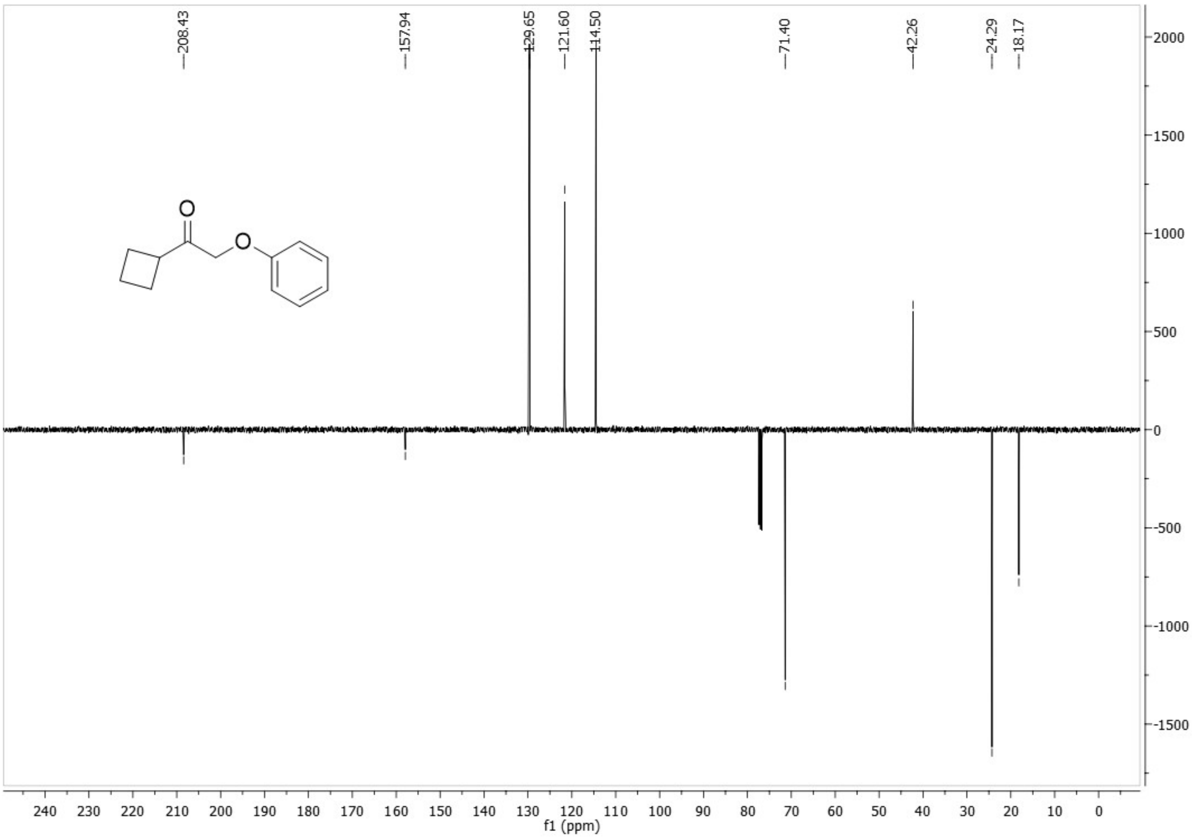

Ketone HPLC of 1-cyclobutyl-2-phenoxyethan-1-one.

Clarity - Chromatography SW
DataApex 2006
www.dataapex.com

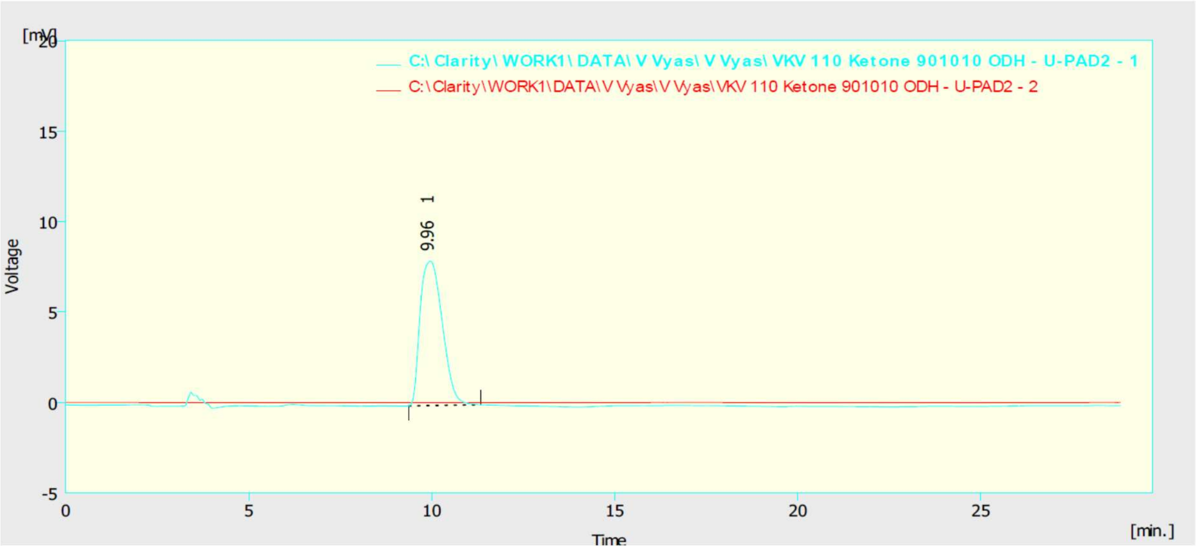

Result Table (Uncal - C:\Clarity\WORK1\DATA\V Vyas\V Vyas\VKV 110 Ketone 901010 ODH - U-PAD2 - 1)

	Reten. Time [min]	Area [mV.s]	Height [mV]	Area [%]	Height [%]	W05 [min]	Compound Name
1	9.960	336.833	7.978	100.0	100.0	0.69	
	Total	336.833	7.978	100.0	100.0		

1-Cyclobutyl-2-phenoxyethan-1-ol

^{1}H NMR (400 MHz, $CDCl_3$)

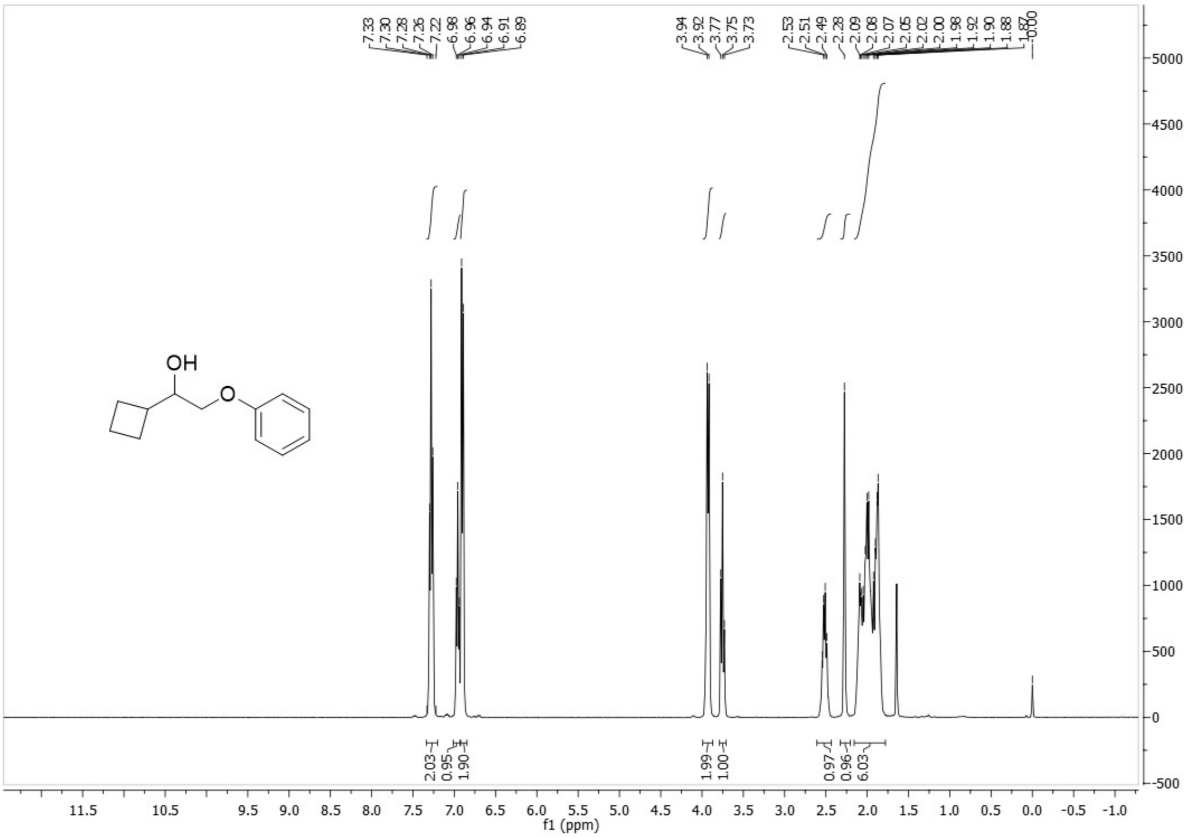

^{13}C NMR (101 MHz, $CDCl_3$

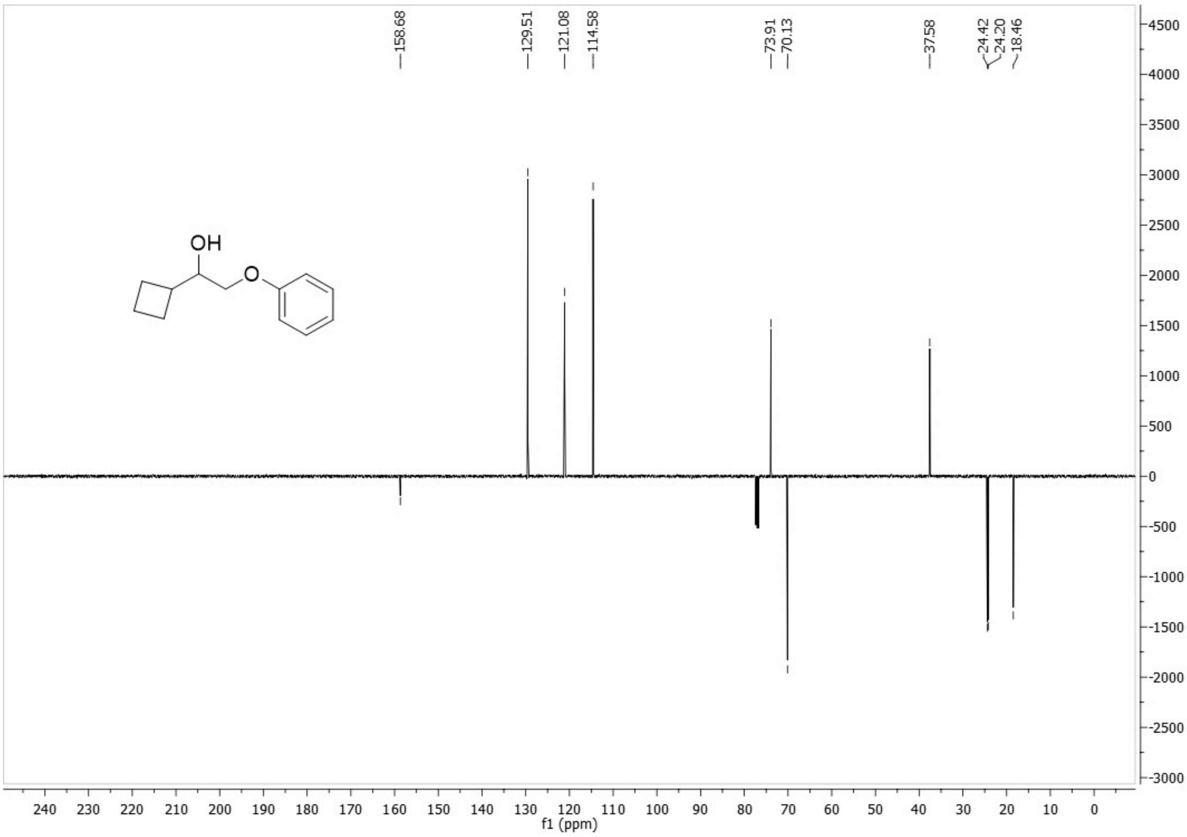

Racemic HPLC of 1-cyclobutyl-2-phenoxyethan-1-ol.

29/03/2019 07:16 Chromatogram C:\Clarity\WORK1\DATA\V Vyas\VKV 118 racemic 901010 ODH.prm Page 1 of 2

Clarity - Chromatography SW
DataApex 2006
www.dataapex.com

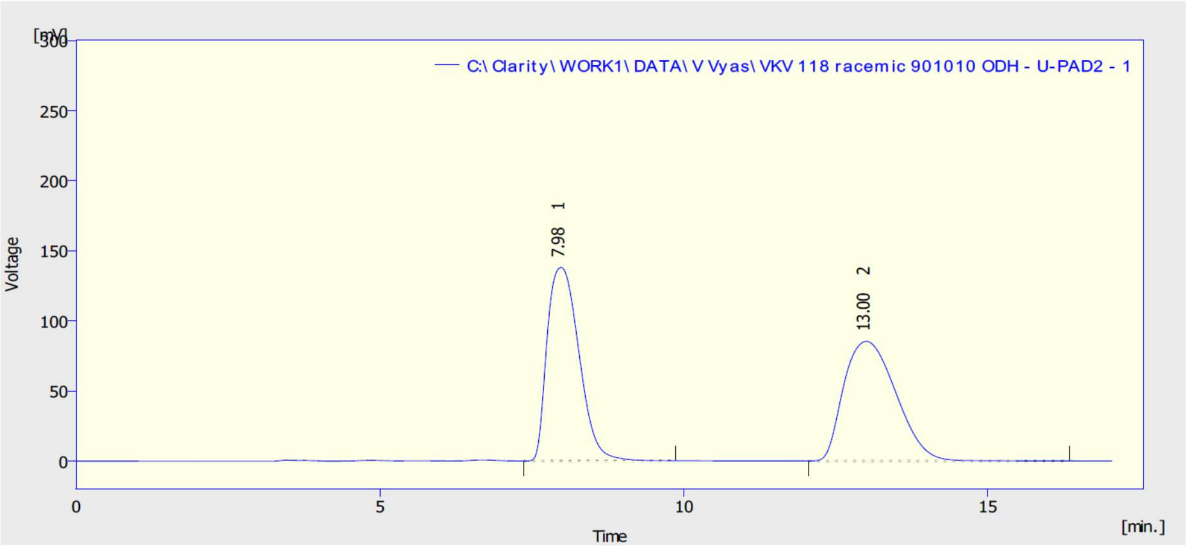

Result Table (Uncal - C:\Clarity\WORK1\DATA\V Vyas\VKV 118 racemic 901010 ODH - U-PAD2 - 1)

	Reten. Time [min]	Area [mV.s]	Height [mV]	Area [%]	Height [%]	W05 [min]	Compound Name
1	7.976	5031.699	138.076	49.7	61.8	0.59	
2	13.000	5093.306	85.380	50.3	38.2	0.97	
	Total	10125.004	223.456	100.0	100.0		

HPLC after ATH 1-cyclobutyl-2-phenoxyethan-1-ol. (100% conversion, 25.6% ee).

29/03/2019 07:17 Chromatogram C:\Clarity\WORK1\DATA\V Vyas\VKV 119 ATH 901010 ODH.prm Page 1 of 2

Clarity - Chromatography SW
DataApex 2006
www.dataapex.com

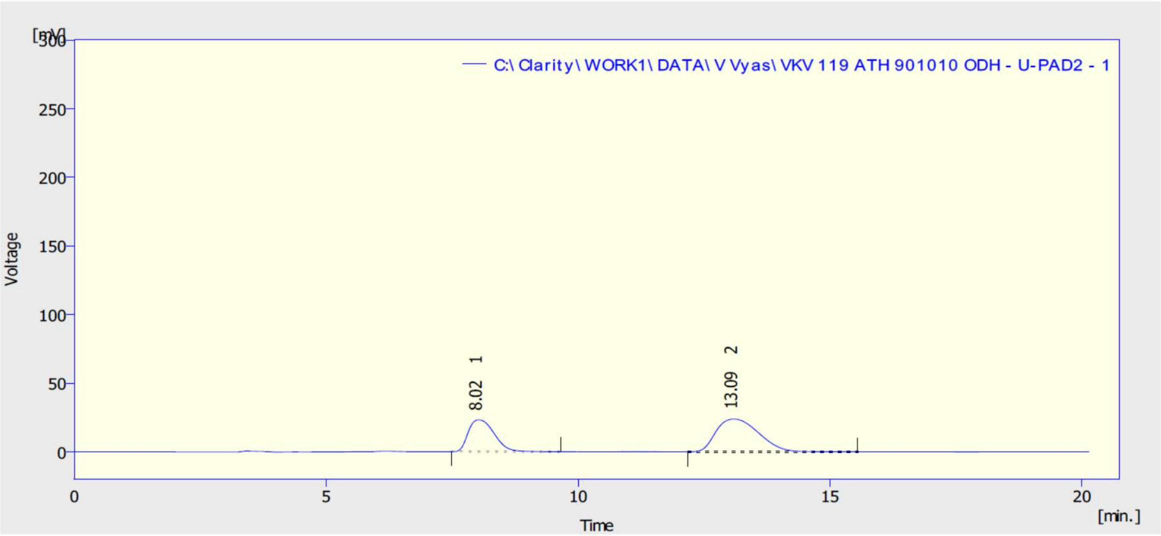

Result Table (Uncal - C:\Clarity\WORK1\DATA\V Vyas\VKV 119 ATH 901010 ODH - U-PAD2 - 1)

	Reten. Time [min]	Area [mV.s]	Height [mV]	Area [%]	Height [%]	W05 [min]	Compound Name
1	8.024	831.558	23.178	37.2	49.3	0.58	
2	13.092	1402.054	23.875	62.8	50.7	0.96	
	Total	2233.612	47.053	100.0	100.0		

1-Cyclopropyl-2-phenoxyethan-1-one

^{1}H NMR (400 MHz, $CDCl_3$)

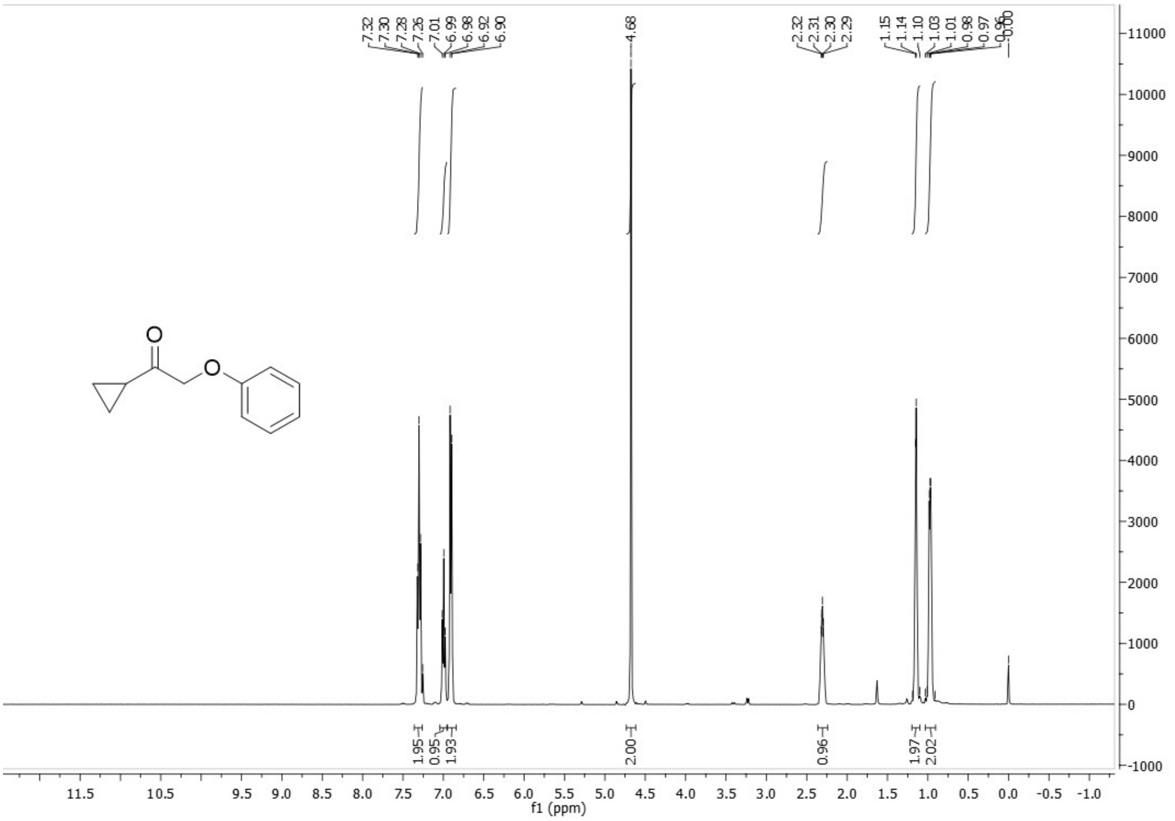

^{13}C NMR (101 MHz, $CDCl_3$

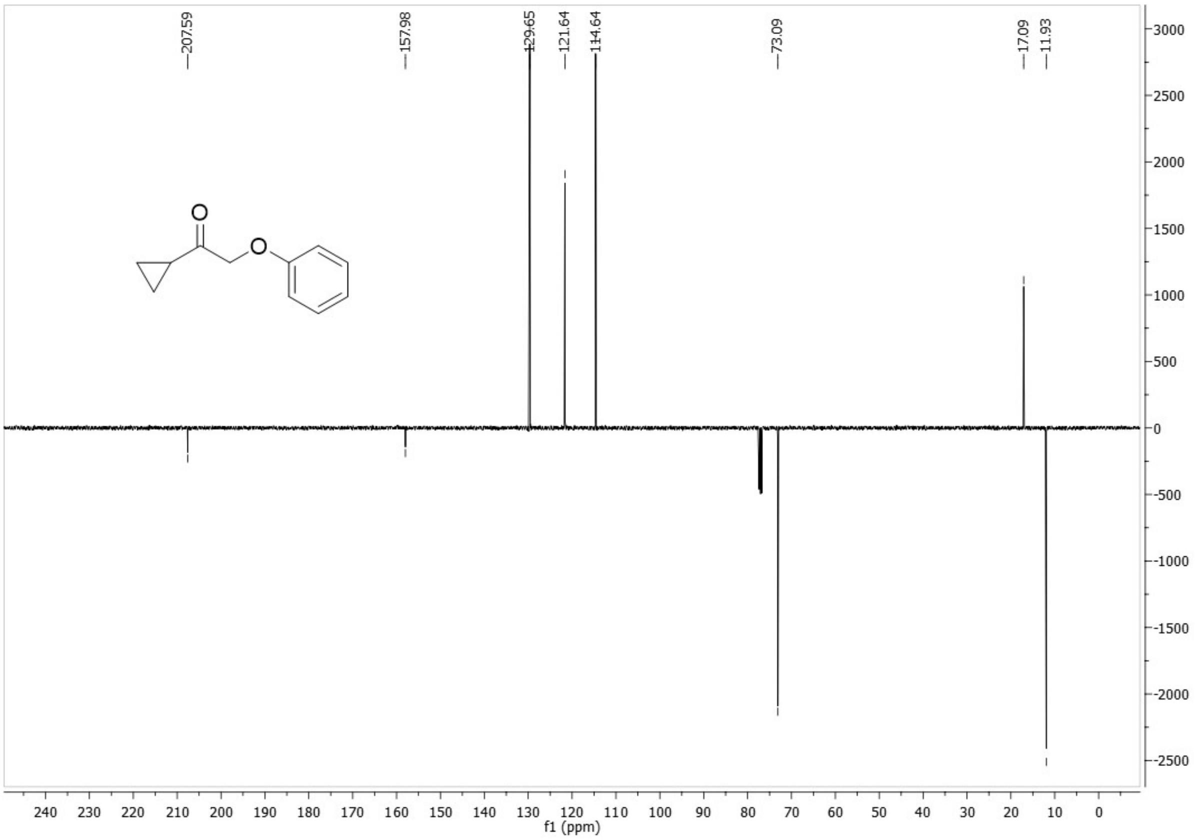

Ketone HPLC of 1-cyclopropyl-2-phenoxyethan-1-one.

Clarity - Chromatography SW
DataApex 2006
www.dataapex.com

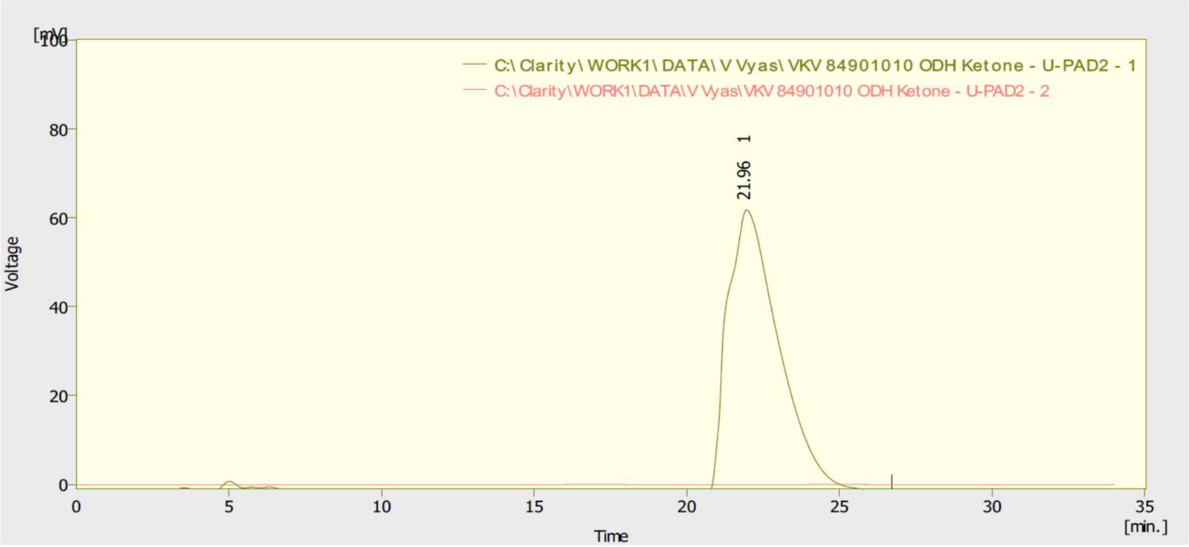

Result Table (Uncal - C:\Clarity\WORK1\DATA\V Vyas\VKV 84901010 ODH Ketone - U-PAD2 - 1)

	Reten. Time [min]	Area [mV.s]	Height [mV]	Area [%]	Height [%]	W05 [min]	Compound Name
1	21.960	7313.532	63.064	100.0	100.0	1.91	
	Total	7313.532	63.064	100.0	100.0		

1-Cyclopropyl-2-phenoxyethan-1-ol

^{1}H NMR (400 MHz, $CDCl_3$)

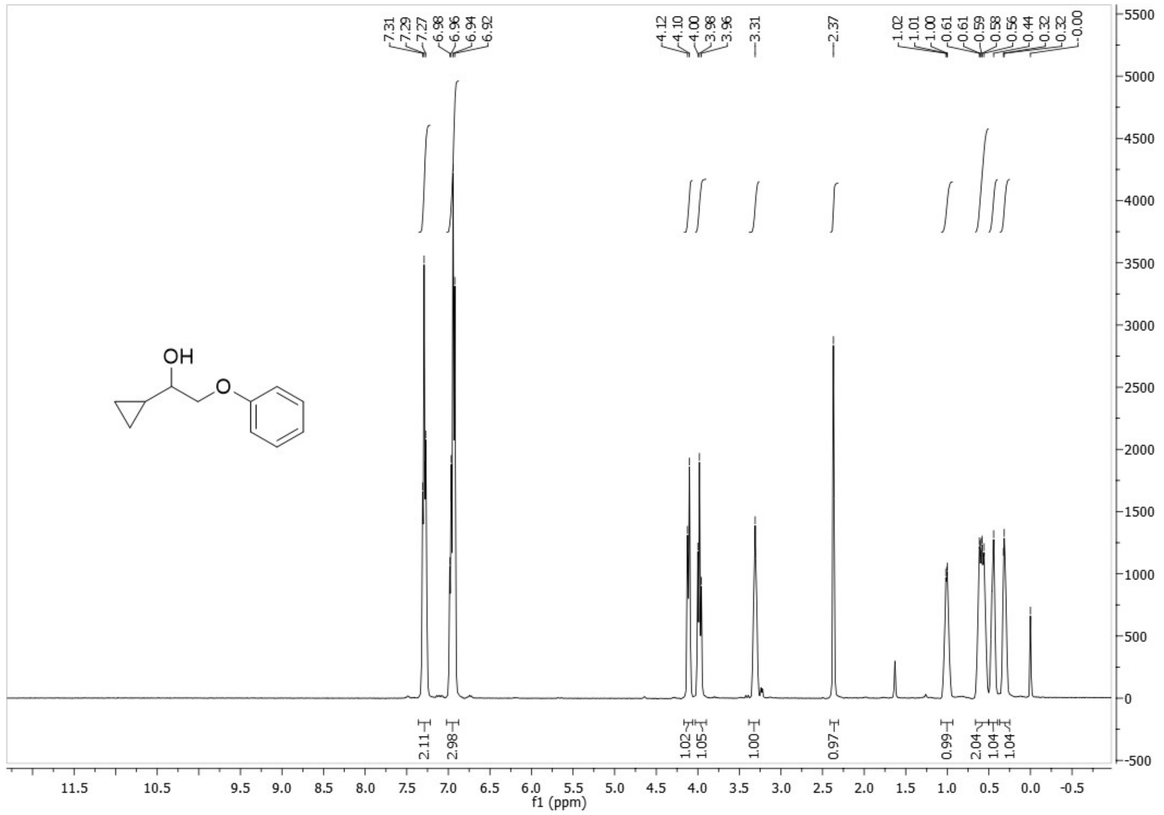

^{13}C NMR (101 MHz, $CDCl_3$

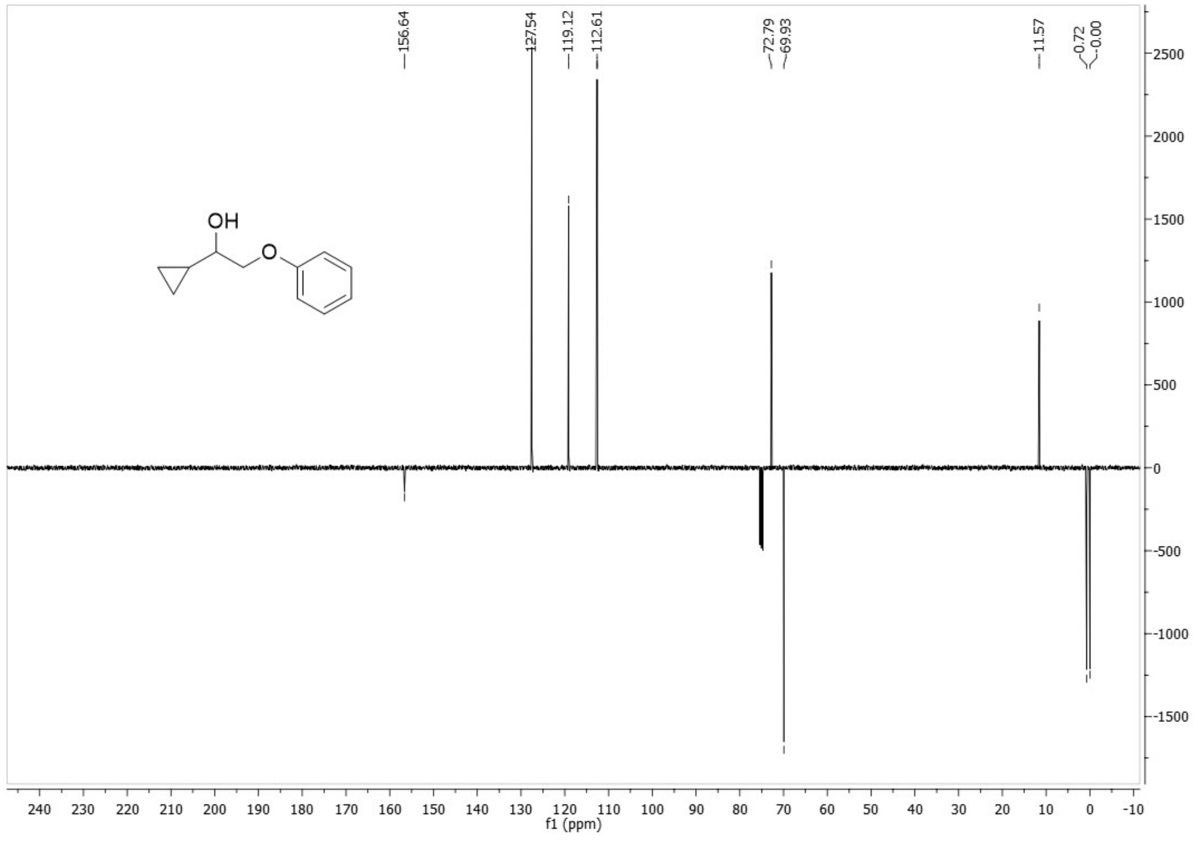

Racemic HPLC of 1-cyclopropyl-2-phenoxyethan-1-ol.

07/03/2019 13:51 Chromatogram C:\Clarity\WORK1\DATA\V Vyas\VKV 87 901010 ATH ODH.prm Page 1 of 2

Clarity - Chromatography SW
DataApex 2006
www.dataapex.com

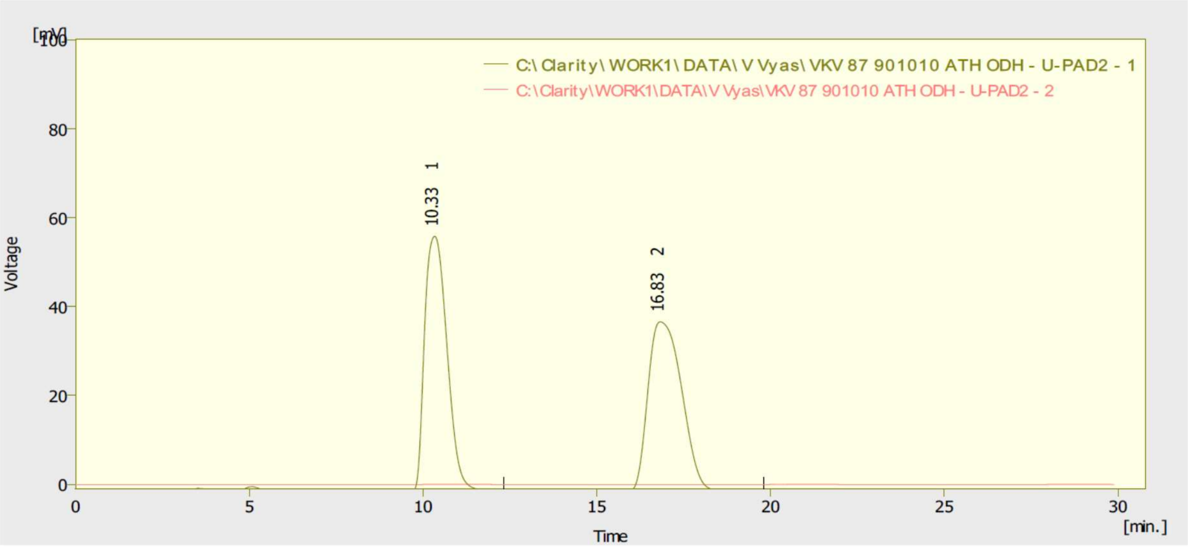

Result Table (Uncal - C:\Clarity\WORK1\DATA\V Vyas\VKV 87 901010 ATH ODH - U-PAD2 - 1)

	Reten. Time [min]	Area [mV.s]	Height [mV]	Area [%]	Height [%]	W05 [min]	Compound Name
1	10.332	2575.801	57.388	49.8	60.0	0.73	
2	16.828	2591.744	38.272	50.2	40.0	1.10	
	Total	5167.545	95.660	100.0	100.0		

HPLC after ATH 1-cyclopropyl-2-phenoxyethan-1-ol. (100% conversion, 35.6% ee).

07/03/2019 13:51 Chromatogram C:\Clarity\WORK1\DATA\V Vyas\VKV 88 901010 ATH ODH.prm Page 1 of 2

Clarity - Chromatography SW
DataApex 2006
www.dataapex.com

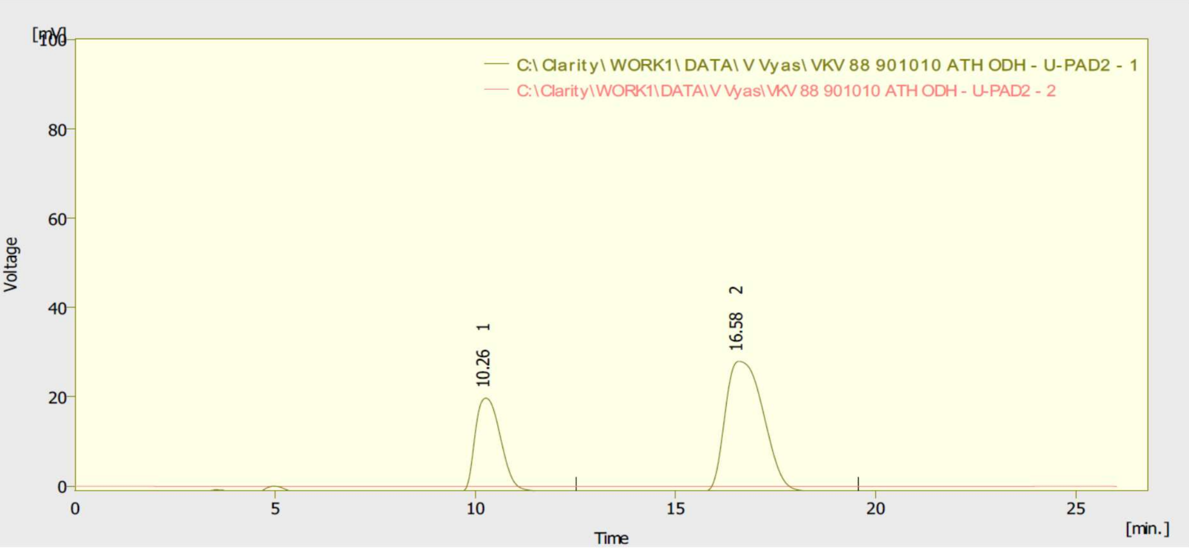

Result Table (Uncal - C:\Clarity\WORK1\DATA\V Vyas\VKV 88 901010 ATH ODH - U-PAD2 - 1)

	Reten. Time [min]	Area [mV.s]	Height [mV]	Area [%]	Height [%]	W05 [min]	Compound Name
1	10.260	924.717	20.910	32.2	41.7	0.71	
2	16.584	1943.177	29.209	67.8	58.3	1.08	
	Total	2867.894	50.119	100.0	100.0		

1-Phenoxypentan-2-one

^{1}H NMR (400 MHz, $CDCl_3$)

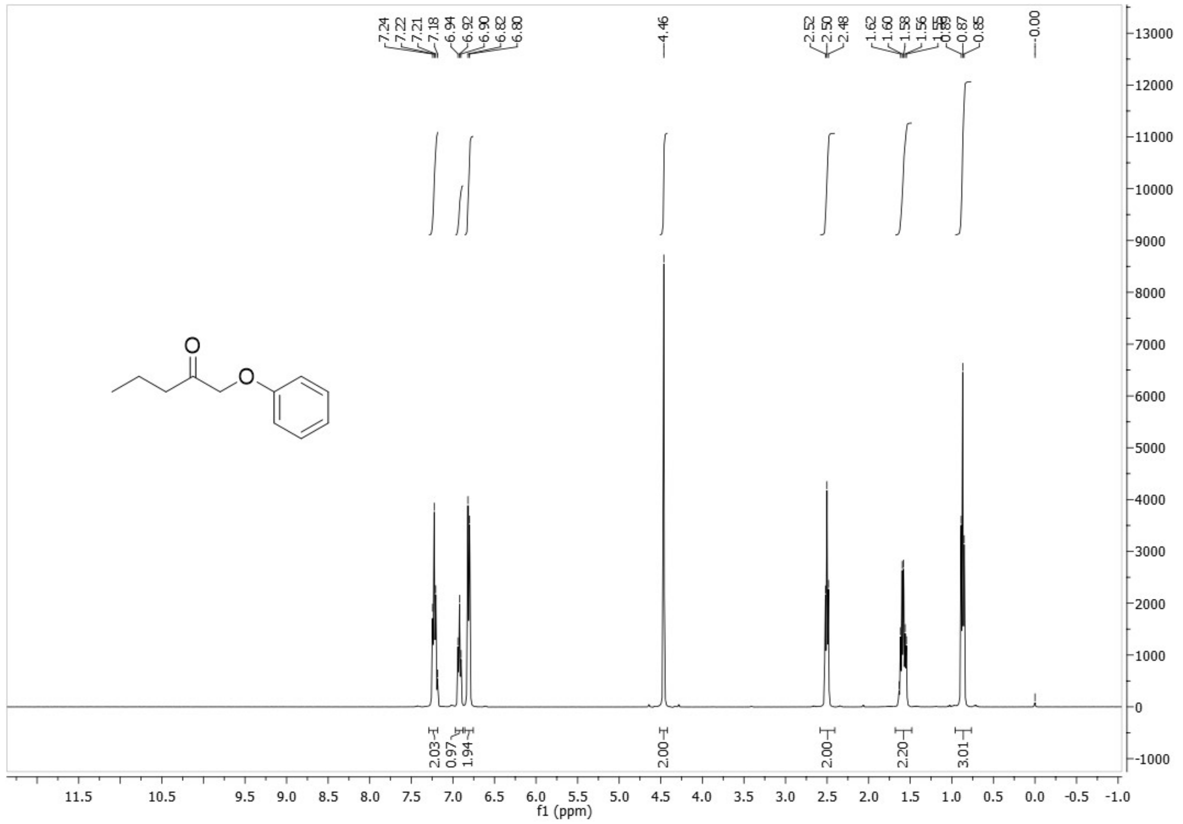

^{13}C NMR (101 MHz, $CDCl_3$

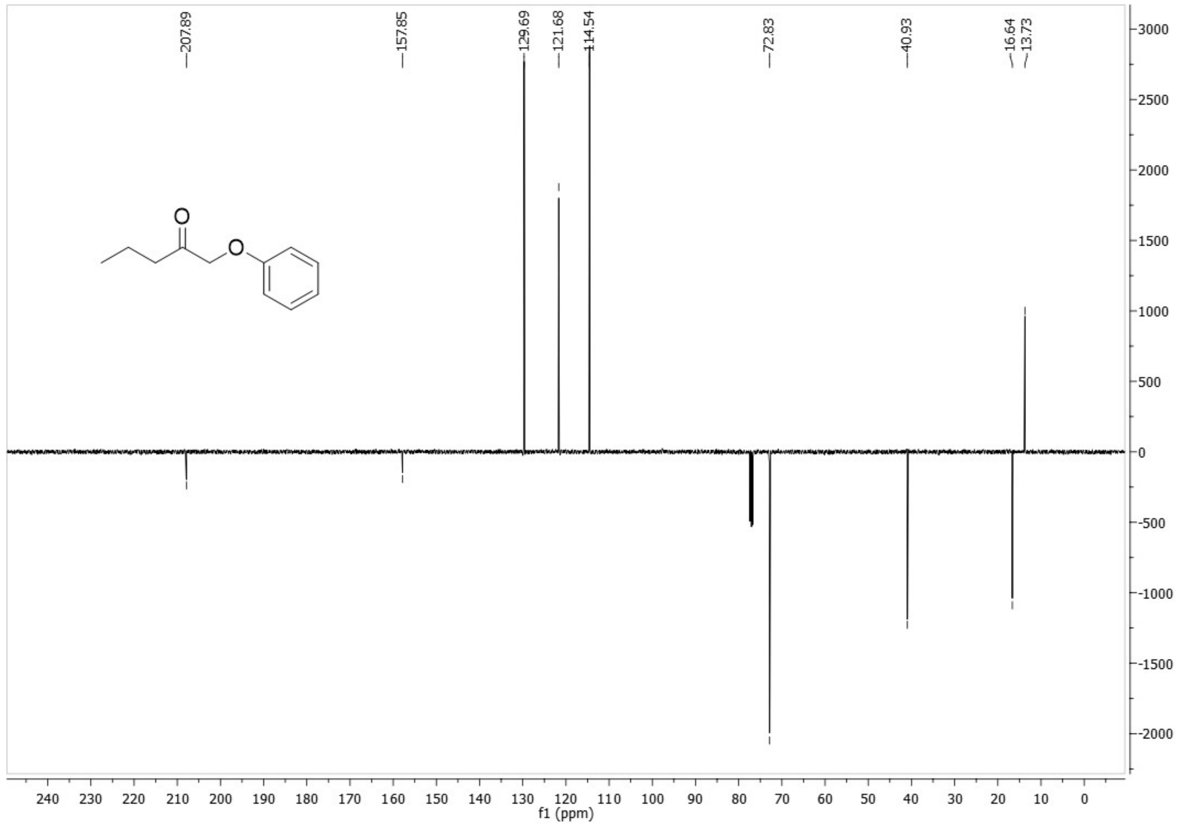

Ketone HPLC of 1-phenoxypentan-2-one.

Clarity - Chromatography SW
DataApex 2006
www.dataapex.com

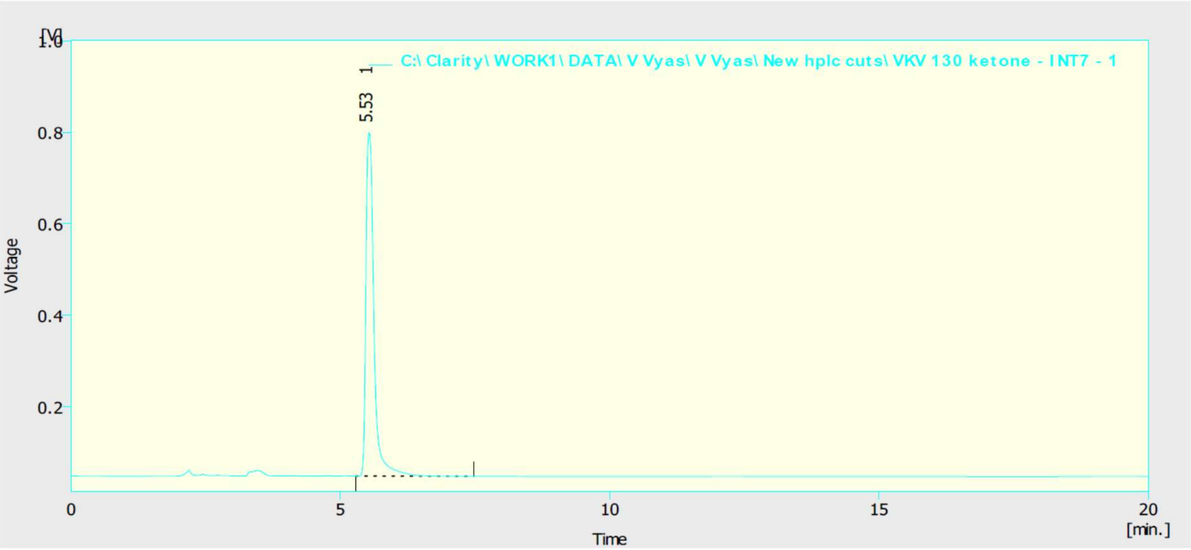

Result Table (Uncal - C:\Clarity\WORK1\DATA\V Vyas\V Vyas\New hplc cuts\VKV 130 ketone - INT7 - 1)

	Reten. Time [min]	Area [mV.s]	Height [mV]	Area [%]	Height [%]	W05 [min]	Compound Name
1	5.533	7767.602	750.219	100.0	100.0	0.15	
	Total	7767.602	750.219	100.0	100.0		

1-Phenoxypentan-2-ol

^{1}H NMR (400 MHz, $CDCl_3$)

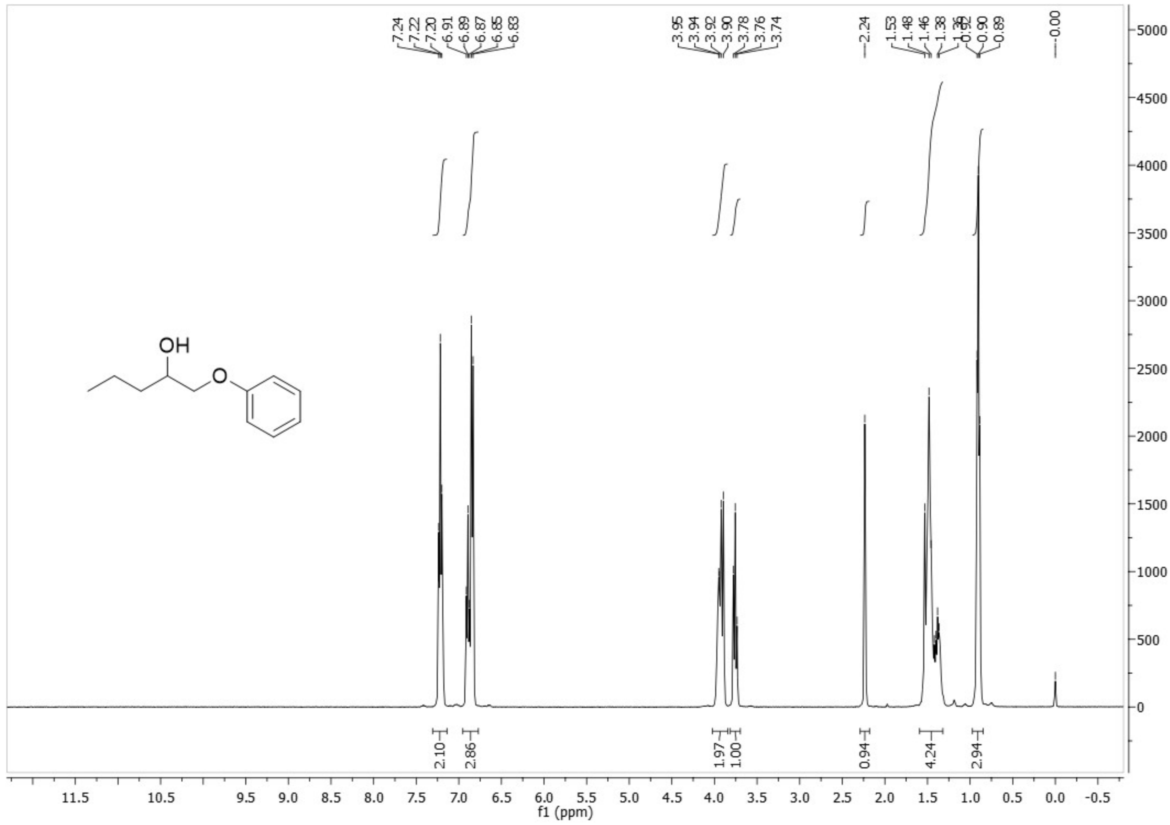

^{13}C NMR (101 MHz, $CDCl_3$

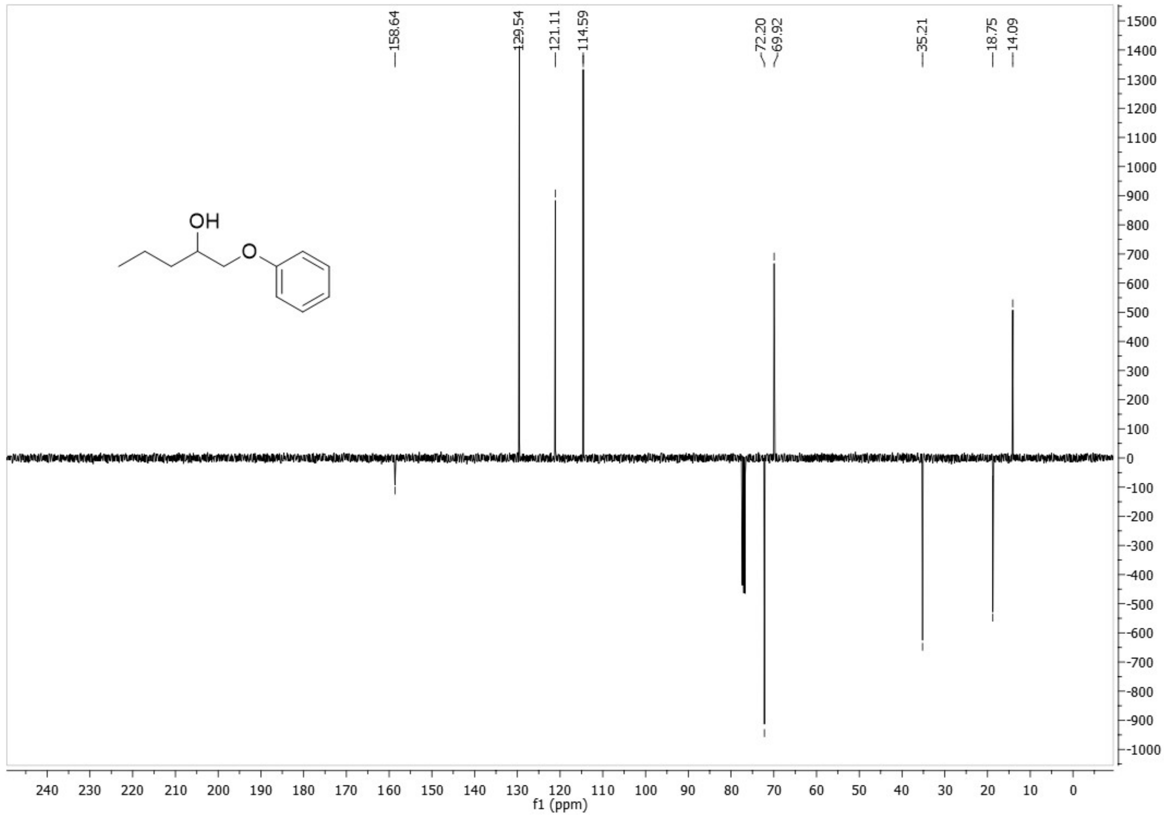

Racemic HPLC of 1-phenoxypentan-2-ol.

Clarity - Chromatography SW
DataApex 2006
www.dataapex.com

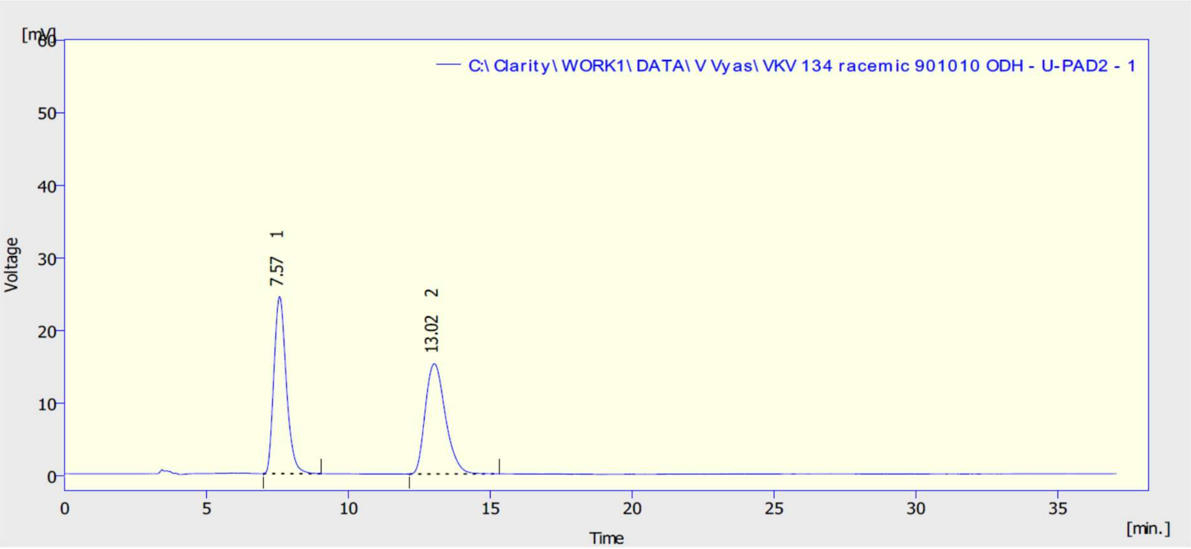

Result Table (Uncal - C:\Clarity\WORK1\DATA\V Vyas\VKV 134 racemic 901010 ODH - U-PAD2 - 1)

	Reten. Time [min]	Area [mV.s]	Height [mV]	Area [%]	Height [%]	W05 [min]	Compound Name
1	7.572	740.820	24.366	49.7	61.6	0.47	
2	13.024	748.460	15.172	50.3	38.4	0.77	
	Total	1489.280	39.538	100.0	100.0		

HPLC after ATH 1-phenoxypentan-2-ol. (100% conversion, 38.2% ee).

Clarity - Chromatography SW
DataApex 2006
www.dataapex.com

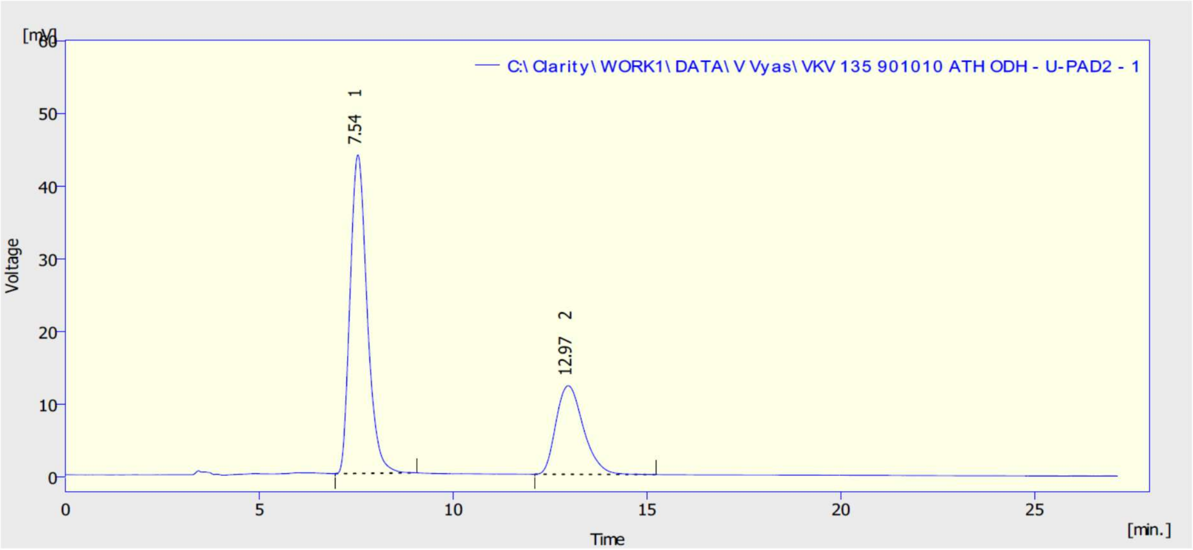

Result Table (Uncal - C:\Clarity\WORK1\DATA\V Vyas\VKV 135 901010 ATH ODH - U-PAD2 - 1)

	Reten. Time [min]	Area [mV.s]	Height [mV]	Area [%]	Height [%]	W05 [min]	Compound Name
1	7.540	1337.737	43.761	69.1	78.2	0.47	
2	12.968	599.150	12.171	30.9	21.8	0.76	
	Total	1936.887	55.931	100.0	100.0		

3,3-Dimethyl-1-phenoxybutan-2-one

^{1}H NMR (400 MHz, $CDCl_3$)

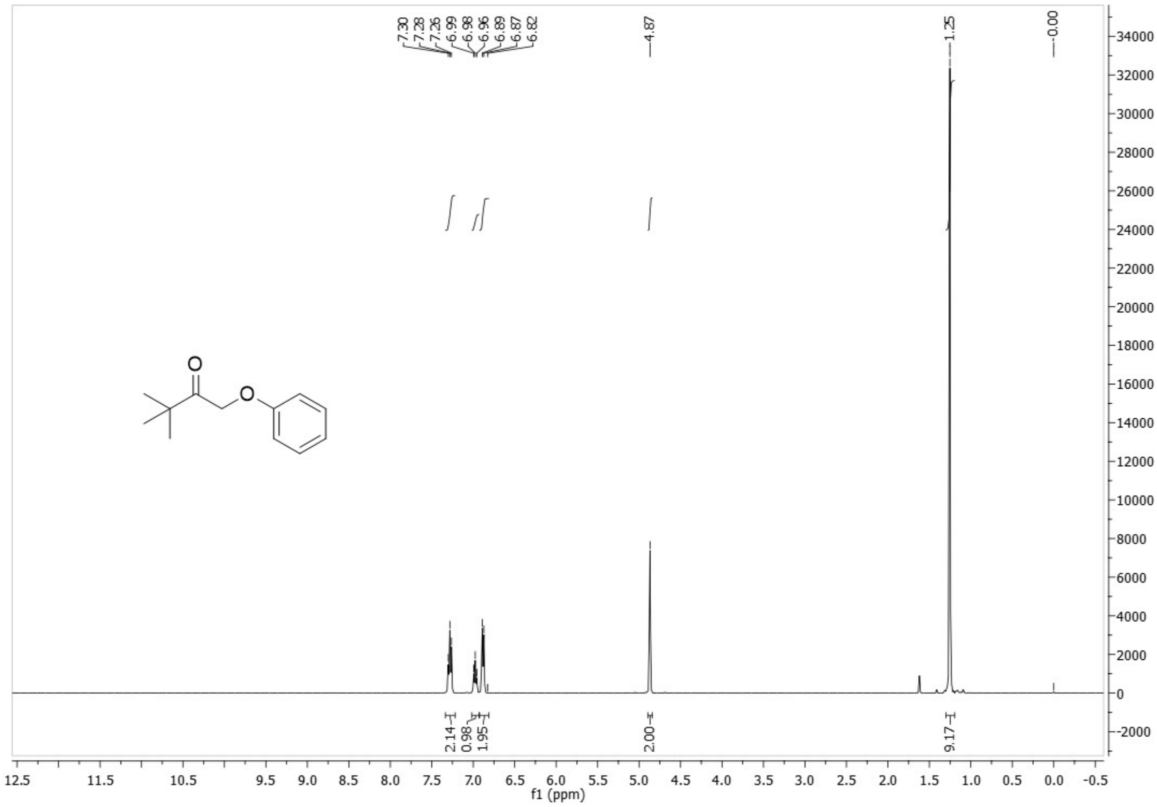

^{13}C NMR (101 MHz, $CDCl_3$

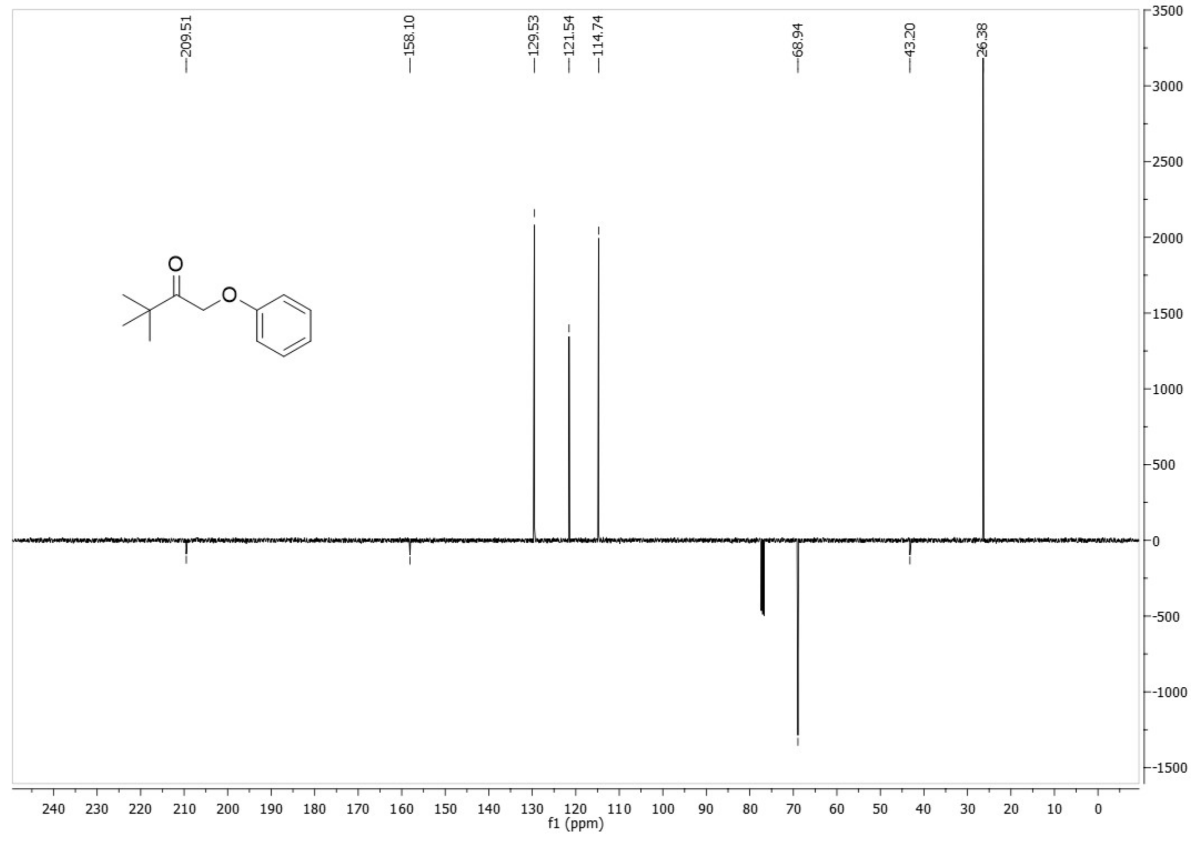

Ketone HPLC of 3,3-dimethyl-1-phenoxybutan-2-one.

Clarity - Chromatography SW
DataApex 2006
www.dataapex.com

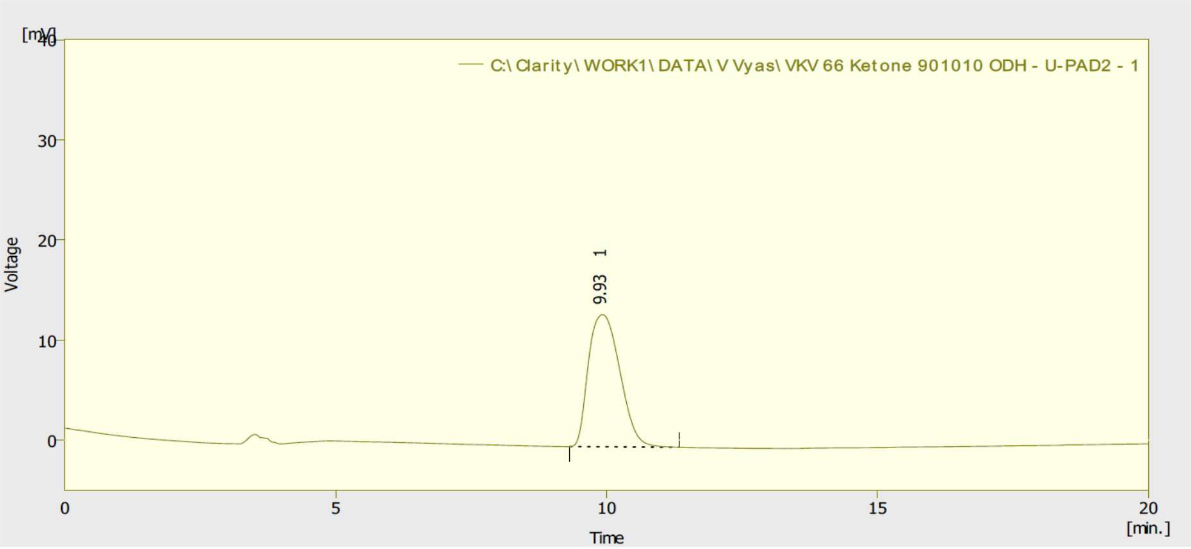

Result Table (Uncal - C:\Clarity\WORK1\DATA\V Vyas\VKV 66 Ketone 901010 ODH - U-PAD2 - 1)

	Reten. Time [min]	Area [mV.s]	Height [mV]	Area [%]	Height [%]	W05 [min]	Compound Name
1	9.928	533.727	13.200	100.0	100.0	0.66	
	Total	533.727	13.200	100.0	100.0		

3,3-Dimethyl-1-phenoxybutan-2-ol

^{1}H NMR (400 MHz, $CDCl_3$)

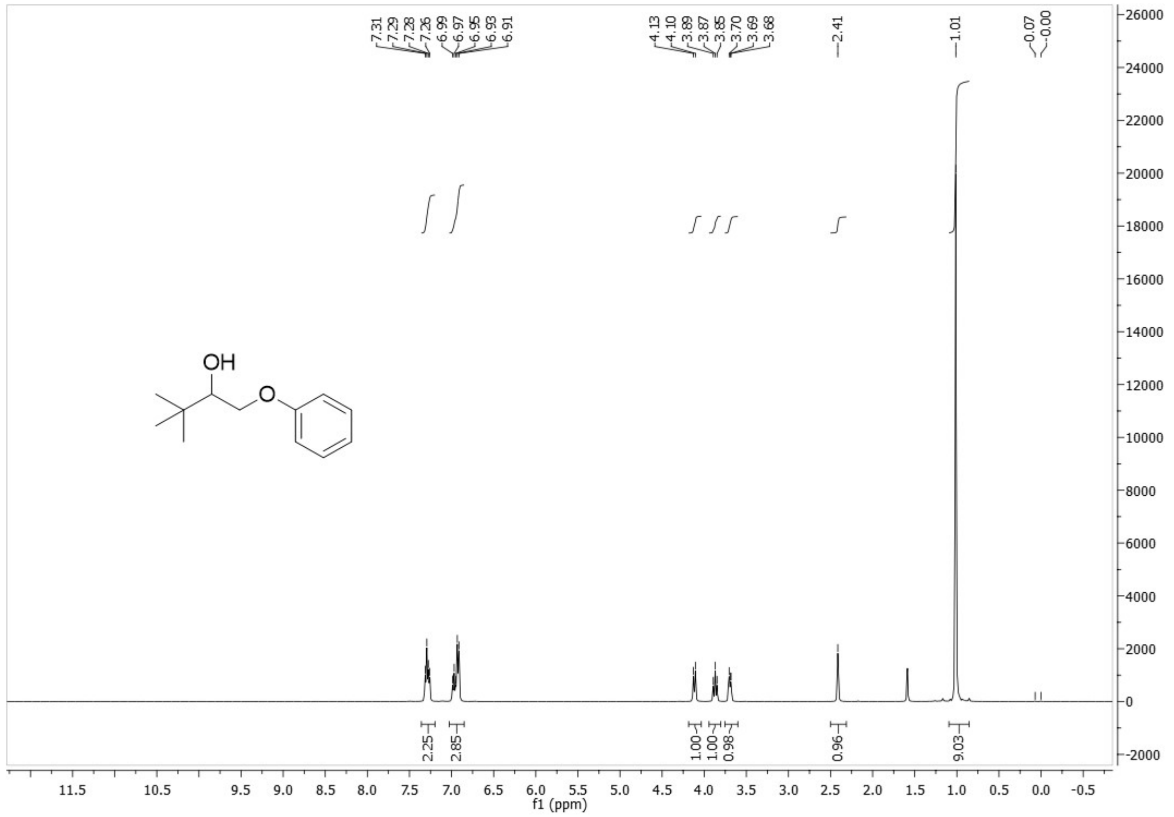

^{13}C NMR (101 MHz, $CDCl_3$

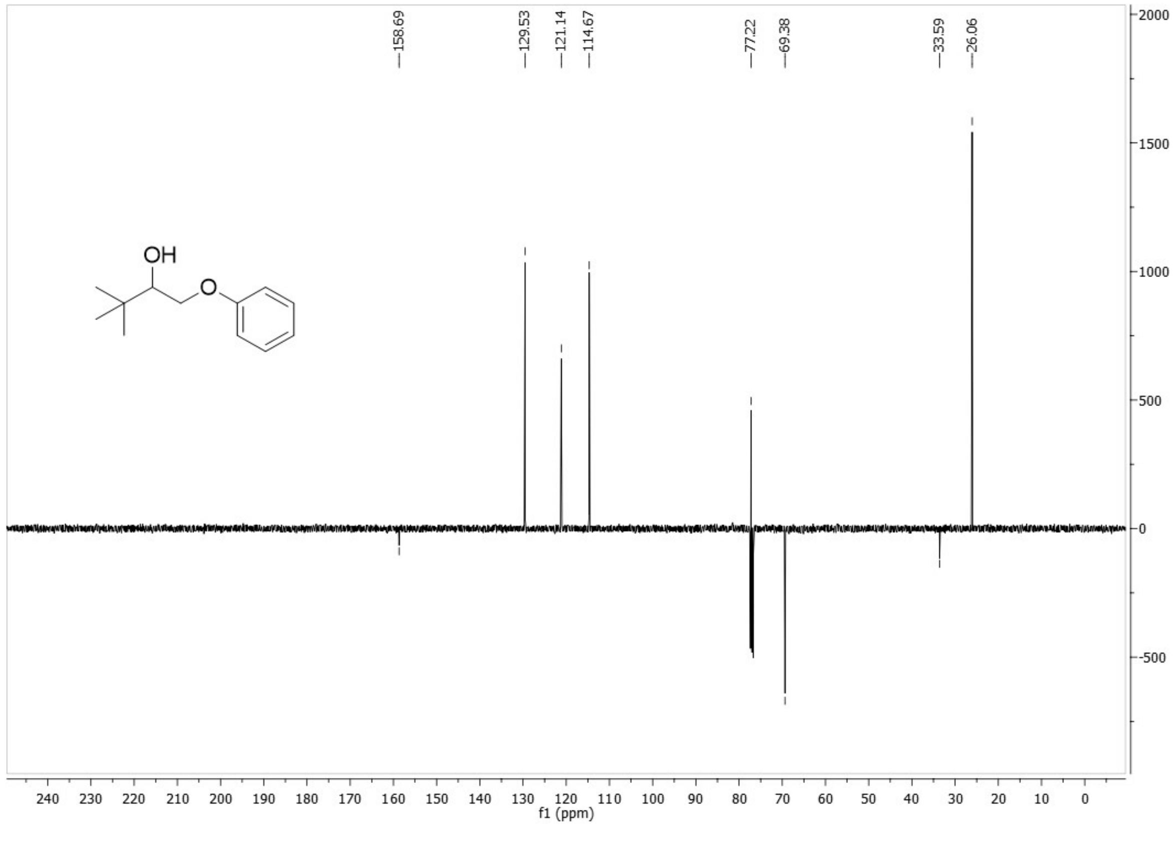

Racemic HPLC of 3,3-dimethyl-1-phenoxybutan-2-ol.

22/02/2019 08:45 Chromatogram C:\Clarity\WORK1\DATA\V Vyas\VKV 69 901010 ODH.prm Page 1 of 2

Clarity - Chromatography SW
DataApex 2006
www.dataapex.com

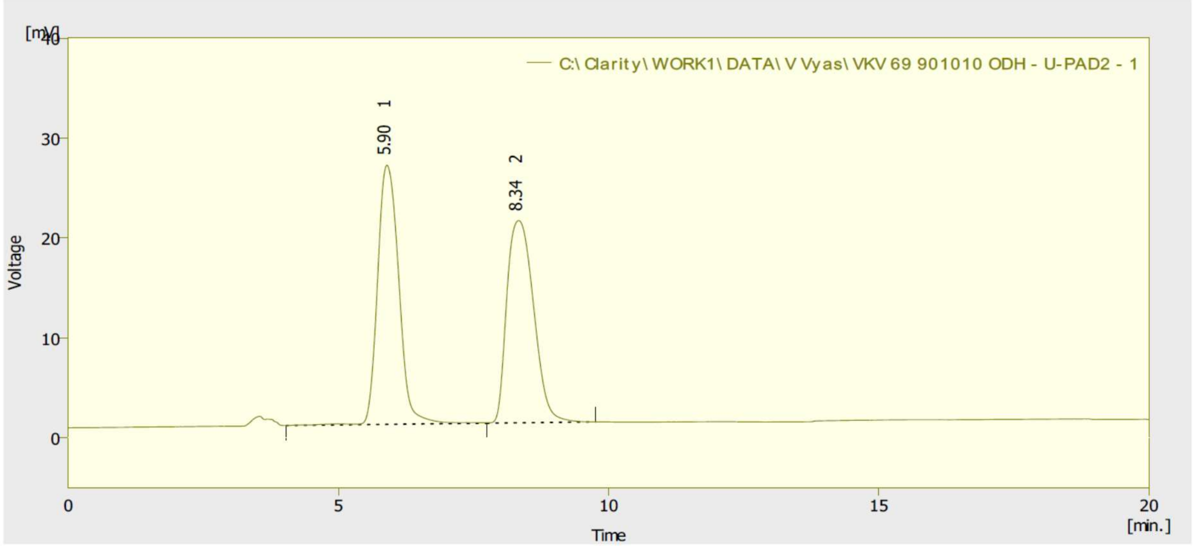

Result Table (Uncal - C:\Clarity\WORK1\DATA\V Vyas\VKV 69 901010 ODH - U-PAD2 - 1)

	Reten. Time [min]	Area [mV.s]	Height [mV]	Area [%]	Height [%]	W05 [min]	Compound Name
1	5.900	712.419	25.924	50.5	56.2	0.43	
2	8.336	698.567	20.233	49.5	43.8	0.56	
	Total	1410.987	46.157	100.0	100.0		

HPLC after ATH 3,3-dimethyl-1-phenoxybutan-2-ol. (100% conversion, 30.4% ee).

22/02/2019 08:46 Chromatogram C:\Clarity\WORK1\DATA\V Vyas\VKV 70 901010 ATH.prm Page 1 of 2

Clarity - Chromatography SW
DataApex 2006
www.dataapex.com

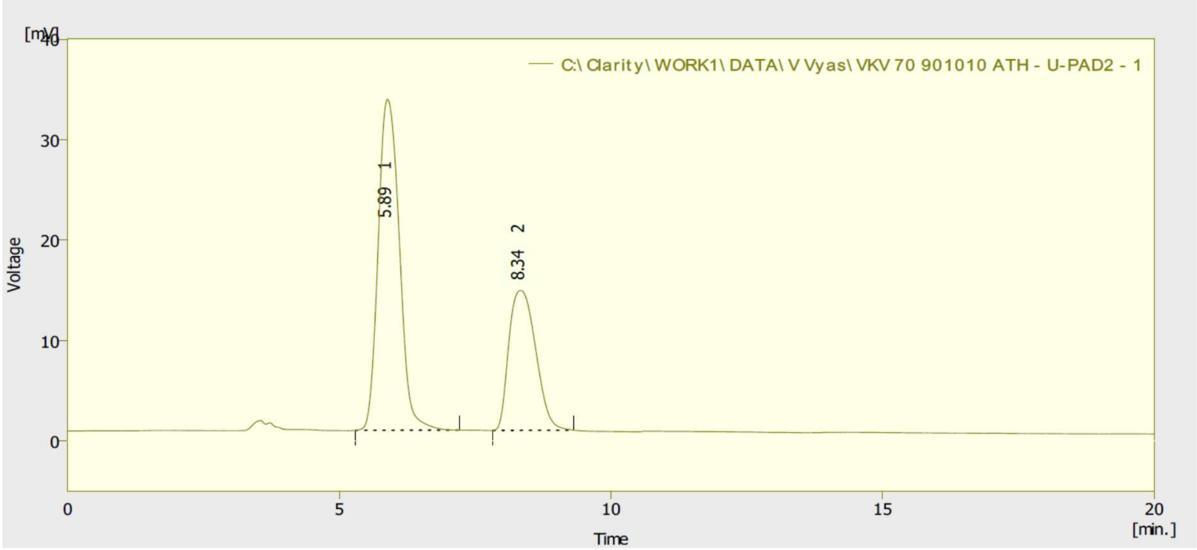

Result Table (Uncal - C:\Clarity\WORK1\DATA\V Vyas\VKV 70 901010 ATH - U-PAD2 - 1)

	Reten. Time [min]	Area [mV.s]	Height [mV]	Area [%]	Height [%]	W05 [min]	Compound Name
1	5.888	893.826	32.932	65.2	70.3	0.43	
2	8.340	477.759	13.930	34.8	29.7	0.56	
	Total	1371.585	46.862	100.0	100.0		

1-Cyclohexyl-2-(phenylthio)ethan-1-one

^{1}H NMR (400 MHz, $CDCl_3$)

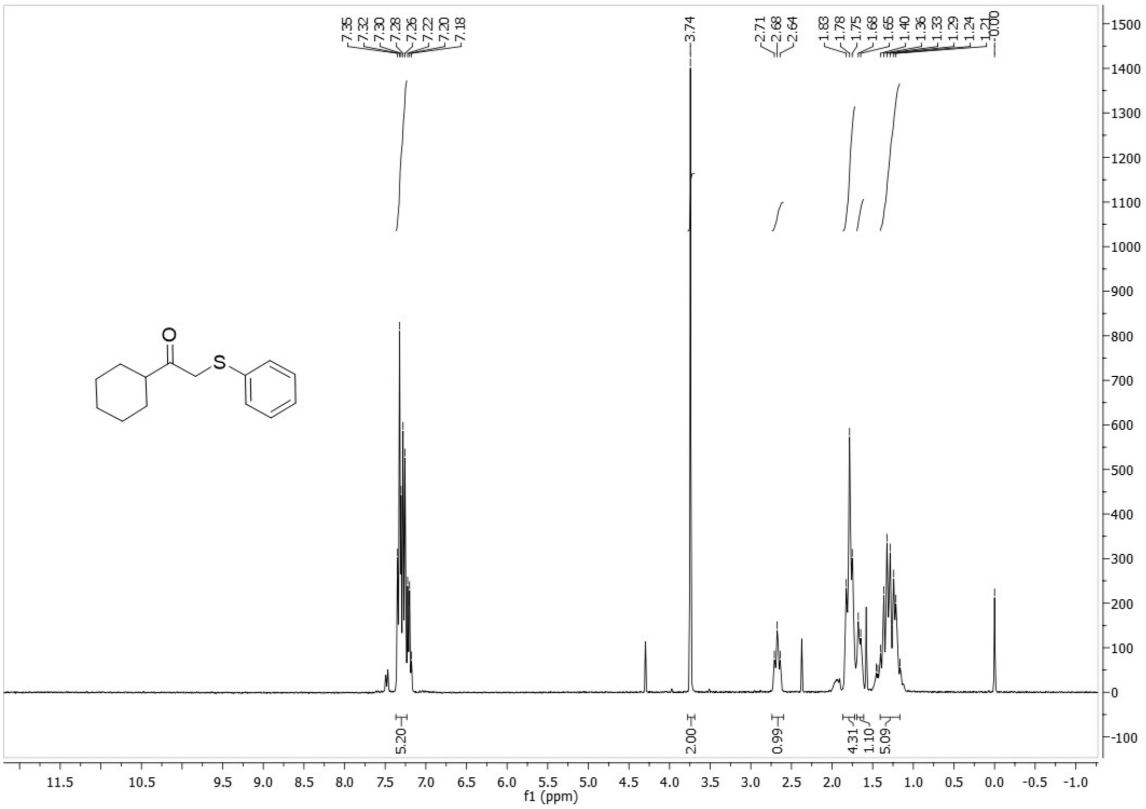

^{13}C NMR (101 MHz, $CDCl_3$

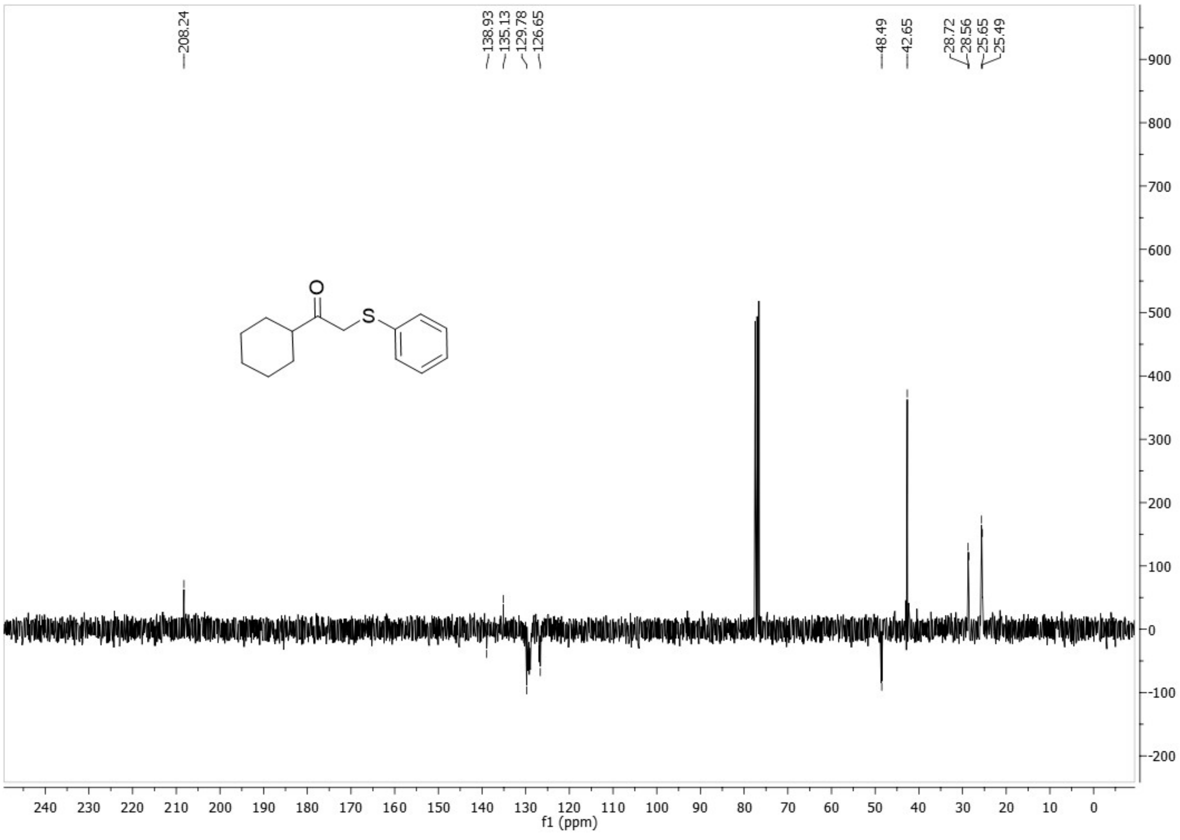

1-Cyclohexyl-2-(phenylthio)ethan-1-ol

^{1}H NMR (400 MHz, $CDCl_3$)

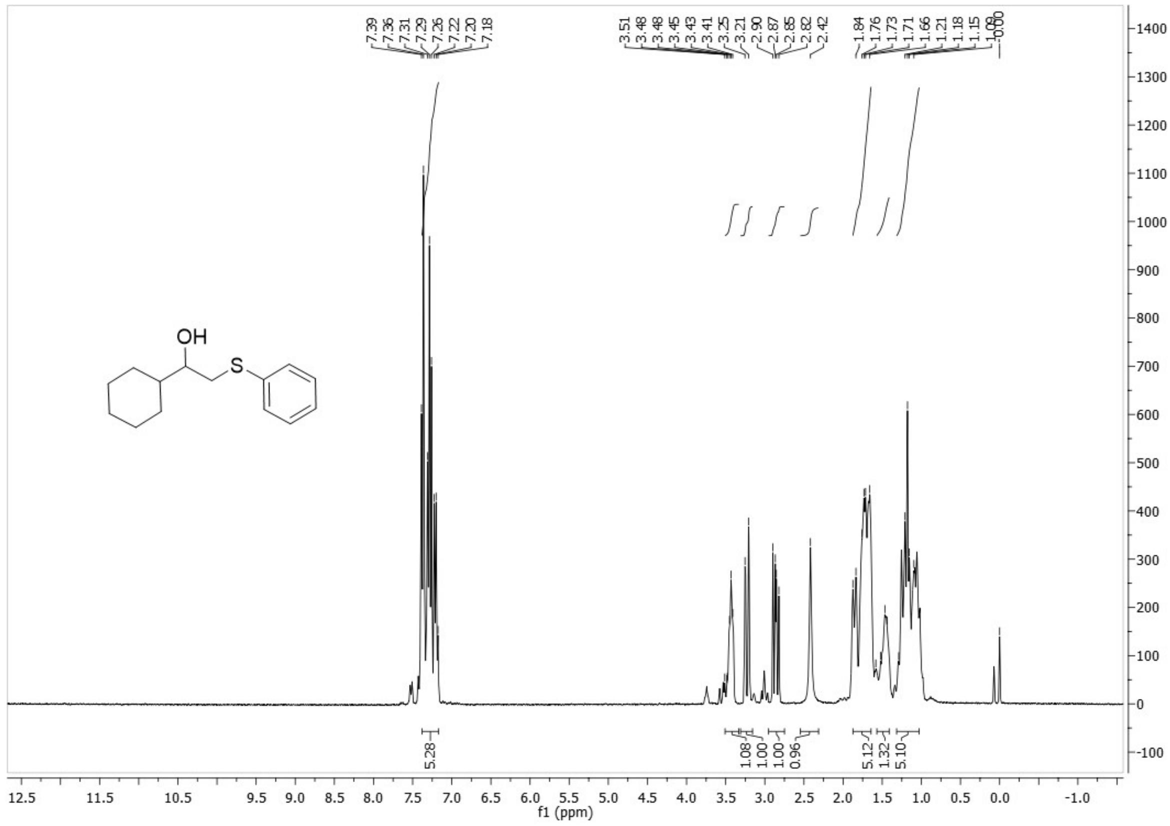

^{13}C NMR (101 MHz, $CDCl_3$

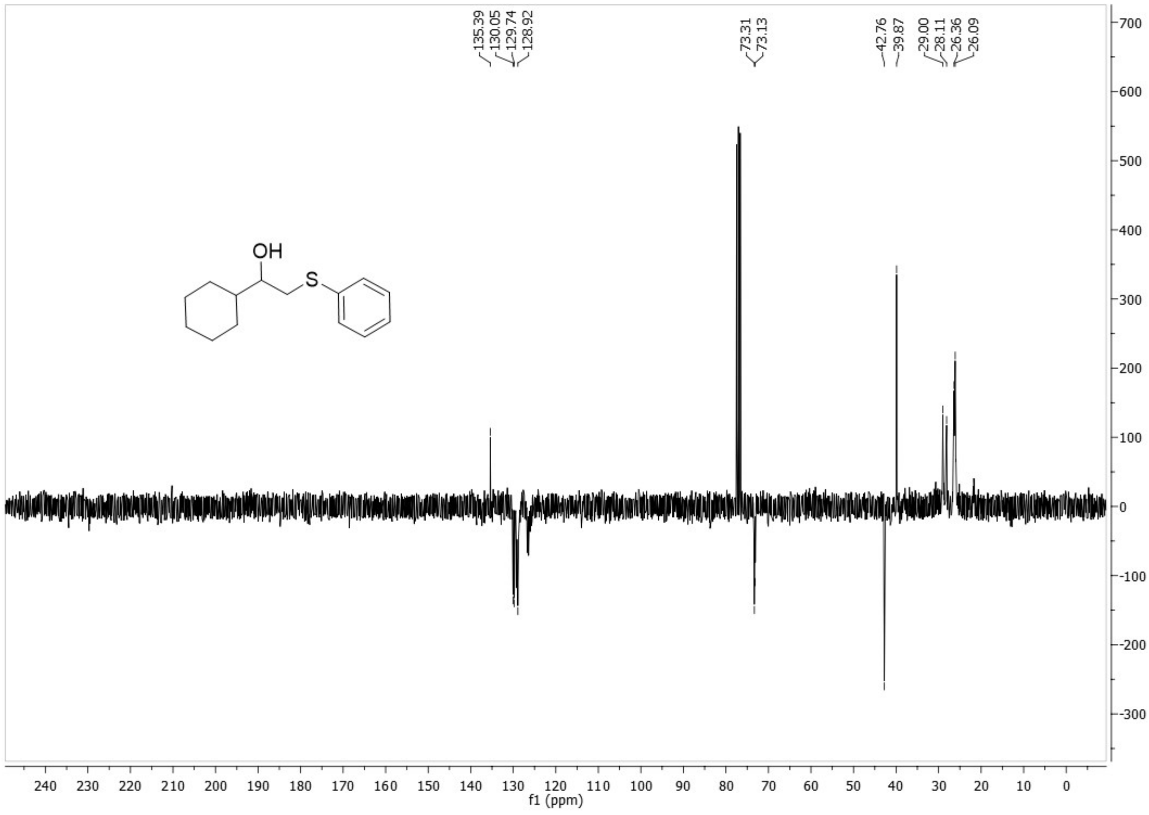

Racemic HPLC of 1-cyclohexyl-2-(phenylthio)ethan-1-ol.

08/02/2019 11:37 Chromatogram C:\Clarity\WORK1\DATA\V Vyas\VKV 53 ODH 930707 racemic dilute.prm Page 1 of 2

Clarity - Chromatography SW
DataApex 2006
www.dataapex.com

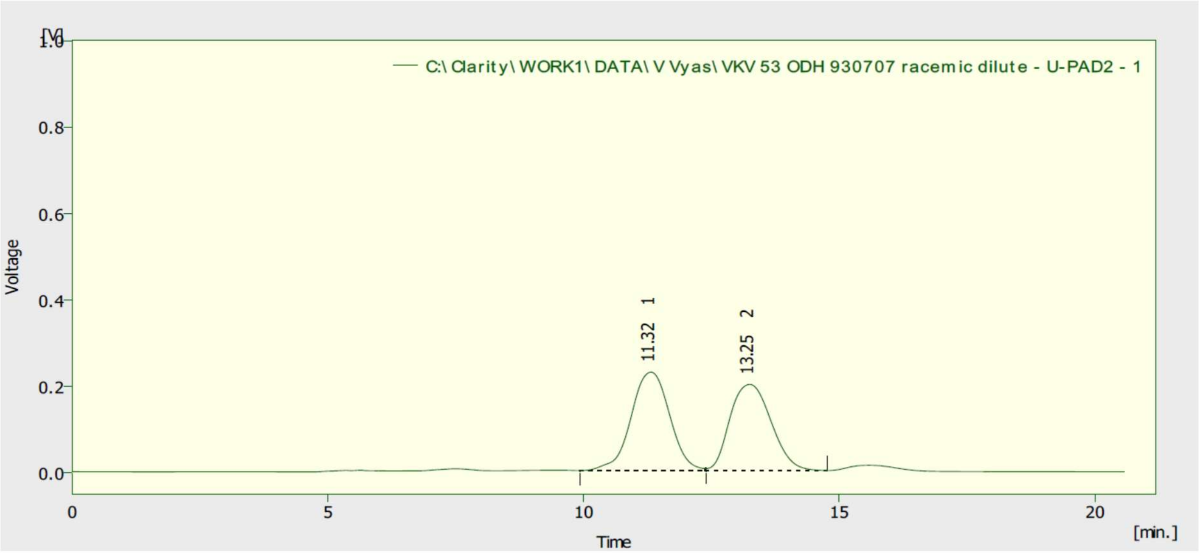

Result Table (Uncal - C:\Clarity\WORK1\DATA\V Vyas\VKV 53 ODH 930707 racemic dilute - U-PAD2 - 1)

	Reten. Time [min]	Area [mV.s]	Height [mV]	Area [%]	Height [%]	W05 [min]	Compound Name
1	11.316	11961.311	227.725	51.8	53.3	0.82	
2	13.248	11149.404	199.349	48.2	46.7	0.90	
	Total	23110.715	427.075	100.0	100.0		

HPLC after ATH 1-cyclohexyl-2-(phenylthio)ethan-1-ol. (100% conversion, 33.4% ee).

08/02/2019 11:36 Chromatogram C:\Clarity\WORK1\DATA\V Vyas\VKV 53 chiral ODH 930707.prm Page 1 of 2

Clarity - Chromatography SW
DataApex 2006
www.dataapex.com

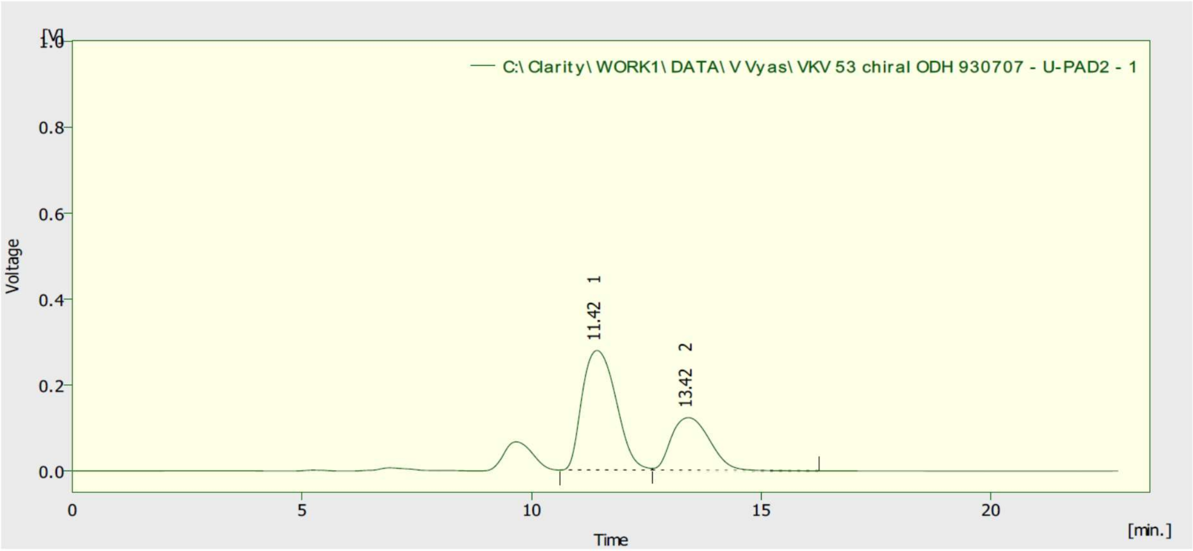

Result Table (Uncal - C:\Clarity\WORK1\DATA\V Vyas\VKV 53 chiral ODH 930707 - U-PAD2 - 1)

	Reten. Time [min]	Area [mV.s]	Height [mV]	Area [%]	Height [%]	W05 [min]	Compound Name
1	11.424	14363.955	278.654	66.7	69.4	0.84	
2	13.416	7174.037	123.059	33.3	30.6	0.94	
	Total	21537.993	401.713	100.0	100.0		

1-Cyclopropyl-2-(phenylthio)ethan-1-one

^{1}H NMR (400 MHz, $CDCl_3$)

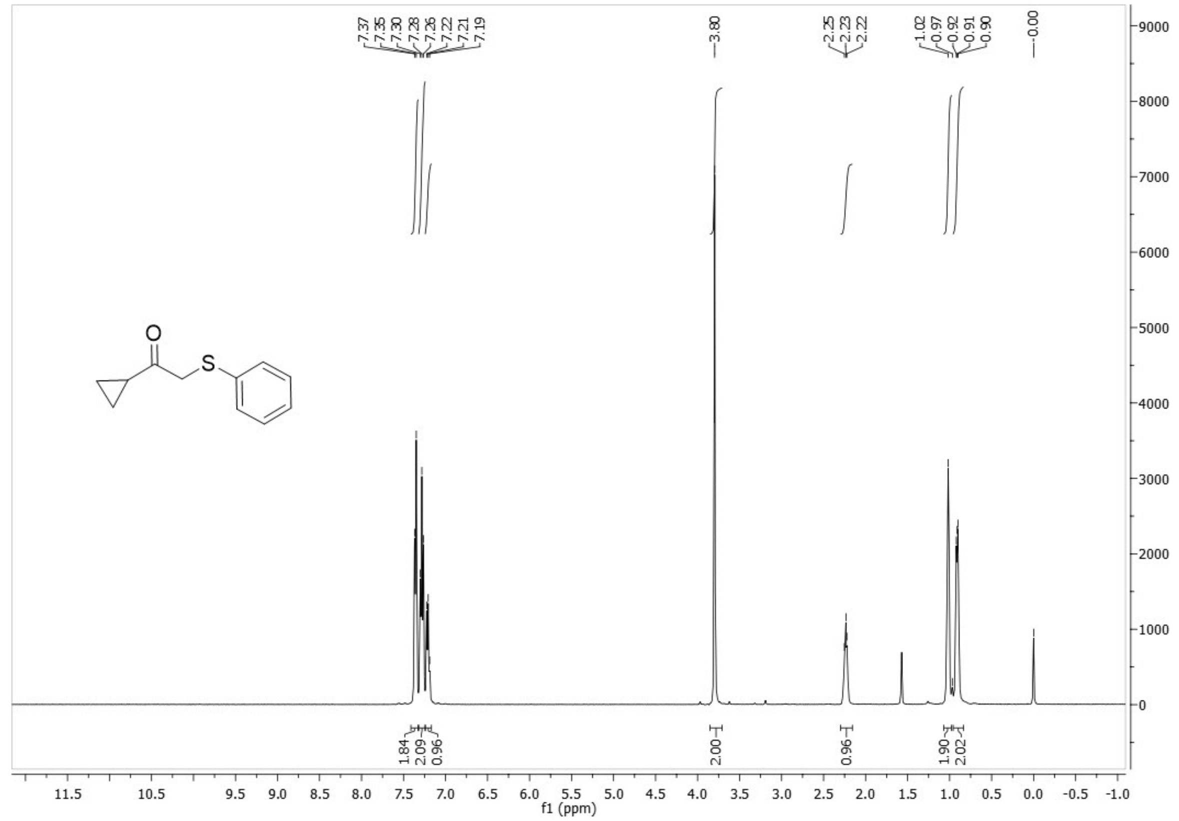

^{13}C NMR (101 MHz, $CDCl_3$

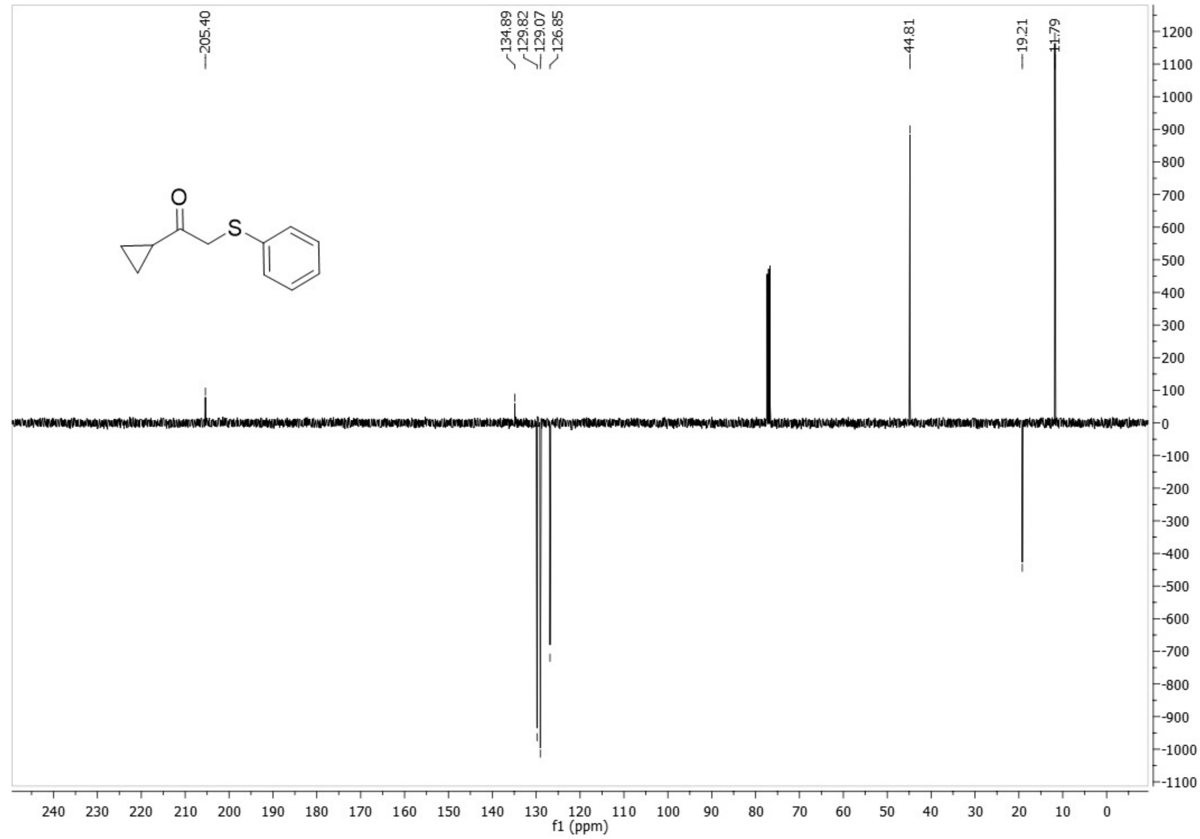

Ketone HPLC of 1-cyclopropyl-2-(phenylthio)ethan-1-one.

Clarity - Chromatography SW
DataApex 2006
www.dataapex.com

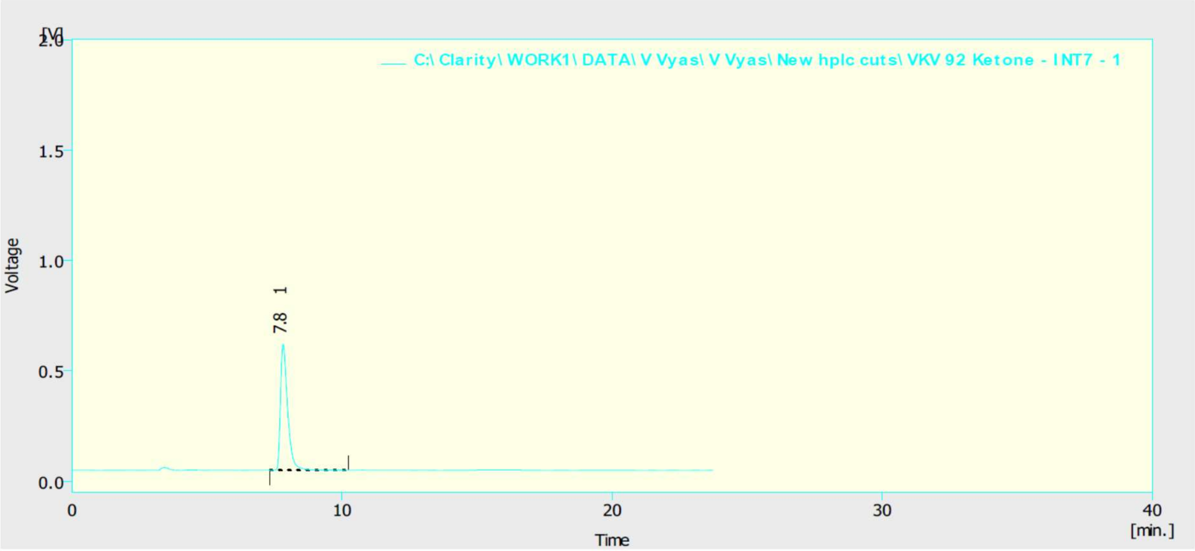

Result Table (Uncal - C:\Clarity\WORK1\DATA\V Vyas\V Vyas\New hplc cuts\VKV 92 Ketone - INT7 - 1)

	Reten. Time [min]	Area [mV.s]	Height [mV]	Area [%]	Height [%]	W05 [min]	Compound Name
1	7.817	10485.878	570.763	100.0	100.0	0.27	
	Total	10485.878	570.763	100.0	100.0		

1-Cyclopropyl-2-(phenylthio)ethan-1-ol

^{1}H NMR (400 MHz, $CDCl_3$)

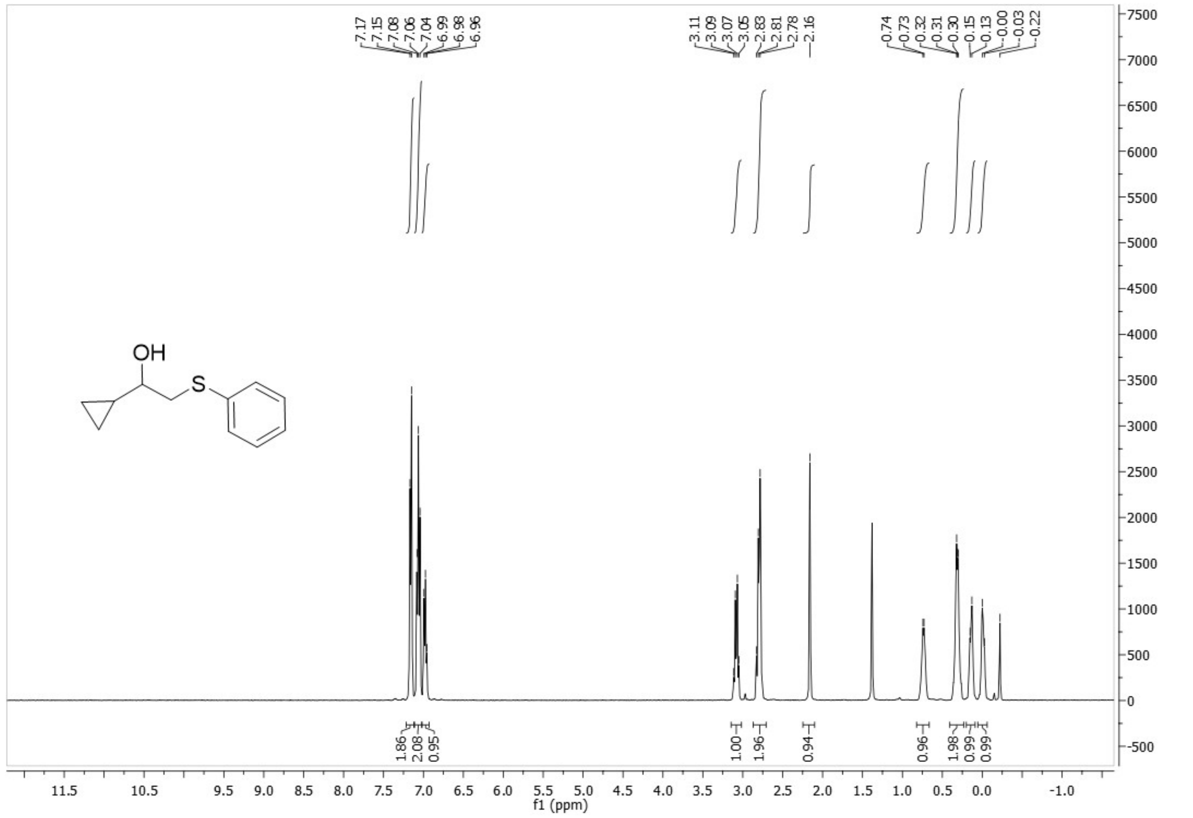

^{13}C NMR (101 MHz, $CDCl_3$

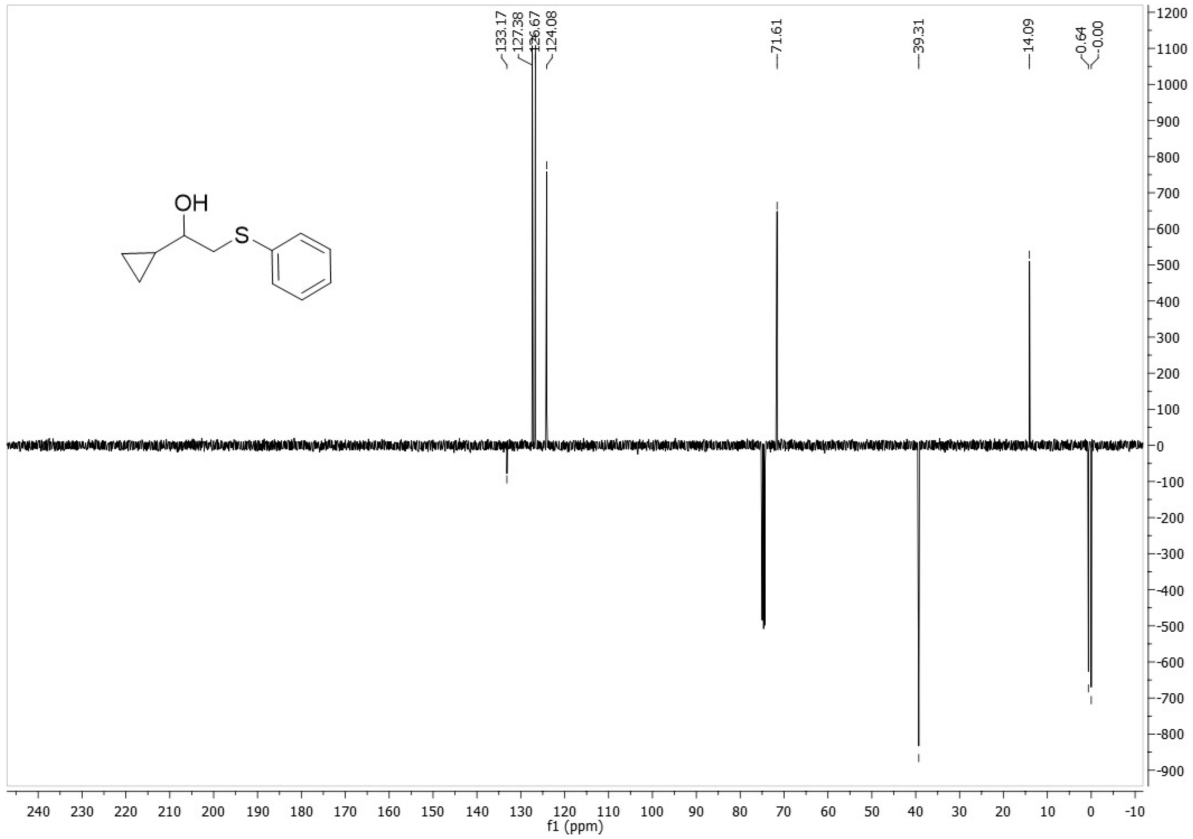

Racemic HPLC of 1-cyclopropyl-2-(phenylthio)ethan-1-ol.

07/03/2019 13:56 Chromatogram C:\Clarity\WORK1\DATA\V Vyas\VKV 98 racemiv 970307 AD.prm Page 1 of 2

Clarity - Chromatography SW
DataApex 2006
www.dataapex.com

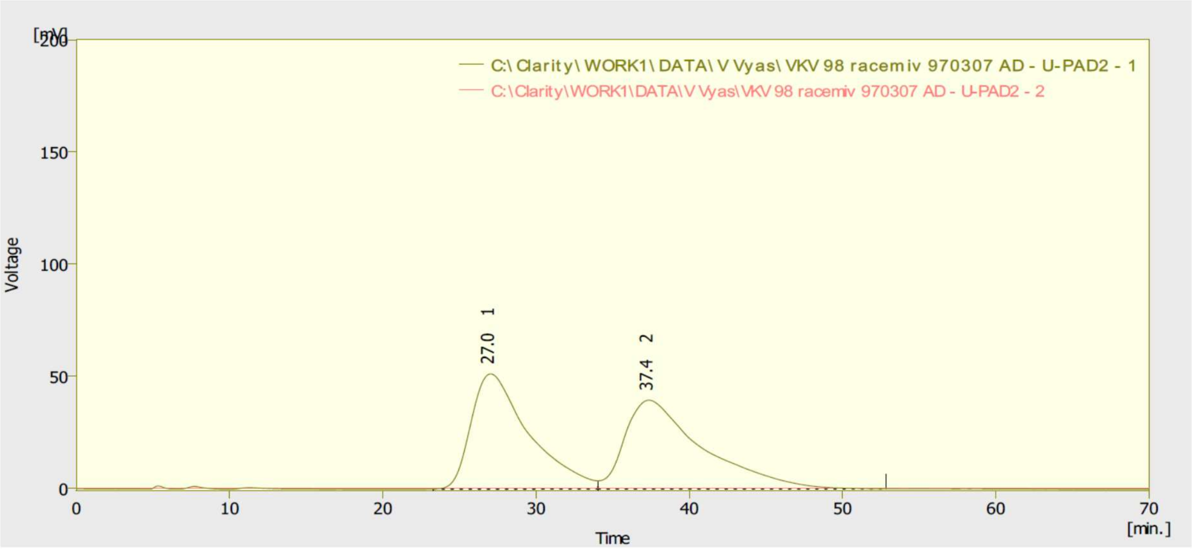

Result Table (Uncal - C:\Clarity\WORK1\DATA\V Vyas\VKV 98 racemiv 970307 AD - U-PAD2 - 1)

	Reten. Time [min]	Area [mV.s]	Height [mV]	Area [%]	Height [%]	W05 [min]	Compound Name
1	27.036	13410.759	51.266	49.2	56.5	3.78	
2	37.380	13841.019	39.524	50.8	43.5	4.90	
	Total	27251.779	90.791	100.0	100.0		

HPLC after ATH 1-cyclopropyl-2-(phenylthio)ethan-1-ol. (100% conversion, 87.2% ee).

07/03/2019 13:55 Chromatogram C:\Clarity\WORK1\DATA\V Vyas\VKV 99 ATH 970307AD.prm Page 1 of 2

Clarity - Chromatography SW
DataApex 2006
www.dataapex.com

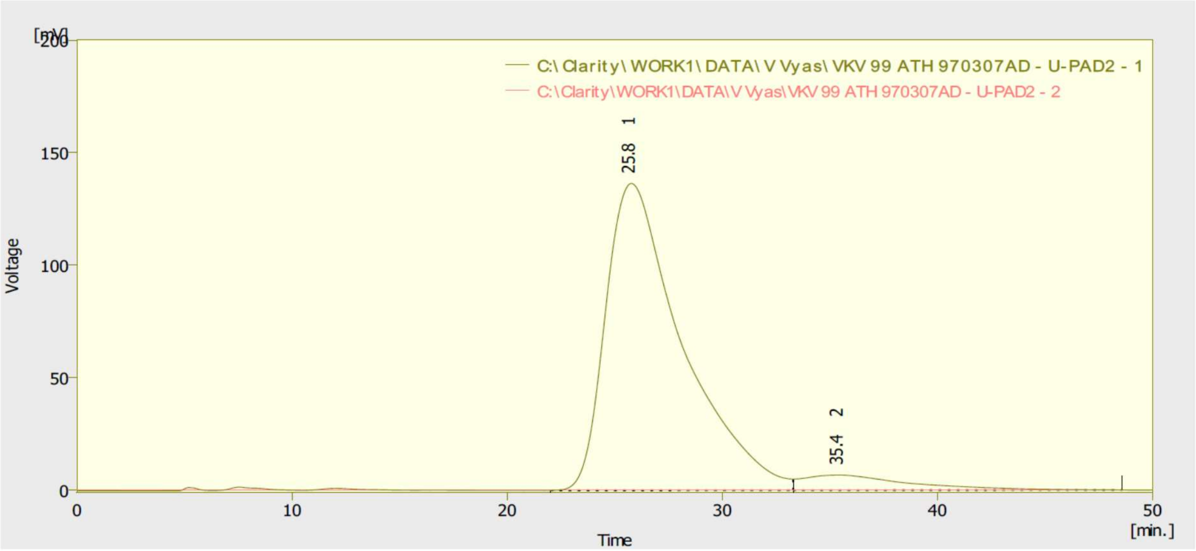

Result Table (Uncal - C:\Clarity\WORK1\DATA\V Vyas\VKV 99 ATH 970307AD - U-PAD2 - 1)

	Reten. Time [min]	Area [mV.s]	Height [mV]	Area [%]	Height [%]	W05 [min]	Compound Name
1	25.780	33698.574	136.333	93.6	95.3	3.55	
2	35.448	2313.247	6.731	6.4	4.7	5.10	
	Total	36011.821	143.063	100.0	100.0		

4-Phenyl-1-(phenylsulfonyl)but-3-yn-2-ol 11h
^{1}H NMR (400 MHz, $CDCl_3$)

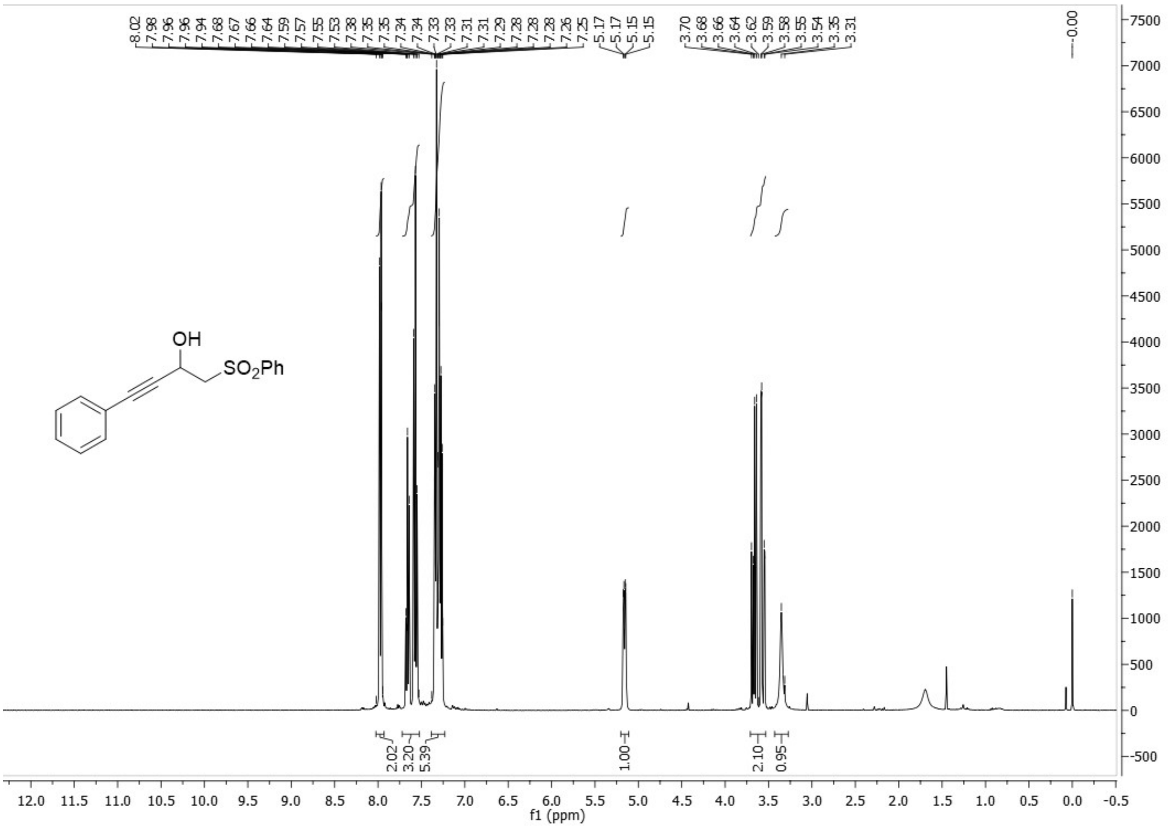

^{13}C NMR (101 MHz, $CDCl_3$)

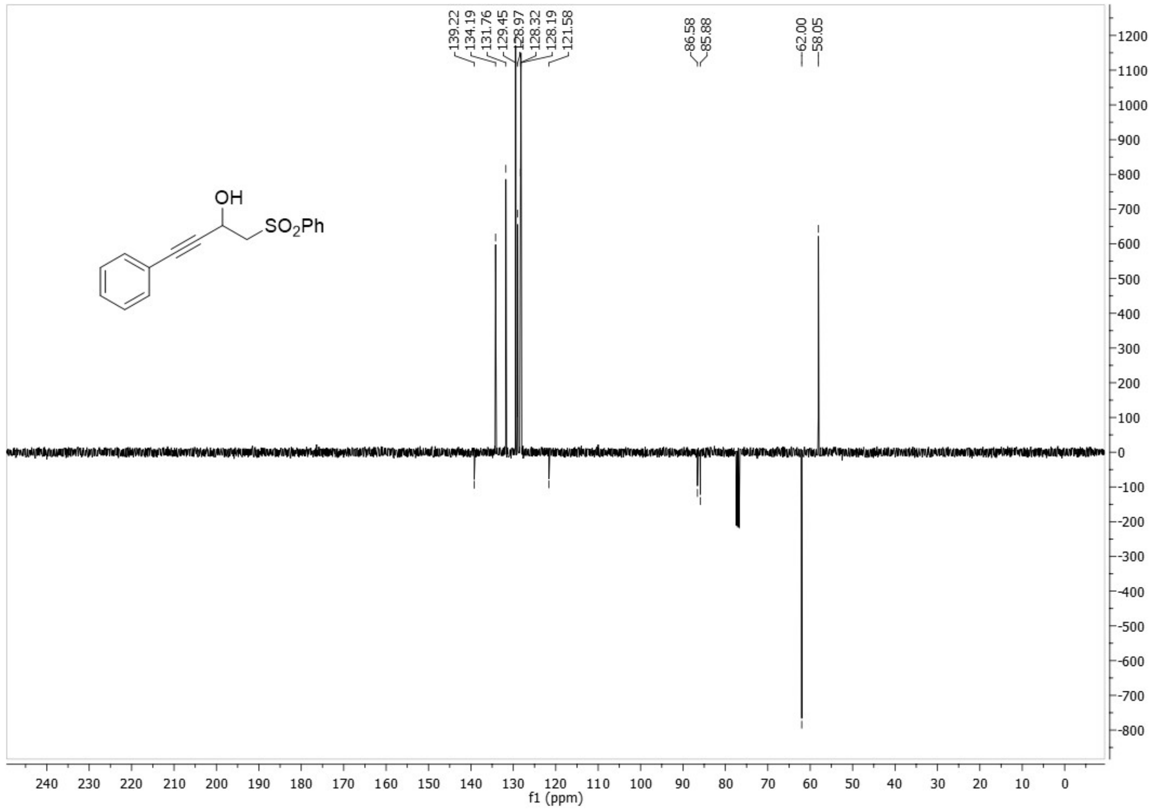

Racemic HPLC of 4-Phenyl-1-(phenylsulfonyl)but-3-yn-2-ol.

Clarity - Chromatography SW
DataApex 2006
www.dataapex.com

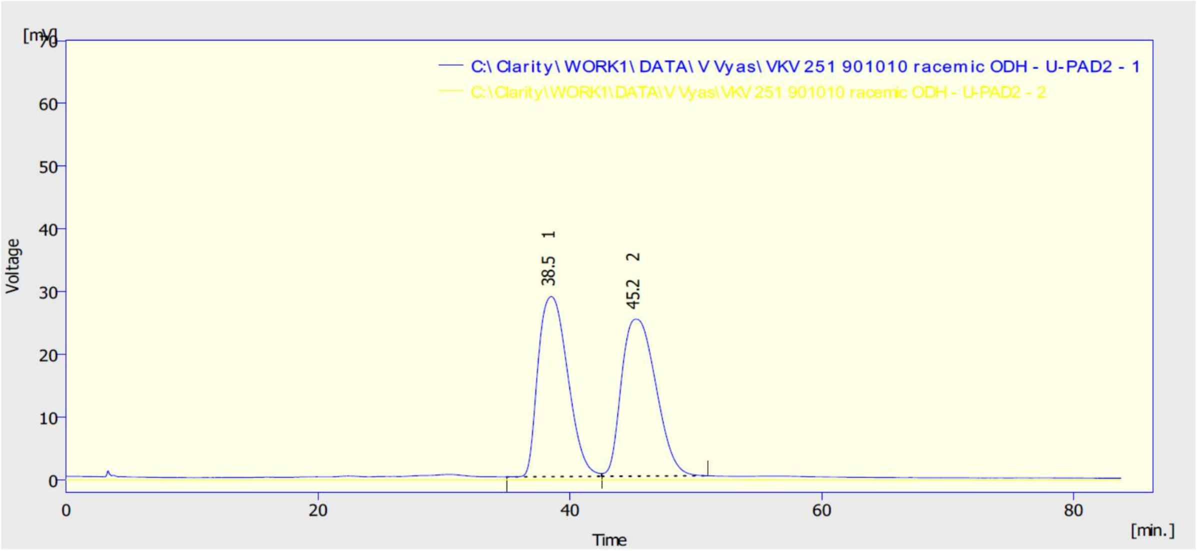

Result Table (Uncal - C:\Clarity\WORK1\DATA\V Vyas\VKV 251 901010 racemic ODH - U-PAD2 - 1)

	Reten. Time [min]	Area [mV.s]	Height [mV]	Area [%]	Height [%]	W05 [min]	Compound Name
1	38.524	4705.542	28.707	49.6	53.4	2.65	
2	45.248	4773.640	25.015	50.4	46.6	3.08	
	Total	9479.182	53.722	100.0	100.0		

HPLC after ATH 4-Phenyl-1-(phenylsulfonyl)but-3-yn-2-ol. (100% conversion, 99.4% ee).

Clarity - Chromatography SW
DataApex 2006
www.dataapex.com

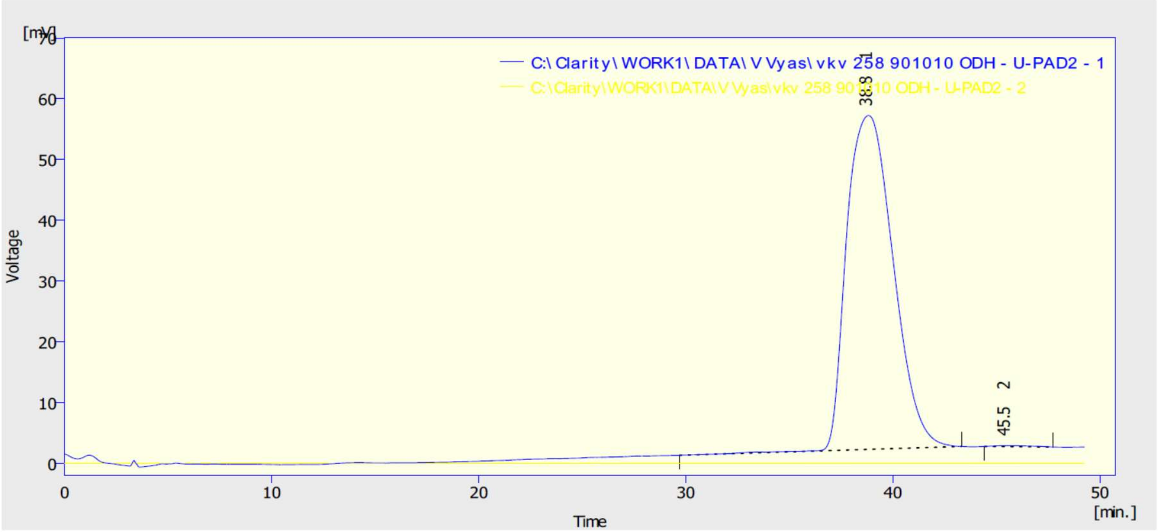

	Reten. Time [min]	Area [mV.s]	Height [mV]	Area [%]	Height [%]	W05 [min]	Compound Name
1	38.832	8583.786	54.962	99.7	99.7	2.53	
2	45.492	23.058	0.181	0.3	0.3	2.01	
	Total	8606.844	55.143	100.0	100.0		

4-Phenyl-1-(phenylsulfonyl)but-3-yn-2-one 10h

^{1}H NMR (400 MHz, $CDCl_3$)

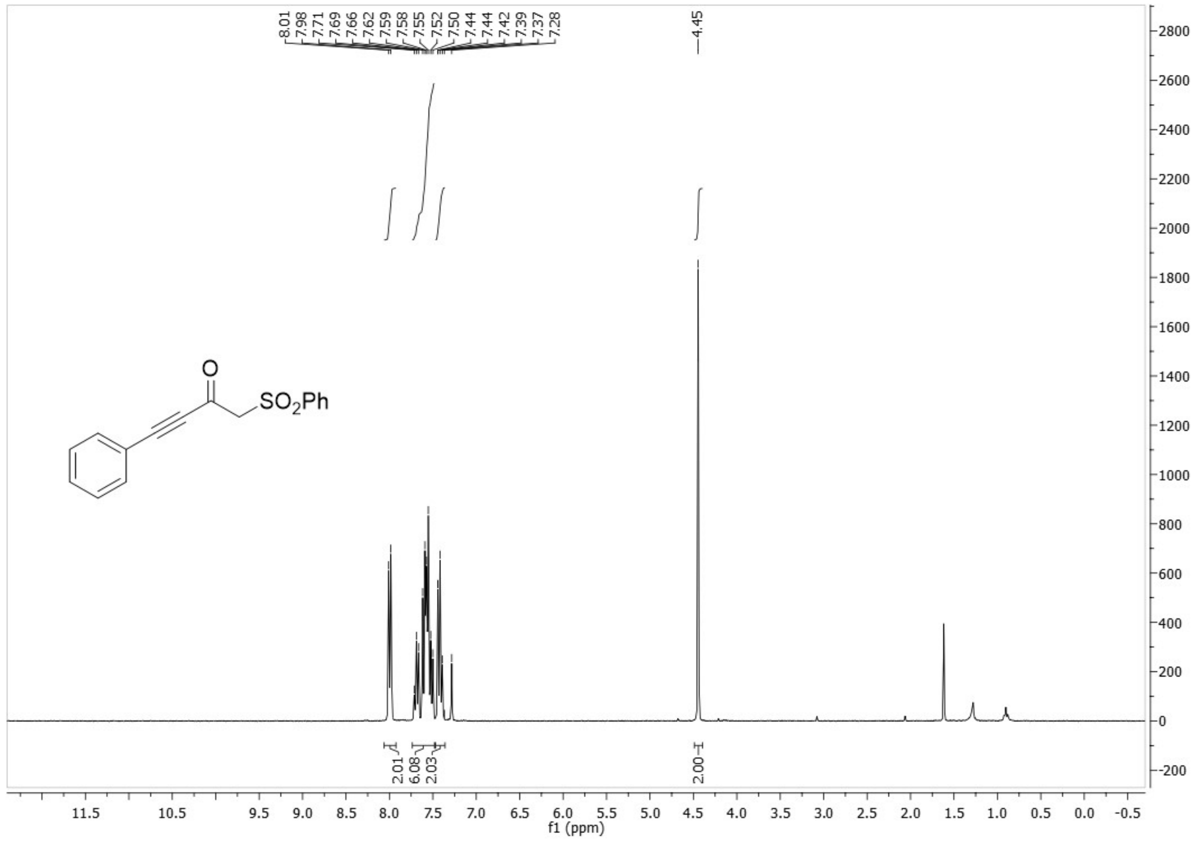

^{13}C NMR (101 MHz, $CDCl_3$)

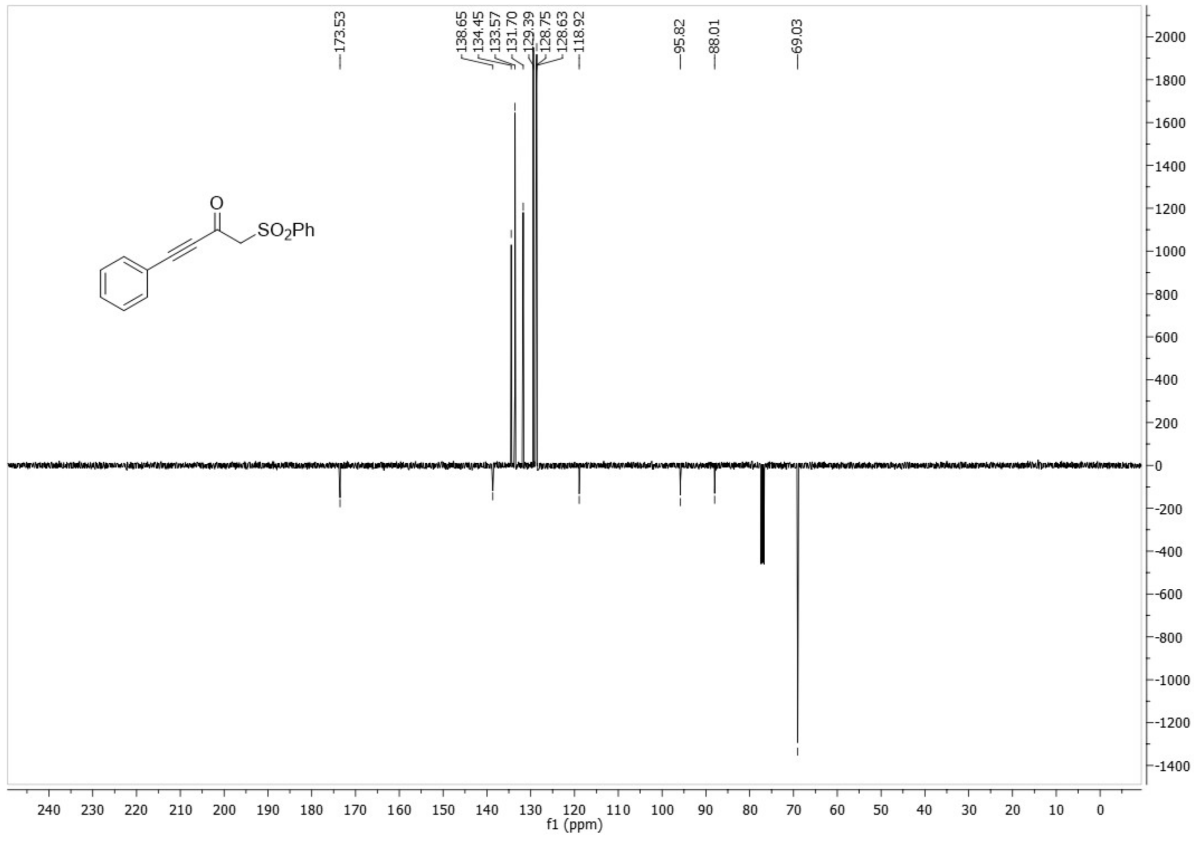

Ketone HPLC of 4-phenyl-1-(phenylsulfonyl)but-3-yn-2-one.

Clarity - Chromatography SW
DataApex 2006
www.dataapex.com

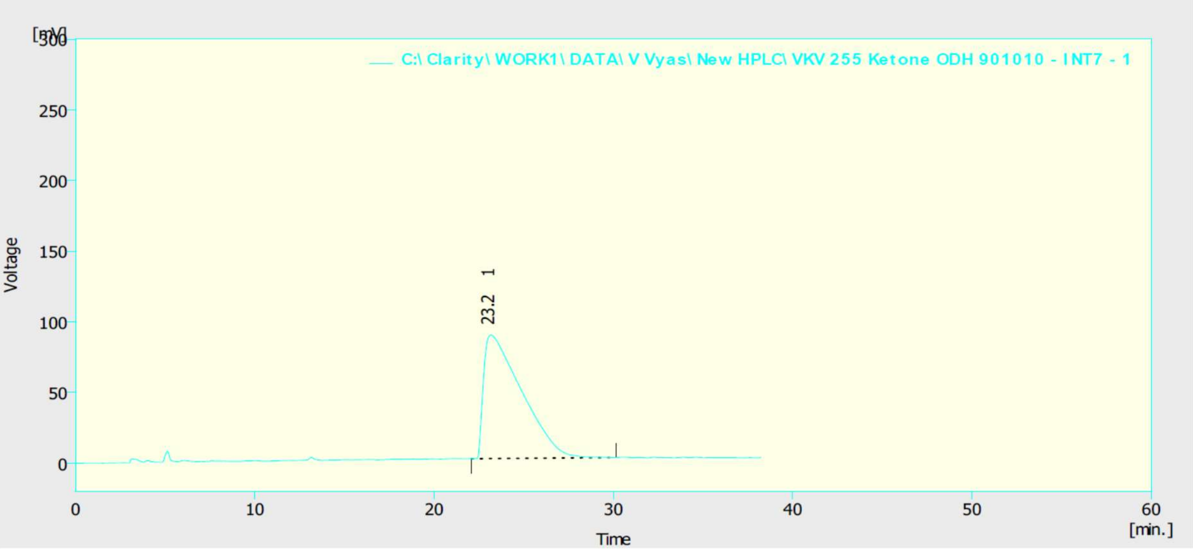

Result Table (Uncal - C:\Clarity\WORK1\DATA\V Vyas\New HPLC\VKV 255 Ketone ODH 901010 - INT7 - 1)

	Reten. Time [min]	Area [mV.s]	Height [mV]	Area [%]	Height [%]	W05 [min]	Compound Name
1	23.170	12919.778	87.420	100.0	100.0	2.37	
	Total	12919.778	87.420	100.0	100.0		

4-Phenyl-1-(phenylsulfonyl)but-3-yn-2-one

^{1}H NMR (400 MHz, $CDCl_3$)

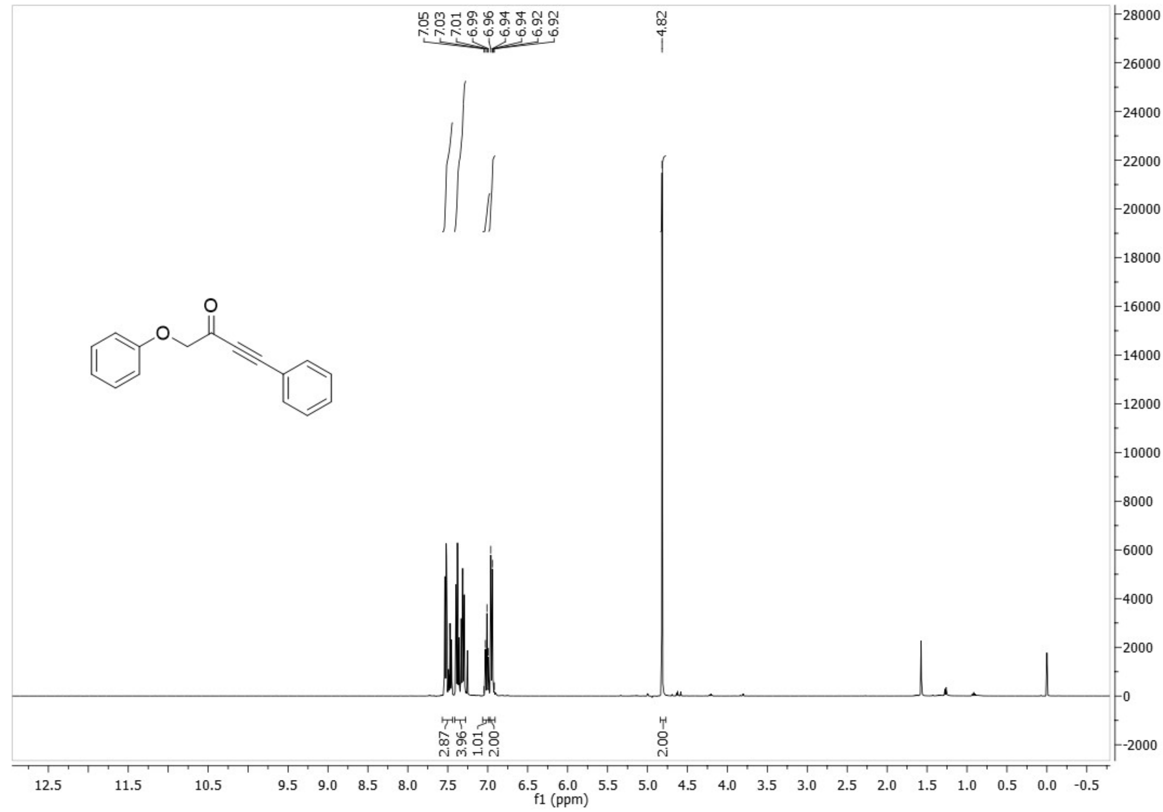

^{13}C NMR (101 MHz, $CDCl_3$)

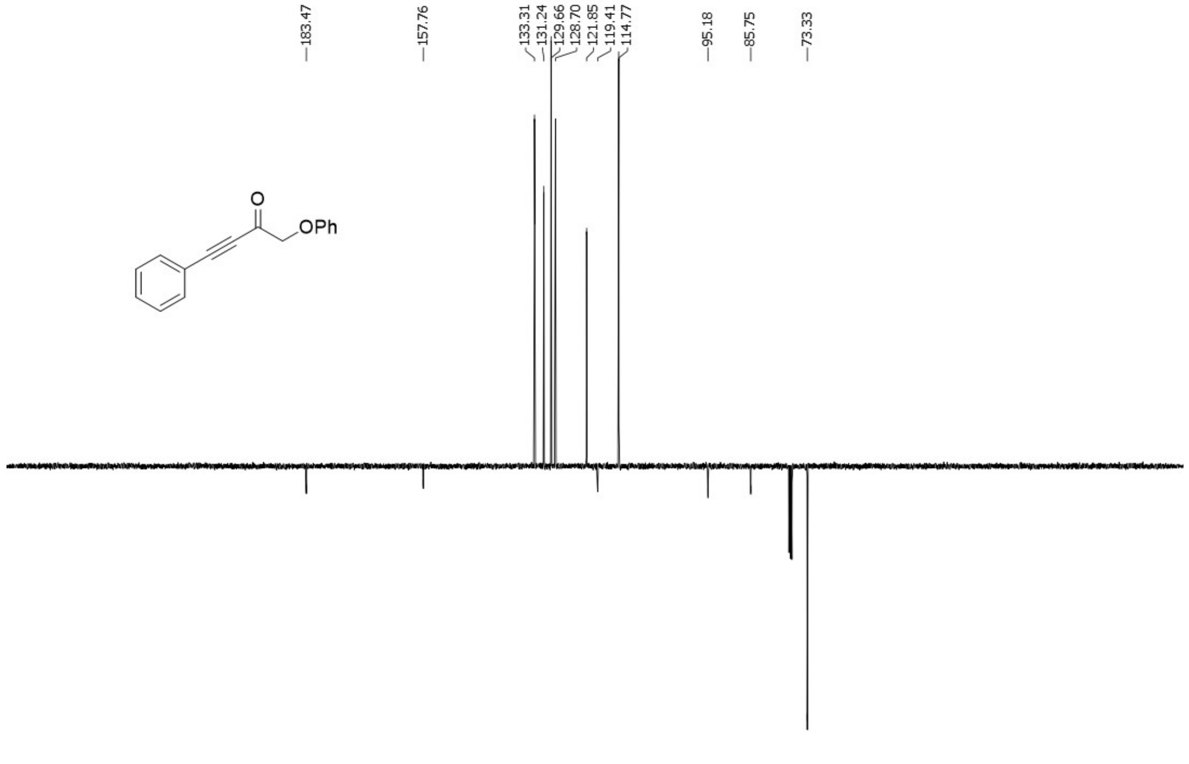

Ketone HPLC of 4-phenyl-1-(phenylsulfonyl)but-3-yn-2-one.

Clarity - Chromatography SW
DataApex 2006
www.dataapex.com

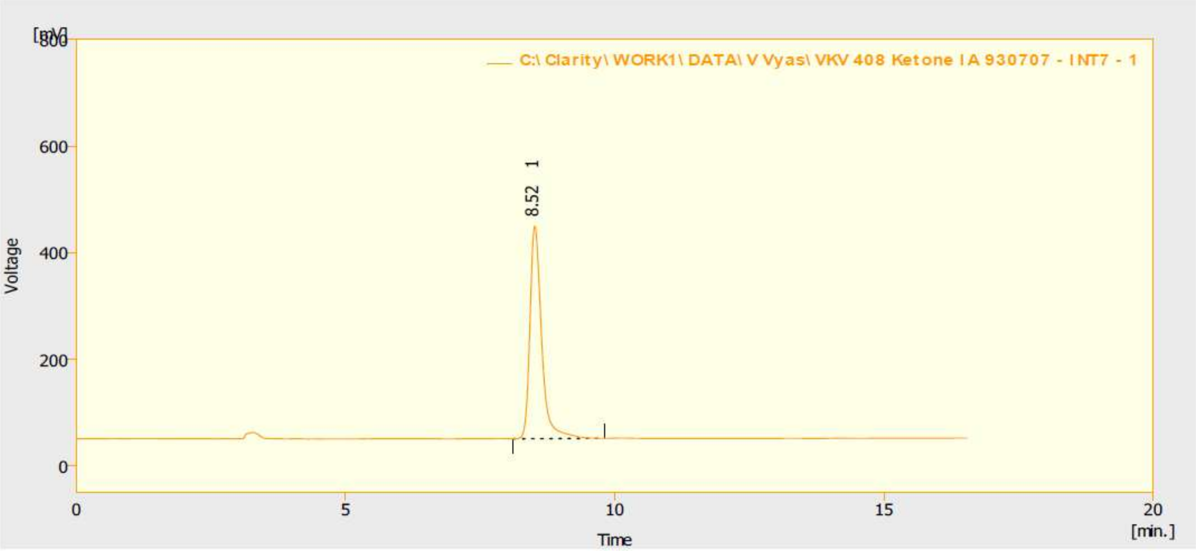

Result Table (Uncal - C:\Clarity\WORK1\DATA\V Vyas\VKV 408 Ketone IA 930707 - INT7 - 1)

	Reten. Time [min]	Area [mV.s]	Height [mV]	Area [%]	Height [%]	W05 [min]	Compound Name
1	8.517	6047.120	398.649	100.0	100.0	0.22	
	Total	6047.120	398.649	100.0	100.0		

1-Phenoxy-4-phenylbut-3-yn-2-ol

^{1}H NMR (400 MHz, $CDCl_3$)

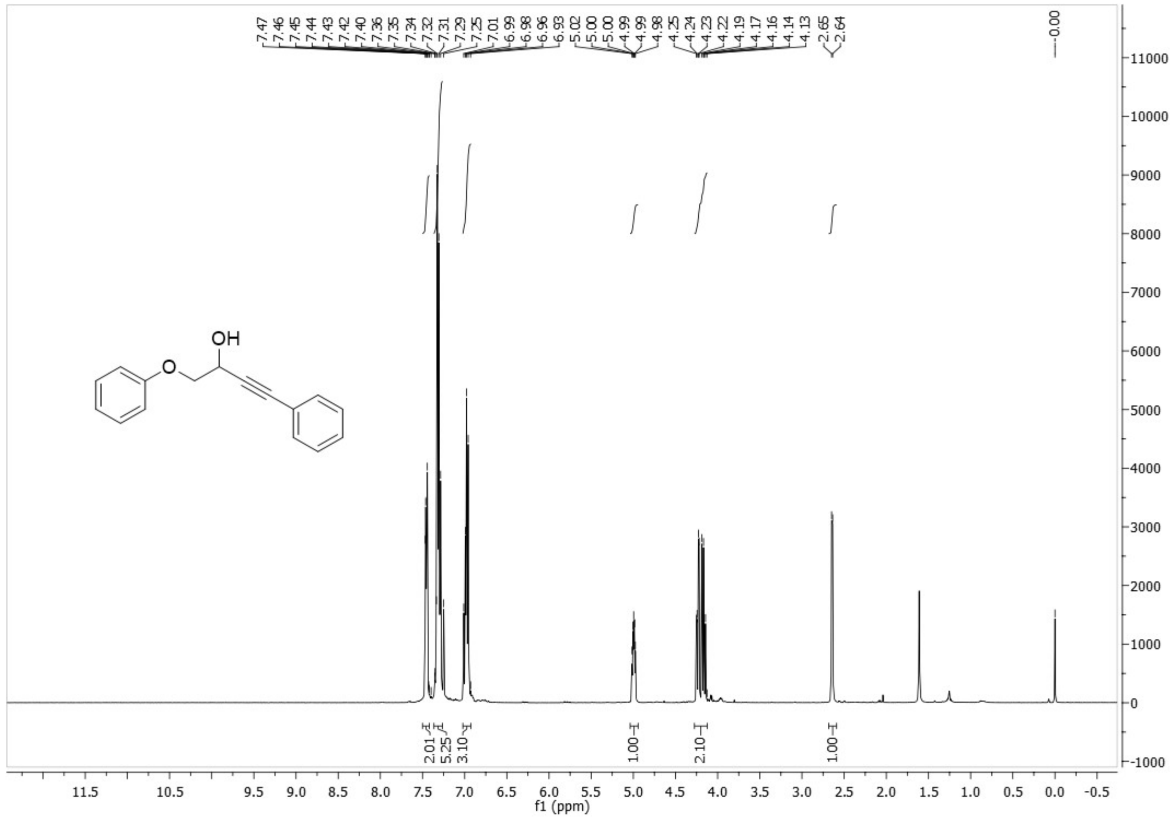

^{13}C NMR (101 MHz, $CDCl_3$)

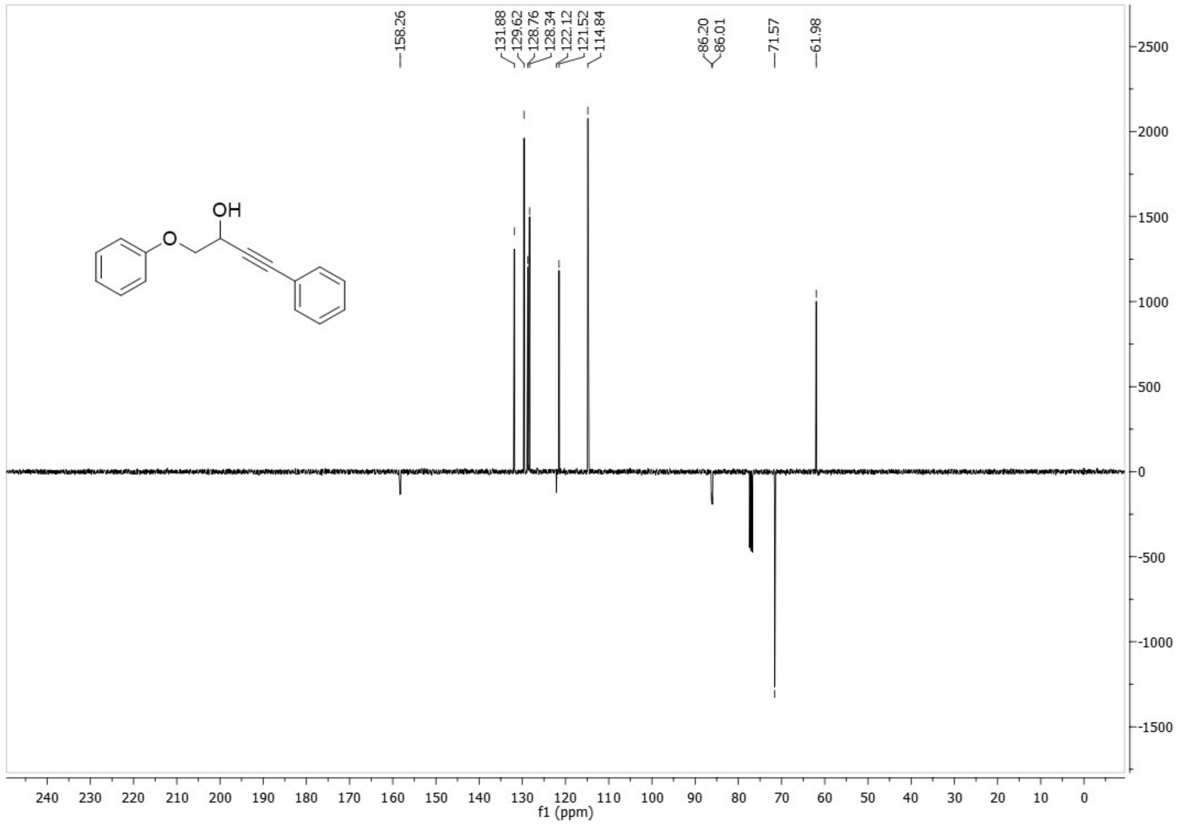

Racemic HPLC of 1-phenoxy-4-phenylbut-3-yn-2-ol.

Clarity - Chromatography SW
DataApex 2006
www.dataapex.com

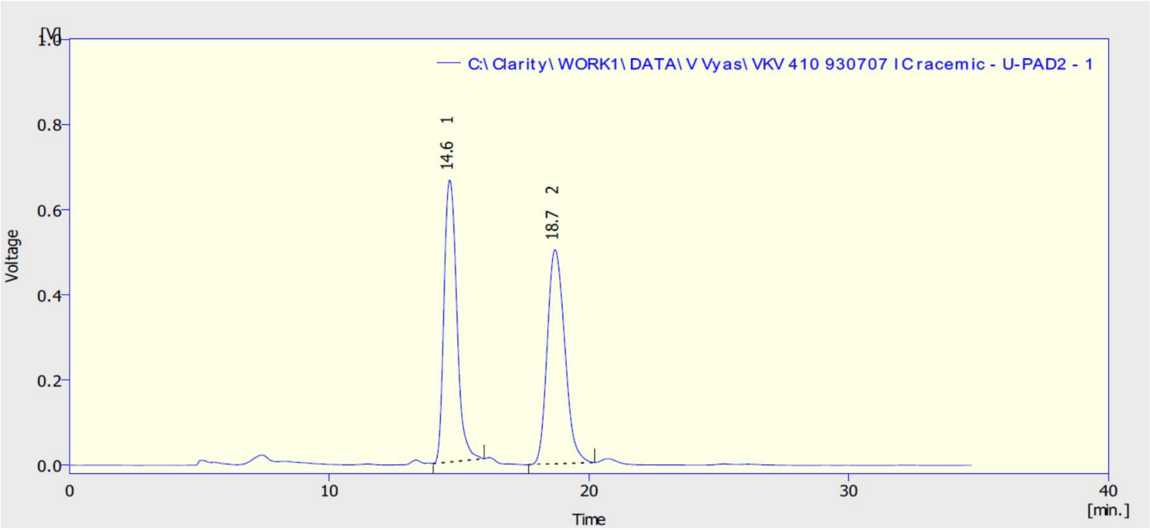

Result Table (Uncal - C:\Clarity\WORK1\DATA\V Vyas\VKV 410 930707 IC racemic - U-PAD2 - 1)

	Reten. Time [min]	Area [mV.s]	Height [mV]	Area [%]	Height [%]	W05 [min]	Compound Name
1	14.640	22700.711	661.752	49.0	56.8	0.53	
2	18.692	23591.928	502.880	51.0	43.2	0.74	
	Total	46292.639	1164.632	100.0	100.0		

HPLC after ATH 1-phenoxy-4-phenylbut-3-yn-2-ol. (100% conversion, 89% ee).

Clarity - Chromatography SW
DataApex 2006
www.dataapex.com

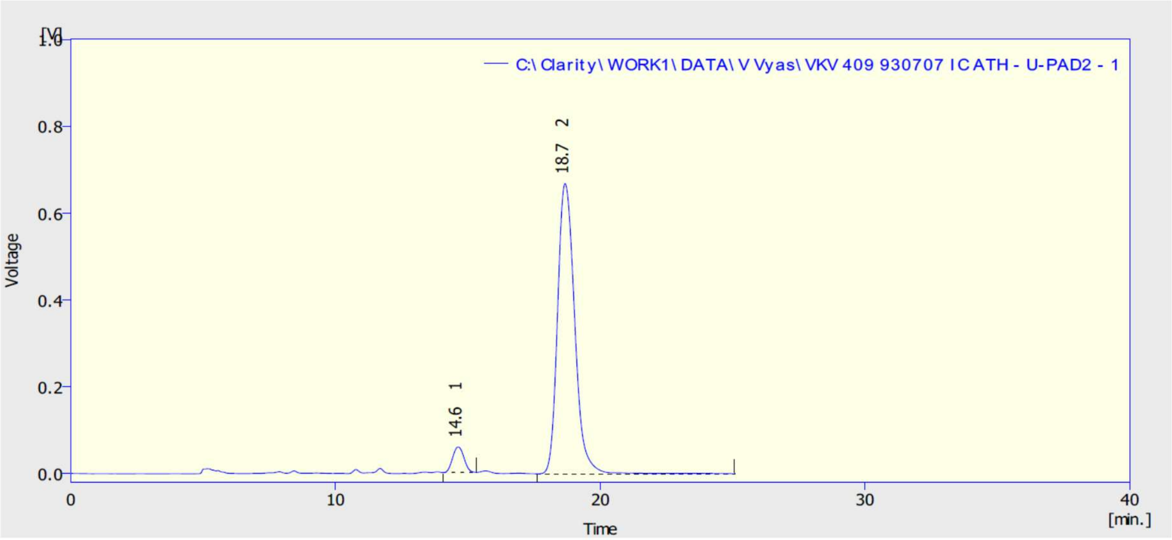

Result Table (Uncal - C:\Clarity\WORK1\DATA\V Vyas\VKV 409 930707 IC ATH - U-PAD2 - 1)

	Reten. Time [min]	Area [mV.s]	Height [mV]	Area [%]	Height [%]	W05 [min]	Compound Name
1	14.636	1759.845	59.004	5.5	8.1	0.48	
2	18.672	30100.624	668.367	94.5	91.9	0.69	
	Total	31860.469	727.371	100.0	100.0		

1-Phenyl-3-(phenylsulfonyl)propan-2-one 10i

^{1}H NMR (400 MHz, $CDCl_3$)

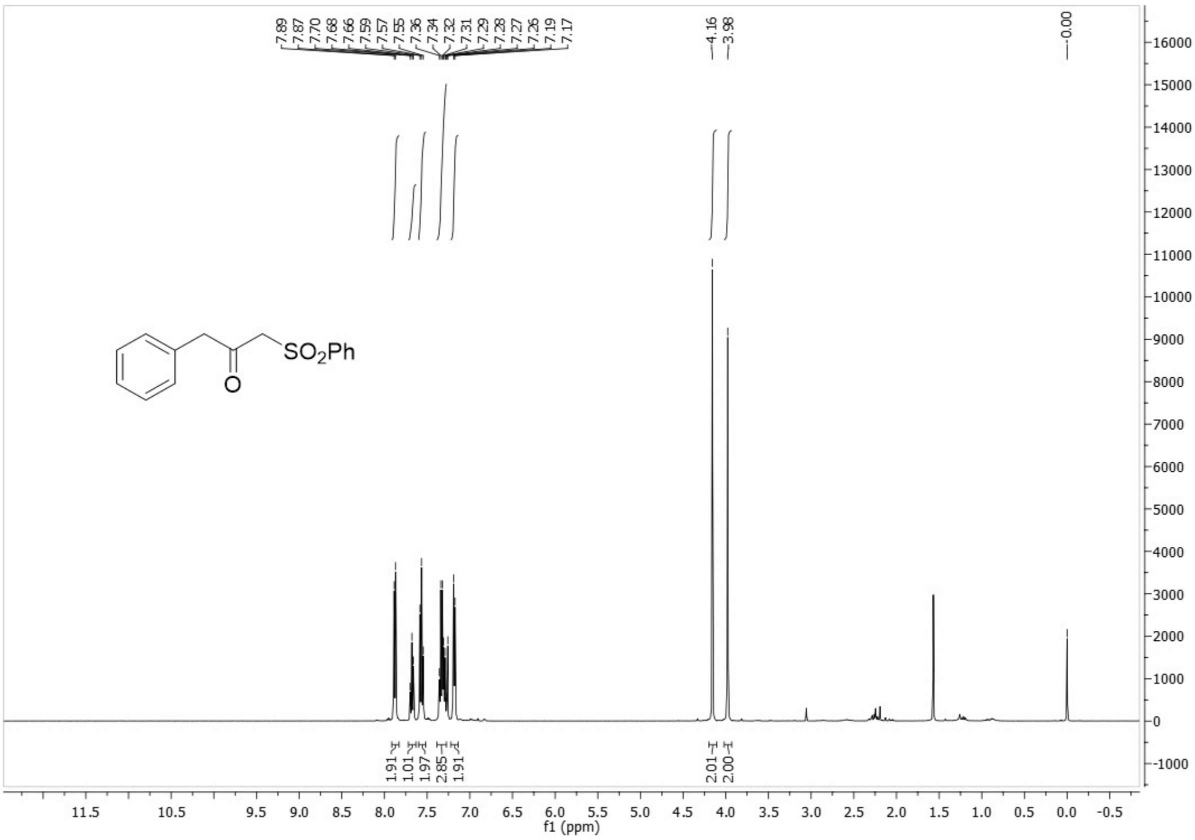

^{13}C NMR (101 MHz, $CDCl_3$)

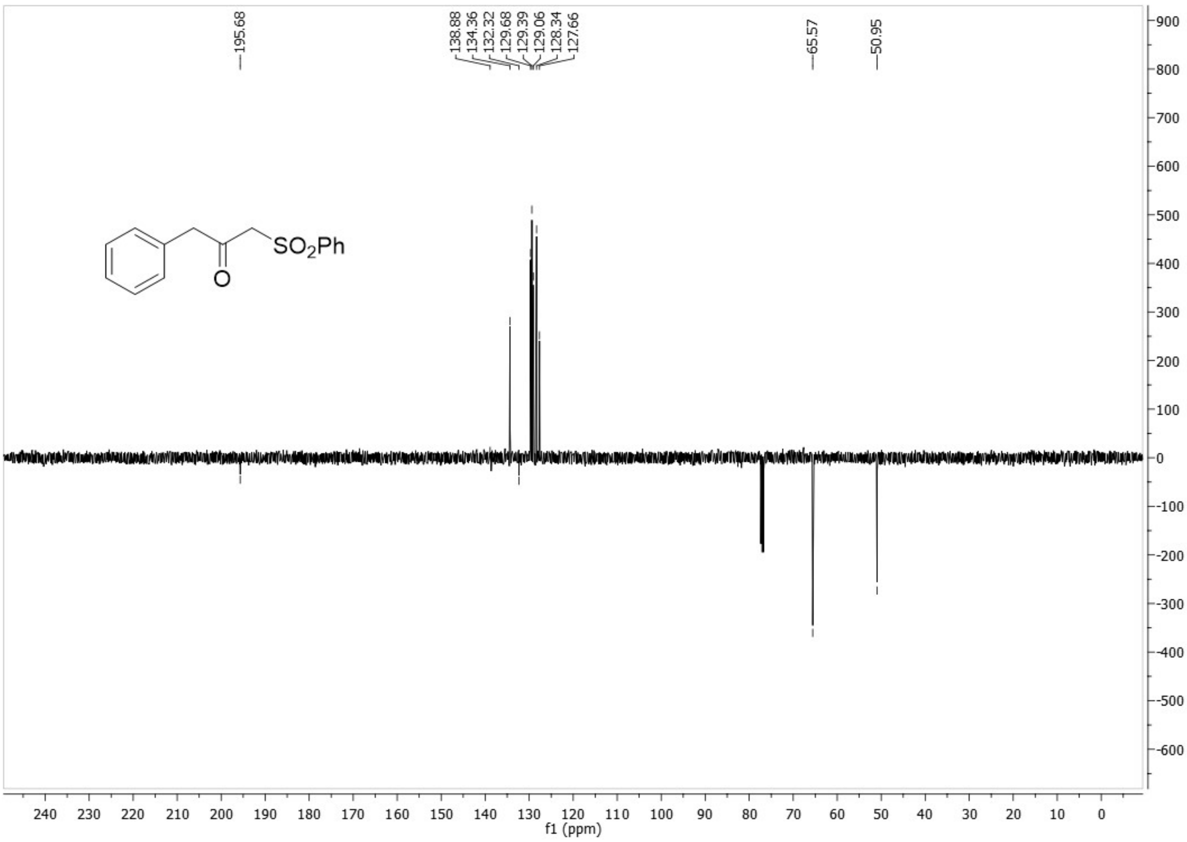

Ketone HPLC of 1-phenyl-3-(phenylsulfonyl)propan-2-one.

Clarity - Chromatography SW

DataApex 2006

www.dataapex.com

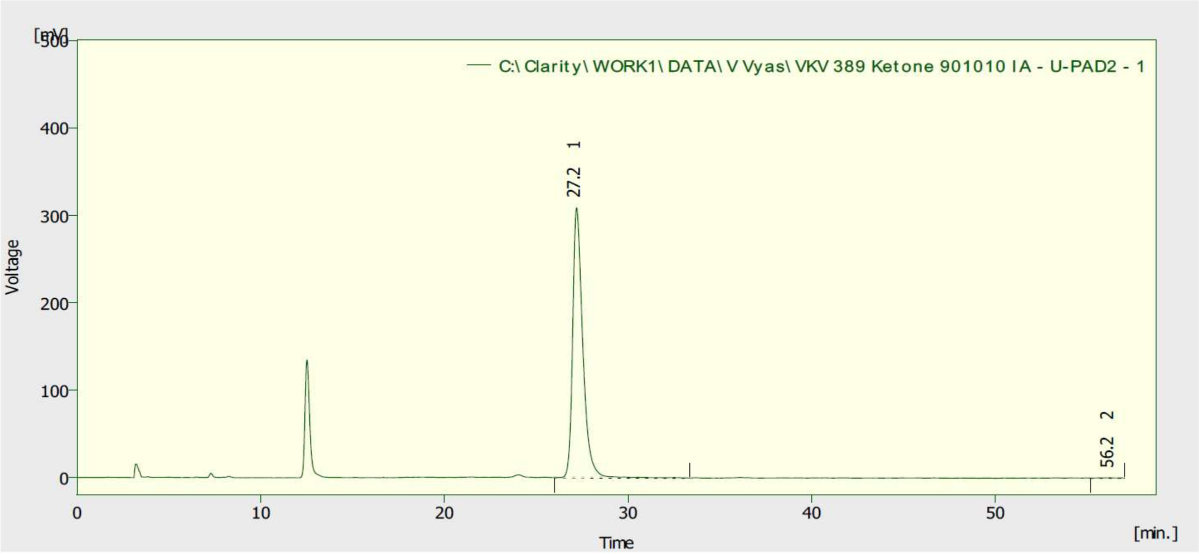

	Reten. Time [min]	Area [mV.s]	Height [mV]	Area [%]	Height [%]	W05 [min]	Compound Name
1	27.200	11866.362	308.672	99.9	99.9	0.56	
2	56.236	15.979	0.368	0.1	0.1	0.35	
	Total	11882.341	309.040	100.0	100.0		

1-Phenyl-3-(phenylsulfonyl)propan-2-ol 11i

^{1}H NMR (400 MHz, $CDCl_3$)

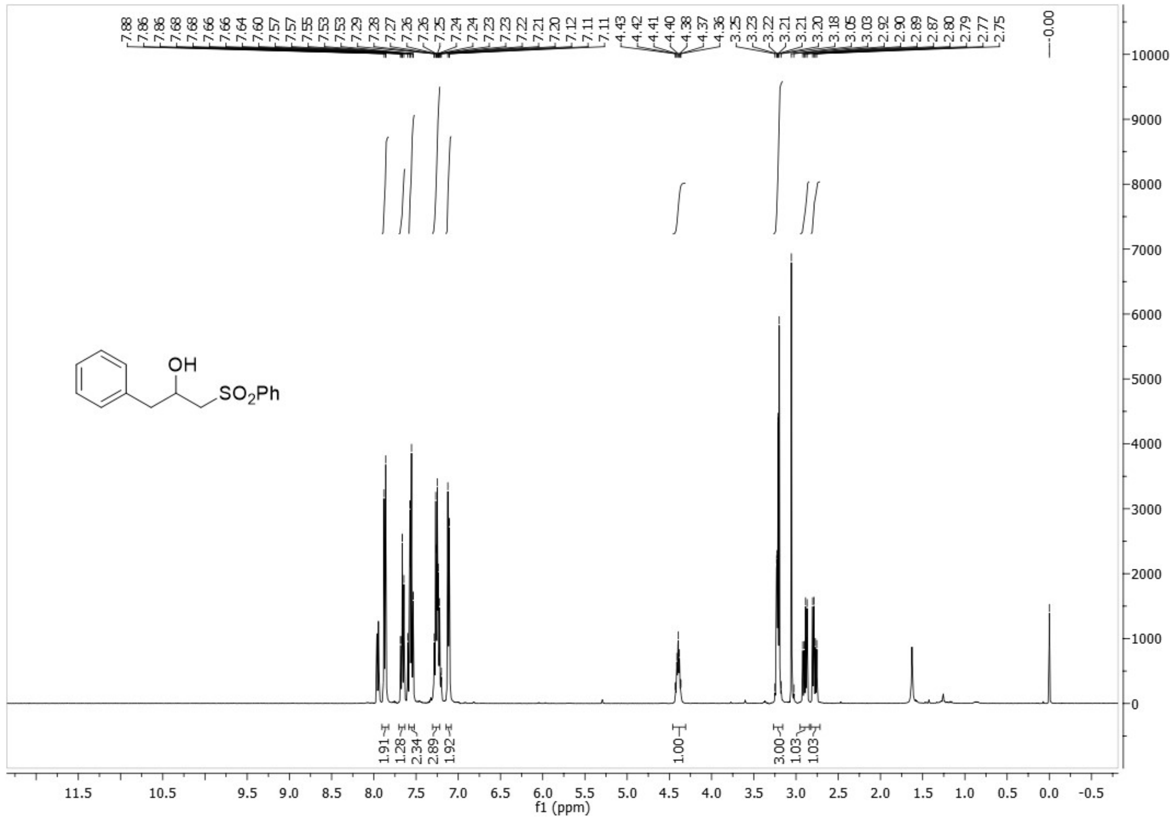

^{13}C NMR (101 MHz, $CDCl_3$)

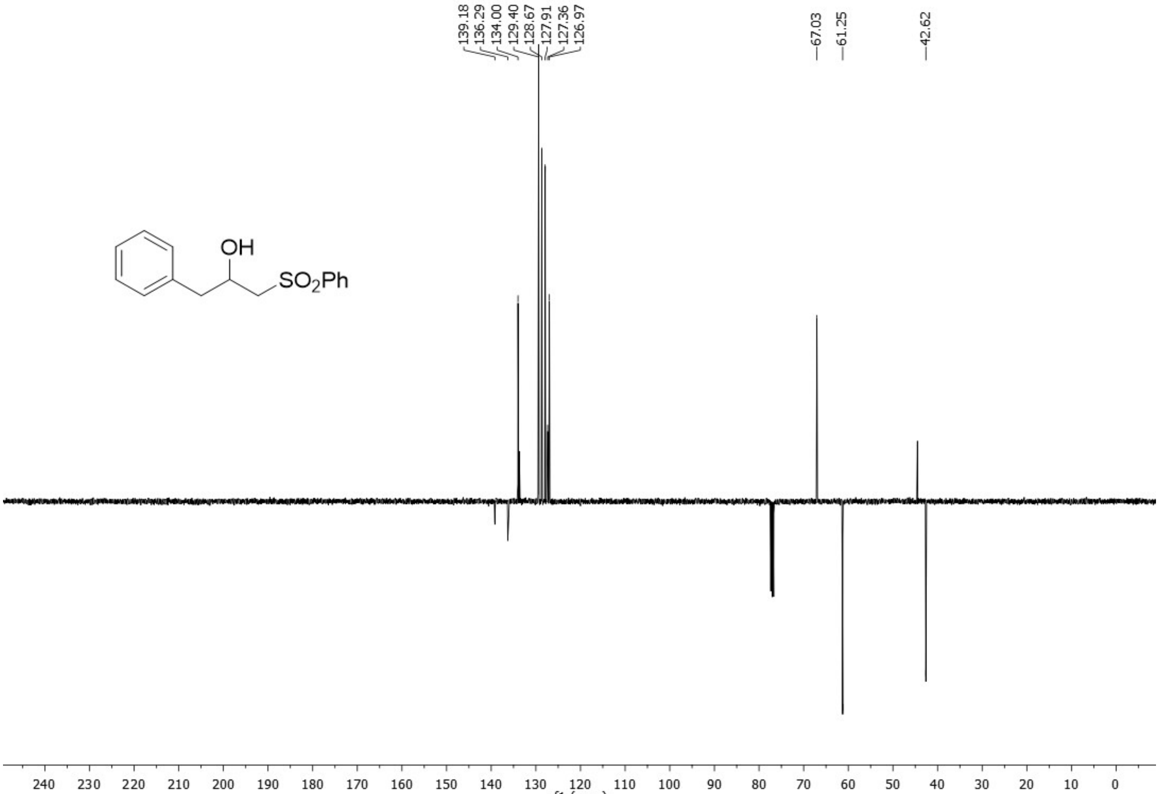

Racemic HPLC of 1-phenyl-3-(phenylsulfonyl)propan-2-one.

Clarity - Chromatography SW
DataApex 2006
www.dataapex.com

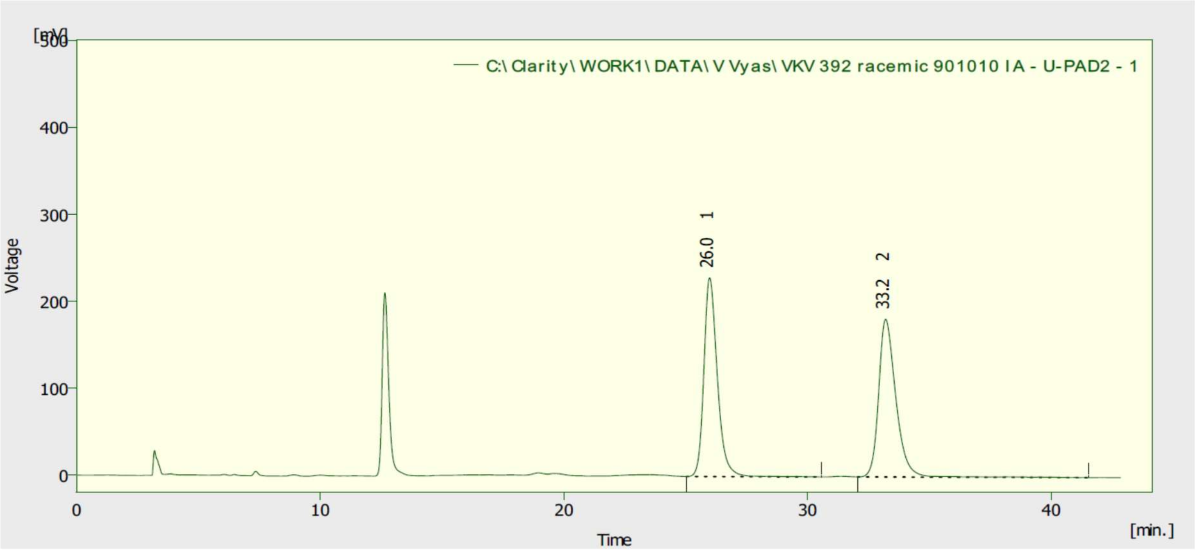

Result Table (Uncal - C:\Clarity\WORK1\DATA\V Vyas\VKV 392 racemic 901010 IA - U-PAD2 - 1)

	Reten. Time [min]	Area [mV.s]	Height [mV]	Area [%]	Height [%]	W05 [min]	Compound Name
1	25.984	8788.163	228.648	49.4	55.8	0.57	
2	33.208	8992.230	181.429	50.6	44.2	0.73	
	Total	17780.394	410.077	100.0	100.0		

HPLC after ATH 1-phenyl-3-(phenylsulfonyl)propan-2-one. (100% conversion, 72% ee).

Clarity - Chromatography SW
DataApex 2006
www.dataapex.com

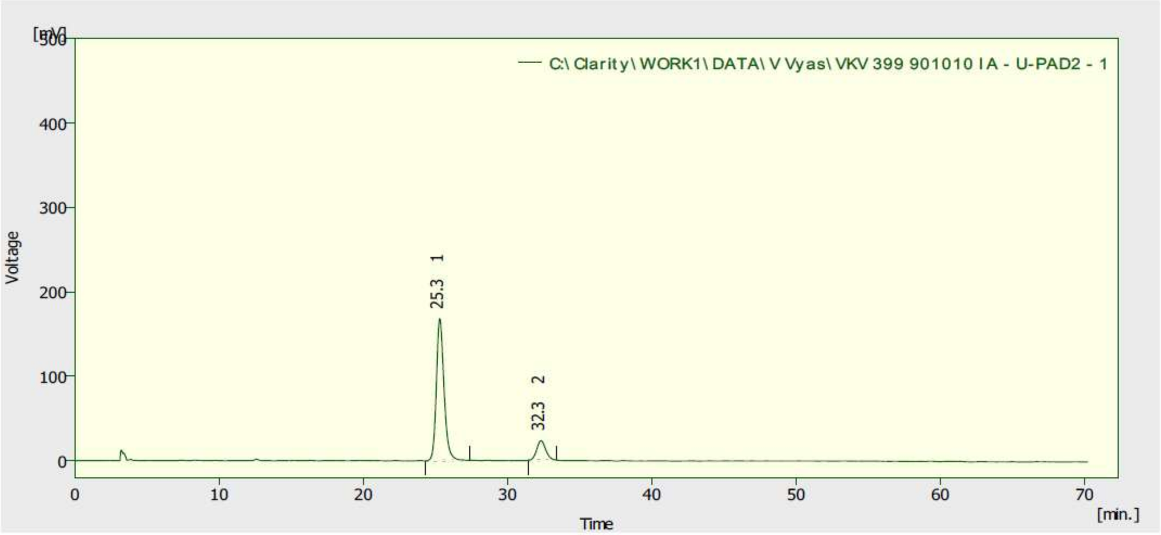

Result Table (Uncal - C:\Clarity\WORK1\DATA\V Vyas\VKV 399 901010 IA - U-PAD2 - 1)

	Reten. Time [min]	Area [mV.s]	Height [mV]	Area [%]	Height [%]	W05 [min]	Compound Name
1	25.304	6247.855	168.289	86.0	87.9	0.56	
2	32.316	1020.034	23.216	14.0	12.1	0.68	
	Total	7267.890	191.504	100.0	100.0		

***tert*-Butyl 2-(2-(phenylsulfonyl)acetyl)cyclopropane-1-carboxylate**

^{1}H NMR (400 MHz, $CDCl_3$)

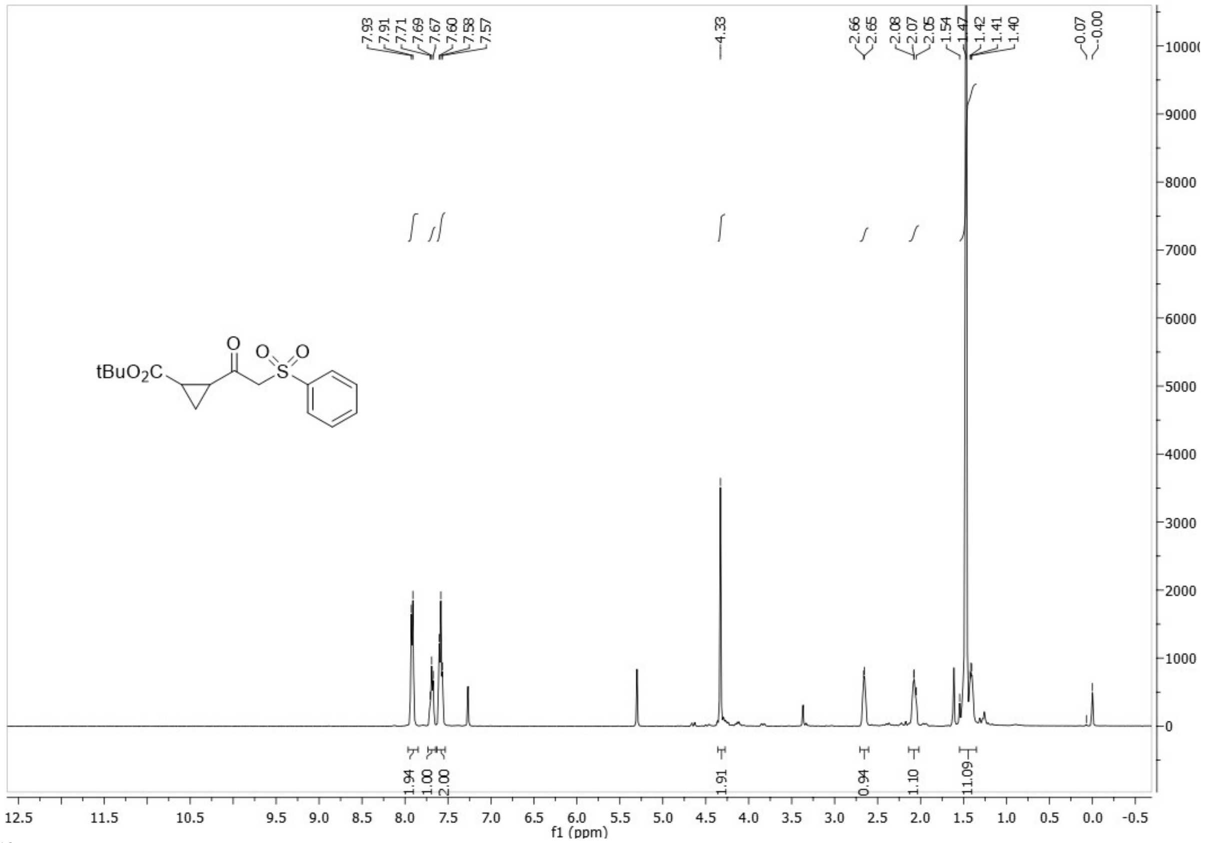

^{13}C NMR (101 MHz, $CDCl_3$)

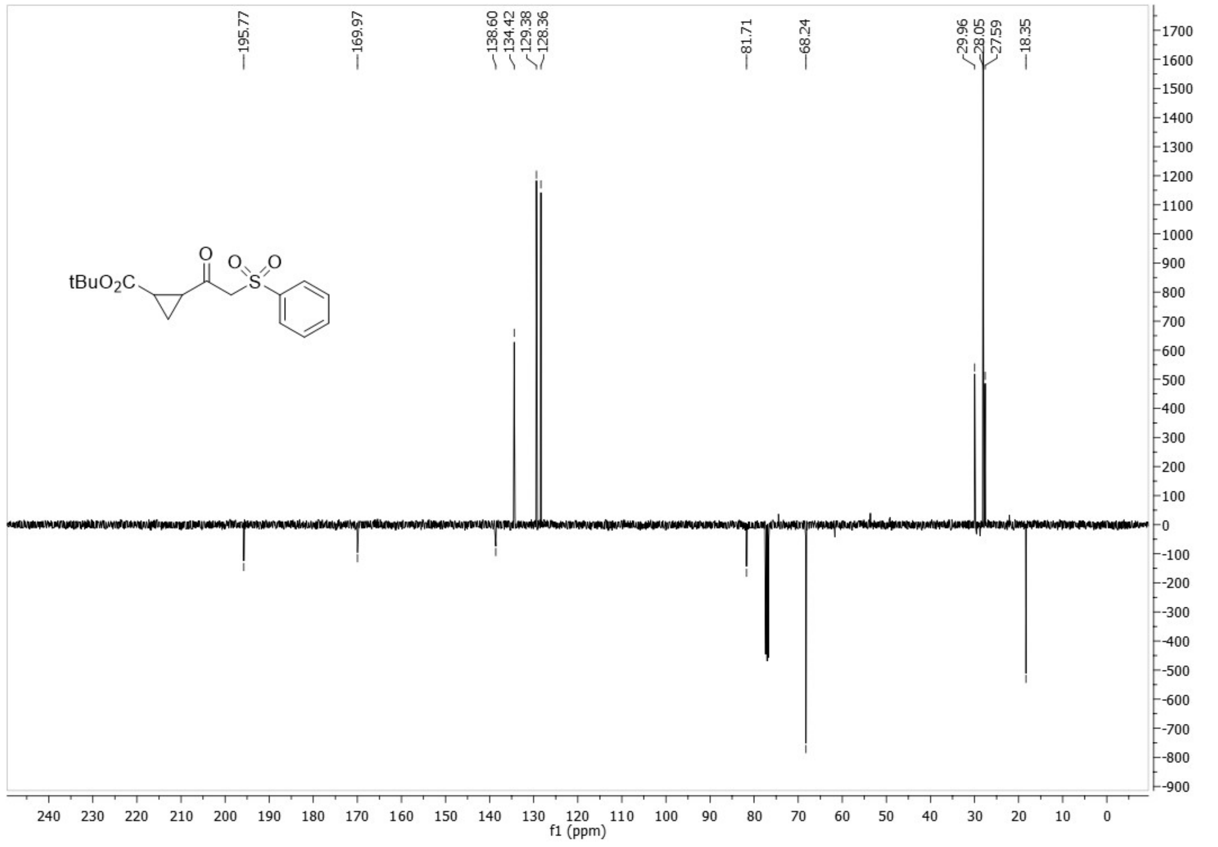

Ketone HPLC of *tert*-butyl 2-(2-(phenylsulfonyl)acetyl)cyclopropane-1-carboxylate.

Clarity - Chromatography SW
DataApex 2006
www.dataapex.com

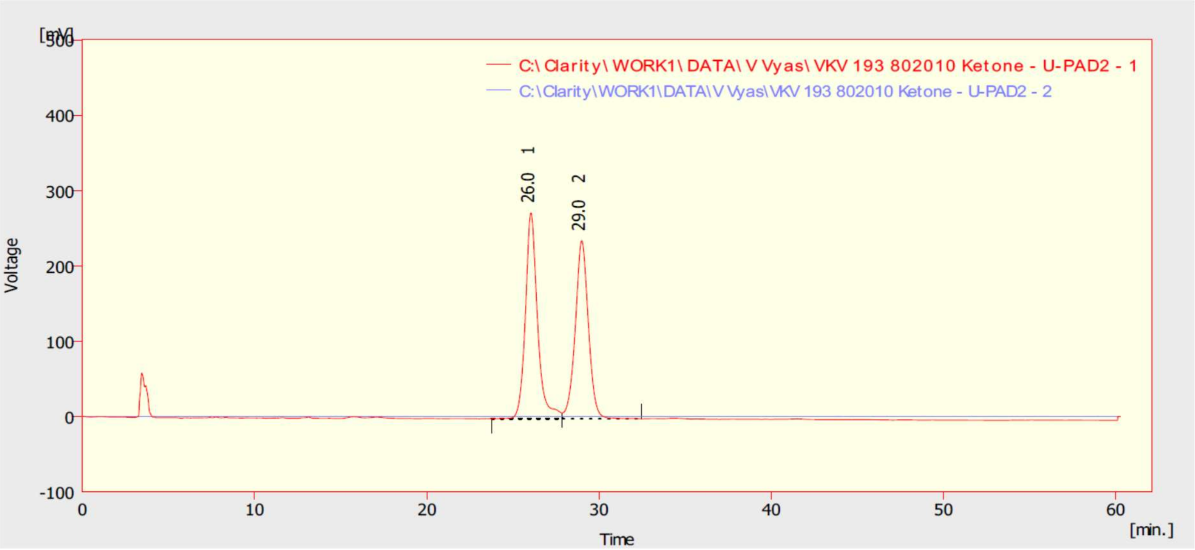

Result Table (Uncal - C:\Clarity\WORK1\DATA\V Vyas\VKV 193 802010 Ketone - U-PAD2 - 1)

	Reten. Time [min]	Area [mV.s]	Height [mV]	Area [%]	Height [%]	W05 [min]	Compound Name
1	26.044	13442.568	273.133	51.7	53.6	0.70	
2	28.992	12552.826	236.395	48.3	46.4	0.80	
	Total	25995.394	509.529	100.0	100.0		

***tert*-Butyl 2-(1-hydroxy-2-(phenylsulfonyl)ethyl)cyclopropane-1-carboxylate 12**

^{1}H NMR (400 MHz, $CDCl_3$)

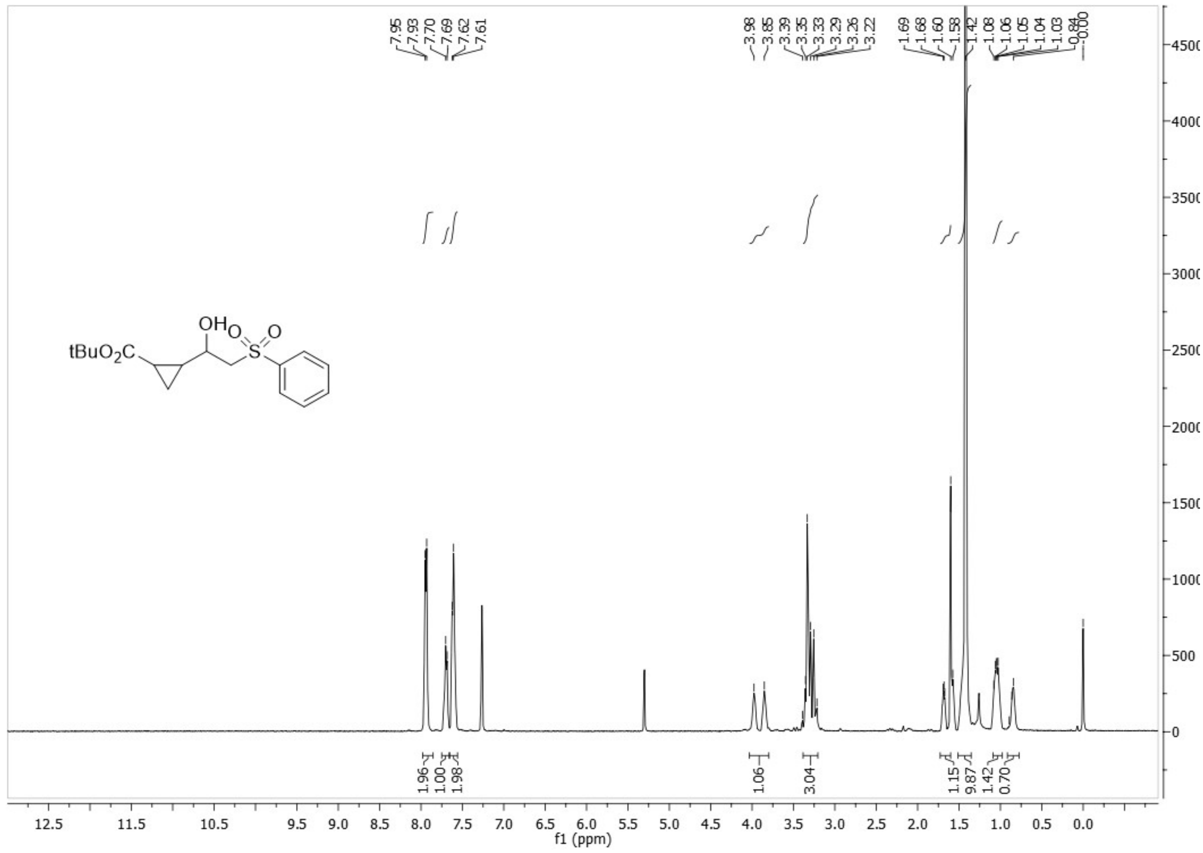

^{13}C NMR (101 MHz, $CDCl_3$)

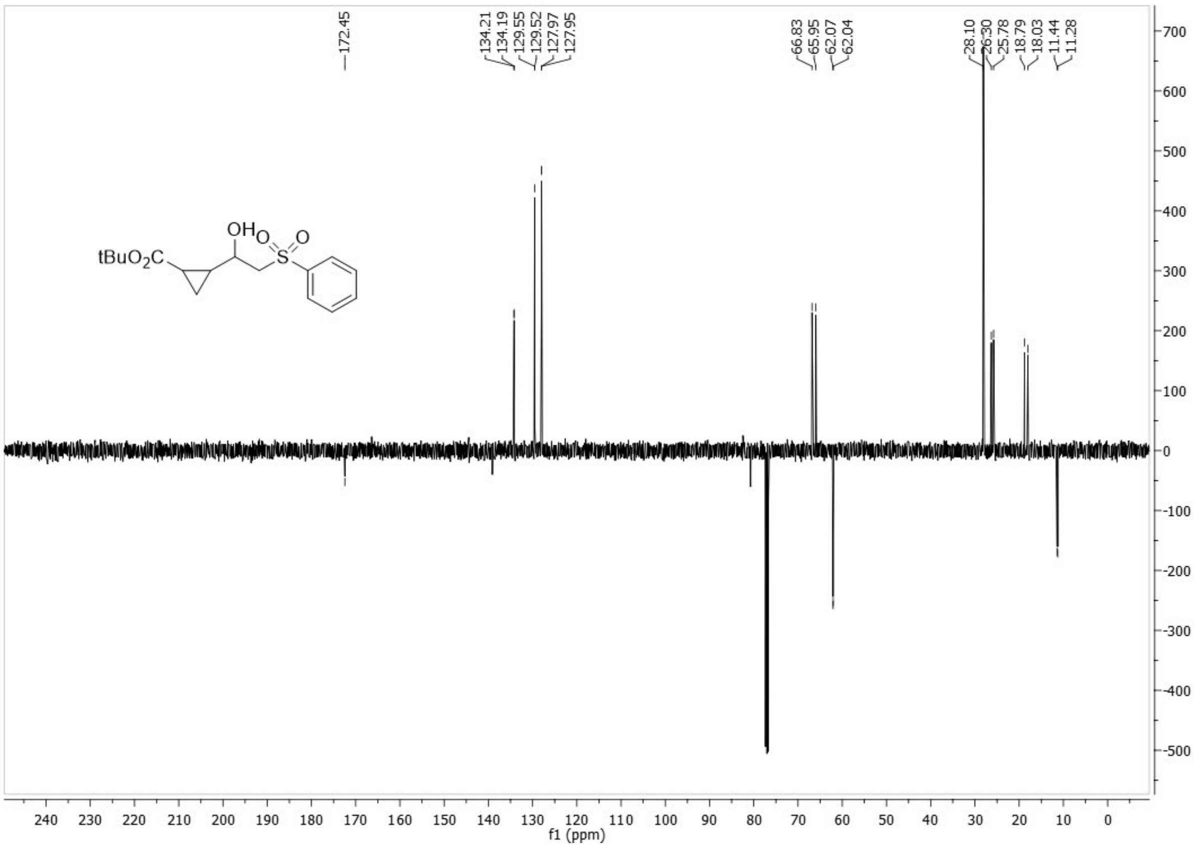

Racemic HPLC of *tert*-butyl 2-(1-hydroxy-2-(phenylsulfonyl)ethyl)cyclopropane-1-carboxylate

Clarity - Chromatography SW
DataApex 2006
www.dataapex.com

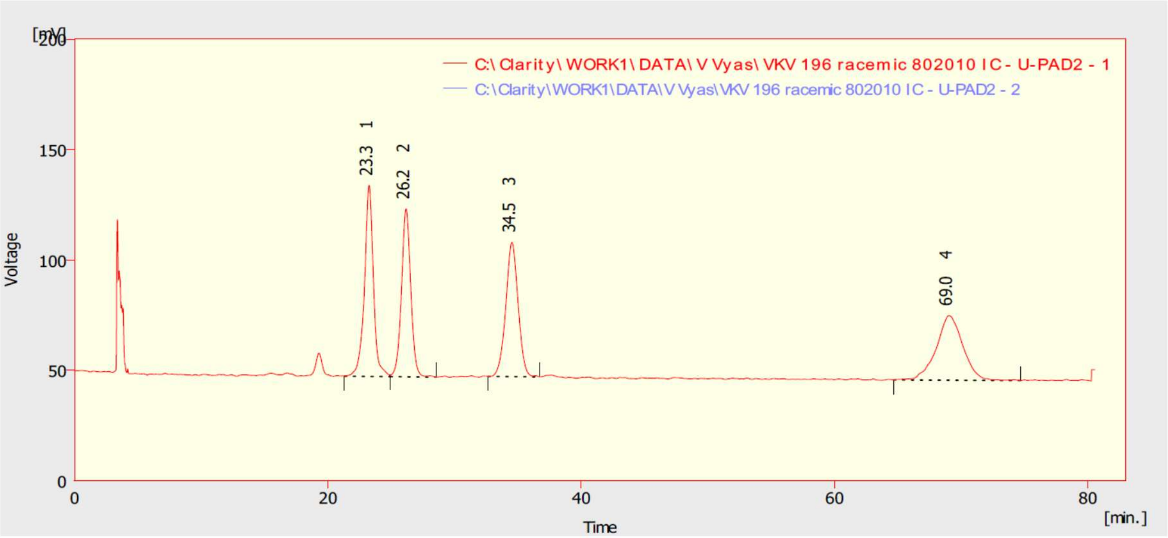

Result Table (Uncal - C:\Clarity\WORK1\DATA\V Vyas\VKV 196 racemic 802010 IC - U-PAD2 - 1)

	Reten. Time [min]	Area [mV.s]	Height [mV]	Area [%]	Height [%]	W05 [min]	Compound Name
1	23.252	4280.647	86.613	25.8	34.3	0.70	
2	26.172	3890.332	76.058	23.4	30.1	0.77	
3	34.528	4125.334	60.817	24.9	24.1	1.04	
4	69.016	4296.720	29.265	25.9	11.6	2.15	
	Total	16593.033	252.753	100.0	100.0		

HPLC after ATH *tert*-butyl 2-(1-hydroxy-2-(phenylsulfonyl)ethyl)cyclopropane-1-carboxylate . (100% conversion, 53:47 dr, 90.7% ee d1, 99.8% ee d2).

Clarity - Chromatography SW
DataApex 2006
www.dataapex.com

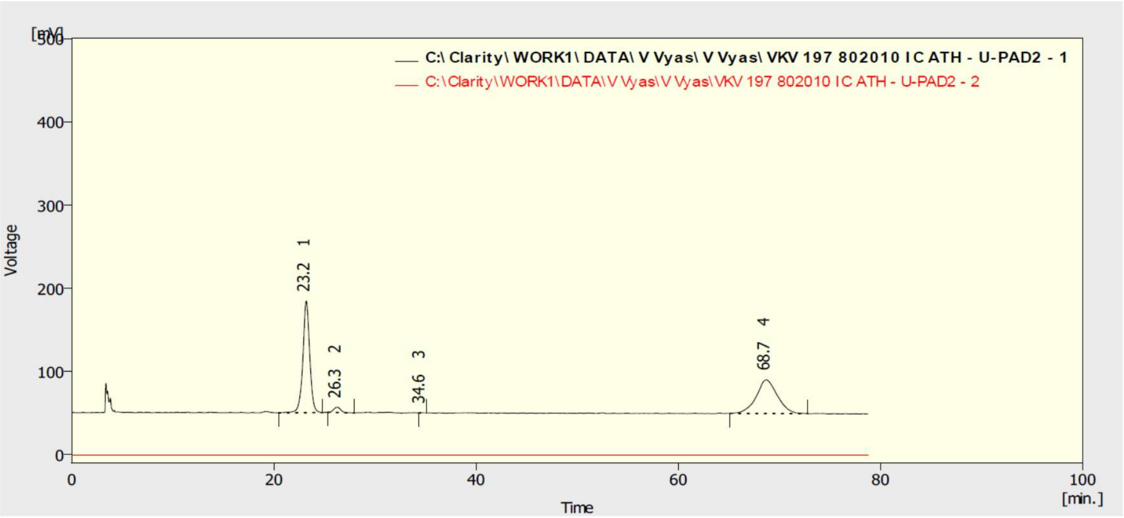

Result Table (Uncal - C:\Clarity\WORK1\DATA\V Vyas\V Vyas\VKV 197 802010 IC ATH - U-PAD2 - 1)

	Reten. Time [min]	Area [mV.s]	Height [mV]	Area [%]	Height [%]	W05 [min]	Compound Name
1	23.200	6325.245	134.285	50.5	74.0	0.68	
2	26.276	314.522	6.460	2.5	3.6	0.76	
3	34.584	7.158	0.324	0.1	0.2	0.34	
4	68.712	5879.594	40.465	46.9	22.3	2.17	
	Total	12526.519	181.535	100.0	100.0		

2-(2-Cyclopropyl-2-oxoethyl)isoindoline-1,3-dione

^{1}H NMR (400 MHz, $CDCl_3$)

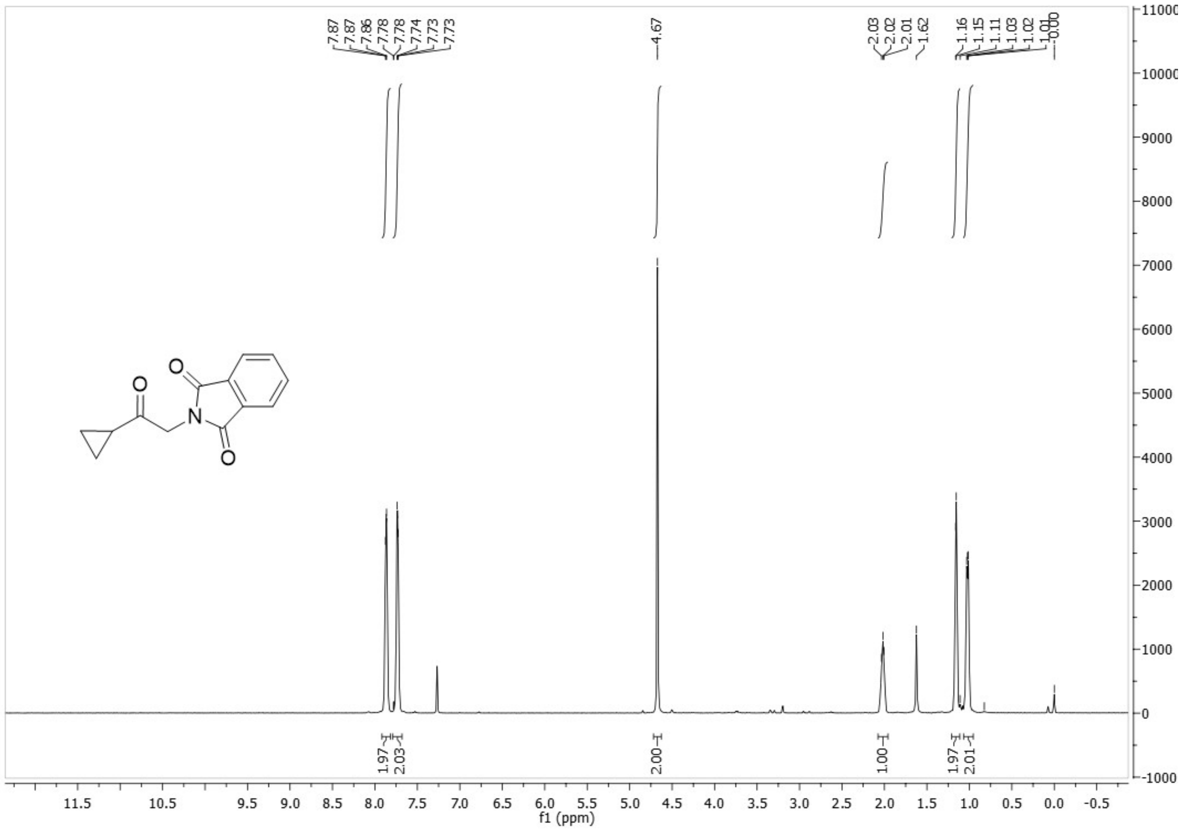

^{13}C NMR (101 MHz, $CDCl_3$

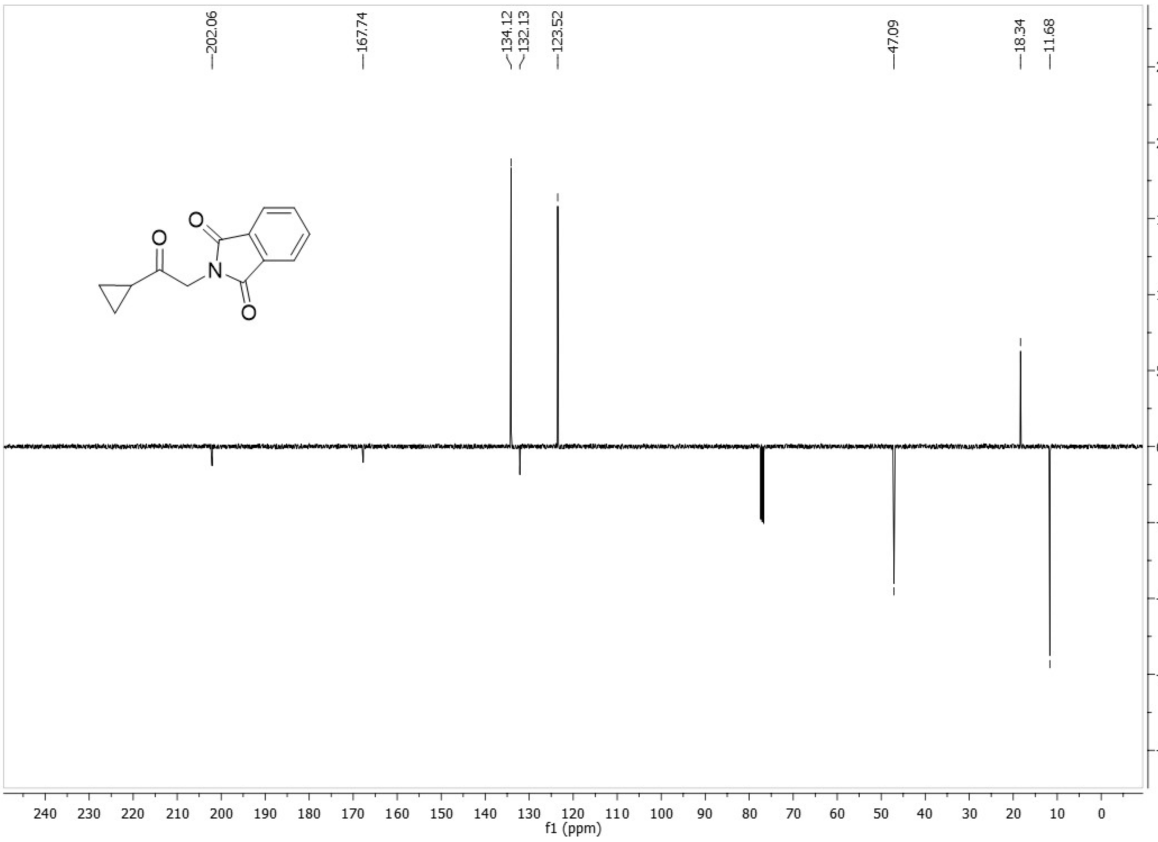

Ketone HPLC of 2-(2-Cyclopropyl-2-oxoethyl)isoindoline-1,3-dione.

Clarity - Chromatography SW
DataApex 2006
www.dataapex.com

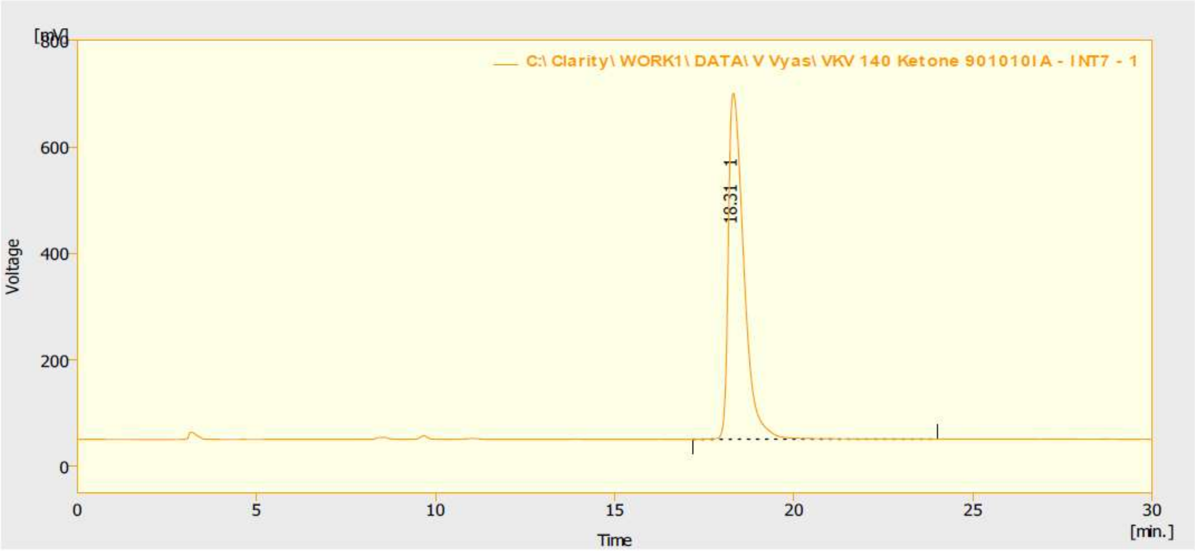

Result Table (Uncal - C:\Clarity\WORK1\DATA\V Vyas\VKV 140 Ketone 901010IA - INT7 - 1)

	Reten. Time [min]	Area [mV.s]	Height [mV]	Area [%]	Height [%]	W05 [min]	Compound Name
1	18.313	19578.627	650.157	100.0	100.0	0.45	
	Total	19578.627	650.157	100.0	100.0		

2-(2-Cyclopropyl-2-hydroxyethyl)isoindoline-1,3-dione 13

^{1}H NMR (400 MHz, $CDCl_3$)

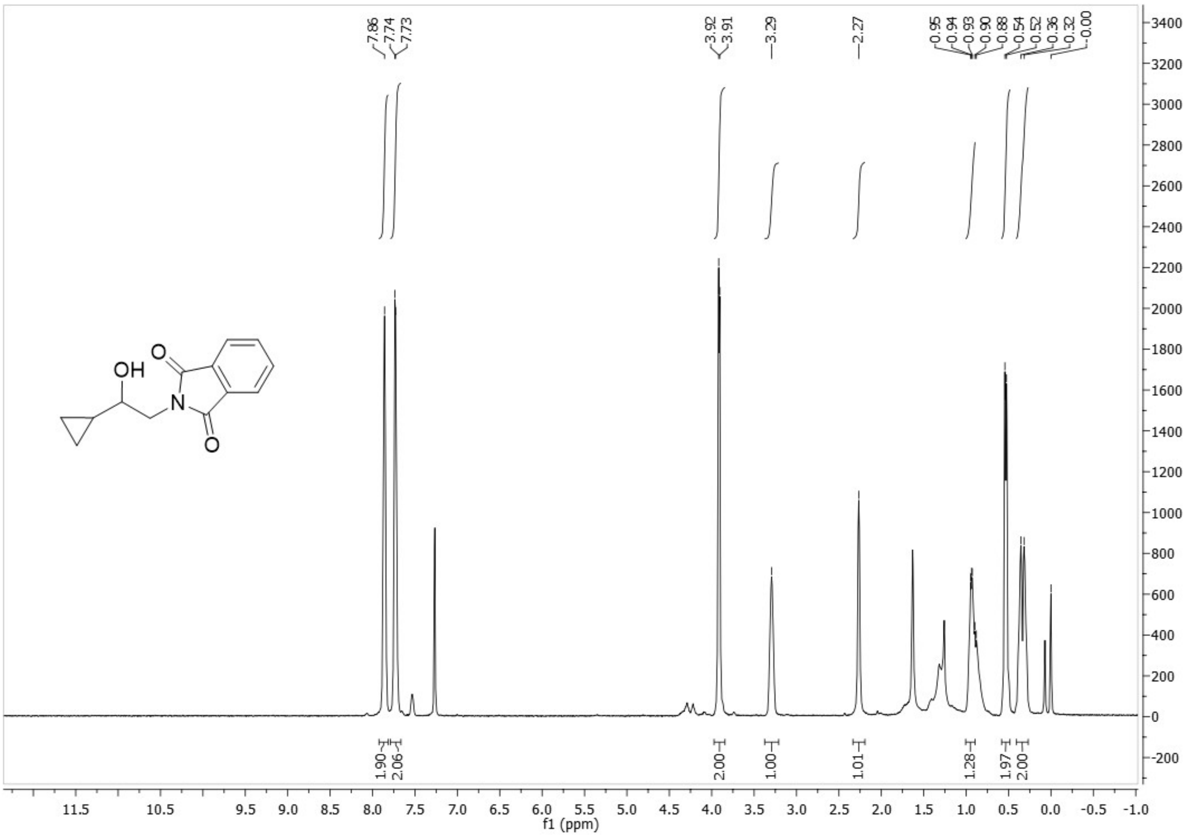

^{13}C NMR (101 MHz, $CDCl_3$

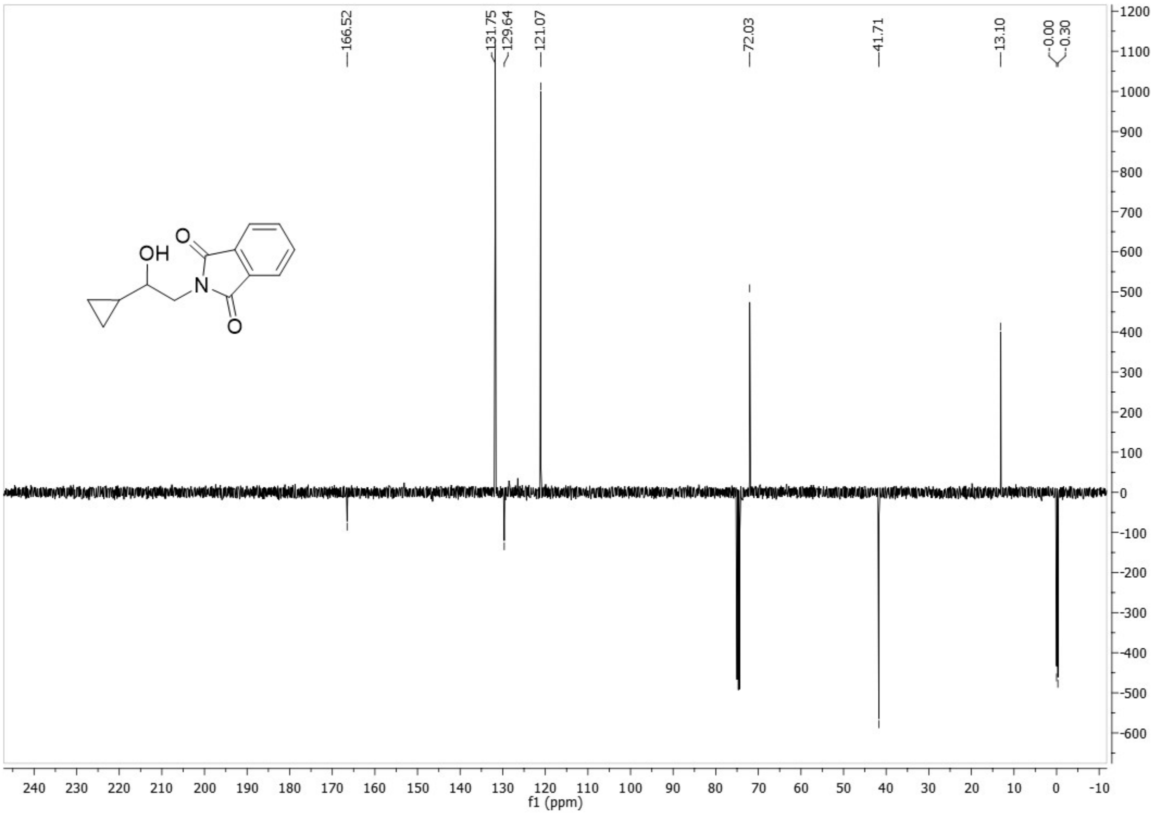

Racemic HPLC of 2-(2-cyclopropyl-2-hydroxyethyl)isoindoline-1,3-dione.

Clarity - Chromatography SW
DataApex 2006
www.dataapex.com

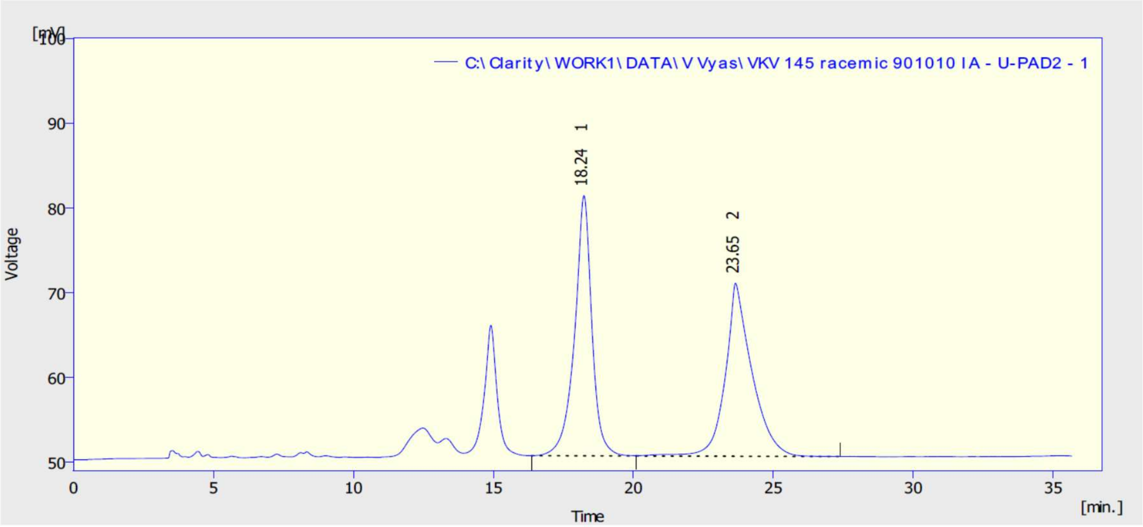

Result Table (Uncal - C:\Clarity\WORK1\DATA\V Vyas\VKV 145 racemic 901010 IA - U-PAD2 - 1)

	Reten. Time [min]	Area [mV.s]	Height [mV]	Area [%]	Height [%]	W05 [min]	Compound Name
1	18.236	1319.684	30.681	49.4	60.1	0.60	
2	23.648	1350.062	20.405	50.6	39.9	0.91	
	Total	2669.747	51.086	100.0	100.0		

HPLC after ATH 2-(2-cyclopropyl-2-hydroxyethyl)isoindoline-1,3-dione. (100% conversion, 96.4% ee).

Clarity - Chromatography SW
DataApex 2006
www.dataapex.com

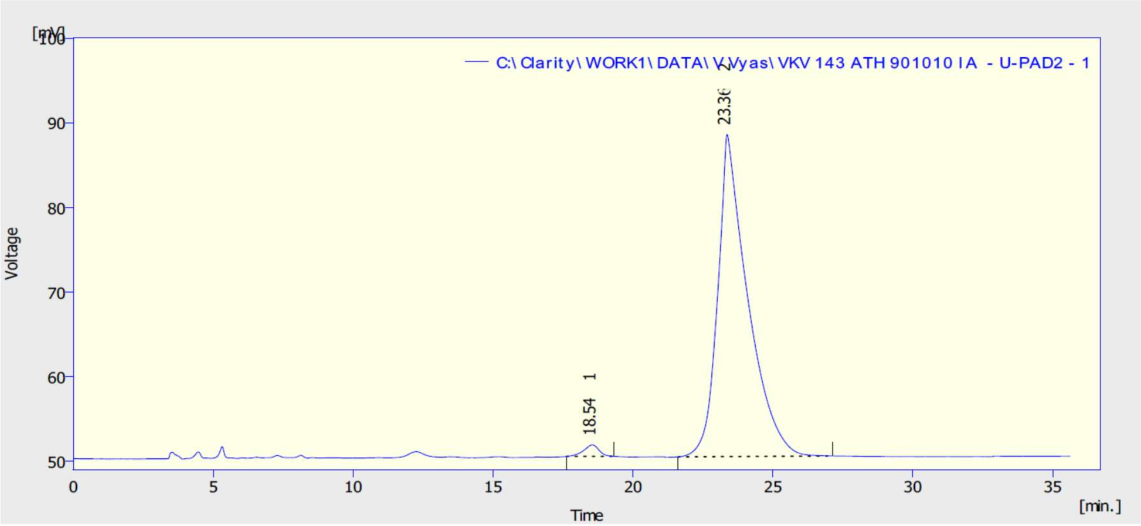

Result Table (Uncal - C:\Clarity\WORK1\DATA\V Vyas\VKV 143 ATH 901010 IA - U-PAD2 - 1)

	Reten. Time [min]	Area [mV.s]	Height [mV]	Area [%]	Height [%]	W05 [min]	Compound Name
1	18.544	51.917	1.372	1.8	3.5	0.57	
2	23.364	2839.225	38.053	98.2	96.5	1.07	
	Total	2891.143	39.424	100.0	100.0		

1-Phenoxy-3-(phenylsulfonyl)propan-2-ol 14

^{1}H NMR (400 MHz, $CDCl_3$)

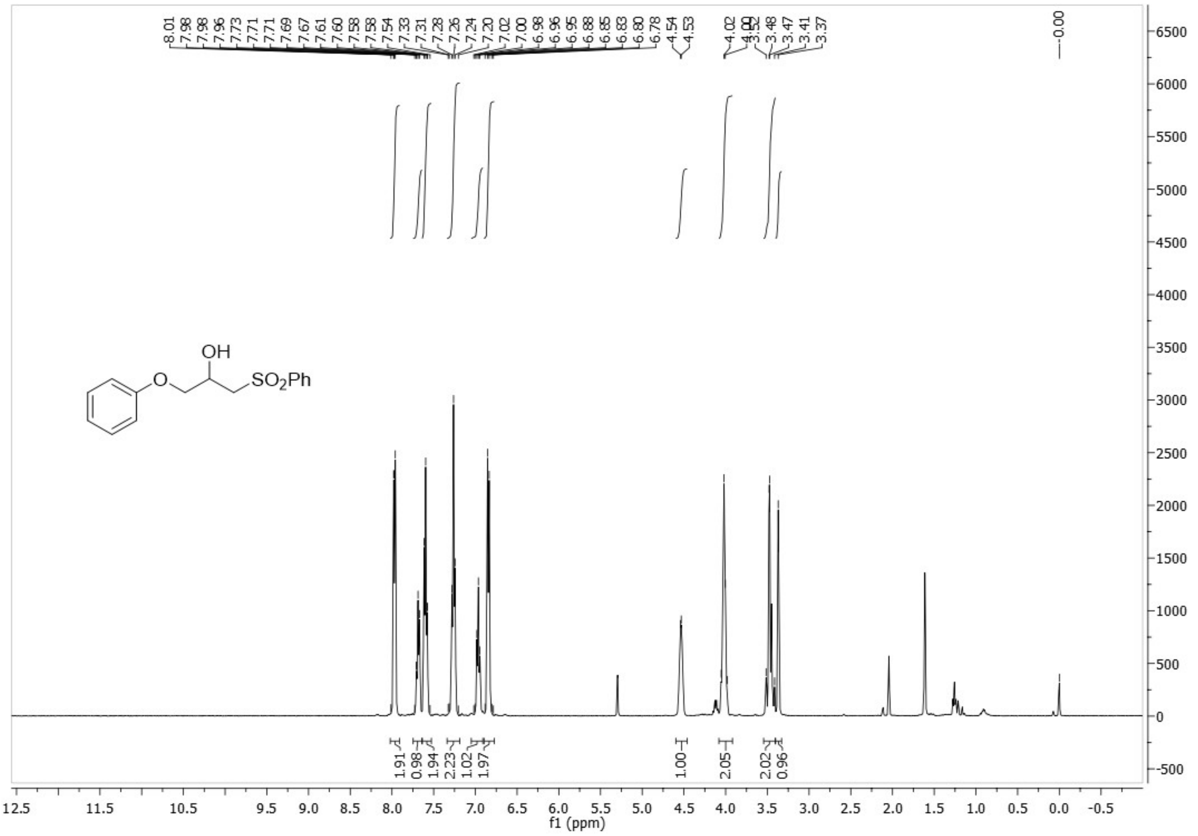

^{13}C NMR (101 MHz, $CDCl_3$

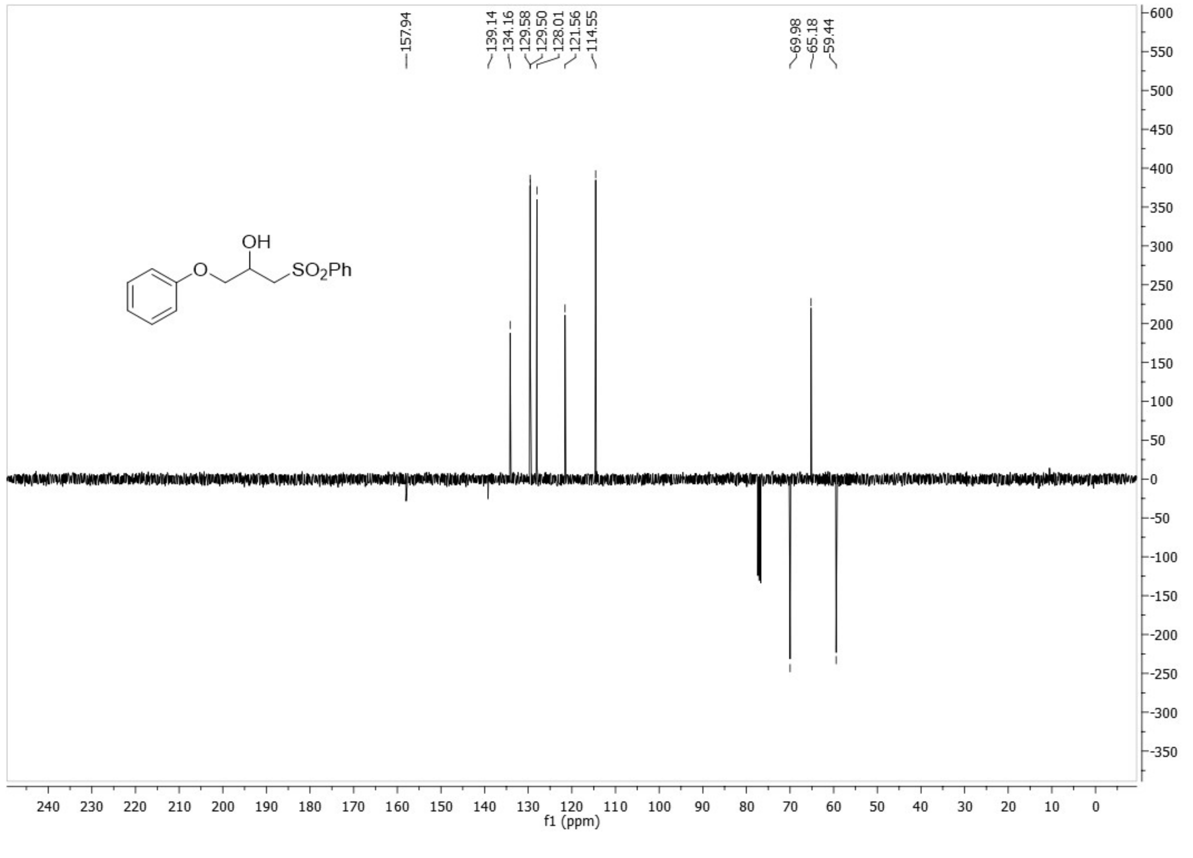

Racemic HPLC of 1-Phenoxy-3-(phenylsulfonyl)propan-2-ol.

Clarity - Chromatography SW
DataApex 2006
www.dataapex.com

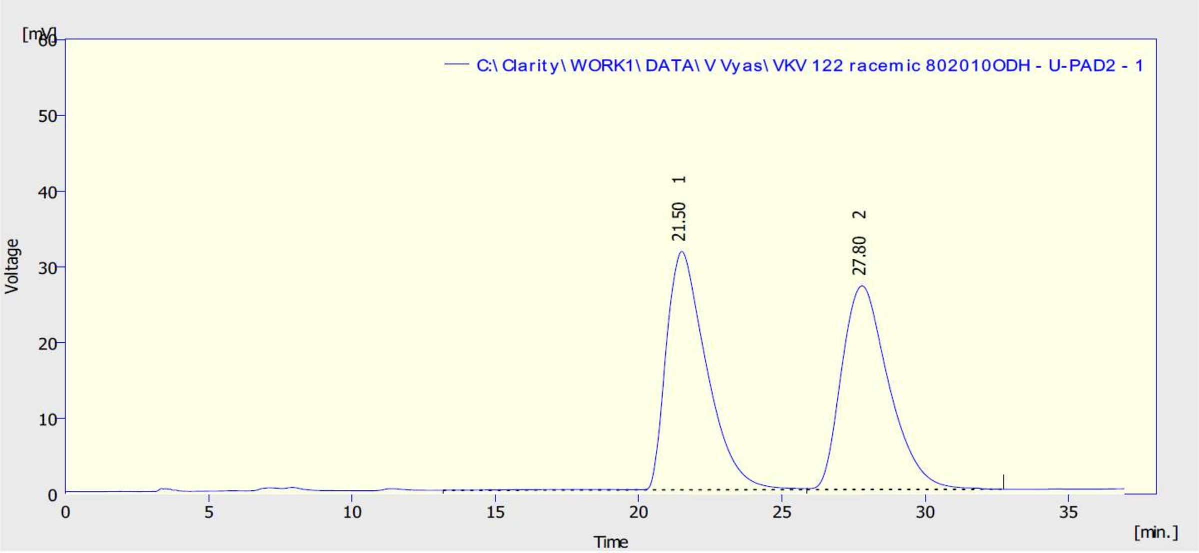

Result Table (Uncal - C:\Clarity\WORK1\DATA\V Vyas\VKV 122 racemic 802010ODH - U-PAD2 - 1)

	Reten. Time [min]	Area [mV.s]	Height [mV]	Area [%]	Height [%]	W05 [min]	Compound Name
1	21.504	3090.773	31.456	50.1	53.9	1.51	
2	27.796	3078.556	26.877	49.9	46.1	1.77	
	Total	6169.329	58.333	100.0	100.0		

HPLC after ATH 1-Phenoxy-3-(phenylsulfonyl)propan-2-ol. (100% conversion, 96.4% ee).

Clarity - Chromatography SW
DataApex 2006
www.dataapex.com

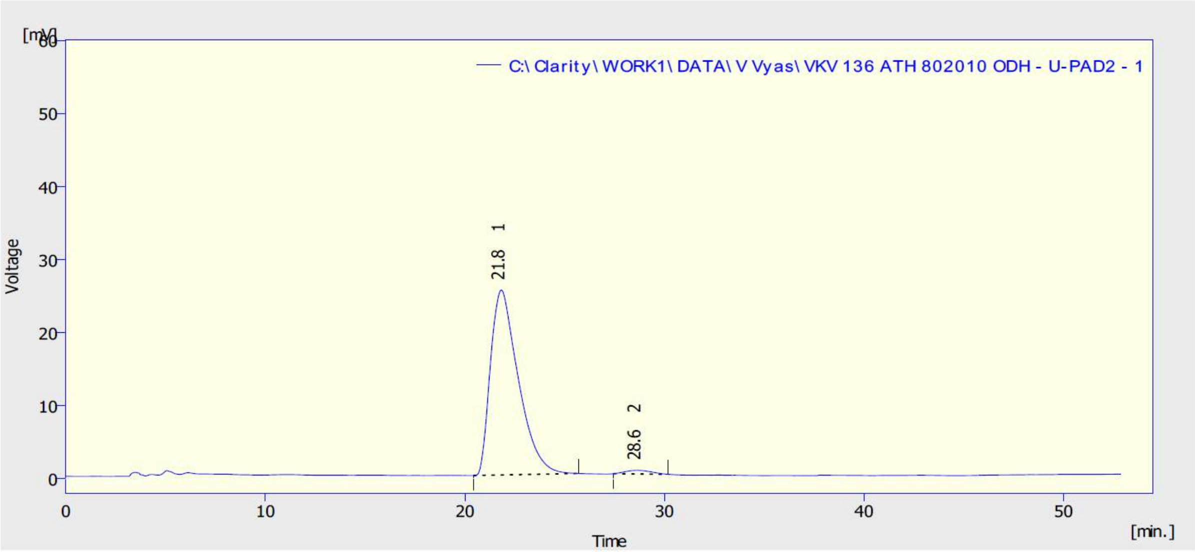

Result Table (Uncal - C:\Clarity\WORK1\DATA\V Vyas\VKV 136 ATH 802010 ODH - U-PAD2 - 1)

	Reten. Time [min]	Area [mV.s]	Height [mV]	Area [%]	Height [%]	W05 [min]	Compound Name
1	21.824	2457.334	25.306	98.2	98.1	1.51	
2	28.628	44.704	0.494	1.8	1.9	1.50	
	Total	2502.037	25.800	100.0	100.0		

1-Phenoxy-3-(phenylsulfonyl)propan-2-one

^{1}H NMR (400 MHz, $CDCl_3$)

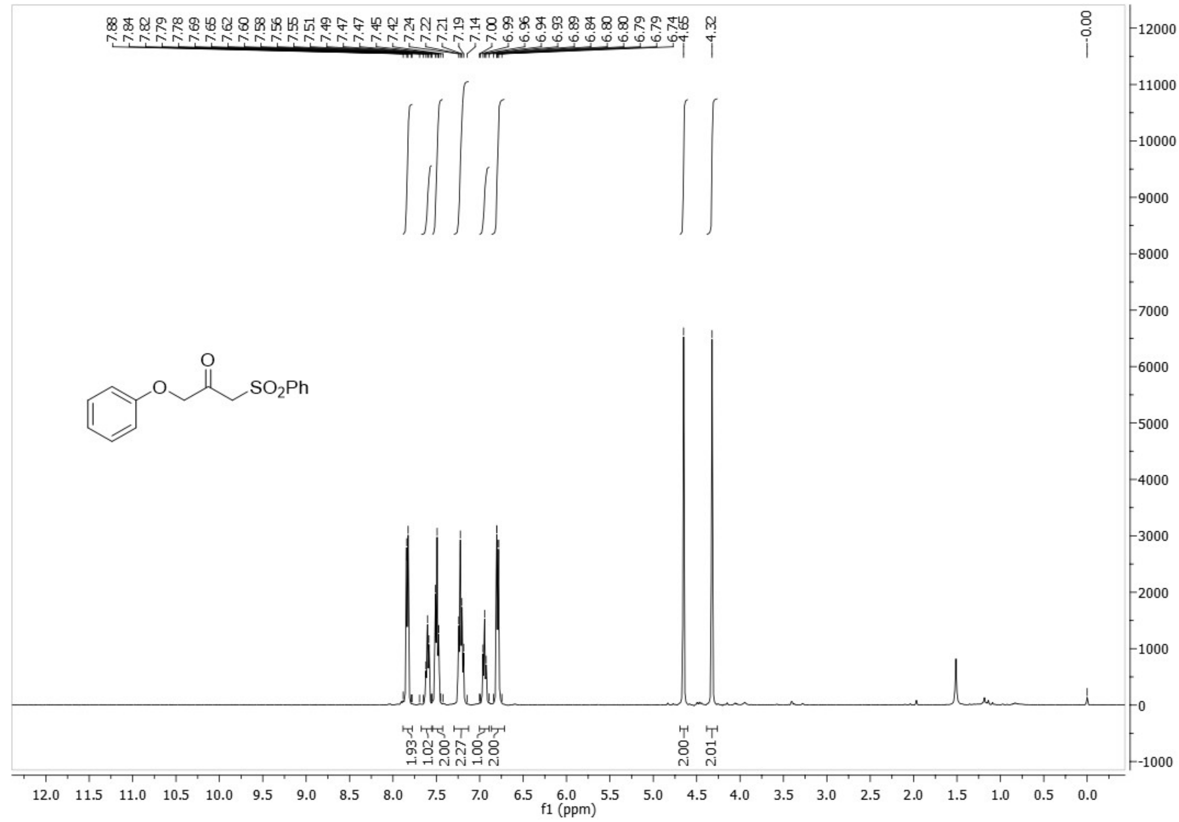

^{13}C NMR (101 MHz, $CDCl_3$

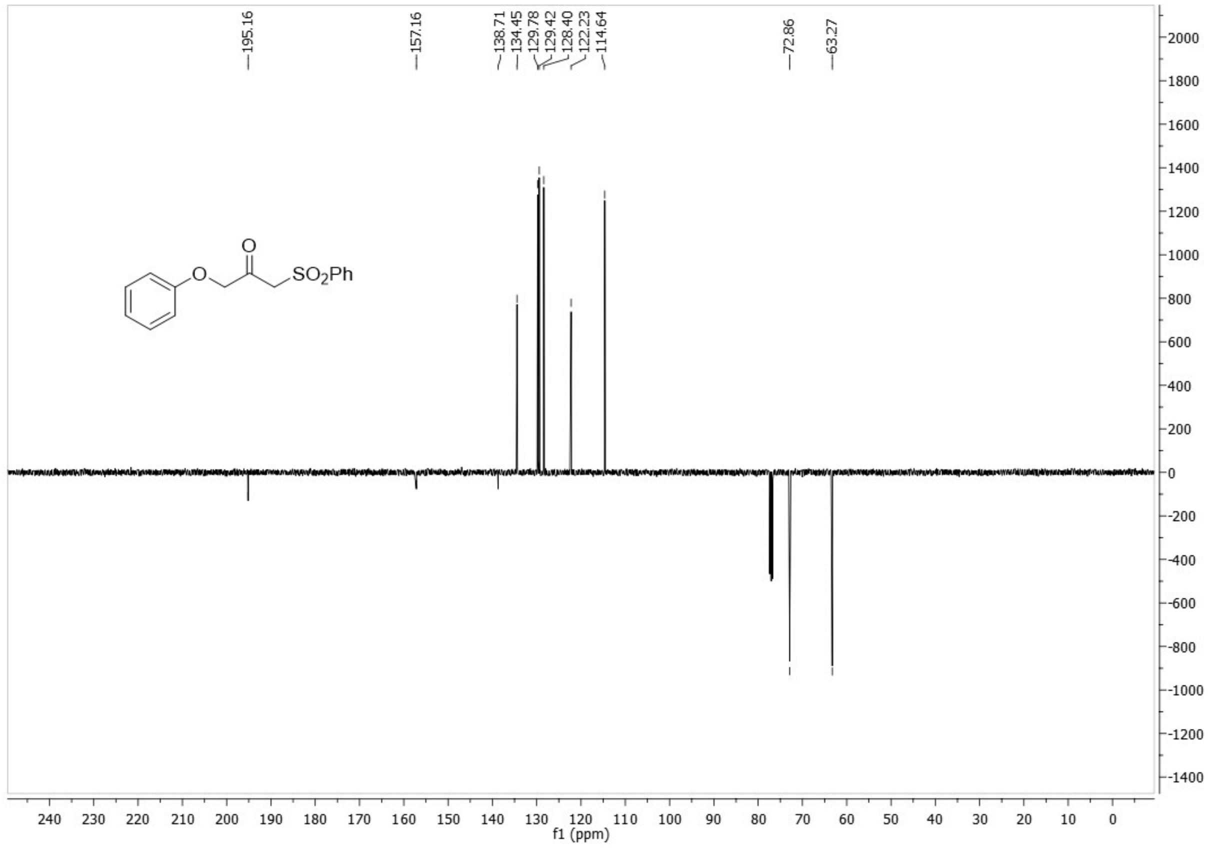

Ketone HPLC of 1-Phenoxy-3-(phenylsulfonyl)propan-2-one.

Clarity - Chromatography SW
DataApex 2006
www.dataapex.com

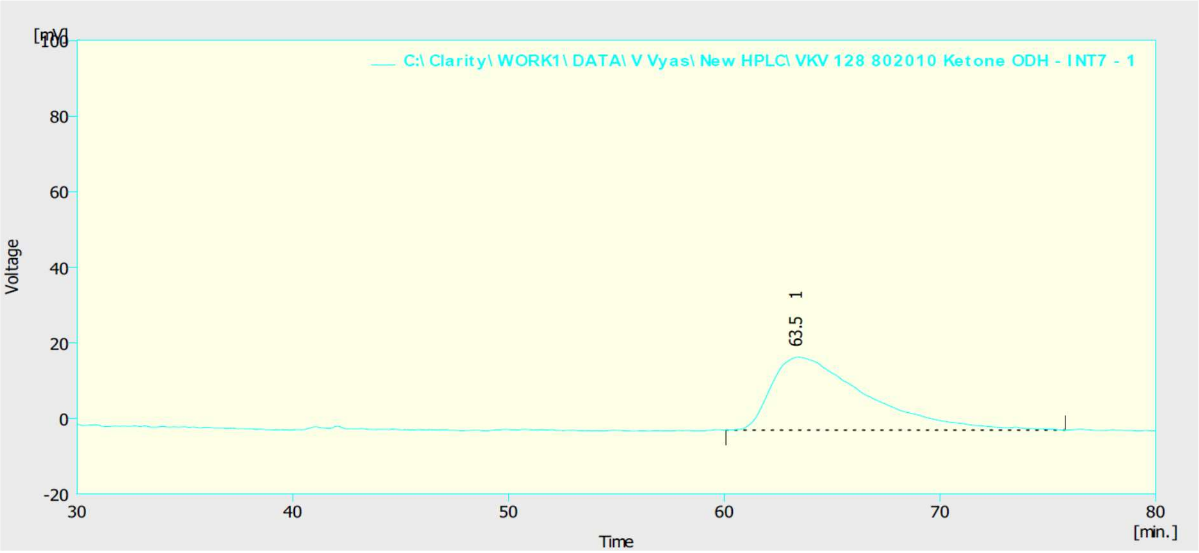

Result Table (Uncal - C:\Clarity\WORK1\DATA\V Vyas\New HPLC\VKV 128 802010 Ketone ODH - INT7 - 1)

	Reten. Time [min]	Area [mV.s]	Height [mV]	Area [%]	Height [%]	W05 [min]	Compound Name
1	63.463	5902.142	19.354	100.0	100.0	4.44	
	Total	5902.142	19.354	100.0	100.0		

1-Phenoxy-3-(phenylthio)propan-2-ol

^{1}H NMR (400 MHz, $CDCl_3$)

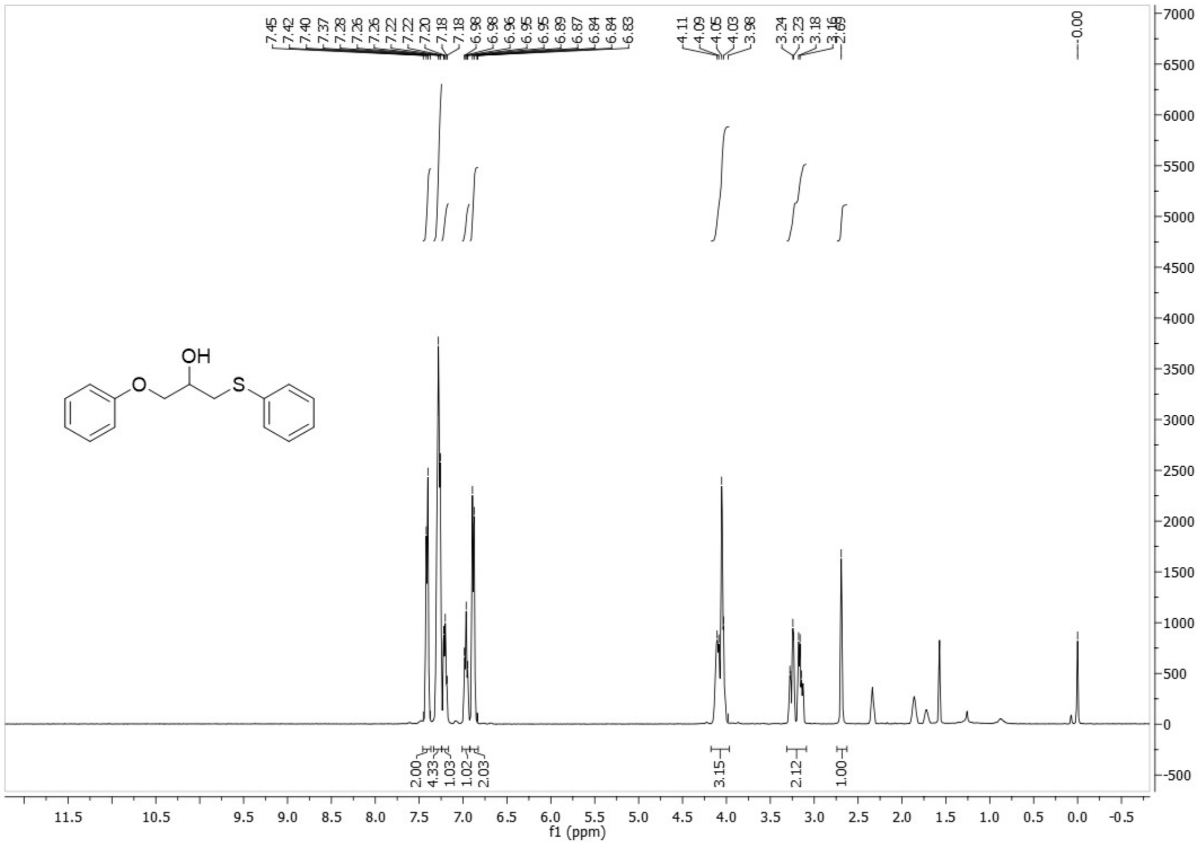

^{13}C NMR (101 MHz, $CDCl_3$

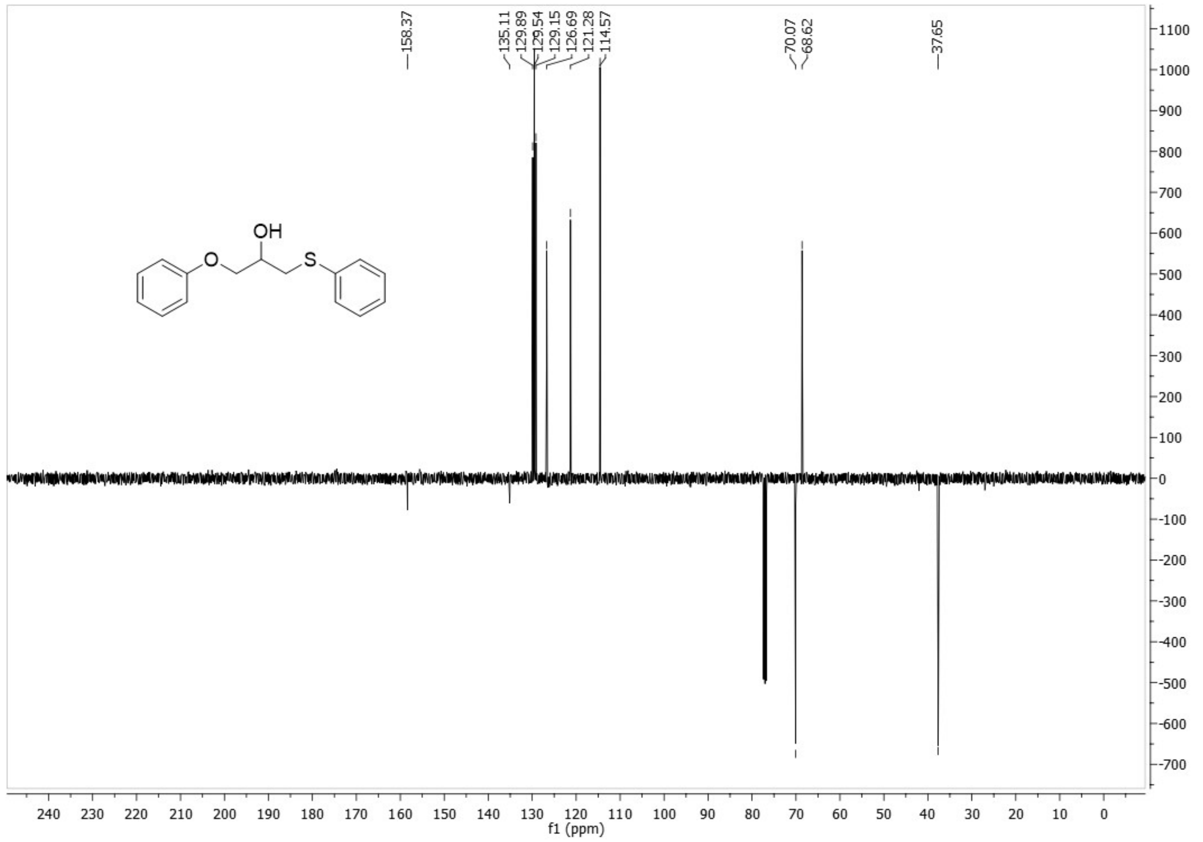

Racemic HPLC of 1-Phenoxy-3-(phenylthio)propan-2-ol.

Clarity - Chromatography SW
DataApex 2006
www.dataapex.com

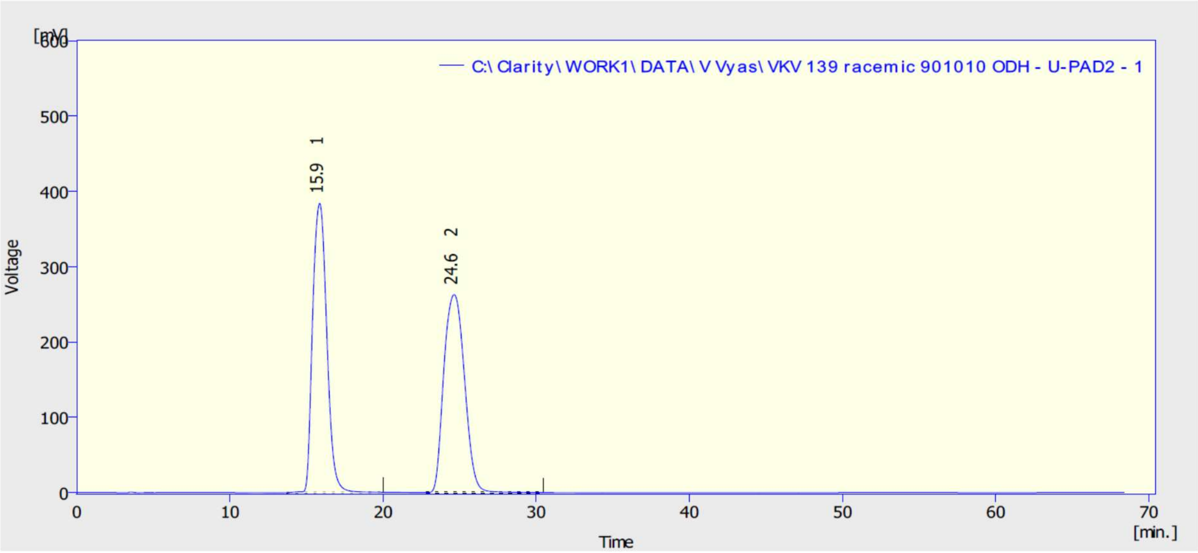

Result Table (Uncal - C:\Clarity\WORK1\DATA\V Vyas\VKV 139 racemic 901010 ODH - U-PAD2 - 1)

	Reten. Time [min]	Area [mV.s]	Height [mV]	Area [%]	Height [%]	W05 [min]	Compound Name
1	15.856	24669.315	383.925	49.9	59.4	1.02	
2	24.636	24722.244	262.938	50.1	40.6	1.53	
	Total	49391.559	646.863	100.0	100.0		

HPLC after ATH 1-Phenoxy-3-(phenylthio)propan-2-ol. (100% conversion, 80.8% ee).

Clarity - Chromatography SW
DataApex 2006
www.dataapex.com

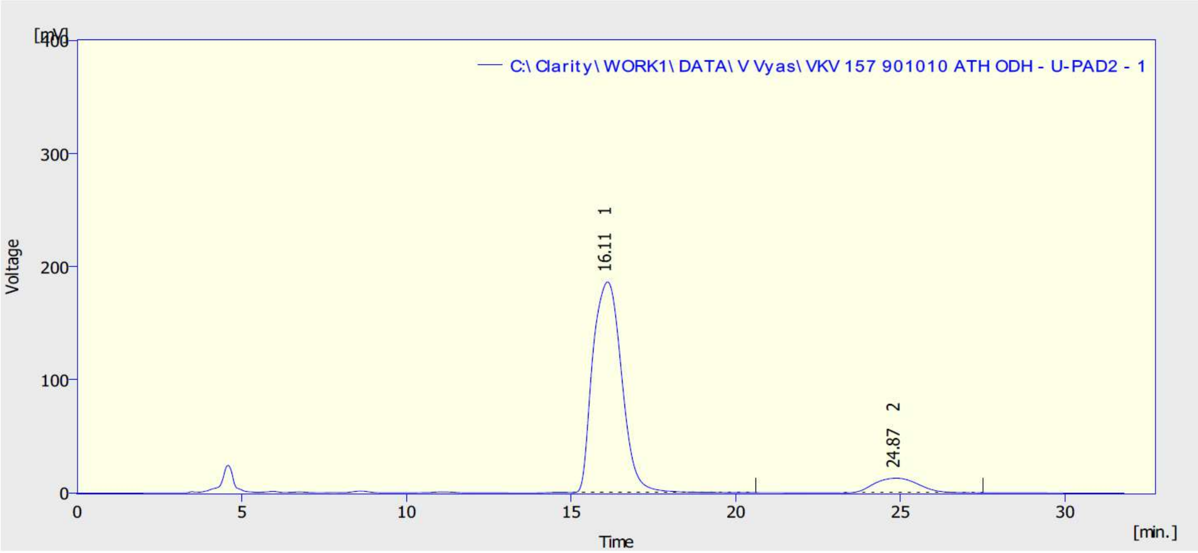

Result Table (Uncal - C:\Clarity\WORK1\DATA\V Vyas\VKV 157 901010 ATH ODH - U-PAD2 - 1)

	Reten. Time [min]	Area [mV.s]	Height [mV]	Area [%]	Height [%]	W05 [min]	Compound Name
1	16.108	11397.873	186.118	90.4	93.5	1.00	
2	24.868	1211.981	12.949	9.6	6.5	1.53	
	Total	12609.854	199.067	100.0	100.0		

1-Phenoxy-3-(phenylthio)propan-2-one

^{1}H NMR (400 MHz, $CDCl_3$)

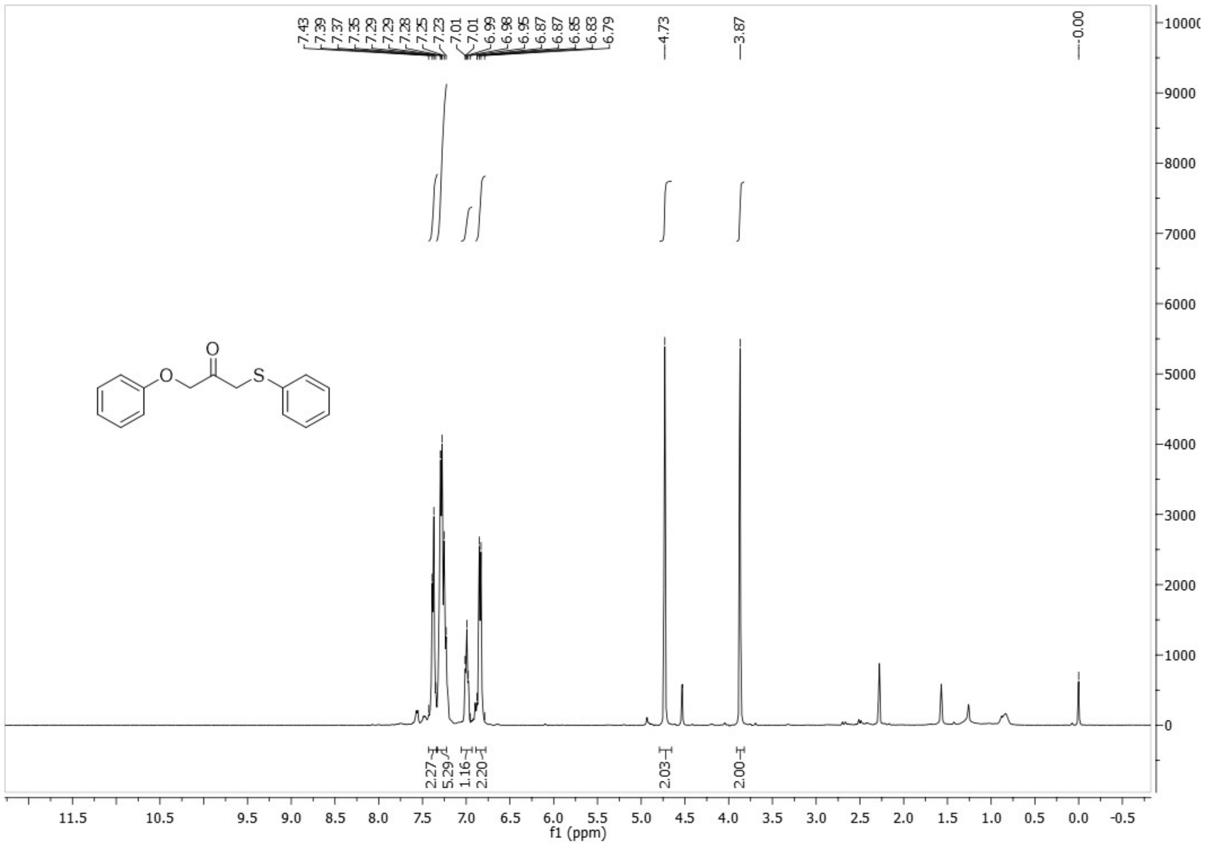

^{13}C NMR (101 MHz, $CDCl_3$

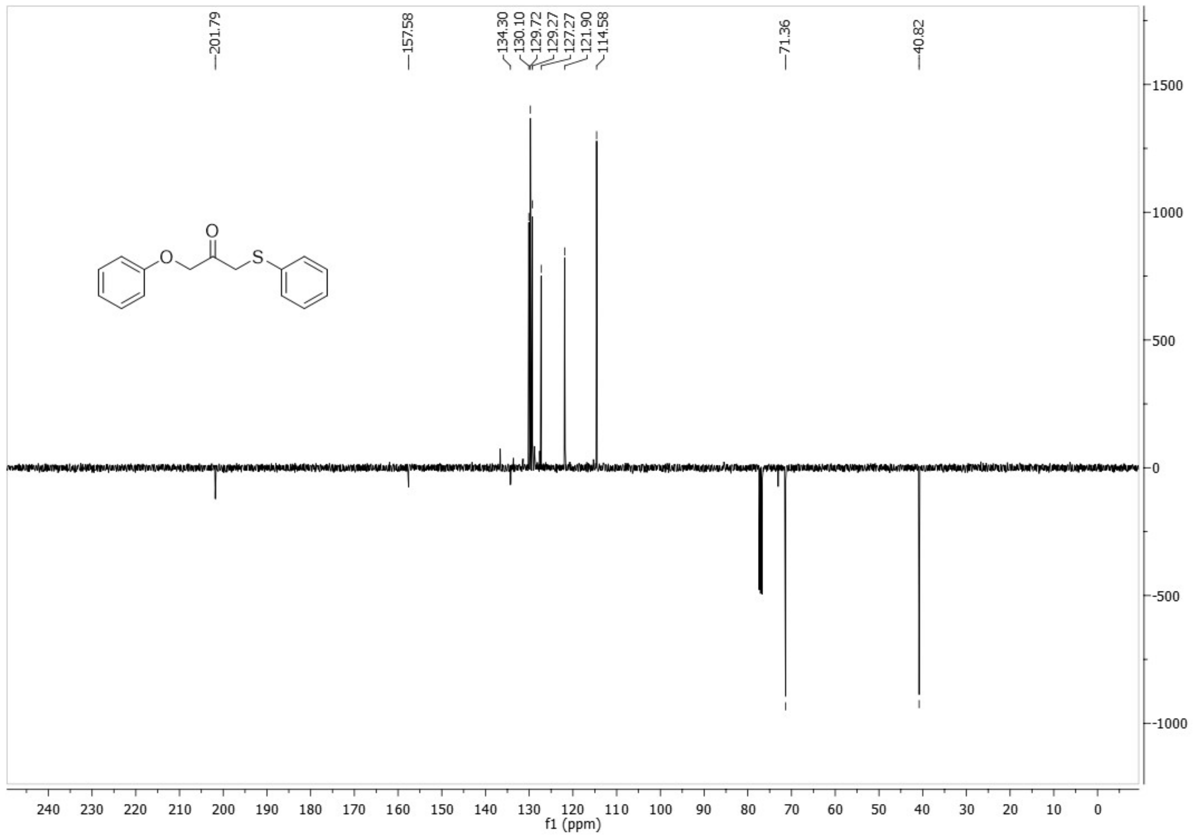

Ketone HPLC of 1-Phenoxy-3-(phenylthio)propan-2-one.

Clarity - Chromatography SW

DataApex 2006

www.dataapex.com

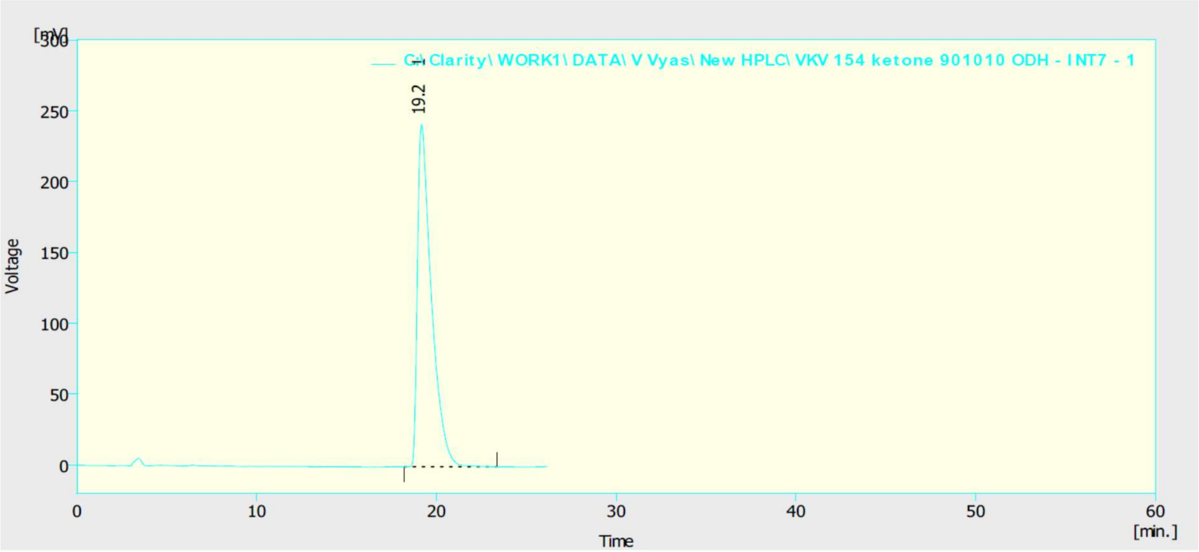

Result Table (Uncal - C:\Clarity\WORK1\DATA\V Vyas\New HPLC\VKV 154 ketone 901010 ODH - INT7 - 1)

	Reten. Time [min]	Area [mV.s]	Height [mV]	Area [%]	Height [%]	W05 [min]	Compound Name
1	19.170	13023.036	241.298	100.0	100.0	0.80	
	Total	13023.036	241.298	100.0	100.0		

1-Isopropoxy-3-(phenylsulfonyl)propan-2-ol 15

^{1}H NMR (400 MHz, $CDCl_3$)

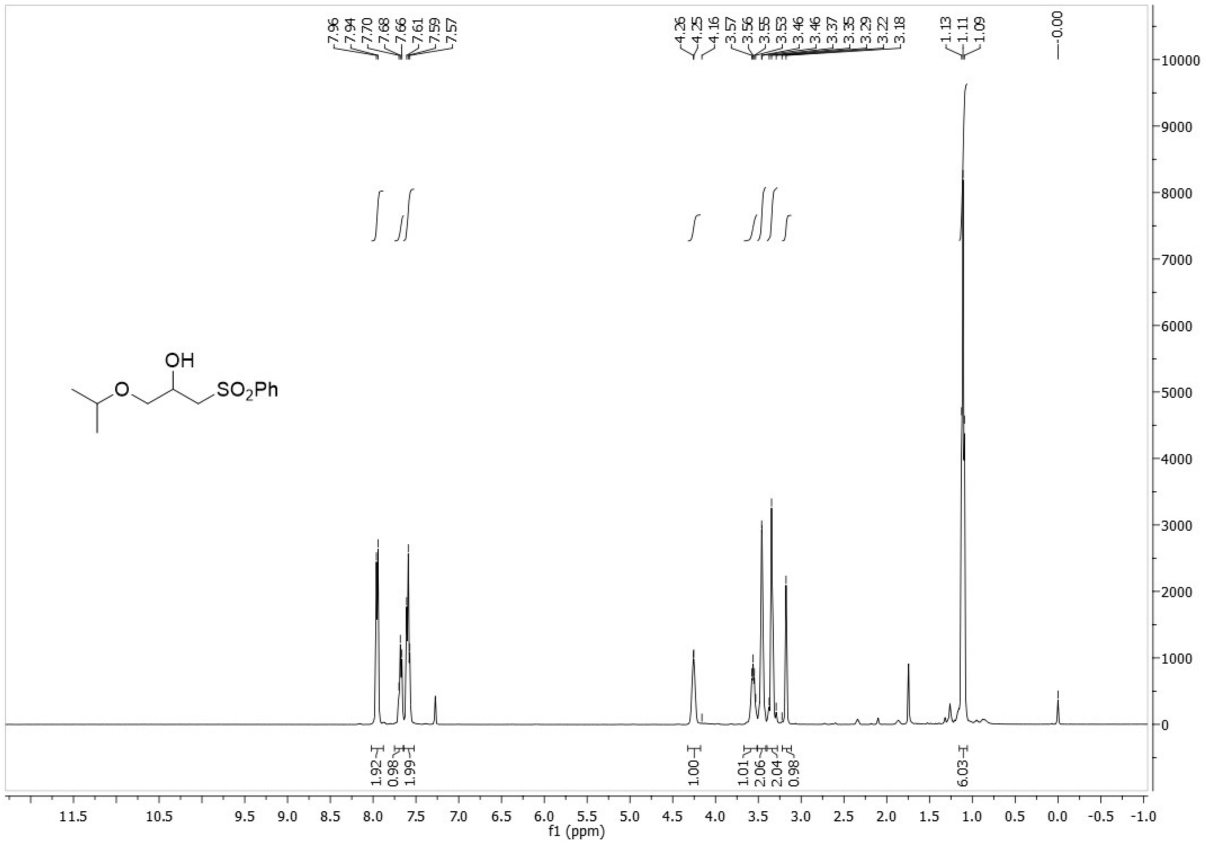

^{13}C NMR (101 MHz, $CDCl_3$

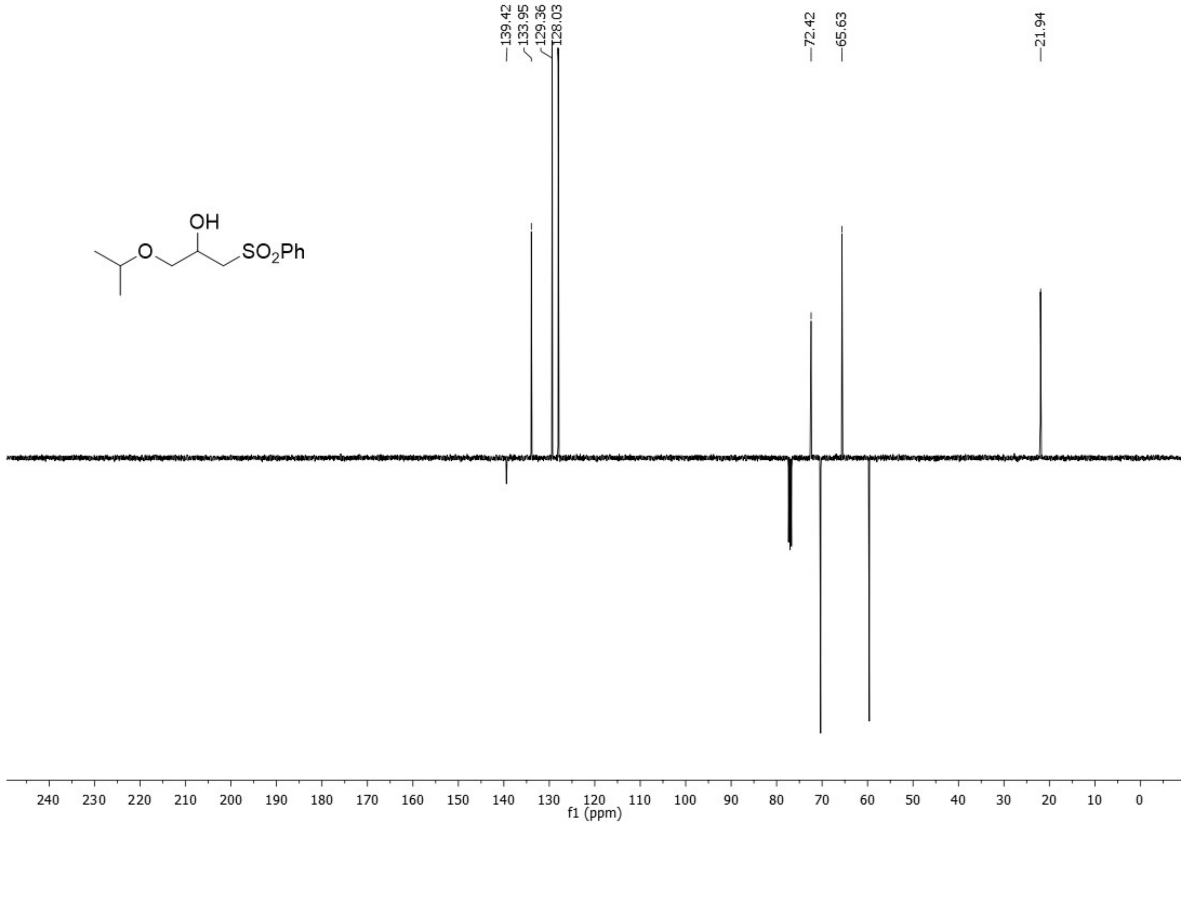

Racemic HPLC of 1-Isopropoxy-3-(phenylsulfonyl)propan-2-ol.

Clarity - Chromatography SW
DataApex 2006
www.dataapex.com

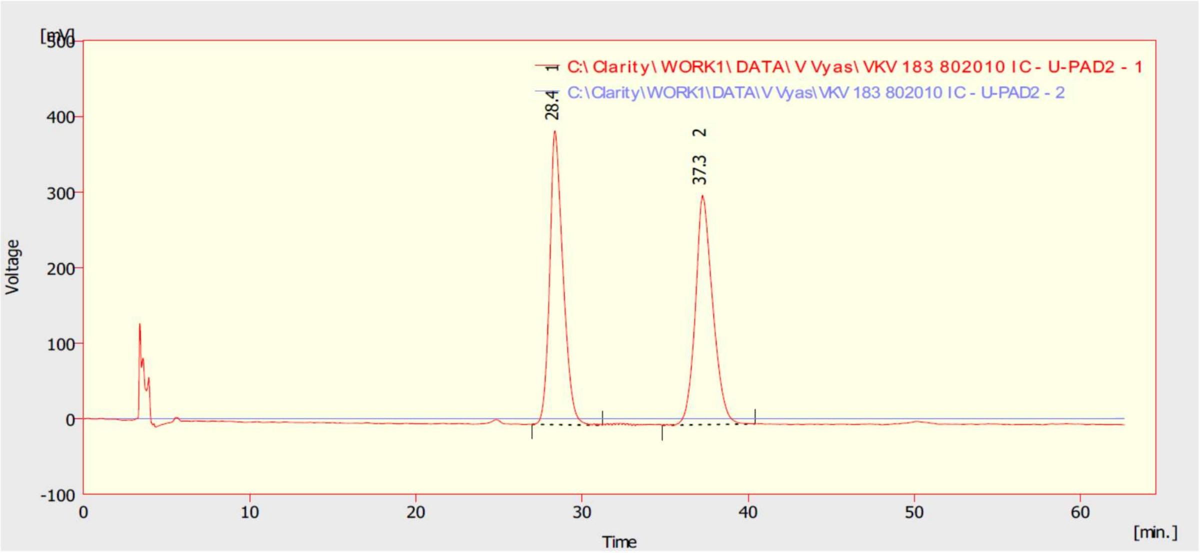

Result Table (Uncal - C:\Clarity\WORK1\DATA\V Vyas\VKV 183 802010 IC - U-PAD2 - 1)

	Reten. Time [min]	Area [mV.s]	Height [mV]	Area [%]	Height [%]	W05 [min]	Compound Name
1	28.372	21770.003	388.906	49.6	56.2	0.84	
2	37.256	22118.697	303.655	50.4	43.8	1.08	
	Total	43888.700	692.561	100.0	100.0		

HPLC after ATH 1-Isopropoxy-3-(phenylsulfonyl)propan-2-ol. (100% conversion, 98.6% ee).

Clarity - Chromatography SW
DataApex 2006
www.dataapex.com

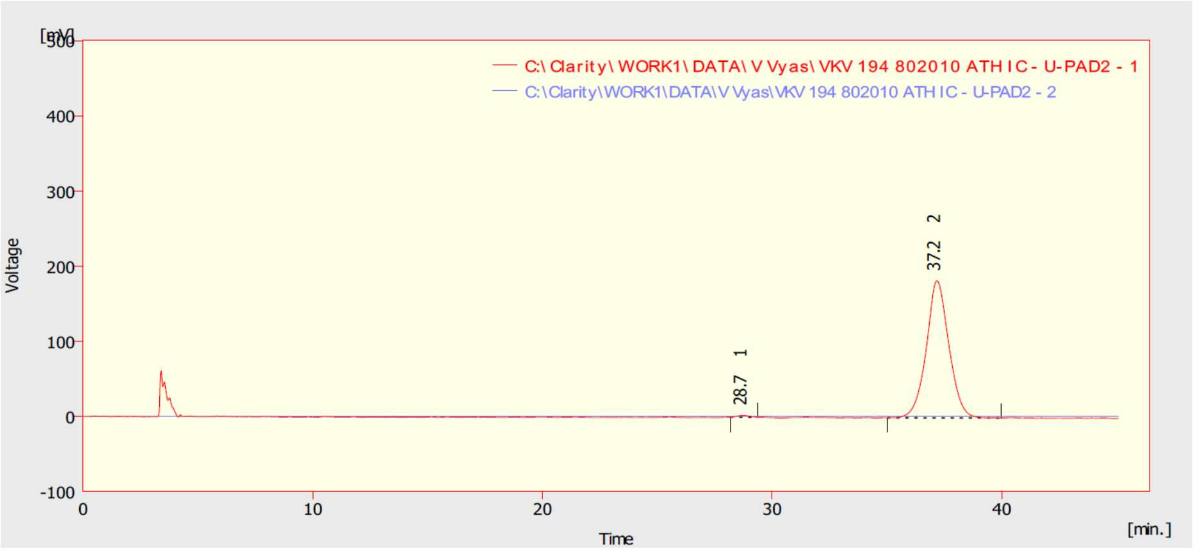

Result Table (Uncal - C:\Clarity\WORK1\DATA\V Vyas\VKV 194 802010 ATH IC - U-PAD2 - 1)

	Reten. Time [min]	Area [mV.s]	Height [mV]	Area [%]	Height [%]	W05 [min]	Compound Name
1	28.744	84.737	2.309	0.7	1.2	0.60	
2	37.176	12472.986	182.587	99.3	98.8	1.02	
	Total	12557.723	184.896	100.0	100.0		

1-Isopropoxy-3-(phenylsulfonyl)propan-2-one

^{1}H NMR (400 MHz, $CDCl_3$)

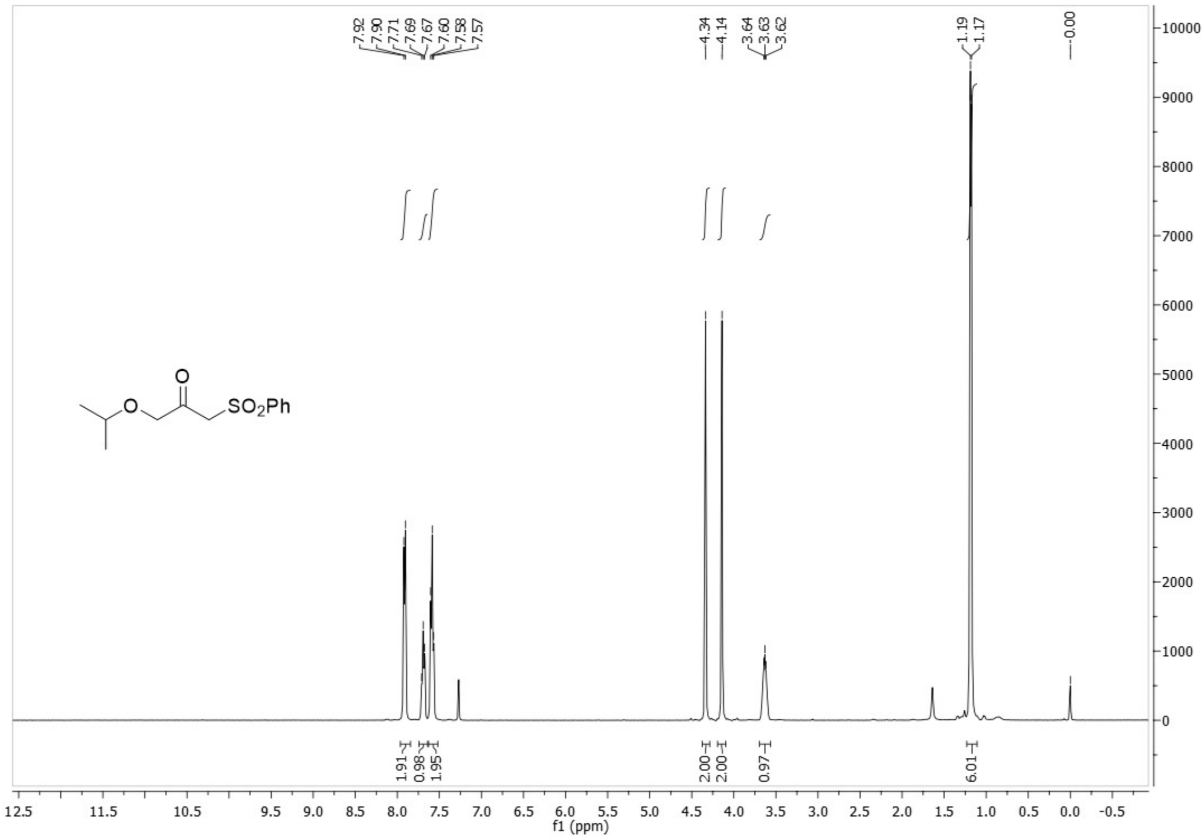

^{13}C NMR (101 MHz, $CDCl_3$

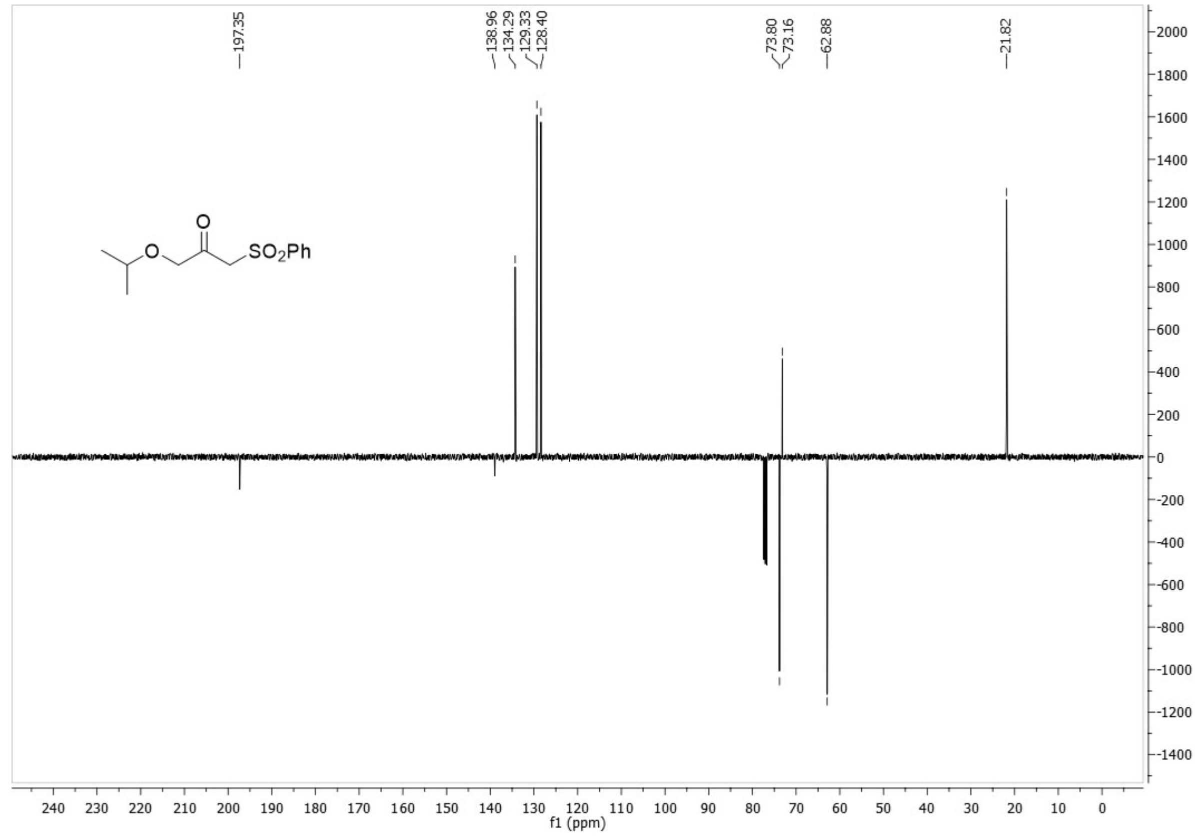

Ketone HPLC of 1-Isopropoxy-3-(phenylsulfonyl)propan-2-one.

Clarity - Chromatography SW

DataApex 2006

www.dataapex.com

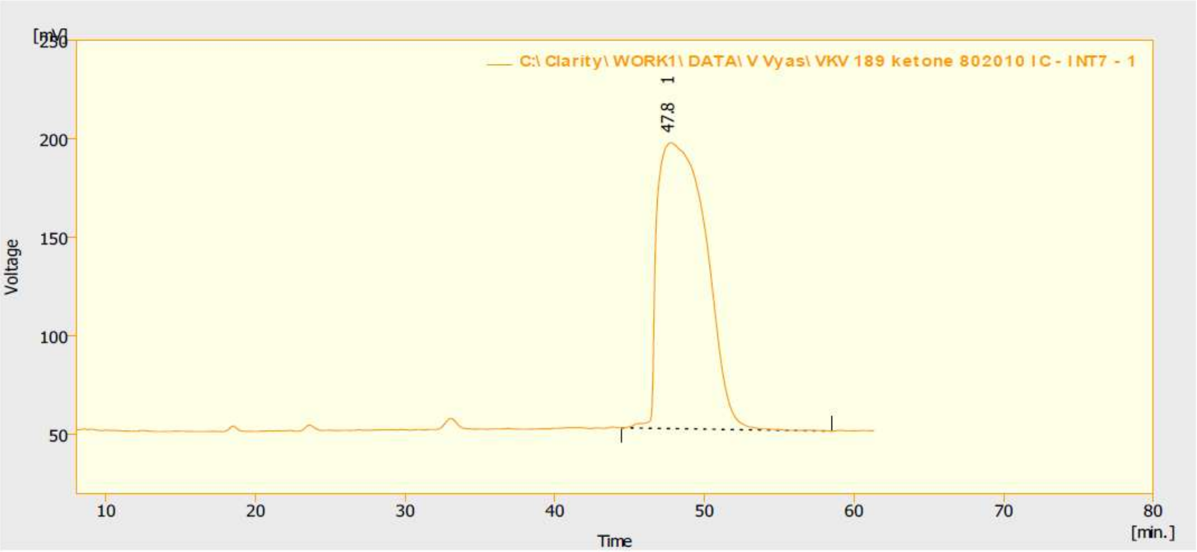

Result Table (Uncal - C:\Clarity\WORK1\DATA\V Vyas\VKV 189 ketone 802010 IC - INT7 - 1)

	Reten. Time [min]	Area [mV.s]	Height [mV]	Area [%]	Height [%]	W05 [min]	Compound Name
1	47.753	32921.493	145.199	100.0	100.0	3.85	
	Total	32921.493	145.199	100.0	100.0		

1-(Allyloxy)-3-(phenylsulfonyl)propan-2-ol 16

^{1}H NMR (400 MHz, $CDCl_3$)

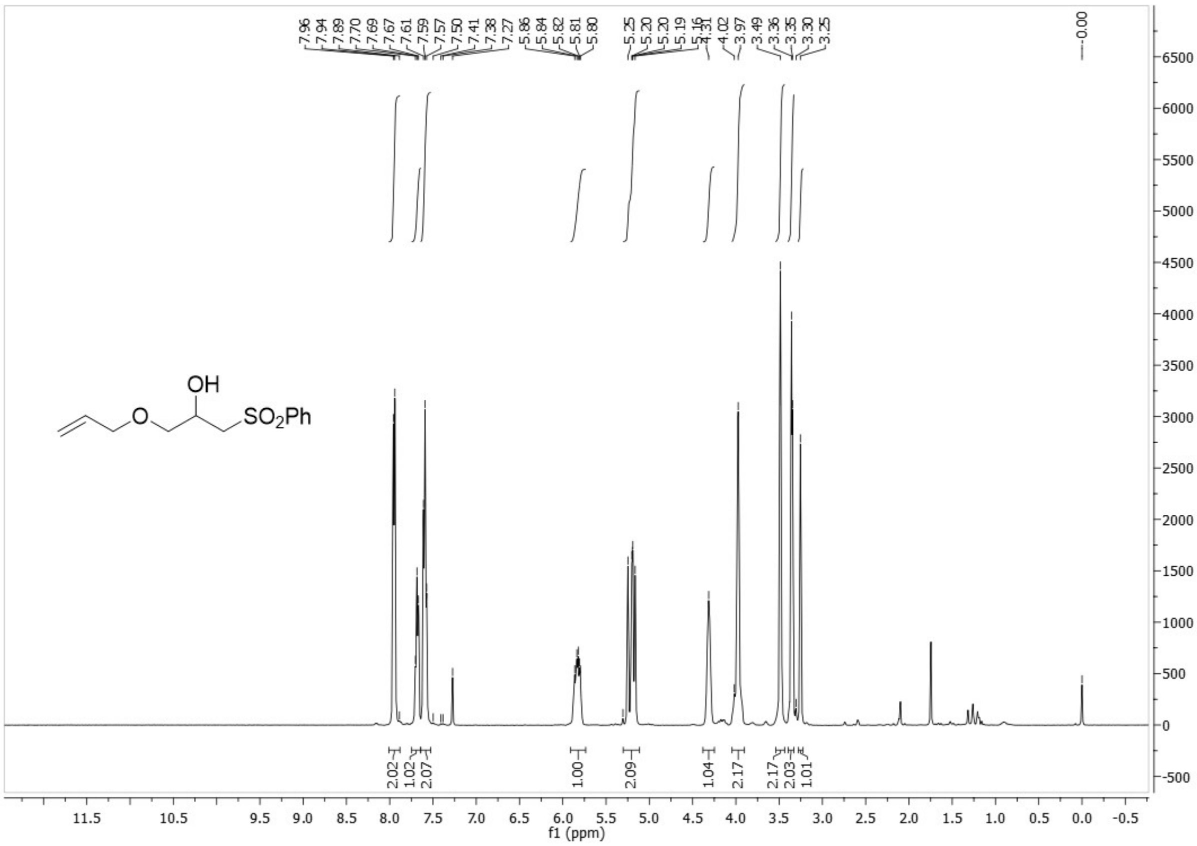

^{13}C NMR (101 MHz, $CDCl_3$

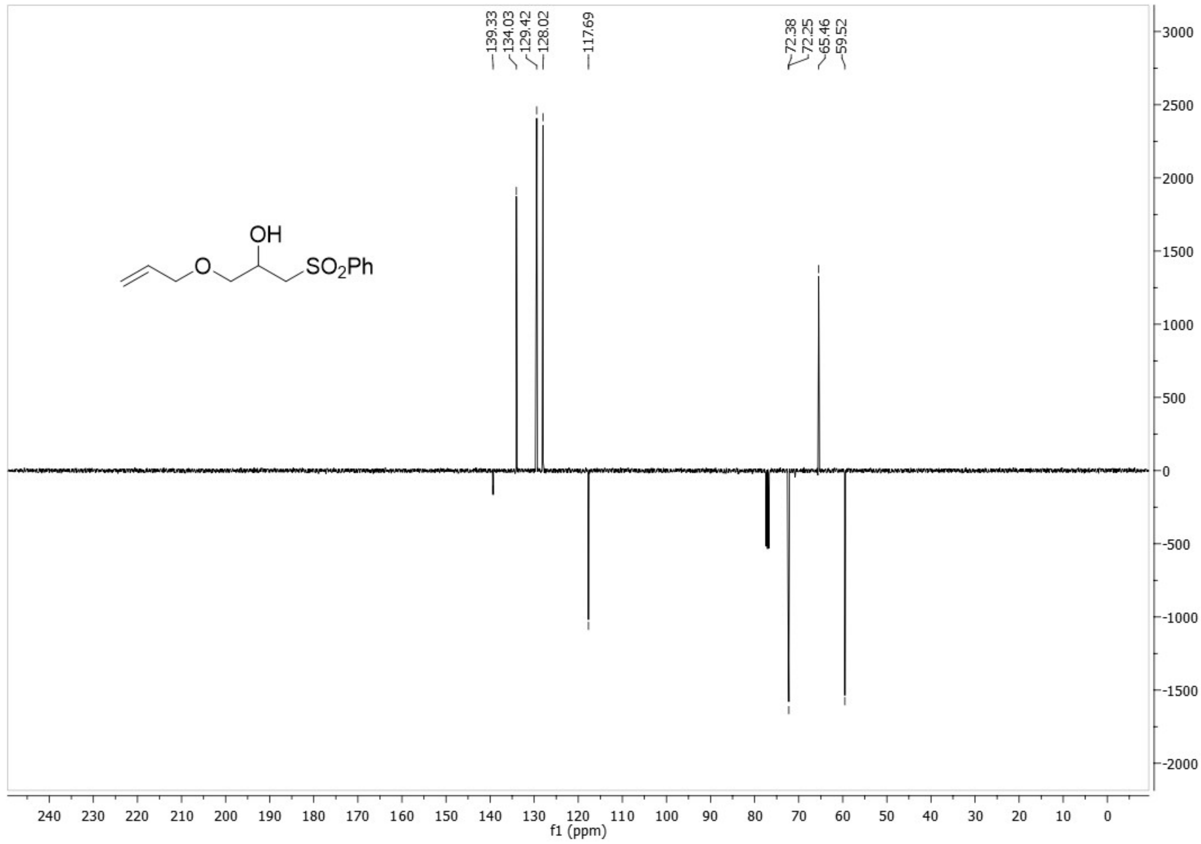

Racemic HPLC of 1-(Allyloxy)-3-(phenylsulfonyl)propan-2-ol.

Clarity - Chromatography SW

DataApex 2006

www.dataapex.com

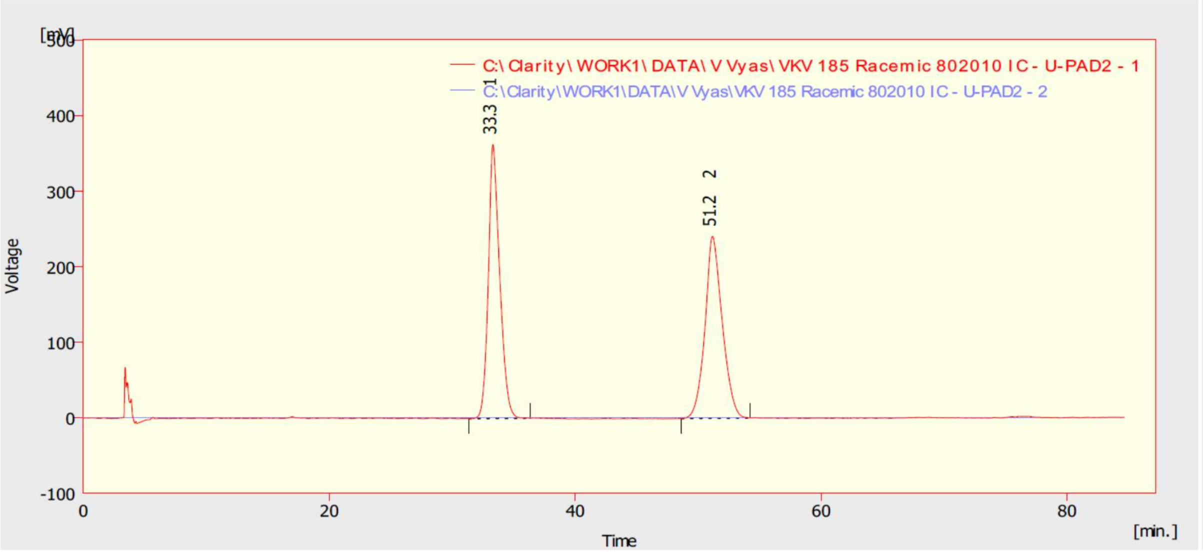

Result Table (Uncal - C:\Clarity\WORK1\DATA\V Vyas\VKV 185 Racemic 802010 IC - U-PAD2 - 1)

	Reten. Time [min]	Area [mV.s]	Height [mV]	Area [%]	Height [%]	W05 [min]	Compound Name
1	33.308	23336.406	362.750	49.8	60.1	0.96	
2	51.168	23491.046	240.831	50.2	39.9	1.45	
	Total	46827.452	603.582	100.0	100.0		

HPLC after ATH 1-(Allyloxy)-3-(phenylsulfonyl)propan-2-ol. (100% conversion, 95.4% ee).

Clarity - Chromatography SW

DataApex 2006

www.dataapex.com

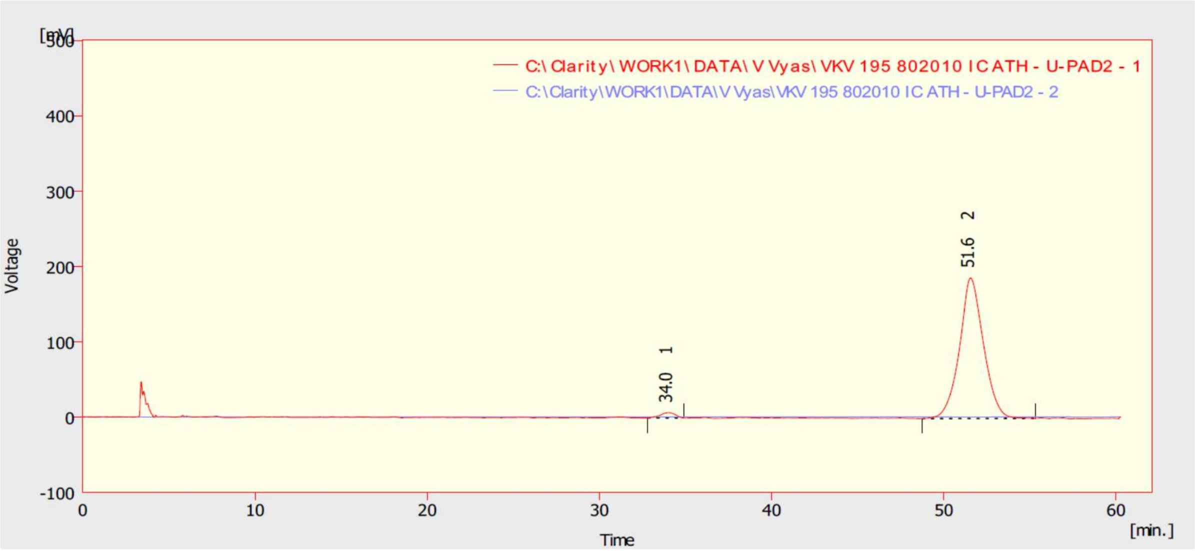

Result Table (Uncal - C:\Clarity\WORK1\DATA\V Vyas\VKV 195 802010 IC ATH - U-PAD2 - 1)

	Reten. Time [min]	Area [mV.s]	Height [mV]	Area [%]	Height [%]	W05 [min]	Compound Name
1	34.016	427.585	7.054	2.3	3.6	0.97	
2	51.552	18109.554	186.618	97.7	96.4	1.44	
	Total	18537.139	193.672	100.0	100.0		

1-(Allyloxy)-3-(phenylsulfonyl)propan-2-one

^{1}H NMR (400 MHz, $CDCl_3$)

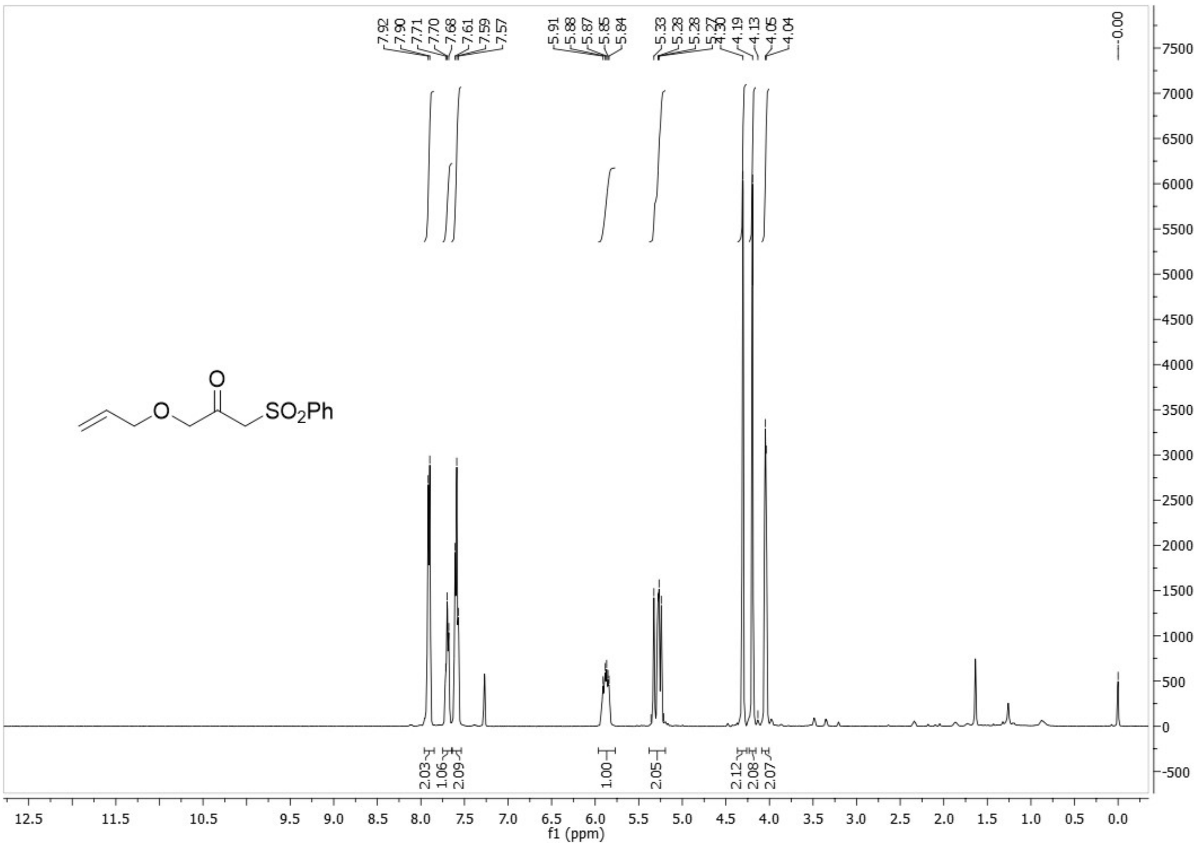

^{13}C NMR (101 MHz, $CDCl_3$

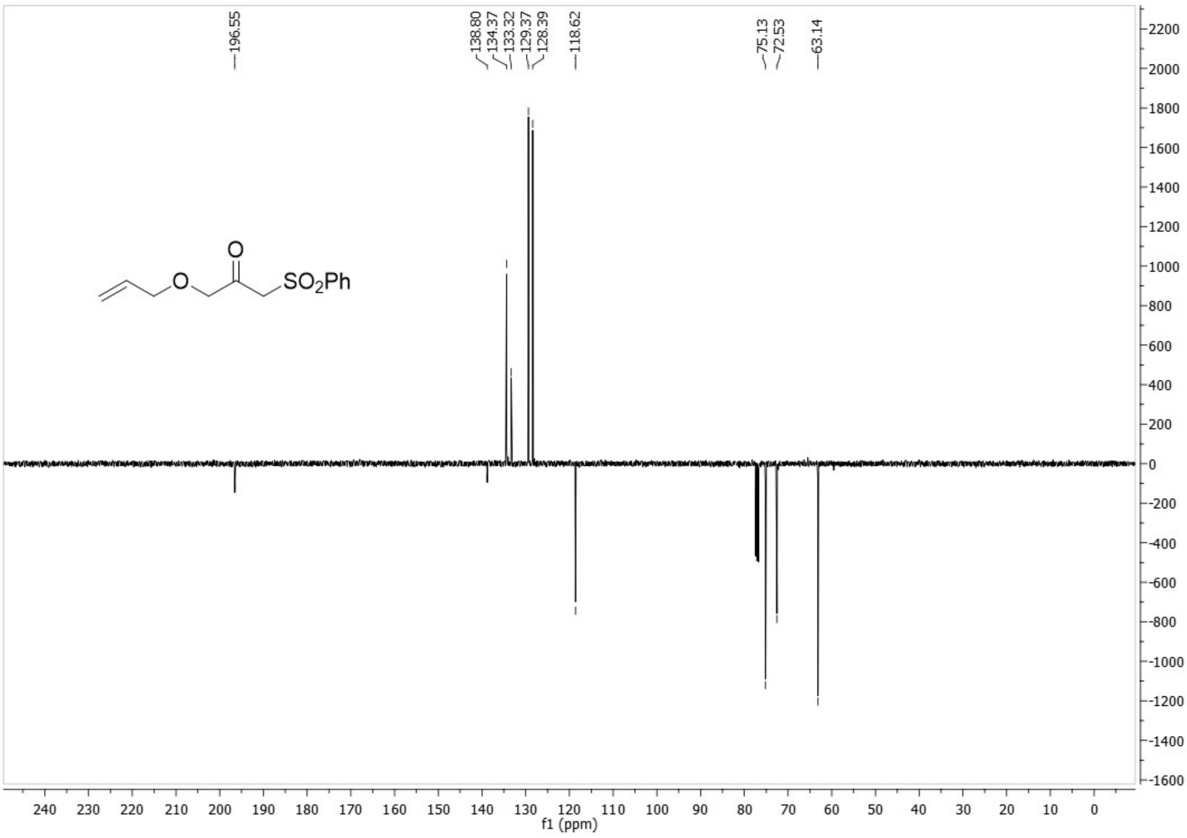

Ketone HPLC of 1-(Allyloxy)-3-(phenylsulfonyl)propan-2-one.

Clarity - Chromatography SW
DataApex 2006
www.dataapex.com

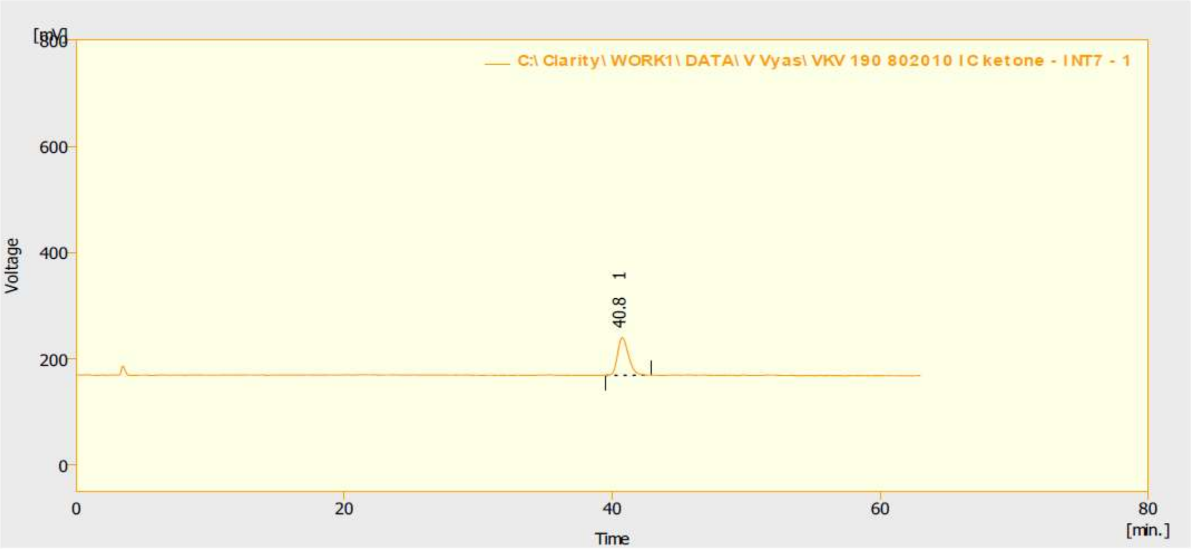

Result Table (Uncal - C:\Clarity\WORK1\DATA\V Vyas\VKV 190 802010 IC ketone - INT7 - 1)

	Reten. Time [min]	Area [mV.s]	Height [mV]	Area [%]	Height [%]	W05 [min]	Compound Name
1	40.760	4046.493	70.887	100.0	100.0	0.88	
	Total	4046.493	70.887	100.0	100.0		

1-Phenoxy-4-(phenylsulfonyl)butan-2-ol 17

^{1}H NMR (400 MHz, $CDCl_3$)

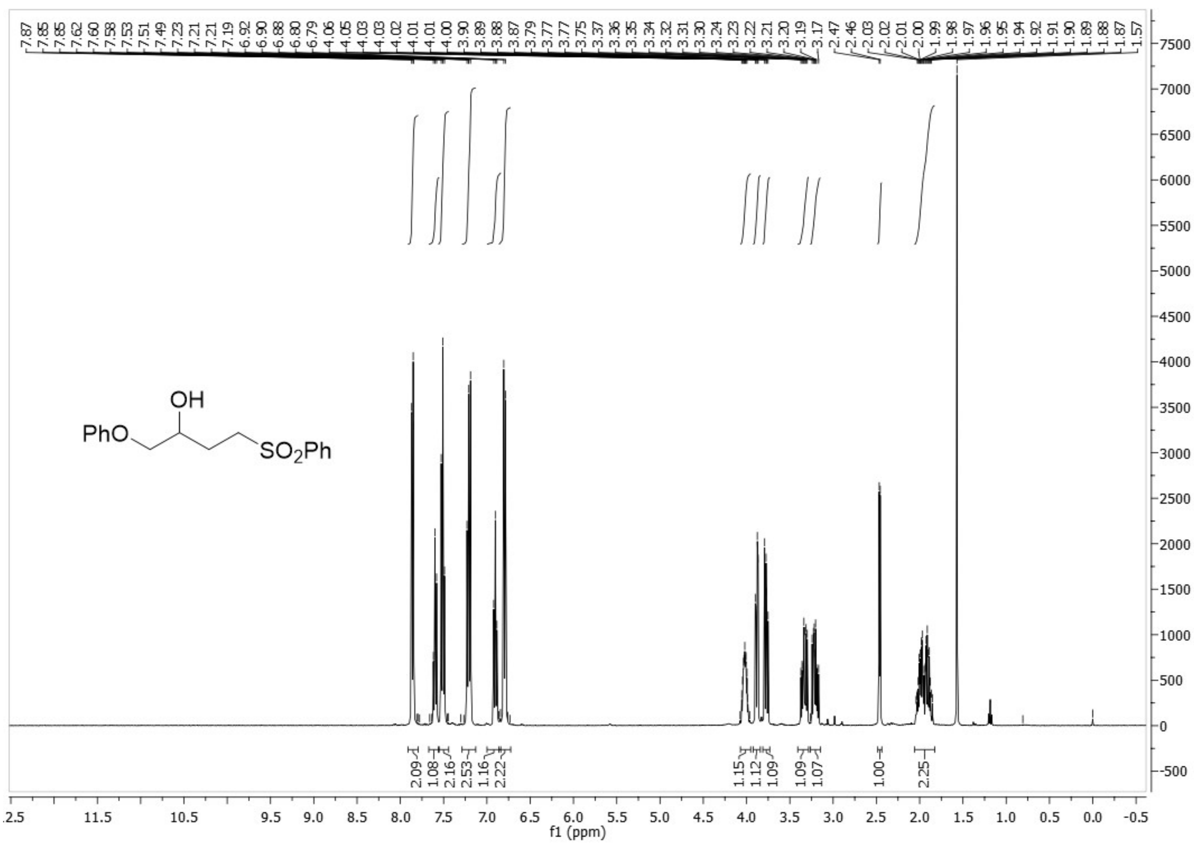

^{13}C NMR (101 MHz, $CDCl_3$

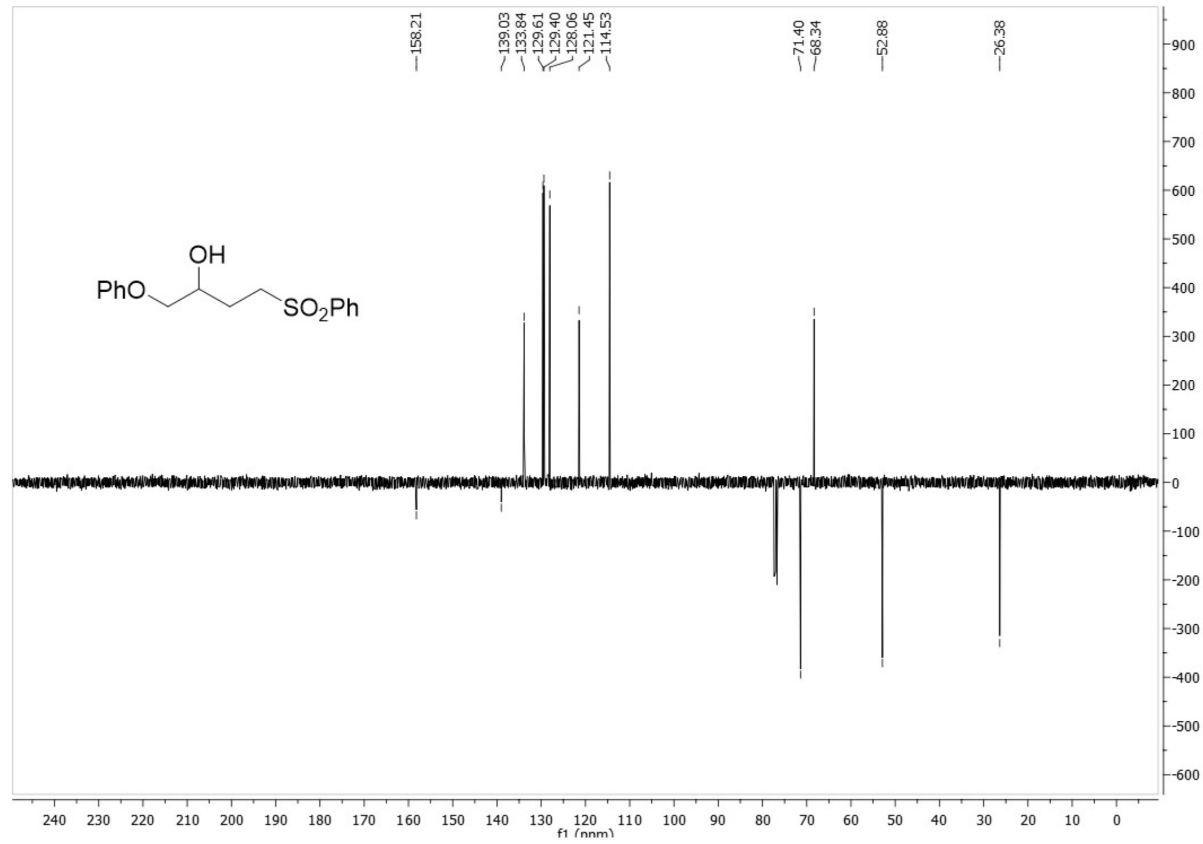

Racemic HPLC of 1-Phenoxy-4-(phenylsulfonyl)butan-2-ol.

Data File C:\CHEM32\1\DATA\DEF_LC 2019-07-05 13-20-37\001-0101.D
Sample Name: VKV 294

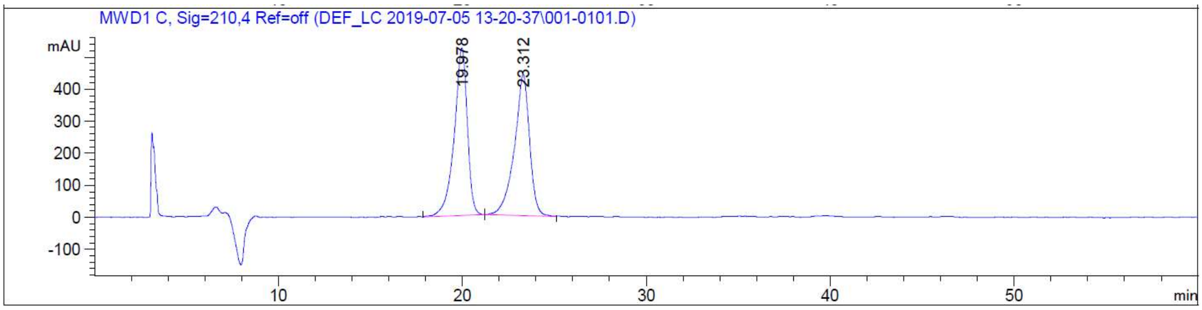

Signal 2: MWD1 C, Sig=210,4 Ref=off

Peak #	RetTime [min]	Type	Width [min]	Area [mAU*s]	Height [mAU]	Area %
1	19.978	BB	0.7343	2.59280e4	523.05493	50.5892
2	23.312	BV	0.8288	2.53241e4	440.41031	49.4108

HPLC after ATH 1-Phenoxy-4-(phenylsulfonyl)butan-2-ol. (100% conversion, 27% ee).

Data File C:\CHEM32\1\DATA\DEF_LC 2019-07-08 12-38-36\001-0101.D
Sample Name: VKV 297

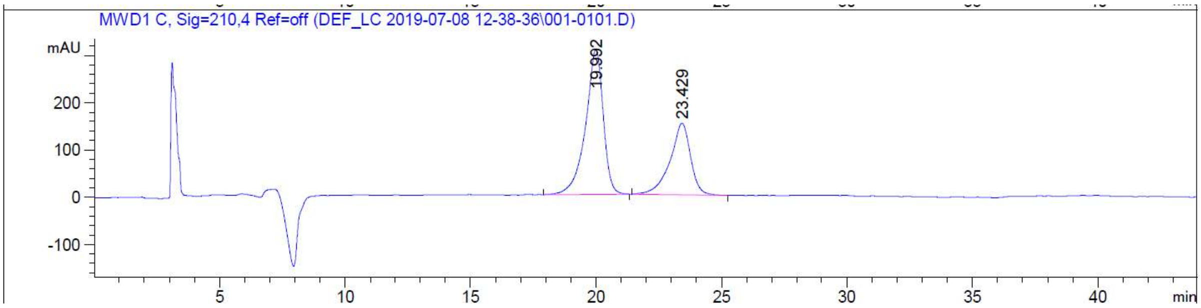

Signal 2: MWD1 C, Sig=210,4 Ref=off

Peak #	RetTime [min]	Type	Width [min]	Area [mAU*s]	Height [mAU]	Area %
1	19.992	BB	0.7194	1.49525e4	306.36484	63.4989
2	23.429	BB	0.8185	8595.11523	151.35109	36.5011

1-Phenoxy-4-(phenylsulfonyl)butan-2-one

^{1}H NMR (400 MHz, $CDCl_3$)

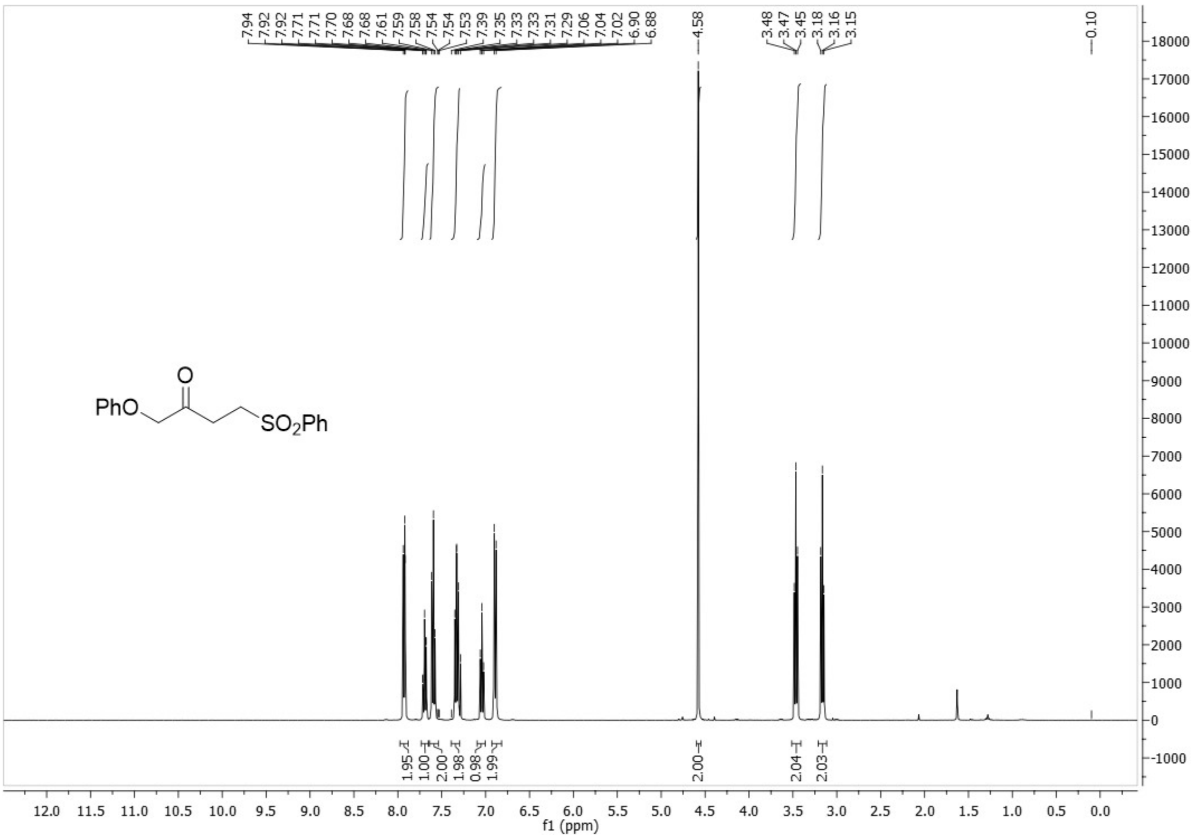

^{13}C NMR (101 MHz, $CDCl_3$

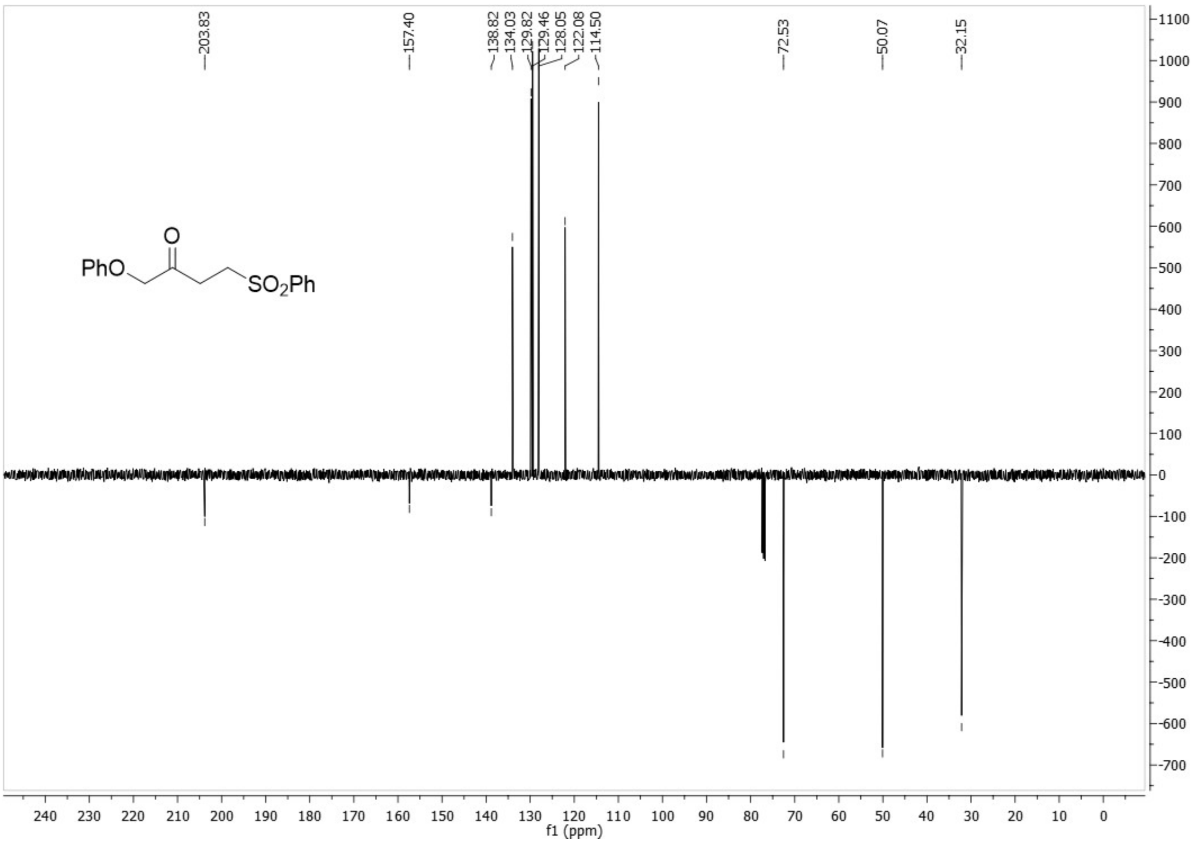

Ketone HPLC of 1-Phenoxy-4-(phenylsulfonyl)butan-2-one.

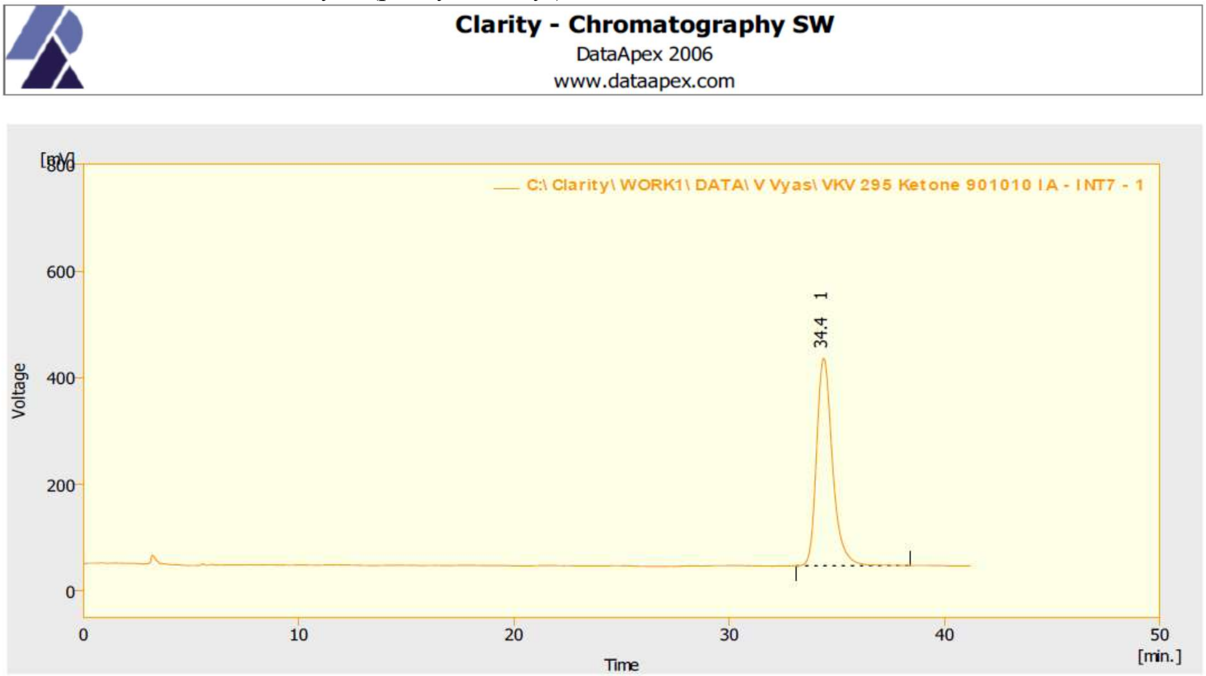

Result Table (Uncal - C:\Clarity\WORK1\DATA\V Vyas\VKV 295 Ketone 901010 IA - INT7 - 1)

	Reten. Time [min]	Area [mV.s]	Height [mV]	Area [%]	Height [%]	W05 [min]	Compound Name
1	34.393	19996.816	389.204	100.0	100.0	0.77	
	Total	19996.816	389.204	100.0	100.0		

***tert*-Butyl (2-hydroxy-3-(phenylsulfonyl)propyl)carbamate 18**

^{1}H NMR (400 MHz, $CDCl_3$)

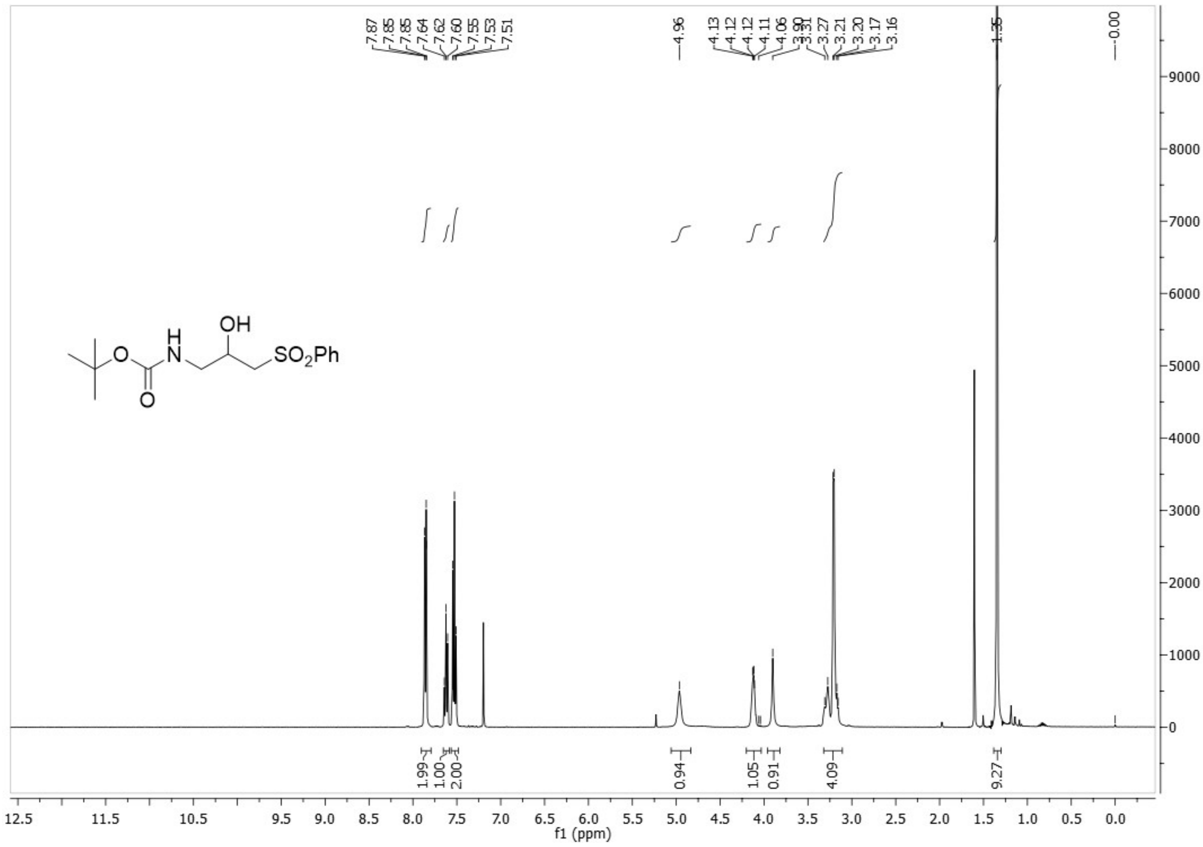

^{13}C NMR (101 MHz, $CDCl_3$

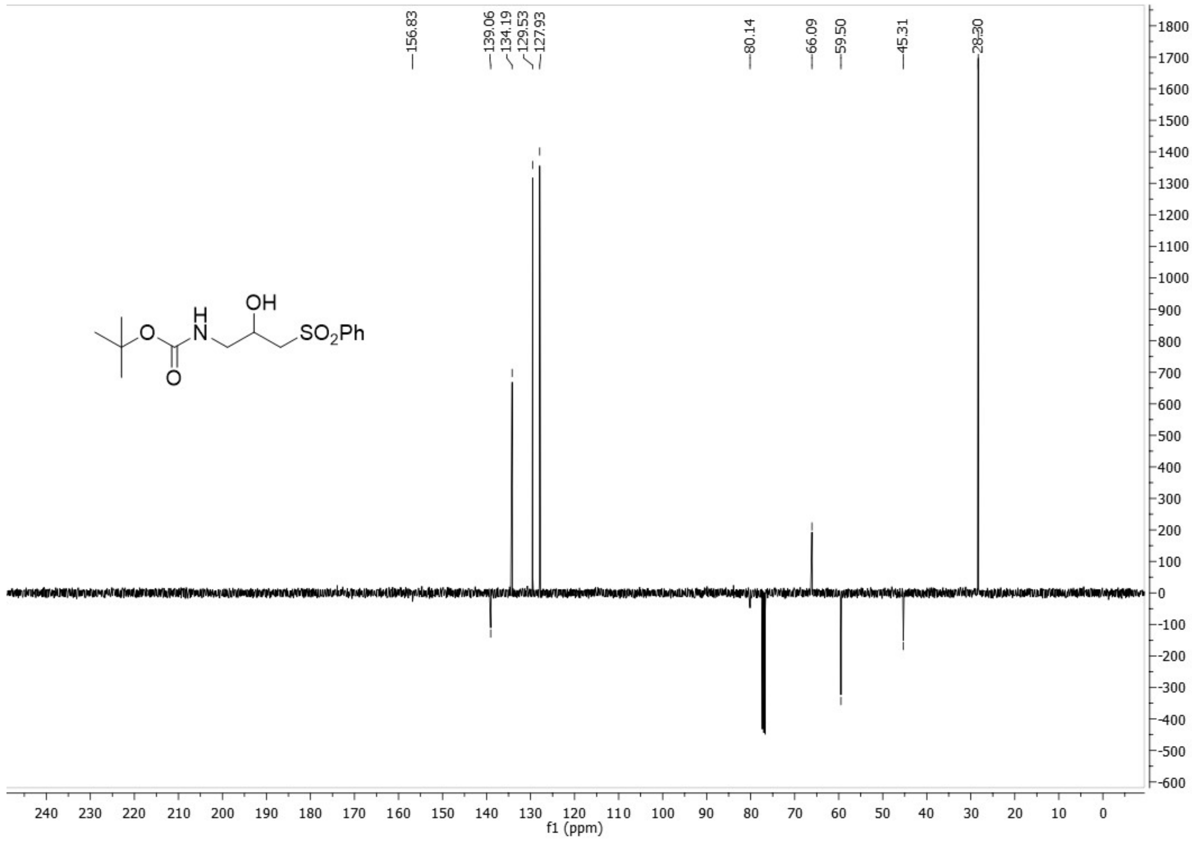

Racemic HPLC of *tert*-Butyl (2-hydroxy-3-(phenylsulfonyl)propyl)carbamate.

```
Data File C:\CHEM32\1\DATA\DEF_LC 2019-07-29 11-41-07\001-0101.D
Sample Name: VKV 314 901007 Rac IA
```

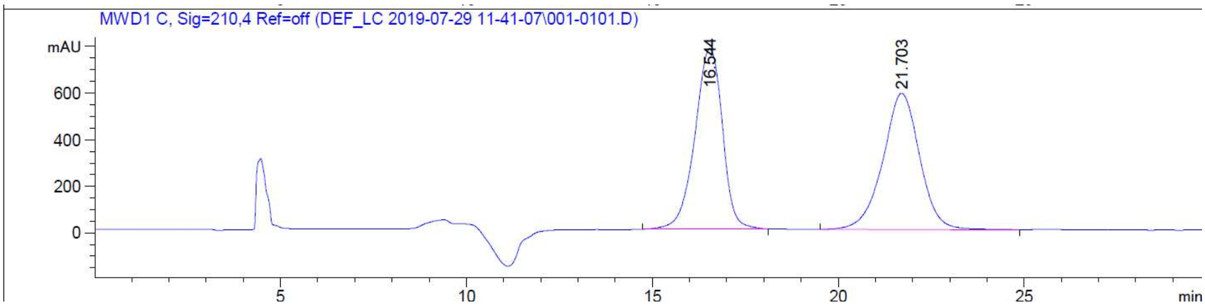

Signal 2: MWD1 C, Sig=210,4 Ref=off

Peak #	RetTime [min]	Type	Width [min]	Area [mAU*s]	Height [mAU]	Area %
1	16.544	BV	0.8023	4.01874e4	775.82172	50.0225
2	21.703	BB	1.0320	4.01512e4	584.06812	49.9775

HPLC after ATH *tert*-Butyl (2-hydroxy-3-(phenylsulfonyl)propyl)carbamate. (100% conversion, 53.5% ee).

```
Data File C:\CHEM32\1\DATA\DEF_LC 2019-07-29 12-13-11\001-0101.D
Sample Name: VKV 323 901007 ATH IA
```

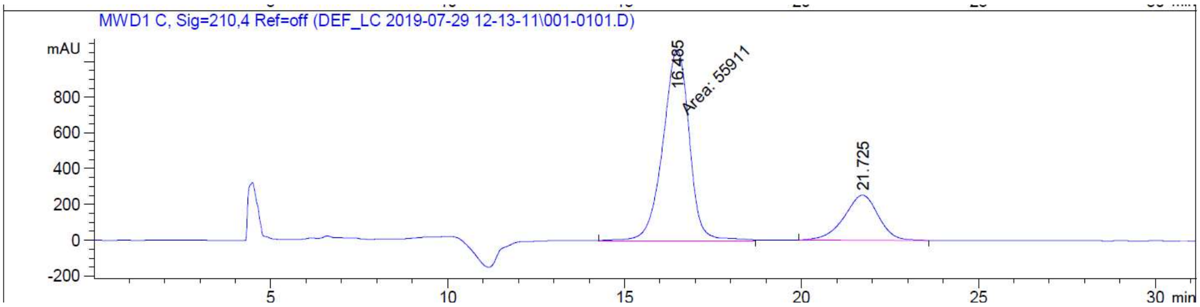

Signal 2: MWD1 C, Sig=210,4 Ref=off

Peak #	RetTime [min]	Type	Width [min]	Area [mAU*s]	Height [mAU]	Area %
1	16.485	MM	0.8718	5.59110e4	1068.90051	76.7436
2	21.725	VB	1.0072	1.69433e4	252.40210	23.2564

***tert*-Butyl 2-oxo-3-(phenylsulfonyl)propyl)carbamate**

^{1}H NMR (400 MHz, $CDCl_3$)

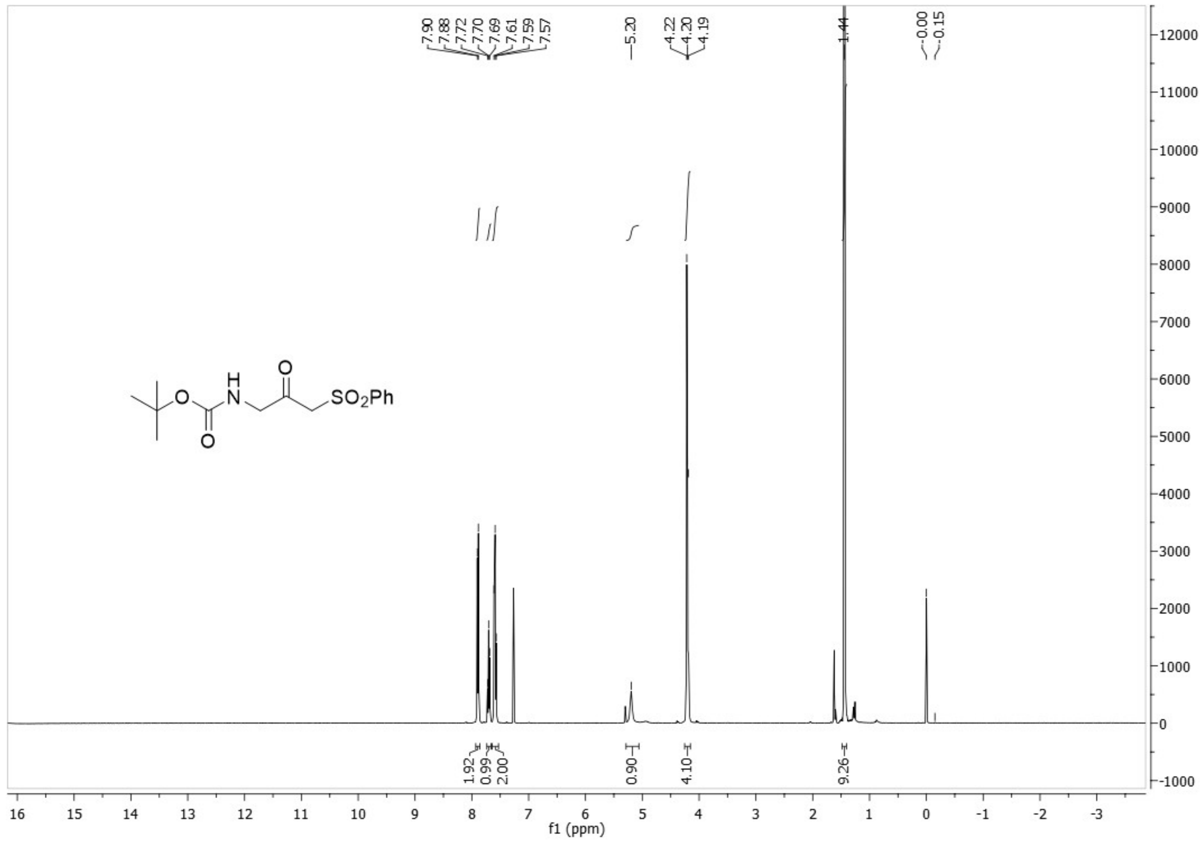

^{13}C NMR (101 MHz, $CDCl_3$

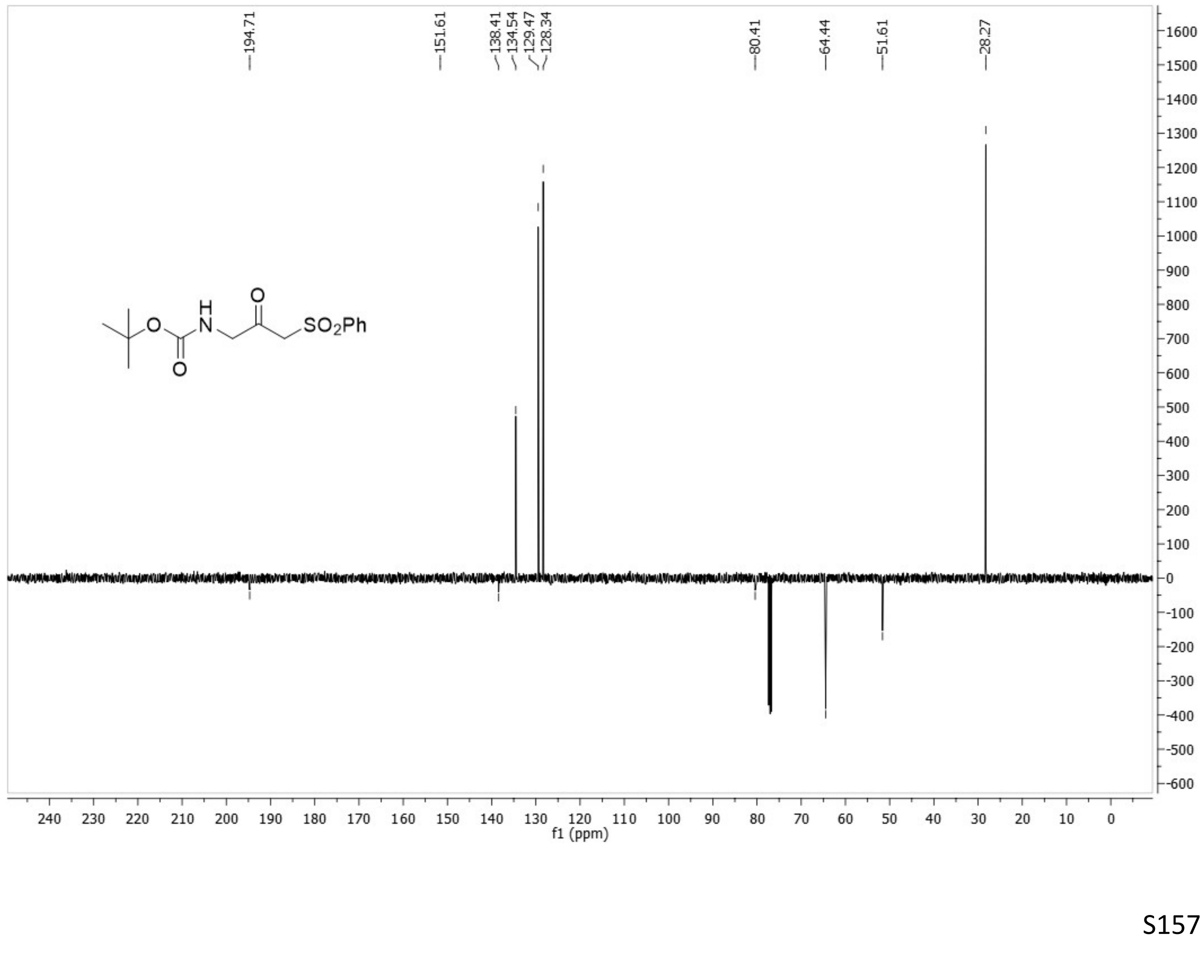

Ketone HPLC of *tert*-Butyl 2-oxo-3-(phenylsulfonyl)propyl)carbamate.

Clarity - Chromatography SW

DataApex 2006

www.dataapex.com

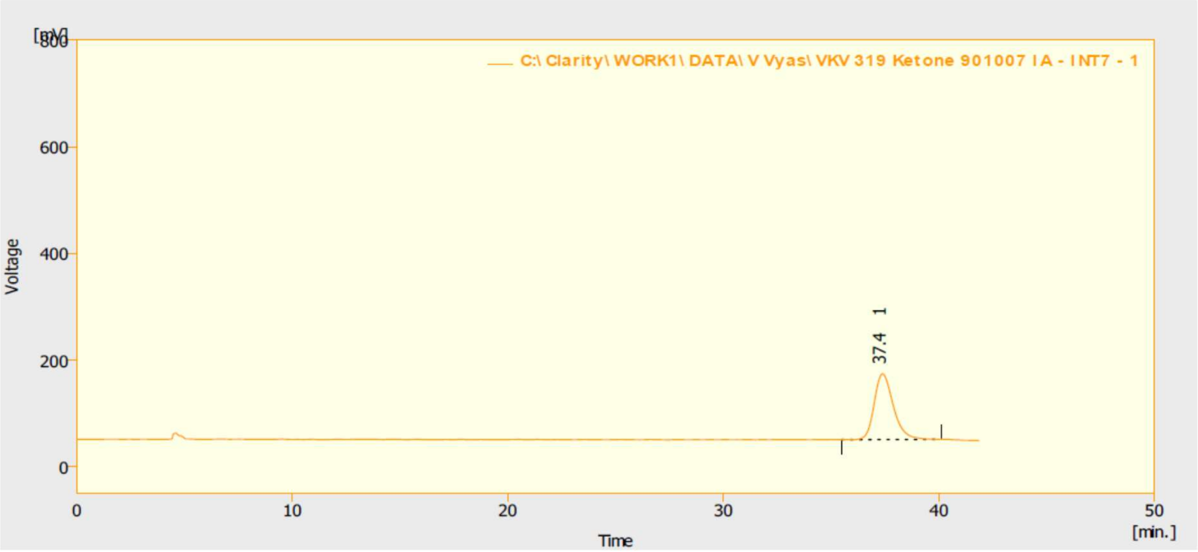

Result Table (Uncal - C:\Clarity\WORK1\DATA\V Vyas\VKV 319 Ketone 901007 IA - INT7 - 1)

	Reten. Time [min]	Area [mV.s]	Height [mV]	Area [%]	Height [%]	W05 [min]	Compound Name
1	37.390	7622.060	123.412	100.0	100.0	0.93	
	Total	7622.060	123.412	100.0	100.0		

3-Phenyl-1-(phenylsulfonyl)-1-(p-tolyl)propan-2-ol 20a

^{1}H NMR (400 MHz, $CDCl_3$)

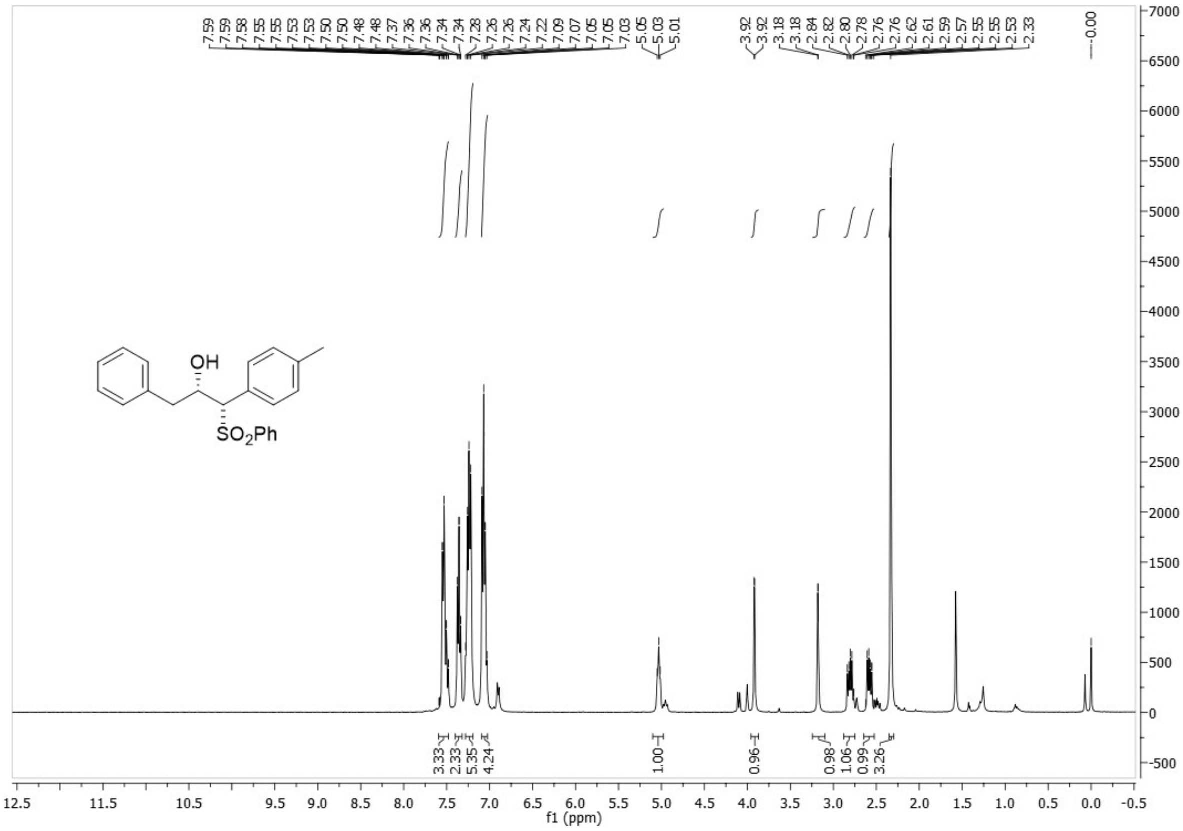

^{13}C NMR (101 MHz, $CDCl_3$)

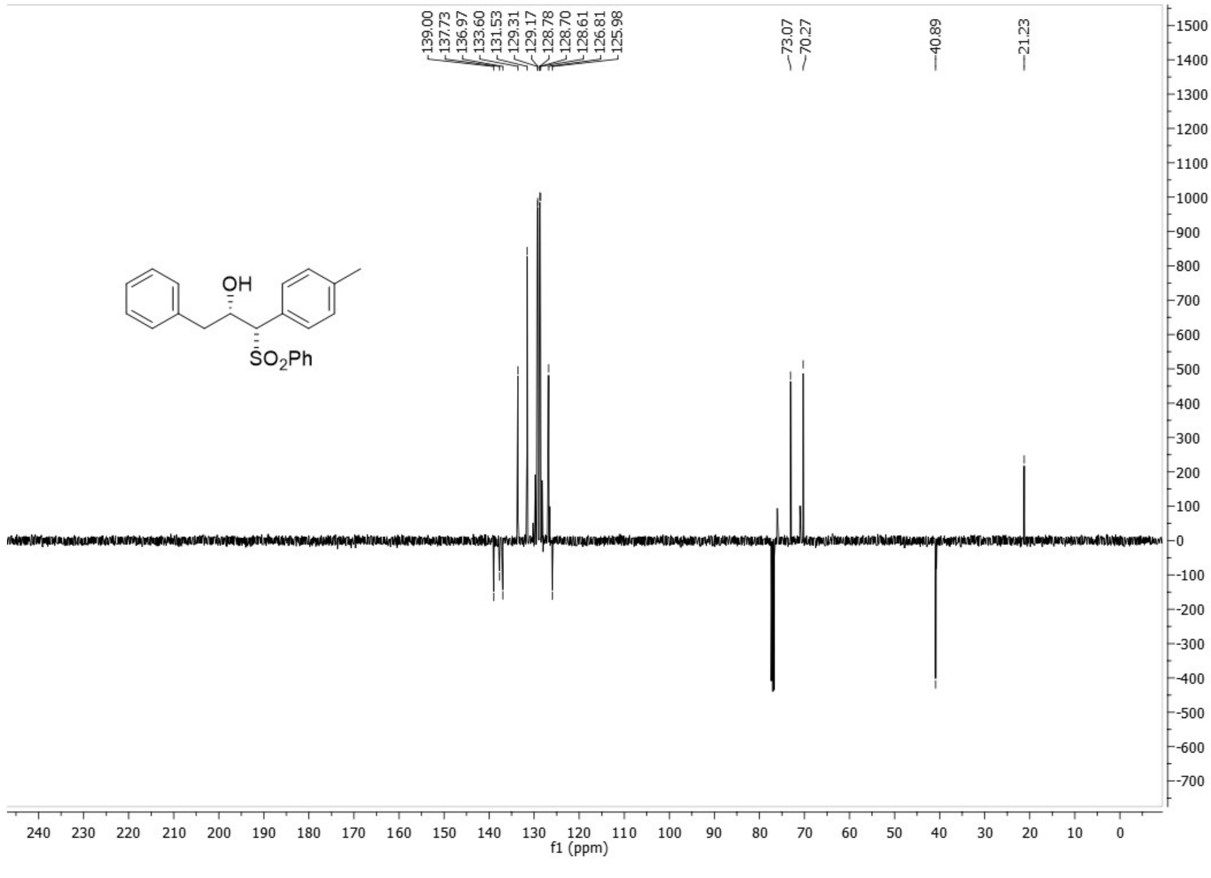

Racemic HPLC of 3-phenyl-1-(phenylsulfonyl)-1-(p-tolyl)propan-2-ol.

Clarity - Chromatography SW
DataApex 2006
www.dataapex.com

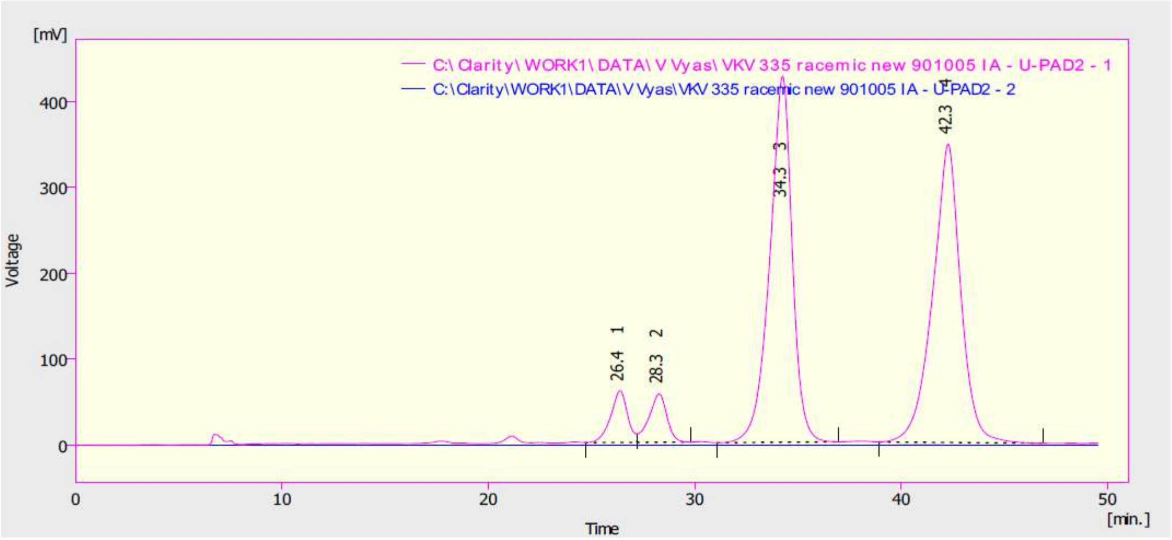

Result Table (Uncal - C:\Clarity\WORK1\DATA\V Vyas\VKV 335 racemic new 901005 IA - U-PAD2 - 1)

	Reten. Time [min]	Area [mV.s]	Height [mV]	Area [%]	Height [%]	W05 [min]	Compound Name
1	26.384	3435.503	60.318	4.8	6.8	0.85	
2	28.276	3405.885	56.419	4.7	6.3	0.91	
3	34.256	32264.924	426.157	44.8	47.9	1.12	
4	42.280	32963.632	347.679	45.7	39.0	1.38	
	Total	72069.944	890.573	100.0	100.0		

HPLC after ATH 3-phenyl-1-(phenylsulfonyl)-1-(p-tolyl)propan-2-ol. (100% conversion, 96.6:3.4 dr, >99.9% ee).

Clarity - Chromatography SW
DataApex 2006
www.dataapex.com

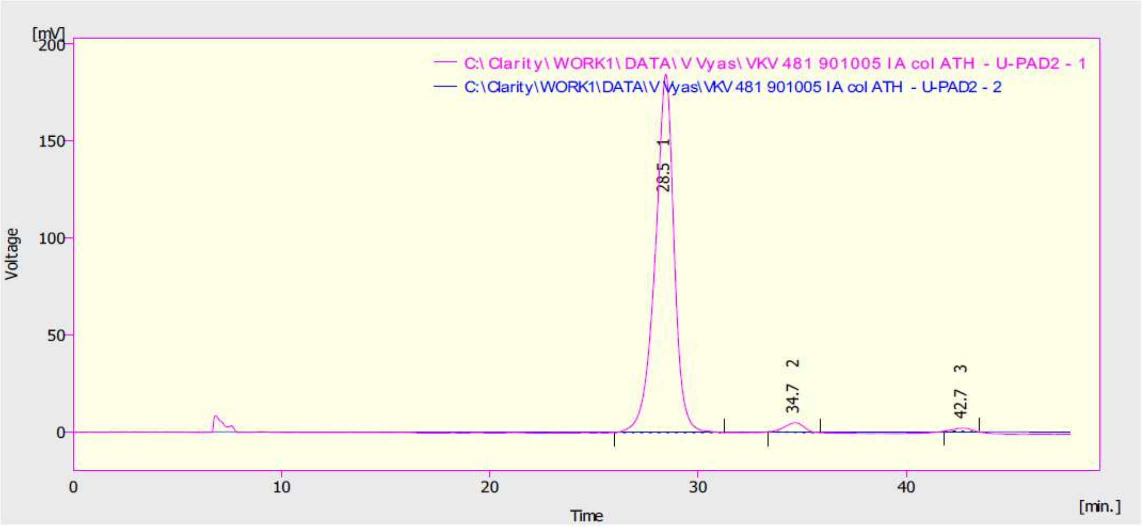

Result Table (Uncal - C:\Clarity\WORK1\DATA\V Vyas\VKV 481 901005 IA col ATH - U-PAD2 - 1)

	Reten. Time [min]	Area [mV.s]	Height [mV]	Area [%]	Height [%]	W05 [min]	Compound Name
1	28.456	11720.242	184.472	96.6	96.5	0.92	
2	34.680	316.196	4.961	2.6	2.6	1.02	
3	42.728	99.117	1.754	0.8	0.9	0.98	
	Total	12135.556	191.187	100.0	100.0		

3-Phenyl-1-(phenylsulfonyl)-1-(p-tolyl)propan-2-one 19a

^{1}H NMR (400 MHz, $CDCl_3$)

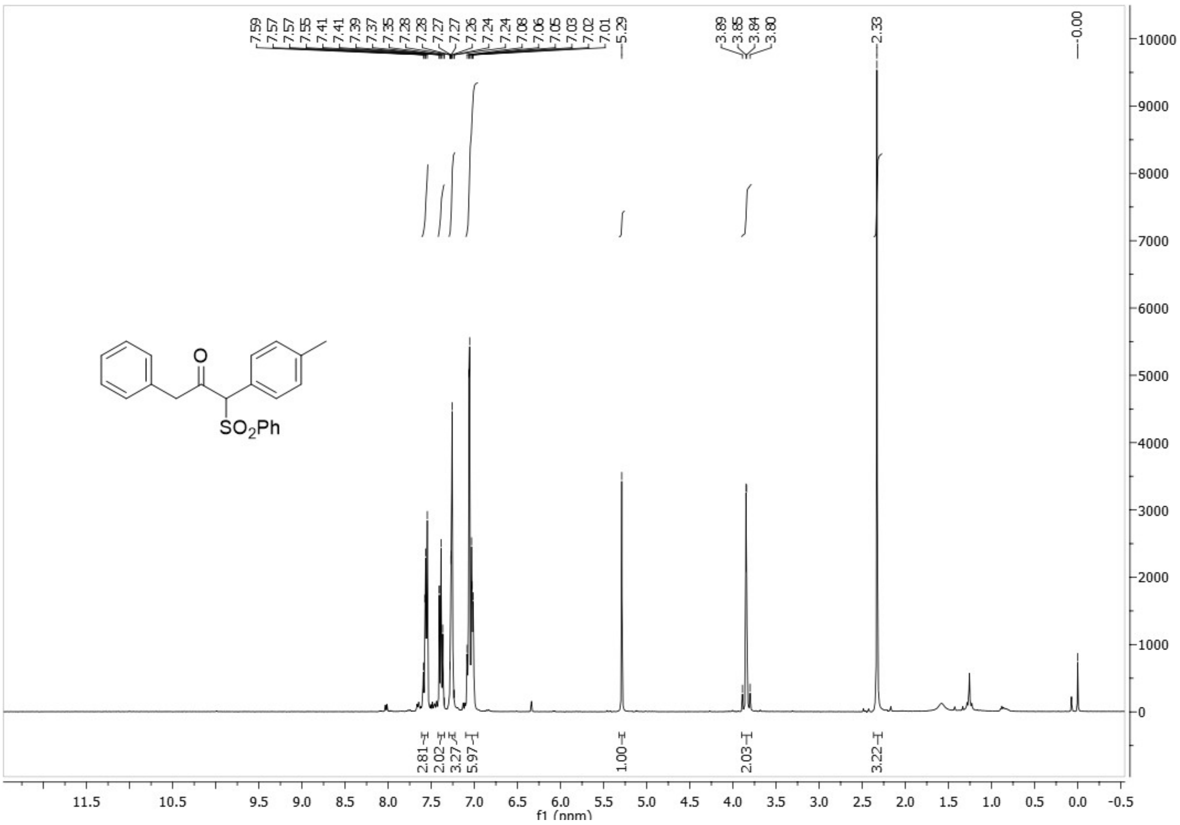

^{13}C NMR (101 MHz, $CDCl_3$)

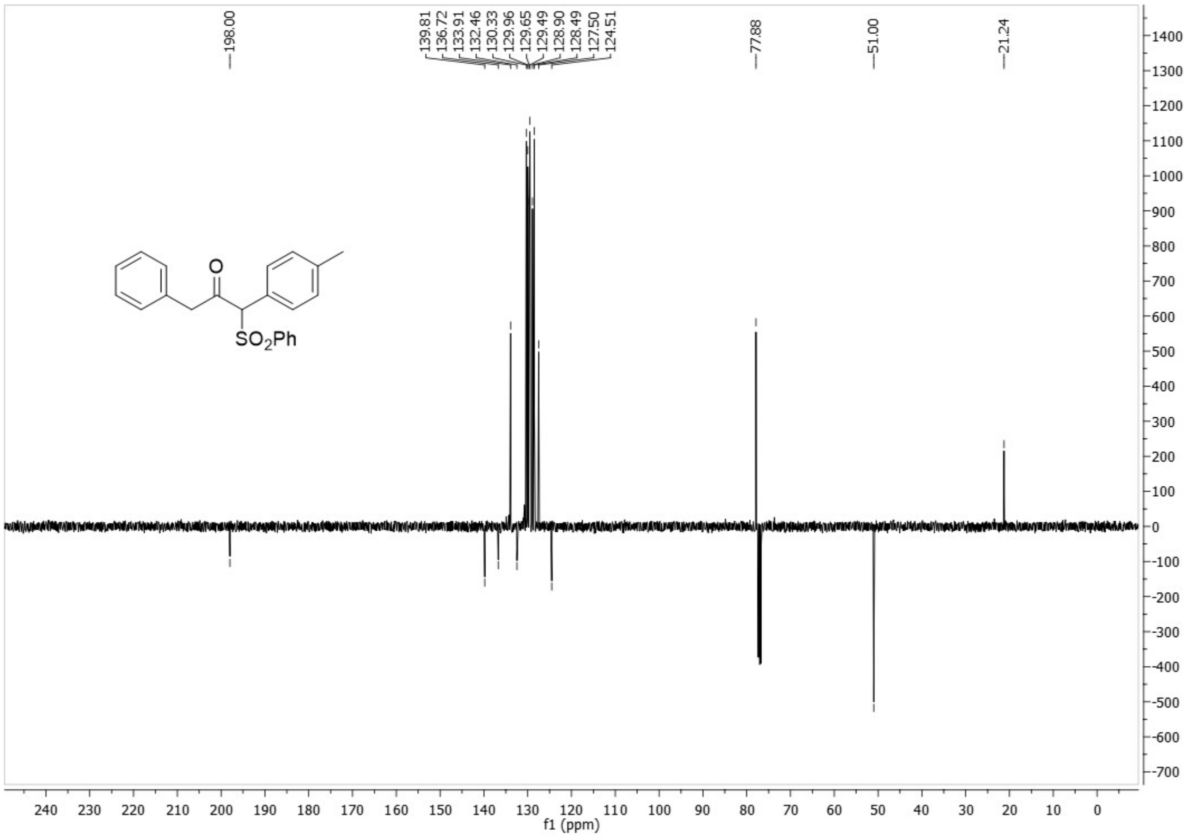

Ketone HPLC of 3-phenyl-1-(phenylsulfonyl)-1-(p-tolyl)propan-2-one.

Clarity - Chromatography SW
DataApex 2006
www.dataapex.com

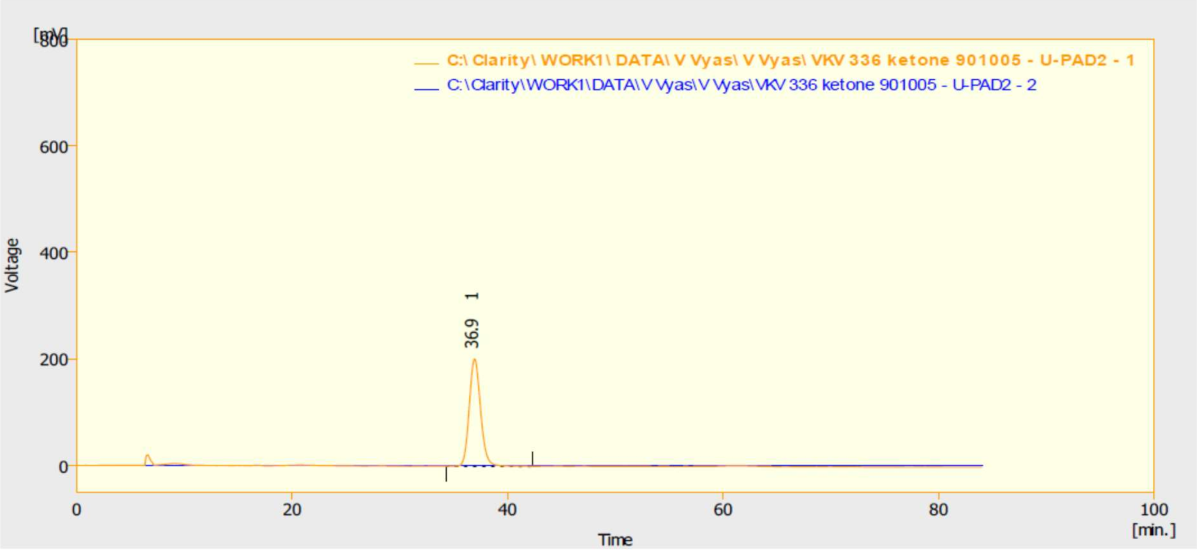

Result Table (Uncal - C:\Clarity\WORK1\DATA\V Vyas\V Vyas\VKV 336 ketone 901005 - U-PAD2 - 1)

	Reten. Time [min]	Area [mV.s]	Height [mV]	Area [%]	Height [%]	W05 [min]	Compound Name
1	36.944	14871.371	201.608	100.0	100.0	1.12	
	Total	14871.371	201.608	100.0	100.0		

1-Phenoxy-3-(phenylsulfonyl)butan-2-ol 20b

^{1}H NMR (400 MHz, $CDCl_3$)

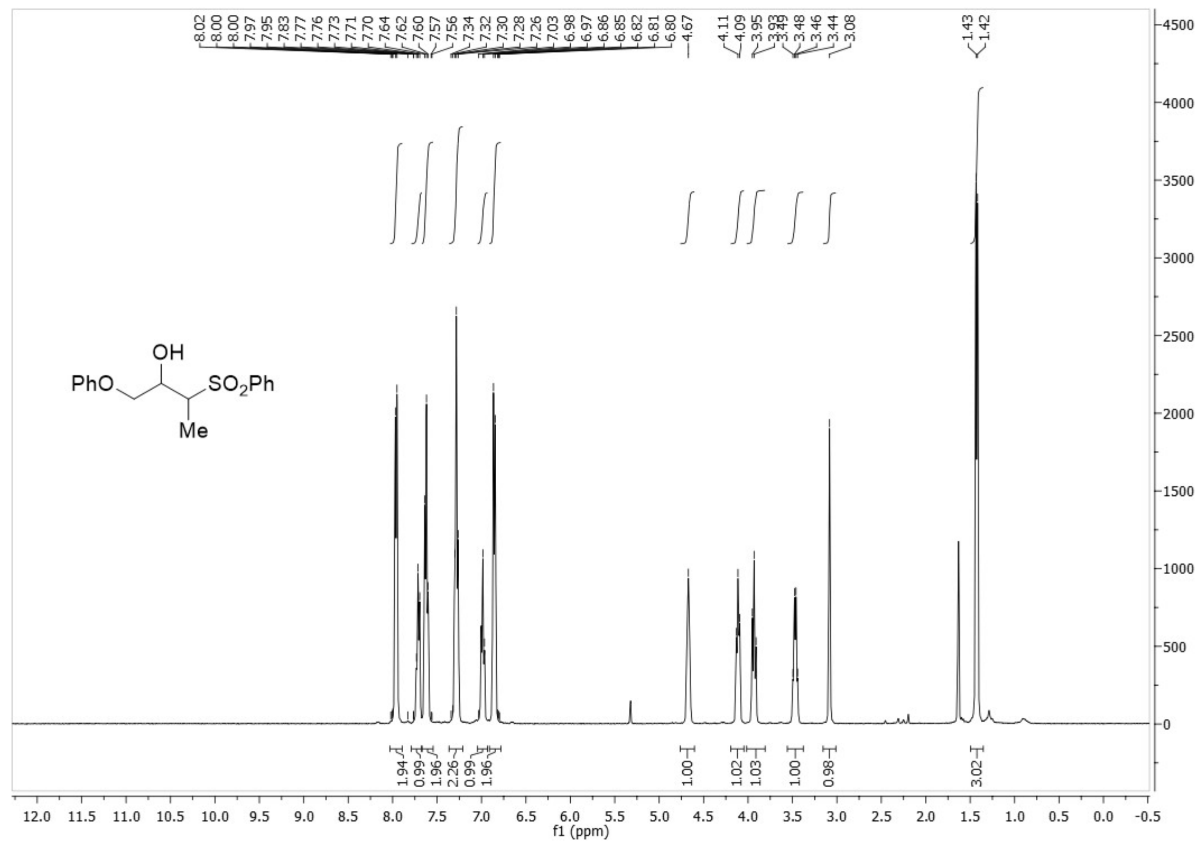

^{13}C NMR (101 MHz, $CDCl_3$

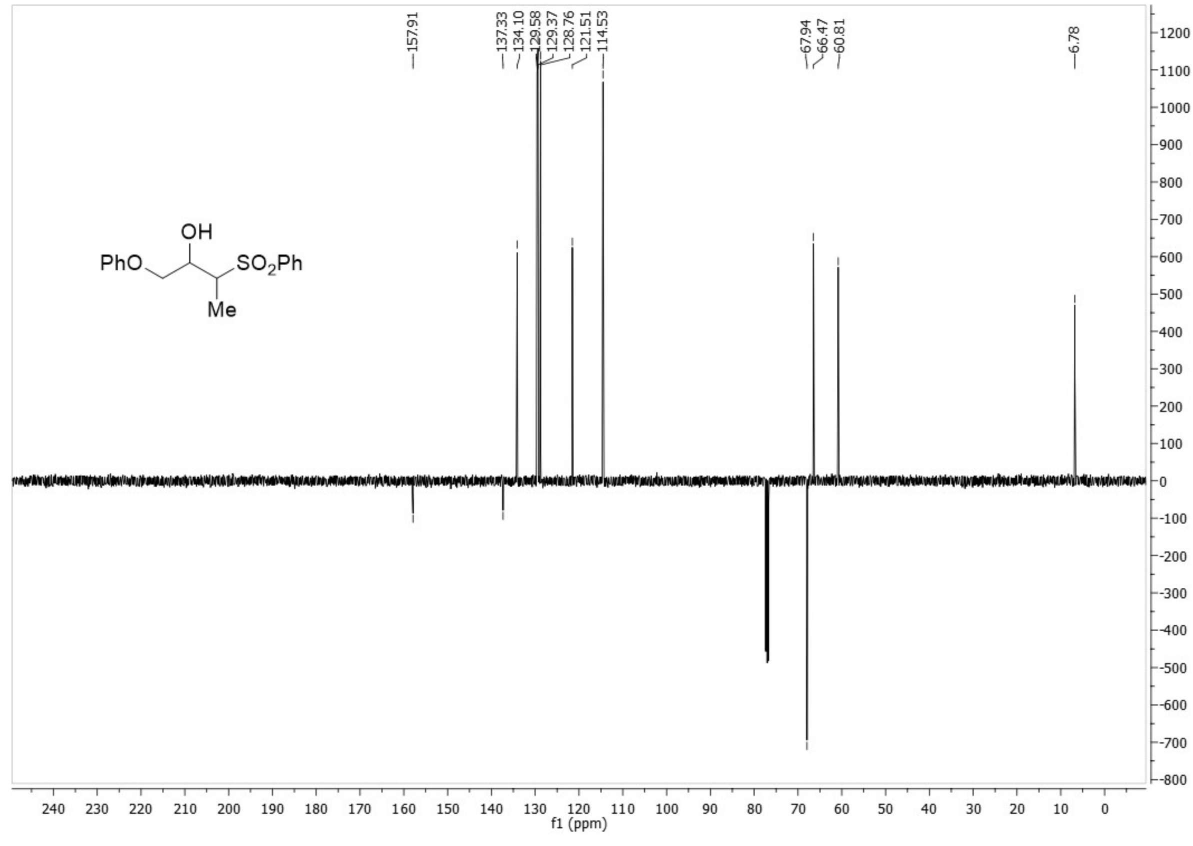

Racemic HPLC of 1-Phenoxy-3-(phenylsulfonyl)butan-2-ol.

Clarity - Chromatography SW
DataApex 2006
www.dataapex.com

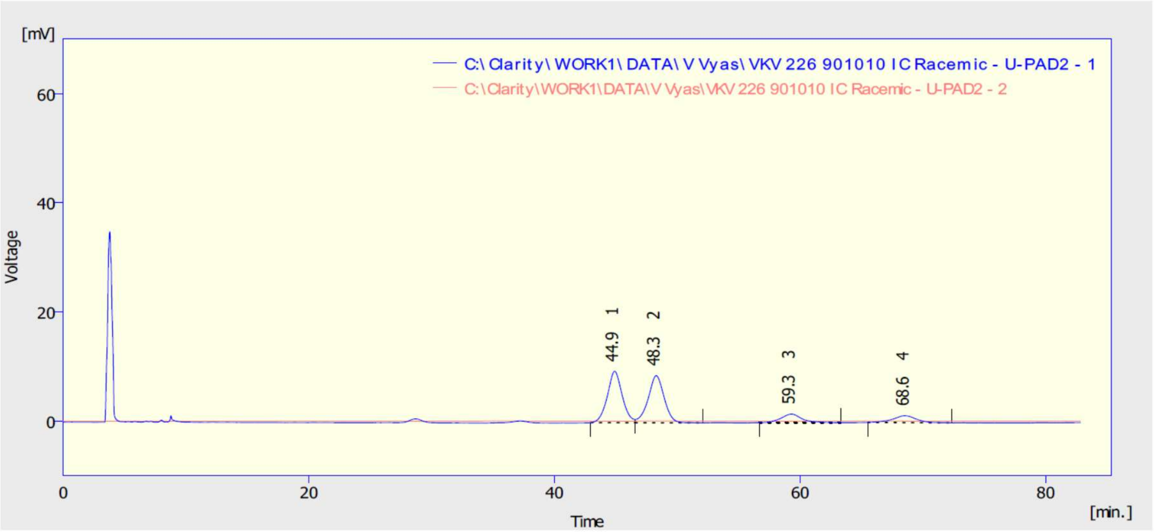

Result Table (Uncal - C:\Clarity\WORK1\DATA\V Vyas\VKV 226 901010 IC Racemic - U-PAD2 - 1)

	Reten. Time [min]	Area [mV.s]	Height [mV]	Area [%]	Height [%]	W05 [min]	Compound Name
1	44.912	794.351	9.442	41.1	45.1	1.27	
2	48.292	780.750	8.642	40.4	41.3	1.36	
3	59.296	188.719	1.582	9.8	7.6	1.70	
4	68.584	167.169	1.269	8.7	6.1	1.94	
	Total	1930.989	20.935	100.0	100.0		

HPLC after ATH 1-Phenoxy-3-(phenylsulfonyl)butan-2-ol. (100% conversion, 99.3:0.7 dr, >99.9% ee).

Clarity - Chromatography SW
DataApex 2006
www.dataapex.com

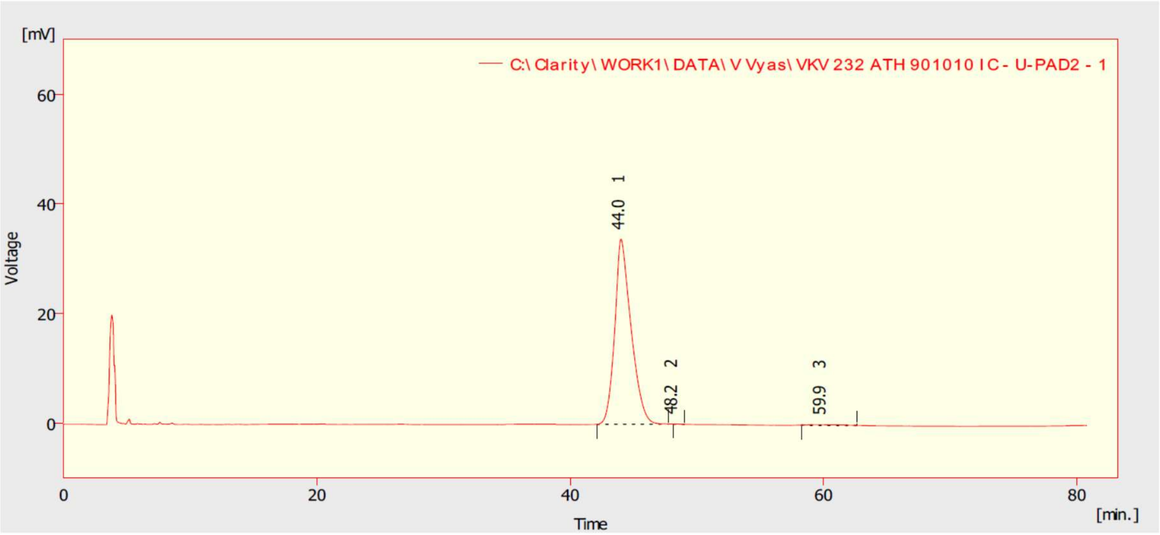

Result Table (Uncal - C:\Clarity\WORK1\DATA\V Vyas\VKV 232 ATH 901010 IC - U-PAD2 - 1)

	Reten. Time [min]	Area [mV.s]	Height [mV]	Area [%]	Height [%]	W05 [min]	Compound Name
1	44.008	3020.639	33.758	99.3	99.6	1.34	
2	48.172	0.409	0.008	0.0	0.0	0.02	
3	59.904	20.970	0.120	0.7	0.4	2.88	
	Total	3042.018	33.886	100.0	100.0		

1-Phenoxy-3-(phenylsulfonyl)butan-2-one 19b

^{1}H NMR (400 MHz, $CDCl_3$)

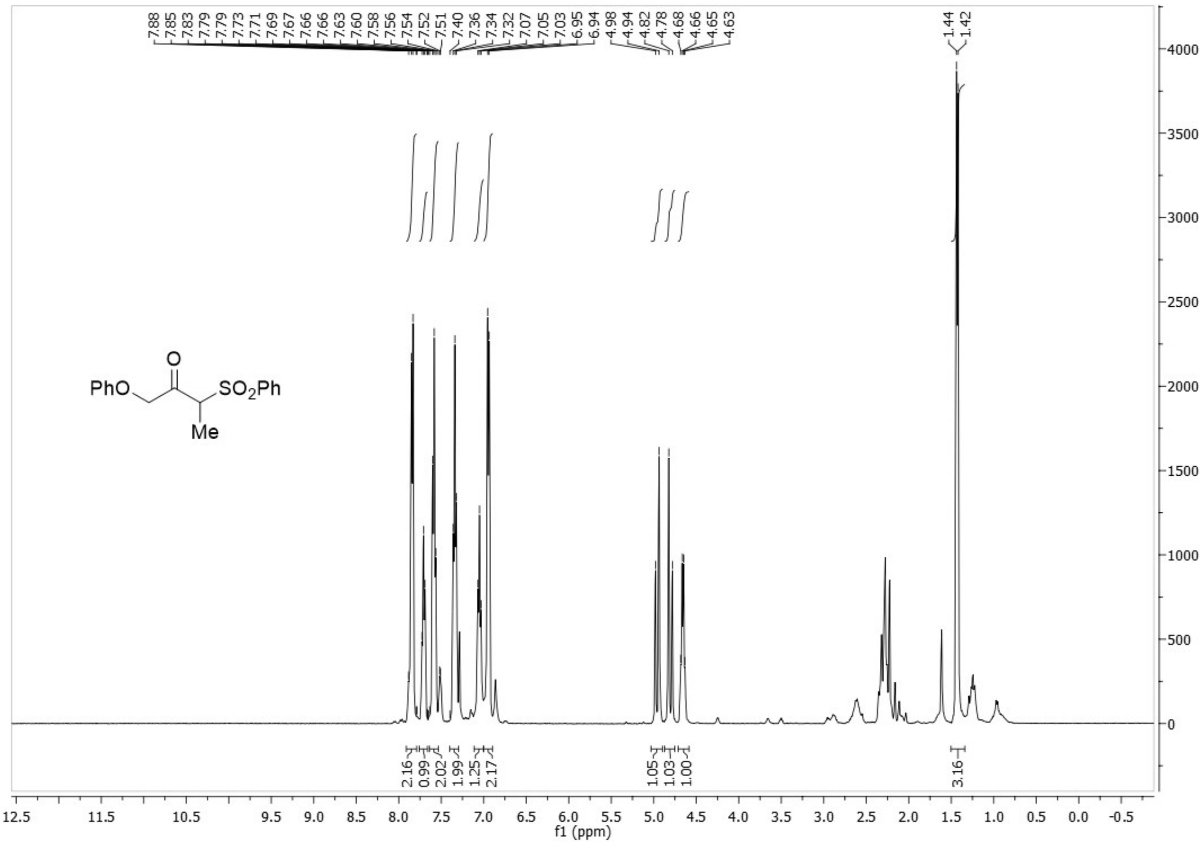

^{13}C NMR (101 MHz, $CDCl_3$

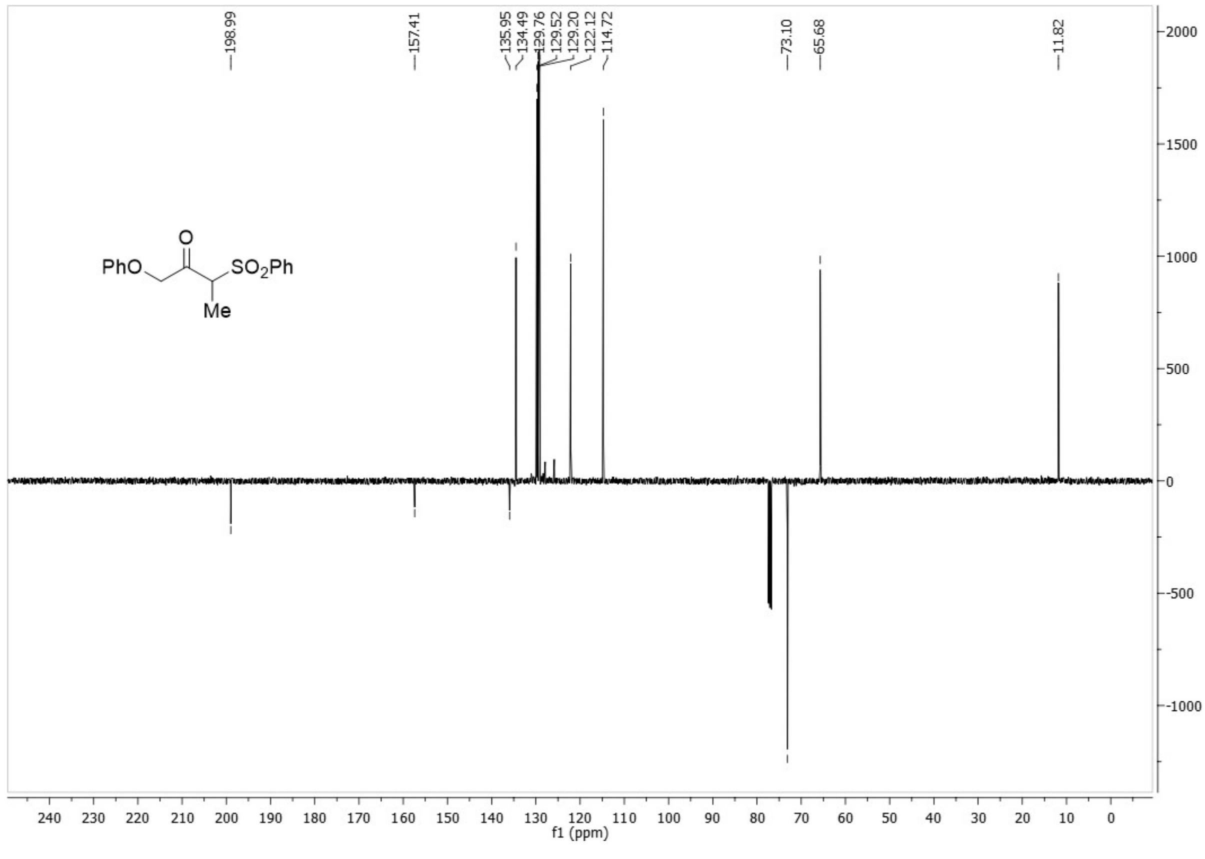

Ketone HPLC of 1-Phenoxy-3-(phenylsulfonyl)butan-2-one.

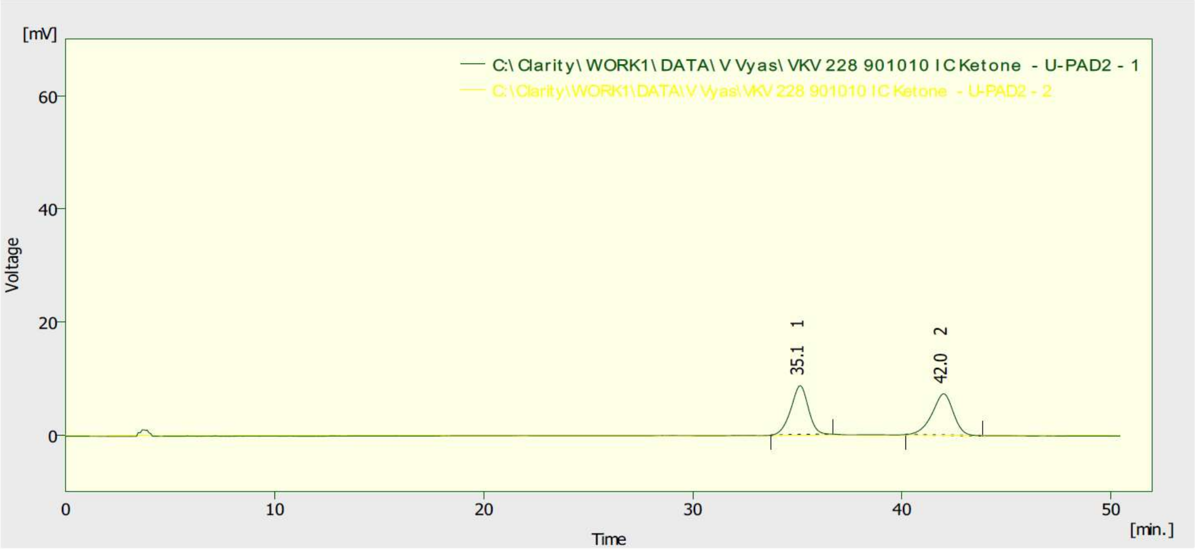

Result Table (Uncal - C:\Clarity\WORK1\DATA\V Vyas\VKV 228 901010 IC Ketone - U-PAD2 - 1)

	Reten. Time [min]	Area [mV.s]	Height [mV]	Area [%]	Height [%]	W05 [min]	Compound Name
1	35.128	505.538	8.666	49.5	54.3	0.89	
2	41.996	516.405	7.306	50.5	45.7	1.08	
	Total	1021.942	15.972	100.0	100.0		

3-Phenoxy-1-phenyl-1-(phenylsulfonyl)propan-2-ol 20c

^{1}H NMR (400 MHz, $CDCl_3$)

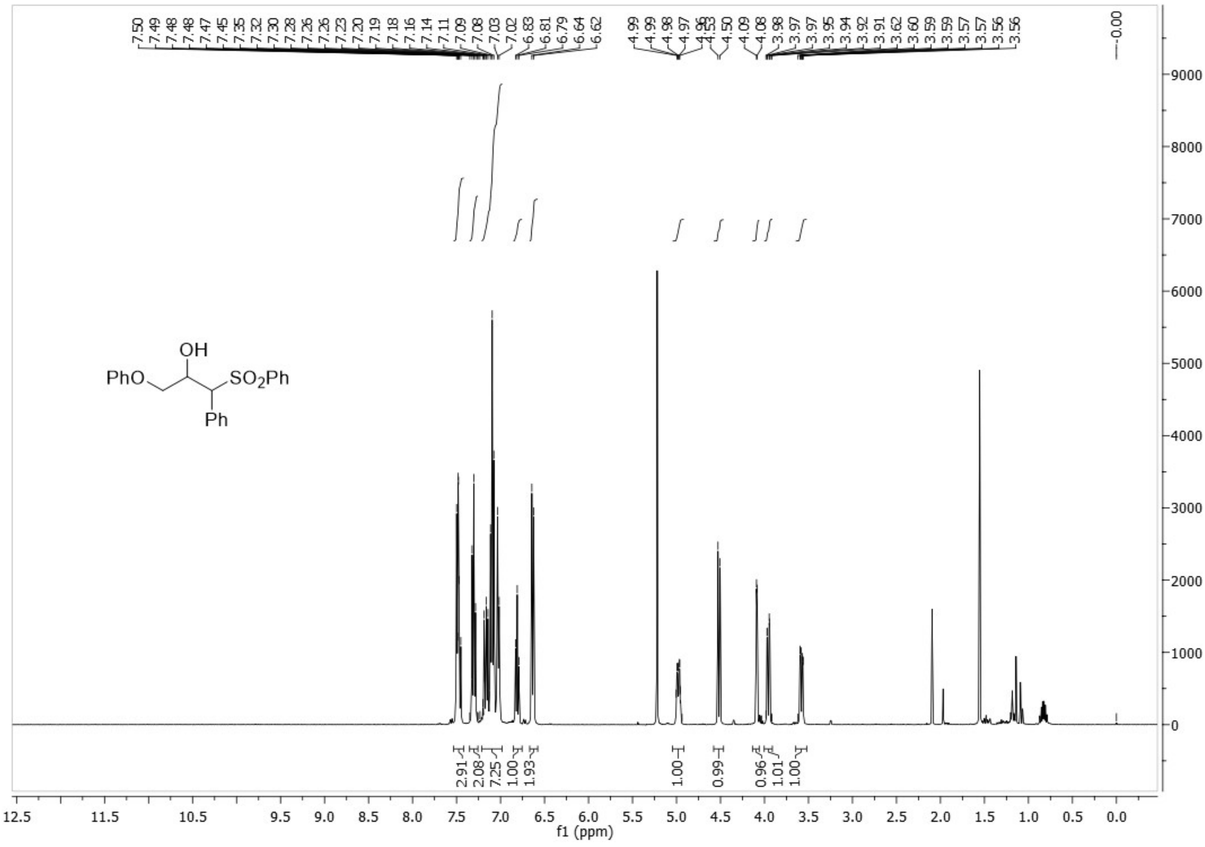

^{13}C NMR (101 MHz, $CDCl_3$

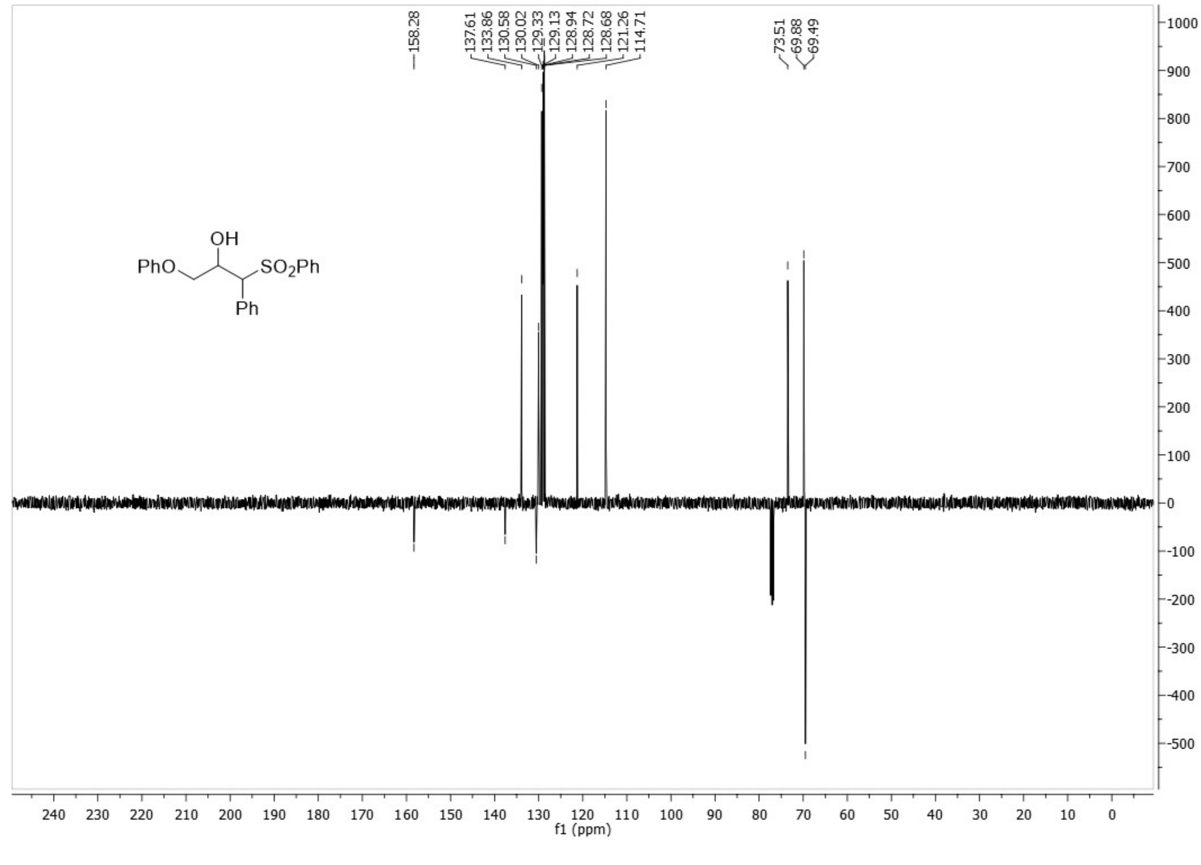

Racemic HPLC of 3-Phenoxy-1-phenyl-1-(phenylsulfonyl)propan-2-ol.

Clarity - Chromatography SW
DataApex 2006
www.dataapex.com

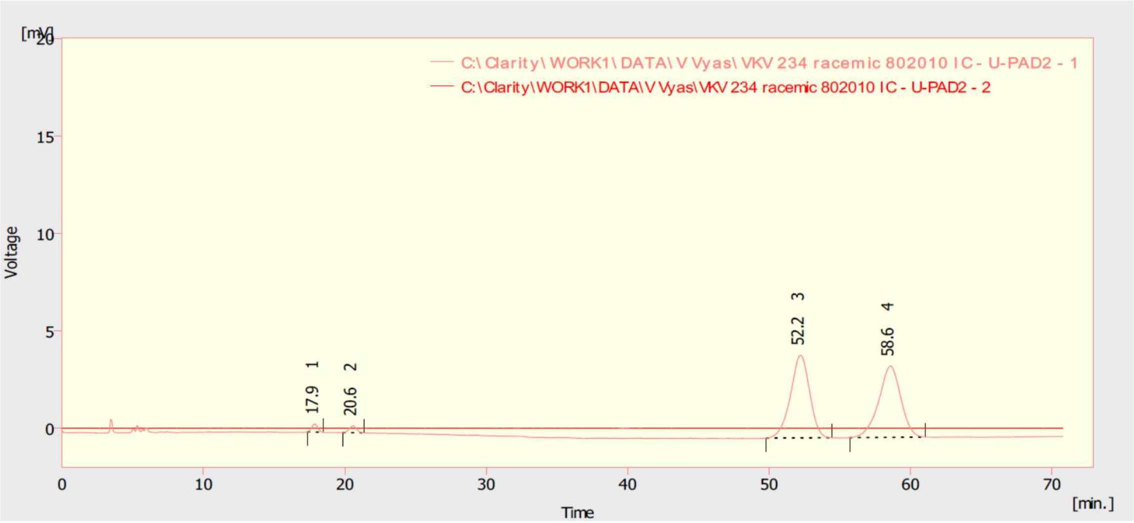

Result Table (Uncal - C:\Clarity\WORK1\DATA\V Vyas\VKV 234 racemic 802010 IC - U-PAD2 - 1)

	Reten. Time [min]	Area [mV.s]	Height [mV]	Area [%]	Height [%]	W05 [min]	Compound Name
1	17.872	12.098	0.415	1.5	4.8	0.46	
2	20.584	12.551	0.348	1.5	4.0	0.57	
3	52.220	398.355	4.237	48.5	48.9	1.44	
4	58.564	398.075	3.658	48.5	42.2	1.65	
	Total	821.079	8.659	100.0	100.0		

HPLC after ATH 3-Phenoxy-1-phenyl-1-(phenylsulfonyl)propan-2-ol. (100% conversion, 93:7 dr, 99.2% ee).

Clarity - Chromatography SW
DataApex 2006
www.dataapex.com

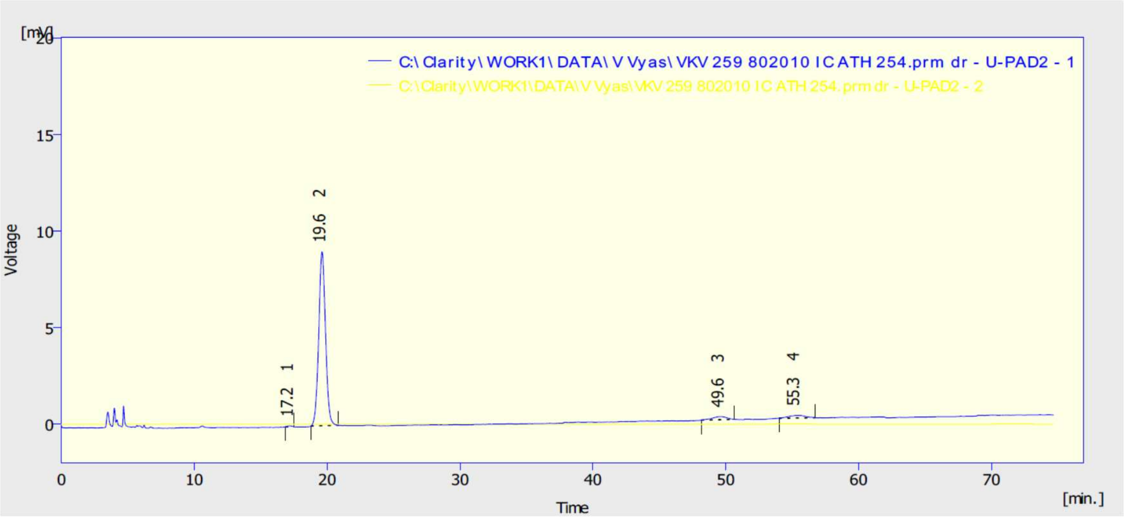

Result Table (Uncal - C:\Clarity\WORK1\DATA\V Vyas\VKV 259 802010 IC ATH 254.prm dr - U-PAD2 - 1)

	Reten. Time [min]	Area [mV.s]	Height [mV]	Area [%]	Height [%]	W05 [min]	Compound Name
1	17.176	1.145	0.052	0.3	0.6	0.40	
2	19.616	319.409	8.997	92.7	96.2	0.54	
3	49.608	12.193	0.166	3.5	1.8	1.14	
4	55.292	11.708	0.137	3.4	1.5	1.41	
	Total	344.455	9.352	100.0	100.0		

3-Phenoxy-1-phenyl-1-(phenylsulfonyl)propan-2-one 19c

^{1}H NMR (400 MHz, $CDCl_3$)

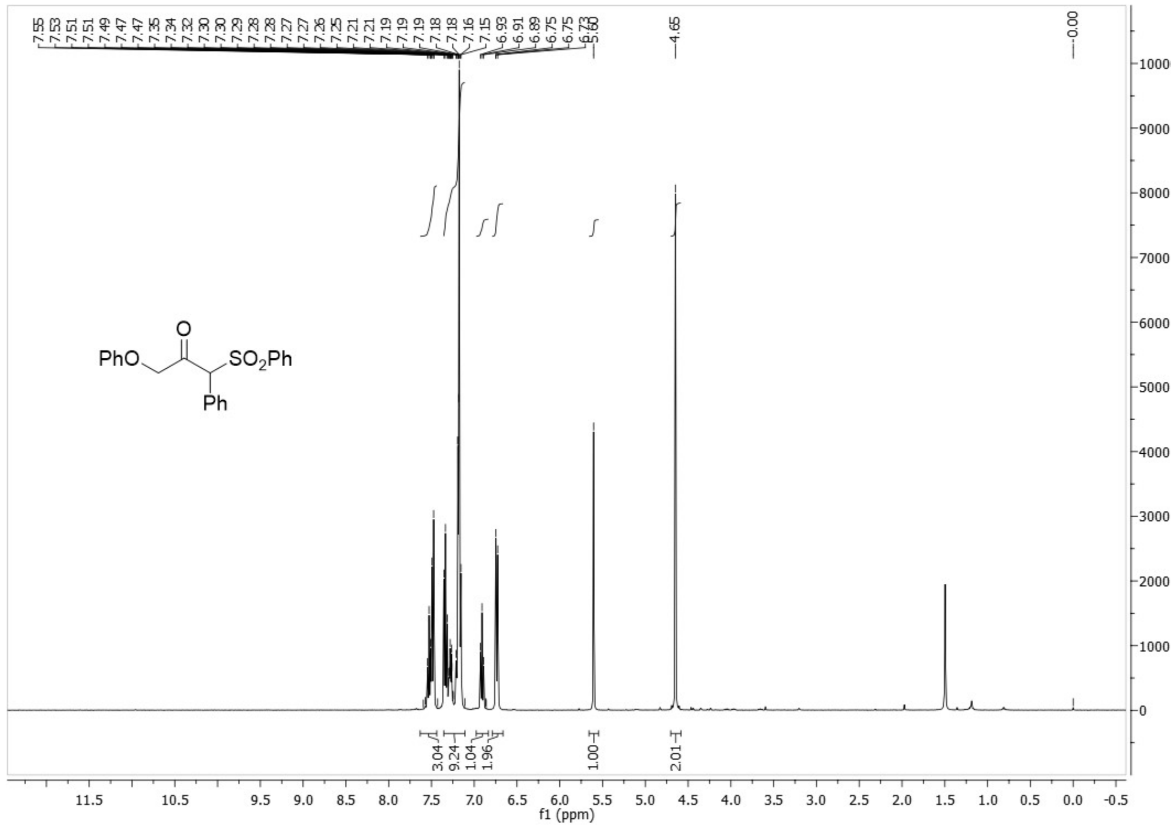

^{13}C NMR (101 MHz, $CDCl_3$

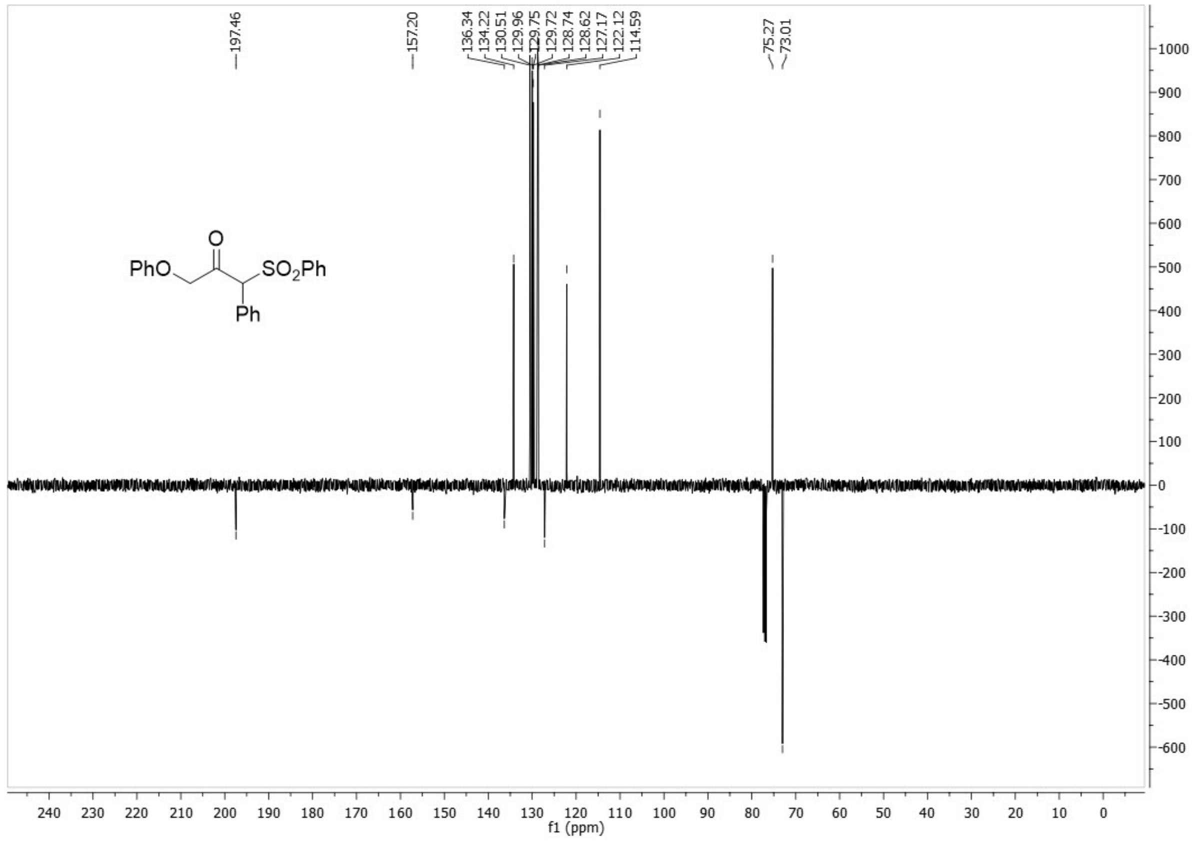

Ketone HPLC of 3-Phenoxy-1-phenyl-1-(phenylsulfonyl)propan-2-one.

Clarity - Chromatography SW
DataApex 2006
www.dataapex.com

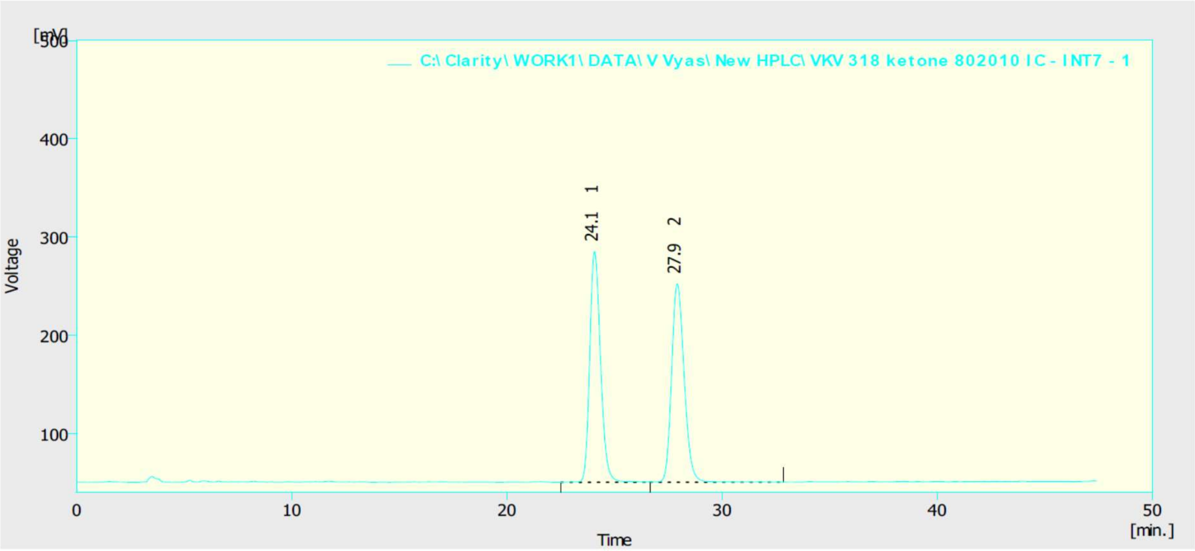

Result Table (Uncal - C:\Clarity\WORK1\DATA\V Vyas\New HPLC\VKV 318 ketone 802010 IC - INT7 - 1)

	Reten. Time [min]	Area [mV.s]	Height [mV]	Area [%]	Height [%]	W05 [min]	Compound Name
1	24.077	8219.981	234.823	49.9	53.8	0.54	
2	27.923	8259.090	202.006	50.1	46.2	0.63	
	Total	16479.071	436.829	100.0	100.0		

3-Phenoxy-1-(phenylsulfonyl)-1-(p-tolyloxy)propan-2-ol 20d

^{1}H NMR (400 MHz, $CDCl_3$)

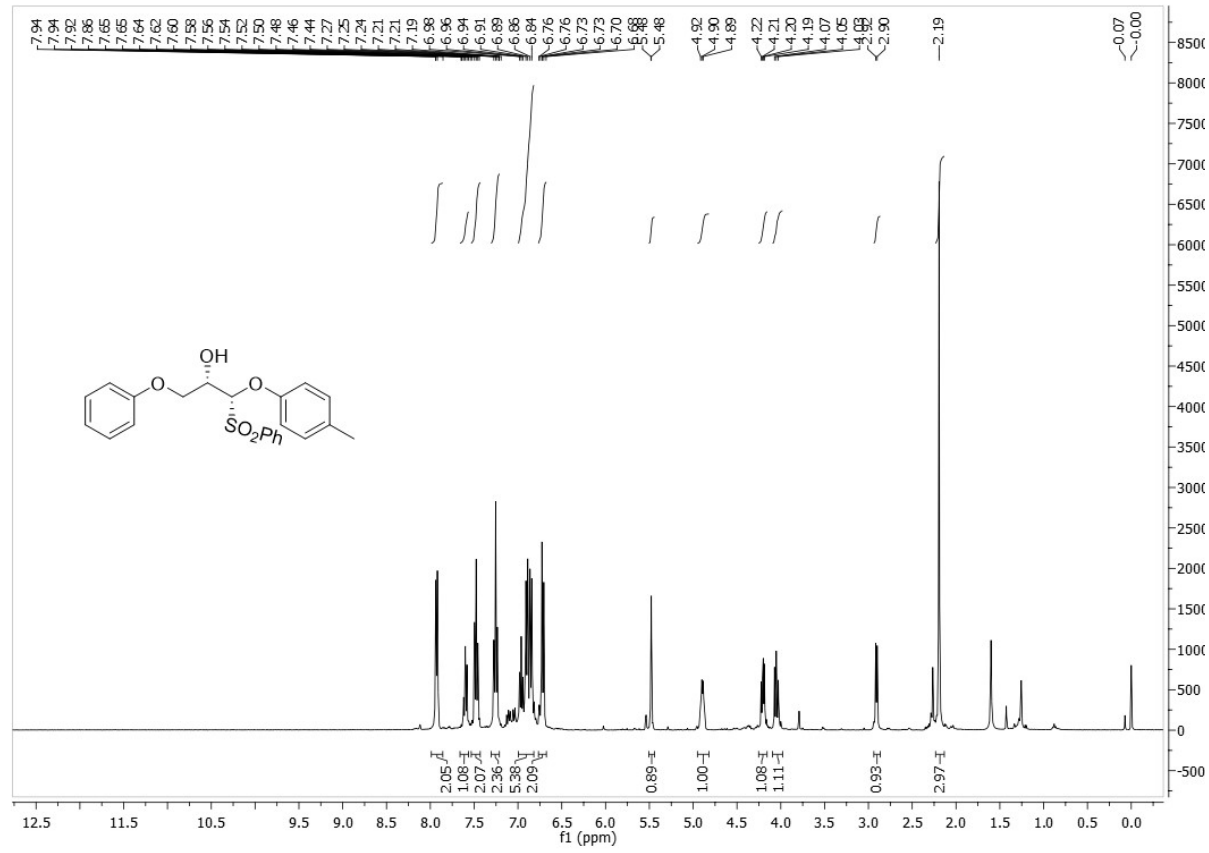

^{13}C NMR (101 MHz, $CDCl_3$)

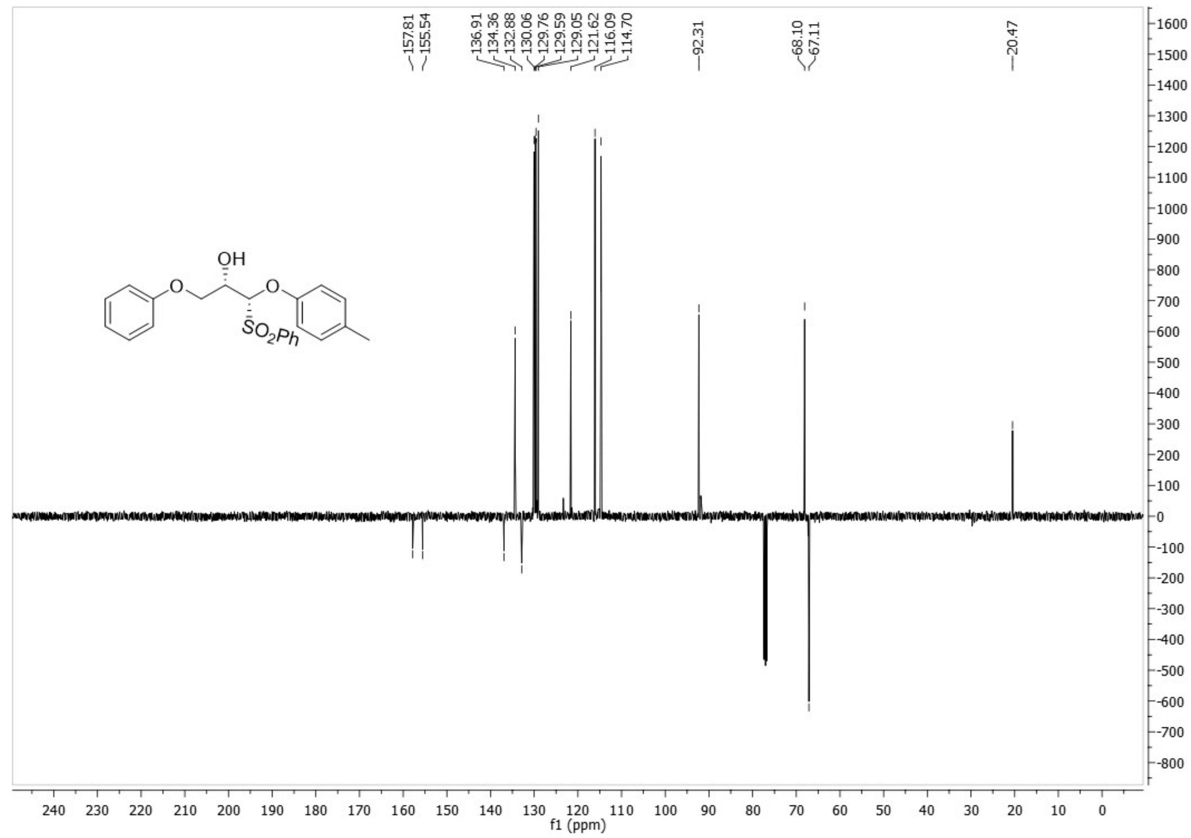

Racemic HPLC of 3-phenoxy-1-(phenylsulfonyl)-1-(p-tolyloxy)propan-2-ol.

Clarity - Chromatography SW
DataApex 2006
www.dataapex.com

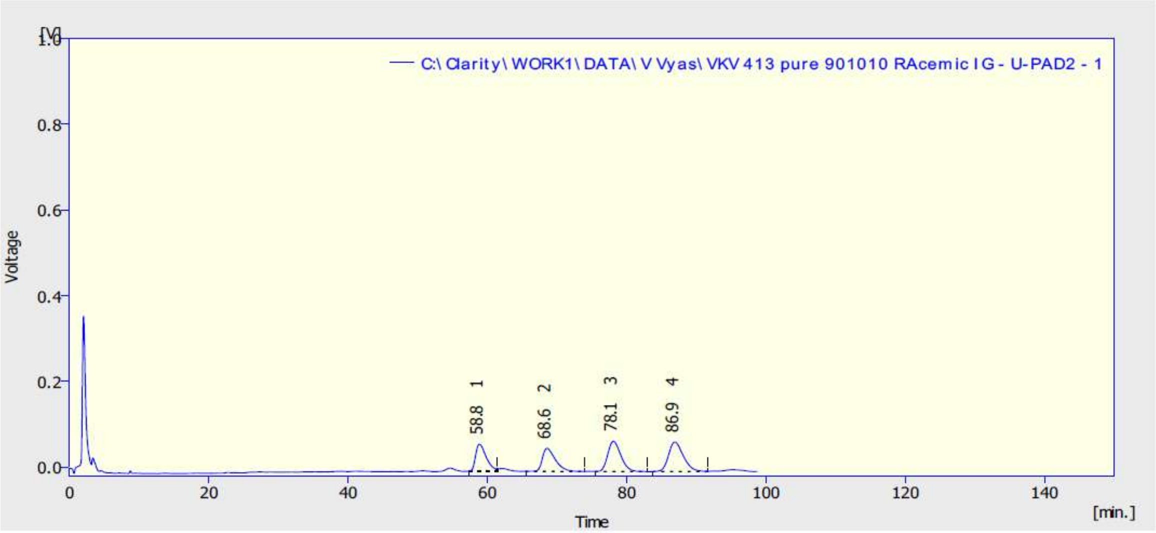

Result Table (Uncal - C:\Clarity\WORK1\DATA\V Vyas\VKV 413 pure 901010 RAcemic IG - U-PAD2 - 1)

	Reten. Time [min]	Area [mV.s]	Height [mV]	Area [%]	Height [%]	W05 [min]	Compound Name
1	58.848	6699.515	63.007	20.3	24.6	1.64	
2	68.560	6986.315	53.879	21.2	21.0	1.96	
3	78.080	9189.734	70.524	27.9	27.5	2.02	
4	86.916	10103.982	68.700	30.6	26.8	2.26	
	Total	32979.546	256.111	100.0	100.0		

HPLC after ATH 3-phenoxy-1-(phenylsulfonyl)-1-(p-tolyloxy)propan-2-ol. (100% conversion, 96:4 dr, >99.9% ee).

Clarity - Chromatography SW
DataApex 2006
www.dataapex.com

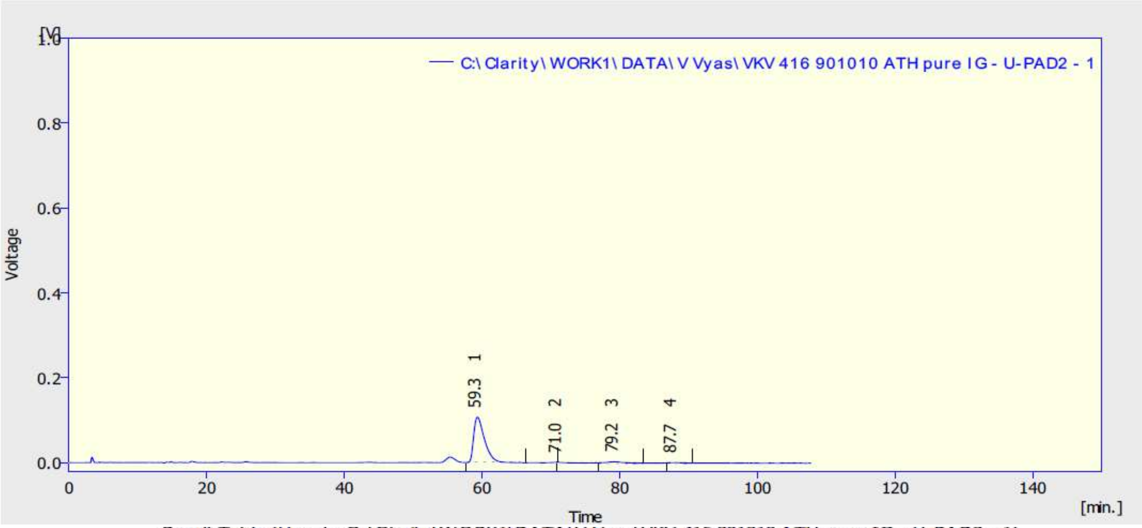

Result Table (Uncal - C:\Clarity\WORK1\DATA\V Vyas\VKV 416 901010 ATH pure IG - U-PAD2 - 1)

	Reten. Time [min]	Area [mV.s]	Height [mV]	Area [%]	Height [%]	W05 [min]	Compound Name
1	59.324	11904.006	107.408	95.9	96.8	1.66	
2	71.016	0.340	0.044	0.0	0.0	0.06	
3	79.200	400.317	2.732	3.2	2.5	2.02	
4	87.688	105.187	0.740	0.8	0.7	1.92	
	Total	12409.851	110.924	100.0	100.0		

3-Phenoxy-1-(phenylsulfonyl)-1-(p-tolyloxy)propan-2-one 19d

^{1}H NMR (400 MHz, $CDCl_3$)

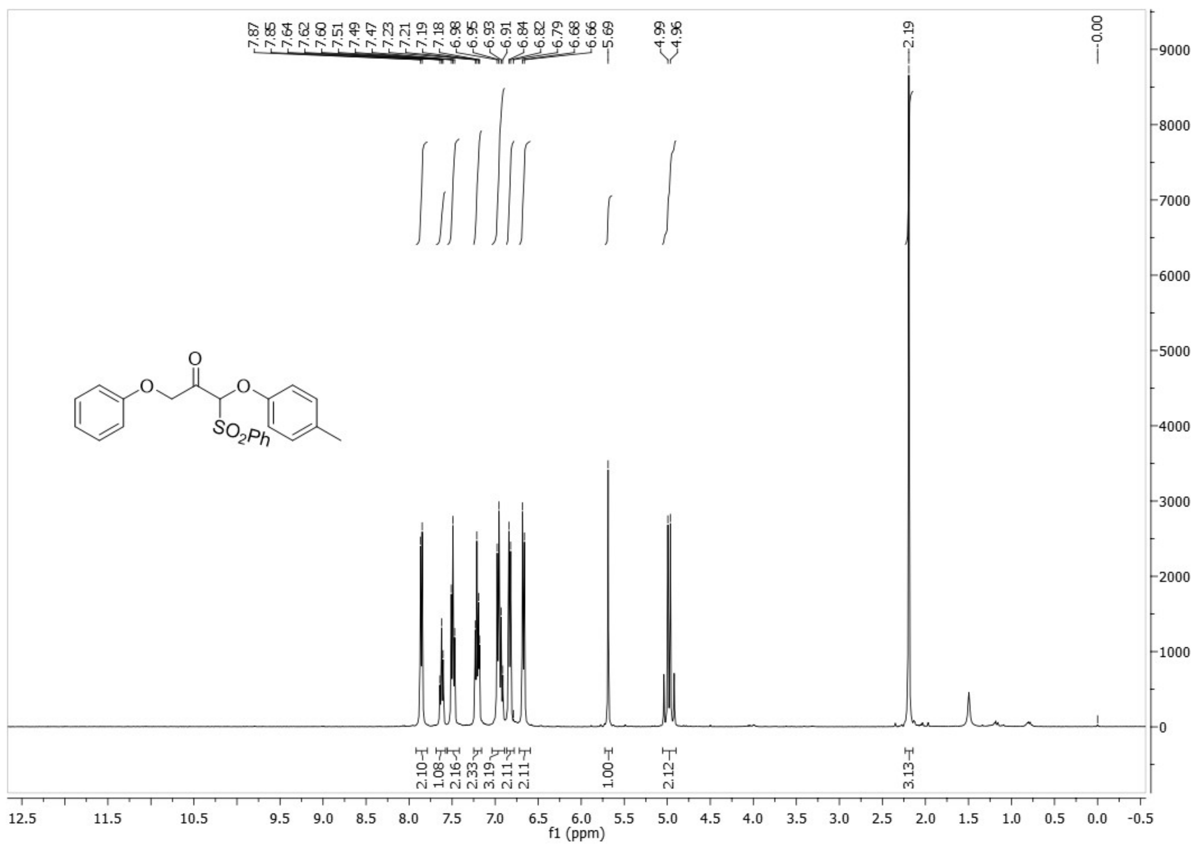

^{13}C NMR (101 MHz, $CDCl_3$)

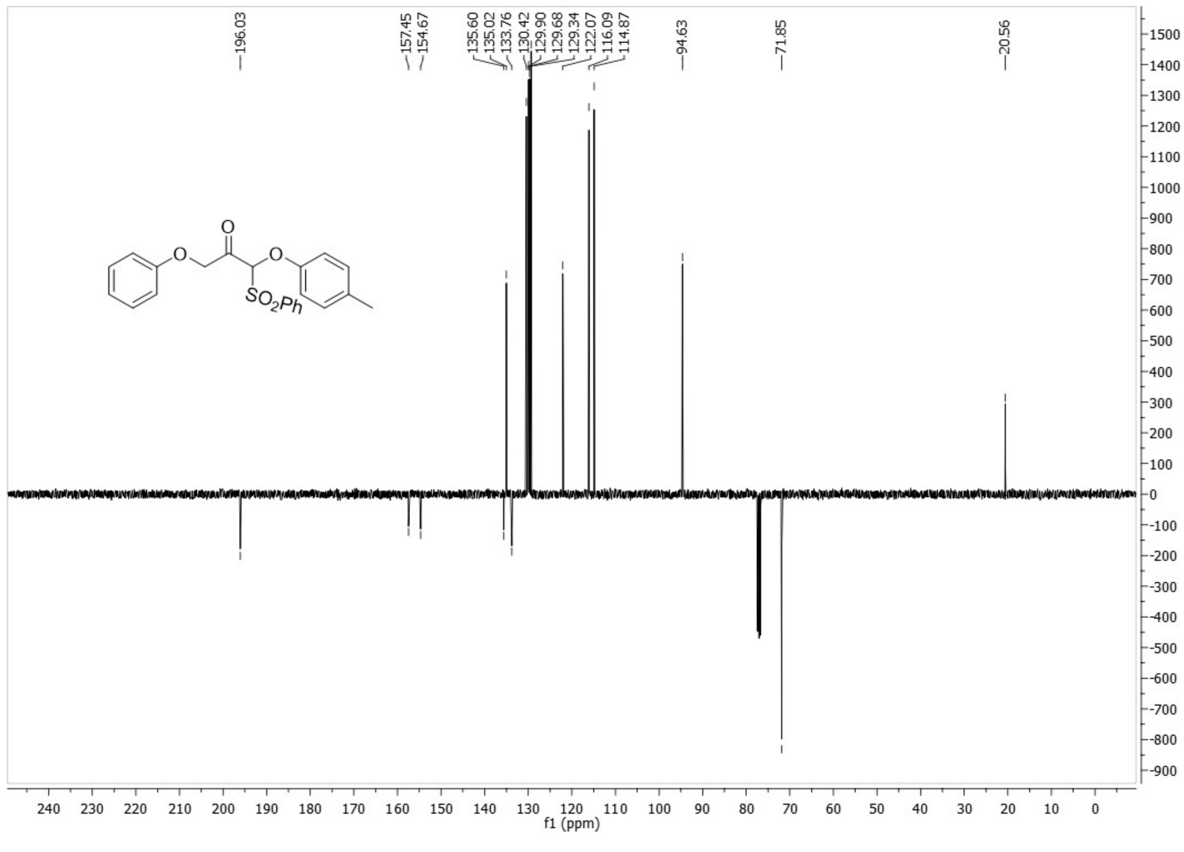

Ketone HPLC of 3-Phenoxy-1-(phenylsulfonyl)-1-(p-tolyloxy)propan-2-one.

Clarity - Chromatography SW
DataApex 2006
www.dataapex.com

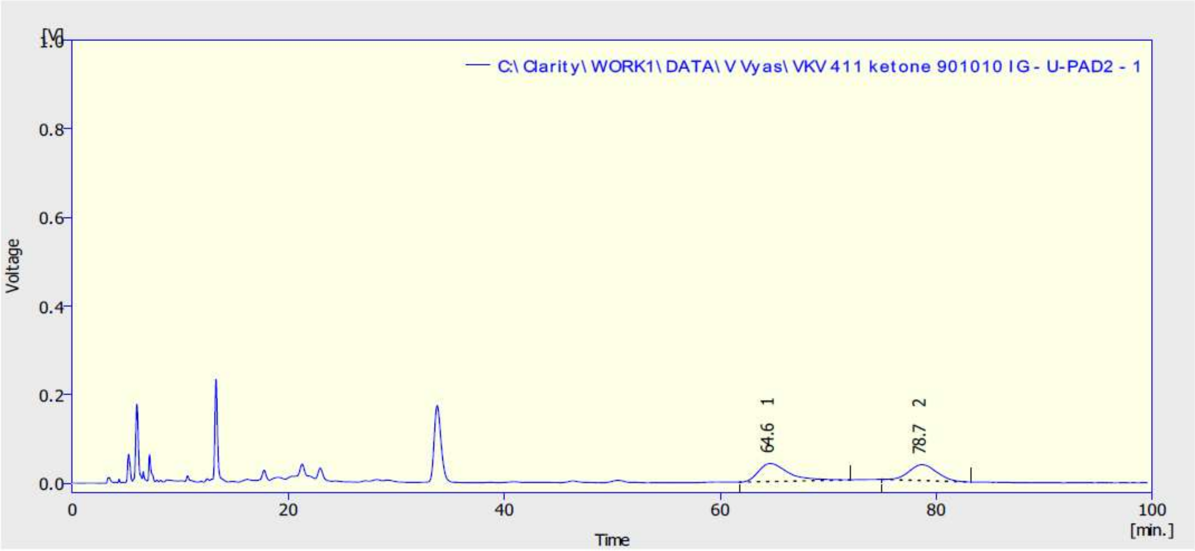

Result Table (Uncal - C:\Clarity\WORK1\DATA\V Vyas\VKV 411 ketone 901010 IG - U-PAD2 - 1)

	Reten. Time [min]	Area [mV.s]	Height [mV]	Area [%]	Height [%]	W05 [min]	Compound Name
1	64.640	7959.025	40.756	55.3	53.1	2.86	
2	78.704	6421.992	35.945	44.7	46.9	2.78	
	Total	14381.017	76.700	100.0	100.0		

1-(Benzyloxy)-3-phenoxy-1-(phenylsulfonyl)propan-2-ol 20e

^{1}H NMR (400 MHz, $CDCl_3$)

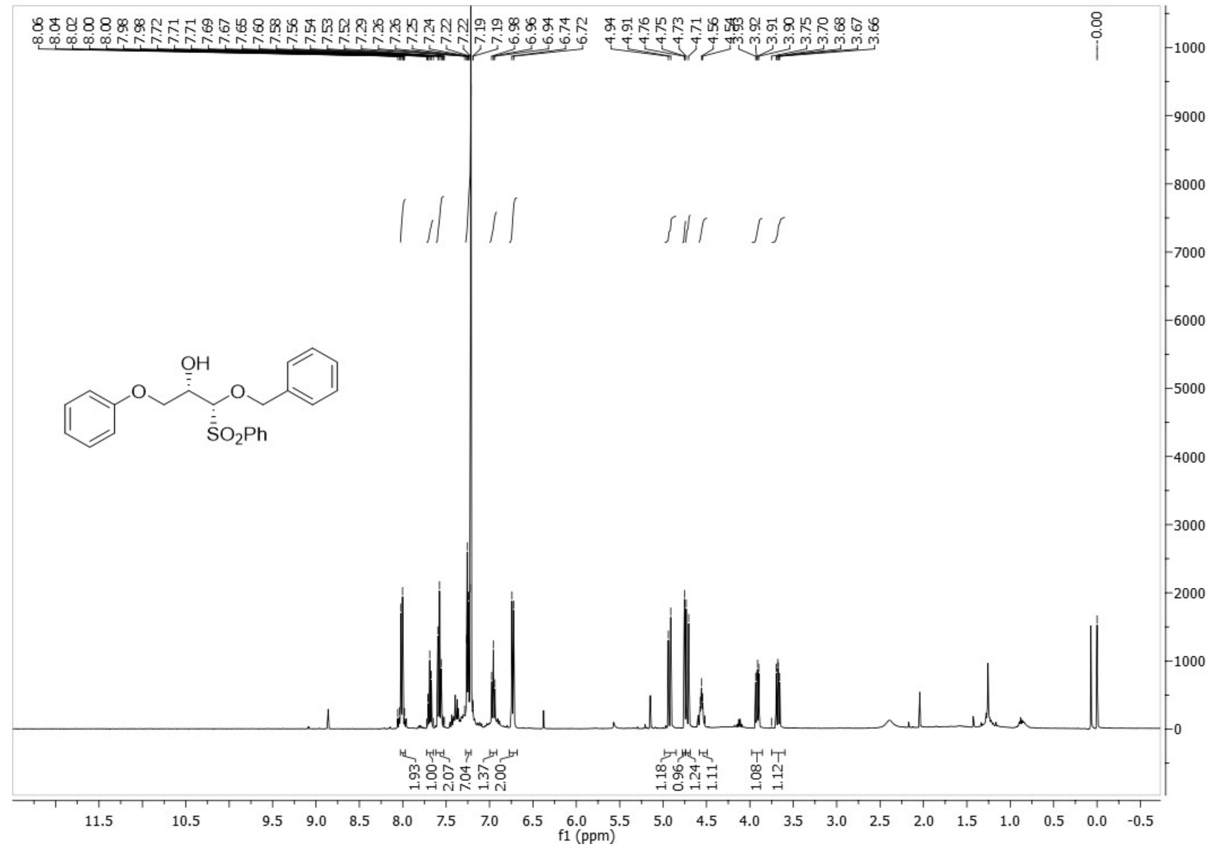

^{13}C NMR (101 MHz, $CDCl_3$)

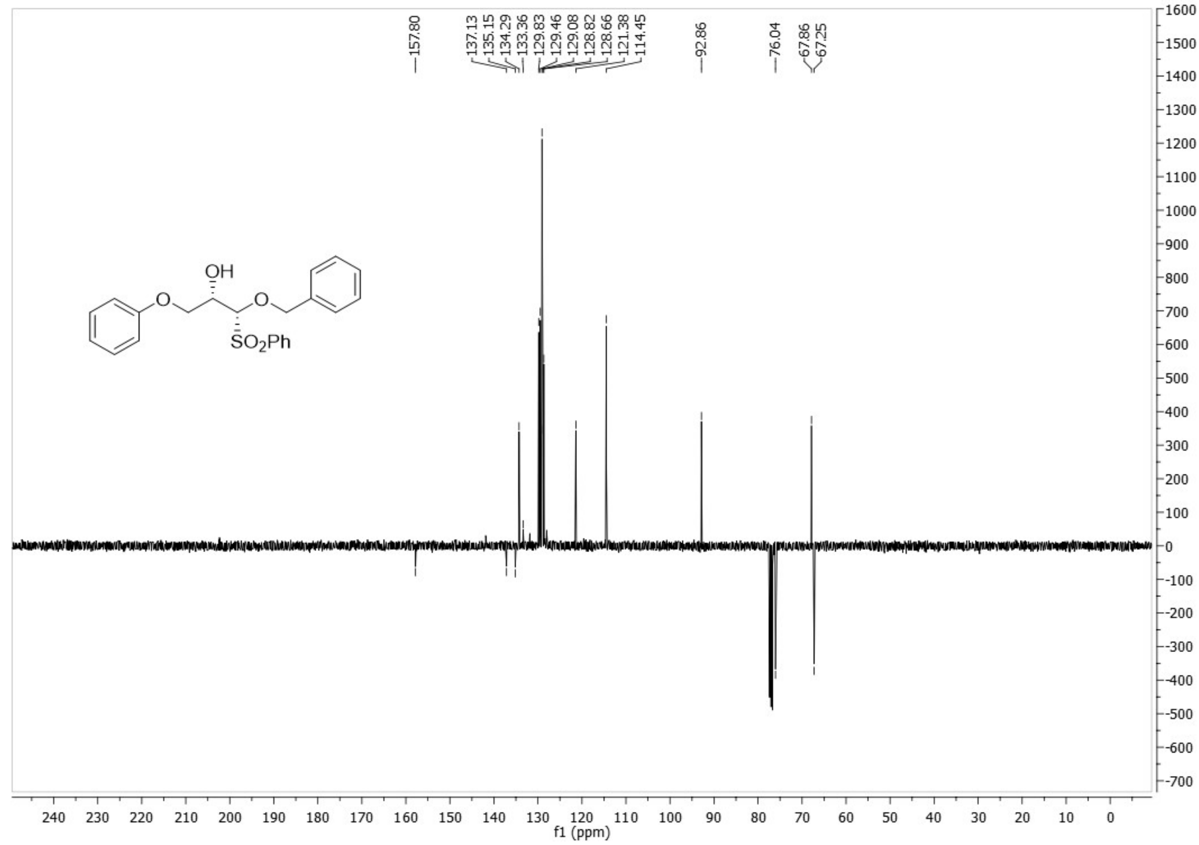

Racemic HPLC of 1-(Benzyloxy)-3-phenoxy-1-(phenylsulfonyl)propan-2-ol.

Clarity - Chromatography SW
DataApex 2006
www.dataapex.com

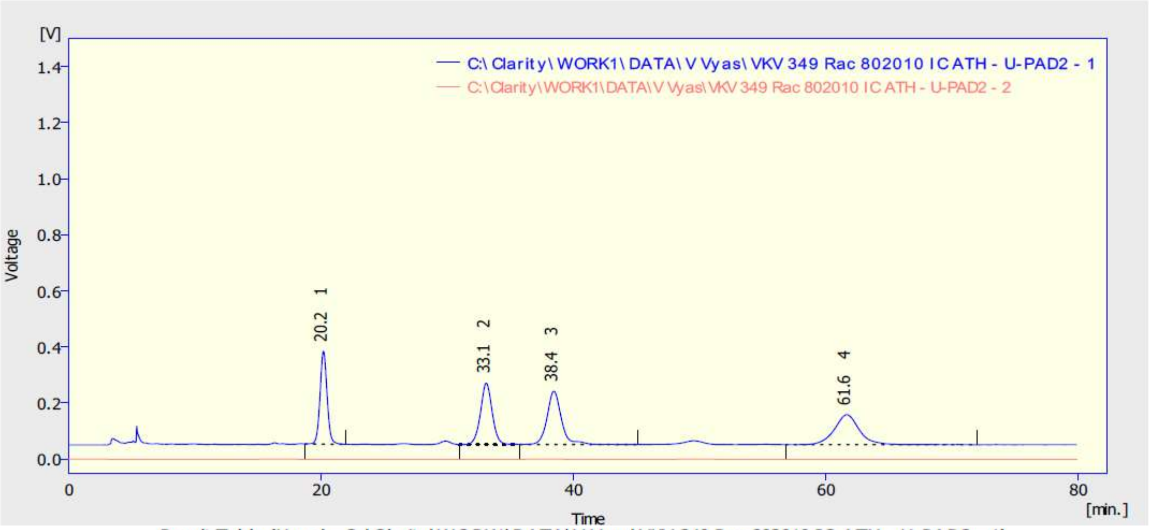

Result Table (Uncal - C:\Clarity\WORK1\DATA\V Vyas\VKV 349 Rac 802010 IC ATH - U-PAD2 - 1)

	Reten. Time [min]	Area [mV.s]	Height [mV]	Area [%]	Height [%]	W05 [min]	Compound Name
1	20.156	12876.087	331.130	22.6	39.1	0.59	
2	33.060	14140.971	218.983	24.8	25.8	0.97	
3	38.420	15141.811	190.412	26.6	22.5	1.13	
4	61.644	14856.886	107.001	26.1	12.6	2.00	
	Total	57015.755	847.525	100.0	100.0		

HPLC after ATH 1-(Benzyloxy)-3-phenoxy-1-(phenylsulfonyl)propan-2-ol. (100% conversion, 99.7:0.3 dr, 99.6% ee).

Clarity - Chromatography SW
DataApex 2006
www.dataapex.com

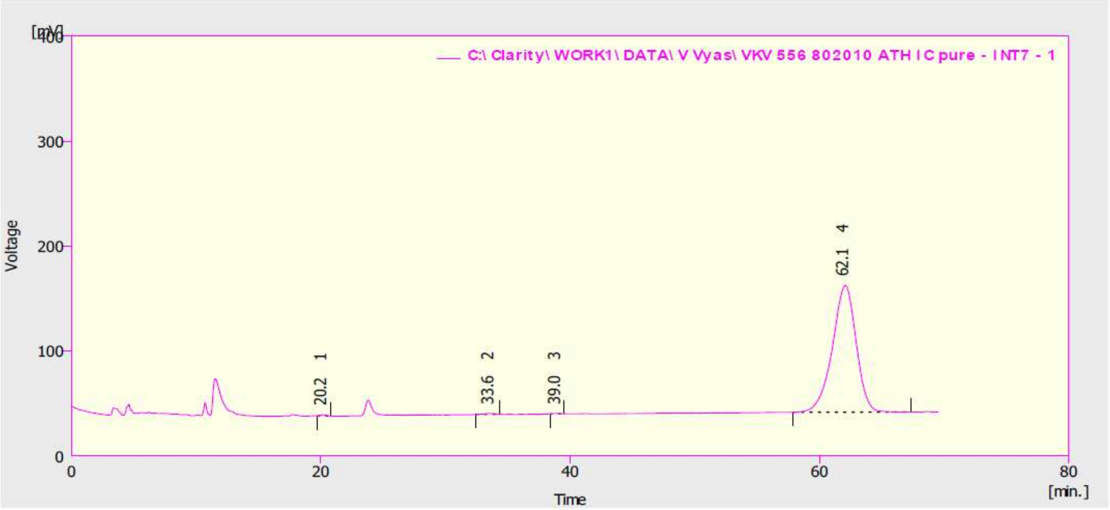

Result Table (Uncal - C:\Clarity\WORK1\DATA\V Vyas\VKV 556 802010 ATH IC pure - INT7 - 1)

	Reten. Time [min]	Area [mV.s]	Height [mV]	Area [%]	Height [%]	W05 [min]	Compound Name
1	20.197	30.980	0.936	0.2	0.8	0.53	
2	33.617	45.183	0.806	0.3	0.7	0.85	
3	38.950	7.345	0.220	0.0	0.2	0.58	
4	62.073	16029.626	120.861	99.5	98.4	2.01	
	Total	16113.135	122.822	100.0	100.0		

1-(Benzyloxy)-3-phenoxy-1-(phenylsulfonyl)propan-2-one 19e

^{1}H NMR (400 MHz, $CDCl_3$)

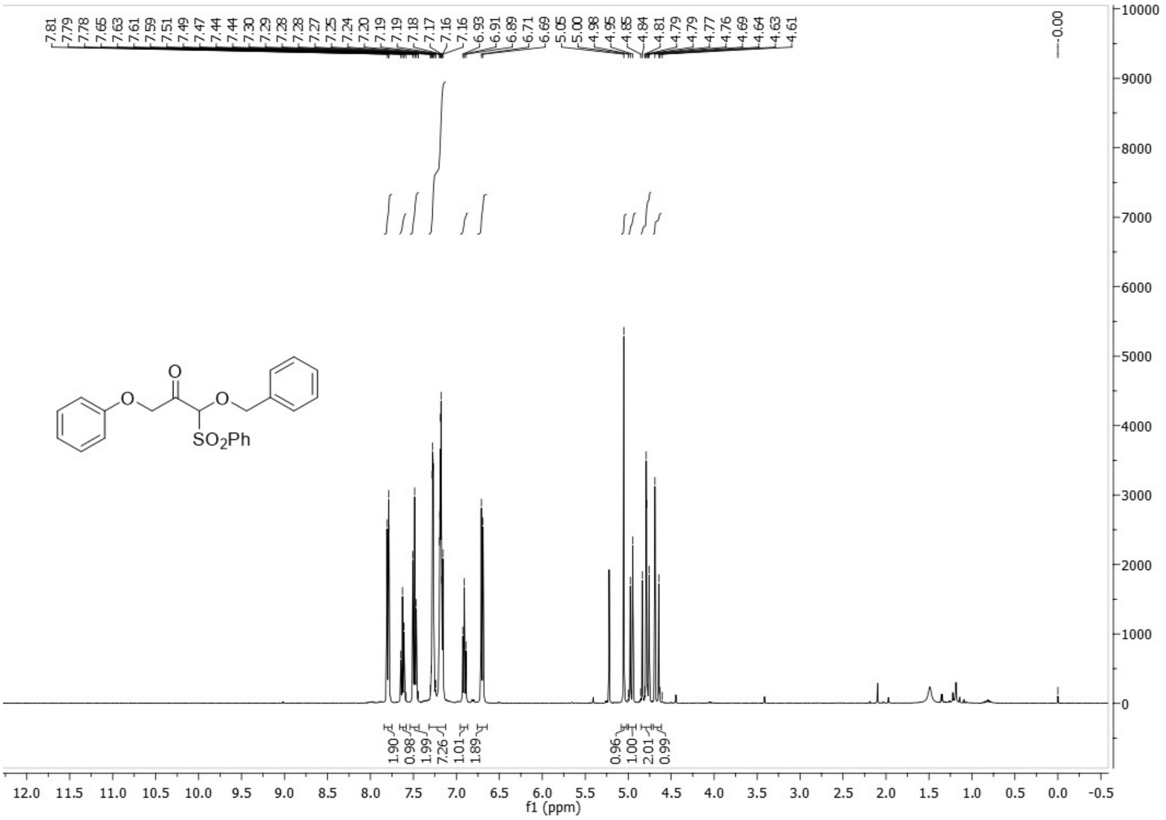

^{13}C NMR (101 MHz, $CDCl_3$)

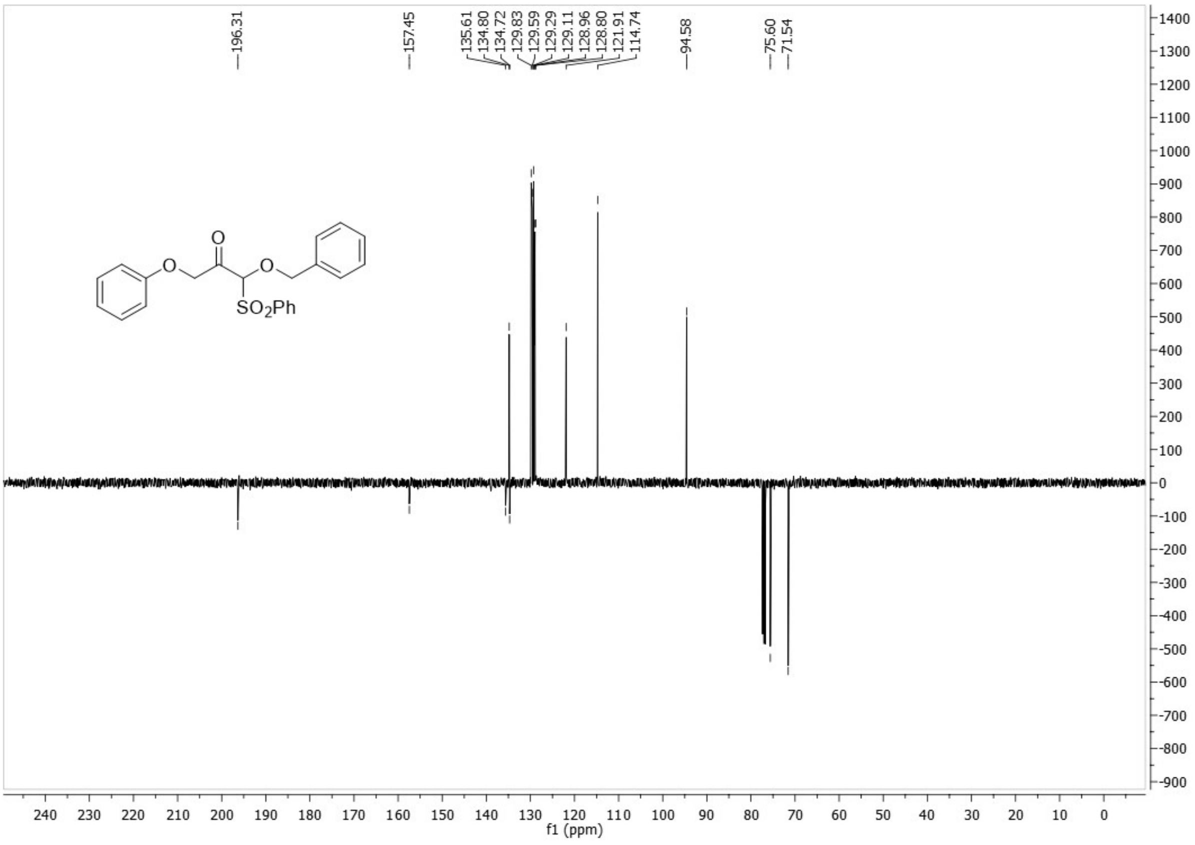

Ketone HPLC of 1-(Benzyloxy)-3-phenoxy-1-(phenylsulfonyl)propan-2-one.

Clarity - Chromatography SW

DataApex 2006

www.dataapex.com

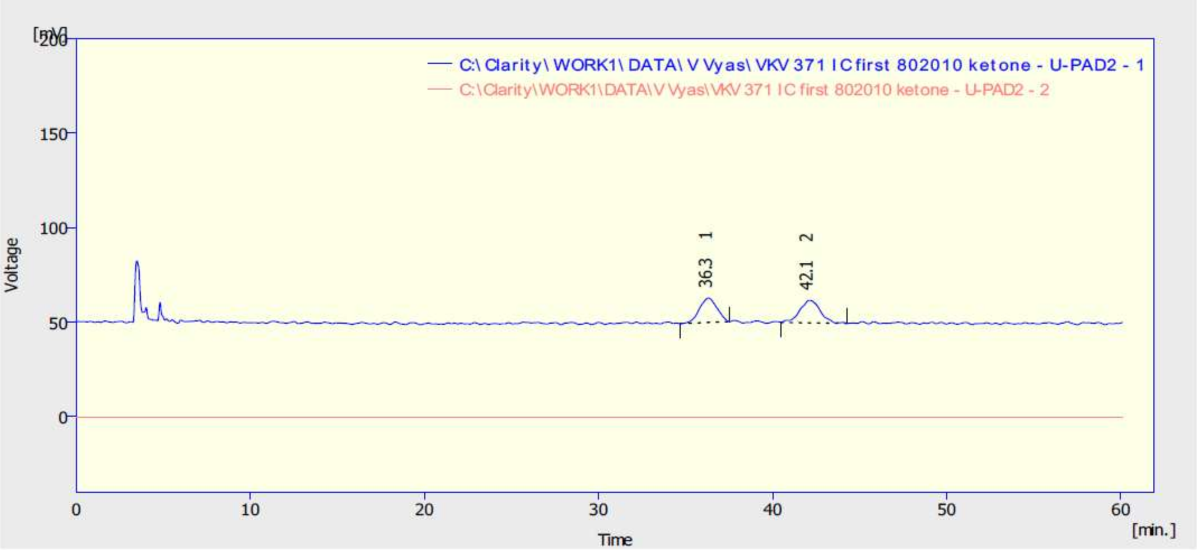

Result Table (Uncal - C:\Clarity\WORK1\DATA\V Vyas\VKV 371 IC first 802010 ketone - U-PAD2 - 1)

	Reten. Time [min]	Area [mV.s]	Height [mV]	Area [%]	Height [%]	W05 [min]	Compound Name
1	36.320	907.391	12.915	48.3	52.1	1.16	
2	42.116	972.198	11.868	51.7	47.9	1.26	
	Total	1879.589	24.783	100.0	100.0		

1-Methoxy-3-phenoxy-1-(phenylsulfonyl)propan-2-ol 20f

^{1}H NMR (400 MHz, $CDCl_3$)

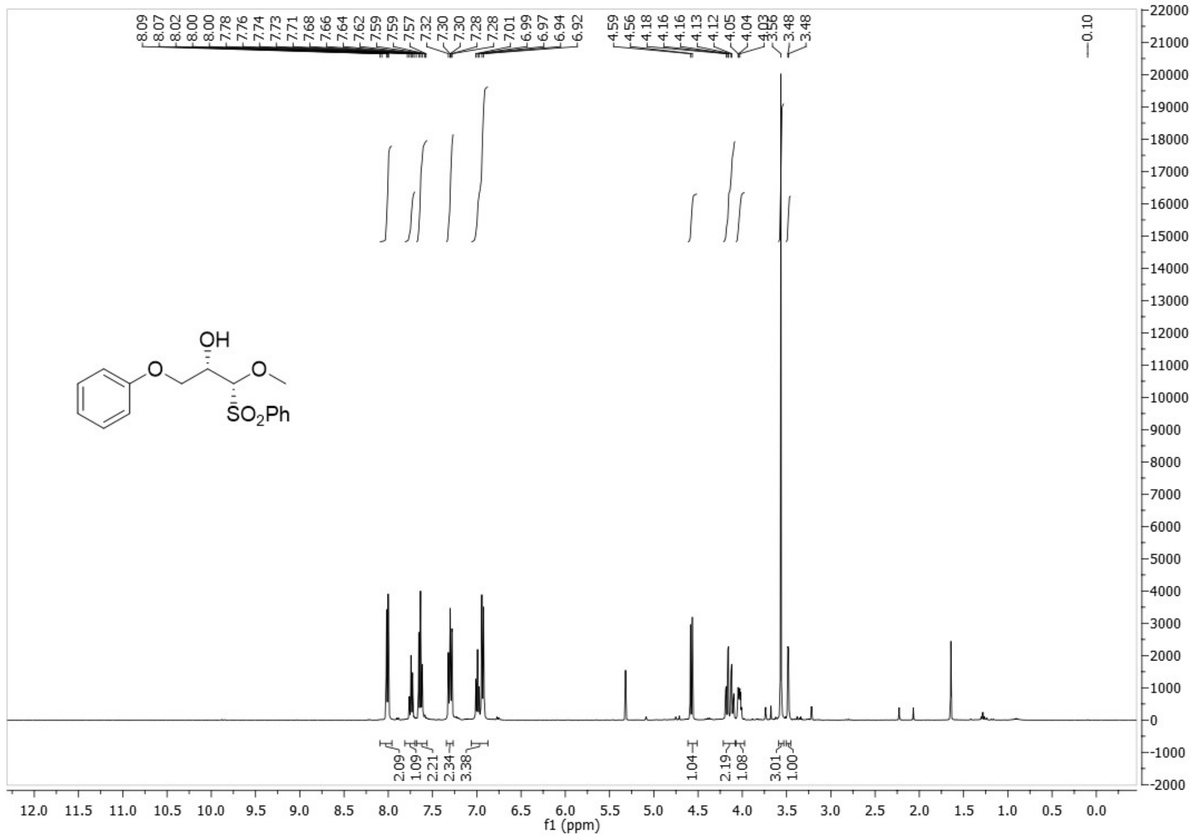

^{13}C NMR (101 MHz, $CDCl_3$)

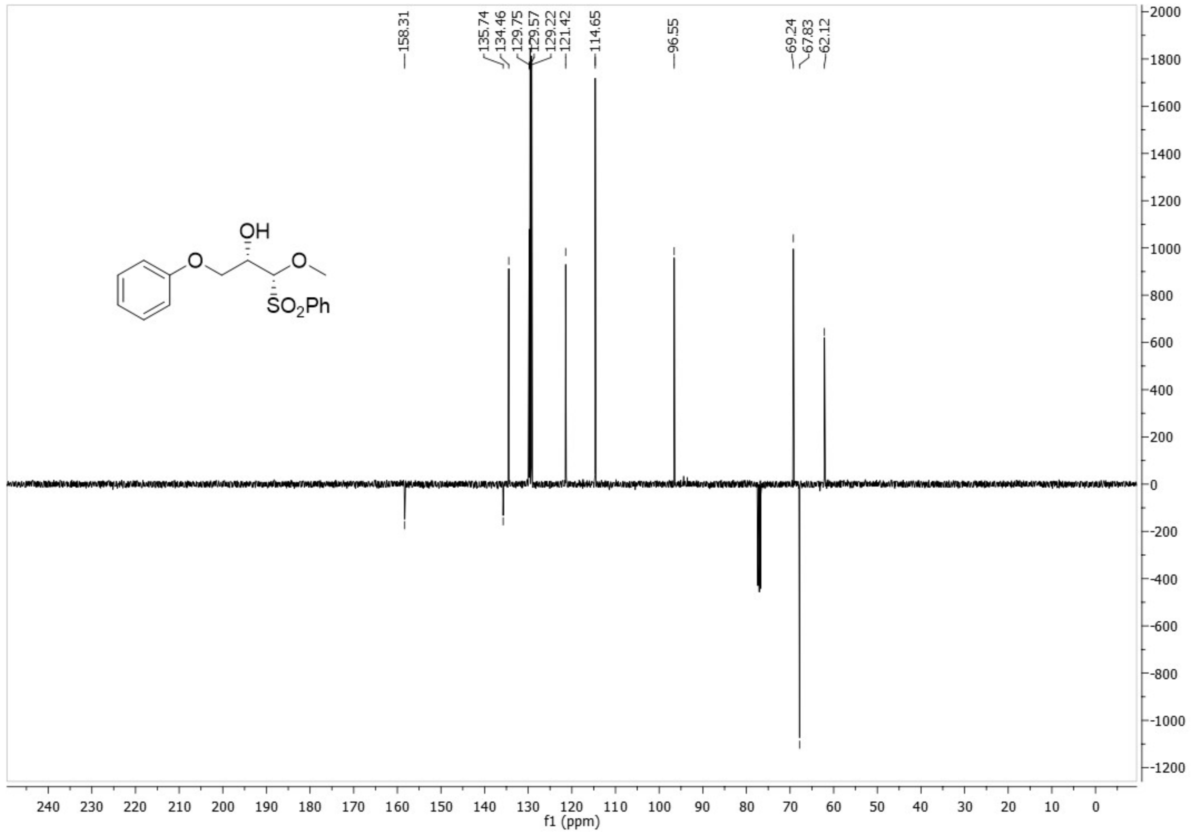

Racemic HPLC of 1-Methoxy-3-phenoxy-1-(phenylsulfonyl)propan-2-ol.

Clarity - Chromatography SW
DataApex 2006
www.dataapex.com

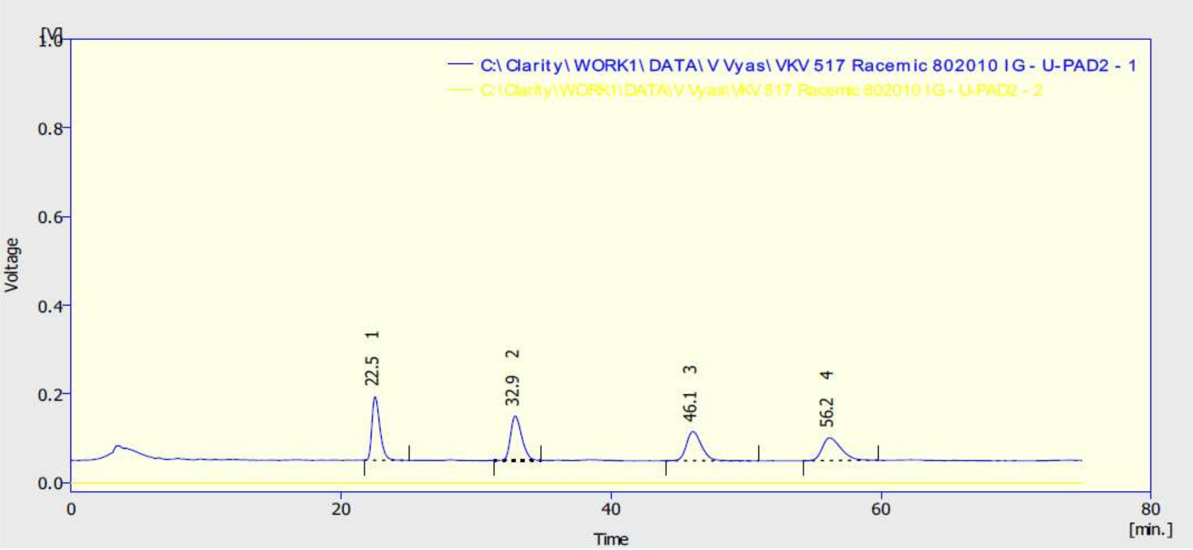

Result Table (Uncal - C:\Clarity\WORK1\DATA\V Vyas\VKV 517 Racemic 802010 IG - U-PAD2 - 1)

	Reten. Time [min]	Area [mV.s]	Height [mV]	Area [%]	Height [%]	W05 [min]	Compound Name
1	22.512	5890.437	142.907	27.1	39.7	0.63	
2	32.896	5821.234	100.332	26.8	27.9	0.90	
3	46.056	5088.854	65.574	23.4	18.2	1.18	
4	56.184	4951.893	51.263	22.8	14.2	1.49	
	Total	21752.418	360.076	100.0	100.0		

HPLC after ATH 1-Methoxy-3-phenoxy-1-(phenylsulfonyl)propan-2-ol. (100% conversion, >99:1 dr, 94.4% ee).

Clarity - Chromatography SW
DataApex 2006
www.dataapex.com

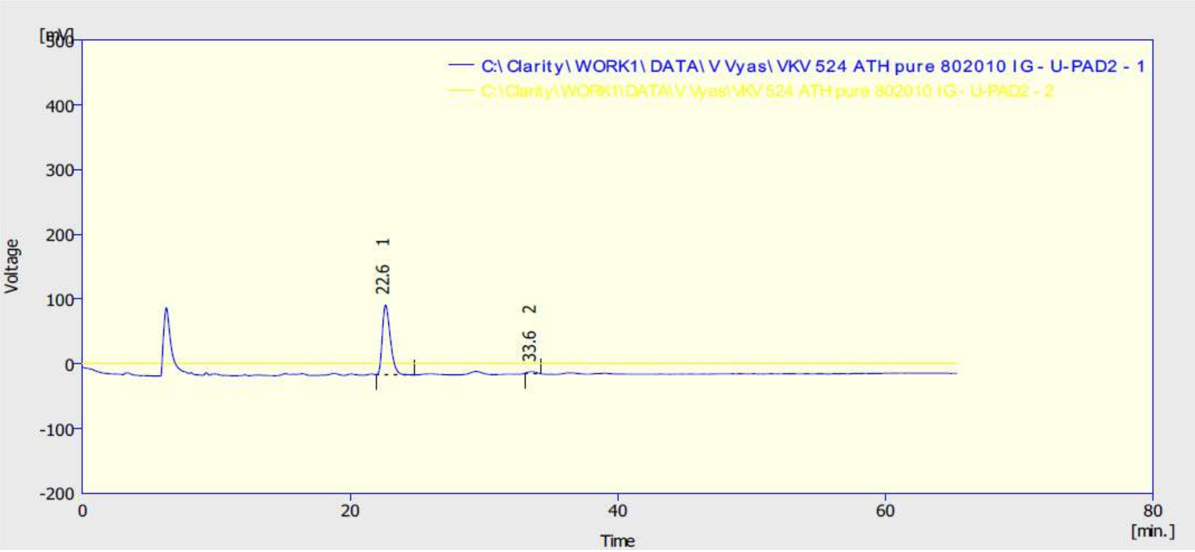

Result Table (Uncal - C:\Clarity\WORK1\DATA\V Vyas\VKV 524 ATH pure 802010 IG - U-PAD2 - 1)

	Reten. Time [min]	Area [mV.s]	Height [mV]	Area [%]	Height [%]	W05 [min]	Compound Name
1	22.644	4453.969	107.698	97.2	97.3	0.64	
2	33.604	126.058	2.974	2.8	2.7	0.72	
	Total	4580.027	110.672	100.0	100.0		

1-Methoxy-3-phenoxy-1-(phenylsulfonyl)propan-2-one 19f

^{1}H NMR (400 MHz, $CDCl_3$)

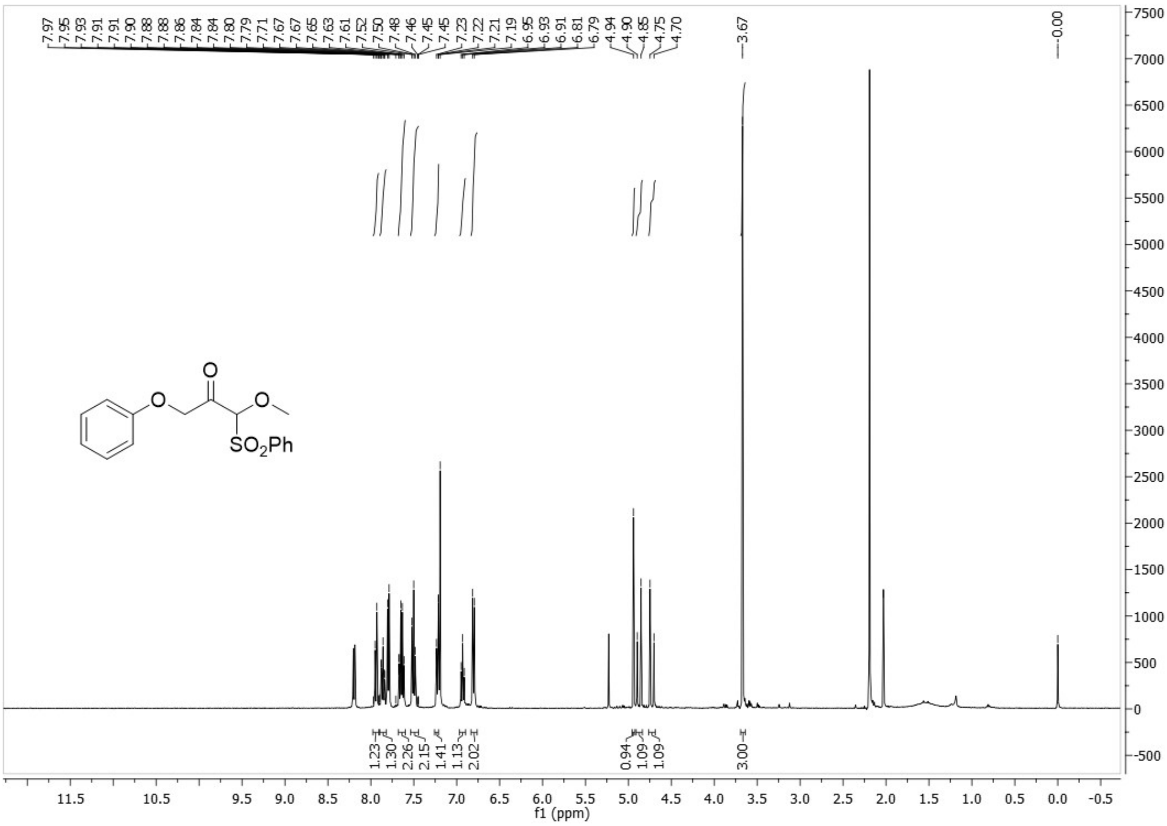

^{13}C NMR (101 MHz, $CDCl_3$)

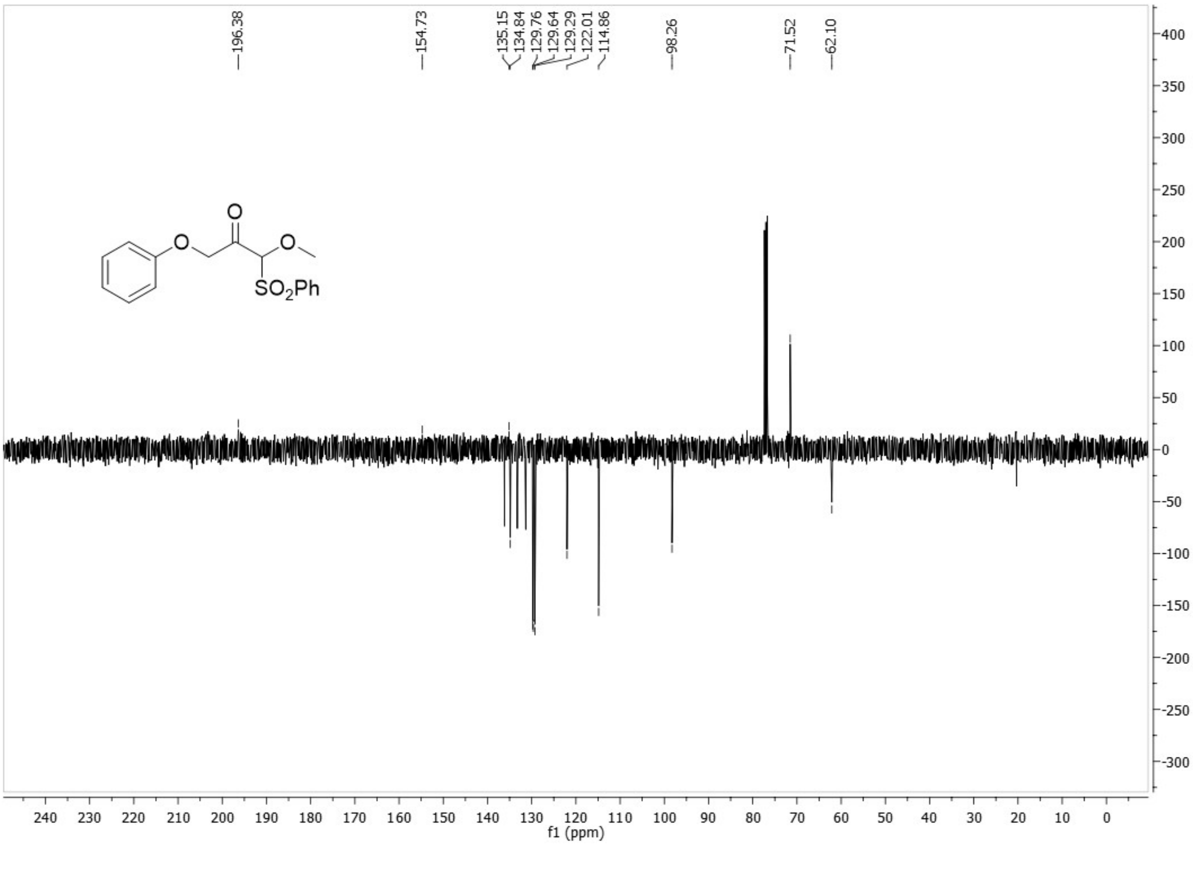

Ketone HPLC of 1-Methoxy-3-phenoxy-1-(phenylsulfonyl)propan-2-one.

Clarity - Chromatography SW
DataApex 2006
www.dataapex.com

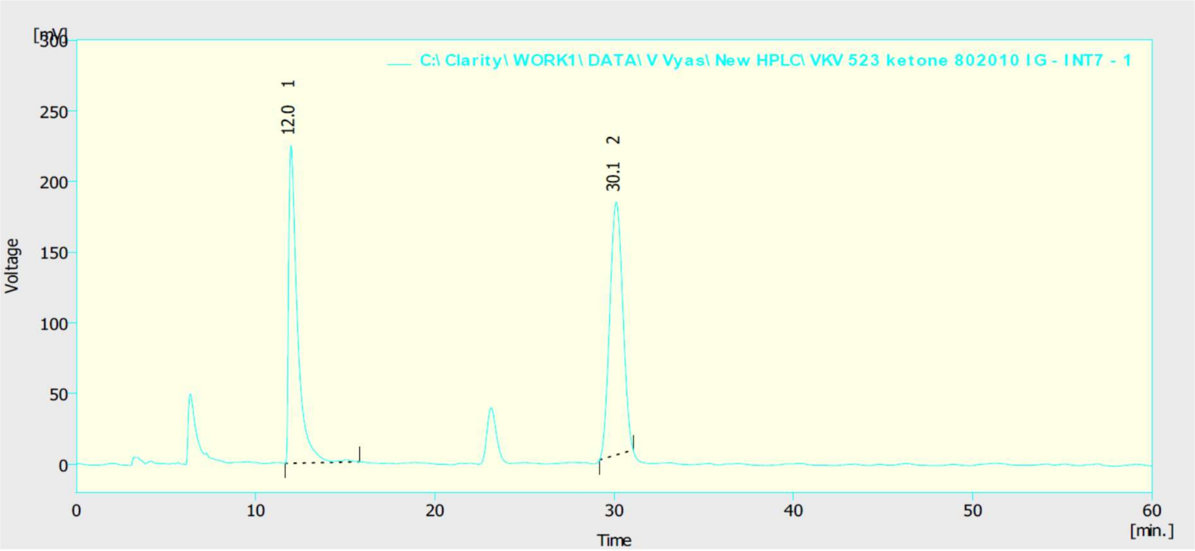

Result Table (Uncal - C:\Clarity\WORK1\DATA\V Vyas\New HPLC\VKV 523 ketone 802010 IG - INT7 - 1)

	Reten. Time [min]	Area [mV.s]	Height [mV]	Area [%]	Height [%]	W05 [min]	Compound Name
1	11.970	7701.645	224.577	46.0	55.7	0.47	
2	30.113	9035.744	178.863	54.0	44.3	0.81	
	Total	16737.389	403.440	100.0	100.0		

1-(4-Methoxyphenoxy)-3-phenoxy-1-(phenylsulfonyl)propan-2-ol 20g

^{1}H NMR (400 MHz, $CDCl_3$)

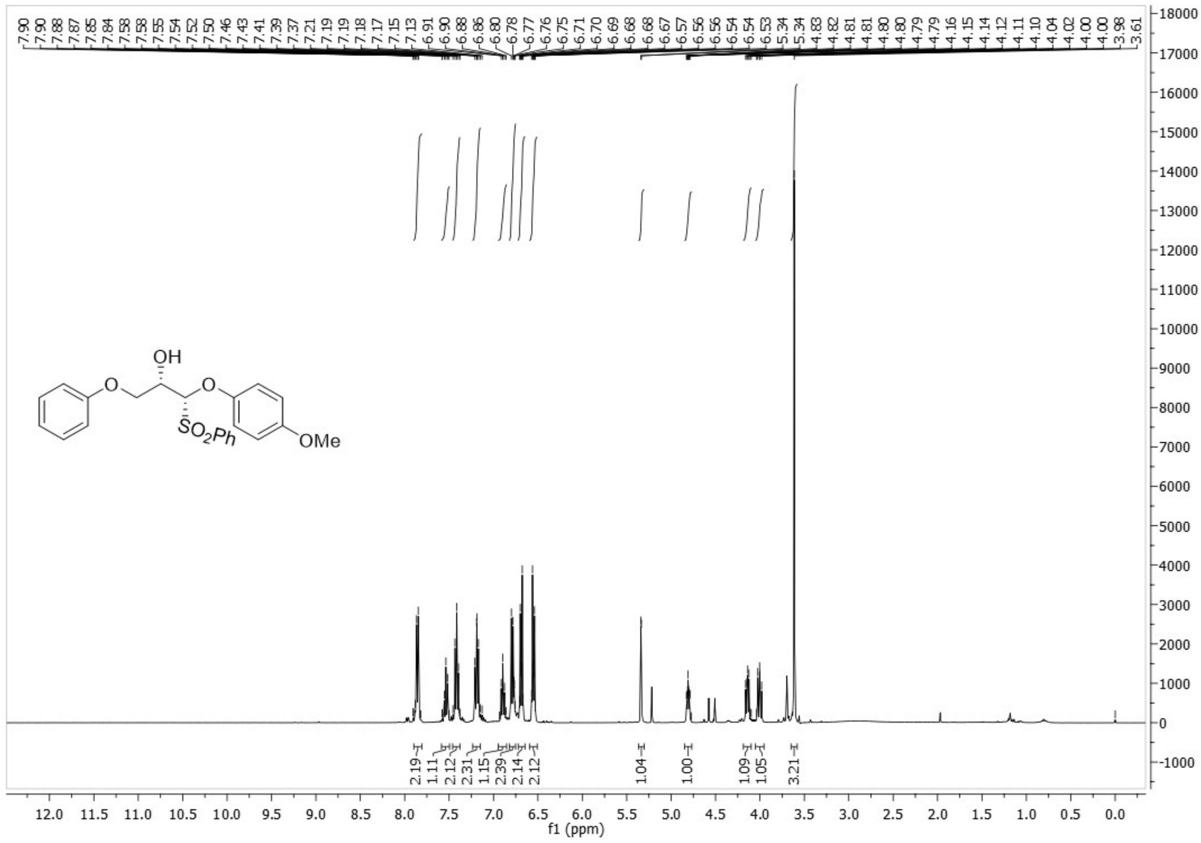

^{13}C NMR (101 MHz, $CDCl_3$)

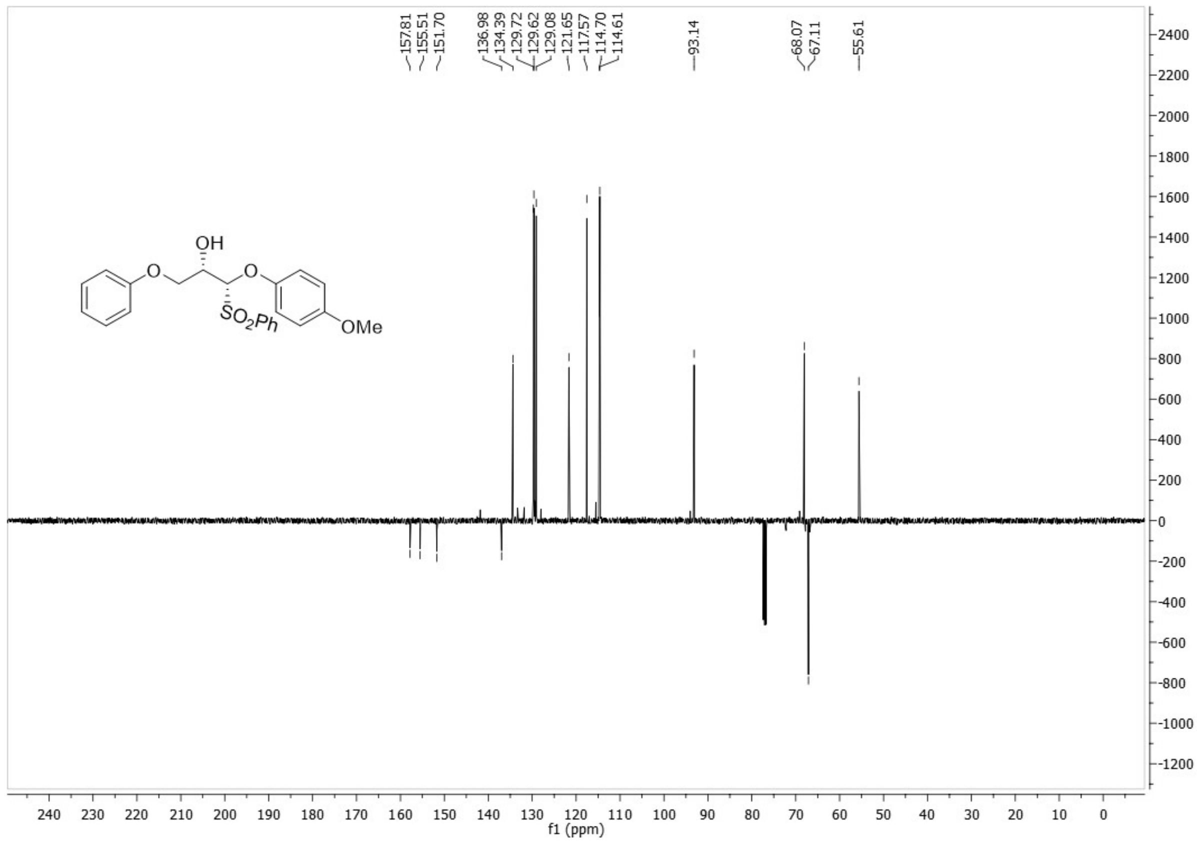

Racemic HPLC of 1-(4-methoxyphenoxy)-3-phenoxy-1-(phenylsulfonyl)propan-2-ol.

Clarity - Chromatography SW
DataApex 2006
www.dataapex.com

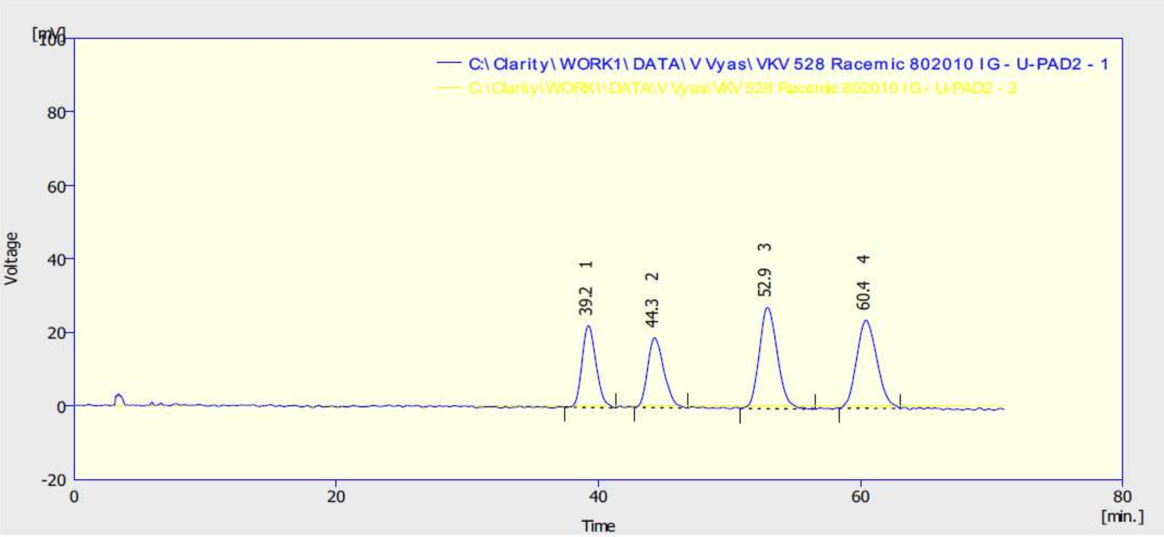

Result Table (Uncal - C:\Clarity\WORK1\DATA\V Vyas\VKV 528 Racemic 802010 IG - U-PAD2 - 1)

	Reten. Time [min]	Area [mV.s]	Height [mV]	Area [%]	Height [%]	W05 [min]	Compound Name
1	39.248	1657.142	22.283	19.4	24.0	1.16	
2	44.284	1643.478	18.997	19.2	20.5	1.33	
3	52.888	2654.586	27.518	31.1	29.7	1.48	
4	60.432	2587.603	23.995	30.3	25.9	1.70	
	Total	8542.808	92.793	100.0	100.0		

HPLC after ATH 1-(4-methoxyphenoxy)-3-phenoxy-1-(phenylsulfonyl)propan-2-ol. (100% conversion, 96.7:3.3 dr, 99.2% ee).

Clarity - Chromatography SW
DataApex 2006
www.dataapex.com

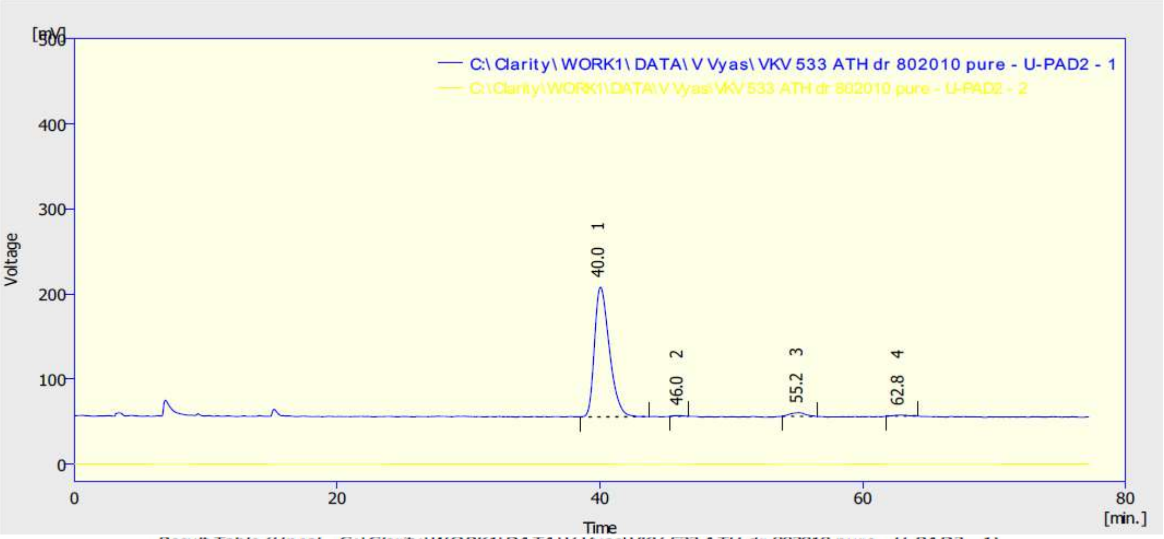

Result Table (Uncal - C:\Clarity\WORK1\DATA\V Vyas\VKV 533 ATH dr 802010 pure - U-PAD2 - 1)

	Reten. Time [min]	Area [mV.s]	Height [mV]	Area [%]	Height [%]	W05 [min]	Compound Name
1	40.048	11978.727	152.316	96.3	96.0	1.21	
2	46.044	45.516	0.899	0.4	0.6	0.89	
3	55.152	349.822	4.284	2.8	2.7	1.32	
4	62.848	68.692	1.084	0.6	0.7	1.10	
	Total	12442.757	158.584	100.0	100.0		

1-(4-Methoxyphenoxy)-3-phenoxy-1-(phenylsulfonyl)propan-2-one 19g

^{1}H NMR (400 MHz, $CDCl_3$)

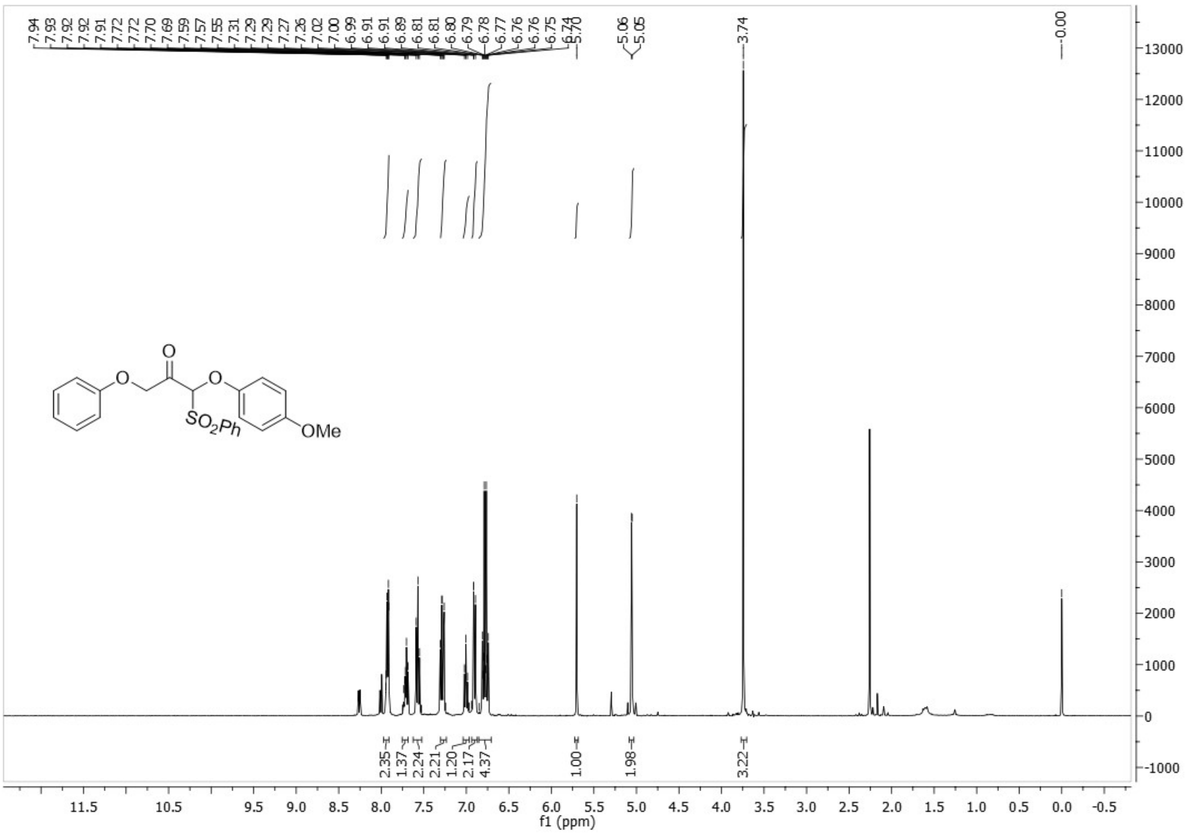

^{13}C NMR (101 MHz, $CDCl_3$)

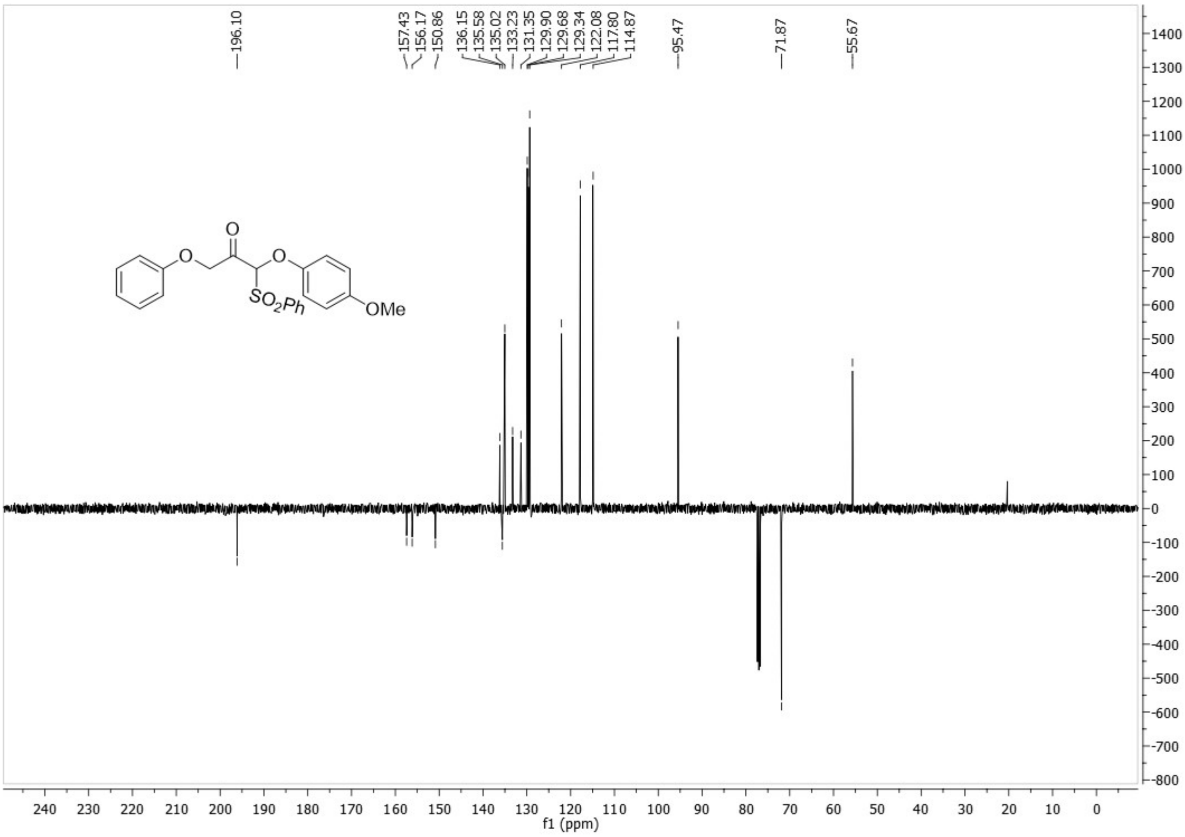

Ketone HPLC of 1-(4-Methoxyphenoxy)-3-phenoxy-1-(phenylsulfonyl)propan-2-one.

Clarity - Chromatography SW
DataApex 2006
www.dataapex.com

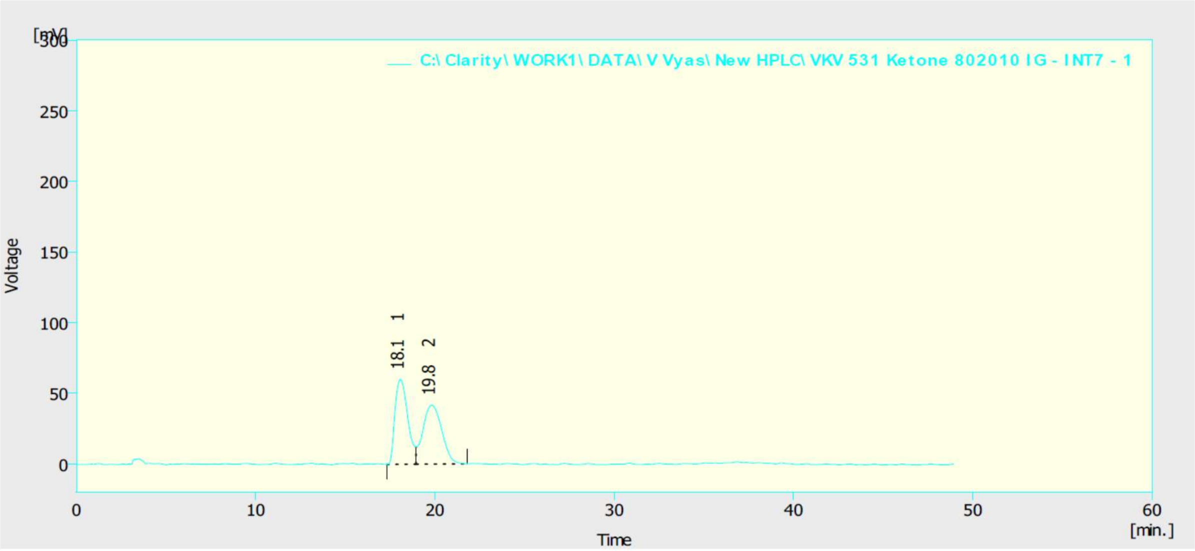

Result Table (Uncal - C:\Clarity\WORK1\DATA\V Vyas\New HPLC\VKV 531 Ketone 802010 IG - INT7 - 1)

	Reten. Time [min]	Area [mV.s]	Height [mV]	Area [%]	Height [%]	W05 [min]	Compound Name
1	18.073	2987.971	60.097	49.3	59.1	0.80	
2	19.820	3077.798	41.650	50.7	40.9	1.17	
	Total	6065.768	101.747	100.0	100.0		

1-Phenyl-3-(p-tolyl)propan-2-ol 21

^{1}H NMR (400 MHz, $CDCl_3$)

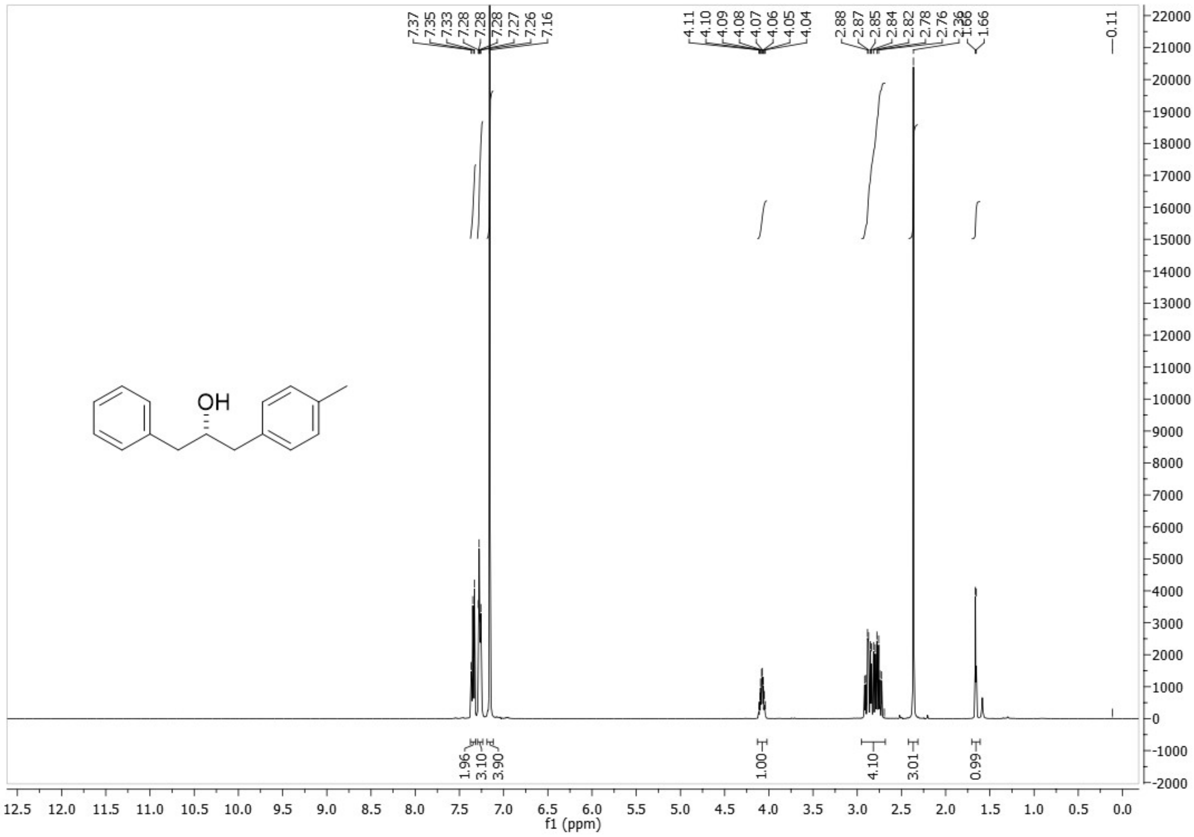

^{13}C NMR (101 MHz, $CDCl_3$)

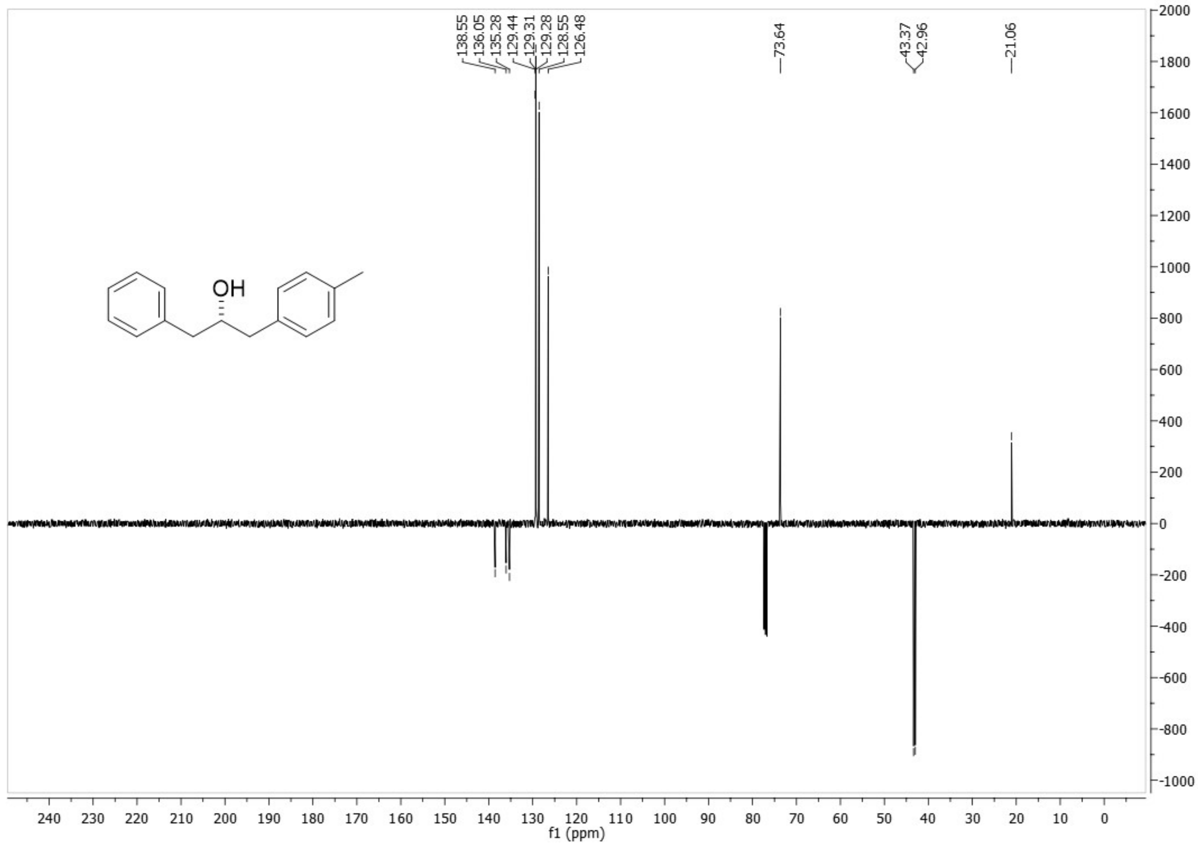

Racemic HPLC of 1-Phenyl-3-(p-tolyl)propan-2-ol.

Clarity - Chromatography SW
DataApex 2006
www.dataapex.com

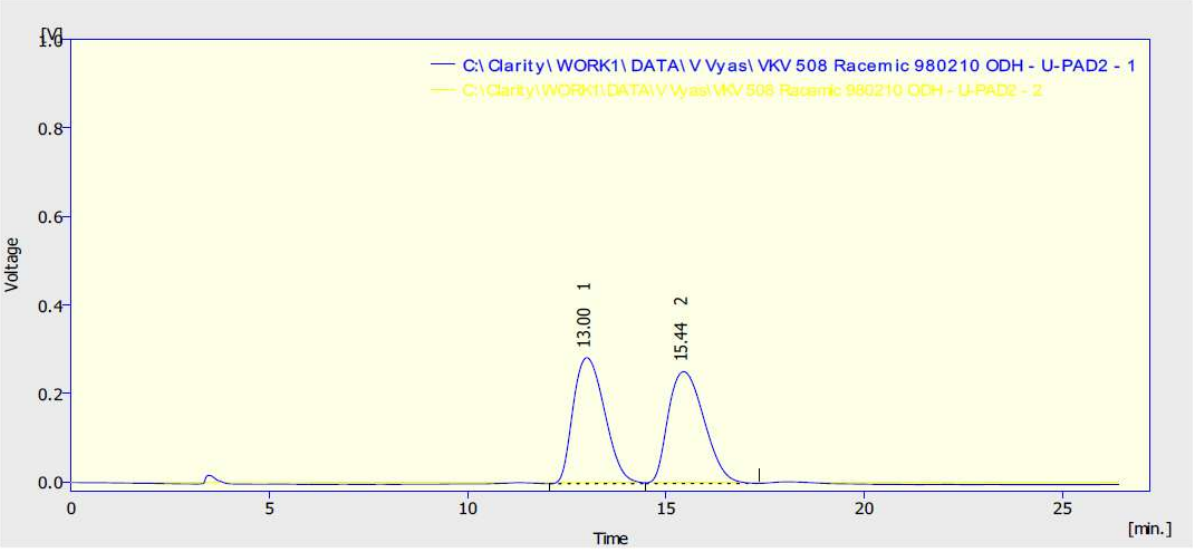

Result Table (Uncal - C:\Clarity\WORK1\DATA\V Vyas\VKV 508 Racemic 980210 ODH - U-PAD2 - 1)

	Reten. Time [min]	Area [mV.s]	Height [mV]	Area [%]	Height [%]	W05 [min]	Compound Name
1	13.000	15805.779	284.823	49.8	53.0	0.90	
2	15.444	15942.586	252.622	50.2	47.0	1.02	
	Total	31748.365	537.444	100.0	100.0		

HPLC after sulphone reduction 1-Phenyl-3-(p-tolyl)propan-2-ol. (100% conversion, 99.4% ee).

Clarity - Chromatography SW
DataApex 2006
www.dataapex.com

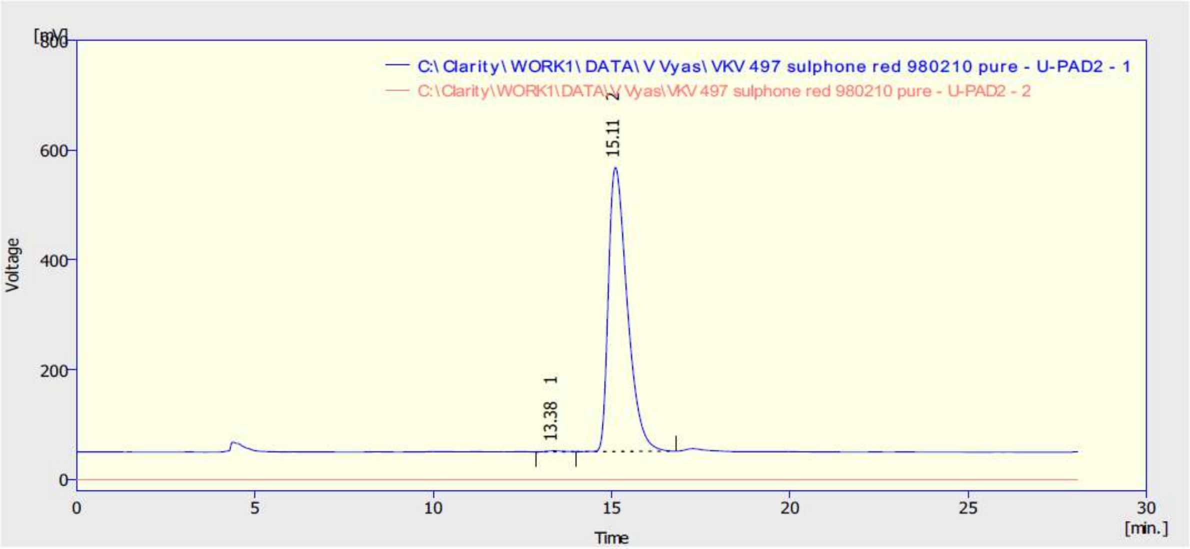

Result Table (Uncal - C:\Clarity\WORK1\DATA\V Vyas\VKV 497 sulphone red 980210 pure - U-PAD2 - 1)

	Reten. Time [min]	Area [mV.s]	Height [mV]	Area [%]	Height [%]	W05 [min]	Compound Name
1	13.376	59.584	1.729	0.3	0.3	0.59	
2	15.112	19032.725	517.502	99.7	99.7	0.56	
	Total	19092.309	519.230	100.0	100.0		

HPLC after ATH 1-Phenyl-3-(p-tolyl)propan-2-ol. (100% conversion, 0% ee).

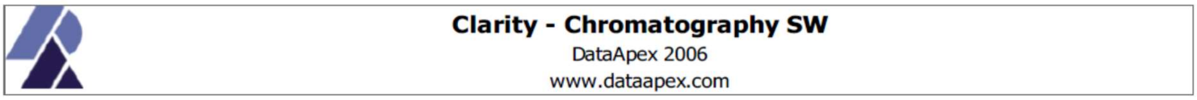

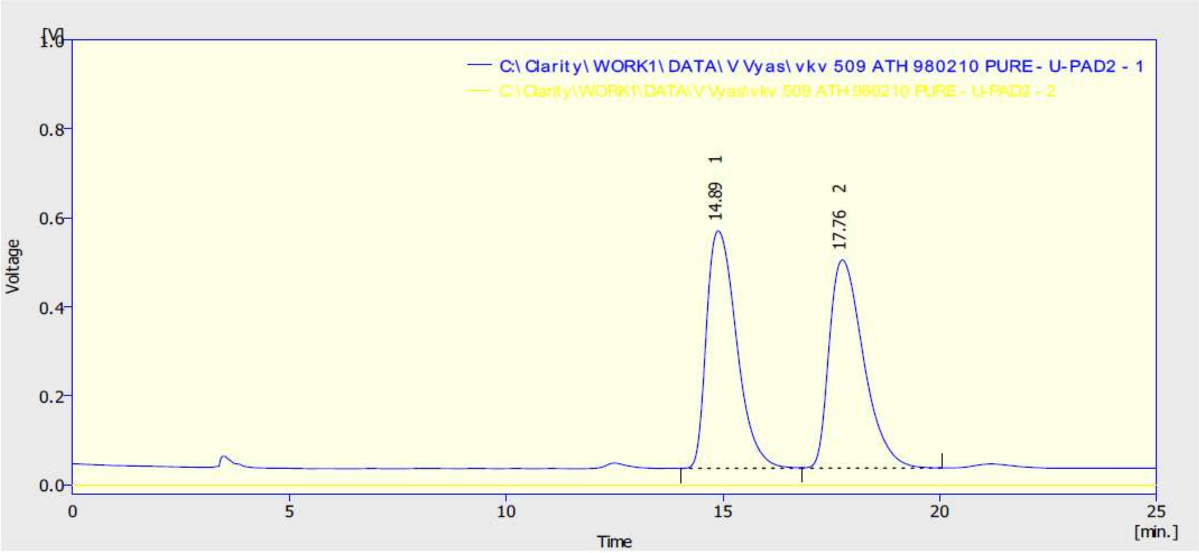

Result Table (Uncal - C:\Clarity\WORK1\DATA\V Vyas\vkv 509 ATH 980210 PURE - U-PAD2 - 1)

	Reten. Time [min]	Area [mV.s]	Height [mV]	Area [%]	Height [%]	W05 [min]	Compound Name
1	14.892	25742.384	533.500	50.0	53.3	0.76	
2	17.756	25766.419	467.938	50.0	46.7	0.85	
	Total	51508.803	1001.437	100.0	100.0		

1-Phenoxy-3-phenylpropan-2-ol 22

^{1}H NMR (400 MHz, $CDCl_3$)

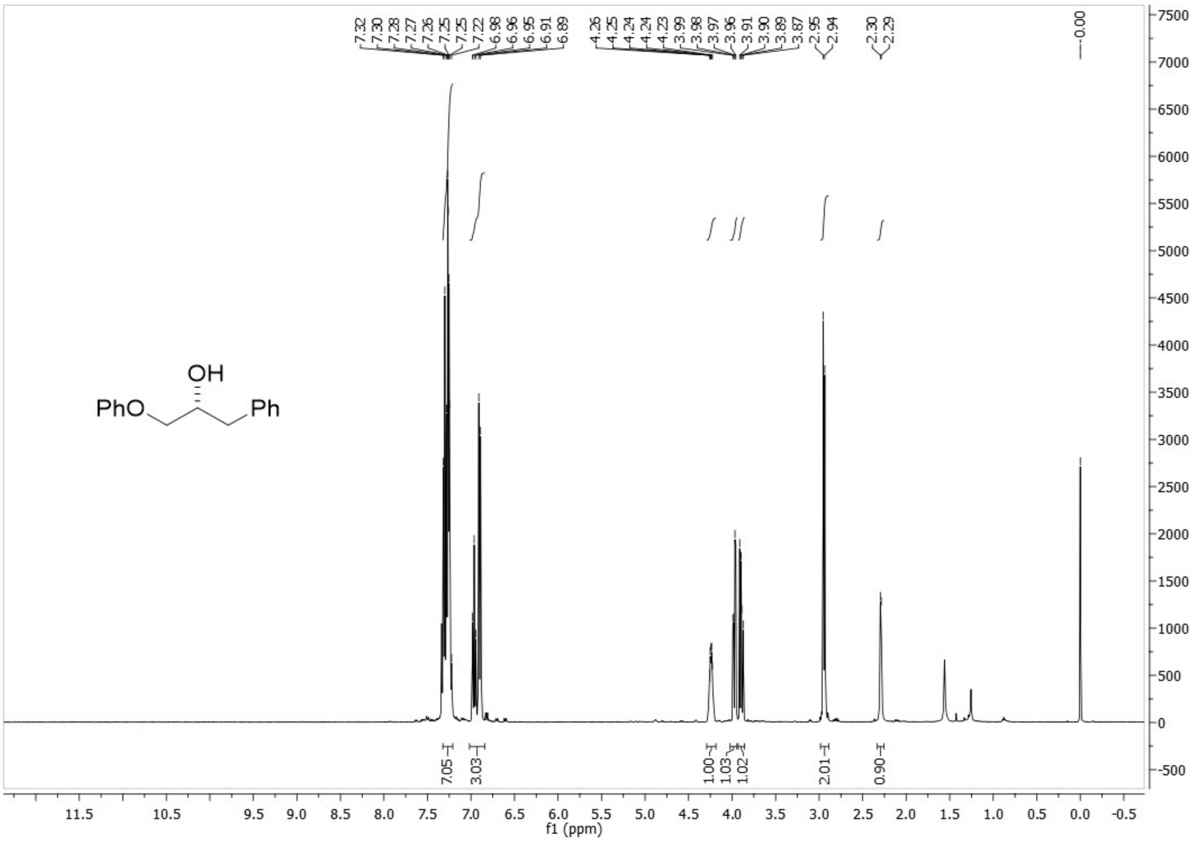

^{13}C NMR (101 MHz, $CDCl_3$)

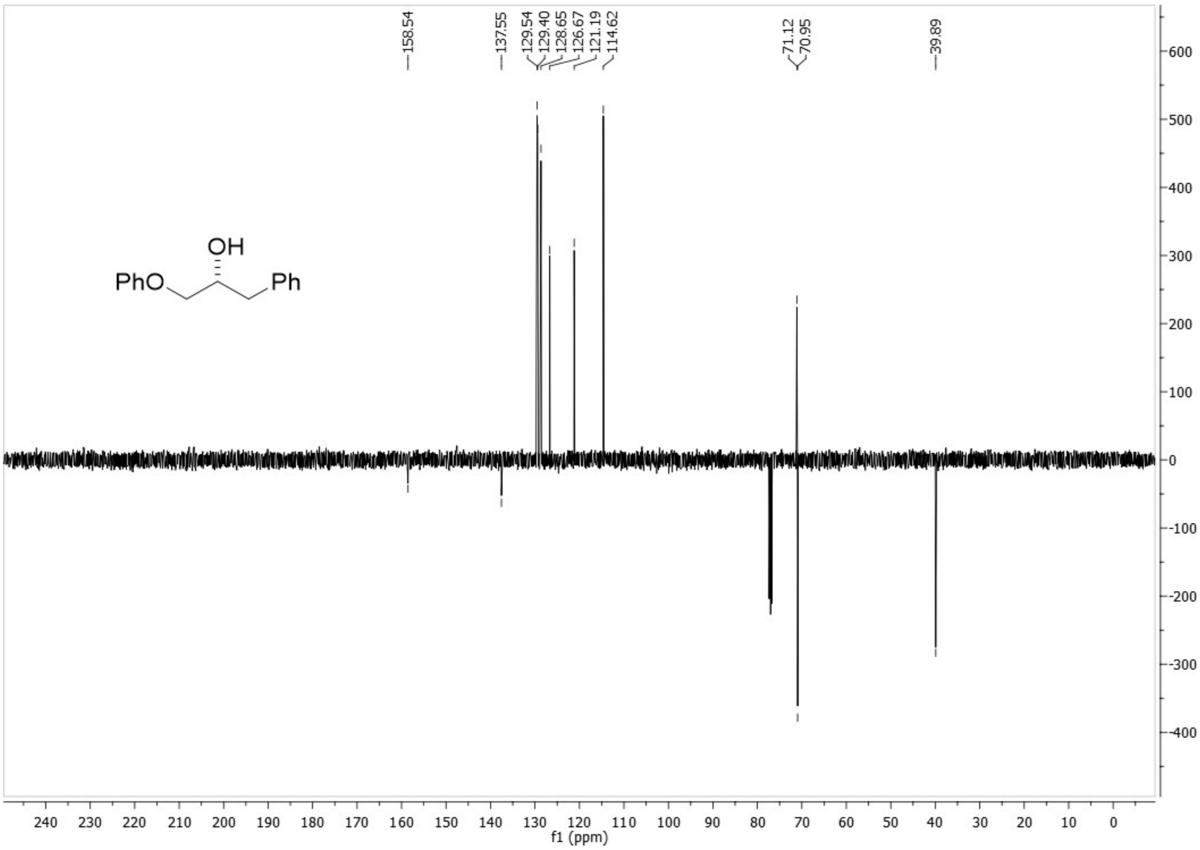

Racemic HPLC of 1-phenoxy-3-phenylpropan-2-ol.

```
=====================================================================
Acq. Operator   : SYSTEM                          Seq. Line :   1
Acq. Instrument : Pattison HPLC                    Location : Vial 1
Injection Date  : 22/07/2019 11:19:36                   Inj :   1
                                                Inj Volume : 20.000 µl
Method          : C:\CHEM32\1\DATA\DEF_LC 2019-07-22 11-17-55\VIJYESH 2.M (Sequence Method)
Last changed    : 22/07/2019 11:17:57 by SYSTEM
Additional Info : Peak(s) manually integrated
```

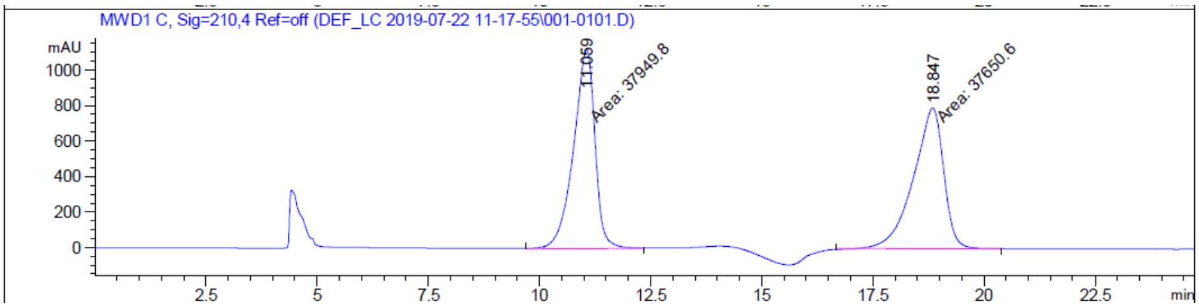

Signal 2: MWD1 C, Sig=210,4 Ref=off

Peak #	RetTime [min]	Type	Width [min]	Area [mAU*s]	Height [mAU]	Area %
1	11.059	MM	0.5621	3.79498e4	1125.23560	50.1979
2	18.847	MM	0.7917	3.76506e4	792.61230	49.8021

HPLC after sulphone reduction 1-phenoxy-3-phenylpropan-2-ol. (100% conversion, 99% ee).

```
Acq. Operator   : SYSTEM                          Seq. Line :   1
Acq. Instrument : Pattison HPLC                    Location : Vial 1
Injection Date  : 26/07/2019 18:35:21                   Inj :   1
                                                Inj Volume : 20.000 µl
Method          : C:\CHEM32\1\DATA\DEF_LC 2019-07-26 18-33-42\VIJYESH 2.M (Sequence Method)
Last changed    : 26/07/2019 18:33:42 by SYSTEM
```

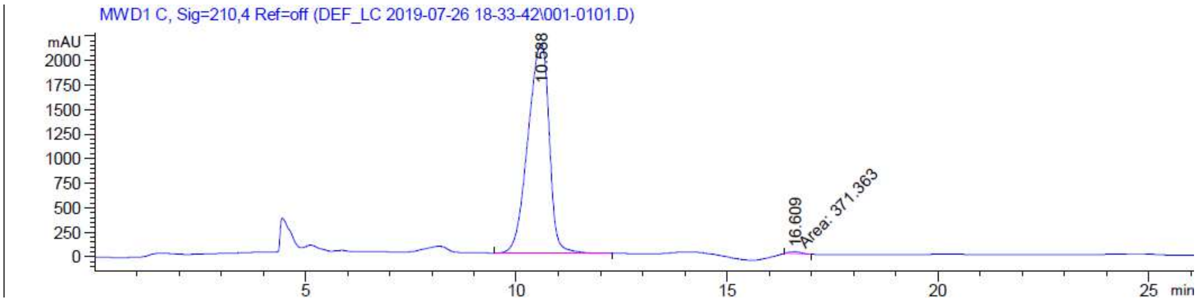

Signal 2: MWD1 C, Sig=210,4 Ref=off

Peak #	RetTime [min]	Type	Width [min]	Area [mAU*s]	Height [mAU]	Area %
1	10.588	BB	0.5691	7.48031e4	2133.33594	99.5060
2	16.609	MM	0.3415	371.36264	18.12333	0.4940

HPLC after ATH 1-phenoxy-3-phenylpropan-2-ol. (100% conversion, 64.2% ee).

```
Acq. Operator   : SYSTEM                          Seq. Line :   1
Acq. Instrument : Pattison HPLC                    Location : Vial 1
Injection Date  : 22/07/2019 11:47:03                   Inj :   1
                                                 Inj Volume : 20.000 µl
Method          : C:\CHEM32\1\DATA\DEF_LC 2019-07-22 11-45-25\VIJYESH 2.M (Sequence Method)
Last changed    : 22/07/2019 11:45:27 by SYSTEM
Additional Info : Peak(s) manually integrated
```

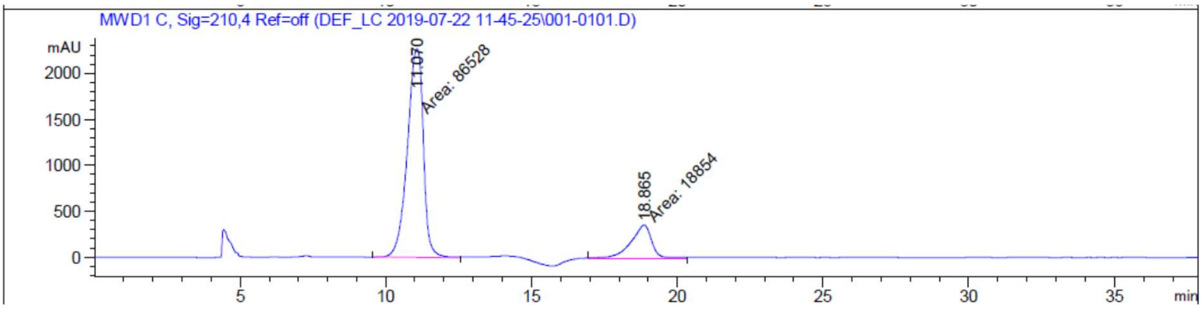

Signal 2: MWD1 C, Sig=210,4 Ref=off

Peak #	RetTime [min]	Type	Width [min]	Area [mAU*s]	Height [mAU]	Area %
1	11.070	MM	0.6369	8.65280e4	2264.26807	82.1089
2	18.865	MM	0.8613	1.88540e4	364.82535	17.8911

1-Phenoxy-3-(p-tolyloxy)propan-2-ol 23

^{1}H NMR (400 MHz, $CDCl_3$)

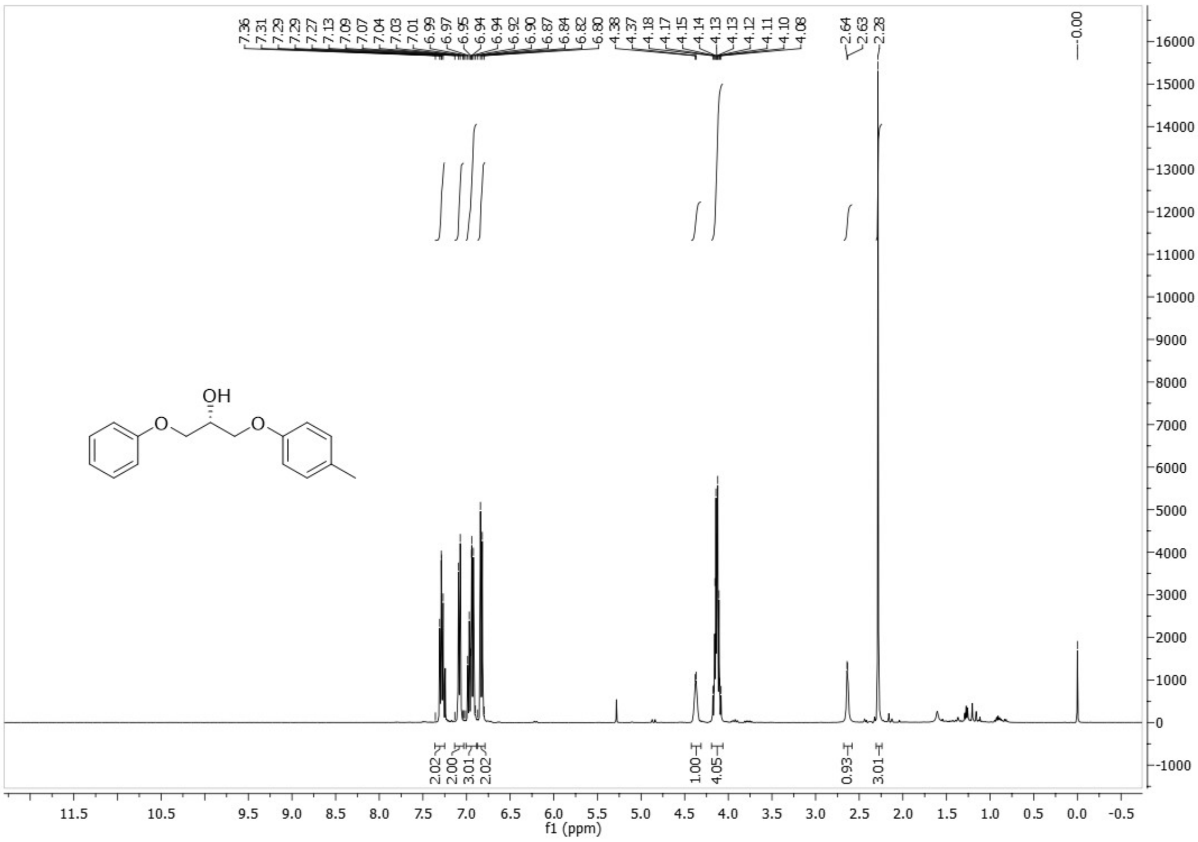

^{13}C NMR (101 MHz, $CDCl_3$)

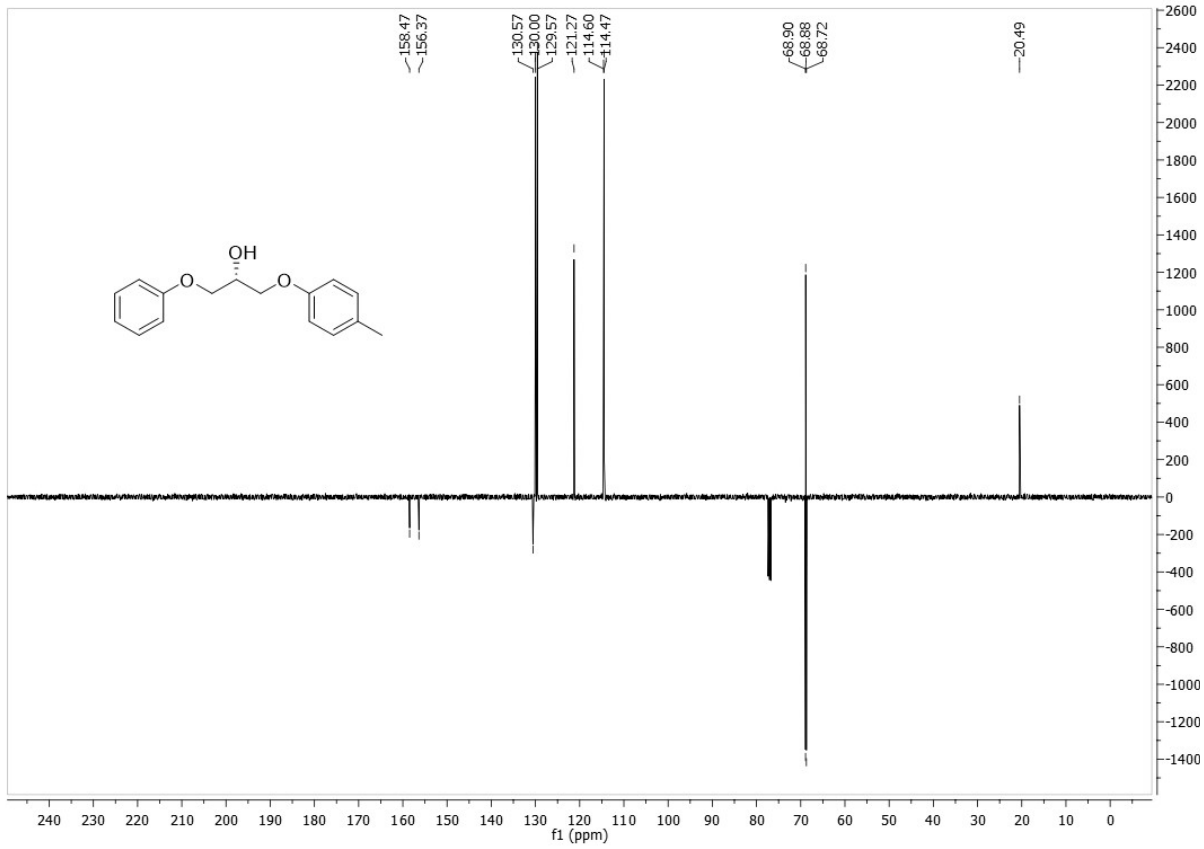

Racemic HPLC of 1-phenoxy-3-(p-tolyloxy)propan-2-ol.

Clarity - Chromatography SW
DataApex 2006
www.dataapex.com

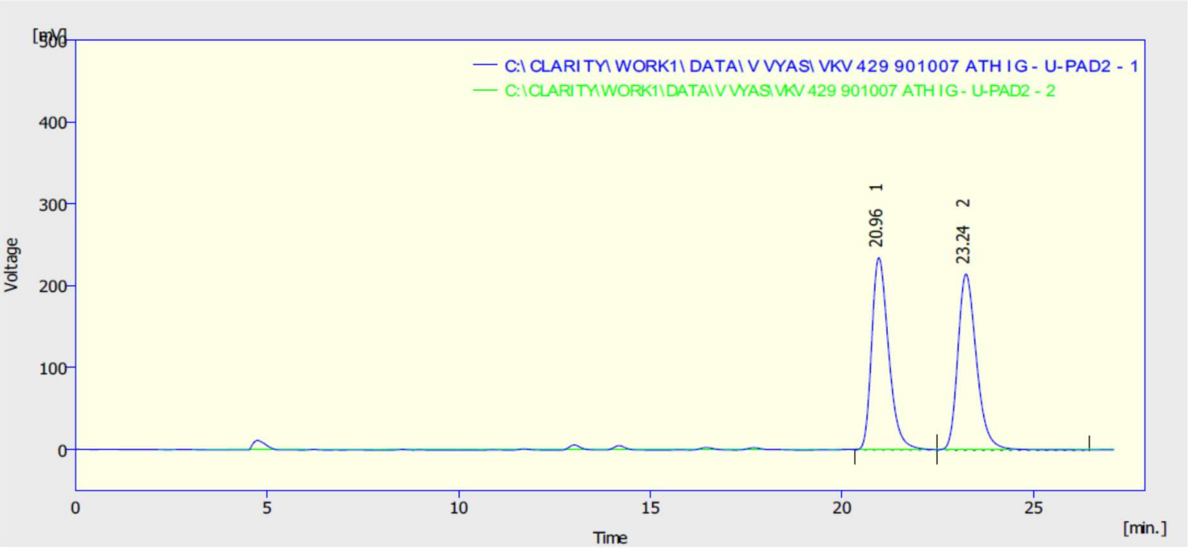

Result Table (Uncal - C:\CLARITY\WORK1\DATA\V VYAS\VKV 429 901007 ATH IG - U-PAD2 - 1)

	Reten. Time [min]	Area [mV.s]	Height [mV]	Area [%]	Height [%]	W05 [min]	Compound Name
1	20.964	7178.426	234.571	49.8	52.2	0.47	
2	23.236	7244.259	214.801	50.2	47.8	0.52	
	Total	14422.685	449.372	100.0	100.0		

HPLC after sulphone reduction 1-phenoxy-3-(p-tolyloxy)propan-2-ol. (100% conversion, 99.8% ee).

Clarity - Chromatography SW
DataApex 2006
www.dataapex.com

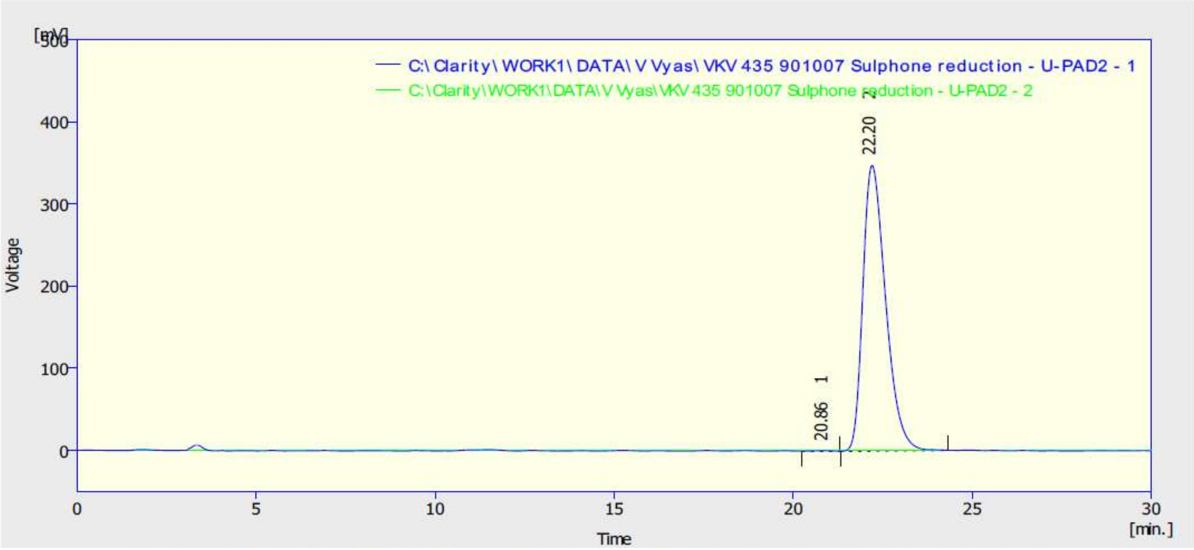

Result Table (Uncal - C:\Clarity\WORK1\DATA\V Vyas\VKV 435 901007 Sulphone reduction - U-PAD2 - 1)

	Reten. Time [min]	Area [mV.s]	Height [mV]	Area [%]	Height [%]	W05 [min]	Compound Name
1	20.856	20.428	0.610	0.1	0.2	0.55	
2	22.204	15362.792	347.805	99.9	99.8	0.68	
	Total	15383.220	348.415	100.0	100.0		

HPLC after ATH 1-phenoxy-3-(p-tolyloxy)propan-2-ol. (100% conversion, 4.6% ee).

Clarity - Chromatography SW
DataApex 2006
www.dataapex.com

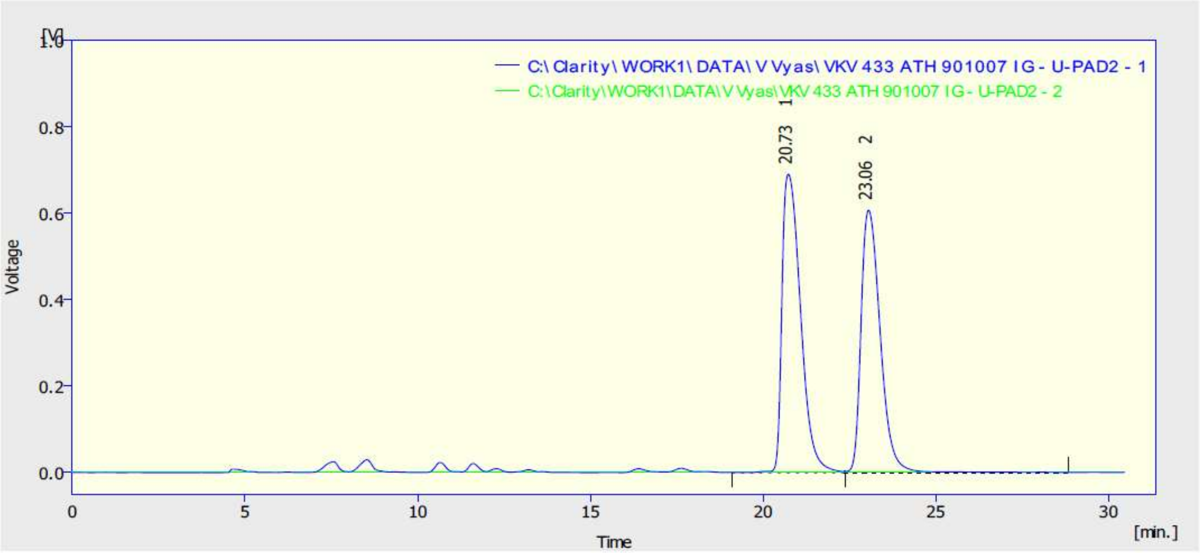

Result Table (Uncal - C:\Clarity\WORK1\DATA\V Vyas\VKV 433 ATH 901007 IG - U-PAD2 - 1)

	Reten. Time [min]	Area [mV.s]	Height [mV]	Area [%]	Height [%]	W05 [min]	Compound Name
1	20.732	25760.906	689.944	52.3	53.2	0.58	
2	23.056	23455.666	606.755	47.7	46.8	0.60	
	Total	49216.572	1296.700	100.0	100.0		

1-Methoxy-3-phenoxypropan-2-ol 24

^{1}H NMR (400 MHz, $CDCl_3$)

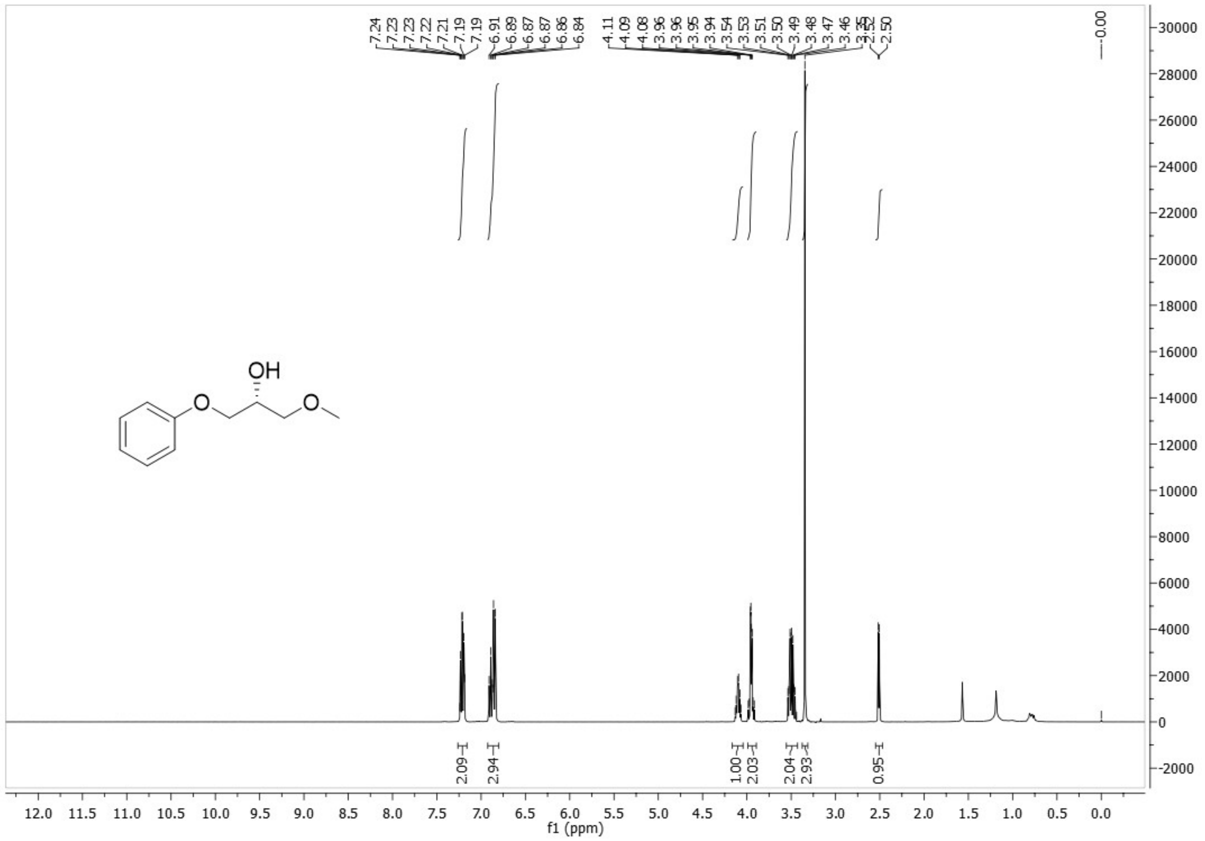

^{13}C NMR (101 MHz, $CDCl_3$)

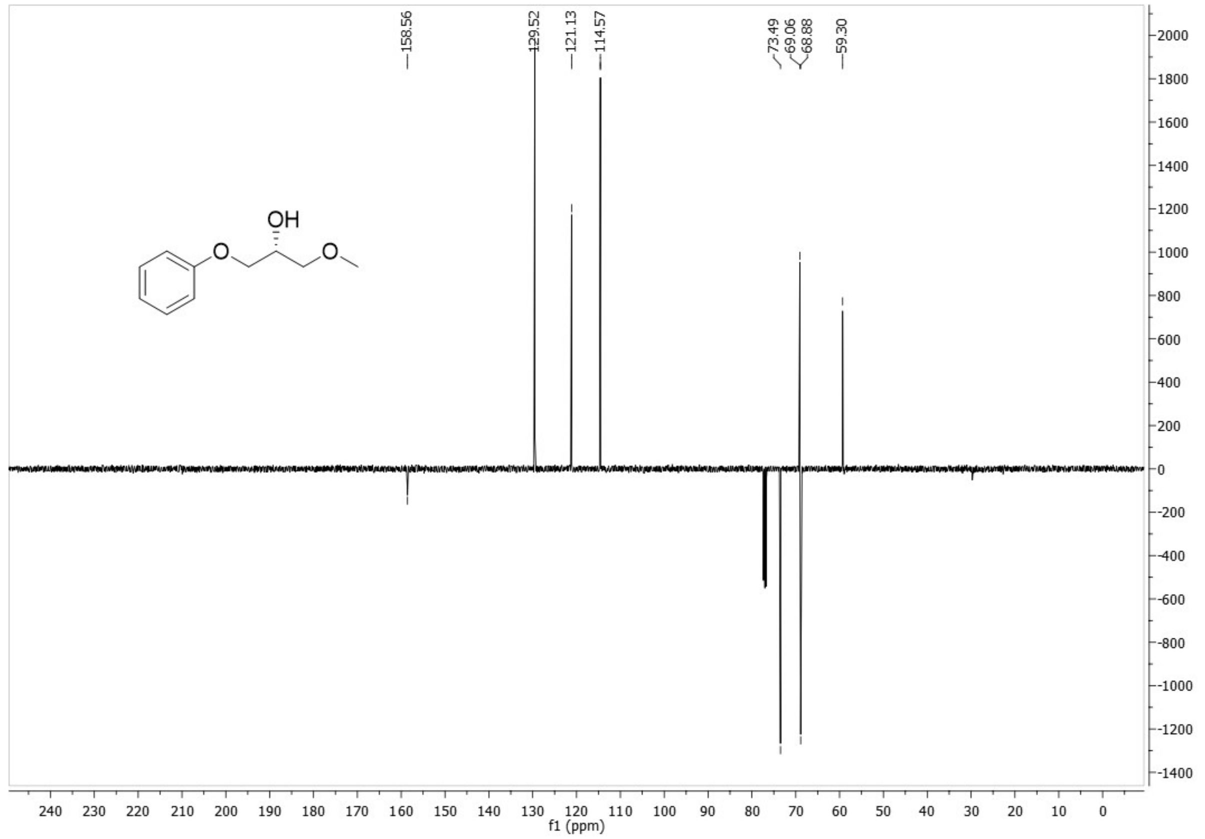

Racemic HPLC of 1-methoxy-3-phenoxypropan-2-ol.

Clarity - Chromatography SW
DataApex 2006
www.dataapex.com

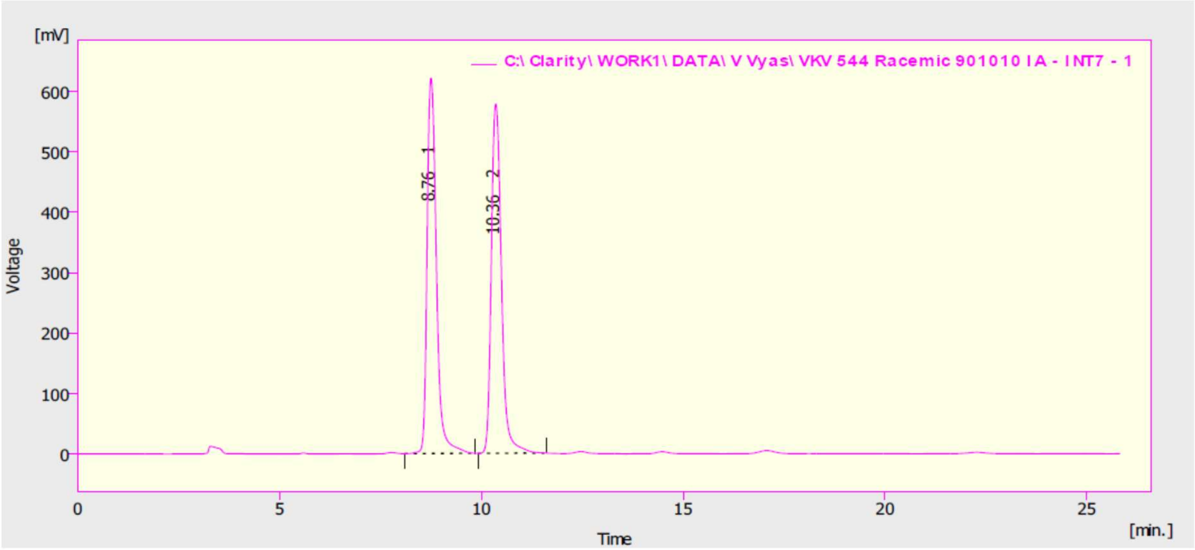

Result Table (Uncal - C:\Clarity\WORK1\DATA\V Vyas\VKV 544 Racemic 901010 IA - INT7 - 1)

	Reten. Time [min]	Area [mV.s]	Height [mV]	Area [%]	Height [%]	W05 [min]	Compound Name
1	8.757	9939.726	621.871	49.7	51.8	0.24	
2	10.360	10056.344	578.731	50.3	48.2	0.26	
	Total	19996.070	1200.602	100.0	100.0		

HPLC after sulphone reduction 1-methoxy-3-phenoxypropan-2-ol. (100% conversion, >99.9% ee).

Clarity - Chromatography SW
DataApex 2006
www.dataapex.com

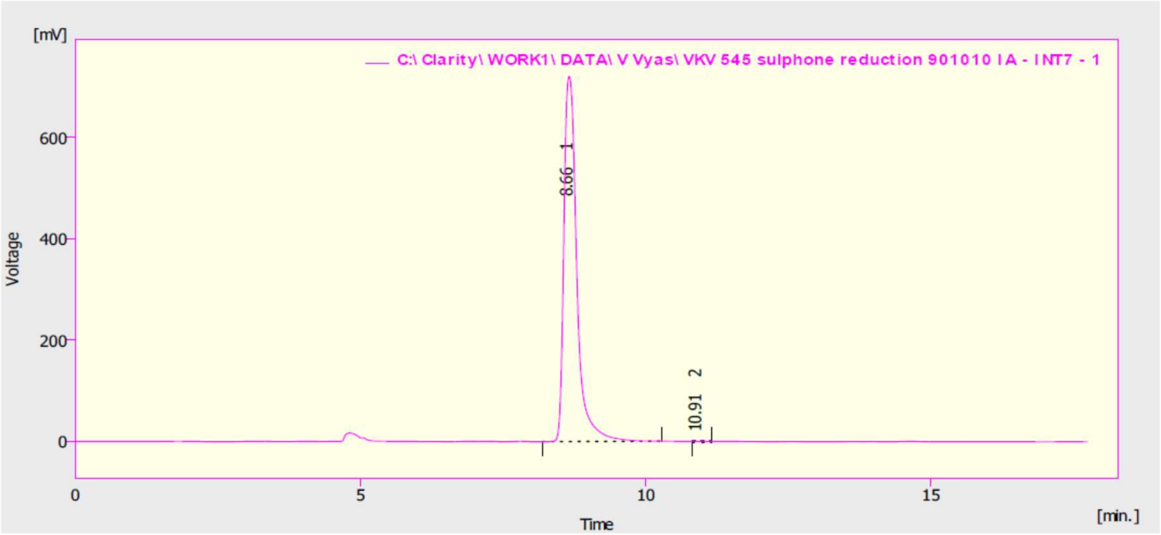

Result Table (Uncal - C:\Clarity\WORK1\DATA\V Vyas\VKV 545 sulphone reduction 901010 IA - INT7 - 1)

	Reten. Time [min]	Area [mV.s]	Height [mV]	Area [%]	Height [%]	W05 [min]	Compound Name
1	8.657	11354.443	720.778	100.0	100.0	0.23	
2	10.910	0.713	0.068	0.0	0.0	0.13	
	Total	11355.157	720.847	100.0	100.0		

1-(4-Methoxyphenoxy)-3-phenoxypropan-2-ol 25

^{1}H NMR (400 MHz, $CDCl_3$)

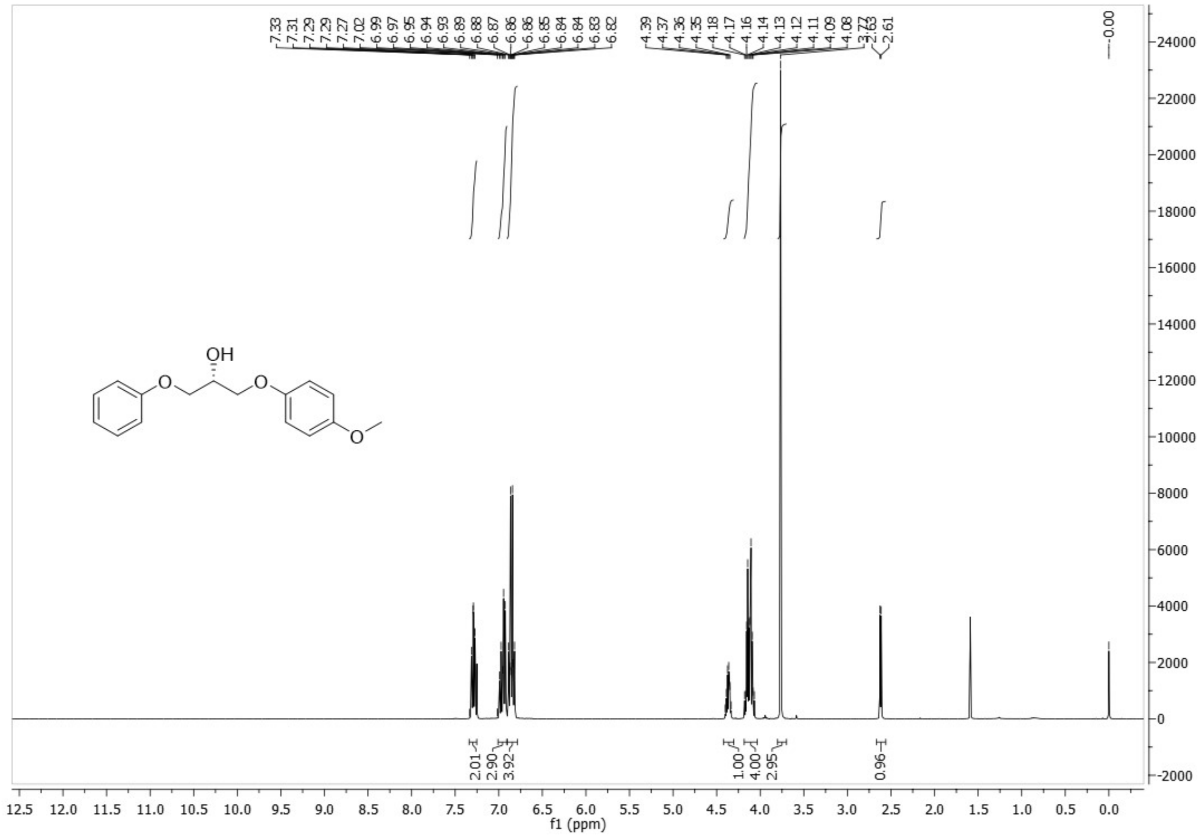

^{13}C NMR (101 MHz, $CDCl_3$)

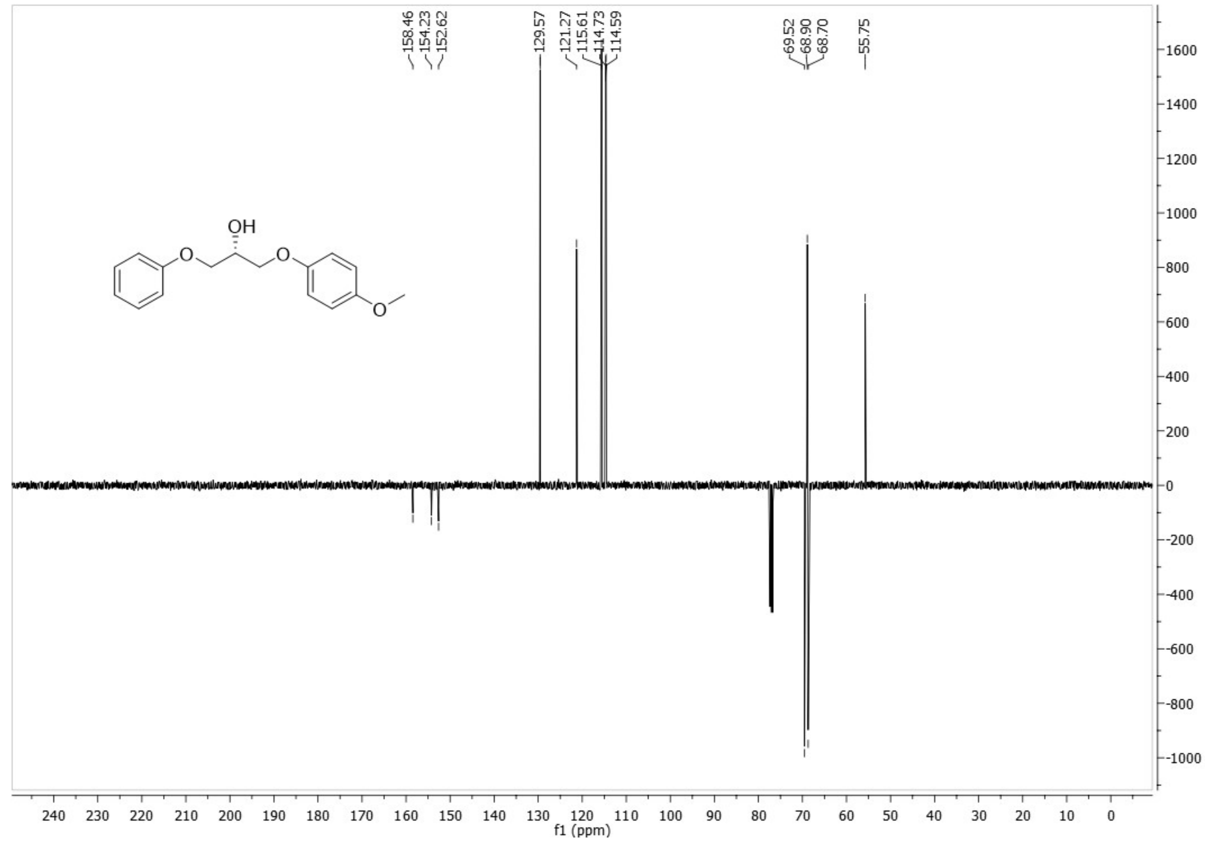

Racemic HPLC of 1-(4-methoxyphenoxy)-3-phenoxypropan-2-ol.

Clarity - Chromatography SW
DataApex 2006
www.dataapex.com

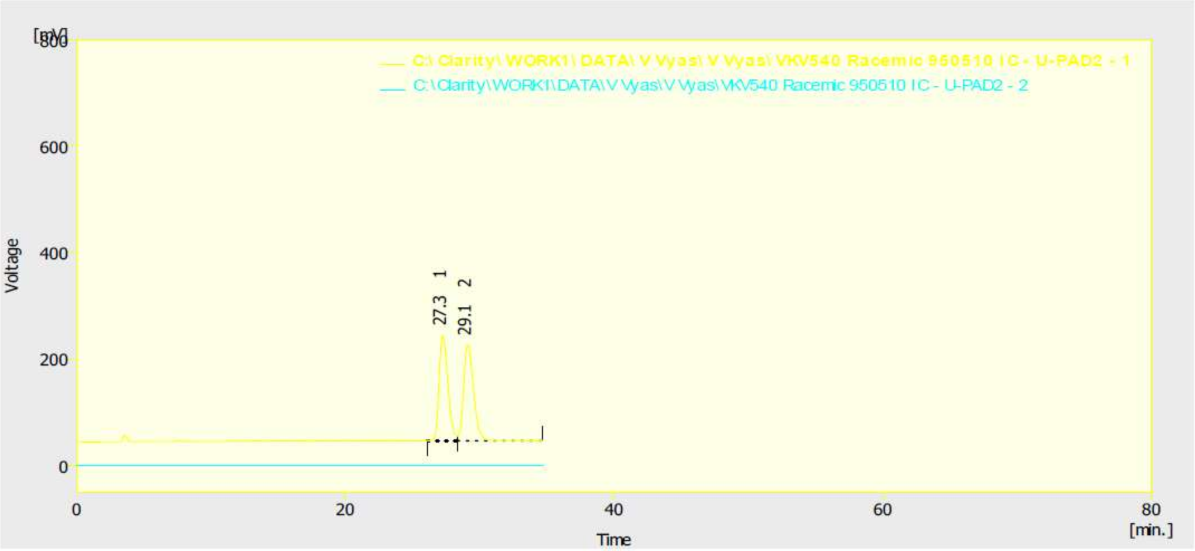

Result Table (Uncal - C:\Clarity\WORK1\DATA\V Vyas\V Vyas\VKV540 Racemic 950510 IC - U-PAD2 - 1)

	Reten. Time [min]	Area [mV.s]	Height [mV]	Area [%]	Height [%]	W05 [min]	Compound Name
1	27.268	9142.115	197.552	49.3	52.3	0.72	
2	29.124	9410.139	180.292	50.7	47.7	0.80	
	Total	18552.254	377.844	100.0	100.0		

HPLC after sulphone reduction 1-(4-methoxyphenoxy)-3-phenoxypropan-2-ol. (100% conversion, 95.4% ee).

Clarity - Chromatography SW
DataApex 2006
www.dataapex.com

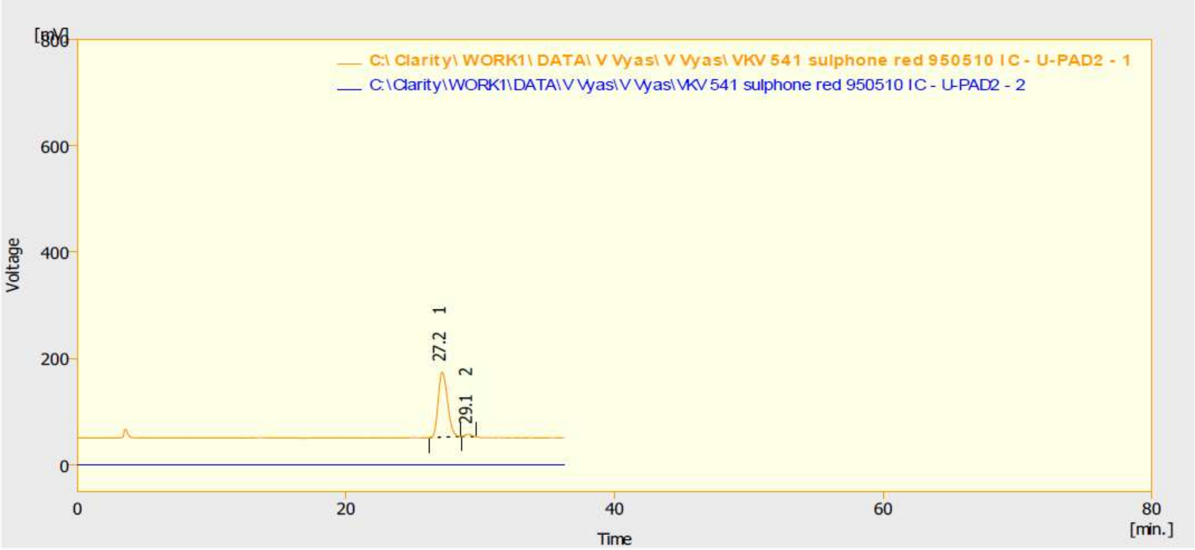

Result Table (Uncal - C:\Clarity\WORK1\DATA\V Vyas\V Vyas\VKV 541 sulphone red 950510 IC - U-PAD2 - 1)

	Reten. Time [min]	Area [mV.s]	Height [mV]	Area [%]	Height [%]	W05 [min]	Compound Name
1	27.176	5890.457	122.312	97.7	96.8	0.76	
2	29.132	138.773	3.998	2.3	3.2	0.58	
	Total	6029.229	126.310	100.0	100.0		

3-Phenoxy-1-(phenylsulfonyl)butan-2-ol

^{1}H NMR (400 MHz, $CDCl_3$)

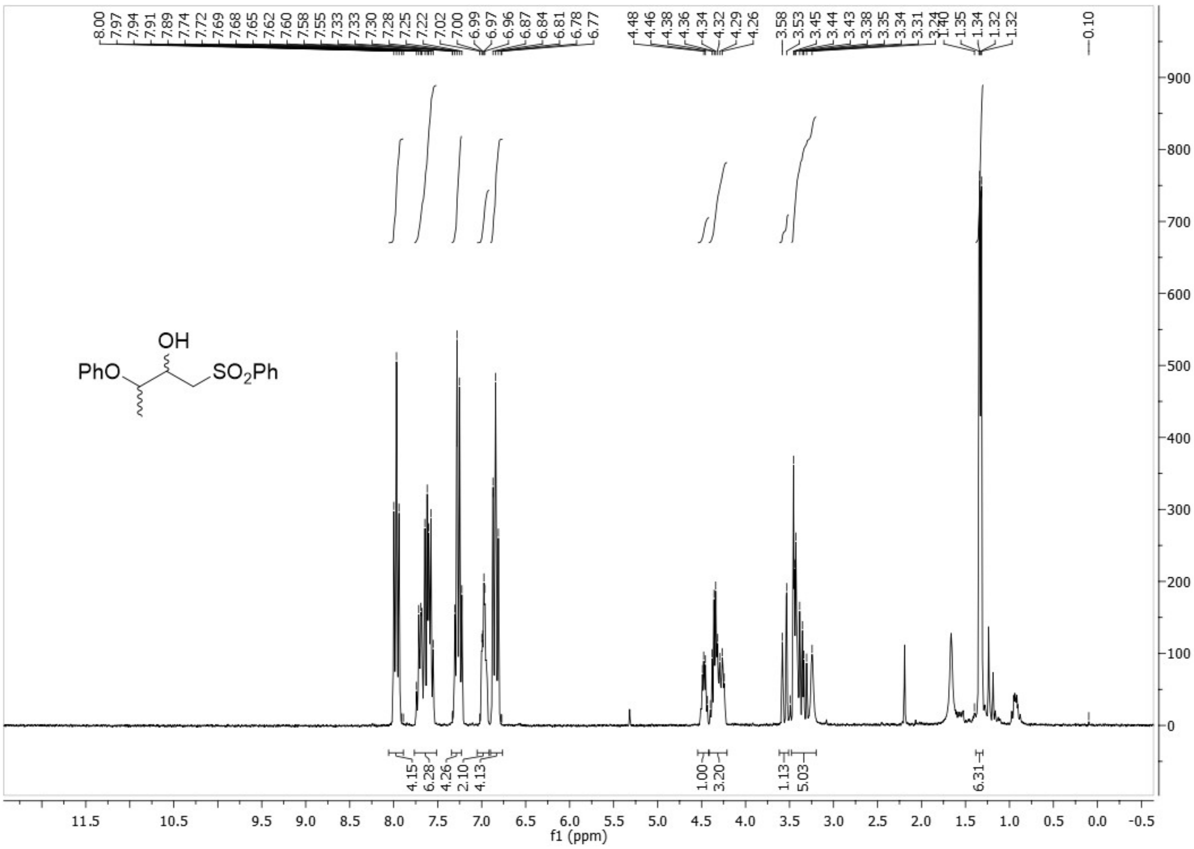

^{13}C NMR (101 MHz, $CDCl_3$

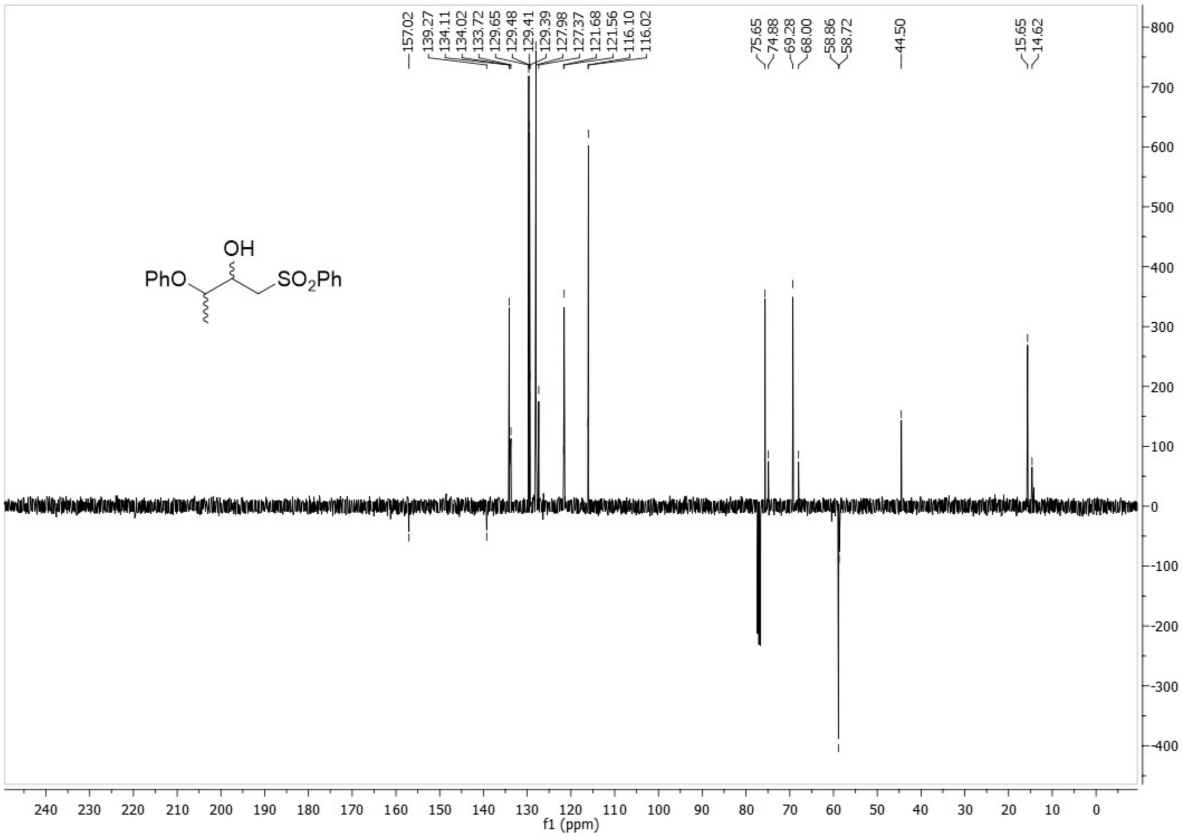

Racemic HPLC of 3-Phenoxy-1-(phenylsulfonyl)butan-2-ol.

Clarity - Chromatography SW
DataApex 2006
www.dataapex.com

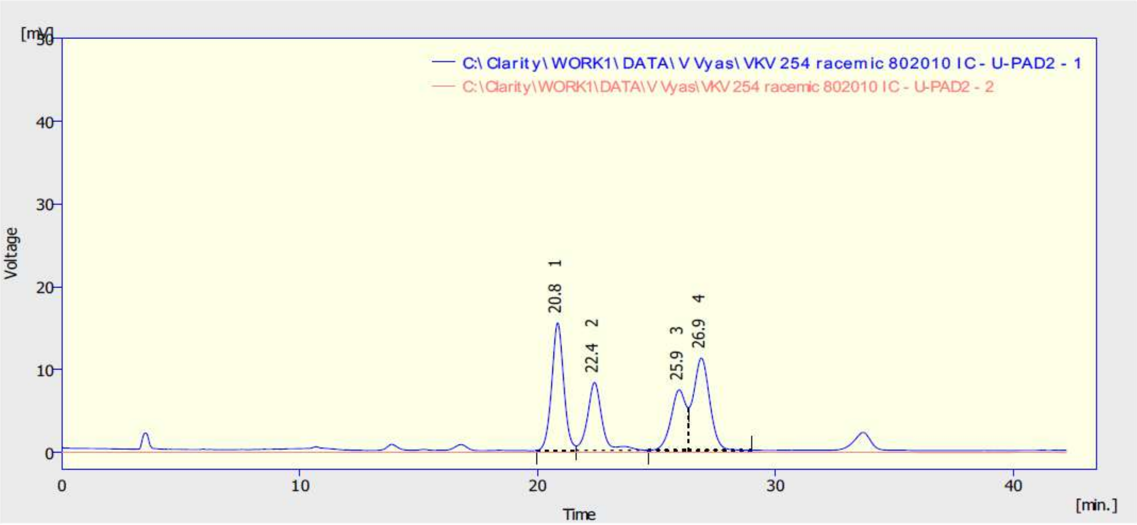

Result Table (Uncal - C:\Clarity\WORK1\DATA\V Vyas\VKV 254 racemic 802010 IC - U-PAD2 - 1)

	Reten. Time [min]	Area [mV.s]	Height [mV]	Area [%]	Height [%]	W05 [min]	Compound Name
1	20.840	539.375	15.392	31.0	36.6	0.53	
2	22.392	346.785	8.193	19.9	19.5	0.59	
3	25.948	320.221	7.317	18.4	17.4	0.75	
4	26.880	536.170	11.148	30.8	26.5	0.81	
	Total	1742.552	42.050	100.0	100.0		

HPLC after ATH 3-Phenoxy-1-phenyl-1-(phenylsulfonyl)propan-2-ol. (100% conversion, 52.5:47.5 dr, >99.9% ee).

Clarity - Chromatography SW
DataApex 2006
www.dataapex.com

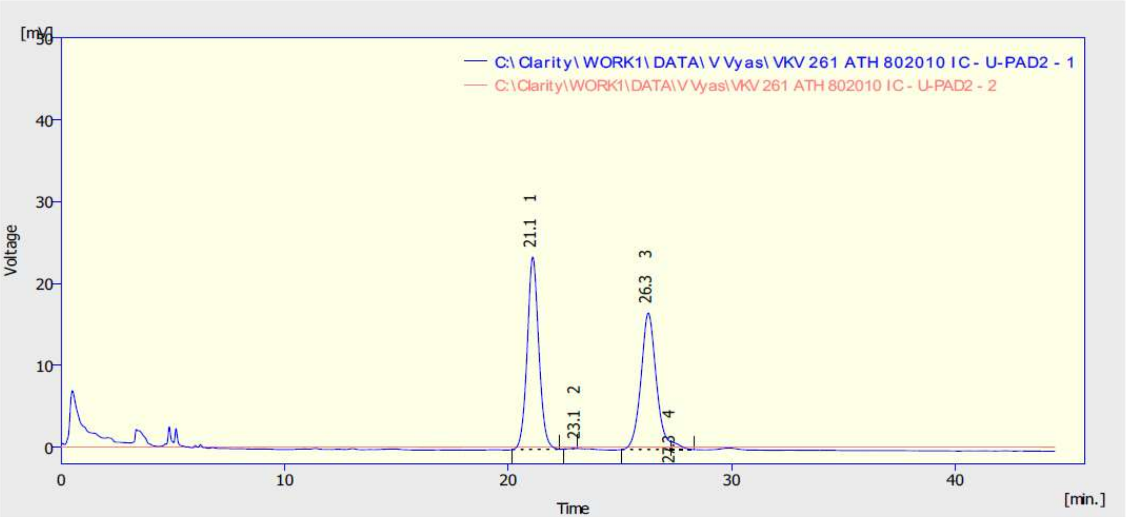

Result Table (Uncal - C:\Clarity\WORK1\DATA\V Vyas\VKV 261 ATH 802010 IC - U-PAD2 - 1)

	Reten. Time [min]	Area [mV.s]	Height [mV]	Area [%]	Height [%]	W05 [min]	Compound Name
1	21.100	849.964	23.503	51.0	57.1	0.55	
2	23.064	0.748	0.013	0.0	0.0	0.17	
3	26.272	791.011	16.666	47.5	40.5	0.71	
4	27.300	24.467	0.951	1.5	2.3	0.42	
	Total	1666.190	41.134	100.0	100.0		

3-Phenoxy-1-(phenylsulfonyl)butan-2-one 26

^{1}H NMR (400 MHz, $CDCl_3$)

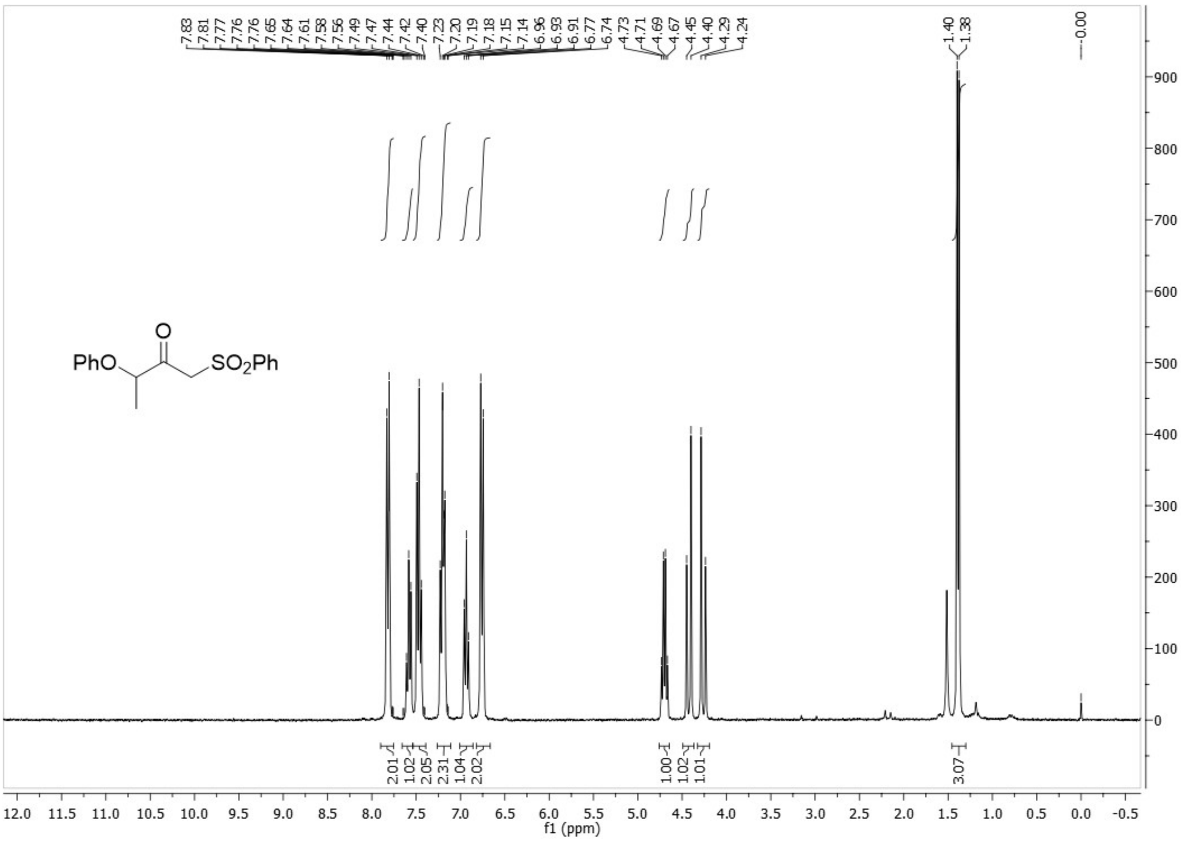

^{13}C NMR (101 MHz, $CDCl_3$)

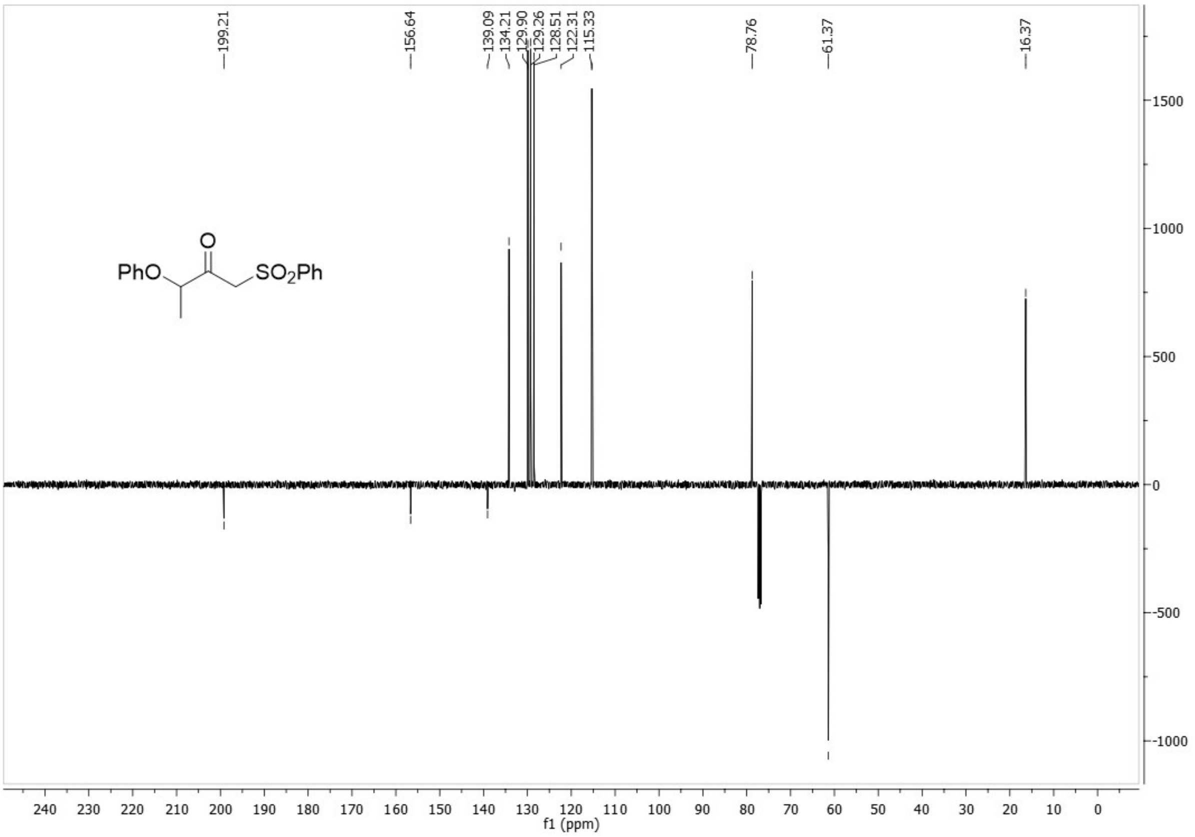

Ketone HPLC of 3-Phenoxy-1-(phenylsulfonyl)butan-2-one.

Clarity - Chromatography SW
DataApex 2006
www.dataapex.com

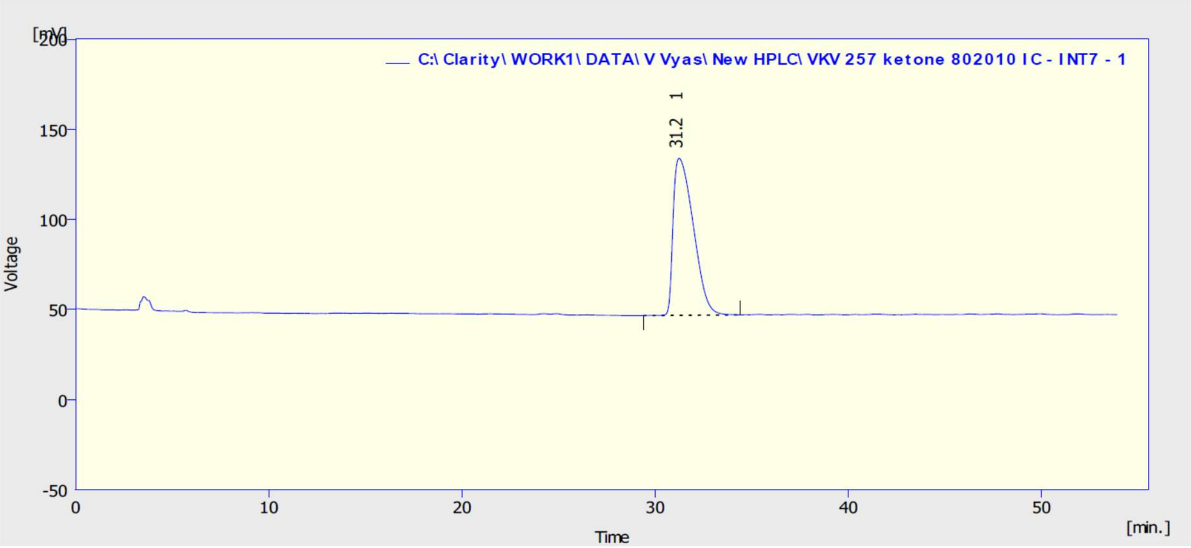

Result Table (Uncal - C:\Clarity\WORK1\DATA\V Vyas\New HPLC\VKV 257 ketone 802010 IC - INT7 - 1)

	Reten. Time [min]	Area [mV.s]	Height [mV]	Area [%]	Height [%]	W05 [min]	Compound Name
1	31.243	6315.480	87.150	100.0	100.0	1.17	
	Total	6315.480	87.150	100.0	100.0		

3-Phenyl-1-(phenylsulfonyl)butan-2-ol

^{1}H NMR (400 MHz, $CDCl_3$)

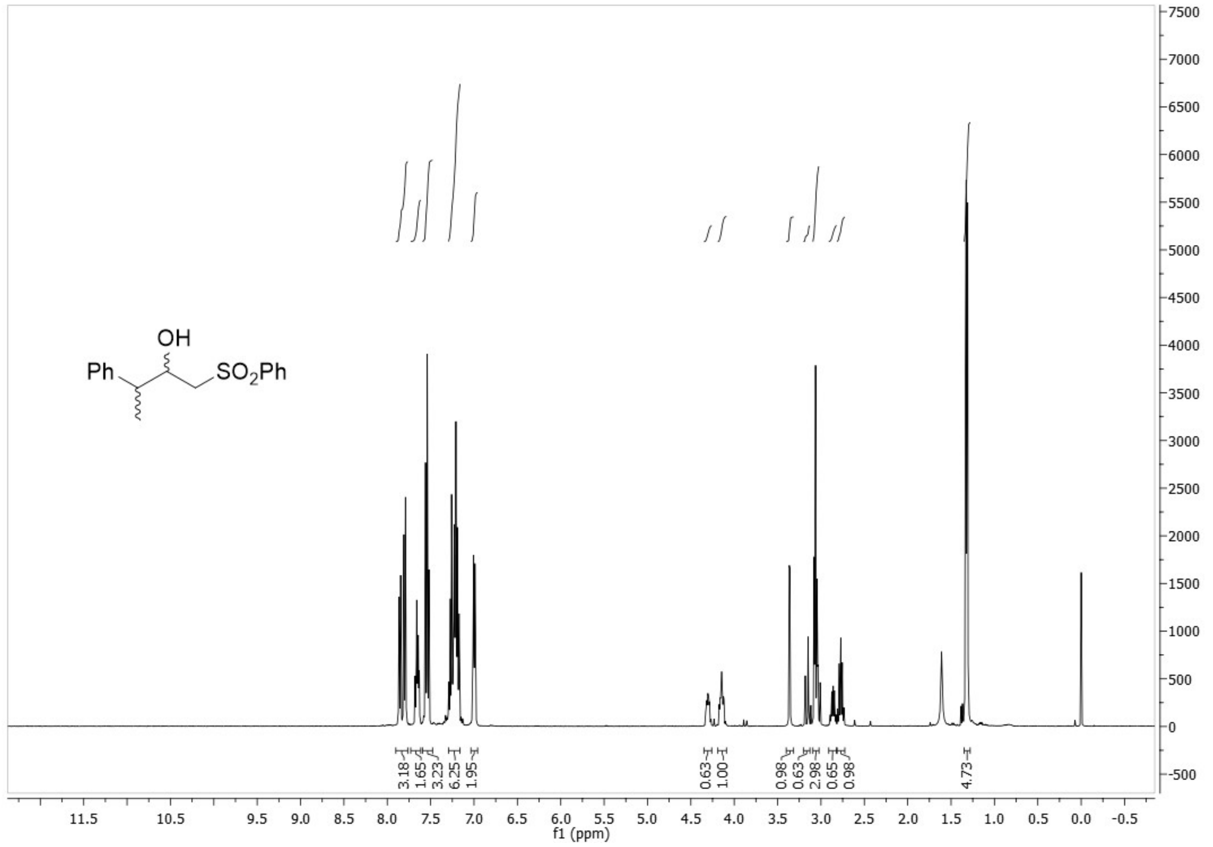

^{13}C NMR (101 MHz, $CDCl_3$)

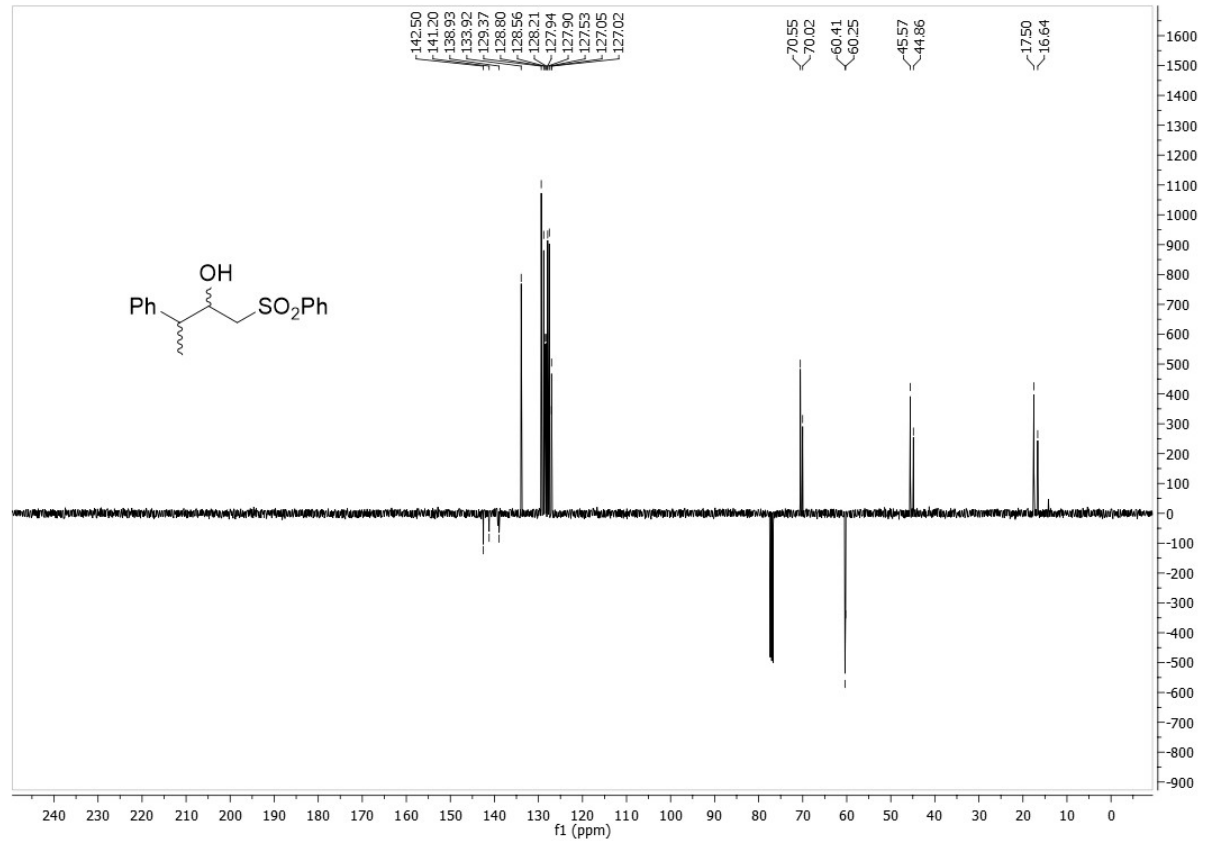

Racemic HPLC of 3-Phenyl-1-(phenylsulfonyl)butan-2-ol.

Clarity - Chromatography SW
DataApex 2006
www.dataapex.com

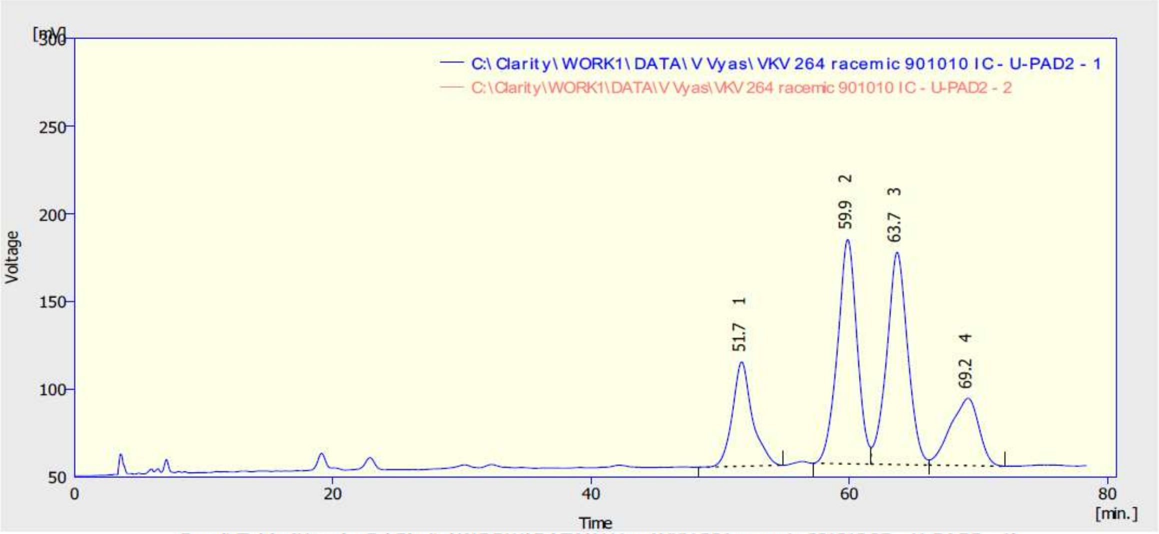

Result Table (Uncal - C:\Clarity\WORK1\DATA\V Vyas\VKV 264 racemic 901010 IC - U-PAD2 - 1)

	Reten. Time [min]	Area [mV.s]	Height [mV]	Area [%]	Height [%]	W05 [min]	Compound Name
1	51.676	6383.737	59.495	16.2	17.1	1.50	
2	59.880	13481.546	127.901	34.2	36.9	1.61	
3	63.704	13693.097	121.067	34.7	34.9	1.72	
4	69.204	5910.136	38.456	15.0	11.1	2.52	
	Total	39468.516	346.919	100.0	100.0		

HPLC after ATH 3-Phenyl-1-(phenylsulfonyl)butan-2-ol. (100% conversion, 59.5:40.5 dr, 83.4% ee).

Clarity - Chromatography SW
DataApex 2006
www.dataapex.com

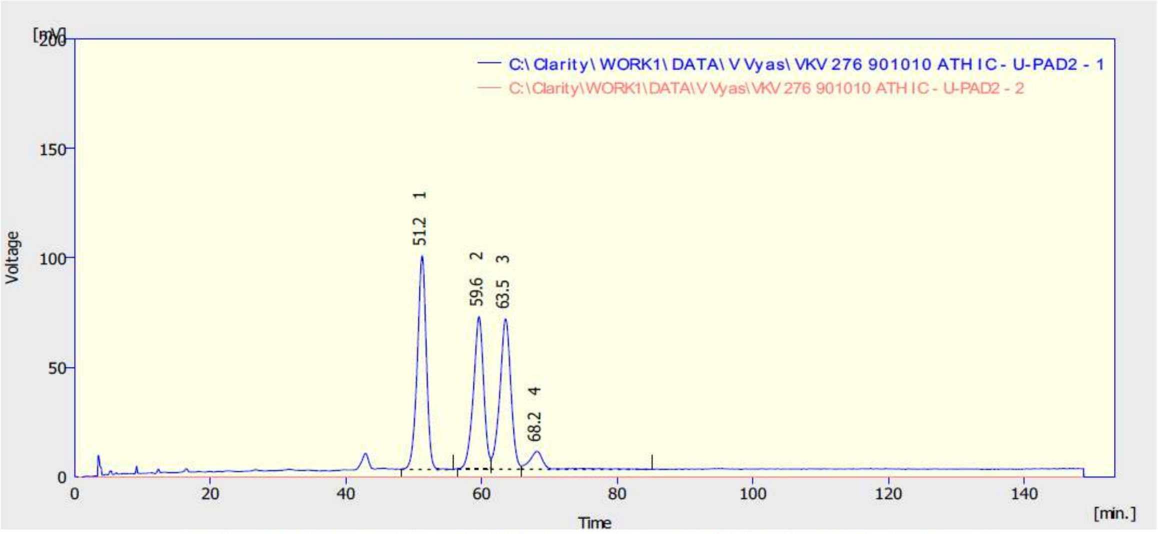

Result Table (Uncal - C:\Clarity\WORK1\DATA\V Vyas\VKV 276 901010 ATH IC - U-PAD2 - 1)

	Reten. Time [min]	Area [mV.s]	Height [mV]	Area [%]	Height [%]	W05 [min]	Compound Name
1	51.240	9032.408	97.589	35.6	40.0	1.40	
2	59.624	7361.527	69.704	29.0	28.6	1.60	
3	63.544	7750.970	68.664	30.5	28.1	1.71	
4	68.196	1261.890	8.183	5.0	3.4	1.93	
	Total	25406.795	244.140	100.0	100.0		

3-Phenyl-1-(phenylsulfonyl)butan-2-one 27

^{1}H NMR (400 MHz, $CDCl_3$)

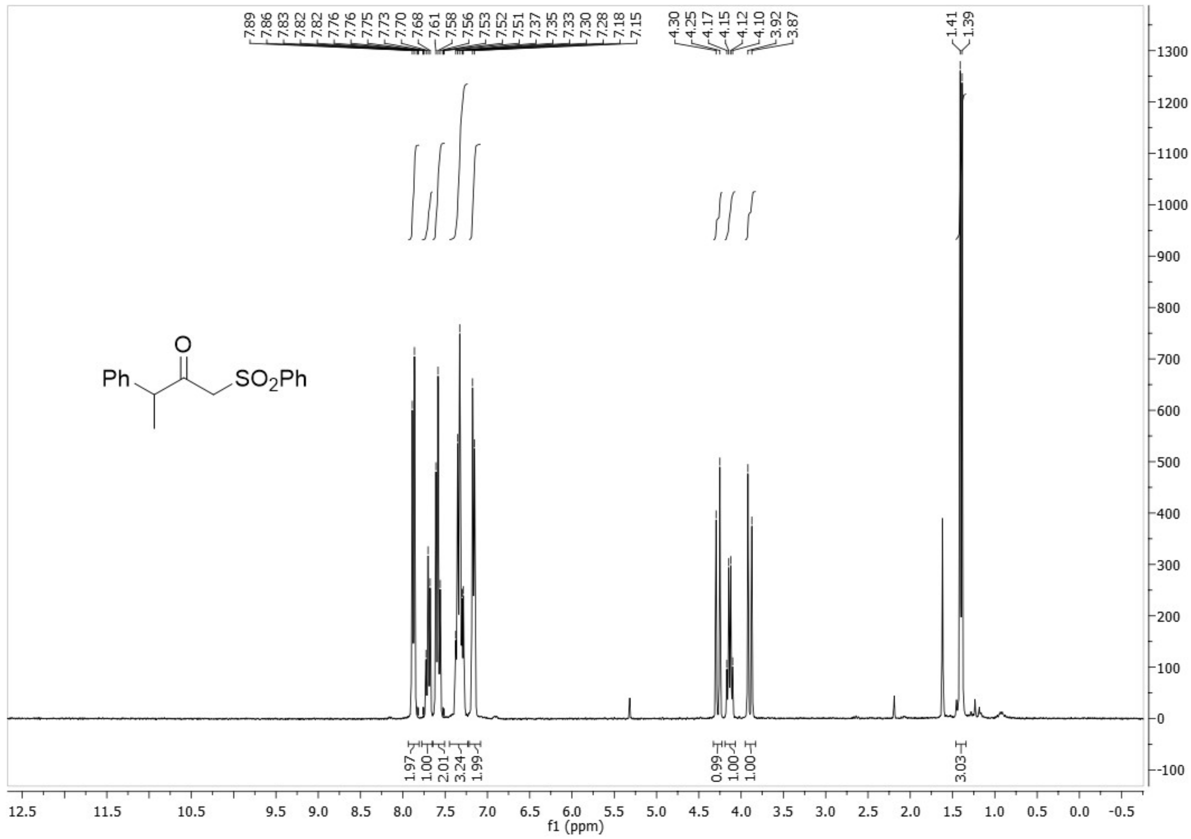

^{13}C NMR (101 MHz, $CDCl_3$)

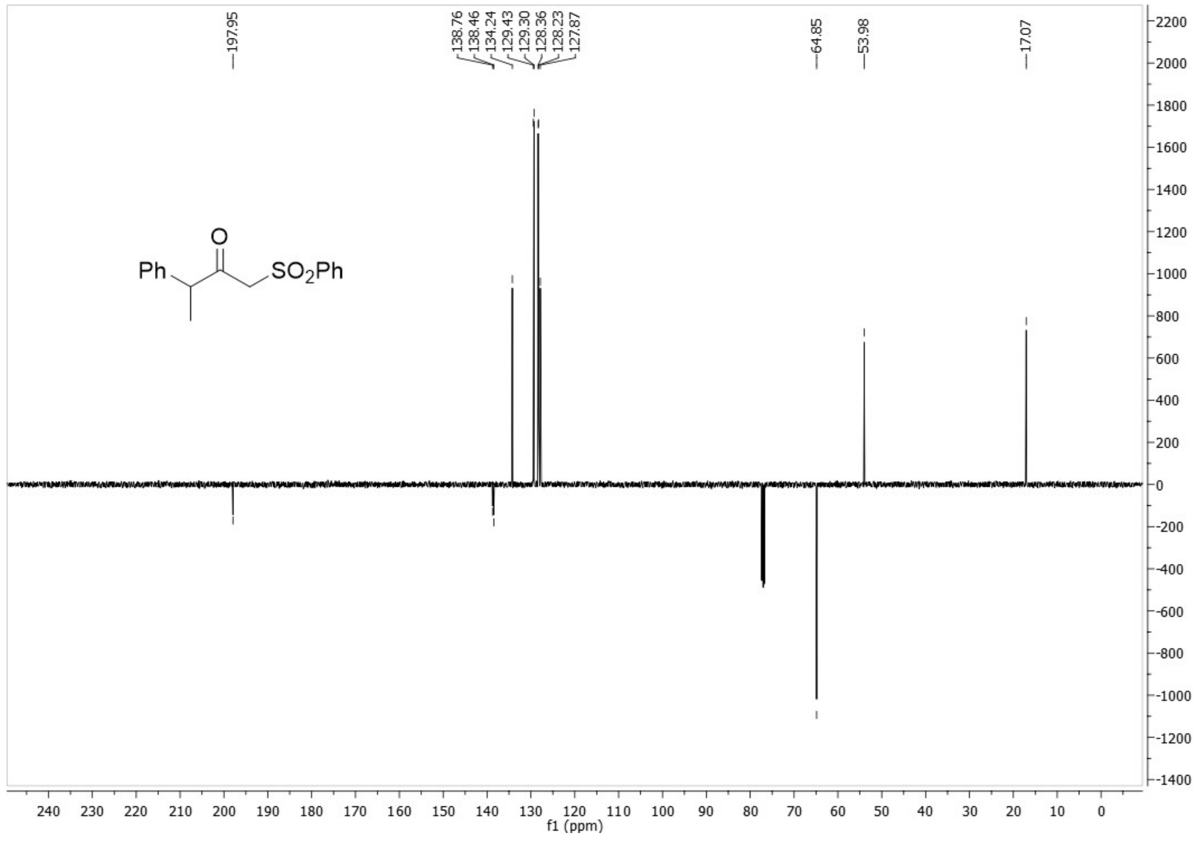

Ketone HPLC of 3-Phenoxy-1-(phenylsulfonyl)butan-2-one.

Clarity - Chromatography SW
DataApex 2006
www.dataapex.com

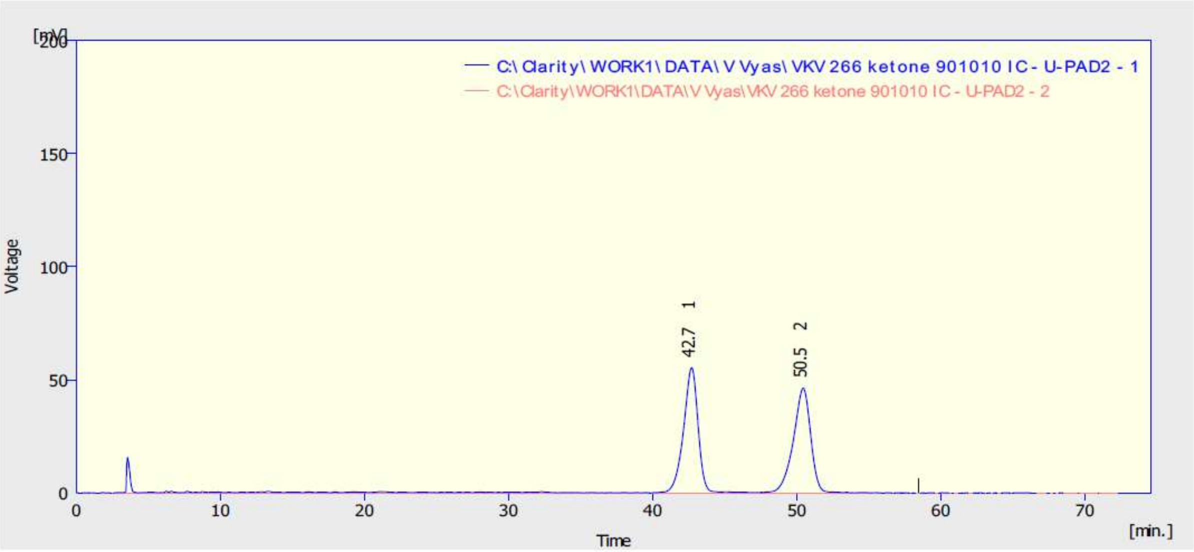

Result Table (Uncal - C:\Clarity\WORK1\DATA\V Vyas\VKV 266 ketone 901010 IC - U-PAD2 - 1)

	Reten. Time [min]	Area [mV.s]	Height [mV]	Area [%]	Height [%]	W05 [min]	Compound Name
1	42.716	3956.235	55.422	49.8	54.4	1.05	
2	50.452	3981.568	46.416	50.2	45.6	1.26	
	Total	7937.803	101.838	100.0	100.0		

1-(Benzo[d]thiazol-2-ylsulfonyl)-3-phenoxypropan-2-one 28

^{1}**H NMR** (400 MHz, CDCl$_3$)

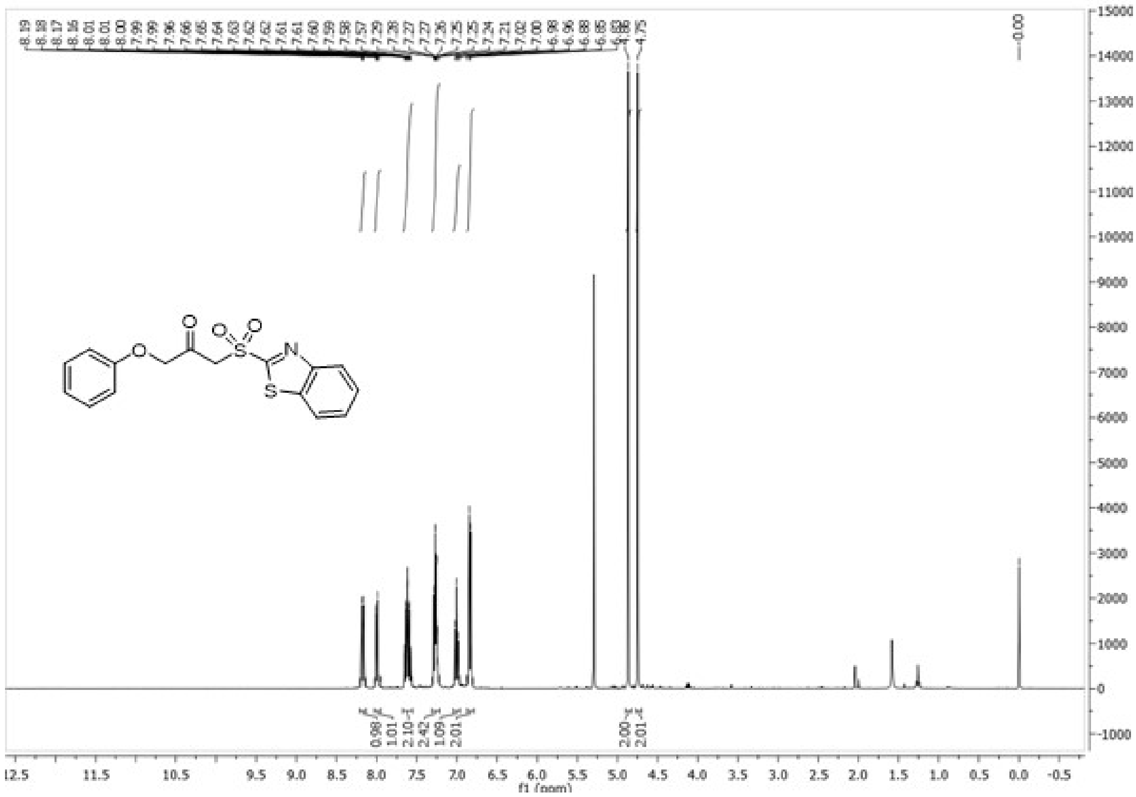

^{13}**C NMR** (101 MHz, CDCl$_3$)

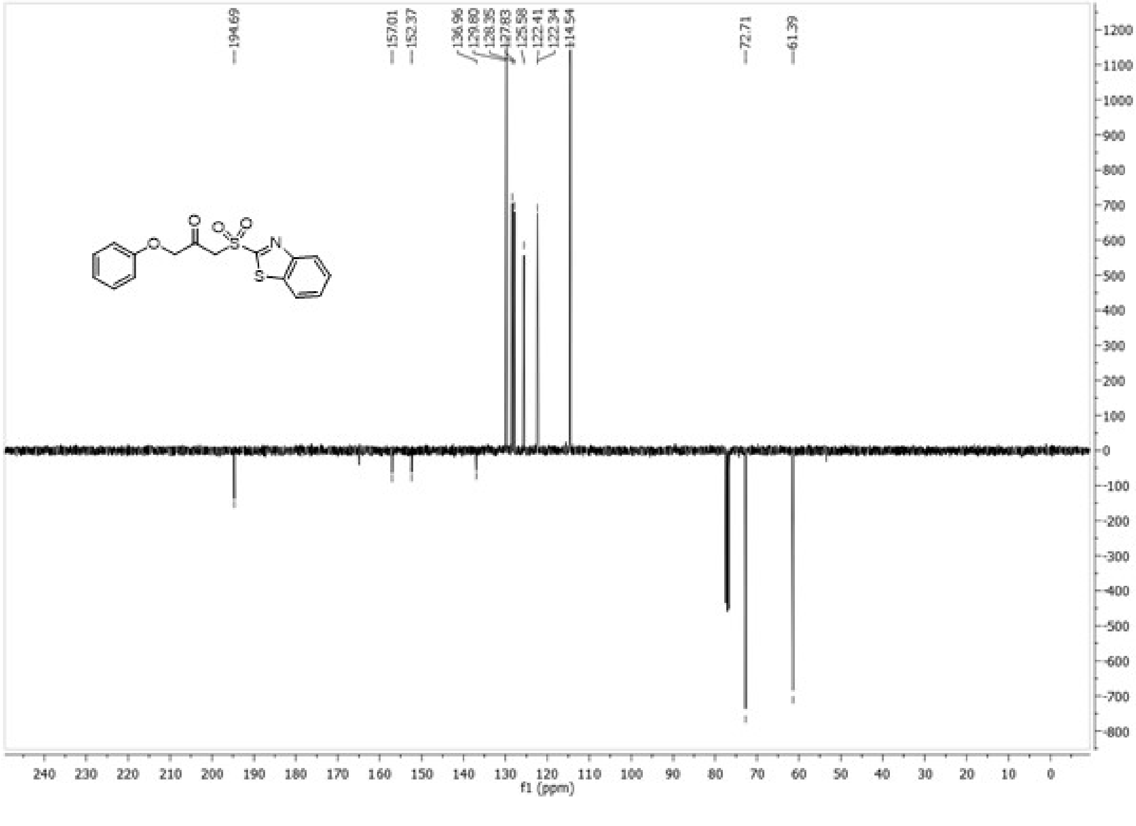

Ketone HPLC of 1-(benzo[d]thiazol-2-ylsulfonyl)-3-phenoxypropan-2-one.

Clarity - Chromatography SW

DataApex 2006

www.dataapex.com

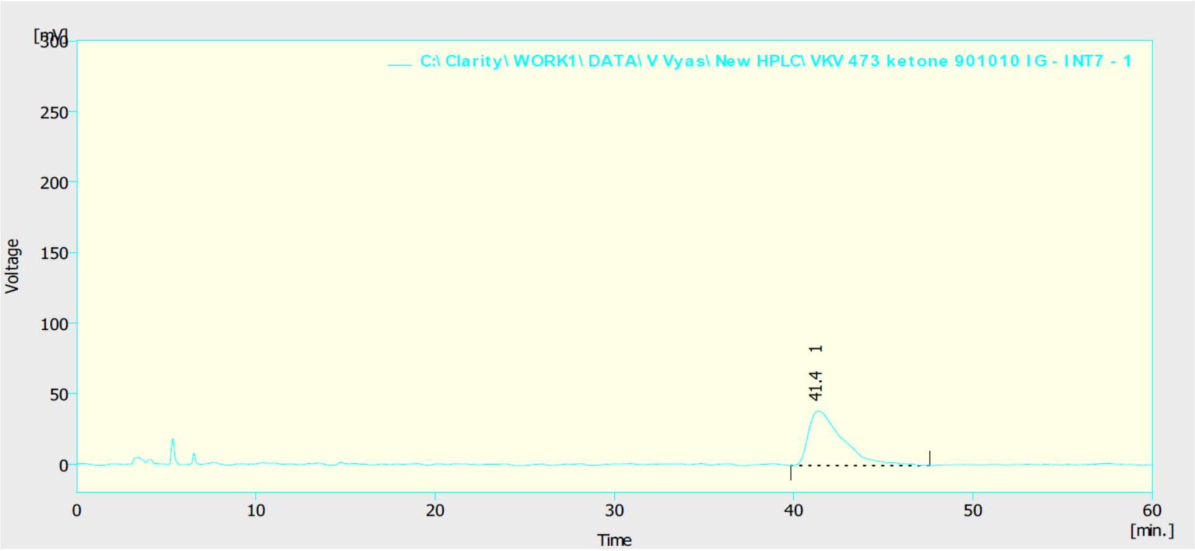

Result Table (Uncal - C:\Clarity\WORK1\DATA\V Vyas\New HPLC\VKV 473 ketone 901010 IG - INT7 - 1)

	Reten. Time [min]	Area [mV.s]	Height [mV]	Area [%]	Height [%]	W05 [min]	Compound Name
1	41.377	5141.341	38.399	100.0	100.0	1.94	
	Total	5141.341	38.399	100.0	100.0		

1-(Benzo[d]thiazol-2-ylsulfonyl)-3-phenoxypropan-2-ol 29

^{1}H NMR (400 MHz, $CDCl_3$)

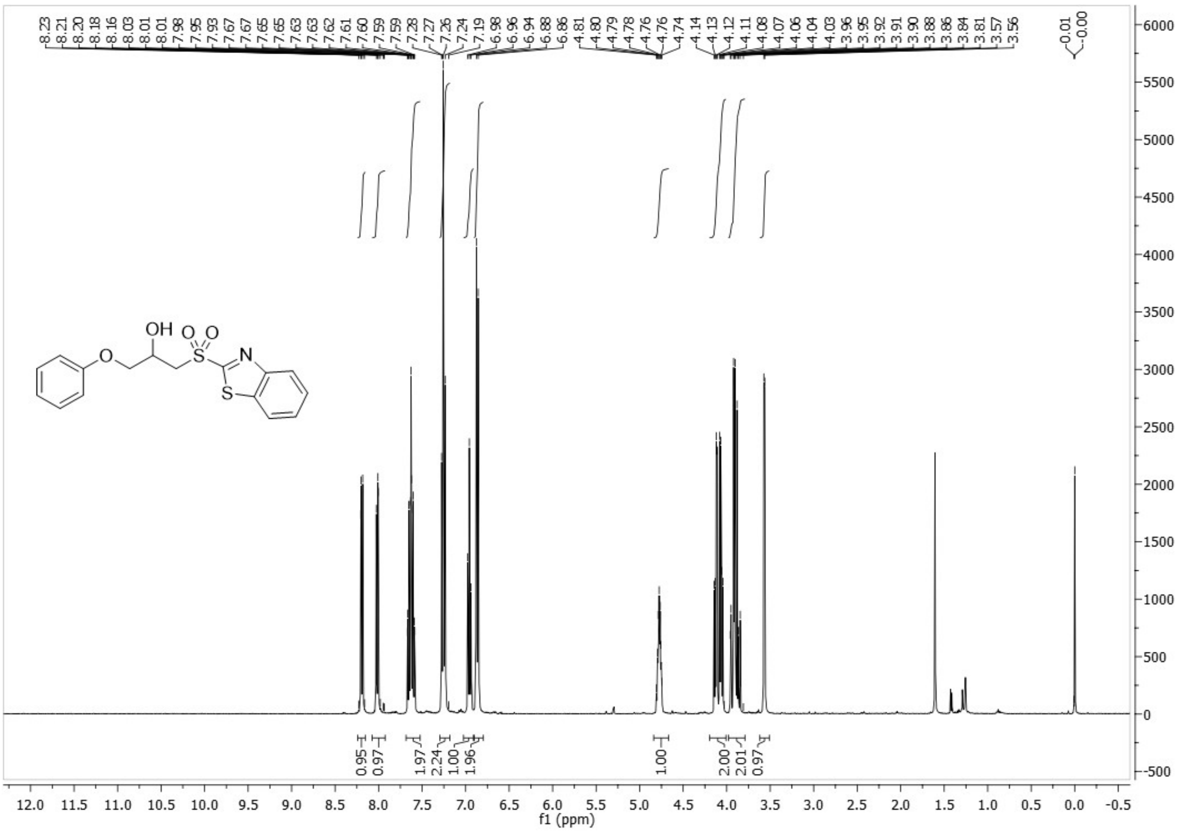

^{13}C NMR (101 MHz, $CDCl_3$)

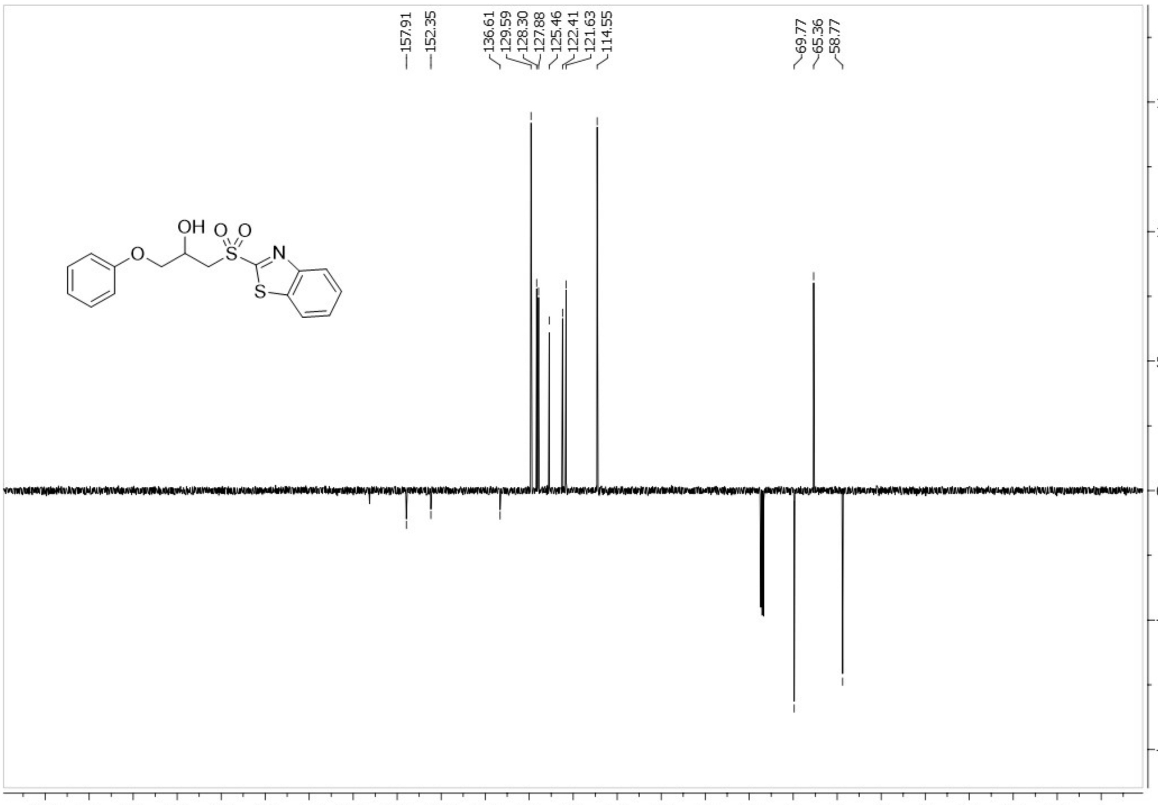

Racemic HPLC of 1-(benzo[d]thiazol-2-ylsulfonyl)-3-phenoxypropan-2-ol.

Clarity - Chromatography SW

DataApex 2006

www.dataapex.com

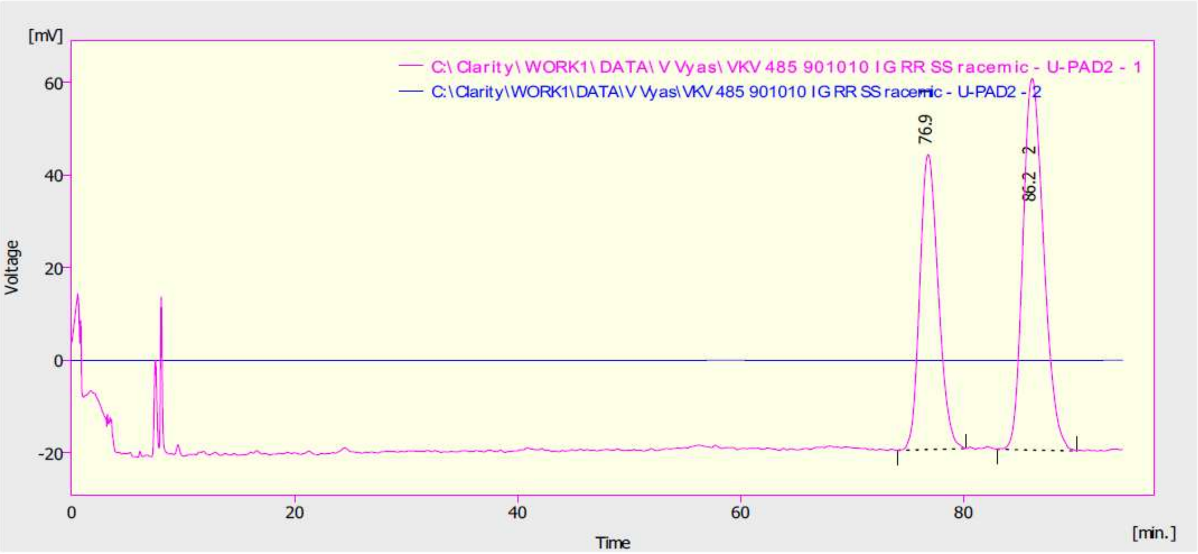

Result Table (Uncal - C:\Clarity\WORK1\DATA\V Vyas\VKV 485 901010 IG RR SS racemic - U-PAD2 - 1)

	Reten. Time [min]	Area [mV.s]	Height [mV]	Area [%]	Height [%]	W05 [min]	Compound Name
1	76.852	7352.031	63.702	41.3	44.3	1.79	
2	86.160	10454.513	80.227	58.7	55.7	2.02	
	Total	17806.544	143.929	100.0	100.0		

HPLC after ATH 1-(benzo[d]thiazol-2-ylsulfonyl)-3-phenoxypropan-2-ol. (100% conversion, 94% ee).

Clarity - Chromatography SW

DataApex 2006

www.dataapex.com

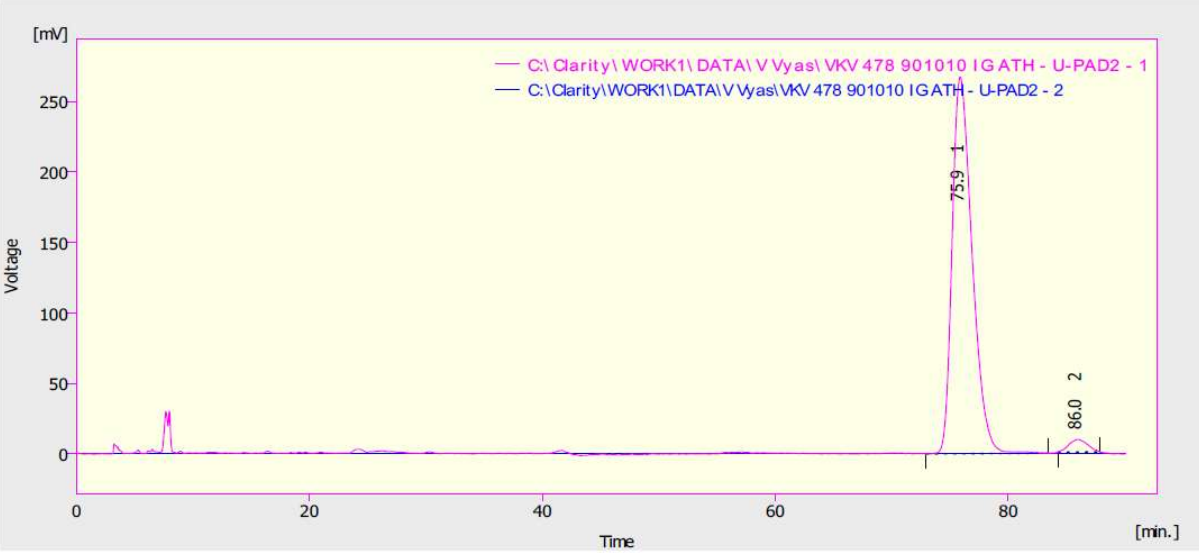

Result Table (Uncal - C:\Clarity\WORK1\DATA\V Vyas\VKV 478 901010 IG ATH - U-PAD2 - 1)

	Reten. Time [min]	Area [mV.s]	Height [mV]	Area [%]	Height [%]	W05 [min]	Compound Name
1	75.872	31919.373	267.304	97.0	96.8	1.83	
2	85.960	989.165	8.908	3.0	3.2	1.84	
	Total	32908.538	276.212	100.0	100.0		

2-((2-((tert-Butyldimethylsilyl)oxy)-3-phenoxypropyl)sulfonyl)benzo[d]thiazole 30

^{1}**H NMR** (400 MHz, $CDCl_3$)

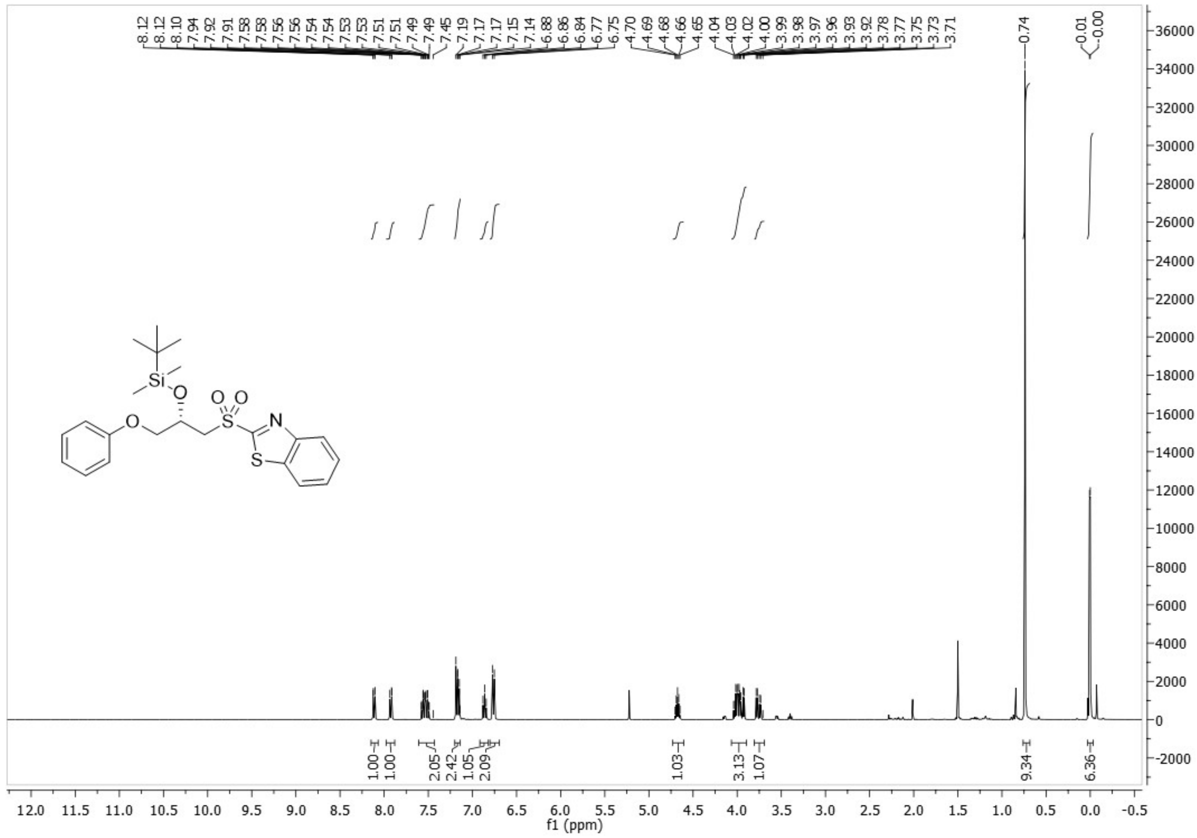

^{13}**C NMR** (101 MHz, $CDCl_3$)

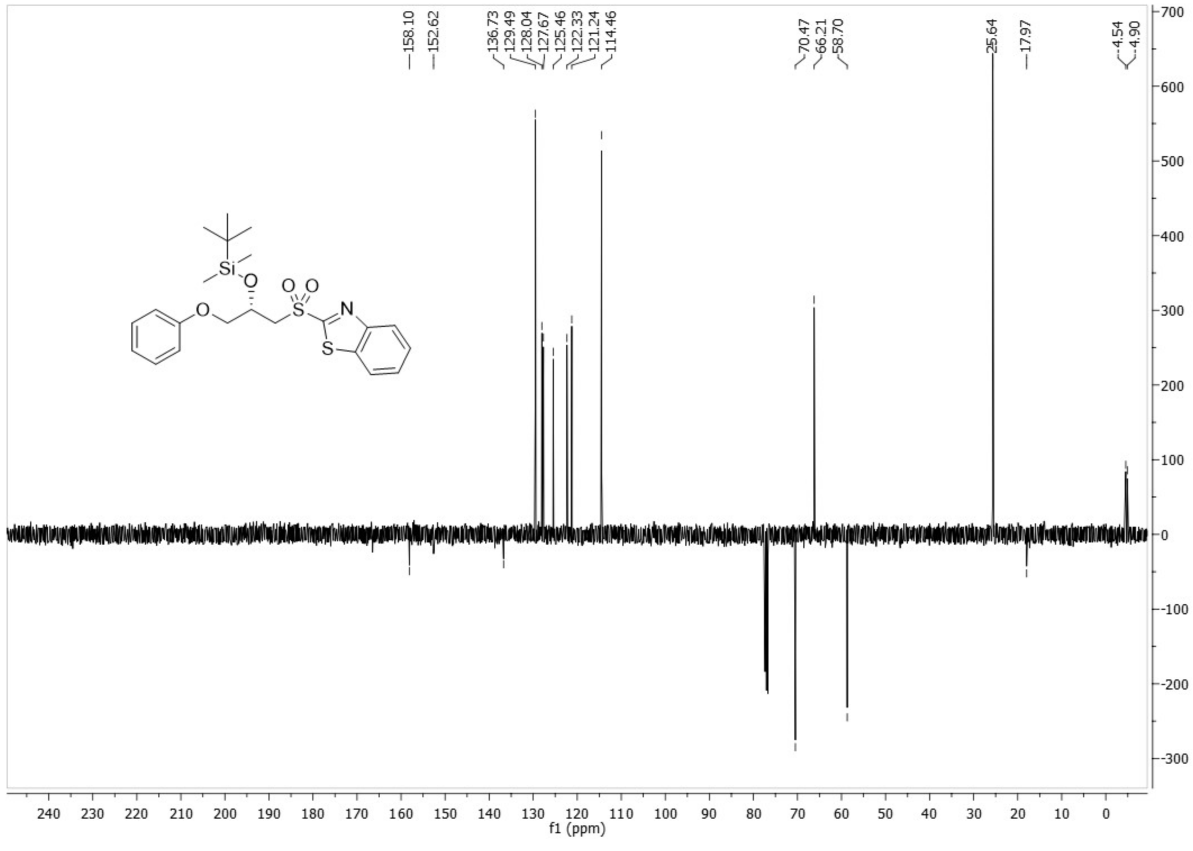

(*R,E*)-1-Phenoxy-4-phenylbut-3-en-2-ol 31

^{1}H NMR (400 MHz, $CDCl_3$)

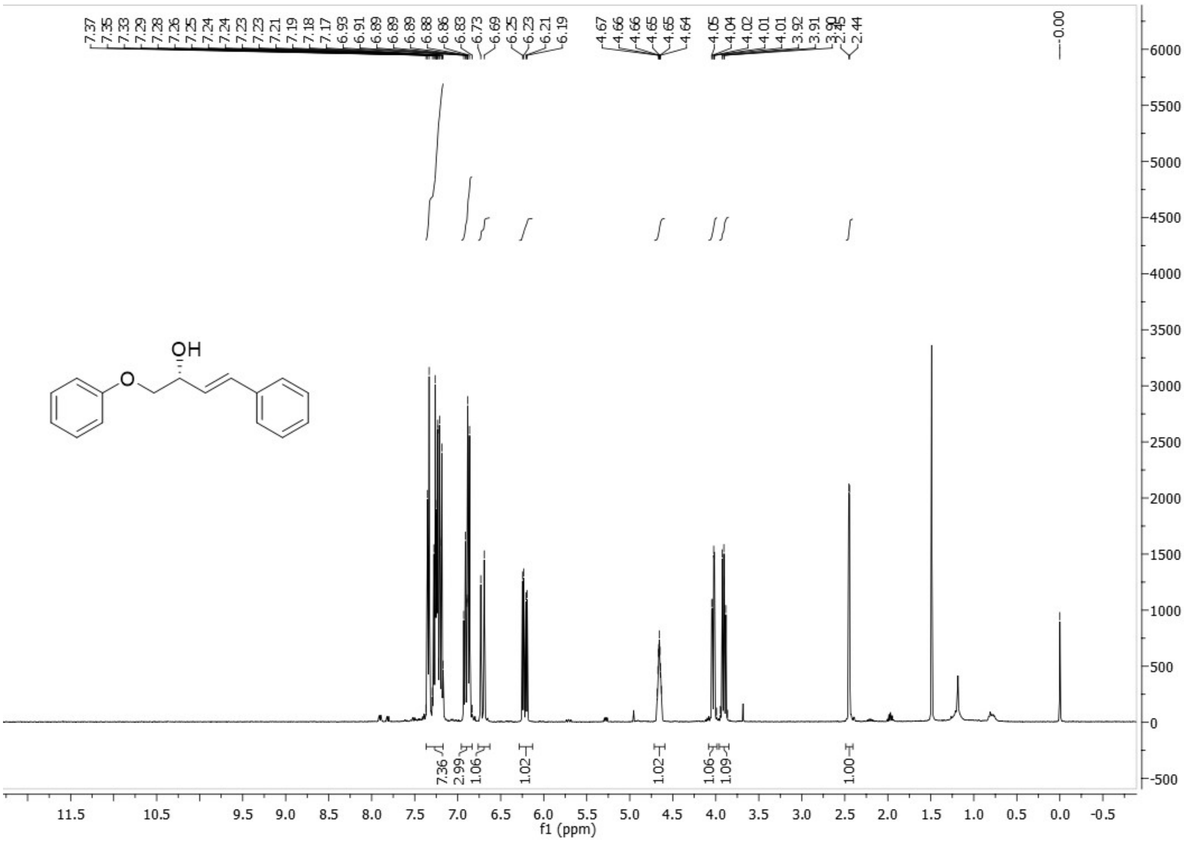

^{13}C NMR (101 MHz, $CDCl_3$)

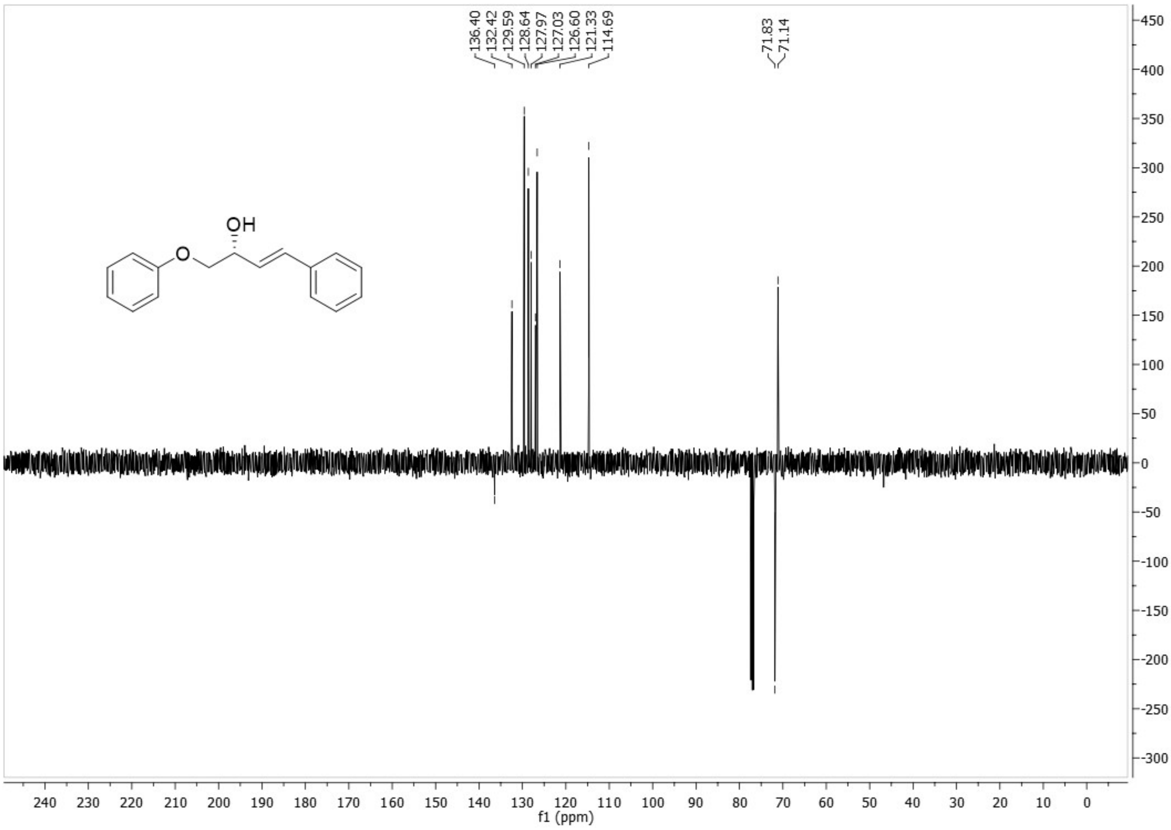

1-Phenoxy-4-phenylbut-3-en-2-ol 31

^{1}H NMR (400 MHz, $CDCl_3$)

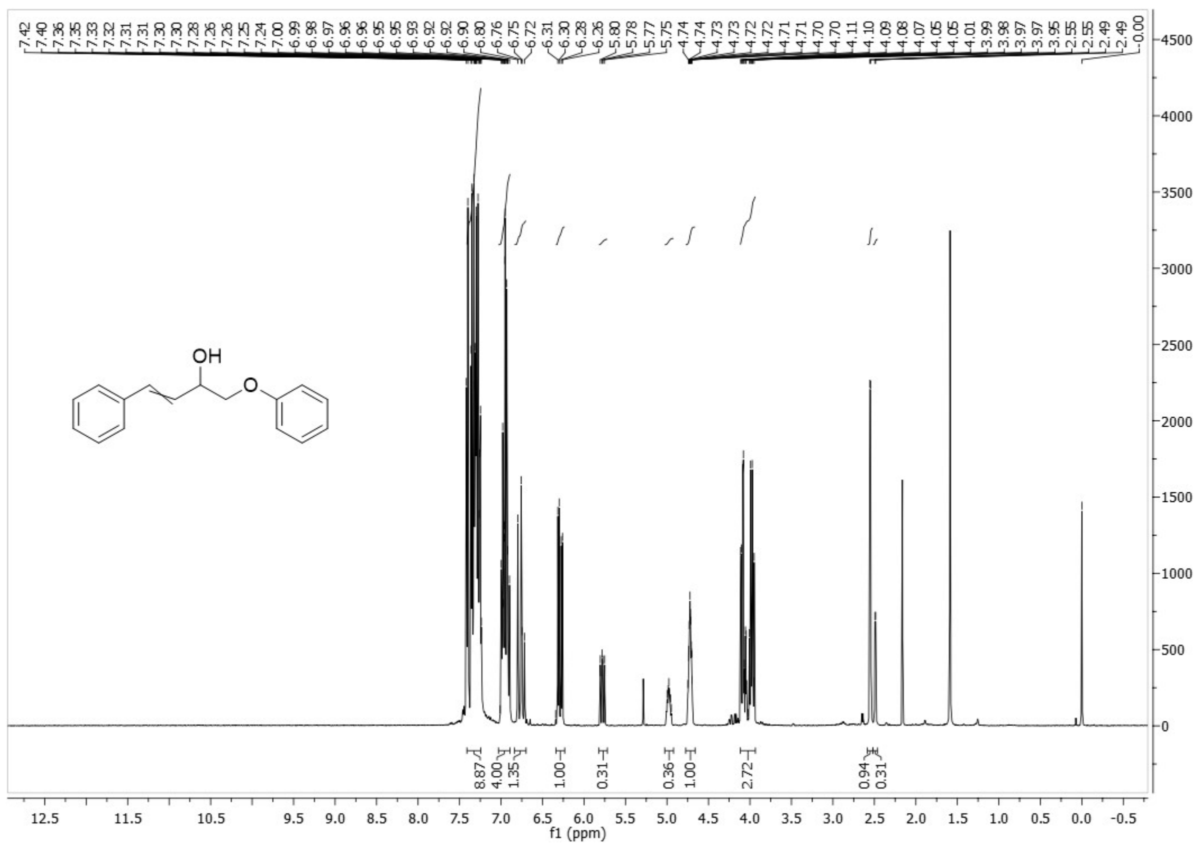

^{13}C NMR (101 MHz, $CDCl_3$)

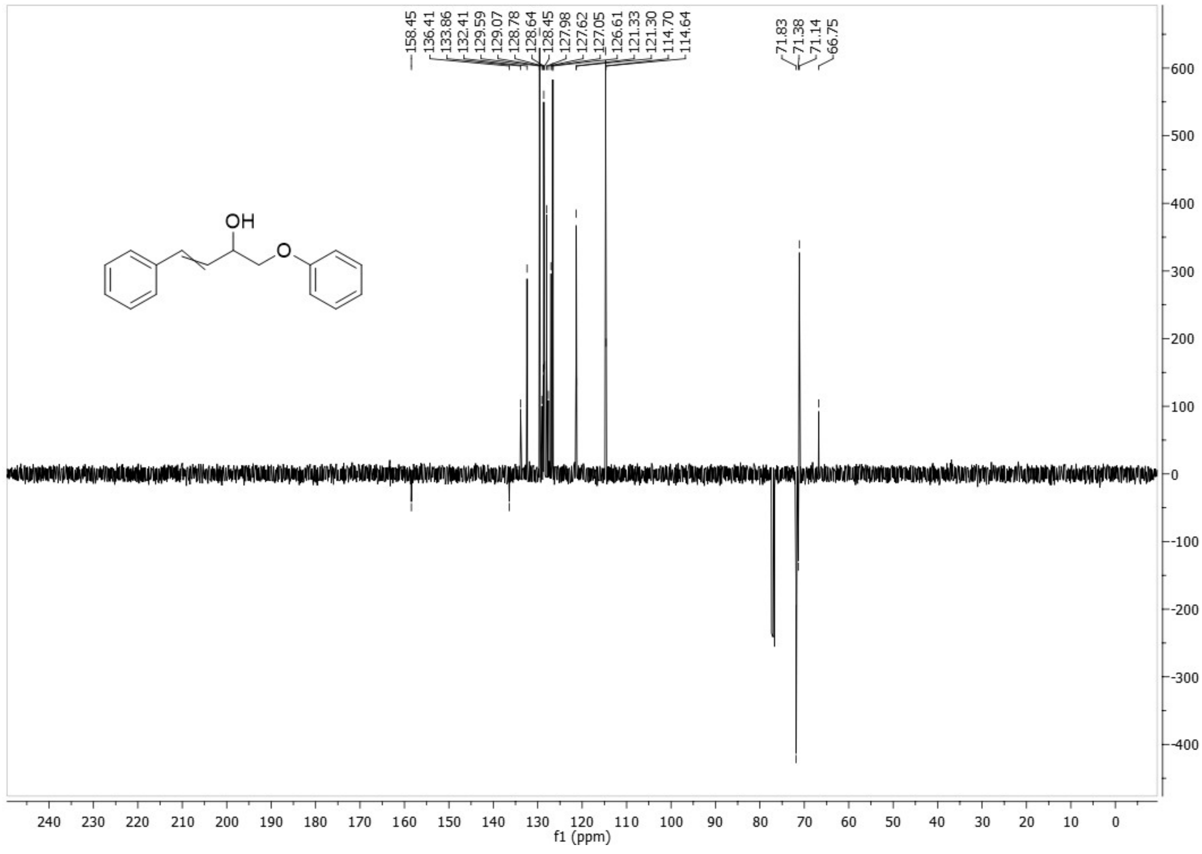

Racemic HPLC of 1-phenoxy-4-phenylbut-3-en-2-ol.

Clarity - Chromatography SW
DataApex 2006
www.dataapex.com

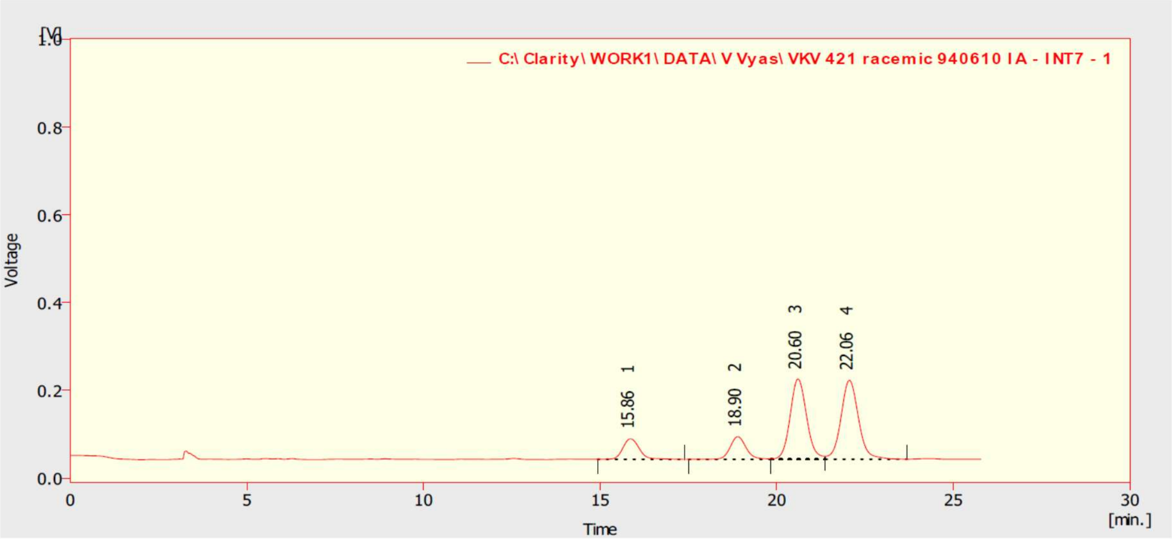

Result Table (Uncal - C:\Clarity\WORK1\DATA\V Vyas\VKV 421 racemic 940610 IA - INT7 - 1)

	Reten. Time [min]	Area [mV.s]	Height [mV]	Area [%]	Height [%]	W05 [min]	Compound Name
1	15.860	1509.867	46.657	9.9	10.1	0.50	
2	18.897	1616.351	51.667	10.6	11.2	0.47	
3	20.603	5922.626	182.884	38.8	39.7	0.50	
4	22.057	6209.786	179.750	40.7	39.0	0.52	
	Total	15258.630	460.958	100.0	100.0		

HPLC after Julia-Kocienski addition (*R*,*E*)-1-phenoxy-4-phenylbut-3-en-2-ol. (100% conversion, dr ratio 98.8(*trans*):1.2(*cis*), 90.8% ee, trans isomer d2, >99.9% ee cis isomer).

Clarity - Chromatography SW
DataApex 2006
www.dataapex.com

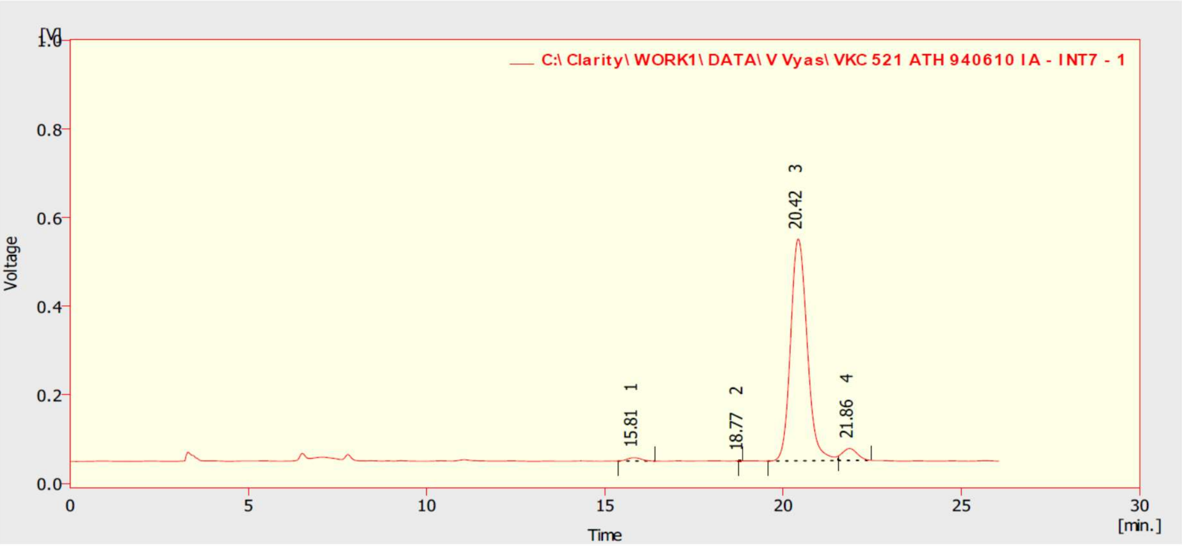

Result Table (Uncal - C:\Clarity\WORK1\DATA\V Vyas\VKC 521 ATH 940610 IA - INT7 - 1)

	Reten. Time [min]	Area [mV.s]	Height [mV]	Area [%]	Height [%]	W05 [min]	Compound Name
1	15.810	203.195	7.541	1.2	1.4	0.43	
2	18.767	0.129	0.033	0.0	0.0	0.02	
3	20.423	16207.786	500.251	94.3	93.5	0.50	
4	21.860	784.706	26.959	4.6	5.0	0.50	
	Total	17195.816	534.784	100.0	100.0		

HPLC after ATH 1-phenoxy-4-phenylbut-3-en-2-ol. (100% conversion, 54.2% ee for d1).

Clarity - Chromatography SW

DataApex 2006

www.dataapex.com

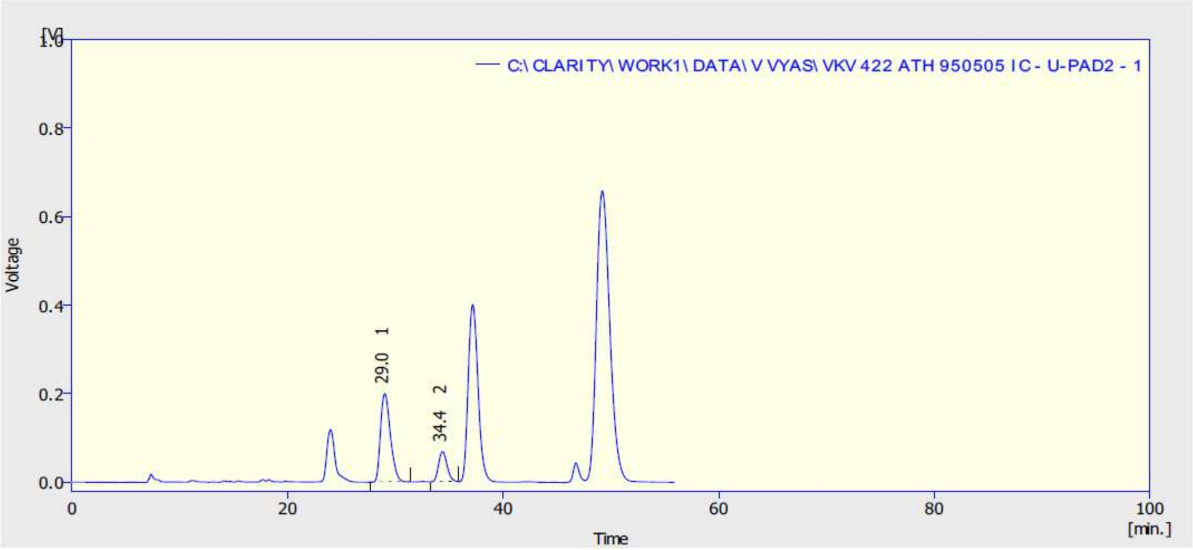

Result Table (Uncal - C:\CLARITY\WORK1\DATA\V VYAS\VKV 422 ATH 950505 IC - U-PAD2 - 1)

	Reten. Time [min]	Area [mV.s]	Height [mV]	Area [%]	Height [%]	W05 [min]	Compound Name
1	29.020	12776.856	199.904	77.1	74.5	0.99	
2	34.388	3794.447	68.382	22.9	25.5	0.87	
	Total	16571.303	268.286	100.0	100.0		

HPLC after ATH 1-phenoxy-4-phenylbut-3-en-2-ol. (100% conversion, 37.6% ee for d2).

Clarity - Chromatography SW

DataApex 2006

www.dataapex.com

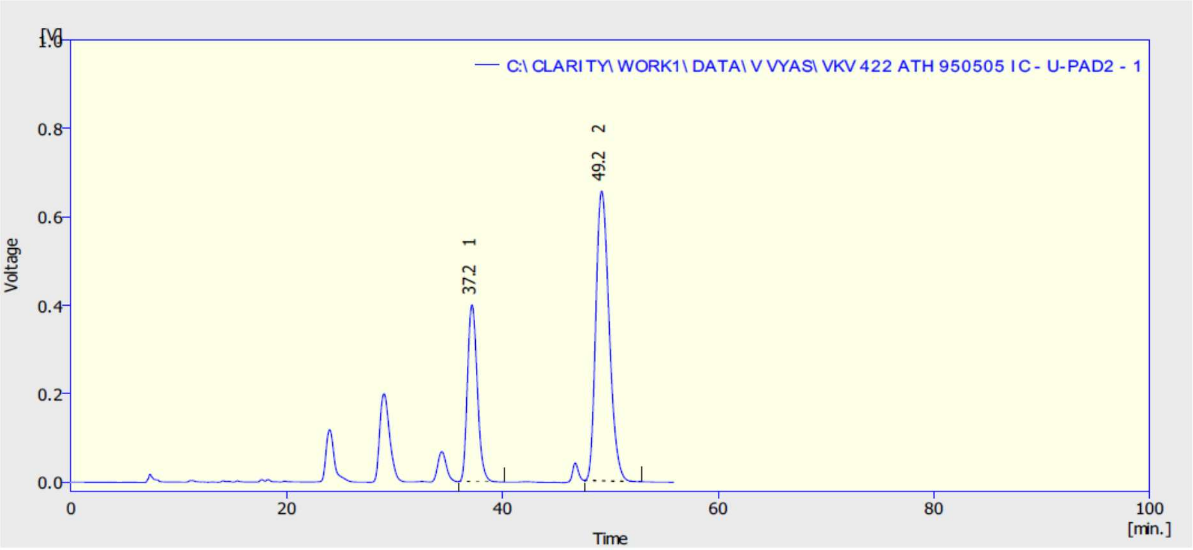

Result Table (Uncal - C:\CLARITY\WORK1\DATA\V VYAS\VKV 422 ATH 950505 IC - U-PAD2 - 1)

	Reten. Time [min]	Area [mV.s]	Height [mV]	Area [%]	Height [%]	W05 [min]	Compound Name
1	37.180	25500.033	400.486	31.2	37.9	1.00	
2	49.204	56188.045	655.200	68.8	62.1	1.34	
	Total	81688.078	1055.686	100.0	100.0		

X-ray crystal structure details.

Determination of absolute configuration of 11a (CCDC 1975604.

(*S*)-1-Cyclohexyl-2-(phenylsulfonyl)ethan-1-ol **11a** (48 mg, 0.20 mmol, 1.0 equiv) was dissolved in DCM (2 mL) at rt in a dry schlenk tube under a nitrogen atmosphere. DMAP (a few crystals) and (R)-(+)-α-Methylbenzyl isocyanate (30 µL, 0.18 mmol, 1.0 equiv) were added. The reaction mixture was stirred overnight. At the end of this time the isocyanate adduct was purified by column chromatography on silica gel (n-hexane:EtOAc 85/15) as a white solid (24 mg, 0.06 mmol, 29%).. Procedure adapted from Simpson, A.F.; Bodkin, C. D.; Butts, C. P.; Armitage, M. A.; Gallagher, T. *J. Chem. Soc., Perkin Trans. 1*, 2000, 3047-3054

The crystal of obtained adduct was grown from DCM/Hexane.

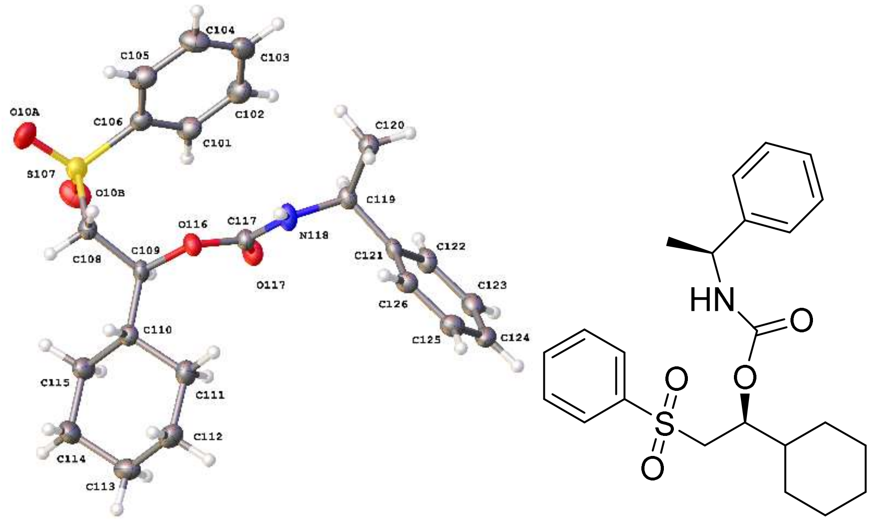

Solid state structure of one of the crystalographically independent but chemically identical molecules in the asymmetric unit of vv1 with atom labelling and thermal ellipsoids drawn at 50% probability level

Crystal structure determination of [vv1_twin1_hklf5]

The asymmetric unit contains two crystallographically independent but chemically identical molecules in the asymmetric unit, twice this in the unit cell.

The twin ratio refined to 0.5071(11) to 0.4929(11) [that's 51:49]. The two twin domains were related by a Rotation = 179.9626 deg around the following vectors:

Reciprocal space (hkl): -0.0001 0.0003 1.0000

Direct space (uvw)   : 0.1548 0.0001 0.9879

The molecule has crystalised in a chiral space group and after the twin refinement, the Flack parameter is smallish with a small error. As there is already a chiral center of known handedness in the molecule from the methylbenzyl carbamate the Flack is not essential for confirming the absolute configuration.

Flack x: 0.010(14) (Shelx2018)

Hooft y: -0.016(8) (Olex2)

The NHs were located in a difference map but placed at calculated positions for the refinement. They form short contacts tabulated below.

Specified hydrogen bonds (with esds except fixed and riding H)

D-H	H...A	D...A	<(DHA)	
0.88	2.34	3.178(7)	160.3	N118-H118...O117_$1
0.88	2.32	3.144(7)	155.4	N218-H218...O217_$1

Experimental

Single crystals of were grown from vapour diffusion of n-hexane into a dichloromethane solution of the compound over several days.A suitable crystal was selected and mounted on a glass fibre with Fomblin oil and placed on a Rigaku Oxford Diffraction SuperNova diffractometer with a duel source (Cu at zero) equipped with an AtlasS2 CCD area detector. The crystal was kept at 150(2) K during data collection.

The harvesting of the spots and integration was performed with the twin module in the CrysAlisPro software package [1]. Using Olex2 [2], the structure was solved using the reflections from the maJor twin component with ShelXT [3] structure solution program using Intrinsic Phasing and the reflection data for the two components refined in the hklf5 format with the ShelXL [4] refinement package using Least Squares minimization

1. Agilent (2014). CrysAllisPRO, Agilent Technologies Ltd., Yamton, Oxfordshire, UK.
2. Dolomanov, O.V., Bourhis, L.J., Gildea, R.J, Howard, J.A.K. & Puschmann, H. (2009), J. Appl. Cryst. 42, 339-341.
3. Sheldrick, G.M. (2015). Acta Cryst. A71, 3-8.
4. Sheldrick, G.M. (2015). Acta Cryst. C71, 3-8.

Crystal Data for $C_{23}H_{29}NO_4S$ (M =415.53 g/mol): monoclinic, space group P2$_1$ (no. 4), a = 5.3568(2) Å, b = 26.0098(5) Å, c = 15.2286(2) Å, β = 93.300(2)°, V = 2118.27(9) Å^3, Z = 4, T = 150(2) K, μ(CuKα) = 1.595 mm^{-1}, $Dcalc$ = 1.303 g/cm^3, 15332 reflections measured

(5.814° ≤ 2Θ ≤ 148.212°), 15332 unique (R_{int} = ?(no meaningful number for this as twinned data), R_{sigma} = 0.0667) which were used in all calculations. The final R_1 was 0.0419 (I > 2σ(I)) and wR_2 was 0.1181 (all data).

Table 1 Crystal data and structure refinement for vv1_twin1_hklf5 (Figure 5B).

Identification code	vv1_twin1_hklf5
Empirical formula	$C_{23}H_{29}NO_4S$
Formula weight	415.53
Temperature/K	150(2)
Crystal system	monoclinic
Space group	$P2_1$
a/Å	5.3568(2)
b/Å	26.0098(5)
c/Å	15.2286(2)
α/°	90
β/°	93.300(2)
γ/°	90
Volume/Å^3	2118.27(9)
Z	4
ρ_{calc}g/cm^3	1.303
μ/mm^{-1}	1.595
F(000)	888.0
Crystal size/mm^3	0.08 × 0.01 × 0.01 colourless block
Radiation	CuKα (λ = 1.54184)
2Θ range for data collection/°	5.814 to 148.212
Index ranges	$-6 \leq h \leq 6$, $-32 \leq k \leq 32$, $-18 \leq l \leq 18$
Reflections collected	15332
Independent reflections	15332 [R_{int} = ?, R_{sigma} = 0.0667]
Data/restraints/parameters	15332/1/527

Goodness-of-fit on F^2	0.958
Final R indexes [I>=2σ (I)]	R_1 = 0.0419, wR_2 = 0.1144
Final R indexes [all data]	R_1 = 0.0535, wR_2 = 0.1181
Largest diff. peak/hole / e $Å^{-3}$	0.22/-0.40
Flack parameter	0.010(14)

Crystal structure of (*1S,2S*)-3-phenoxy-1-phenyl-1-(phenylsulfonyl)propan-2-ol 20c, CCDC 1975605.

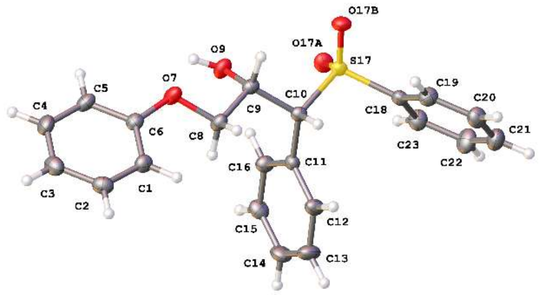

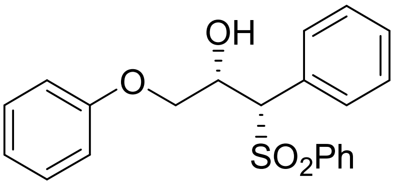

Crystal structure determination of 23 [vv3]

The asymmetric unit contains the sulphone, there are four in the unit cell. The OH was located in a difference map but refined with distance restraints. It forms a short intermolecular contact tabulated below

Specified hydrogen bonds (with esds except fixed and riding H)

D-H	H...A	D...A	<(DHA)	
0.84	2.25	2.6753(17)	111.3	O9-H9A...O7

The Flack parameter as a measure of the confidence you can have in the handedness of the crystal refined was

Flack x: 0.001(3) Shelx 2018

Hooft y: -0.009(2) Olex2

This is small with a small error so you can be confident in the assignment of the handedness of the crystal measured.

Experimental

Single crystals of were grown from vapour diffusion of n-hexane into a dichloromethane solution of the compound over several days A suitable crystal was selected and mounted on a glass fibre with Fomblin oil and placed on a Rigaku Oxford Diffraction SuperNova diffractometer with a duel source (Cu at zero) equipped with an AtlasS2 CCD area detector. The crystal was kept at 150(2) K during data collection. Using Olex2 [1], the structure was solved with the ShelXT [2] structure solution program using Intrinsic Phasing and refined with the ShelXL [3] refinement package using Least Squares minimisation.

1. Dolomanov, O.V., Bourhis, L.J., Gildea, R.J, Howard, J.A.K. & Puschmann, H. (2009), J. Appl. Cryst. 42, 339-341.
2. Sheldrick, G.M. (2015). Acta Cryst. A71, 3-8.
3. Sheldrick, G.M. (2015). Acta Cryst. C71, 3-8.

Crystal Data for $C_{21}H_{20}O_4S$ (M =368.43 g/mol): orthorhombic, space group $P2_12_12_1$ (no. 19), a = 5.67747(2) Å, b = 16.22744(6) Å, c = 19.54508(7) Å, V = 1800.703(12) Å^3, Z =

4, T = 150(2) K, μ(CuKα) = 1.796 mm^{-1}, $Dcalc$ = 1.359 g/cm^3, 51275 reflections measured (7.08° ≤ 2Θ ≤ 147.08°), 3630 unique (R_{int} = 0.0375, R_{sigma} = 0.0123) which were used in all calculations. The final R_1 was 0.0224 (I > 2σ(I)) and wR_2 was 0.0590 (all data).

Table 1 Crystal data and structure refinement for vv3.

Identification code	vv3
Empirical formula	$C_{21}H_{20}O_4S$
Formula weight	368.43
Temperature/K	150(2)
Crystal system	orthorhombic
Space group	$P2_12_12_1$
a/Å	5.67747(2)
b/Å	16.22744(6)
c/Å	19.54508(7)
α/°	90
β/°	90
γ/°	90
Volume/Å^3	1800.703(12)
Z	4
ρ_{calc}g/cm^3	1.359
μ/mm^{-1}	1.796
F(000)	776.0
Crystal size/mm^3	0.28 × 0.1 × 0.08 colourless block
Radiation	CuKα (λ = 1.54184)
2Θ range for data collection/°	7.08 to 147.08
Index ranges	-7 ≤ h ≤ 7, -20 ≤ k ≤ 20, -23 ≤ l ≤ 24
Reflections collected	51275
Independent reflections	3630 [R_{int} = 0.0375, R_{sigma} = 0.0123]
Data/restraints/parameters	3630/0/236
Goodness-of-fit on F^2	1.048
Final R indexes [I>=2σ (I)]	R_1 = 0.0224, wR_2 = 0.0590

Final R indexes [all data]	R_1 = 0.0225, wR_2 = 0.0590
Largest diff. peak/hole / e Å^{-3}	0.14/-0.31
Flack parameter	0.001(3)
